# Synthesis, Conformal Analysis, and Antibody Binding of *Staphylococcus aureus* Capsular Polysaccharide Type 5 Oligosaccharides

**DOI:** 10.1002/anie.202511378

**Published:** 2025-08-18

**Authors:** Kitt E. Østerlid, Sizhe Li, Luca Unione, Linda Del Bino, Charlotte Sorieul, Filippo Carboni, Francesca Berni, Sara Bertuzzi, Bob van Puffelen, Ana Arda, Herman S. Overkleeft, Gijsbert A. van der Marel, Maria Rosaria Romano, Jesús Jiménez‐Barbero, Roberto Adamo, Jeroen D. C. Codée

**Affiliations:** ^1^ Leiden Institute of Chemistry Leiden University Einsteinweg 55 Leiden 2333 CC The Netherlands; ^2^ GSK Siena Via Fiorentina, 1 SI Siena 53100 Italy; ^3^ Center for Cooperative Research in Biosciences (CIC bioGUNE) Basque Research and Technology Alliance (BRTA) Derio,Bizkaia 48160 Spain; ^4^ Ikerbasque, Basque Foundation for Science Bilbao 48009 Spain; ^5^ Department of Organic & Inorganic Chemistry Faculty of Science and Technology University of the Basque Country EHU‐UPV Leioa,Bizkaia 48940 Spain; ^6^ Centro de Investigacion Biomedica En Red de Enfermedades Respiratorias Madrid 28029 Spain

**Keywords:** Conformational analysis, Oligosaccharides, Stereoselectivity, Synthetic vaccines

## Abstract

*Staphylococcus aureus* is one of the most prominent pathogens responsible for life‐threatening hospital acquired infections. Most clinical isolates belong to serotype 5 or 8, which express unique capsular polysaccharides (CP), composed of the rare *N*‐acetyl‐β‐d‐mannosaminuronic acid (β‐d‐ManNAcA), *N*‐acetyl‐α‐l‐fucosamine (α‐l‐FucNAc) and *N*‐acetyl‐β‐d‐fucosamine (β‐d‐FucNAc) that can be used for the development of conjugate vaccines. Different acetylation patterns of CP5 create microheterogeneous polymers, carrying partial zwitterionic character, which may be important for immunological activity. We here report on the assembly of a set of conjugation‐ready CP5 oligosaccharides, ranging in length from trisaccharides to nonasaccharides. The developed protecting group strategy has allowed the incorporation of *N‐*acetyl, ‐NH_3_^+^ and *O*‐acetyl groups. The reported syntheses offer solutions for the construction of the challenging *cis*‐glycosidic linkages, the incorporation of many different functional groups and the installation of an appropriate linker for future conjugation purposes. Conformational analysis of the *O*‐acetylated oligomers has revealed a distinctive linear conformation with the repeating units (RUs) being flipped ∼180° with respect to the flanking RUs. Binding studies with CP5‐antibodies revealed the trisaccharide to be too short for relevant binding, while the hexa‐ and nonasaccharides exhibited strong binding. The l‐FucNAc acetyl esters and d‐FucNAc acetamides were shown to be crucial for binding.

## Graphical abstract

A set of conjugation‐ready *Staphylococcus aureus* serotype 5 oligosaccharides has been generated using a stereoselective [3 + 3*n*] glycosylation strategy. The structures differ in chain length (up to three repeating units) and *O*‐ and *N‐*acetylation pattern. The oligomers adopt a unique extended shape with hydrophobic patches formed by the *O/N*‐acetyl groups. The acetylated hexasaccharide is revealed as a promising epitope for vaccine development.

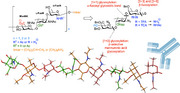

## Introduction

**Figure 1:**
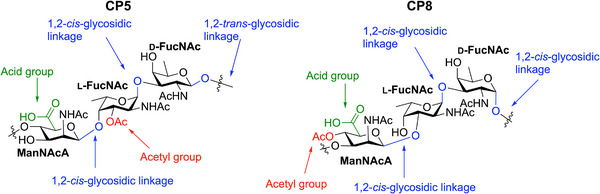
Schematic representation of the repeating unit of CP5 and CP8.

*Staphylococcus aureus* (*S. aureus*) is a commensal Gram‐positive bacterium that is part of our healthy microbiota. However, it can cause a variety of infections ranging from minor skin infections and abscesses to heart valve infections (endocarditis), bone infections (osteomyelitis), lung infections (pneumonia), meningitis, and deadly bloodstream infections (bacteremia and septic shock).^[^ ^1^, ^2^ ^]^ Especially newborns, elderly, immunocompromised, post‐surgical and dialysis patients are at risk. The general treatment for *S. aureus* infections involves the use of antibiotics,^[^ ^3^ ^]^ but due to the emergence of methicillin‐resistant *S. aureus* (MRSA) strains, that have acquired resistance to virtually all types of antibiotics,^[^ ^4^ ^]^ *S. aureus* poses a major health threat and new approaches to combat this remarkably adaptive bacterium is imperative.^[^ ^5^, ^6^, ^7^ ^]^ Attention has been focused on the development of different immunization strategies in the past several years.^[^ ^8^ ^]^ Bacterial capsular polysaccharides (CP) have been used in the generation of various very successful antibacterial vaccines. *S. aureus* expresses unique glycans^[^ ^9^ ^]^ that are important for the virulence and pathogenicity of the bacterium^[^ ^10^, ^11^ ^]^ and that may be considered as potential antigen candidates for the generation of conjugate vaccines.^[^ ^12^, ^13^ ^]^ CPs can be found at the outer layer of encapsulated bacteria. They are anchored to the cell wall by covalent attachment to the peptidoglycan layer and can be built up of various monosaccharides to form large linear or branched polymers. The diversity of these compounds is enormous as they can be composed of different monosaccharides that are interconnected through different glycosidic linkages, featuring varying *N*‐ and *O*‐acetylation patterns, and further modified with pyruvic acid ketals, phosphor mono‐ or diesters and lactic acids, among others.^[^ ^14^ ^]^ Thirteen different *S. aureus* serotypes have been identified to date based on different capsular polysaccharides. CP type 5 (CP5), CP type 1 (CP1), and CP type 8 (CP8) represent the most studied strains,^[^ ^15^, ^16^ ^]^ and CP5 and CP8 comprise the majority of the clinical isolates.^[^ ^16^ ^]^ CP5 and CP8 are very similar in chemical structure as they consist of the same three rare monosaccharides, but differ in glycosidic linkages and *O*‐acetylation patterns. CP5 was first isolated in 1987 from the *S. aureus* Reynolds strain,^[^ ^17^ ^]^ and its structure was elucidated through efforts of Vann,^[^ ^18^ ^]^ Moreau,^[^ ^19^ ^]^ and Jones.^[^ ^20^ ^]^ The structure has been established to be →4)‐*O*‐(2‐acetamido‐2‐deoxy‐β‐d‐mannopyranosyl uronic acid‐1→4)‐*O*‐(2‐acetamido‐3‐*O*‐acetyl‐2‐deoxy‐α‐l‐fucopyranosyl‐1→3)‐2‐acetamido‐2‐deoxy‐β‐d‐fucopyranosyl‐(1→. The *O*‐acetyl was found on the C‐3‐OH of l‐FucNAc residue, as shown in Figure 1, differing from CP8, which has the *O*‐acetyl on the d‐ManNAcA. Structural studies have indicated that *O*‐acetylation at this position occurs for 90 to 100%.^[^ ^21^ ^]^ It has also been reported that partial de‐*N*‐acetylation may occur,^[^ ^22^ ^]^ and this provides the CP with partial zwitterionic character, which may confer peculiar features for the immunogenicity of the CP. Zwitterionic polysaccharides (ZPs) represent an unique class of bacterial polysaccharides as they can elicit an adequate immune response as stand‐alone entities.^[^ ^23^, ^24^, ^25^ ^]^ That is, they do not have to be conjugated to a carrier protein—as opposed to neutral or anionic bacterial polysaccharides—to recruit T‐cell help, and ZPs are processed by immune cells and loaded onto MHC‐II molecules to be presented to specific T‐cells (referred to as “T_carbs_”).^[^ ^26^, ^27^, ^28^, ^29^, ^30^ ^]^

As serotype 5 is one the most abundant *S. aureus* strains, its CP has been proposed for use as antigen and has been tested in different conjugate vaccine candidates.^[^ ^31^, ^32^, ^33^ ^]^ Conjugates based on CP5 have been tested combined with other antigens in clinical trials from Phase I up to Phase III, involving diverse target populations (hemodialysis patients and subjects undergoing spinal surgery), but unfortunately showing lack of efficacy.^[^ ^33^, ^34^ ^]^ It has recently been described that *S. aureus* can tone down the immune response by inducing the formation of the cytokine IL‐10, leading to inactivation of antibodies^[^ ^35^ ^]^ and dampening the T‐cell response.^[^ ^36^ ^]^ It has also been suggested that IgM and not IgG is more suited to combat *S. aureus*.^[^ ^37^ ^]^ Regardless of the reasons behind the failure of previous clinical trials, the structural heterogeneity of the *S. aureus* polysaccharide, arising from differences in polysaccharide chain length and *N‐* and *O*‐acetylation pattern, challenges the production of well‐defined conjugate vaccines. To focus the immune response toward optimal glycan epitopes to improve vaccine immunogenicity, much attention has recently been directed towards the generation of well‐defined synthetic fragments,^[^ ^38^, ^39^ ^]^ which allows full control over length of the saccharides, *N/O*‐acetylation pattern, as well as conjugation site.^[^ ^40^, ^41^ ^]^ Notably, the synthetic fragments should be long enough to harbor the epitopes that can be recognized by B‐cell receptors (antibodies). The receptors may require multiple repeating units for optimal recognition or recognize a particular frameshift.^[^ ^42^ ^]^ In addition, longer oligosaccharides can take up a secondary structure to present conformational epitopes.^[^ ^43^, ^44^, ^45^ ^]^ The presence of functional groups (such as *O*‐ and *N*‐acetyl groups) can be important to present hydrophobic moieties but also to control conformation.^[^ ^46^ ^]^ Given the huge diversity in bacterial glycan structures, it is difficult‐if not impossible– to predict up‐front what the most successful oligosaccharide will be for antibody engagement.

**Figure 2:**
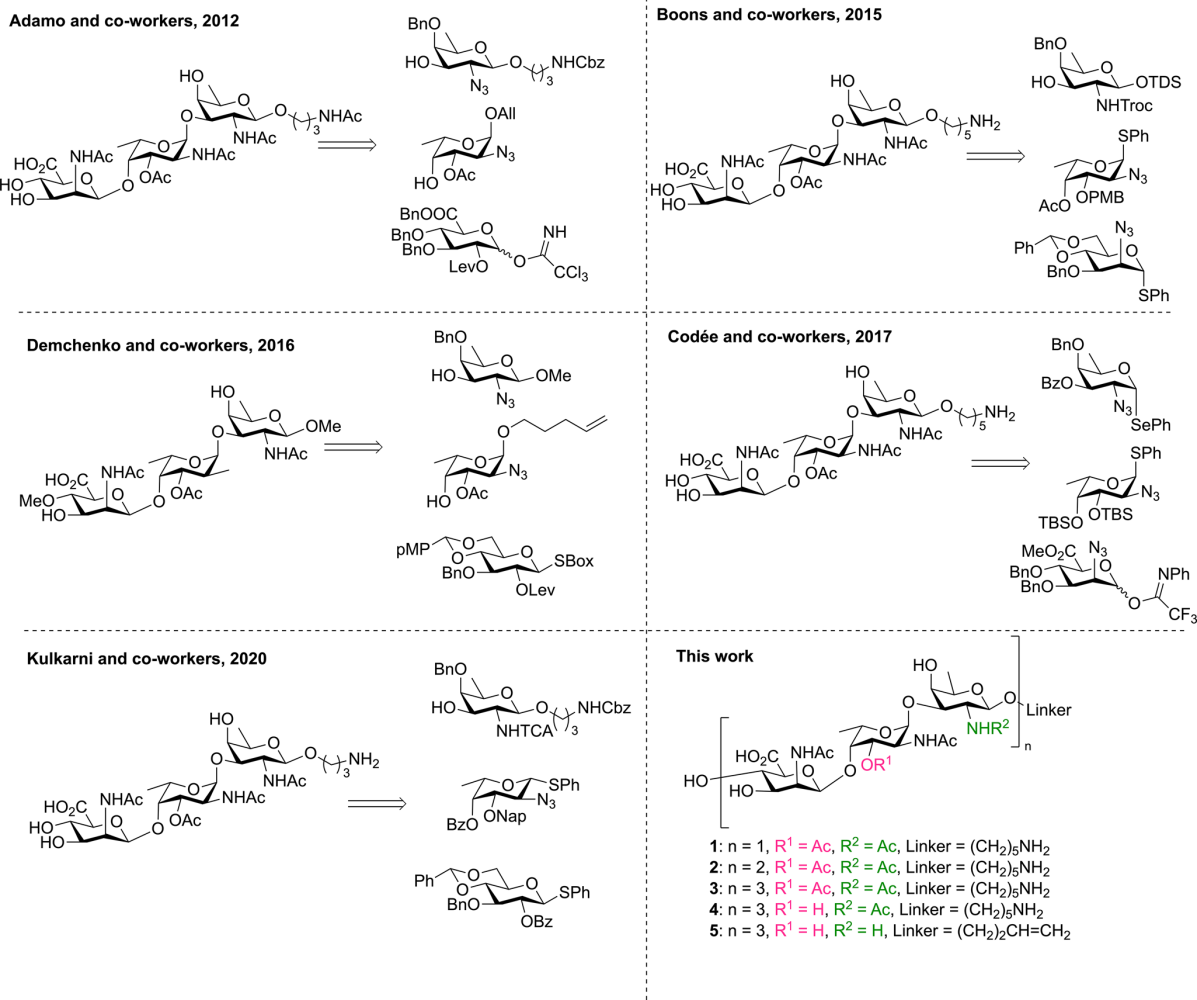
Previously reported synthesis of CP5 trisaccharides and the set of CP5 oligosaccharides reported here.

To date only CP5‐trisaccharides have been assembled, attesting to the challenges associated with the synthesis of these complex glycans. Different CP5 trisaccharides have been synthesized using various strategies to install the 1,2‐*cis* linkages, incorporate the *O*‐acetyl esters, and introduce the carboxylic acid moieties, as summarized in Figure 2. The first synthetic trisaccharide reported^[^ ^47^ ^]^ was constructed from the nonreducing end in a strategy relying on a post‐glycosylation epimerization of C‐2 of a glucuronic acid derivative to generate the β‐ManNAcA residue. The generated trisaccharide was evaluated in a competitive ELISA, but no signification binding to murine anti‐CP5 serum was found. A dot blot assay did show weak binding of the trisaccharide to the anti‐CP5 serum, indicating that larger fragments of CP5 would be needed for potent antibody binding. In 2015, Boons and co‐workers reported a synthetic strategy, relying on late stage oxidation of the C‐6‐OH of a ManNAc residue to avoid lactam formation, which may form when mannuronic acid building blocks are used.^[^ ^48^ ^]^ The trisaccharide was synthesized from the reducing end with late‐stage *O*‐acetylation of the l‐FucN_3_ residue. In 2016 Demchenko and co‐workers reported a synthetic trisaccharide with methyl groups on both capping ends.^[^ ^49^ ^]^ This trisaccharide was constructed from the nonreducing end and the synthetic plan relied on post‐glycosylation epimerization and oxidation on a disaccharide and installation of the *O*‐acetyl group prior to the final [2 + 1] glycosylation. In 2017, it was reported that α‐selectivity in the glycosylation delivering the l‐Fuc‐d‐Fuc disaccharide could be realized through the use of a reactive fucose donor and a weak fucose acceptor nucleophile. For the β‐mannosylation a ManN_3_A donor was used to avoid a post‐glycosylation oxidation step.^[^ ^50^ ^]^ In 2020, Kulkarni and co‐workers published a synthetic route towards the CP5 repeating unit,^[^ ^51^ ^]^ through an [1 + 2] glycosylation of a benzylidene glucose donor and a l‐Fuc‐d‐Fuc disaccharide.

We have recently described the synthesis of *S. aureus* CP8 oligosaccharides, encompassing 1–4 repeating units (RUs), and we found that these oligosaccharides adopt a unique extended structure, in which at least 3 RUs were required to present an adequate epitope for binding.^[^ ^46^ ^]^ The work of Adamo and co‐workers has revealed that CP5 trisaccharides are too short to bind to anti‐CP5 antibodies,^[^ ^47^ ^]^ and therefore, we here set out to generate longer, conjugation ready CP5 fragments, varying in length from a trisaccharide to a nonasaccharide, and varying in *O*‐ and *N*‐acetylation pattern. With the generated fragments, we have been able to establish their three‐dimensional structure, and through SPR‐experiments, we have established that a hexasaccharide presents the minimal epitope. Both *O*‐ and *N*‐acetylation was found to be crucial for antibody binding, leading to the identification of the *O*/*N‐*acetylated CP‐5 hexasaccharide as a promising candidate to further development in a synthetic vaccine modality.

## Results and Discussion

### Synthesis of the CP5‐Fragments

**Scheme 1:**
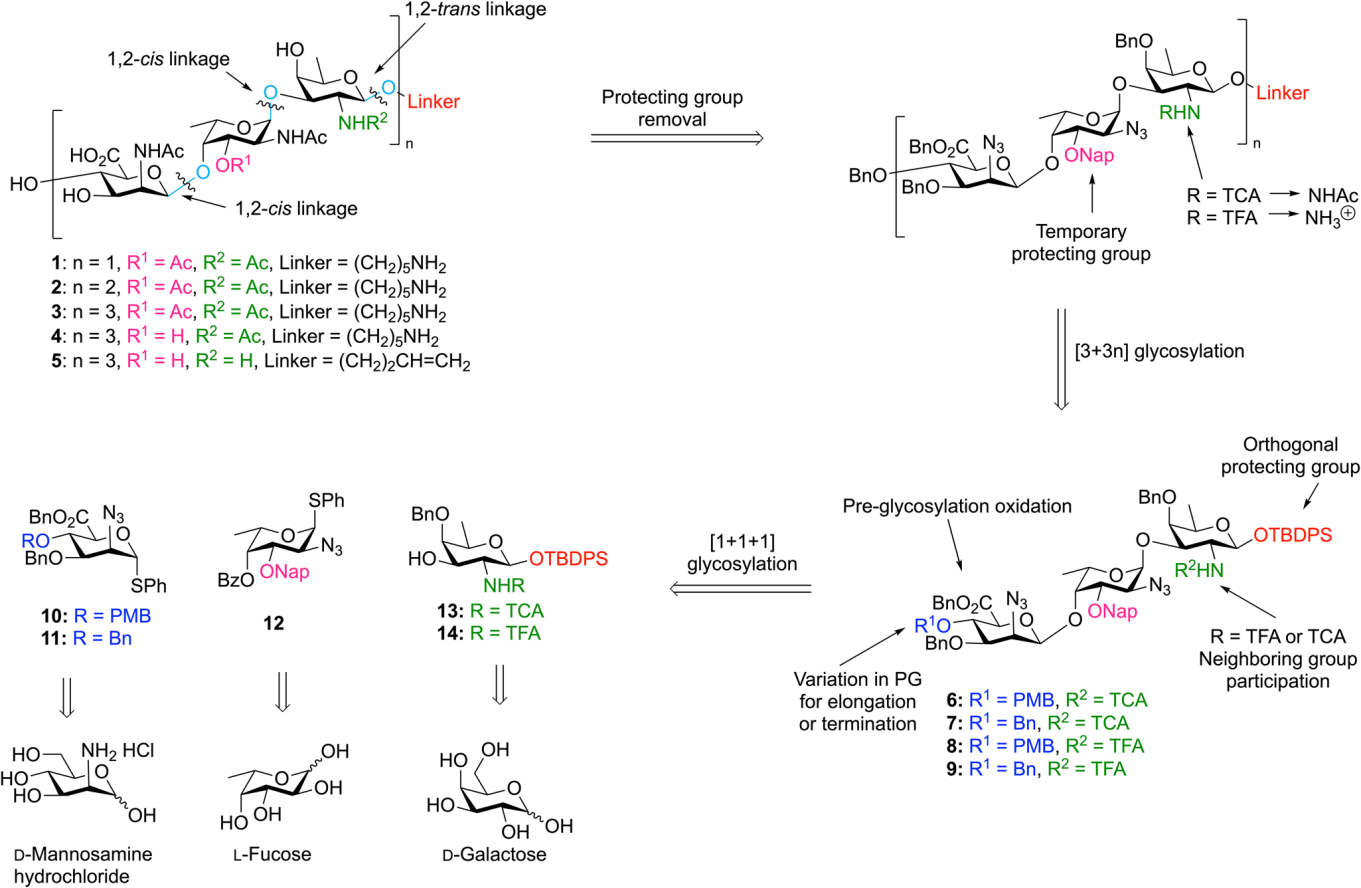
Retrosynthetic strategy for the assembly of the CP5‐oligosaccharides.

Five different CP5 oligosaccharides were targeted in this study: three CP5 fragments ranging in length from one to three RUs (**1**, **2** and **3**, respectively) with all amino groups being acetylated and carrying the l‐FucNAc *O*‐acetyl group, and—as we anticipated that the longest fragments would show best binding^[^ ^46^ ^]^—two additional nonasaccharides, with nonamer **4** representing the de*‐O*‐acetylated counterpart of **3**, and nonamer **5** being a zwitterionic compound. Our retrosynthetic plan to assess the target of the different *S. aureus* CP5 oligosaccharides is shown in Scheme 1. We opted for the use of trisaccharide building blocks with the mannuronic acid at the “nonreducing” end and the d‐fucosamine at the reducing end so that the donor glycosides could be equipped with a participating group to guide the formation of the required β‐fucosamine linkage. This also sets the d‐fucose amino group apart from the other amines in the target molecule, and while azides will be used as a precursor for the acetamides in the ManNAcA and l‐FucNAc residues the amine in the d‐FucN residue is masked with either a trichloroacetyl (TCA) or trifluoroacetyl (TFA) group to enable the formation of either the anionic (containing all *N*‐acetyl groups) or zwitterionic (carrying both *N*‐acetyl and ‐NH_3_^+^ groups) oligosaccharides. The *O*‐acetyl at the C‐3‐OH position of the l‐FucN moiety would be installed at a late‐stage as the electron withdrawing group lowers the α/β selectivity of the l‐FucN‐d‐FucN glycosylation as found by Hagen et al.^[^ ^50^ ^]^ Therefore, a temporary 2‐methylnaphthyl (Nap) ether is introduced as a precursor for the C‐3‐*O*─acetate. This also allowed the generation of the *O*‐acetylated and non‐*O*‐acetylated fragments from the same nonasaccharide precursor. The anomeric position of the d‐FucN moiety in the key trisaccharide synthon was protected with an orthogonal *tert*‐butyldiphenylsilyl (TBDPS) group. For the d‐ManA building block, two different protecting groups were installed at the C‐4‐OH: either a *p*‐methoxybenzyl (PMB) ether, enabling orthogonal deprotection and ensuing chain elongation, or a benzyl ether to generate a chain terminating building block. The use of mannosaminuronic acid building blocks prevents the challenging oxidation reactions on larger oligosaccharides^[^ ^52^, ^53^ ^]^ and enables the stereoselective construction of the β‐ManN_3_A bond.^[^ ^50^, ^54^ ^]^ We equipped all the fragments with a linker for future conjugation purposes. An amine‐functionalized linker was installed on the anionic compounds, while an allyl linker was chosen for the zwitterionic target nonasaccharide. Global deprotection should be facilitated using hydrogenation‐labile permanent protecting groups.

**Scheme 2:**
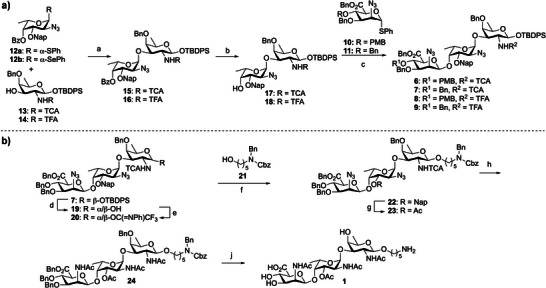
a) Synthesis of trisaccharides 6–9. b) Deprotection of the trisaccharide to form target 1. *Reaction conditions*: a) a NIS, TMSOTf, DCM, –60 °C → –30 °C, 15:91%, *α*/*β* = 95:5; NIS, TBSOTf, DCM –78 °C → –0 °C, 16: 92%, *α*/*β* = 90:10; b NaOMe, MeOH, 17: 92%, 18: 79%; c NIS, TMSOTf, DCM, –60 °C → –10 °C, 6, 86%, *α*/*β* = 30:70, 7:91%, *α*/*β* = 21:79, 8:93%, *α*/*β* = 10:90, 9:86%, *α*/*β* = 10:90. b) d TBAF, AcOH, THF, 0 °C → rt, 81%, e ClC(═NPh)CF_3_, K_2_CO_3_, acetone, 92%, f TBSOTf, DCM/MeCN, –50 °C, 73%, g i DDQ, DCM/H_2_O, ii) Ac_2_O, DMAP, pyridine, 89% over two steps, h zinc, AcOH, Ac_2_O, THF, 50 °C, 18%, j Pd(OH)_2_/C, AcOH, H_2_, *t*‐BuOH/H_2_O, 56%.

All required building blocks **10**–**14** were synthesized from commercially available monosaccharides as detailed in the Supporting Information (SI). We started with the assembly of trisaccharides **6**–**9** to be used for elongation or termination purposes, carrying either a TCA or TFA amine protecting group for formation of the anionic or zwitterionic compounds. A [1 + 2] glycosylation strategy was implemented (Scheme 2a), by first synthesizing the two required l‐FucN‐d‐FucN disaccharides through glycosylation of the l‐FucN_3_ donor **12a/b** and acceptor **13** or **14** in the presence of *N*‐iodosuccinimide (NIS) and trimethylsilyl trifluoromethanesulfonate (TMSOTf, for **12a**) or *tert*‐butyldimethylsilyl triflate (TBSOTf, for **12b**) to deliver the disaccharides **15** and **16** with good stereoselectivity in 91% and 92% yield, respectively. Acceptors **17** and **18** were obtained by saponification of the benzoyl group under Zemplén conditions in 92 and 79%, respectively. Cleavage of the benzoyl esters proceeded extremely sluggishly, requiring 10 days when a catalytic amount (0.2 equiv.) of NaOMe was used. The reaction time could be reduced to two days by using a stoichiometric amount of NaOMe. The [1 + 2] glycosylation between TCA‐acceptor **17** and C‐4‐O‐benzyl donor **11** or C‐4‐O‐PMB‐donor **10** in the presence of NIS and TMSOTf proceeded in 91% (*α*/*β* = 21:79) and 86% (*α*/*β* = 30:70) yield, respectively. The [1 + 2] glycosylations with the TFA‐acceptor **18** proceeded in similar fashion giving the trisaccharides **8** and **9** in 93% (*α*/*β* = 10:90) and 86% (*α*/*β* = 10:90), respectively. All product mixtures were readily separated by silica gel column chromatography to provide the pure anomers.

With the required trisaccharide building blocks in hand, we assembled trisaccharide **1** as depicted in Scheme 2b. First, the TBDPS group in building block **7** was removed with *tetra*‐butylammonium fluoride (TBAF) buffered by acetic acid (AcOH), to yield hemiacetal **19** in 81% yield, which was followed by installation of the *N*‐phenyl trifluoroacetimidate^[^ ^55^ ^]^ to give donor **20**. Glycosylation between donor **20** and the 5‐amino‐*N*‐benzyl‐*N*‐benzyloxycarbonylpentanol^[^ ^56^ ^]^ linker **21** in the presence of TBSOTf gave **22** in 73%, with the β‐product being the only isolated product, due to neighboring group participation of the trichloroacetamide. Next, the l‐FucN‐3‐*O*‐Nap ether was oxidatively cleaved with 2,3‐dichloro‐5,6‐dicyano‐1,4‐benzoquinone (DDQ), after which the 3‐OH was acetylated to give **23**. Subsequently, the azides and the TCA group in **23** were reduced with zinc and AcOH and the resulting free amines acetylated using acetic anhydride (Ac_2_O) in one pot. Unfortunately, after hydrogenation of the thus formed product, we could only obtain the product **1** in sub‐optimal purity (See Supporting Information; purity 60–75% as assessed by NMR). We reasoned that this was likely caused by incomplete reduction of the TCA group. Therefore, different conditions were investigated to optimize the reduction reaction. The use of activated zinc or performing the reduction twice did not lead to more pure material after hydrogenation. Chemoselective reduction of the azides using either Adams’ catalyst (PtO_2_)^[^ ^57^, ^58^ ^]^ or Ru/Al_2_O_3_^[^ ^59^ ^]^ did not lead to any reduced product at all, and employing a chemoselective Staudinger reduction of the azides did not improve the purity either. Finally, the pure product was obtained by implementation of a HPLC‐purification step after the zinc reduction, which delivered material that was > 95% pure, as based on NMR analysis. Unfortunately, this did lead to a significant loss of yield and trisaccharide **24** was obtained in 18% yield. Hydrogenation of this trimer uneventfully provided **1** in 56% after gel filtration.

**Scheme 3:**
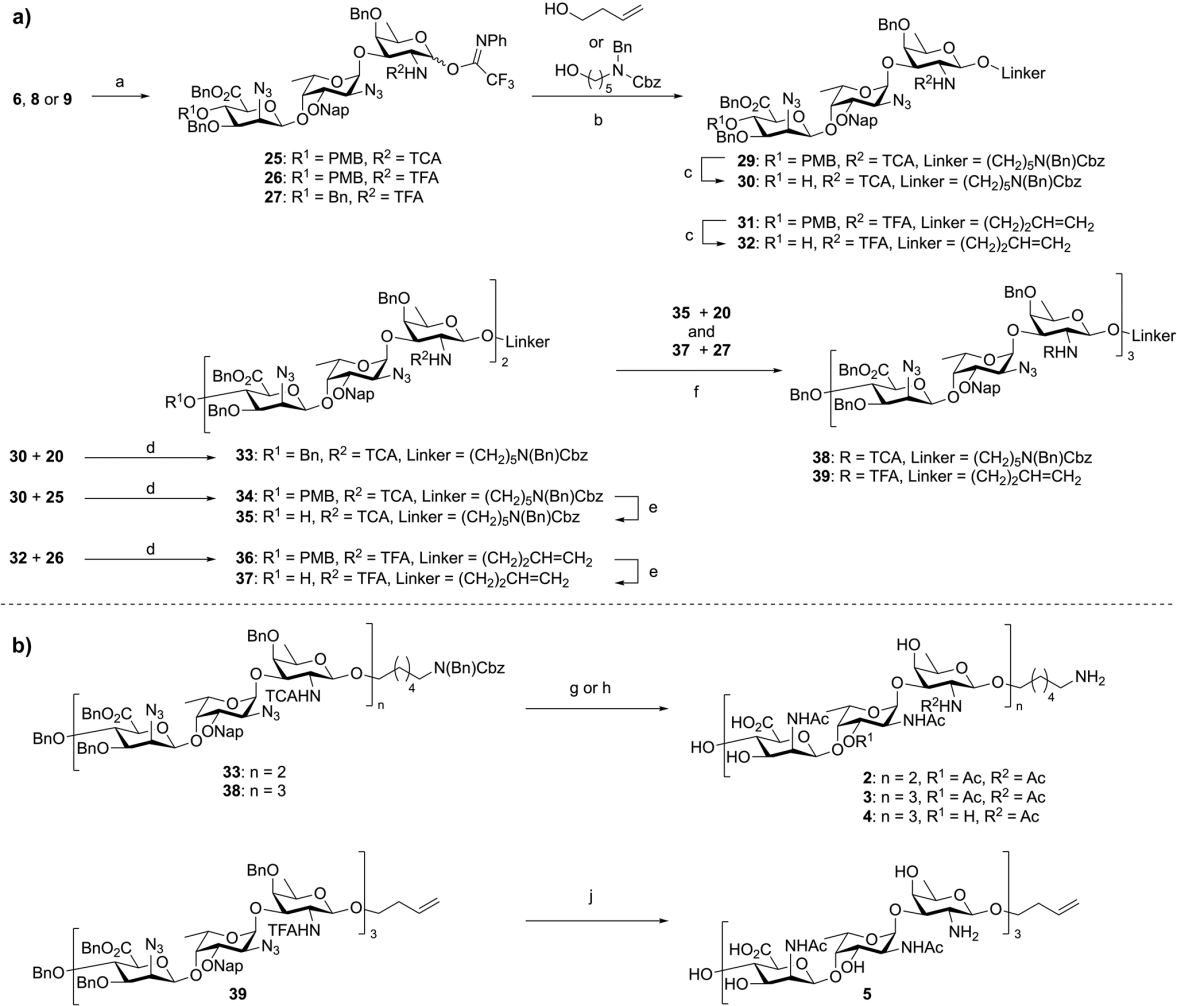
Assembly of oligosaccharides 2–5. *Reaction conditions*:a) a i) TBAF, AcOH, THF, 0 °C → rt °C, ii) ClC(═NPh)CF_3_, K_2_CO_3_, acetone, 96%, 25:87%, 26:79%, 27:83%, b TBSOTf, DCM/MeCN, –50 °C, 29:88%, β only, 31:79%, β only; c HCl in HFIP, TES, DCM, 0 °C, 30:76%, 32:75% d TBSOTf, DCM/MeCN, –78 °C, 33:70%, 34:80%, 36:75%; e HCl in HFIP, TES, DCM, 0 °C, 35:76%, 36:69%; f TBSOTf, DCM/MeCN, –78 °C, 38:79%, 39:65%. b) g for 2:i 33, DDQ, DCM/H_2_O, ii) Ac_2_O, DMAP, pyridine, 82% over two steps, iii) zinc, AcOH, Ac_2_O, THF, 50 °C, 11%, iv) Pd(OH)_2_/C, AcOH, H_2_, *t*‐BuOH/H_2_O, 47%; for 3:i 38, DDQ, DCM/H_2_O, ii) Ac_2_O, DMAP, pyridine, 74% over two steps, iii) zinc, AcOH, Ac_2_O, THF, 50 °C, 16%, iv) Pd(OH)_2_/C, AcOH, H_2_, *t*‐BuOH/H_2_O, 49%; h for 4:i 38, zinc, AcOH, Ac_2_O, THF, 50 °C, ii) Pd(OH)_2_/C, AcOH, H_2_, *t*‐BuOH/H_2_O, 60%; j i) 1 M aq. NaOH, MeOH, THF, (ii) 1,3‐propanedithiol, Et_3_N, H_2_O, pyridine, (iii) THF, H_2_O, NaHCO_3_, Ac_2_O, (iv) Na, liq. NH_3_, *t*BuOH, 3‐buten‐1‐ol, THF, –78 °C, (v) conc, NH_4_OH, 60 °C, 38%.

Next, we set out to assemble the larger saccharides by extending trisaccharides **6** and **8**. This was done by implementing a [3 + 3*n*] glycosylation strategy by turning the trisaccharide synthons **6**, **7**, **8,** and **9** into suitable donors and acceptors. Starting with the synthesis of the anionic CP5 oligosaccharides, hexasaccharide **33** was assembled by desilylation of **6**, which was followed by installation of the *N*‐phenyl trifluoroacetimidate to give donor **25** (Scheme 3a). Glycosylation with linker **21** delivered **29** in good yield and excellent β‐selectivity. The PMB‐ether in **29** could be selectively removed in the presence of the other benzyl ethers using mild acidic conditions (HCl in hexafluoroisopropanol (HFIP))^[^ ^60^ ^]^ to provide acceptor **30**. The fully protected hexasaccharide was generated by glycosylation of acceptor **30** and donor **20** at −78 °C with TBSOTf as promoter. Surprisingly, the use of a 1:1 DCM/acetonitrile (MeCN) solvent system at −30 °C delivered the product as an inseparable *α*/*β* mixture. The use of a higher reaction temperature did not improve the stereoselectivity, while lowering the temperature to −78 °C led to a frozen reaction mixture. By increasing the amount of DCM in the solvent system (to a 2:1 DCM/MeCN ratio), the reaction temperature could be lowered to −78 °C, which led to the formation of the desired β‐linkage with excellent selectivity, providing hexasaccharide **33** in 70% yield. This hexasaccharide was deprotected by oxidative cleavage of the Nap ethers and installation of the *O*‐acetyl ester, which was followed by reduction of the azides and TCA groups (Scheme 3b). Again, purification by silica gel column chromatography and subsequent HPLC purification was found necessary, delivering the acetamide intermediate in 11%. Omission of the HPLC purification did lead to a significantly higher yield, but this material was only ∼75% pure after hydrogenation (as estimated from ^1^H‐NMR). Hydrogenation gave hexasaccharide **2** in 47% yield.

For the synthesis of nonasaccharides **3** and **4** (Scheme 3a), first hexasaccharide **34** was assembled from trisaccharides **30** and **25**. The use of the optimized glycosylation conditions described above in the synthesis of **33**, stereoselectively delivered the hexamer **34** in 80% yield. The PMB group was removed to set the stage for the [3 + 6] glycosylation and the union of acceptor **35** and donor **27** gave nonasaccharide **38** in 70% yield. The established deprotection scheme was again implemented by first transforming the Nap ethers into *O*‐acetyl esters and ensuing reduction of the azides and *N*‐acetylation, followed by the two‐step purification protocol to give the acetamide protected product in 16% yield (Scheme 3b). Finally, hydrogenation afforded pure nonasaccharide **3** in 49% yield. For nonasaccharide **4**, the fully protected intermediate **38** was directly reduced and hydrogenated to afford the non‐*O*‐acetylated target nonasaccharide **4** in 60% overall yield (Scheme 3b).

For the synthesis of the zwitterionic nonasaccharide **5** (Scheme 3a), the intermediate nonasaccharide **39** was assembled using a similar strategy, implementing the TFA‐protected trisaccharide building blocks. Thus, the anomeric TBDPS of trisaccharides **8** and **9** was removed using TBAF buffered by AcOH, and subsequent installation of the *N*‐phenyl trifluoroacetimidate at the anomeric center afforded the trisaccharide donors **26** and **27** in excellent yield over two steps. Next, a butenol linker was installed on donor **26** through a glycosylation reaction in a mixture of DCM/MeCN at –78 °C to stereoselectively provide linker‐equipped trisaccharide **31** in 79% yield as the single β‐anomer. The PMB‐ether in **31** could be selectively removed using HCl/HFIP, and the trisaccharide acceptor **32** was then coupled with donor **26** to deliver the hexasaccharide **36**. Next, the PMB group was removed from hexasaccharide **36** to provide alcohol **37**. The glycosylation to provide the target nonamer was performed with trisaccharide building block **27**, carrying a C‐4″‐*O*‐Bn ether and the [3 + 6] glycosylation proceeded smoothly to deliver nonasaccharide **39** in 65% yield with excellent β selectivity. The deprotection scheme differed from the anionic CP5 oligomers, to enable the installation of the ‐NH_3_^+^ groups. We first saponified the esters before reduction of the azides and then transformed the azides into the acetamides. We found that this could best be achieved using 1,3‐propandedithiol in the presence of Et_3_N, followed by *N*‐acetylation under aqueous conditions. Global removal of all benzyl‐type ethers was then affected using sodium in liquid ammonia at –78 °C. 3‐Butene‐1‐ol was added to the reaction mixture to prevent reduction of the double bond in the linker system. It was noted that the TFA protecting group was not cleaved under the basic Birch reduction conditions and its removal required an additional step using aqueous ammonia at 60 °C. This five‐step reaction sequence delivered the zwitterionic nonasaccharide **5** in 38% yield after size exclusion column chromatography.

### Structural and Conformational Studies

**Figure 3:**
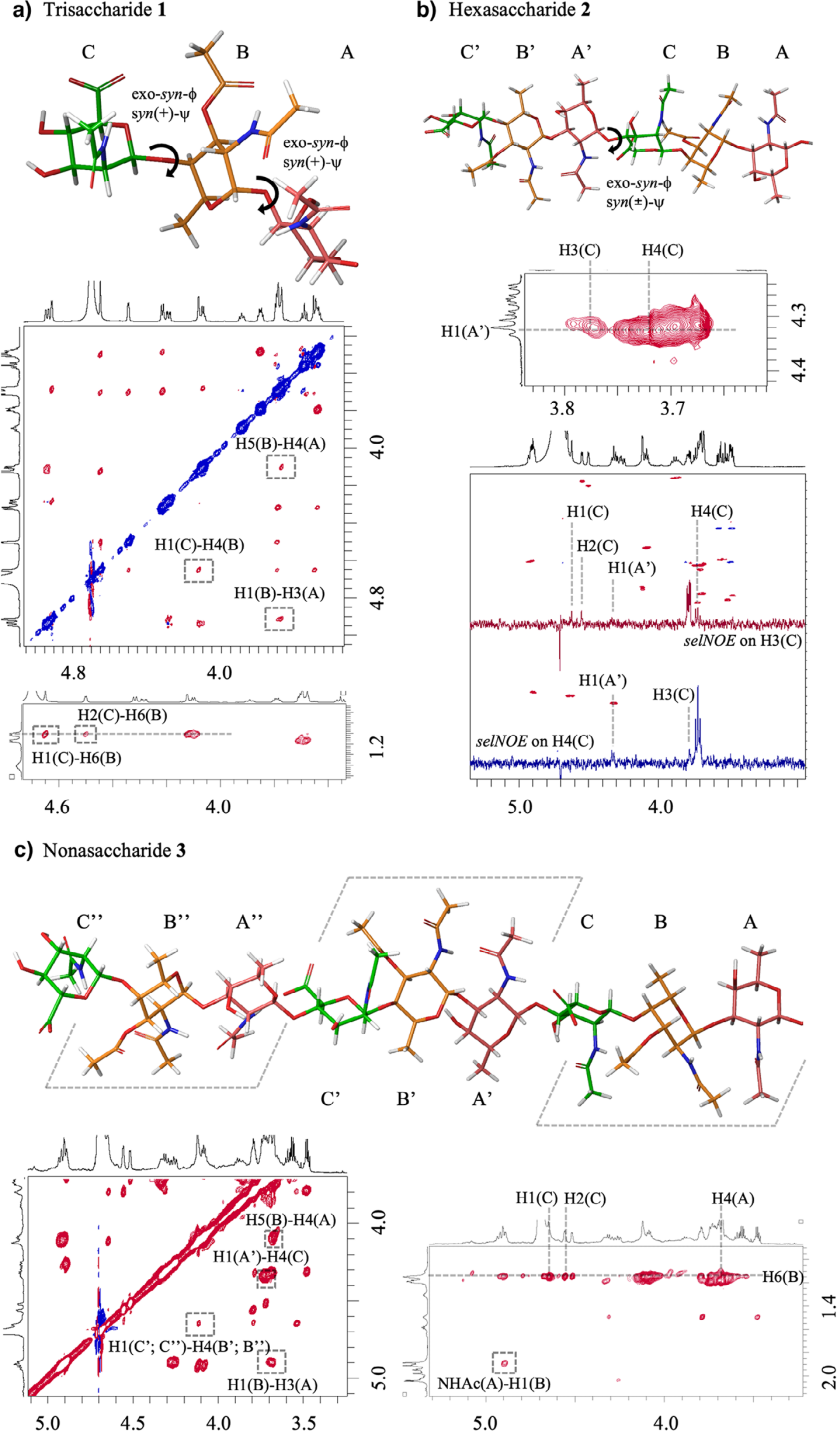
Conformational analysis by NMR and MM calculations of trisaccharide 1, hexasaccharide 2, and nonasaccharide 3, showing the main conformations of the glycosidic linkages and the spatial orientation of the acetyl groups. a) Main conformation and 2D ROESY spectrum of trisaccharide 1. b) Main conformation, section of the two‐dimensional NOESY spectrum taken at the frequency of H1(A’), and 1D selective NOE spectra of hexasaccharide 2. c) Main conformation and 2D sections of the NOE correlations defining the main conformations around the glycosidic linkages of nonasaccharide 3.

With the synthetic oligosaccharides in hand, we sought to establish their conformation and dynamics in solution (Figure 3). The three‐dimensional structure of the CP5 oligomers was determined by using a combination of NMR‐methodologies (*J*‐couplings and NOE‐interactions) and computational protocols (MM). First, trisaccharide **1** was investigated. Analysis of the intra‐residue NOE cross‐peaks and *J*‐couplings determined that the three pyranosides (A:d‐FucNAc, B:l‐FucNAc, and C:d‐ManNAcA) adopt the usual low‐energy chair conformations, which are the ^4^C_1_ for the d‐ManNAcA and d‐FucNAc and the ^1^C_4_ for l‐FucNAc. The conformation around the glycosidic linkages was established using inter residue‐NOEs and MM calculations (Tables S1–S3). For the C–B linkage, strong NOEs between the H1(C)–H4(B) and H1(C)–H6(B) proton pairs, together with the medium‐strong NOE between H2(C)–H6(B) are indicative of a well‐defined and major exo‐*syn*‐ϕ/*syn*(+)‐ψ conformation. The absence, or very minor population, of the alternative exo‐*syn*‐ϕ/*syn*(‐)‐ψ conformation is supported by the extremely weak H5(C)–OAc(B) NOE, which would be expected to be quite strong when ψ adopts negative values. Inspection of the 3D derived structures reveals critical steric contacts between the O‐acetyl group in B and the carboxylate in C in the exo‐*syn*‐ϕ/*syn*(‐)‐ψ conformation. MM calculations estimate an energy difference of ∼2 Kcal·mol^−1^ in favor of the exo‐*syn*‐ϕ/*syn*(+)‐ψ conformation. For the b‐a linkage, the strong H1(B)–H3(A) and H5(B)–H4(A) NOEs, together with the medium/weak H5(B)–H3(A) NOE suggest an equilibrium between the exo‐*syn*‐ϕ/*syn*(+)‐ψ and the exo‐*syn*‐ϕ/*syn*(‐)‐ψ conformations. MM calculations showed the exo‐*syn*‐ϕ/*syn*(‐)‐ψ to be only 1 kcal·mol^−1^ higher in energy. Yet, the absence of the H6(B)–H4(A) and H1(B)–H4(A) NOEs indicates the exo‐*syn*‐ϕ/*syn*(+)‐ψ is most populated. When expanding to hexasaccharide **2**, the same conformations within the trisaccharide units were found, while the A’–C dihedral angle, corresponding to the glycosidic linkage between the two trisaccharidic units, was found to adopt the exo‐*syn*‐ϕ/*syn*(±)‐ψ conformation (Figure 1b). The 2D ^1^H‐^1^H‐NOE spectrum showed correlations between H1 of the a’ residue with both H3 and H4 of the c residue, indicating two alternative conformations around the ψ dihedral angle. To differentiate between these conformations and given the severe ^1^H‐NMR signal overlap in the 2D spectrum, 1D selective NOE experiments were performed. Selective irradiation of the H4(C) NMR signal clearly revealed the inter‐residue NOE correlation with H1(A’). Instead, selective irradiation of the H3(C) signal showed only a very weak NOE correlation with H1(A’) (Figure 1b). These results support the equilibrium between the exo‐*syn*‐ϕ/*syn*(+)‐ψ and the exo‐*syn*‐ϕ/*syn*(‐)‐ψ conformations, with the exo‐*syn*‐ϕ/*syn*(+)‐ψ being the most populated one. The same experimental observations as described for **1** and **2** extend to the nonasaccharide **3** (Figure 1c). Analysis of the 2D NOE experiment revealed that **3** adopts an extended conformation in which the acetyl groups within a repeating unit (RU) point in the same direction, while the relatively flexible glycosidic linkages between the RUs allow the generation of alternating hydrophobic patches along the oligosaccharide chain. In line with the structure established for the CP8, also in CP5, the acetyl groups form hydrophobic flanks in an alternating fashion along the oligosaccharide chain, but while the RUs in CP8 are tilted ∼90° with respect to each other, in CP5 the RUs are flipped ∼180°. This structural difference, along with the higher flexibility of the inter‐RUs glycosidic linkage results in a more disordered structure for CP5 than for CP8 oligosaccharides.

### Antibody Binding

**Figure 4:**
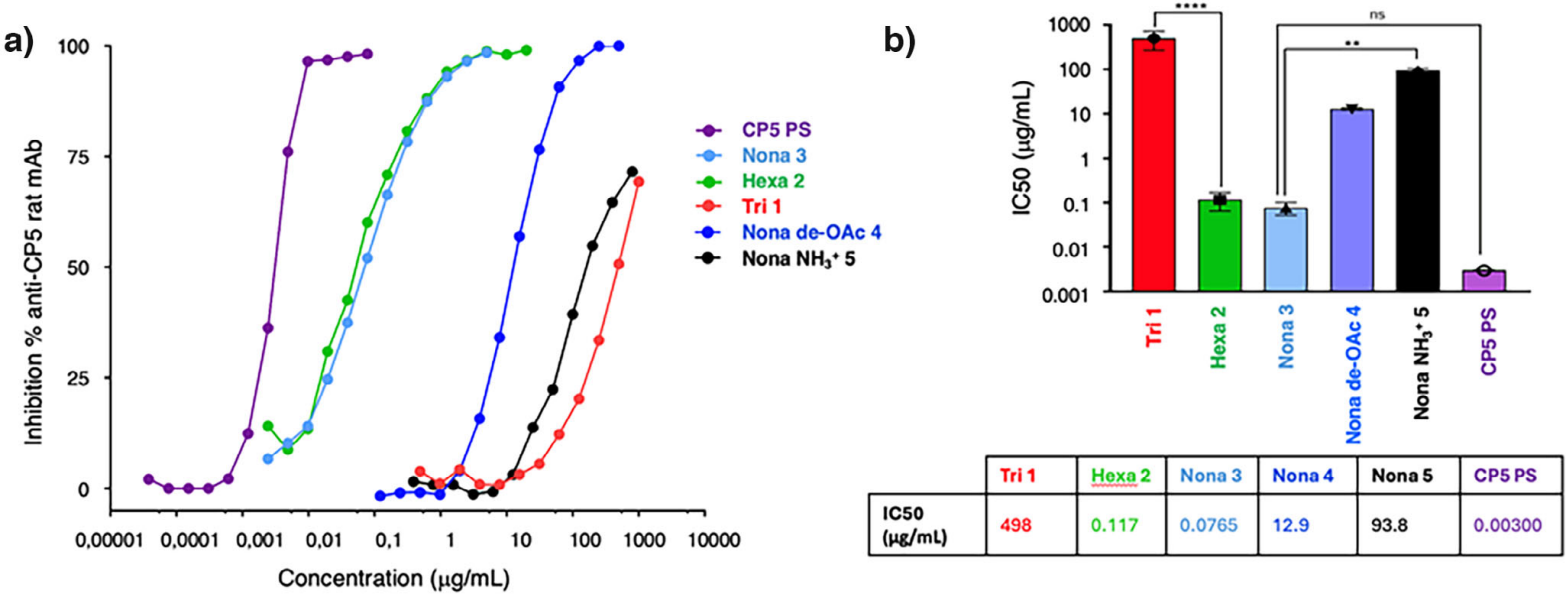
a) Competitive SPR using the synthetic oligosaccharides and rat anti‐CP5 mAb showed that 1 was barely recognized by the antibody, while 2 and 3 bind the antibody well, leading to effective competition. b) IC50 values obtained through competitive SPR. **** p < 0.001, ** p, 0.01, ns = not significant.

Next, we investigated the interaction with anti‐CP5 antibodies. Binding of the synthetic fragments to anti‐CP5 antibodies (anti‐CP5 mAb), generated in rats via hybridoma monoclonal antibody production using the polysaccharide as antigen, was tested by competitive surface plasmon resonance (SPR) using the natural CP5 polysaccharide (CP5‐PS) as the immobilized component. Figure 4 depicts the results of these studies. For the trisaccharide, very poor inhibition of binding was found, indicating that a structure larger than a trisaccharide is required for adequate binding. In contrast, the hexasaccharide showed strong competition of binding to the rat mAb, with a 50% inhibitory concentration (IC_50_) of 0.117 µg mL^−1^ as seen in Figure 2b. Notably, competition of the nonasaccharide **3** was only marginally stronger, indicating that hexasaccharide **2** harbors the minimal epitope for the rat anti‐CP5 mAb. The stronger competition for binding observed with the natural PS (>400 kDa) compared to oligomers can be attributed to the avidity effect, where the multivalent binding of the antibodies is facilitated by multiple epitopes present within the same polysaccharide chain. The importance of the *O‐*acetyl esters and d‐FucNAc acetamides becomes clear from Figure 4a,b. Poor competition is observed for both nonasaccharide **4** and **5** and the IC_50_‐values for these nonasaccharides are significantly higher than that of nonamer **3** (12.9 and 93.8 µg mL^−1^ versus 0.0765 µg mL^−1^). The importance of the *O*‐acetyl ester is in line with what has been found for the activity of the isolated polysaccharide.^[^ ^61^ ^]^ The detrimental effect of the free amines may be explained by unfavorable interactions with key residues in the antibody binding cleft. It may also be that too many positive charges are present in the nonasaccharide or that the free amines are not positioned properly. Notably, it has not yet been revealed whether the positive charges in the natural polysaccharide reside in the d‐FucN or the l‐FucN residues.^[^ ^22^ ^]^ Overall, these studies pinpoint that hexamer **2**, encompassing two repeating units and carrying both *O*‐ and *N*‐acetyl groups, is sufficient to comprise an effective glycan epitope and to be an attractive lead structure for the further development of a synthetic vaccine against *S. aureus* serotype 5.

## Conclusion

We here describe the first synthetic protocol to generate conjugation‐ready CP5 oligosaccharides, larger than a trisaccharide, and featuring different *N‐* and *O*‐acetyl patterns. Our synthetic strategy has employed a pre‐glycosylation oxidation method to introduce the mannosaminuronic acids and [3 + 3*n*] glycosylation sequence to assemble the longer oligomers. Different participating amine protecting groups (TCA and TFA respectively) were used on the d‐fucosamine building block to enable the incorporation either acetamides or introduce free amino groups. Neighboring group participation by the d‐FucN TCA and TFA groups required low reaction temperatures to guarantee stereoselective glycosylation reactions. Conformational analysis showed that the CP5 oligosaccharides adopt distinctive linear structures, with the alternating trisaccharide repeating units being flipped ∼180°. In these structures, the *O*‐ and *N*‐acetyl groups within the trisaccharide repeating units align, forming alternating hydrophobic patches along the oligosaccharide chain. Initial binding studies with an anti‐CP5 antibody have highlighted the importance of the continuous hydrophobic patches, as removal of the *O*‐ and *N*‐acetyl groups leads to poor binding. The importance of the *O*‐acetyl esters is in line with studies performed on the isolated polysaccharide. Our study represents the first report on a well‐defined *S. aureus* CP5 zwitterionic oligosaccharide, and while the generated fragment showed poor binding to the antibodies used, it can be that other substitution patterns, for example, carrying less positive charges, or placing the positively charged amines on the l‐FucN residues rather than the d‐FucN monosaccharides, leads to active fragments. The chemistry disclosed here will be a starting point to develop syntheses to generate these fragments. The binding studies have revealed the requirement for a longer structure to provide good binding. While the trisaccharide bound the antibody poorly, the hexamer does effectively present the minimal epitope for interaction with the antibody. As such, this hexasaccharide represents an attractive structure to further evaluate in synthetic vaccine constructs to fight *S. aureus* serotype 5. The availability of well‐defined synthetic CP5 now opens up avenues to generate glycoconjugate vaccines in a controlled manner to target these important nosocomial pathogens.

## Supporting Information

The supporting information is available free of charge at http://Pubs.Acs.Org. Elaborated synthetic optimizations and building blocks, synthesis, experimental procedures, characterization data, NMR spectra, SPR binding, and structural characterization data (PDF).

## Conflict of Interests

FC, LDB, MRR, and RA are employees of the GSK group of companies.

## Supporting information



Supporting Information

## Data Availability

The data that support the findings of this study are available in the Supporting Information of this article.
