# Supplementary material for: Synthesis, Conformal Analysis, and Antibody Binding of Staphylococcus aureus Capsular Polysaccharide Type 5 Oligosaccharides
Source: Angew Chem Int Ed Engl. 2025 Aug 18;64(39):e202511378. doi: 10.1002/anie.202511378 (PMC12455396; doi:10.1002/anie.202511378)
Supplement: Supplementary file 1 — Supporting Information [file ANIE-64-e202511378-s001.pdf]

Supporting information

Synthesis, conformational analysis and antibody binding of *Staphylococcus aureus* capsular polysaccharide type 5 oligosaccharides

Kitt Emilie Østerlid[1,+], Sizhe Li[1,+], Luca Unione[3,4], Linda Del Bino[2], Charlotte Sorieul[1], Filippo Carboni[2], Francesca Berni[1], Bob van Puffelen[1], Ana Arda[3,4], Herman S. Overkleeft[1], Gijsbert A. van der Marel[1], Maria Rosaria Romano[2], Jesús Jiménez-Barbero[3,4,5,6], Roberto Adamo[2], Jeroen D. C. Codée[1,*]

[1] Leiden Institute of Chemistry, Leiden University, Einsteinweg 55, 2333 CC Leiden, The Netherlands

[2] GSK Siena, Via Fiorentina, 1, 53100 Siena SI, Italy

[3] Center for Cooperative Research in Biosciences (CIC bioGUNE), Basque Research and Technology Alliance (BRTA), 48160 Derio, Bizkaia, Spain.

[4] Ikerbasque, Basque Foundation for Science, Bilbao, Spain.

[5] Department of Organic & Inorganic Chemistry, Faculty of Science and Technology, University of the Basque Country, EHU-UPV, 48940 Leioa, Bizkaia, Spain.

[6] Centro de Investigacion Biomedica En Red de Enfermedades Respiratorias, 28029 Madrid, Spain.

Table of Contents

Overview of the synthesis of the building blocks 2

Deprotection of the trisaccharide 3

Experimental 5

General experimental procedures 5

Synthesis of the building blocks 5

Synthesis of longer fragments 17

SPR experiments 37

Immobilization of CP5-biotin on SA sensor chip 37

Binding analysis of CP5 oligosaccharides 38

Sensorgrams of competitive SPR analyses 39

Structural conformation 45

Overview of the synthesis of the building blocks

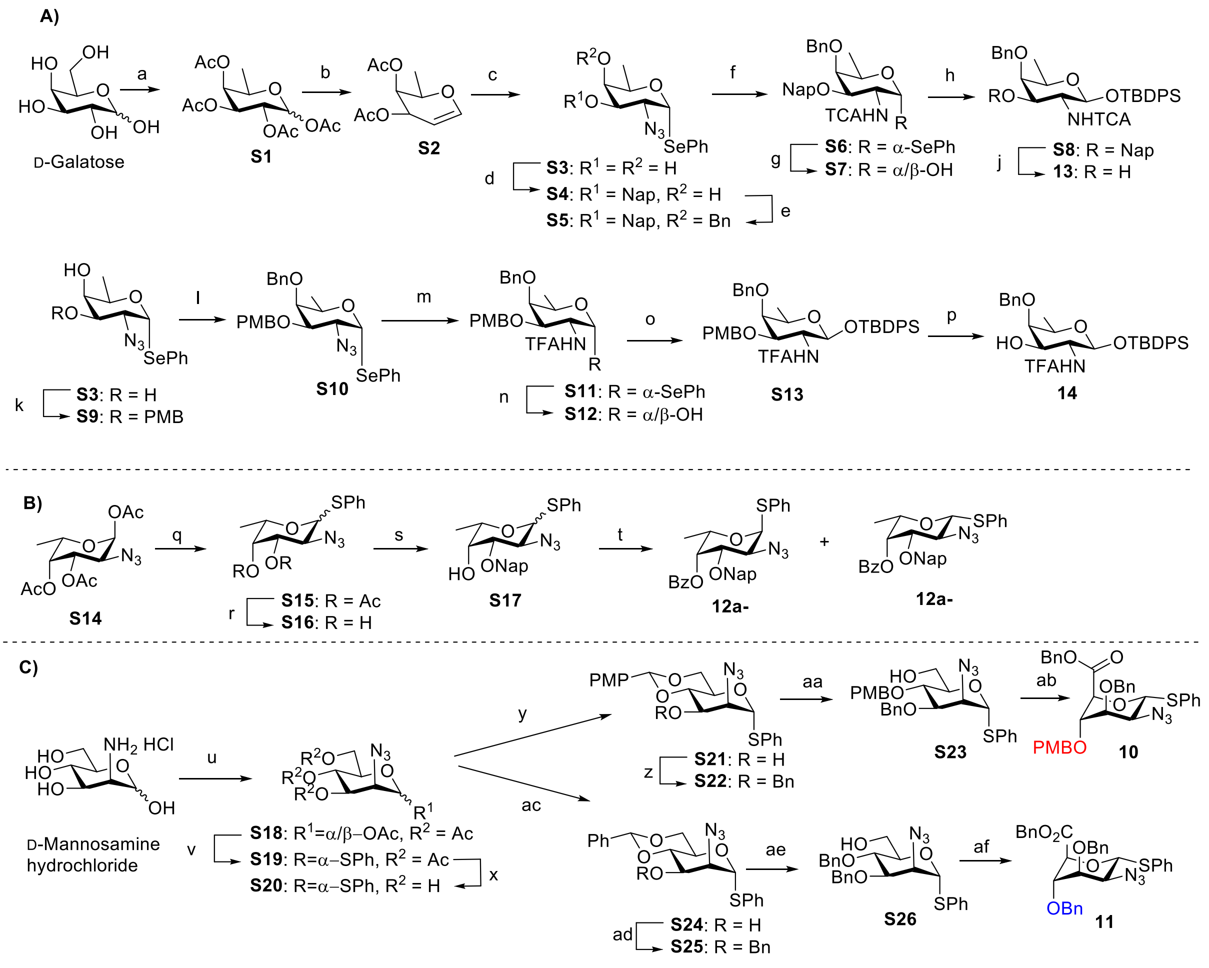

Scheme S1: Synthesis of the building blocks. *Reaction conditions*: A) a) i) conc. H_2SO_4, acetone, ii) PPh_3, I_2, imidazole, toluene/MeCN, 90 °C, iii) VA-044, aq. H_3PO_2, Et_3N, i-PrOH, 60 °C, iv) 80% aq. AcOH, 90 °C, v)Ac_2O, pyridine, 0 °C → rt, 5 steps 54%, b) i) HBr in AcOH 33%, DMC, 0 °C → rt, ii) Zn, NH_4Cl, EtOAc, 60 °C, 2 steps 48%, c) i) $(PhSe)_2$, BAIB, $TMSN_3$, DCM, -20 °C → -30 °C, ii) NaOMe, MeOH, 2 steps 67%, d) Bu_2SnO, toluene, 140 °C then Bu_4NBr, CsF, NapBr, 120 °C, 93%, e) BnBr, NaH, DMF, 0 °C → rt, 88%, f) zinc, AcOH, THF, ii) TCACl, THF, 0 °C, 93% over two steps, g) NIS, acetone/H_2O, 0 °C, 91%, h) TBDPSCl, imidazole, DMAP, DCM, 0 °C → rt, 92%, j) DDQ, DCM/H_2O, 92%, k) Bu_2SnO, toluene, 140 °C then Bu_4NBr, CsF, PMBCl, 120 °C, 92%, l) BnBr, NaH, DMF, 0 °C → rt, 88%, m) PPh_3, H_2O, THF, 40 °C ii) $(TFA)_2O$ pyridine, 0 °C 94% over two steps, n) NIS, acetone/H_2O, 0 °C, crude used, o) TBDPSCl, imidazole, DMAP, DCM, 0 °C → rt, 82% over two steps, p) DDQ, DCM/H_2O, 96%; B) q) PhSH, $BF_3 \cdot Et_2O$, DCM, 84%, α/β=2:1, r) NaOMe, MeOH, 99%, s) Bu_2SnO, toluene, 140 °C then Bu_4NBr, CsF, NapBr, 120 °C, 99%, t) BzCl, DMAP, DCM/pyridine, 0 °C → rt, 95%, α/β = 56:44; C) u) i) Tf_2O, NaN_3, $CuSO_4 \cdot 5\ H_2O$, pyridine, 0 °C ii) Ac_2O, 0 °C → rt, 2 steps 98%, v) SHPh, $BF_3 \cdot Et_2O$, DCM, 0 °C → rt, 88%, x) NaOMe, MeOH, 90%, y) p-MeO-PhCH$(OMe)_2$, CSA, MeCN, 300 mbar, 50 °C, 88%, z) BnBr, NaH, DMF, 0 °C → rt, 88%, aa) $BH_3 \cdot THF$, TMSOTf, DCM, 0 °C, 89%, ab) i) TEMPO, BAIB, AcOH, DCM/*t*-BuOH/H_2O, 0 °C → rt °C, ii) BnBr, K_2CO_3, DMF, 91% over two steps, ac) PhCH$(OMe)_2$, CSA, MeCN, 50 °C, 300 mbar, 79%, ad) BnBr, NaH, DMF, 0 °C → rt, 75%, n) $BH_3 \cdot THF$, TMSOTf, DCM, 0 °C, 96%, af) i) TEMPO, BAIB, AcOH, DCM/*t*-BuOH/H_2O, 0 °C → rt, ii) BnBr, K_2CO_3, DMF, 79% over two steps.

Deprotection of the trisaccharide

The azides and the TCA group were reduced with zinc in AcOH and acetylated using acetic anhydride (Ac_2O) in one pot. Table S1, Entry 1). Unfortunately, after hydrogenation of the so-formed product, we could only obtain the product in sub-optimal purity (See Table 1, entry 1; purity 60-75% as assessed by NMR). We reasoned that this was likely caused by incomplete reduction of the TCA group. Therefore, different conditions were investigated to optimize the reduction reaction (see Table S1). The use of activated zinc (Entry 2) or performing the reduction twice (Entry 3) did not lead to more pure material after hydrogenation. Chemoselective reduction of the azides using either Adams' catalyst (PtO_2)[48,49] (Entry 4) or Ru/Al_2O_3[50], (Entry 5 and 6) did not lead to any reduced product at all, and employing a chemoselective Staudinger reduction of the azides did not improve the purity either (Entry 7). Finally, the pure product was obtained by implementation of a HPLC-purification step after the zinc reduction, which delivered material that was >95% pure, as based on NMR analysis, after hydrogenation. Unfortunately, this did lead to a significant loss of yield and trisaccharide **34** was obtained in 18% yield. Hydrogenation of this trimer uneventfully provided **1** in 56% after gel-filtration.

Table S1: Deprotection optimization towards trisaccharide **1**.

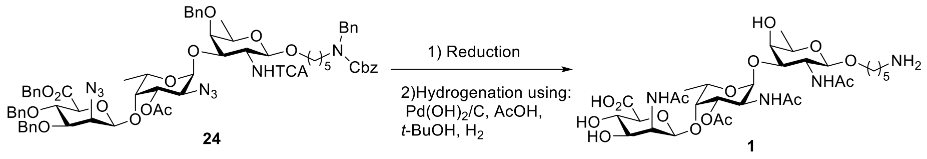

Entry	Conditions	Time	Yield[(a)]	Purity after hydrogenation [(b)]
1	1) Zinc, AcOH, Ac_2O, THF, silica gel purification<br>2) $Pd(OH)_2/C$, AcOH, H_2, *t*-BuOH/H_2O	1) 22 h<br>2) 3 days	1) 78%<br>2) 43%	~75%
2	1) Activated zinc, AcOH, Ac_2O, THF, silica gel purification<br>2) $Pd(OH)_2/C$, AcOH, H_2, *t*-BuOH/H_2O	1) 22 h<br>2) 3 days	1) 49%<br>2) 79%	~60%
3	1) Zinc, AcOH, Ac_2O, THF, silica gel column; repeated once<br>2) $Pd(OH)_2/C$, AcOH, H_2, *t*-BuOH/H_2O	1) 22 h + 22 h<br>2) 3 days	1) 72%<br>2) 81%	~75%
4	1) PtO_2, EtOAc, H_2 then Ac_2O, MeOH<br>2) --	1) 3 days then 1 day<br>2) --	1) --	No reaction
5	1) Ru/Al_2O3, AcOH, EtOAc/ toluene, H_2 then Ac_2O, MeOH<br>2) --	1) 1 day then 1 day<br>2) --	1) --	No reaction

6	1) Ru/Al_2O_3, AcOH, MeOH, H_2 then Ac_2O, MeOH<br>2) --	1) 1 day then 1 day<br>2) --	1) --	No reaction
7	1) PPh_3, H_2O, THF then H_2O,<br>$NaHCO_3$, Ac_2O<br>2) $Pd(OH)_2$/C, AcOH, H_2, *t*-BuOH/H_2O	1) 1 day then 2 h<br>2) 3 days	1) <83% [(c)]<br>2) --	Complex mixture [(d)]
8	1) Zinc, AcOH, Ac_2O, THF, silica gel purification, followed by HPLC<br>2) $Pd(OH)_2$/C, AcOH, H_2, *t*-BuOH/H_2O	1) 22 h<br>2) 3 days	1) 18%<br>2) 56%	>95%

[(a)] Isolated yields [(b)] Purity was assessed using ^{1}H-NMR. [(c)] Estimated yield. [(d)] NMR showed the presence of the product in a complex mixture.

Experimental

General experimental procedures

All reagents were of commercial grade and used as received unless otherwise noted. All moisture sensitive reactions were performed under an argon or nitrogen (N_2) atmosphere. Dried solvents (DCM, DMF, THF, toluene, Et_2O) were stored over flame-dried 3 or 4Å molecular sieves. Reactions were monitored by thin layer chromatography (TLC) analysis conducted with Merck aluminum sheets with 0.20 mm of silica gel 60. The plates were detected by UV (254 nm) and were applicable by spraying with 20% sulfuric acid in EtOH or with a solution of $(NH_4)_6Mo_7O_{24}\cdot 4H_2O$ (25 g/L) and $(NH_4)_4Ce(SO_4)_4\cdot 2H_2O$ (10 g/L) in 10% sulfuric acid (aq.) followed by charring at ~150 °C. Flash column chromatography was performed with silica gel (40-63μm). Size-exclusion chromatography was carried out using SephadexTM (LH-20, GE Healthcare Life Sciences) by isocratic elution with DCM/MeOH (1:1, v:v). HW40 columns were performed using 0.15 M NH4OAc and 10% ACN in milliQ as eluent on a 16x600 mm column. For reversed-phase HPLC purifications an Agilent Technologies 1200 Series instrument equipped with a semi-preparative column (Gemini C18, 250 x 10 mm, 5 micrometer particle size, Phenomenex) was used. A 90/10 to 10/90 A/B gradient was applied, using eluent system A (1% HOAc in H2O) and B (ACN).

High-resolution mass spectra were recorded on a Thermo Finigan LTQ Orbitrap mass spectrometer equipped with an electrospray ion source in positive mode (source voltage 3.5 kV, sheath gas flow 10, capillary temperature 275 °C) with resolution R=60.000 at m/z=400 (mass range 150-4000). ^{1}H and ^{13}C spectra were recorded on a Bruker AV-400 (400 and 101 MHz respectively), Bruker AV-500 (500 and 126 MHz respectively), Bruker AV-600 (600 and 151 MHz respectively), Bruker AV-850 (800 and 214 MHz respectively) or a Bruker AV-1200 (1200 and 302 MHz respectively). Chemical shifts (δ) are given in ppm relative to the residual signal of the deuterated solvent (^{1}H-NMR: 7.26 ppm for $CDCl_3$, 3.31 ppm for MeOD, 1.94 for $CNCD_3$ or 4.79 for D_2O. ^{13}C-NMR: 77.16 ppm for $CDCl_3$, 49.00 ppm for MeOD, 1.32 for $CNCD_3$). Coupling constants (*J*) are given in Hz. All ^{13}C spectra are proton decoupled. NMR peak assignments were made using COSY and HSQC experiments, where applicable, HMBC and GATED experiments were used to further elucidate the structure. The anomeric product ratios were analyzed through integration of proton NMR signals.

Synthesis of the building blocks

1,2,3,4-tetra-*O*-acetyl-D-fucopyranose (S1)

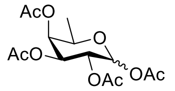

Acetone (1200 mL) was cooled to 0 °C and conc. H_2SO_4 (40 mL) was slowly added dropwise. D-Galactose (50 g, 277.5 mmol) was added portion wise and the reaction was allowed to warm to rt and stirred for 7 h until TLC (Pent/EtOAc, 1:1) showed full conversion. The now yellow solution was cooled to 0 °C and neutralized with $NaHCO_3$ (sat. aq.) until pH~8-9. The acetone was evaporated and the aqueous phase was extracted with EtOAc (x3). The combined organic phases were washed with brine (x1), dried over Na_2SO_4, filtered and concentrated *in vacuo*. The crude product (62.04 g, 238.3 mmol) was dissolved in toluene/MeCN (2:1, 700 mL). First PPh_3 (118.66 g, 524.4 mmol, 2.2 equiv.) and imidazole (71.39 g, 1050 mmol, 4.4 equiv.) were added and next I_2 (90.74 g, 357.5 mmol, 1.5 equiv.) was added portion wise. The reaction was heated to 90 °C and stirred for 24 h until TLC (Pent/EtOAc 1:1) showed full conversion. After cooling to rt, the solvents were evaporated and the residue was dissolved in EtOAc, washed with $Na_2S_2O_3$ (aq., sat., x2), H_2O (x2) and brine (x1), dried over Na_2SO_4, filtered and concentrated *in vacuo*. To the crude product in *i*-PrOH (700 mL) was added Et_3N (199 mL, 1450 mmol, 6 equiv.) and aq. H_3PO_2 (50%, 84 mL, 953.2 mmol, 4 equiv.) and stirred under N_2 for 30 min. VA-044 (23.11 g, 71.49 mmol, 0.3 equiv.) was added at rt and the reaction was heated to 80 °C and stirred under N_2 for 1 h until TLC (Pent/EtOAc 4:1) showed full conversion. The solvent was evaporated and residue was dissolved in EtOAc, washed with NH_4Cl (sat. aq.;

x1) and brine (x1), dried over Na_2SO_4, filtered and concentrated *in vacuo*. The crude residue was dissolved in 80% aq. AcOH (600 mL) and stirred at 90 °C for 18 h until TLC (Pent/EtOAc 4:1) showed full conversion. The solvents were evaporated and the residue was co-evaporated with toluene (x4). The residue was dissolved in pyridine (700 mL) and cooled to 0 °C. Ac_2O (400 mL) was added and the reaction was slowly allowed to warm to rt and stirred for 18 h until TLC (Pent/EtOAc 4:1) showed full conversion. The solvents were evaporated and the residue wash dissolved in EtOAc, washed with 1 M HCl (x2), sat. aq. $NaHCO_3$ (sat. aq.; x3) and brine (x1), dried over Na_2SO_4, filtered and concentrated *in vacuo*. Column chromatography (Pentane/EtOAc 85:15 → 70:30) gave the wanted product as in 54% yield (49.42 g, 149 mmol) in a α/β ratio = 0.8:1. **^{1}H NMR (400 MHz, $CDCl_3$)** δ 6.34 (s, 1H, α-H-1), 5.68 (d, *J* = 8.3 Hz, 1H, *β-H-1*), 5.33 (t, *J* = 1.4 Hz, 2H, α-H-4, α-H-3), 5.33 – 5.32 (m, 1H, α-H-2), 5.32 – 5.29 (m, 1H, β-H-4), 5.27 (dd, *J* = 3.5, 1.1 Hz, 1H, β-H-2), 5.07 (dd, *J* = 10.4, 3.4 Hz, 1H, β-H-3), 4.30 – 4.23 (q, *J* = 6,5 Hz, 1H, α-H-5), 3.95 (*J* = 6.5 Hz, 1H, β-H-5), 2.19 (s, 3H, COC*H₃*), 2.18 (s, 3H, COC*H₃*), 2.14 (s, 3H, COC*H₃*), 2.11 (s, 3H, COC*H₃*), 2.04 (s, 3H, COC*H₃*), 2.01 (s, 3H, COC*H₃*), 2.00 (s, 3H, COC*H₃*), 1.99 (s, 3H, COC*H₃*), 1.22 (d, *J* = 6.4 Hz, 3H, *β-H-6*), 1.15 (d, *J* = 6.5 Hz, 3H, *α-H-6*). **^{13}C NMR (101 MHz, $CDCl_3$)** δ 170.70 (C=O), 170.68 (C=O), 170.36 (C=O), 170.20 (C=O), 170.11 (C=O), 169.63 (C=O), 169.33 (C=O), 169.32 (C=O), 92.31 (β-C-1), 90.09 (α-C-1), 71.39 (β-C-3), 70.71 (α-C-3/ β-C-4/ α-C-4), 70.39 (β-C-5), 70.05 (β-C-2), 68.03 (α-C-3/ β-C-4/ α-C-4), 67.96 (α-C-2), 67.42 (α-C-5), 66.59 (α-C-3/ β-C-4/ α-C-4), 21.08 (CO*C*H₃), 21.00 (CO*C*H₃), 20.83 (CO*C*H₃), 20.80 (CO*C*H₃), 20.77 (CO*C*H₃), 20.73 (CO*C*H₃), 16.07 (C-6), 16.06 (C-6). **ESI HRMS**: $[M+Na]^+$ calculated for $C_{14}H_{20}O_9Na$: 355.10050; found 355.09974

3,4-di-*O*-acetyl-D-fucal (S2)

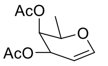

S1 (17.83 g, 53.7 mmol) was dissolved in DCM (215 mL, 0.25 M) and cooled to 0 °C. HBr in AcOH (33%, 14.6 mL, 80.55 mmol, 1.5 equiv.) was added using a dropping funnel and stirred at 0 °C under N_2 for 2 h until TLC (Pent/EtOAc 4:1) showed full conversion. The solution was poured over ice and stirred until the ice was molten. The aqueous phase was extracted with DCM (x3) and the combined organic phases were washed with H_2O (x1) and brine (x1), dried over Na_2SO_4, filtered and concentrated *in vacuo*. The residue was co-evaporated with toluene (x3) and used immediately without any further purification. The crude product (17.51 g, 49.74 mmol) was dissolved in EtOAc (166 mL, 0.3 M) and Zn (22.77 g, 348.2 mmol, 7 equiv.) and NH_4Cl (18.62 g, 348.2 mmol, 7 equiv.) were added portion wise. The reaction was stirred at 60 °C under N_2 for 1 h until TLC (Pent/EtOAc 4:1) showed full conversion, cooled to rt, filtered and concentrated *in vacuo*. Column chromatography (Pentane/EtOAc + 1% Et_3N 9:1 → 7:3) gave **S2** in 44% yield (5.11 g, 23.9 mmol) (49% brsm). **^{1}H NMR (400 MHz, $CDCl_3$)** δ 6.46 (dd, *J* = 6.4, 2.0 Hz, 1H, H-1), 5.60 – 5.52 (m, 1H, H-3), 5.28 (dq, *J* = 4.7, 1.8 Hz, 1H, H-4), 4.63 (dt, *J* = 6.3, 1.9 Hz, 1H, H-2), 4.21 (q, *J* = 6.5 Hz, 1H, H-5), 2.15 (s, 3H, COC*H*₃), 2.01 (s, 3H, COC*H*₃), 1.27 (d, *J* = 6.6 Hz, 3H, H-6). **^{13}C NMR (101 MHz, $CDCl_3$)** δ 170.86 (C=O), 170.56 (C=O), 146.25 (C-1), 98.39 (C-2), 71.65 (C-3), 66.37 (C-4), 65.17 (C-5), 21.00 (CO*C*H₃), 20.85 (CO*C*H₃), 16.66 (C-6). **ESI HRMS**: $[M+Na]^+$ calculated for $C_{10}H_{14}O_5Na$: 237.07389; found 237.07422

Phenyl 2-azido-2-deoxy-1-seleno-α-D-fucopyranoside (S3)

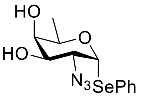

S2 (6.562 g, 30.66 mmol) and $(PhSe)_2$ (9.57 g, 30.66 mmol, 1 equiv.) was dissolved in DCM (155 mL, 0.2 M) and degassed under argon at rt for 30 min. The reaction was cooled to -30 °C, added BAIB (9.87 g, 30.66 mmol, 1 equiv.) and $TMSN_3$ (8.1 mL, 61.31 mmol, 2 equiv.) and stirred at -20 °C and stirred overnight until TLC (Tol/EtOAc 9:1) showed full conversion. Cyclohexene (12 mL) was added, and the reaction was stirred at rt for 30 min before concentration. The lipophilic by products were removed by Column chromatography (Pentane/EtOAc 10:0 → 7:3) were all the carbohydrate positive fraction was collected. The crude residue (14.46 g, 35 mmol, impure) was dissolved in MeOH (120 mL, 0.3 M) and added NaOMe (1.6 mL, 7 mmol, 0.2 equiv.). The reaction was stirred at rt for 2 h until TLC (Pent/EtOAc 1:1) showed full conversion and then neutralized with Amberlite IR-120 H^+ resins, filtered and concentrated *in vacuo*. The crude product was recrystallized in hot toluene to give **S3** in 67% yield (6.78 g, 20.7 mmol). **^{1}H NMR (400 MHz, MeOD)** δ 7.60 – 7.55 (m, 2H, Ar-*H*), 7.34 – 7.22 (m, 3H, Ar-*H*), 5.91 (dd,

J = 5.2, 0.6 Hz, 1H, H-1), 4.29 (q, *J* = 6.5 Hz, 1H, H-5), 4.01 (dd, *J* = 9.9, 5.3 Hz, 1H, H-2), 3.76 – 3.68 (m, 2H, H-4, H-3), 1.15 (d, *J* = 6.6 Hz, 3H, H-6). **^{13}C NMR (101 MHz, MeOD)** δ 135.91 (Ar-*C*), 130.04 (Ar-*C*), 128.72 (Ar-C_q), 86.89 (C-1), 72.91 (C-3), 72.68 (C-4), 70.61 (C-5), 62.91 (C-2), 16.42 (C-6). **ESI HRMS**: $[M+Na]^+$ calculated for $C_{12}H_{15}N_3O_3SeNa$: 352.01763; found 352.01709

Phenyl 2-azido-2-deoxy-3-*O*-(2-naphthylmethyl)-1-seleno-α-D-fucopyranoside (S4)

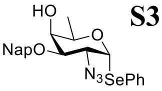

S3 (3.60 g, 10.94 mmol) was co-evaporated with toluene (x3) and dissolved in dry toluene (55 ml, 0.2 M). Bu_2SnO (2.778 g, 11.16 mmol, 1.02 equiv.) was added. The flask was equipped with a Dean-Stark and the reaction was heated to 140 °C for 3 h. The now clear solution was cooled to 60 °C before adding Bu_4NBr (3.704 g, 11.49 mmol, 1.05 equiv.), CsF (1.965 g, 11.16 mmol, 1.02 equiv.) and NapBr (2.540 g, 11.49 mmol, 1.05 equiv.). The reaction was heated to 120 °C for 1 h until TLC (Pent/EtOAc 3:2) showed full conversion. The reaction was allowed to cool to rt before a 10% KF solution was added and the reaction was stirred for 30 min. The aqueous phase was extracted with EtOAc (x3) and the combined organic phases were washed with brine (x1), dried over Na_2SO_4, filtered and concentrated. Column chromatography (Pentane/EtOAc 9:1 → 7:3) gave **S4** in 93% yield (4.768 g, 10.18 mmol). **^{1}H NMR (400 MHz, $CDCl_3$)** δ 7.92 – 7.82 (m, 4H, Ar-*H*), 7.63 – 7.45 (m, 5H, Ar-*H*), 7.33 – 7.27 (m, 3H, Ar-*H*), 5.91 (d, *J* = 5.4 Hz, 1H, H-1), 4.88 (dd, *J* = 13.6, 11.5 Hz, 2H, Ar-CH_2), 4.30 (qt, *J* = 6.6, 1.5 Hz, 1H, H-5), 4.21 (dd, *J* = 10.2, 5.3 Hz, 1H, H-2), 3.91 (dt, *J* = 3.2, 1.6 Hz, 1H, H-4), 3.76 (dd, *J* = 10.2, 3.1 Hz, 1H, H-3), 2.39 (t, *J* = 1.6 Hz, 1H, OH), 1.26 (d, *J* = 6.5 Hz, 3H, H-6). **^{13}C NMR (101 MHz, $CDCl_3$)** δ 133.35 (Ar-*C*), 133.33 (Ar-C_q), 129.24 (Ar-C_q), 128.79 (Ar-*C*), 128.63 (Ar-C_q) 128.13 (Ar-*C*), 127.94 (Ar-*C*), 127.91 (Ar-*C*), 127.17 (Ar-*C*), 126.53 (Ar-*C*), 126.45 (Ar-*C*), 125.81 (Ar-*C*), 85.30 (C-1), 79.35 (C-3), 72.42 (Ar-CH_2), 68.71 (C-5, C-4), 60.40 (C-2), 16.83 (C-6). **ESI HRMS**: $[M+H]^+$ calculated for $C_{23}H_{23}N_3O_3SeH$: 470.09829; found 470.09776

Phenyl 2-azido-4-*O*-benzyl-2-deoxy-3-*O*-(2-naphthylmethyl)-1-seleno-α-D-fucopyranoside (S5)

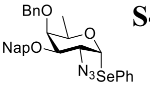

S4 (3.228 g, 6.89 mmol) was dissolved in DMF (67 mL, 0.1 M) and cooled to 0 °C. BnBr (1.06 mL, 8.96 mmol, 1.3 equiv.) and NaH (60% suspension in mineral oil, 358 mg, 8.96 mmol, 1.3 equiv.) was added and the solution was slowly allowed to warm to rt and stirred under N_2 for 18 h until TLC (Pent/EtOAc 9:1) showed full conversion. The reaction was quenched with H_2O and extracted with Et_2O (x3). The combined organic phases were washed with brine (x1), dried over Na_2SO_4, filtered and concentrated *in vacuo*. Column chromatography (Pentane/EtOAc 95:5 → 85:15) gave **S5** in 88% yield (3.627 g. 6.49 mmol). **^{1}H NMR (400 MHz, $CDCl_3$)** δ 7.92 – 7.81 (m, 4H, Ar-*H*), 7.60 – 7.53 (m, 3H, Ar-*H*), 7.53 – 7.47 (m, 2H, Ar-*H*), 7.37 – 7.19 (m, 8H, Ar-*H*), 5.95 (d, *J* = 5.3 Hz, 1H, H-1), 5.01 – 4.87 (m, 3H, Ar-CH_2, Ar-CH_2), 4.65 (d, *J* = 11.4 Hz, 1H, Ar-CH_2), 4.40 (dd, *J* = 10.3, 5.3 Hz, 1H, H-2), 4.23 (q, *J* = 6.3 Hz, 1H, H-5), 3.79 (dd, *J* = 10.3, 2.7 Hz, 1H, H-3), 3.74 (dd, *J* = 2.8, 1.2 Hz, 1H, H-4), 1.14 (d, *J* = 6.5 Hz, 3H, H-6). **^{13}C NMR (101 MHz, $CDCl_3$)** δ 138.24 (Ar-C_q), 135.09 (Ar-C_q), 134.50 (Ar-*C*), 133.42 (Ar-C_q), 133.23 (Ar-C_q), 128.54 (Ar-*C*), 128.45 (Ar-*C*), 128.29 (Ar-*C*), 128.13 (Ar-*C*), 127.92 (Ar-*C*), 127.89 (Ar-*C*), 127.80 (Ar-*C*), 126.76 (Ar-*C*), 126.41 (Ar-*C*), 126.26 (Ar-*C*), 125.81 (Ar-*C*), 85.68 (C-1), 80.79 (C-3), 75.98 (C-4), 75.16 (Ar-*C*H_2-3), 72.77 (Ar-*C*H_2), 69.55 (C-5), 61.17 (C-2), 16.69 (C-6). **ESI HRMS**: $[M+Na]^+$ calculated for $C_{30}H_{29}N_3O_3SeNa$: 582.12718; found 582.12685

Phenyl 4-*O*-benzyl-2-deoxy-3-*O*-(2-naphthylmethyl)-2-*N*-trichloroacetamide-1-seleno-α-D-fucopyranoside (S6)

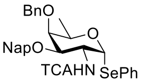

S5 (3.471 g, 6.214mmol) was dissolved in distilled THF (62 mL, 0.1 M). zinc powder (4.47 g, 68.36 mmol, 11 equiv.) was gently added to the solution followed by AcOH (3.2 mL, 55.92 mmol, 9 equiv.). The reaction was stirred under nitrogen at rt overnight until TLC (pentane/EtOAc, 95:5) showed full conversion. The reaction mixture was filtered over a path of celite and concentrated *in vacuo*. The crude was co-evaporated with toluene (x3) before being dissolved in distilled THF (41 mL, 0.15 M). Flamed dried 3Å molecular sieves were added to the solution and the mixture was stirred under nitrogen for 30 min. Then. The solution was cooled to 0 °C and trichloroacetyl chloride (1.4 mL, 12.43

mmol, 2 equiv.) was added. The reaction mixture was stirred for 30 min at 0 °C under nitrogen until TLC (pentane/EtOAc, 8:2) showed full conversion. The reaction mixture was diluted in DCM, washed with brine (x1), dried over Na_2SO_4, filtered and concentrated *in vacuo*. Column chromatography (pentane/EtOAc, 95:15 → 85:15) yielded **S6** in 76% yield (3.212 g, 4.598 mmol). **^{1}H NMR (400 MHz, $CDCl_3$)** δ 7.90 – 7.78 (m, 4H, *Ar-H*), 7.55 – 7.44 (m, 5H, Ar-H), 7.41 – 7.20 (m, 8H, Ar-H), 6.86 (d, *J* = 7.5 Hz, 1H, *H*N(CO)CCl_3), 6.04 (d, *J* = 4.7 Hz, 1H, H-1), 5.01 (d, *J* = 11.5 Hz, 1H, ArC*H*$_2$), 4.90 (d, *J* = 12.2, 0.8 Hz, 1H, ArC*H*$_2$), 4.84 – 4.74 (m, 1H, H-2), 4.74 – 4.67 (m, 2H, ArC*H*$_2$), 4.27 – 4.17 (m, 1H, H-5), 3.86 (dd, *J* = 2.6, 1.3 Hz, 1H, H-4), 3.65 (dd, *J* = 11.0, 2.5 Hz, 1H, H-3), 1.28 (d, *J* = 6.5 Hz, 3H, *H*-6). **^{13}C NMR (101 MHz, $CDCl_3$)** δ 161.68 (*C*=O), 138.13 (Ar-C_q), 134.66 (Ar-C_q), 134.18 (Ar-C_q), 133.39 (Ar-C_q), 133.25 (Ar-C_q), 129.41 (Ar-*C*), 128.93 (Ar-C_q), 128.84 (Ar-*C*), 128.50 (Ar-*C*), 128.39 (Ar-*C*), 128.09 (Ar-*C*), 128.06 (Ar-*C*), 127.96 (Ar-*C*), 127.92 (Ar-*C*), 126.77 (Ar-*C*), 126.60 (Ar-*C*), 126.41 (Ar-*C*), 125.57 (Ar-*C*), 89.27 (C-1), 78.81 (C-3), 74.93 (Ar-*C*H$_2$), 74.60 (C-4), 71.70 (Ar-*C*H$_2$), 70.62 (C-5), 52.07 (C-2), 16.97 (C-6). **ESI HRMS**: $[M+Na]^+$ calculated for $C_{32}H_{30}Cl_3NO_4SeNa$: 700.03033; found 700.0293

4-*O*-benzyl-2-deoxy-3-*O*-(2-naphthylmethyl)-2-*N*-trichloroacetamide-α-D-fucopyranose (S7)

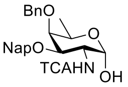

S6 (3.212 g, 4.738 mmol) was dissolved in acetone/water (9:1; 95 mL, 0.05 M). The reaction was cooled to 0 °C, followed by addition of NIS (2.132 g, 9.475 mmol, 2 equiv.). The reaction mixture was stirred 15 min at 0 °C until TLC (pentane/EtOAc, 8:2) showed full conversion. The reaction was quenched with $Na_2S_2O_3$ (sat., aq.) and the solvent was evaporated *in vacuo*. The crude residue was diluted in EtOAc and the organic phase was washed with $Na_2S_2O_3$ (sat. aq.; x1), $NaHCO_3$ (sat. aq.; x1) and brine (x1), dried over Na_2SO_4, filtered and concentrated *in vacuo*. Column chromatography (pentane/EtOAc, 80:20 → 60:40) yielded hemiacetal **S7** in 95% (2.431 g, 4.512 mmol). **^{1}H NMR (400 MHz, $CDCl_3$)** δ 7.86 – 7.77 (m, 4H, Ar-*H*), 7.56 – 7.43 (m, 3H, Ar- *H*), 7.41 – 7.35 (m, 2H, Ar- *H*), 7.34 – 7.26 (m, 3H, Ar- *H*), 6.81 (d, *J* = 8.9 Hz, 1H, *H*N(CO)CCl_3), 5.38 (t, *J* = 3.6 Hz, 1H, H-1), 5.03 (d, *J* = 11.6 Hz, 1H, ArC*H*$_2$), 4.87 (d, *J* = 12.2 Hz, 1H, ArC*H*$_2$), 4.81 – 4.61 (m, 3H, H-2, ArC*H*$_2$), 4.14 – 4.06 (m, 1H, H-5), 3.85 (dd, *J* = 10.9, 2.5 Hz, 1H, *H*-3), 3.81 – 3.73 (m, 1H, H-4), 2.94 (dd, *J* = 3.5, 1.5 Hz, 1H, O*H*), 1.20 (d, *J* = 6.5 Hz, 3H, H-6). **^{13}C NMR (101 MHz, $CDCl_3$)** δ 161.97 (*C*=O), 138.29 (Ar-C_q), 135.13 (Ar-C_q), 133.35 (Ar-C_q), 133.18 (Ar-C_q), 128.63 (Ar-*C*), 128.56 (Ar-*C*), 128.51 (Ar-*C*), 128.46 (Ar-*C*), 128.07 (Ar-*C*), 127.91 (Ar-*C*), 127.87 (Ar-*C*), 126.63 (Ar-*C*), 126.43 (Ar-*C*), 126.23 (Ar-*C*), 125.70 (Ar-*C*), 91.78 (C-1), 77.03 (C-3), 75.01 (C-4), 74.74 (Ar*C*H$_2$), 71.84 (Ar*C*H$_2$), 67.09 (C-5), 51.25 (C-2), 17.11 (C-6). **ESI HRMS**: $[M+Na]^+$ calculated for $C_{26}H_{26}Cl_3NO_5Na$: 560.07743; found 560.07688

***Tert*-butyldiphenylsilyl 4-*O*-benzyl-2-deoxy-3-*O*-(2-naphthylmethyl)-2-*N*-trichloroacetamide-β-D-fucopyranoside (S8)**

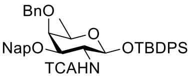

S7 (2.417 g, 4.486 mmol) was co-evaporated with toluene (x3) before being dissolved in dry DCM (22 mL, 0.2 M). The reaction was cooled to 0 °C followed by addition of DMAP (110 mg, 0.897 mmol, 0.2 equiv.) and imidazole (764 mg, 11.216 mmol, 2.5 equiv.) and TBDPS-Cl (1.4 mL, 5.383 mmol, 1.5 equiv.). The reaction mixture was stirred under nitrogen at rt overnight until TLC analysis (pentane/EtOAc, 7:3) showed full conversion. The reaction mixture was diluted in EtOAc, washed with 1 M HCl (aq.; x1) and brine (x1), dried over Na_2SO_4, filtered and concentrated *in vacuo*. Column chromatography (pentane/EtOAc, 98:5 → 90:10) furnished **S8** in 86% yield (2.99 g, 3.846 mmol). **^{1}H NMR (400 MHz, $CDCl_3$)** δ 7.80 (m, 3H, Ar-*H*), 7.76 – 7.69 (m, 3H, Ar-*H*), 7.67 – 7.59 (m, 2H, Ar-*H*), 7.52 – 7.27 (m, 14H, Ar-*H*), 6.80 (d, *J* = 7.1 Hz, 1H, *H*N(CO)CCl_3), 4.96 (d, *J* = 11.8 Hz, 1H, Ar-C*H*$_2$), 4.88 (d, *J* = 7.3 Hz, 1H, H-1), 4.78 (d, *J* = 11.7 Hz, 1H, Ar-C*H*$_2$), 4.68 (d, *J* = 11.7 Hz, 1H, Ar-C*H*$_2$), 4.67 (d, *J* = 11.8 Hz, 1H, ArC*H*$_2$), 4.12 – 3.97 (m, 2H, H-2, H-3), 3.59 (dd, *J* = 2.6, 1.1 Hz, 1H, H-4), 3.30 – 3.20 (m, 1H, H-5), 1.09 – 1.01 (m, 12H, H-6, (C*H*$_3$)$_3$). **^{13}C NMR (101 MHz, $CDCl_3$)** δ 138.55 (Ar-C_q), 136.24 (Ar-*C*), 135.98 (Ar-*C*), 135.19 (Ar-C_q), 133.55 (Ar-C_q), 133.42 (Ar-C_q), 133.32 (Ar-C_q), 133.16 (Ar-C_q), 129.76 (Ar-*C*), 129.65 (Ar-*C*), 128.49 (Ar-*C*), 128.39 (Ar-*C*), 128.18 (Ar-*C*), 128.04 (Ar-*C*), 127.85 (Ar-*C*), 127.75 (Ar-*C*), 127.60 (Ar-*C*), 127.34 (Ar-*C*), 126.87 (Ar-*C*), 126.38 (Ar-*C*), 126.21 (Ar-*C*), 125.97 (Ar-*C*), 94.84 (C-1), 77.95 (C-3),

75.14 (C-4), 74.74 (Ar-*C*H_2), 72.28 (Ar-*C*H_2), 70.60 (C-5), 57.66 (C-2), 27.11 ((*C*H_3)$_3$), 16.82 (*C*-6). **ESI HRMS**: $[M+Na]^+$ calculated for $C_{42}H_{44}Cl_3NO_5SiNa$: 798.19520; found 798.19465

***Tert*-butyldiphenylsilyl 4-*O*-benzyl-2-deoxy-2-*N*-trichloroacetamide-β-D-fucopyranoside (13)**

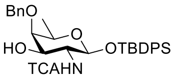

S8 (2.99 g, 3.846 mmol) was dissolved in DCM/H_2O (20:1, 38 mL, 0.1 M) followed by addition of DDQ (1.309 g, 5.769 mmol, 1.5 equiv.). The reaction mixture was stirred under nitrogen for 2 h at rt until TLC (pentane/EtOAc, 9:1) showed full conversion. The reaction mixture was subsequently quenched with $Na_2S_2O_3$ (sat. aq.) and diluted in EtOAc. The organic phase was washed with $NaHCO_3$ (sat. aq.; x5) and brine (x1), dried over Na_2SO_4, filtered and concentrated *in vacuo*. Column chromatography (pentane/EtOAc, 85:15 → 80:20) furnished acceptor **13** in 85% yield (2.088g, 3.278 mmol). **^{1}H NMR (400 MHz, CDCl$_3$)** δ 7.76 – 7.68 (m, 2H, Ar-*H*), 7.68 – 7.61 (m, 2H, Ar-*H*), 7.46 – 7.28 (m, 11H, Ar-*H*), 6.61 (d, *J* = 7.9 Hz, 1H, *H*N(CO)CCl$_3$), 4.80 (d, *J* = 11.6 Hz, 1H, ArC*H*$_2$), 4.71 (d, *J* = 11.6 Hz, 1H, ArC*H*$_2$), 4.56 (d, *J* = 7.9 Hz, 1H, H-1), 4.00 (dt, *J* = 10.8, 7.9 Hz, 1H, H-2), 3.66 (td, *J* = 10.7, 9.8, 3.5 Hz, 1H, H-3), 3.50 (dd, *J* = 3.6, 1.1 Hz, 1H, H-4), 3.34 – 3.24 (m, 1H, H-5), 2.56 (d, *J* = 9.8 Hz, 1H, O*H*), 1.14 (d, J = 6.4 Hz, 3H, H-6), 1.07 (s, 9H, (C*H*$_3$)$_3$). **^{13}C NMR (101 MHz, CDCl$_3$)** δ 136.24 (Ar-*C*), 135.96 (Ar-*C*), 133.19 (Ar-*C$_q$*), 129.95 (Ar-*C*), 129.85 (Ar-*C*), 128.74 (Ar-*C*), 128.18 (Ar-*C*), 127.74 (Ar-*C*), 127.46 (Ar-*C*), 95.46 (C-1), 79.26 (C-4), 76.17 (Ar*C*H$_2$), 72.70 (C-3), 70.99 (C-5), 58.70 (C-2), 27.05 ((*C*H$_3$)$_3$), 19.34 (*C*(CH$_3$)$_3$), 16.74 (C-6). **ESI HRMS**: $[M+Na]^+$ calculated for $C_{31}H_{36}Cl_3NO_5SiNa$: 658.13260; found 658.13205

Phenyl 2-azido-2-deoxy-3-*O*-(*p*-methoxybenzyl)-1-seleno-α-D-fucopyranoside (S9)

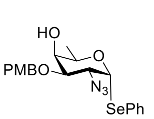

Compound **S3** (19 g, 46 mmol) was dissolved in methanol (200 mL) and 5 mL of CH_3ONa (4.73 M in methanol solution) was added. The mixture was stirred until TLC analysis (petroleum ether/acetone, 4/1) indicated complete conversion of the starting material. The reaction mixture was neutralized by addition of Amberlite IR-120 (H^+ from) to pH = 6. The resin was filtered, and the filtrate was concentrated *in vacuo* to afford the diol intermediate as a solid. The solid was co-evaporated with toluene for 3 times and dissolved in 150 mL of toluene. To the reaction mixture was added Bu_2SnO (12.6 g, 50.6 mmol), and the mixture was heated to 140 °C for 3 h, after which the reaction was cooled to room temperature, followed by the addition of PMBCl (7.9 g, 6.9 mL, 50.6 mmol), TBAB (16.3 g, 50.6 mmol) and CsF (7.7 g, 50.6 mmol). The reaction mixture was heated to 120 °C and stirred for 2 h until TLC (petroleum ether/EtOAc, 4/1) indicated complete conversion of the starting material. The solvent was evaporated, and the given black residue was re-dissolved in 200 mL EA. The organic phase was washed with 10% KF solution (100 mL), water (200 mL) and brine (200 mL), dried over Na_2SO_4, filtered, and concentrated *in vacuo*. The crude product was purified by column chromatography (silica gel, petroleum ether/EtOAc = 5/1) to afford the **S9** (22 g, 92% yield). **^{1}H NMR (400 MHz, CDCl$_3$)** δ 7.60 – 7.54 (m, 2H, Ar-*H*), 7.36 – 7.26 (m, 5H, Ar-*H*), 6.95 – 6.88 (m, 2H, Ar-*H*), 5.88 (d, *J* = 5.3 Hz, 1H, H-1), 4.73 – 4.59 (m, 2H, Ar-C*H*$_2$), 4.29 (qd, *J* = 6.5, 1.4 Hz, 1H, H-5), 4.15 (dd, *J* = 10.2, 5.3 Hz, 1H, H-2), 3.86 (dd, *J* = 3.1, 1.3 Hz, 1H, H-4), 3.82 (s, 3H, PMB-OC*H*$_3$), 3.69 (dd, *J* = 10.2, 3.1 Hz, 1H, H-3), 2.37 (s, 1H, OH), 1.26 (d, *J* = 6.6 Hz, 3H, H-6). **^{13}C NMR (101 MHz, CDCl$_3$)** δ 159.83 (Ar-*C$_q$*), 134.52 (Ar-*C*), 129.93 (Ar-*C*), 129.24 (Ar-*C*), 128.79 (Ar-*C$_q$*), 128.68 (Ar-*C$_q$*), 127.91 (Ar-*C*), 114.24 (Ar-*C*), 85.38 (C-1), 78.99 (C-3), 71.95 (Ar-*C*H$_2$), 68.70 (C-4/ C-5), 68.61 (C-4/ C-5), 60.23 (C-2), 55.44 (PMB-OCH$_3$), 16.20 (C-6). **ESI HRMS**: $[M+Na]^+$ calculated for $C_{20}H_{23}N_3O_4SeNa$: 472.07515; found 472.07463

Phenyl 2-azido-4-*O*-benzyl-2-deoxy-3-*O*-(*p*-methoxybenzyl)-1-seleno-α-D-fucopyranoside (S10)

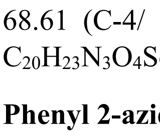

To a solution of **S9** (17.5 g, 40.3 mmol) in 300 mL DMF was added BnBr (10.3 g, 7.2 mL, 60.4 mmol) and NaH (2.4 g, 60.4 mmol, 60% in mineral oil) at 0 °C. The reaction was stirred at 0 °C for 2 h until TLC (petroleum ether/EtOAc, 9/1) indicated complete conversion of the starting material. The reaction was quenched by addition of methanol, and the mixture was diluted in 300 mL Et_2O and washed with water (500 mL) and brine (500 mL). The organic layer was dried over Na_2SO_4, filtered, and concentrated *in vacuo*. The crude product was purified by column chromatography (silica gel, petroleum

ether/EtOAc, 13/1) to afford the **S10** (19 g, 88% yield) as a colorless oil.). **^{1}H NMR (400 MHz, $CDCl_3$)** δ 7.58 – 7.54 (m, 2H, Ar-*H*), 7.40 – 7.26 (m, 9H, Ar-*H*), 6.96 – 6.89 (m, 2H, Ar-*H*), 5.92 (d, *J* = 5.3 Hz, 1H, H-1), 4.95 (d, *J* = 11.4 Hz, 1H, Ar-C*H*2), 4.70 (d, *J* = 1.6 Hz, 2H, Ar-C*H*2), 4.60 (d, *J* = 11.4 Hz, 1H, Ar-C*H*2), 4.33 (dd, *J* = 10.2, 5.3 Hz, 1H, H-2), 4.21 (dd, *J* = 7.0, 5.9 Hz, 1H, H-5), 3.83 (s, 3H, PMB-OC*H*3), 3.76 – 3.66 (m, 2H, H-3, H-4), 1.12 (d, *J* = 6.5 Hz, 3H, H-6). **^{13}C NMR (101 MHz, $CDCl_3$)** δ 160.04 (Ar-C_q), 138.08 (Ar-C_q), 135.15 (Ar-*C*), 134.47 (Ar-*C*), 129.73 (Ar-*C*), 129.16 (Ar-*C*), 128.45 (Ar-*C*), 128.34 (Ar-*C*), 127.91 (Ar-*C*), 127.78 (Ar-*C*), 114.12 (Ar-*C*), 85.76 (C-1), 80.45 (C-3), 75.93 (C-4), 75.11 (Ar-*C*H2), 72.37 (Ar-*C*H2), 69.53 (C-5), 60.97 (C-2), 55.44 (PMB-O*C*H3), 16.68 (C-6). **ESI HRMS**: $[M+Na]^+$ calculated for $C_{27}H_{29}N_3O_4SeNa$: 562.12210; found 562.12173. The NMR data of above compounds **S3**, **S9**, **S10** are consistent with reported.[6]

Phenyl 4-*O*-benzyl-2-deoxy-3-*O*-(*p*-methoxybenzyl)-2-*N*-trifluoroacetamide-1-seleno-α-D-fucopyranoside (S11)

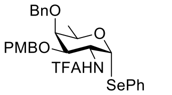

Compound **S10** (9.0 g, 17.2 mmol) was dissolved in THF (170 mL), PPh_3 (9.0 g, 34.4 mmol) was added under nitrogen. The mixture was stirred at 40 °C for 2 h. After the starting material was completely consumed, water (4.6 mL) was added. The mixture was refluxed at 65 °C overnight. When TLC (petroleum ether/acetone, 4/1) showed the reaction was completed, the mixture was concentrated to give the crude product, which was co-evaporated with toluene for three times, and used for the next step without further purification. The crude white solid was dissolved in pyridine (170 mL), followed by the addition of trifluoroacetic anhydride (5.4 g, 3.6 mL, 25.7 mmol) at 0 °C. The reaction mixture was stirred from 0 °C to room temperature for 3 h until TLC (petroleum ether/EtOAc, 9/1) indicated complete conversion of the starting material. The reaction was quenched by addition of methanol and the solvents were evaporated *in vacuo*. The given brown residues were purified by silica gel column chromatography (DCM/ACE = 100/1 to 50/1, due to the solubility of these compounds, petroleum ether/EtOAc system did not elute efficiently) to furnish product **S11** (9.6 g, 94% yield). **^{1}H NMR (500 MHz, $CDCl_3$)** δ 7.51 – 7.46 (m, 2H, ArH), 7.41 – 7.32 (m, 4H, ArH), 7.32 – 7.21 (m, 6H, ArH), 6.97 – 6.90 (m, 2H, ArH), 6.13 (d, *J* = 7.5 Hz, 1H, NH), 5.99 (d, *J* = 4.7 Hz, 1H, H1), 4.98 (d, *J* = 11.6 Hz, 1H, Ph*CH2*), 4.69 (ddd, *J* = 16.3, 9.5, 3.6 Hz, 3H, Ph*CH2*, H2), 4.39 (d, *J* = 11.8 Hz, 1H, Ph*CH2*), 4.18 (q, *J* = 6.4 Hz, 1H, H5), 3.84 (s, 3H, OCH_3), 3.79 (t, *J* = 2.0 Hz, 1H, H4), 3.52 (dd, *J* = 11.1, 2.5 Hz, 1H, H3), 1.25 (d, *J* = 6.3 Hz, 3H, H6). **^{13}C NMR (126 MHz, $CDCl_3$)** δ 159.92 (C=O), 138.12, 134.14, 129.75, 129.44, 129.11, 128.73, 128.50, 128.43, 128.11, 127.99, 114.46(Ar*C*-PMB), 88.83 (C1), 77.32 (C3), 75.00 (Ph*C*H2), 74.45 (C4), 70.98 (Ph*C*H2), 70.59 (C5), 55.50 (O*C*H3), 50.71 (C2), 16.94 (C6). **ESI HRMS:** $[M + Na]^+$ calculated for $C_{29}H_{30}F_3NO_5SeNa$, 632.1139; found 632.1130.

***Tert*-butyldiphenylsilyl 4-*O*-benzyl-2-deoxy-3-*O*-(*p*-methoxybenzyl)-2-*N*-trifluoroacetamide-β-D-fucopyranoside (S13)**

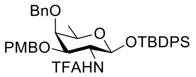

To a solution of compound **S11** (9.0 g, 15.1 mmol) in acetone and water (4/1, v/v) was added *N*-iodosuccinimide (6.8 g, 30.2 mmol) at 0 °C. The resulting dark purple mixture was stirred at 0 °C until TLC (petroleum ether/acetone, 4/1) indicated complete conversion of the starting material. Acetone was evaporated and the given residue was dissolved in DCM (100 mL), washed with sat. $Na_2S_2O_3$ (100 mL), sat. $NaHCO_3$ (100 mL), brine (100 mL), dried with Na_2SO_4, and concentrated *in vacuo*. Then the crude hemiacetal **S12** compounds were dissolved in DCM, followed by the addition of imidazole (2.6 g, 37.8 mmol), catalytic amount of DMAP and TBDPSCl (4.57 g, 4.3 mL, 16.6 mmol). The resulting mixture was stirred at room temperature for overnight until TLC (petroleum ether/EtOAc, 10/1) indicated complete conversion of the starting material. The mixture was washed with 1 M HCl (100 mL), sat. $NaHCO_3$ (100 mL) and brine (100 mL). The organic phase was separated, dried with Na_2SO_4, and concentrated *in vacuo*. The residue was purified by column chromatography (silica gel, petroleum ether/EtOAc, 10/1) to yield the title compound **S13** (8.75 g, 82% yield). **^{1}H NMR (500 MHz, $CDCl_3$)** δ 7.73 – 7.67 (m, 2H, ArH), 7.62 – 7.57 (m, 2H, ArH), 7.44 – 7.26 (m, 11H, ArH), 7.20 – 7.13 (m, 2H, ArH), 6.88 – 6.82 (m, 2H, ArH), 6.06 (d, *J* = 8.2 Hz, 1H, NH), 4.94 (d, *J* = 11.8 Hz, 1H, Ph*CH2*), 4.69 (d, *J* = 7.9 Hz, 1H, H1), 4.65 (d, *J* = 11.8 Hz, 1H, Ph*CH2*), 4.56 (d, *J* = 11.3 Hz, 1H, Ph*CH2*), 4.31 (d, *J* = 11.3 Hz, 1H, Ph*CH2*), 4.03 (dt, *J* = 11.0, 8.0

Hz, 1H, H2), 3.80 (s, 3H, OCH_3), 3.75 (dd, *J* = 11.0, 2.8 Hz, 1H, H3), 3.55 (dd, *J* = 2.9, 1.0 Hz, 1H, H4), 3.26 – 3.19 (m, 1H, H5), 1.08 (d, *J* = 6.4 Hz, 3H, H6), 1.04 (s, 9H, *t*Bu). **^{13}C NMR (126 MHz, $CDCl_3$)** δ 159.65 (C=O), 157.10 (C=O), 138.57, 136.21, 135.40, 133.31, 133.15, 129.91, 129.79, 129.73, 129.58, 128.42, 128.34, 127.83, 127.68, 127.36, 114.12 (Ar*C*-PMB), 94.94 (C1), 77.60 (C3), 74.73 (Ph*C*H_2), 74.61 (C4), 71.49 (Ph*C*H_2), 70.65 (C5), 56.21 (C2), 55.43 (OCH_3), 26.95 (*t*Bu*C*), 19.29 (Si-*Ct*Bu), 16.88 (C6). **ESI HRMS:** $[M + Na]^+$ calculated for $C_{39}H_{44}F_3NO_6SNa$, 730.2788; found 730.2781.

***Tert*-butyldiphenylsilyl 4-*O*-benzyl-2-deoxy-2-*N*-trifluoroacetamide-β-D-fucopyranoside (14)**

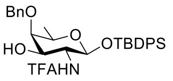

To a solution of compound **S13** (8.0 g, 11.32 mmol) in DCM and water (10/1, v/v) was added DDQ (3.85 g, 16.98 mmol) at 0 °C. The resulting dark purple mixture was stirred from 0 °C to room temperature until TLC (petroleum ether/EtOAc, 6/1) indicated complete conversion of the starting material. The reaction was quenched by sat. $Na_2S_2O_3$ (10 mL) and washed with sat. $NaHCO_3$ (50 mL), brine (50 mL). The organic phase was separated, dried with Na_2SO_4, and concentrated *in vacuo*. The residue was purified by column chromatography (silica gel, petroleum ether/EtOAc, 6/1) to yield the title compound **14** (6.35 g, 96% yield). **^{1}H NMR (400 MHz, $CDCl_3$)** δ 7.86 – 7.77 (m, 2H, ArH), 7.75 – 7.71 (m, 2H, ArH), 7.52 – 7.29 (m, 10H, ArH), 6.77 (d, *J* = 9.0 Hz, 1H, NH), 4.75 (s, 2H, Ph*CH₂*), 4.55 (d, *J* = 7.9 Hz, 1H, H1), 4.21 – 4.05 (m, 1H, H2), 3.58 (td, *J* = 10.3, 3.4 Hz, 1H, H3), 3.48 (dd, *J* = 3.4, 1.1 Hz, 1H, H4), 3.30 – 3.21 (m, 1H, H5), 3.04 (d, *J* = 10.0 Hz, 1H, OH), 1.16 (d, *J* = 10.5 Hz, 12H, H6, *t*Bu). **^{13}C NMR (101 MHz, $CDCl_3$)** δ 158.65 (C=O), 158.28 (C=O), 157.91 (C=O), 157.54 (C=O), 138.08, 136.04, 135.76, 133.18, 133.02, 129.92, 129.74, 128.56, 127.94, 127.89, 127.65, 127.33, 120.21, 117.34 (CF_3), 114.48 (CF_3), 111.61(CF_3), 95.43 (C1), 78.92 (C4), 75.79 (Ph*C*H_2), 71.71 (C3), 70.71 (C5), 56.76 (C2), 26.80(*t*Bu*C*), 19.15 (Si-*Ct*Bu), 16.57 (C6). **ESI HRMS:** $[M + Na]^+$ calculated for $C_{31}H_{36}F_3NO_5SiNa$, 610.2213; found 610.2208.

Phenyl 3,4-di-*O*-acetyl-2-azido-2-deoxy-1-thio-α/β-L-fucopyranoside (S15)

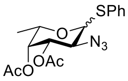

2-azido-2-deoxy-1,3,4-tri-*O*-acetyl-α-L-fucopyranoside **S14** (3.155 g, 10 mmol) was co-evaporated with toluene (x3) and dissolved in dry DCM (50 mL, 0.2 M). The solution was cooled to 0 °C followed by addition of PhSH (1 mL, 10 mmol, 1 equiv.) and $BF_3 \cdot OEt_2$ (2.5 mL, 20 mmol, 2 equiv.). The reaction mixture was stirred for 6 days under nitrogen at rt until TLC (pentane/EtOAc, 9:1) showed full conversion after which the mixture was quenched with Et_3N and diluted in DCM. The organic phase was washed with $NaHCO_3$ (sat. aq.; x1), 1 M NaOH (aq.; x3) and brine (x1), dried over Na_2SO_4, filtered and concentrated *in vacuo*. Column chromatography (pentane/EtOAc, 95:5 → 85:15) furnished **S15** in 90% yield. (3.289 g, 9 mmol) in a α/β ratio = 1.5: 1. **^{1}H NMR (400 MHz, $CDCl_3$)** δ 7.67 – 7.60 (m, 1H, Ar-*H*), 7.52 – 7.44 (m, 2H, Ar-*H*), 7.41 – 7.25 (m, 7H, Ar-*H*), 5.65 (d, *J* = 5.5 Hz, 1H, H-1α), 5.33 (dd, *J* = 3.3, 1.3 Hz, 1H, H-4α), 5.22 – 5.19 (m, 1H, H-3α), 5.17 (d, *J* = 3.2 Hz, 1H, H-4β), 4.86 (dd, *J* = 10.2, 3.2 Hz, 1H, *H*-3β), 4.67 – 4.58 (m, 1H, H-5α), 4.50 (d, *J* = 10.1 Hz, 1H, H-1β), 4.29 (dd, *J* = 11.1, 5.5 Hz, 1H, H-2α), 3.78 (qd, *J* = 6.4, 1.1 Hz, 1H, H-5β), 3.64 (t, *J* = 10.2 Hz, 1H, H-2β), 2.19 (s, 3H, CO*CH₃*α), 2.12 (s, 3H, CO*CH₃*β), 2.07 (s, 3H, CO*CH₃*α), 2.04 (s, 3H, CO*CH₃*β), 1.24 (d, *J* = 6.4 Hz, 3H, *H*-6β), 1.14 (d, *J* = 6.5 Hz, 3H, *H*-6α). **^{13}C NMR (101 MHz, $CDCl_3$)** δ 170.48 (*C*=O), 169.81 (*C*=O), 133.47 (Ar-*C*), 133.29 (Ar-C_q), 132.20 (Ar-*C*), 129.27 (Ar-*C*), 129.09 (Ar-*C*), 128.54 (Ar-*C*), 127.90 (Ar-*C*), 87.26 (C-1α), 86.57 (C-1β), 73.52 (C-3β), 73.23 (C-5β), 70.73 (C-4α), 70.50 (C-4β), 69.76 (C-3α), 65.99 (C-5α), 59.43 (C-2β), 58.28 (C-2α), 20.81 (CO*C*H_3), 20.77 (CO*C*H_3), 16.74 (C-6β), 16.00 (C-6α). **ESI HRMS**: $[M+Na]^+$ calculated for $C_{16}H_{19}N_3O_5SNa$: 388.09431; found 388.09376

Phenyl 2-azido-2-deoxy-1-thio-α/β-L-fucopyranoside (S16)

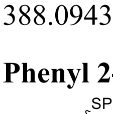

S15 (3.273 g, 8.957 mmol) was dissolved in MeOH (30 mL, 0.3 M) followed by addition of NaOMe (25 wt.% in MeOH, 0.2 mL, mmol, 0.1 equiv.). The resulting solution was stirred for 3 h at rt until TLC (pentane/EtOAc, 7:3) showed full conversion. The reaction was quenched with Amberlite (IR-120, H^+ form), filtered and concentrated *in vacuo* to yield diol **S16** in 92% (2.318 g, 8.24 mmol) in a α/β = 1:1.7. **^{1}H NMR (400 MHz, $CDCl_3$)** δ 7.64 – 7.54 (m, 2H, Ar-*H*), 7.53 – 7.46 (m, 1H, Ar-*H*), 7.40 – 7.28 (m, 7H, Ar-*H*), 5.61 (d, *J* = 5.5 Hz, 1H, H-1α), 4.54 – 4.47 (m, 1H, *H*-5α), 4.42 (d, *J* = 10.0 Hz, 1H, H-

1β), 4.16 – 4.06 (m, 1H, H-2α), 3.90 – 3.84 (m, 2H, H-3α, H-4α), 3.74 (dd, *J* = 3.2, 1.1 Hz, 1H, H-4β), 3.63 (qd, *J* = 6.5, 1.1 Hz, 1H, H-5β), 3.54 (dd, *J* = 9.5, 3.2 Hz, 1H, H-3β), 3.50 – 3.41 (m, 1H, H-2β), 2.29 (s, 4H, 3-O*H*, 4-O*H*), 1.36 (d, *J* = 6.5 Hz, 3H, H-6β), 1.30 (d, *J* = 6.6 Hz, 3H, H-6α). **^{13}C NMR (101 MHz, $CDCl_3$)** δ 133.79 (Ar-*C$_q$*), 133.16 (Ar-*C*), 132.10 (Ar-*C*), 131.97 (Ar-*C$_q$*), 129.23 (Ar-*C*), 129.18 (Ar-*C*), 128.42 (Ar-*C*), 127.76 (Ar-*C*), 87.31 (C-1α), 86.67 (C-1β), 74.82 (C-5β), 74.53 (C-3β), 71.63 (C-3α), 71.12 (C-4β), 70.59 (C-4α), 67.16 (C-5α), 63.00 (C-2β), 61.25 (C-2α), 16.81 (C-6β), 16.18 (C-6α). **ESI HRMS**: $[M+Na]^+$ calculated for $C_{12}H_{15}N_3O_3SNa$: 304.07318; found 304.07263

Phenyl 2-azido-2-deoxy-3-*O*-(2-naphthylmethyl)-1-thio-α/β-L-fucopyranoside (S17)

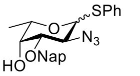

S16 (2.314 g, 8.226 mmol) in dry toluene (40 mL, 0.2 M) was added Bu_2SnO (2.089 g, 8.39 mmol, 1.02 equiv.) and the flask was equipped with a Dean-Stark apparatus. The reaction mixture was stirred at 140 °C for 3 h under nitrogen after which it was cooled to 60 °C followed by addition of CsF (1.274 g, 8.39 mmol, 1.02 equiv.), Bu_4NBr (2.784 g, 8.637 mmol, 1.05 equiv.) and NapBr (1.909 g, 8.637 mmol, 1.05 equiv.). The mixture was heated to 120 °C for 1 h until TLC (pentane/EtOAc, 6:4) showed full conversion. The reaction mixture was cooled to rt and quenched with 10% KF (aq.). After stirring for 30 minutes, the aqueous phase was extracted with EtOAc (x3). The combined organic phases were washed with brine (x1), dried over Na_2SO_4, filtered and concentrated *in vacuo*. Column chromatography (pentane/EtOAc, 95:5 → 80:20) yielded alcohol **S17** in 100% (3.467 g, 8.22 mmol) in a α/β = 1:1.7. **^{1}H NMR (400 MHz, $CDCl_3$)** δ 7.91 – 7.77 (m, 7H, Ar-*H*), 7.65 – 7.54 (m, 3H, Ar-*H*), 7.54 – 7.44 (m, 7H, Ar-*H*), 7.36 – 7.22 (m, 7H, Ar-*H*), 5.59 (d, *J* = 5.5 Hz, 1H, H-1α), 4.96 – 4.79 (m, 4H, ArC*H$_2$*α/β), 4.45 – 4.37 (m, 1H, H-5α), 4.34 (d, *J* = 10.2 Hz, 1H, H-1β), 4.27 (dd, *J* = 10.4, 5.5 Hz, 1H, H-2α), 3.94 – 3.88 (m, 1H, H-4α), 3.84 – 3.75 (m, 2H, H-3α, H-4β), 3.60 (t, *J* = 9.8 Hz, 1H, H-2β), 3.51 (qt, *J* = 6.5, 1.1 Hz, 1H, H-5β), 3.44 (dd, *J* = 9.5, 3.2 Hz, 1H, H-3β), 2.41 (t, *J* = 1.6 Hz, 1H, O*H*α), 2.19 (dd, *J* = 3.2, 1.1 Hz, 1H, O*H*β), 1.36 (d, J = 6.5 Hz, 3H, H-6β), 1.29 (d, J = 6.5 Hz, 3H, H-6α**). ^{13}C NMR (101 MHz, $CDCl_3$)** δ 134.54 (Ar-*C$_q$*), 134.51 (Ar-*C$_q$*), 133.83 (Ar-*C$_q$*), 133.40 (Ar-*C*), 133.31 (Ar-*C$_q$*), 133.28 (Ar-*C$_q$*), 131.92 (Ar-*C*), 131.77 (Ar-*C$_q$*), 129.19 (Ar-*C*), 129.10 (Ar-*C*), 128.78 (Ar-*C*), 128.72 (Ar-*C*), 128.34 (Ar-*C*), 128.12 (Ar-*C*), 128.06 (Ar-*C*), 127.89 (Ar-*C*), 127.62 (Ar-*C*), 127.21 (Ar-*C*), 127.17 (Ar-*C*), 126.52 (Ar-*C*), 126.49 (Ar-*C*), 126.44 (Ar-*C*), 126.41 (Ar-*C*), 125.87 (Ar-*C*), 125.83 (Ar-*C*), 87.38 (C-1α), 86.22 (C-1β), 81.34 (C-3β), 78.34 (C-3α), 74.54 (C-5β), 72.42 (Ar-*C*H_2α), 72.23 (Ar-*C*H_2β), 68.95 (C-4α), 68.26 (C-4β), 67.03 (C-5α), 61.04 (C-2β), 59.74 (C-2α), 16.93 (C-6β), 16.25 (C-6α). **ESI HRMS**: $[M+Na]^+$ calculated for $C_{23}H_{23}N_3O_3SNa$: 444.13578; found 444.13523

Phenyl 2-azido-4-*O*-benzoyl-2-deoxy-3-*O*-(2-naphthylmethyl)-1-thio-α/β-L-fucopyranoside (12a)

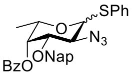

S17 (3.553 g, 8.43 mmol) was co-evaporated with toluene (x3) before dissolving in DCM/pyridine (42 mL, 4:1, 0.2 M). The reaction mixture was cooled to 0 °C followed by addition of DMAP (103 mg, 0.843 mmol, 0.1 equiv.) and BzCl (1.2 mL, 10.12 mmol, 1.2 equiv.). The reaction mixture was allowed to warm to rt and stirred overnight under nitrogen. When TLC (pentane/EtOAc, 8:2) showed full conversion, the reaction mixture was quenched by the addition of H_2O, diluted in EtOAc, washed with HCl (1 M, x3), $NaHCO_3$ (sat. aq.; x3) and brine (x1), dried over Na_2SO_4, filtered and concentrated *in vacuo*. Column chromatography (pentane/EtOAc; 95:5 → 90:10) furnished **12a** in 88% (α: 2.184 g, 4.15 mmol; β: 1.712 g, 3.26 mmol) in a α/β = 56:44. NMR reported for the α-anomer: **^{1}H NMR (400 MHz, $CDCl_3$)** δ 8.15 – 8.08 (m, 2H, Ar-*H*), 7.78 (m, *J* = 12.5, 6.1, 3.1 Hz, 4H, Ar-*H*), 7.63 – 7.54 (m, 1H, Ar-*H*), 7.53 – 7.41 (m, 8H, Ar-*H*), 7.36 – 7.26 (m, 2H, Ar-*H*), 5.76 (dd, *J* = 3.3, 1.3 Hz, 1H, H-4), 5.70 (d, *J* = 5.5 Hz, 1H, H-1), 5.01 (d, *J* = 11.1 Hz, 1H, Ar-C*H$_2$*), 4.76 (d, *J* = 11.1 Hz, 1H, Ar-C*H$_2$*), 4.71 – 4.61 (m, 1H, H-5), 4.34 (dd, *J* = 10.5, 5.5 Hz, 1H, H-2), 3.98 (dd, *J* = 10.6, 3.2 Hz, 1H, H-3), 1.23 (d, *J* = 6.5 Hz, 3H, H-6). **^{13}C NMR (101 MHz, $CDCl_3$)** δ 166.20 (*C*=O), 134.55 (Ar-*C$_q$*), 133.58 (Ar-*C$_q$*), 133.53 (Ar-*C*), 133.35 (Ar-*C$_q$*), 133.22 (Ar-*C$_q$*), 132.18 (Ar-*C*), 130.04 (Ar-*C*), 129.68 (Ar-*C$_q$*), 129.25 (Ar-*C*), 128.67 (Ar-*C*), 128.35 (Ar-*C*), 128.13 (Ar-*C*), 127.81 (Ar-*C*), 127.79 (Ar-*C*), 127.32 (Ar-*C*), 126.16 (Ar-*C*), 126.09 (Ar-*C*), 87.58 (C-1), 76.45 (C-3), 71.78 (Ar-C*H$_2$*), 69.86 (C-4), 66.56 (C-5), 60.14 (C-2), 16.41 (C-6). NMR reported for the β-anomer**: ^{1}H NMR (400 MHz, $CDCl_3$)** δ 7.93 – 7.86 (m, 2H, Ar-*H*), 7.84 – 7.72 (m, 4H, Ar-*H*), 7.76 – 7.65 (m, 2H, Ar-*H*), 7.64

– 7.55 (m, 1H, Ar-*H*), 7.52 – 7.38 (m, 8H, Ar-*H*), 5.60 (d, *J* = 1.1 Hz, 1H, H-4), 4.92 (d, *J* = 11.5 Hz, 1H, Ar-C*H₂*), 4.70 (d, *J* = 11.5 Hz, 1H, Ar-C*H₂*), 4.42 – 4.35 (m, 1H, H-1), 3.83 – 3.73 (m, 1H, H-5), 3.66 – 3.56 (m, 2H, H-2, H-3), 1.30 (d, *J* = 6.4 Hz, 3H, H-6). **^{13}C NMR (101 MHz, $CDCl_3$)** δ 166.13 (*C*=O), 135.00 (Ar-*C*), 134.49 (Ar-C_q), 133.50 (Ar-*C*), 133.28 (Ar-C_q), 130.38 (Ar-C_q), 130.13 (Ar-*C*), 129.50 (Ar-C_q), 129.10 (Ar-*C*), 128.71 (Ar-*C*), 128.55 (Ar-*C*), 128.44 (Ar-*C*), 128.08 (Ar-*C*), 127.81 (Ar-*C*), 127.42 (Ar-*C*), 126.23 (Ar-*C*), 126.17 (Ar-*C*), 126.15 (Ar-*C*), 85.32 (C-1), 79.39 (C-3), 73.73 (C-5), 71.71 (Ar-*C*H_2), 68.95 (C-4), 60.81 (C-2), 17.09 (C-6). **ESI HRMS**: $[M+Na]^+$ calculated for $C_{30}H_{27}N_3O_4SNa$: 548.16200; found 548.16145

1,3,4,6 Tetra-*O*-acetyl-α/β-D-mannopyranose (S18)

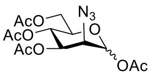

To an ice-cool solution of NaN_3 (4.522 g, 69.55 mmol, 1.5 equiv.) in pyridine (80 mL) was slowly added Tf_2O (9.3 mL, 55.65 mmol, 1.2 equiv.) and the resulting orange mixture was stirred at 0 °C for 2 h. Mannosamine hydrochloride (10 g, 46.37 mmol) was dissolved in pyridine (47 mL) and added Et_3N (12.9 mL, 92.74 mmol, 2 equiv.) and $CuSO_4$* 5 H_2O (116 mg, 0.46 mmol, 0.01 equiv.) dissolved in as little water as possible. The resulting blue mixture was cooled to 0 °C and the freshly made TfN_3 solution was added dropwise via a dropping funnel. The resulting green mixture was stirred at 0 °C for 4 h until TLC (DCM/MeOH/Et_3N 20:75:5) showed full conversion of the starting material. The solution turned yellow. Ac_2O (48.2 mL) was added to the reaction and the reaction was stirred overnight after which TLC (Pent/EtOAc 3:2) showed full conversion. The reaction was dissolved in EtOAc and the organic phase was washed with 1 M HCl aq. (x3), sat. aq. $NaHCO_3$ (sat. aq.; x3), water (x1) and brine (x1), dried over Na_2SO_4, filtered and concentrated *in vacuo*. Co-evaporated with toluene x2 to remove pyridine and **S18** was obtained in an quant. yield and a α/β ratio on 5:2. Used without further purification. **^{1}H NMR (400 MHz, $CDCl_3$)** δ 6.12 (d, J = 1.9 Hz, 1H, α-H-1), 5.84 (d, J = 1.4 Hz, 1H, β-H-1), 5.43 – 5.34 (m, 2H, α-H-4, α-H-3), 5.30 (t, J = 9.9 Hz, 1H, β-H-4), 5.07 (dd, J = 9.8, 3.7 Hz, 1H, β-H-3), 4.27 (m, 2H, α/β-H-6), 4.18 – 4.12 (m, 1H, β-H-2), 4.09 (dd, J = 12.4, 2.4 Hz, 2H, α/β-H-6), 4.06 – 3.98 (m, 2H, α-H-2, α-H-5), 3.74 (ddd, J = 9.9, 4.8, 2.3 Hz, 1H, β-H-5), 2.19 (s, 3H, β-COCH_3), 2.17 (s, 3H, α-COCH_3), 2.12 (d, J = 1.1 Hz, 6H, α/β-COCH_3), 2.10 (s, 3H, α-COCH_3), 2.09 (s, 3H, β-COCH_3), 2.06 (s, 3H, α-COCH_3), 2.05 (s, 3H, β-COCH_3). **^{13}C NMR (101 MHz, $CDCl_3$)** δ 170.93 (C=O), 170.23 (C=O), 169.53 (C=O), 168.39 (C=O), 91.52 (α -C-1), 91.33 (β-C-1), 73.45 (β-C-5), 72.02 (β-C-3), 70.89 (α -C-3), 70.70 (α-C-5), 65.43 (α-C-4), 65.01 (β-C-4), 61.90 (α-C-6), 61.84 (β-C-6), 61.20 (β-C-2), 60.65 (α-C-2), 21.05 (CO*C*H_3), 20.88 (CO*C*H_3), 20.77 (CO*C*H_3), 20.68 (CO*C*H_3). **ESI HRMS**: $[M+Na]^+$ calculated for $C_{14}H_{19}N_3O_9Na$: 396.10190; found 396.10135

Phenyl 3,4,6 tri-*O*-acetyl-2-azido-2-deoxy-1-thio-α/β-D-mannopyranoside (S19)

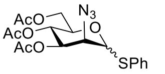

To an ice-cooled solution of **S18** (17.09 g, 45.78 mmol) in dry DCM (230 mL, 0.2 M) was slowly added PhSH (4.7 mL, 45.78 mmol, 1 equiv.) and BF_3OEt_2 (11.3 mL, 91.56 mmol, 2 equiv.) the resulting mixture was allowed to warm to rt and stirred under N_2 until TLC (Pent/EtOAc 3:2) showed full composition of the starting material (3 days). The reaction was quenched with Et_3N, diluted in DCM and the organic phase was washed with sat. aq. $NaHCO_3$ (sat. aq.; x1), 1 M NaOH (x3), water (x1) and brine (x1), dried over Na_2SO_4, filtered and concentrated *in vacuo*. Column chromatography (Pentane/EtOAc 90:10 → 70:30) gave **S19** in 88% yield (16.99 g, 40.12 mmol) and a α/β ratio on 89:11. NMR reported for the α-anomer. **^{1}H NMR (400 MHz, $CDCl_3$)** δ 7.54 – 7.44 (m, 2H, Ar-*H*), 7.37 – 7.29 (m, 3H, Ar-*H*), 5.53 (d, *J* = 1.0 Hz, 1H, *H-1*), 5.39 – 5.33 (m, 2H, H-3, H-*4*), 4.52 – 4.46 (m, 1H, *H-5*), 4.31 – 4.24 (m, 2H, H-2, H-6), 4.08 (dd, J = 12.3, 2.4 Hz, 1H, H-6), 2.12 (s, 3H, COCH_3), 2.08 (s, 3H, COCH_3), 2.06 (s, 3H, COCH_3). **^{13}C NMR (101 MHz, $CDCl_3$)** δ 170.84 (C=O), 170.11 (C=O), 169.63 (C=O), 132.08 (Ar-*C*), 129.45 (Ar-*C*), 128.40 (Ar-*C*), 85.96 (C-1), 71.30 (C-4), 69.68 (C-5), 66.17 (C-3), 62.82 (C-2), 62.29 (C-6), 20.86 (CO*C*H_3), 20.83 (CO*C*H_3), 20.72 (CO*C*H_3). **ESI HRMS**: $[M+Na]^+$ calculated for $C_{18}H_{21}N_3O_7SNa$: 446.09979; found 446.09924

Phenyl 2-azido-2-deoxy-1-thio-α-D-mannopyranoside[12] (S20)

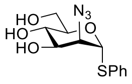

α-**S19** (14.95 g, 35.30 mmol) was dissolved in MeOH (117 mL, 0.3 M) and added NaOMe (25% wt in MeOH, 0.8 mL, 3.53 mmol, 0.1 equiv.). The reaction was stirred at rt for 3 h until TLC

(Pent/EtoAc 4:6) showed full conversion and neutralized with Amberlite IR-120 H^+ resins, filtered and concentrated. The crude **S20** was isolated in 90% (9.42 g, 31.69 mmol) and used without further purification. **^{1}H NMR (400 MHz, MeOD)** δ 7.57 – 7.50 (m, 2H, Ar-*H*), 7.36 – 7.26 (m, 3H, Ar-*H*), 5.48 (d, *J* = 1.4 Hz, 1H, H-1), 4.14 (dd, *J* = 3.8, 1.5 Hz, 1H, H-2), 4.06 – 4.01 (m, 1H, H-5), 3.94 (dd, *J* = 9.3, 3.8 Hz, 1H, H-3), 3.81 (dd, *J* = 12.1, 2.4 Hz, 1H, H-6), 3.72 (dd, *J* = 12.1, 5.7 Hz, 1H, H-6), 3.67 (t, *J* = 9.5 Hz, 1H, H-4). **^{13}C NMR (101 MHz, MeOD)** δ 135.99 (Ar- C_q), 133.73 (Ar-*C*), 130.16 (Ar-*C*), 128.31 (Ar-*C*), 87.29 (C-1), 75.82 (C-5), 73.09 (C-3), 69.30 (C-4), 67.10 (C-2), 60.72 (C-6). **HRMS**: $[M+Na]^+$ calculated for $C_{12}H_{15}N_3O_4SNa$: 320.06810; found 320.06755

Phenyl 2-azido-2-deoxy-4,6-*O*-(*p*-methoxybenzylidene)-1-thio-α-D-mannopyranoside (S21)

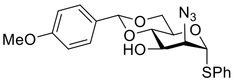

S20 (9.47 g, 31.85 mmol) was co-evaporated with toluene (x3) and dissolved in dry MeCN (160 mL, 0.2 M). Anisaldehyde dimethyl acetal (7 mL, 41.41 mmol, 2 equiv.) and camphorsulfonic acid (370 mg, 1.59 mmol, 5 mol%) was added sequentially and the reaction was stirred on the rotary evaporator (300 mbar at 50 °C) until TLC (Pent/EtOAc 7:3) showed full conversion (~1 h). The reaction was quenched with Et_3N and concentrated *in vacuo*. Column chromatography (Pentane/EtOAc 95:5 → 75:25) gave **S21** in 90% (11.93 g, 28.72 mmol). **^{1}H NMR (500 MHz, CDCl$_3$)** δ 7.50 – 7.39 (m, 4H, Ar-*H*), 7.39 – 7.29 (m, 3H, Ar-*H*), 6.95 – 6.88 (m, 2H, Ar-*H*), 5.55 (s, 1H, PMP-C*H*), 5.47 (d, *J* = 1.2 Hz, 1H, H-1), 4.33-4.28 (td, *J* = 9.7, 4.9 Hz, 1H, H-5), 4.26-4.23 (dt, *J* = 9.7, 3.9 Hz, 1H, H-3), 4.22 – 4.20 (m, 1H, H-2), 4.19 (d, *J* = 5.0 Hz, 1H, H-6), 3.81 (s, 3H, OCH_3), 3.79 (d, *J* = 10.3 Hz, 1H, H-6), 2.82 (d, *J* = 3.9 Hz, 1H, OH). **^{13}C NMR (126 MHz, CDCl$_3$)** δ 160.48 (Ar-C_q), 133.11 (Ar-C_q), 132.06 (Ar-*C*), 130.4 (Ar-C_q), 129.42 (Ar-*C*), 128.25 (Ar-*C*), 127.77 (Ar-*C*) 113.92 (Ar-*C*), 102.43 (PMB-*C*H), 87.65 (C-1), 79.16 (C-4), 69.39 (C-3), 68.43 (C-6), 65.20 (C-2), 64.73 (C-5), 55.46 (O*C*H$_3$). **ESI HRMS**: $[M+H]^+$ calculated for $C_{20}H_{21}N_3O_5SH$: 416.12802; found 416.12876

Phenyl 2-azido-2-deoxy-4,6-*O*-*p*-methoxybenzylidene-3-*O*-benzyl-1-thio-α-D-mannopyranoside (S22)

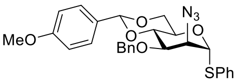

S21 (1.826 g, 4.395 mmol) was co-evaporated with toluene (x3) and dissolved in DMF (44 mL, 0.1 M). The mixture was cooled to 0°C followed by addition of NaH (229 mg, 5.173 mmol, 1.3 equiv.) and BnBr (0.68 mL, 5.713 mmol, 1.3 equiv.). The reaction mixture was allowed to warm to rt and stirred under nitrogen overnight until TLC (pentane/EtOAc, 9:1) showed full conversion. The reaction was quenched by the addition of H_2O and diluted in Et_2O. The aqueous phase was extracted with Et_2O (x3) and the combined organic phases were washed with brine (x1), dried over Na_2SO_4, filtered and concentrated *in vacuo*. Column chromatography (pentane/EtOAc, 100:0 → 90:10) provided thioglycoside **S22** in 98% yield (2.177g, 4.31 mmol). **^{1}H NMR (400 MHz, CDCl$_3$)** δ 7.47 – 7.25 (m, 12H, Ar-*H*), 6.95 – 6.87 (m, 2H, Ar-*H*), 5.59 (s, 1H, H-1), 5.43 (d, *J* = 1.3 Hz, 1H, PMPC*H*), 4.92 (d, *J* = 12.1 Hz, 1H, H-5, Ar-CH_2), 4.75 (d, *J* = 12.1 Hz, Ar-CH_2), 4.36 – 4.26 (m, 1H, H-5), 4.23 – 4.06 (m, 4H, H-2, H-3, H-4, H-6), 3.82 (t, 4H, OCH_3, H-6). **^{13}C NMR (101 MHz, CDCl$_3$)** δ 160.19 (Ar-C_q), 137.90 (Ar-C_q), 132.97 (Ar-C_q), 132.13 (Ar-*C*), 129.93 (Ar-C_q), 129.41 (Ar-*C*), 128.65 (Ar-*C*), 128.25 (Ar-*C*), 128.05 (Ar-*C*), 127.82 (Ar-*C*), 127.52 (Ar-*C*), 113.73 (Ar-*C*), 101.79 (C-1), 87.35 (Ar-*C*H), 79.23 9 (C-2, C-3, C-4), 75.86 (C-2, C-3, C-4), 73.63 (Ar-*C*H$_2$), 68.44 (C-6), 65.28 (C-5), 64.29 (C-2, C-3, C-4), 55.45 (O*C*H$_3$). **ESI HRMS**: $[M+Na]^+$ calculated for $C_{27}H_{27}N_3O_5SNa$: 528.15691; found 528.15636

Phenyl 2-azido-2-deoxy-3-*O*-benzyl-4-*O*-*p*-methoxybenzyl-1-thio-α-D-mannopyranoside (S23)

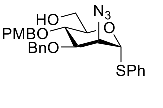

S22 (1.637 g, 3.238 mmol) was co-evaporated with toluene (x3) before being dissolved in dry DCM (32 mL, 0.1 M). 3Å molecular sieves was added and stirred for 30 min at rt. The solution cooled to 0 °C and added BH_3·THF (1 M in THF; 16 mL, mmol, 5 equiv.) and TMSOTf (0.09 mL, 0.486 mmol, 0.15 equiv.). The reaction was stirred for 3 h at rt under argon until TLC (pentane/EtOAc, 9:1) showed full conversion. The reaction mixture was quenched by the addition of NEt_3 and MeOH. The resulting solution was concentrated *in vacuo* and co-evaporated with MeOH (x3). Column chromatography (pentane/EtOAc, 9:1 → 8:2) yielded alcohol **S23** in 82% yield (1.343 g, 2.65 mmol). **^{1}H NMR (400 MHz, CDCl$_3$)** δ 7.47 – 7.25 (m, 10H, Ar-*H*), 7.29 – 7.20 (m, 2H, Ar-*H*), 6.92 – 6.84 (m, 2H, Ar-*H*) 5.41 (d, *J* = 1.5

Hz, 1H, H-1), 4.82 (d, *J* = 10.5 Hz, 1H, Ar-C*H*$_2$), 4.77 (s, 2H, Ar-C*H*$_2$), 4.60 (d, *J* = 10.5 Hz, 1H, Ar-C*H*$_2$), 4.16 – 4.07 (m, 2H, H-2, H-5), 4.04 (dd, *J* = 9.1, 3.5 Hz, 1H, H-3), 3.90 (t, *J* = 9.4 Hz, 1H, H-4), 3.81 (s, 3H, OC*H*$_3$), 3.79 – 3.73 (m, 2H, H-6), 1.72 (dd, *J* = 7.4, 5.9 Hz, 1H, O*H*). **^{13}C NMR (101 MHz, CDCl$_3$)** δ 159.57 (Ar-*C$_q$*), 137.59 (Ar-*C$_q$*), 133.09 (Ar-*C$_q$*), 132.34 (Ar-*C*), 130.24 (Ar-*C$_q$*), 129.99 (Ar-*C*), 129.38 (Ar-*C*), 128.79 (Ar-*C*), 128.29 (Ar-*C*), 128.26 (Ar-*C*), 128.23 (Ar-*C*), 114.07 (Ar-*C*), 86.47 (C-1), 79.98 (C-3), 75.25 (Ar-*C*H$_2$), 74.14 (C-4), 73.34 (C-5), 72.96 (Ar-*C*H$_2$), 62.90 (C-2), 62.02 (C-6), 55.44 (O*C*H$_3$). **ESI HRMS**: [M+Na]$^+$ calculated for $C_{27}H_{29}N_3O_5SNa$: 530.17256; found 530.17201

Benzyl (phenyl 2-azido-3-*O*-benzyl-2-deoxy-4-*O*-*p*-methoxybenzyl-1-thio-α-D-mannopyranosiduronate) (10)

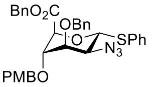

Alcohol **24** (1.678 g, 3.51 mmol) was dissolved in DCM/*t*BuOH/H_2O (18 mL, 8:4:1, 0.2 M). The mixture was cooled to 0 °C, followed by addition of TEMPO (110 mg, 0.703 mmol, 0.2 equiv.), BAIB (2.829 g, 8.785 mmol, 2.5 equiv.) and AcOH (0.02 mL, 0.351 mmol, 0.2 equiv.). The reaction mixture was stirred for 2h at rt until TLC analysis (pentane/EtOAc, 7:3) showed full conversion. The reaction mixture was quenched with $Na_2S_2O_3$ (sat. aq.) and the aqueous phase was extracted with EtOAc (x3). The organic phases were washed with brine (x1), dried over Na_2SO_4, filtered and concentrated *in vacuo*. The crude was co-evaporated with toluene (x3) before dissolving in DMF (35 mL, 0.1 M). The reaction mixture was cooled to 0 °C, followed by addition of K_2CO_3 (971 mg, 7.028 mmol, 2 equiv.) and BnBr (0.8 mL, 7.028 mmol, 2 eq). The mixture was allowed to warm to rt and stirred overnight under nitrogen until TLC analysis (pentane/EtOAc, 7:3) showed full conversion. The reaction mixture was quenched with H_2O. The aqueous phase was extracted with Et_2O (x3), and the combined the organic phases were washed with brine (x1), dried over Na_2SO_4, filtered and concentrated *in vacuo*. Column chromatography (pentane/EtOAc, 95:5 → 85:15) furnished donor **10** in 78% yield (1.493 g, 2.44 mmol). **^{1}H NMR (400 MHz, CDCl$_3$)** δ 7.57 – 7.50 (m, 2H, Ar-*H*), 7.40 – 7.12 (m, 15H, Ar-*H*), 6.88 – 6.80 (m, 2H, Ar-*H*), 5.65 (d, *J* = 7.8 Hz, 1H, H-1), 5.02 (d, *J* = 12.2 Hz, 1H Ar-C*H*$_2$), 4.91 (d, *J* = 12.2 Hz, 1H, Ar-C*H*$_2$), 4.64 (d, *J* = 4.2 Hz, 1H, Ar-C*H*$_2$), 4.56 (d, *J* = 11.1 Hz, 1H, H-5), 4.52 – 4.46 (m, 3H, Ar-C*H*$_2$), 4.20 (dd, *J* = 5.6, 4.3 Hz, 1H, H-4), 3.88 (dd, *J* = 5.6, 3.0 Hz, 1H, H-3), 3.81 (s, 3H, OC*H*$_3$), 3.68 (dd, *J* = 7.9, 2.9 Hz, 1H, H-2). **^{13}C NMR (101 MHz, CDCl$_3$)** δ 169.04 (*C*=O), 159.59 (Ar-*C$_q$*), 136.95 (Ar-*C$_q$*), 135.17 (Ar-*C$_q$*), 132.37 (Ar-*C$_q$*), 132.28 (Ar-*C*), 129.70 (Ar-*C*), 129.49 (Ar-*C$_q$*), 129.01 (Ar-*C*), 128.68 (Ar-*C*), 128.62 (Ar-*C*), 128.55 (Ar-*C*), 128.53 (Ar-*C*), 128.32 (Ar-*C*), 128.30 (Ar-*C*), 127.84 (Ar-*C*), 114.02 (Ar-*C*), 77.00 (C-3), 74.38 (C-4), 73.23 (C-5), 73.06 (Ar-*C*H$_2$), 72.83 (Ar-*C*H$_2$), 67.32 (Ar-*C*H$_2$), 55.44 (O*C*H$_3$). **ESI HRMS**: [M+Na]$^+$ calculated for $C_{34}H_{33}N_3O_6SNa$: 634.19878; found 634.19823

Phenyl 2-azido-4,6-*O*-benzylidene-2-deoxy-1-thio-α-D-mannopyranoside (S24)

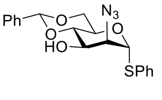

Triol **S20** (2.11 g, 7.1 mmol) was dissolved in MeCN (23 mL, 0.3 M) followed by addition of benzaldehyde dimethyl acetal (1.3 mL, 8.52 mmol, 1.2 equiv.) and CSA (165 mg, 0.71 mmol, 0.1 equiv.). The reaction mixture was stirred on the rotary evaporator at 50 °C under reduced pressure (300 mbar) for 2 h until TLC (pentane/EtOAc, 7:3) showed full conversion. The reaction was quenched by the addition of Et_3N and concentrated *in vacuo*. Purification by column chromatography (pentane/EtOAc, 90:10 → 80:20) yielded **S24** in 80% (2.00 g, 5.71 mmol). **^{1}H NMR (400 MHz, CDCl$_3$)** δ 7.54 – 7.44 (m, 4H, Ar-*H*), 7.44 – 7.38 (m, 3H, Ar-*H*), 7.38 – 7.30 (m, 3H, Ar-*H*), 5.60 (s, 1H, Ph-C*H*), 5.49 (d, *J* = 1.2 Hz, 1H, H-1), 4.37 – 4.19 (m, 4H, H-3, H-4, H-2, H-6a), 3.98 (t, *J* = 9.5 Hz, 1H, H-5), 3.83 (t, *J* = 10.3 Hz, 1H, H-6b), 2.72 (d, *J* = 3.8 Hz, 1H, O*H*). **^{13}C NMR (101 MHz, CDCl$_3$)** δ 137.05 (Ar-*C$_q$*), 133.07 (Ar-*C$_q$*), 132.09 (Ar-*C*), 129.56 (Ar-*C*), 129.44 (Ar-*C*), 128.58 (Ar-*C*), 128.29 (Ar-*C*), 126.41 (Ar-*C*), 102.48 (Ph-*C*H), 87.68 (C-1), 78.86 (C-5), 69.43 (C-3/ C-4), 68.46 (C-6), 65.18 (C-2), 64.71 (C-3/ C-4). **ESI HRMS**: [M+Na]$^+$ calculated for $C_{19}H_{19}N_3O_4SNa$: 408.09940; found 408.09885

Phenyl 2-azido-4,6-*O*-benzylidene-3-*O*-benzyl-2-deoxy-1-thio-α-D-mannopyranoside (S25)

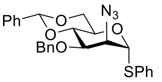

S24 (2.194 g, 5.69 mmol) was co-evaporated with toluene (x3) before being dissolved in DMF (57 mL, 0.1 M). The reaction mixture was cooled to 0 °C followed by addition of NaH (60% suspension in mineral oil, 341 mg, 8.45 mmol, 1.5 equiv.) and BnBr (1 mL, 8.54 mmol, 1.5 equiv.). The reaction was stirred overnight at rt under nitrogen until TLC (pentane/EtOAc, 8:2) showed full conversion. The reaction mixture was quenched by addition of H_2O and diluted in Et_2O. The aqueous phase was extracted with Et_2O (x3) and the combined organic phases were washed with brine (x1), dried over Na_2SO_4, filtered and concentrated *in vacuo*. Column chromatography (pentane/EtOAc, 98:2 → 90:10) provided **S25** in 100 % yield (2.75 g, 5.69 mmol) **^{1}H NMR (400 MHz, CDCl$_3$)** δ 7.54 – 7.49 (m, 2H, Ar-*H*), 7.45 – 7.29 (m, 14H, Ar-*H*), 5.64 (s, 1H, Ph-C*H*-1), 5.44 (d, *J* = 1.2 Hz, 1H, *H*-1), 4.94 (d, *J* = 12.1 Hz, 1H, Ar-C*H*$_2$), 4.76 (d, *J* = 12.1 Hz, 1H, Ar-C*H*$_2$), 4.33 (ddd, *J* = 9.9, 8.9, 4.7 Hz, 1H, H-5), 4.25 – 4.16 (m, 3H, H-6, H-4, H-2), 4.16 – 4.12 (m, 1H, H-3), 3.85 (t, *J* = 10.2 Hz, 1H, *H*-6). **^{13}C NMR (101 MHz, CDCl$_3$)** δ 137.88 (Ar-*C$_q$*), 137.42 (Ar-*C$_q$*), 132.14 (Ar-*C$_q$*), 129.42 (Ar-*C*), 129.16, 128.66 (Ar-*C*), 128.40 (Ar-*C*), 128.27 (Ar-*C*), 128.07 (Ar-*C*), 127.83 (Ar-*C*), 126.20 (Ar-*C*), 101.79 (Ph-*C*H), 87.36 (C-1), 79.30 (C-4), 75.86 (C-3), 73.64 (Ar-*C*H$_2$), 68.49 (C-6), 65.26 (C-5), 63.77 (C-2). **ESI HRMS**: [M+Na]$^+$ calculated for $C_{26}H_{25}N_3O_4SNa$: 498.14635; found 498.14580

Phenyl 2-azido-2-deoxy-3,4-di-*O*-benzyl-1-thio-α-D-mannopyranoside (S26)

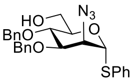

S25 (2.356 g, 4.659 mmol) was co-evaporated with toluene (x3) and dissolved in dry DCM (46 mL, 0.1 M). 3Å molecular sieves was added and stirred for 30 min and rt. The solution cooled to 0 °C and added BH_3·THF (1 M in THF; 23 mL, 23.29 mmol, 5 equiv.) and TMSOTf (0.13 mL, 0.699 mmol, 0.15 equiv.). The reaction was stirred for 4 h at rt under argon until TLC (pentane/EtOAc, 9:1) showed full conversion. The reaction was quenched with NEt_3 and MeOH. The mixture was concentrated *in vacuo* and co-evaporated with MeOH (x3). Column chromatography (pentane/EtOAc, 9:1 → 8:2) gave **S26** in 100 % yield (2.252 g, 4.659 mmol). **^{1}H NMR (400 MHz, CDCl$_3$)** δ 7.45 – 7.28 (m, 15H, Ar-*H*), 5.42 (d, *J* = 1.5 Hz, 1H, *H*-1), 4.92 (d, *J* = 10.9 Hz, 1H, Ar-C*H*$_2$), 4.77 (s, 2H, Ar-C*H*$_2$), 4.67 (d, *J* = 10.9 Hz, 1H, Ar-C*H*$_2$), 4.17 – 4.11 (m, 1H, H-5), 4.12 – 4.09 (m, 1H, H-2), 4.06 (dd, *J* = 9.0, 3.6 Hz, 1H, H-3), 3.93 (dd, *J* = 9.7, 9.1 Hz, 1H, H-4), 3.84 – 3.74 (m, 2H, H-6), 1.75 (dd, *J* = 7.4, 6.0 Hz, 1H, O*H*). **^{13}C NMR (101 MHz, CDCl$_3$)** δ 138.09 (Ar-*C$_q$*), 137.53 (Ar-*C$_q$*), 133.07 (Ar-*C$_q$*), 132.34 (Ar-*C*), 129.39 (Ar-*C*), 128.79 (Ar-*C*), 128.66 (Ar-*C*), 128.30 (Ar-*C*), 128.10 (Ar-*C*), 86.47 (C-1), 79.95 (C-3), 75.5 (Ar-*C*H$_2$), 74.42 (C-4), 73.34 (C-5), 72.96 (Ar-*C*H$_2$), 63.36 (C-2), 61.97 (C-6). **ESI HRMS**: [M+Na]$^+$ calculated for $C_{26}H_{27}N_3O_4SNa$: 500.16200; found 500.16145

Benzyl (phenyl 2-azido-3,4-di-*O*-benzyl-2-deoxy-1-thio-α-D-mannopyranosiduronate) (11)

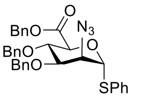

Alcohol **S26** (2.473 g, 5.178 mmol) was dissolved in DCM/*t*-BuOH/H_2O (26 mL, 8:4:1, 0.2 M). TEMPO (162 mg, 1.036 mmol, 0.2 equiv.), BAIB (4.169 g, 12.944 mmol, 2.5 equiv.) and AcOH (30 µL, 0.518 mmol, 0.1 equiv.) were added and the reaction mixture was stirred at rt for 2 h until TLC (pentane/EtOAc, 7:3) showed full conversion. The reaction was quenched with $Na_2S_2O_3$ (sat. aq.) and the aqueous phase was extracted with DCM (x3). The combined organic phases were washed with brine (x1), dried over Na_2SO_4, filtered and concentrated *in vacuo*. The crude was co-evaporated with toluene (x3) before being dissolved in DMF (52 mL, 0.1 M). The solution was cooled to 0 °C before K_2CO_3 (1.431 g, 10.356 mmol, 2 equiv.) and BnBr (1.2 mL, 10.356 mmol, 2 equiv.) were added. The reaction was stirred at rt overnight under nitrogen until TLC (pentane/EtOAc, 7:3) showed full conversion. The reaction was quenched by the addition of H_2O and the mixture was diluted in Et_2O. The aqueous phase was extracted with Et_2O (x3) and the combined organic phases were washed with brine (x1), dried over Na_2SO_4, filtered and concentrated *in vacuo*. Column chromatography (pentane/EtOAc, 95:15 → 85:15) furnished donor **11** in 79% yield (2.393 g, 4.11 mmol). **^{1}H NMR (400 MHz, CDCl$_3$)** δ 7.57 – 7.52 (m, 2H, Ar-*H*), 7.40 – 7.27 (m, 13H, Ar-*H*), 7.24 – 7.15 (m, 7H, Ar-*H*), 5.66 (d, *J* = 7.8 Hz, 1H, H-1), 5.03 (d, *J* = 12.1 Hz, 1H, Ar-C*H*$_2$), 4.91 (d, *J* = 12.2 Hz, 1H, Ar-C*H*$_2$), 4.67 (d, *J* = 4.2 Hz, 1H, H-5), 4.61 (q, *J* = 11.4 Hz, 2H, Ar-C*H*$_2$), 4.51 (d, *J* = 2.3 Hz, 2H, Ar-C*H*$_2$), 4.22 (dd, *J* = 5.7, 4.2 Hz, 1H, H-4), 3.92 (dd, *J* = 5.6, 3.0 Hz, 1H, H-3), 3.70 (dd, *J* = 7.8, 2.9 Hz, 1H, H-2). **^{13}C**

NMR (101 MHz, $CDCl_3$) δ 168.98 (*C*=O), 137.41 (Ar-C_q), 136.90 (Ar-C_q), 135.13 (Ar-C_q), 132.37 (Ar-*C*), 132.24 (Ar-C_q), 129.01 (Ar-*C*), 128.67 (Ar-*C*), 128.62 (Ar-*C*), 128.54 (Ar-*C*), 128.52 (Ar-*C*), 128.33 (Ar-*C*), 128.31 (Ar-*C*), 128.13 (Ar-*C*), 127.95 (Ar-*C*), 127.88 (Ar-*C*), 82.55 (C-1), 76.84 (C-3), 74.74 (C-4), 73.17 (C-5), 73.08 (Ar-CH_2), 67.33 (Ar-CH_2), 58.97 (C-2). **ESI HRMS**: $[M+Na]^+$ calculated for $C_{33}H_{31}N_3O_5SNa$: 604.18821; found 604.18766

Synthesis of longer fragments

***Tert*-butyldiphenylsilyl 2-azido-4-*O*-benzoyl-2-deoxy-3-*O*-(2-naphthylmethyl)-α-L-fucopyranosyl-(1→3)-2-deoxy-2-*N*-trichloroacetamide-4-*O*-benzyl-β-D-fucopyranoside (15)**

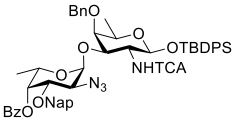

Acceptor **13** (1.605 g, 2.52 mmol, 1 equiv.) and donor **12a-α** (1.721 g, 3.275 mmol, 1.3 equiv.) were co-evaporated with toluene (x3) before being dissolved in dry DCM (25 mL, 0.1 M). Activated 3Å molecular sieves were added and the solution was stirred for 30 min under argon at rt. The reaction was cooled to -60 °C followed by addition of NIS (850 mg, 3.779 mmol, 1.5 equiv.) and TMSOTf (91 µL, 0.504 mmol, 0.2 equiv.). The reaction was allowed to warm to -30 °C and stirred for 1 h under argon until TLC (pentane/EtOAc, 8:2) showed full conversion. The reaction was quenched with NEt_3 at -30 °C and diluted in EtOAc. The organic phase was washed $Na_2S_2O_3$ (sat. aq.; x1), $NaHCO_3$ (sat. aq.; x1) and brine (x1), dried over Na_2SO_4, filtered and concentrated *in vacuo*. Column chromatography (pentane/EtOAc, 95:5 → 80:20) yielded the α-1,3-linked disaccharide **15** in 98% yield (α: 2.331 g, 2.21 mmol; β: 263 mg, 0.25 mmol) in a α/β = 9:1. **^{1}H NMR (400 MHz, $CDCl_3$)** δ 8.08 – 8.01 (m, 2H, Ar-*H*), 7.81 – 7.71 (m, 6H, Ar-*H*), 7.71 – 7.62 (m, 2H, Ar-*H*), 7.61 – 7.52 (m, 1H, Ar-*H*), 7.48 – 7.25 (m, 15H, Ar-*H*), 7.20 (d, *J* = 6.9 Hz, 1H, *H*N(CO)CCl_3), 5.49 (dd, J = 3.3, 1.3 Hz, 1H, H-4'), 5.01 (d, *J* = 3.7 Hz, 1H, H-1'), 4.97 (d, *J* = 7.3 Hz, 1H, H-1), 4.90 (d, *J* = 11.0 Hz, 1H, Ar-CH_2), 4.78 (d, *J* = 12.2 Hz, 1H, Ar-CH_2), 4.69 (d, *J* = 12.2 Hz, 1H, Ar-CH_2), 4.59 (d, *J* = 11.0 Hz, 1H, Ar-CH_2), 4.17 – 4.01 (m, 3H, H-2, H-3, H-5'), 3.96 (dd, *J* = 10.5, 3.2 Hz, 1H, H-3'), 3.80 (dd, *J* = 10.5, 3.6 Hz, 1H, H-2'), 3.49 (d, *J* = 1.9 Hz, 1H, *H*-4), 3.33 (q, *J* = 6.3 Hz, 1H, H-5), 1.07 (t, 15H, $(CH_3)_3$), H-6, H-6'). **^{13}C NMR (101 MHz, $CDCl_3$)** δ 166.18 (*C*=O), 161.93 (*C*=O), 138.83 (Ar-C_q), 136.21 (Ar-*C*), 135.98 (CH_{Ar}), 134.73 (Ar-$C_{q\ r}$), 133.71 (Ar-C_q), 133.51 (Ar-C_q), 133.47 (Ar-*C*), 133.35 (Ar-C_q), 133.14 (Ar-C_q), 129.98 (Ar-*C*), 129.73 (Ar-*C*), 129.63 (Ar-*C*), 129.62 (Ar-*C*), 128.63 (Ar-C_q), 128.55 (Ar-*C*), 128.25 (Ar-*C*), 128.10 (Ar-*C*), 127.75 (Ar-*C*), 127.55 (Ar-*C*), 127.32 (Ar-*C*), 127.09 (Ar-*C*), 127.01 (Ar-*C*), 126.10 (Ar-*C*), 126.02 (Ar-*C*), 99.45 (C-1'), 94.78 (C-1), 79.55 (C-4), 78.54 (C-3), 75.22 (Ar-CH_2), 75.18 (C-3'), 71.51 (Ar-CH_2), 70.70 (C-5), 69.64 (C-5'), 66.17 (C-4'), 60.15 (C-2'), 57.63 (C-2), 27.16 ($(CH_3)_3$), 19.40 ($C(CH_3)_3$), 16.93 (C-6'), 16.48 (C-6). **ESI HRMS**: $[M+Na]^+$ calculated for $C_{55}H_{57}Cl_3N_4O_9SiNa$: 1073.28581; found 1073.28526

***Tert*-butyldiphenylsilyl 2-azido-4-*O*-benzoyl-2-deoxy-3-*O*-(2-naphthylmethyl)-α-L-fucopyranosyl-(1→3)-2-deoxy-2-*N*-triflouroacetamide-4-*O*-benzyl-β-D-fucopyranoside (16)**

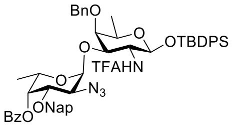

The selenoglycoside donor **12b** (6.2 g, 11.6 mmol, 2.0 eq) and acceptors **14** (3.4 g, 5.8 mmol) were co-evaporated with toluene for three times. The residue was dissolved in dry DCM (58 mL, 0.1 M). The reaction mixture was cooled to -78 °C and followed by adding NIS (3.9 g, 17.4 mmol, 3.0 eq) and TBSOTf (307 mg, 1.16 mmol, 0.2 eq). The reaction was allowed to stir for 1 h from -78 °C to 0 °C until all acceptor was consumed completely. The reaction was quenched with Et_3N, diluted in DCM, washed with sat. $Na_2S_2O_3$, brine, and the organic phase was dried over Na_2SO_4, concentrated *in vacuo*. Purification of the crude residue by column chromatography (silica gel, petroleum ether/EtOAc, 8/1) afforded the title compound **16** (5.35 g, 92% yield). **^{1}H NMR (500 MHz, $CDCl_3$)** δ 8.04 (dd, *J* = 8.4, 1.3 Hz, 2H, ArH), 7.79 – 7.69 (m, 6H, ArH), 7.65 – 7.61 (m, 2H, ArH), 7.55 (ddt, *J* = 8.7, 7.2, 1.3 Hz, 1H, ArH), 7.46 – 7.27 (m, 16H, ArH), 6.65 (d, *J* = 8.0 Hz, 1H, NH), 5.48 (dd, *J* = 3.2, 1.3 Hz, 1H, H_{4L}), 4.93 – 4.88 (m, 2H, H_{1L}, Ar*C*H_2), 4.79 (d, *J* = 12.2 Hz, 1H, Ar*C*H_2), 4.75 – 4.68 (m, 2H, H_{1D}, Ar*C*H_2), 4.60 (d, *J* = 10.8 Hz, 1H, Ar*C*H_2), 4.19 (dt, *J* = 11.1, 7.9 Hz, 1H, H_{2D}), 4.10 – 4.03 (m, 1H, H_{5L}), 3.95 – 3.83 (m, 3H, H_{3L}, H_{3D}, H_{2L}), 3.50 (d, *J* = 2.9 Hz, 1H, H_{4D}), 3.31 – 3.25 (m, 1H, H_{5D}), 1.10 (d, *J* = 6.4

Hz, 3H, H6), 1.07 (s, 9H, *t*Bu), 1.05 (d, *J* = 6.5 Hz, 3H, H6). **^{13}C NMR (126 MHz, CDCl$_3$)** δ 166.12 (C=O), 157.85 (F$_3$CC=O), 157.56 (F$_3$CC=O), 157.27 (F$_3$CC=O), 138.72, 136.22, 135.95, 134.73, 133.45, 133.40, 133.36, 133.16, 133.15, 129.96, 129.94, 129.75, 128.62, 128.54, 128.20, 128.11, 127.77, 127.74, 127.68, 127.36, 127.16, 127.12, 126.14, 126.06, 126.00, 119.47 (CF$_3$), 117.17 (CF$_3$), 114.87 (CF$_3$), 112.58 (CF$_3$), 99.63 (C$_{1L}$), 95.25 (C$_{1D}$), 79.85 (C$_{3D}$), 78.98 (C$_{4D}$), 75.51 (C$_{3L}$), 75.18 (Ar*C*H$_2$), 71.68 (Ar*C*H$_2$), 70.77 (C$_{5D}$), 69.67 (C$_{4L}$), 66.43 (C$_{5L}$), 60.54 (C$_{2L}$), 55.80 (C$_{2D}$), 26.97 (*t*Bu), 19.30 (*t*BuC), 16.94 (C6), 16.44 (C6). **ESI HRMS:** [M + Na]$^+$ calculated for C$_{55}$H$_{57}$F$_3$N$_4$O$_9$SiNa, 1025.3745; found 1025.3749.

***Tert*-butyldiphenylsilyl 2-azido-2-deoxy-3-*O*-(2-naphthylmethyl)-α-L-fucopyranosyl-(1→3)-2-deoxy-2-*N*-trichloroacetamide-4-*O*-benzyl-β-D-fucopyranoside (17)**

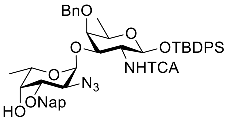

Disaccharide **15** (2.296 g, 2.182 mmol) was dissolved in MeOH (11 mL, 0.2 M), followed by addition of NaOMe (25% wt. in MeOH, 0.5 mL, 2.182 mmol, 1 equiv.). The reaction mixture was stirred for 2 days at rt until TLC analysis (pentane/EtOAc, 8:2) showed full conversion. The reaction mixture was neutralized by the addition of Amberlite (IR-120, H$^+$ form) until pH≈8-9, filtered and concentrated *in vacuo*. Column chromatography (pentane/EtOAc, 90:10 → 70:30) provided acceptor **17** in 90% yield (1.852 g, 1.95 mmol). **^{1}H NMR (400 MHz, CDCl$_3$)** δ 7.88 – 7.69 (m, 6H, Ar-*H*), 7.69 – 7.61 (m, 2H, Ar-*H*), 7.52 – 7.44 (m, 3H, Ar-*H*), 7.43 – 7.22 (m, 11H, Ar-*H*), 7.13 (d, *J* = 7.5 Hz, 1H, *H*N(CO)CCl$_3$), 4.94 – 4.87 (m, 2H, H-1, H-1'), 4.82 – 4.67 (m, 4H, Ar-C*H$_2$*), 4.11 (m, 1H, H-2), 4.02 (dd, *J* = 11.1, 2.8 Hz, 1H, H-3'), 3.86 (q, *J* = 6.8 Hz, 1H, H-5'), 3.81 (dd, *J* = 10.3, 3.0 Hz, 1H, H-3), 3.73 (dd, *J* = 10.4, 3.6 Hz, 1H, H-2'), 3.68 (d, *J* = 3.0 Hz, 1H, H-4'), 3.47 (d, *J* = 3.1 Hz, 1H, H-4), 3.34 – 3.24 (m, 1H, H-5), 2.27 (s, 1H, O*H*), 1.16 (d, *J* = 6.6 Hz, 3H, H-6'), 1.05 (m, 12H, H-6, (C*H$_3$*)$_3$). **^{13}C NMR (101 MHz, CDCl$_3$)** δ 161.83 (*C*=O), 138.89 (Ar-*C$_q$*), 136.21 (Ar-*C*), 135.98 (Ar-*C*), 134.62 (Ar-*C$_q$*), 133.68 (Ar-*C$_q$*), 133.49 (Ar-*C$_q$*), 133.35 (Ar-*C$_q$*), 133.28 (Ar-*C$_q$*), 129.72 (Ar-*C*), 129.62 (Ar-*C*), 128.75 (Ar-*C*), 128.46 (Ar-*C*), 128.11 (Ar-*C*), 127.88 (Ar-*C*), 127.64 (Ar-*C*), 127.54 (Ar-*C*), 127.31 (Ar-*C*), 127.18 (Ar-*C*), 126.95 (Ar-*C*), 126.50 (Ar-*C*), 126.41 (Ar-*C*), 125.70 (Ar-*C*), 99.32 (C-1'), 94.98 (C-1), 79.25 (C-4), 78.61 (C-3'), 77.28 (C-3), 75.13 (Ar-*C*H$_2$), 72.05 (Ar-*C*H$_2$), 70.67 (C-5), 68.58 (C-4'), 66.58 (C-5'), 59.67 (C-2'), 57.43 (C-2), 27.15 ((*C*H$_3$)$_3$), 19.39 (*C*(CH$_3$)$_3$), 16.84 (C-6), 16.33 (C-6'). **ESI HRMS**: [M+Na]$^+$ calculated for C$_{48}$H$_{53}$Cl$_3$N$_4$O$_8$SiNa: 969.25959; found 969.25905

***Tert*-butyldiphenylsilyl 2-azido-2-deoxy-α-L-fucopyranosyl-(1→3)-2-deoxy-2-*N*-trichloroacetamide-4-*O*-benzyl-β-D-fucopyranoside (18)**

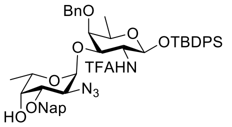

Disaccharide **16** (3.0 g, 3.0 mmol) was dissolved in MeOH (15 mL, 0.2 M), followed by addition of NaOMe (25% wt. in MeOH, 3.5 mL). The reaction mixture was stirred for 2 days at rt until TLC analysis (pentane/EtOAc, 8:2) showed full conversion. The reaction mixture was neutralized by the addition of Amberlite (IR-120, H$^+$ form) until pH≈8-9, filtered and concentrated *in vacuo*. Column chromatography (pentane/EtOAc, 90:10 → 70:30) provided acceptor **18** in 91% yield (2.45 g, mmol). **^{1}H NMR (400 MHz, CDCl$_3$)** δ 7.87 – 7.77 (m, 4H, ArH), 7.75 – 7.70 (m, 2H, ArH), 7.67 – 7.61 (m, 2H, ArH), 7.51 – 7.45 (m, 3H, ArH), 7.44 – 7.28 (m, 10H, ArH), 7.28 – 7.22 (m, 1H, ArH), 6.71 (d, *J* = 8.4 Hz, 1H, NH), 4.82 – 4.76 (m, 2H, Ar*CH$_2$*, H$_{1L}$), 4.75 – 4.69 (m, 3H, Ar*CH$_2$*), 4.62 (d, *J* = 7.9 Hz, 1H, H$_{1D}$), 4.27 (dt, *J* = 11.2, 8.1 Hz, 1H, H$_{2D}$), 3.90 – 3.83 (m, 1H, H$_{5L}$), 3.78 (t, *J* = 1.8 Hz, 2H, H$_{2L}$, H$_{4L}$), 3.71 (dd, *J* = 11.1, 2.9 Hz, 1H, H$_{3D}$), 3.65 (t, *J* = 1.8 Hz, 1H, H$_{3L}$), 3.47 (d, *J* = 2.8 Hz, 1H, H$_{4D}$), 3.27 – 3.18 (m, 1H, H$_{5D}$), 1.11 (d, *J* = 6.6 Hz, 3H, H6), 1.07 (d, *J* = 6.8 Hz, 12H, H6, *t*Bu). **^{13}C NMR (126 MHz, CDCl$_3$)** δ 158.06 (C=O), 157.77 (C=O), 157.48 (C=O), 157.19 (C=O), 138.72, 136.19, 135.93, 134.67, 133.35, 133.32, 133.24, 133.14, 129.90, 129.71, 128.66, 128.40, 128.09, 127.83, 127.64, 127.61, 127.32, 127.22, 127.00, 126.44, 126.35, 125.77, 119.51 (CF$_3$), 117.22 (CF$_3$), 114.92 (CF$_3$), 112.62 (CF$_3$), 99.43 (C$_{1L}$), 95.57 (C$_{1D}$), 80.13 (C$_{4D}$), 78.36 (C$_{3D}$), 77.79 (C$_{4L}$), 74.95 (Ar*C*H$_2$), 72.16 (Ar*C*H$_2$), 70.70 (C$_{5D}$), 68.53 (C$_{3L}$), 66.87 (C$_{5L}$), 60.10 (C$_{2L}$), 55.20 (C$_{2D}$), 26.91 (*t*Bu), 19.24 (*t*BuC), 16.83 (C6), 16.25 (C6). **ESI HRMS:** [M + Na]$^+$ calculated for C$_{48}$H$_{53}$F$_3$N$_4$O$_8$SiNa, 921.3482; found 921.3486.

***Tert*-butyldiphenylsilyl (Benzyl (2-azido-3,4-di-*O*-benzyl-2-deoxy-β-D-mannopyranosiduronsyl)-(1→4)-2-azido-2-deoxy-3-*O*-(2-naphthylmethyl)-α-L-fucopyranosyl-(1→3)-4-*O*-benzyl-2-deoxy-2-*N*-trichloroacetamide-β-D-fucopyranoside (7)**

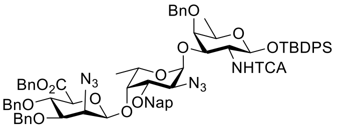

Acceptor **17** (500 mg, 0.527 mmol, 1 equiv.) and thio-donor **11** (460 mg, 0.791 mmol, 1.5 equiv.) were co-evaporated with toluene (x3) before being dissolved in dry DCM (3.5 mL, 0.15 M). Activated 3Å molecular sieves were added and the solution was stirred for 30 min under argon at rt. The reaction mixture was cooled to -78 °C, followed by addition of NIS (237 mg, 1.054 mmol, 2 equiv.) and TBSOTf (24 µL, 0.105 mmol, 0.2 equiv.). The reaction was stirred for 4 h and allowed to warm to -20 °C. When TLC (pentane/EtOAc, 8:2) showed full conversion of the acceptor, the reaction mixture was quenched with NEt_3 and diluted in EtOAc. The organic phase was washed with $Na_2S_2O_3$ (sat. aq.; x1), $NaHCO_3$ (sat. aq.; x1) and brine (x1), dried over Na_2SO_4, filtrated and concentrated *in vacuo*. Column chromatography (pentane/EtOAc, 90:10 → 75:25) furnished trisaccharide **7** in 91% yield (α: 145.5 mg, 0.102 mmol; β: 537 g, 0.377 mmol) in a α/β = 21:79**.** **^{1}H NMR (500 MHz, $CDCl_3$)** δ 7.82 – 7.74 (m, 3H, Ar-*H*), 7.75 – 7.68 (m, 1H, Ar-*H*), 7.67 – 7.57 (m, 3H, Ar-*H*), 7.54 – 7.49 (m, 1H, Ar-*H*), 7.45 – 7.42 (m, 2H, Ar-*H*), 7.38 – 7.23 (m, 22H, Ar-*H*), 7.21 – 7.10 (m, 5H, Ar-*H*), 7.08 – 7.01 (m, 4H, Ar-*H*), 4.91 (d, *J* = 11.3 Hz, 1H, Ar-CH_2), 4.89 (d, *J* = 3.7 Hz, 1H, H-1’), 4.85 (d, *J* = 7.8 Hz, 1H, H-1), 4.78 – 4.62 (m, 7H, Ar-CH_2), 4.61 (d, *J* = 1.1 Hz, 1H, H-1’’), 4.41 (t, *J* = 10.8 Hz, 2H, Ar-CH_2), 4.15 – 4.08 (m, 1H, H-2), 4.06 (t, *J* = 9.5 Hz, 1H, H-4’’), 4.03 – 4.00 (m, 2H, H-2’’, H-4’), 3.96 (dd, *J* = 10.5, 3.6 Hz, 2H, H-2’, H-3), 3.90 (q, *J* = 6.6 Hz, 1H, H-5’), 3.79 (dd, *J* = 10.5, 2.9 Hz, 1H, H-3’), 3.74 (d, *J* = 9.7 Hz, 1H, H-5’’), 3.53 (dd, *J* = 9.2, 3.6 Hz, 1H, H-3’’), 3.44 (d, *J* = 2.2 Hz, 1H, H-4), 3.24 (q, *J* = 6.8 Hz, 1H, H-5), 1.09 (d, *J* = 6.6 Hz, 3H, H-6’), 1.06 (s, 9H, (CH_3)$_3$)), 1.01 (d, *J* = 6.4 Hz, 3H, H-6). **^{13}C NMR (126 MHz, $CDCl_3$)** δ 167.19 (*C*=O), 161.87 (*C*=O), 138.70 (Ar-C_q), 137.91 (Ar-C_q), 137.32 (Ar-C_q), 136.14 (Ar-*C*), 135.92 (*C*H_{Ar}), 135.47 (Ar-C_q), 134.84 (Ar-C_q), 133.66 (Ar-C_q), 133.43 (Ar-C_q), 133.35 (Ar-C_q), 133.02 (Ar-*C*), 132.33 (Ar-*C*), 130.01 (Ar-*C*), 129.64 (Ar-*C*), 129.53 (Ar-*C*), 129.45 (Ar-*C*), 128.74 (Ar-*C*), 128.70 (Ar-*C*), 128.50 (Ar-*C*), 128.40 (Ar-*C*), 128.33 (Ar-*C*), 128.23 (Ar-*C*), 128.14 (Ar-*C*), 128.08 (Ar-*C*), 127.93 (Ar-*C*), 127.90 (Ar-*C*), 127.77 (Ar-*C*), 127.75 (Ar-*C*), 127.60 (Ar-*C*), 127.47 (Ar-*C*), 127.23 (Ar-*C*), 126.45 (Ar-*C*), 126.02 (Ar-*C*), 125.98 (Ar-*C*), 125.82 (Ar-*C*), 100.97 (C-1’’), 98.91 (C-1’), 95.19 (C-1), 79.76 (C-3’’), 78.89 (C-3), 78.58 (C-4), 75.69 (C-3’), 75.34 (C-5’’), 75.25 (Ar-CH_2), 75.13 (C-4’’), 74.91 (Ar-CH_2), 74.89 (C-4’), 72.25 (Ar-CH_2), 70.76 (Ar-CH_2), 70.52 (C-5), 67.34 (Ar-CH_2), 67.11 (C-5’), 61.33 (C-2’’), 59.50 (C-2’), 57.07 (C-2), 27.07 ((*C*H_3)$_3$), 19.30 (*C*(CH_3)$_3$), 17.08 (C-6’), 16.72 (C-6). **ESI HRMS**: $[M+Na]^+$ calculated for $C_{75}H_{78}Cl_3N_7O_{13}SiNa$: 1440.43901; found 1440.43847

***Tert*-butyldiphenylsilyl (Benzyl (2-azido-3,4-di-*O*-benzyl-2-deoxy-β-D-mannopyranosiduronsyl)-(1→4)-2-azido-2-deoxy-3-*O*-(2-naphthylmethyl)-α-L-fucopyranosyl-(1→3)-4-*O*-benzyl-2-deoxy-2-*N*-trichloroacetamide-β-D-fucopyranoside (9)**

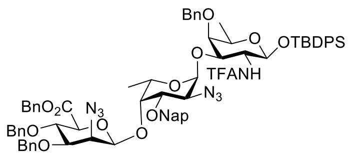

The thioglycoside donor **11** (209 mg, 0.36 mmol, 2.0 eq) and acceptor **18** (170 mg, 0.18 mmol, 1.0 eq) were co-evaporated with toluene for three times. The residue was dissolved in dry DCM (1.8 mL 0.1 M). The reaction mixture was cooled to -78 °C and followed by addition of NIS (162 mg, 0.72 mmol, 4.0 eq) and TBSOTf (9.5 mg, 0.036 mmol, 0.2 eq). The reaction was allowed to stir for 6 h from -78 °C to -10 °C until TLC analysis indicated the complete conversion of starting material. The reaction was quenched with Et_3N, diluted in DCM, washed with sat. $Na_2S_2O_3$, brine, and the organic phase was dried over Na_2SO_4, concentrated *in vacuo*. Purification by column chromatography (silica gel, petroleums ether/ EtOAc, 4/1) give the product **9** (225 mg, 88% yield). **^{1}H NMR (500 MHz, $CDCl_3$)** δ 7.81 – 7.74 (m, 4H, ArH), 7.71 (dd, *J* = 8.1, 1.4 Hz, 2H, ArH), 7.63 (dd, *J* = 8.0, 1.5 Hz, 2H, ArH), 7.53 – 7.49 (m, 1H, ArH), 7.47 – 7.41 (m, 2H, ArH), 7.39 – 7.21 (m, 19H, ArH), 7.19 – 7.10 (m, 4H, ArH, NH), 7.08 – 6.98 (m, 4H, ArH), 4.91 (d, *J* = 11.2 Hz, 1H, Ar*C*H_2), 4.88 (d, *J* = 3.7 Hz, 1H, H_{1L}), 4.86 (d, *J* = 7.8 Hz, 1H, H_{1D}), 4.78 – 4.62 (m, 7H, Ar*C*H_2), 4.60 (d, *J* = 1.1 Hz, 1H, H_{1M}), 4.41 (dd, *J* = 10.9, 8.7 Hz, 2H, Ar*C*H_2), 4.13 – 4.03 (m, 2H, H_{2D}, H_{4M}), 4.01 (dd, *J* = 3.4, 1.3 Hz, 2H, H_{2M}, H_{4L}), 3.96 (ddd, *J* = 10.5, 7.7, 3.2 Hz, 2H, H_{3D}, H_{2L}), 3.89

(d, *J* = 6.7 Hz, 1H, H_{5D}), 3.78 (dd, *J* = 10.5, 2.9 Hz, 1H, H_{3L}), 3.73 (d, *J* = 9.7 Hz, 1H, H_{5M}), 3.55 – 3.49 (m, 1H, H_{3M}), 3.45 – 3.42 (m, 1H, H_{4D}), 3.28 – 3.22 (m, 1H, H_{5L}), 1.08 (d, *J* = 6.6 Hz, 3H, H_{6D}), 1.05 (s, 9H, *t*Bu), 1.01 (d, *J* = 6.4 Hz, 3H, H_{6L}). **^{13}C NMR (126 MHz, CDCl$_3$)** δ 167.23 (C=O), 161.93 (C=O), 138.77, 137.99, 137.39, 136.21, 135.98, 135.53, 134.92, 133.74, 133.51, 133.42, 133.10, 129.68, 129.57, 128.80, 128.76, 128.56, 128.45, 128.39, 128.29, 128.20, 128.13, 128.00, 127.96, 127.81, 127.65, 127.52, 127.28, 126.52, 126.07, 126.04, 125.87, 101.04 (C_{1M}), 98.99 (C_{1L}), 95.21 (C_{1D}), 92.85 (CCl_3), 79.84 (C_{3M}), 78.93 (C_{3D}), 78.69 (C_{4D}), 75.75 (C_{3L}), 75.43 (C_{5M}), 75.32 (Ar*C*H$_2$), 75.20 (C_{4M}), 74.98 (Ar*C*H$_2$), 74.95 (C_{4L}), 72.33 (Ar*C*H$_2$), 70.84 (Ar*C*H$_2$), 70.59 (C_{5L}), 67.41 (Ar*C*H$_2$), 67.16 (C_{5D}), 61.41 (C_{2M}), 59.56 (C_{2L}), 57.19 (C_{2D}), 27.13 (*t*Bu), 19.36 (*t*BuC), 17.14 (C_{6L}), 16.77 (C_{6D}). **ESI HRMS:** $[M + Na]^+$ calculated for $C_{75}H_{78}Cl_3N_7O_{13}SiNa$, 1440.4390; found 1440.4396

(Benzyl (2-azido-3,4-di-*O*-benzyl-2-deoxy-β-D-mannopyranosiduronsyl)-(1→4)-2-azido-2-deoxy 3-*O*-(2-naphthylmethyl)-α-L-fucopyranosyl-(1→3)-4-*O*-benzyl-2-deoxy-2-*N*-trichloroacetamide-α-D-fucopyranose (19)

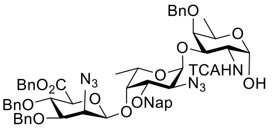

Trisaccharide **7** (529 mg, 0.372 mmol) was dissolved in THF (3.7 mL, 0.1 M) and cooled to 0 °C. AcOH (0.03 mL, 0.558 mmol, 1.5 equiv.) and TBAF (1 M in THF; 0.6 mL, 0.558 mmol, 1.5 equiv.) were added. The reaction mixture was stirred overnight at rt under nitrogen until TLC (pentane/EtOAc, 6:4) showed full conversion. The reaction was quenched by the addition of NH_4Cl (sat. aq.) and diluted in EtOAc. The organic phase was washed with water (x3) and brine (x1), dried over Na_2SO_4, filtrated and concentrated *in vacuo*. Column chromatography (pentane/EtOAc, 80:20 → 60:40) furnished hemiacetal **19** in 86% yield (377 mg, 0.319 mmol). **^{1}H NMR (500 MHz, CDCl$_3$)** δ 7.86 – 7.76 (m, 5H, Ar-*H*), 7.69 (d, *J* = 6.4 Hz, 1H, Ar-*H*), 7.55 – 7.42 (m, 3H, Ar-*H*), 7.41 – 7.21 (m, 17H, Ar-*H*), 7.21 – 7.10 (m, 3H, Ar-*H*), 7.09 – 7.03 (m, 2H, Ar-*H*), 7.03 – 6.96 (m, 2H, Ar-*H*), 5.59 (t, *J* = 3.5 Hz, 1H, H-1), 4.99 (d, *J* = 3.6 Hz, 1H, H-1'), 4.97 (d, *J* = 10.9 Hz, 1H, Ar-C*H$_2$*), 4.85 (d, *J* = 11.7 Hz, 1H, Ar-C*H$_2$*), 4.75 – 4.65 (m, 6H, Ar-C*H$_2$*), 4.64 (s, 1H, H-1''), 4.61 (d, *J* = 12.2 Hz, 1H, Ar-C*H$_2$*), 4.47 – 4.39 (m, 3H, *H*-2, ArC*H$_2$*), 4.20 – 4.01 (m, 7H, H-2', H-4', H-5, H-3, H-4'', H-5', H-2''), 3.89 (dd, *J* = 10.5, 2.9 Hz, 1H, H-3'), 3.73 (d, *J* = 9.8 Hz, 1H, H-5''), 3.69 (d, *J* = 1.5 Hz, 1H, H-4), 3.53 (dd, *J* = 9.2, 3.6 Hz, 1H, H-3''), 2.81 (dd, *J* = 3.7, 1.4 Hz, 1H, O*H*), 1.16 (d, *J* = 6.5 Hz, 3H, H-6), 1.11 (d, *J* = 6.5 Hz, 3H, H-6'). **^{13}C NMR (126 MHz, CDCl$_3$)** δ 167.21 (*C*=O), 162.35 (*C*=O), 138.37 (Ar-*C_q*), 137.97 (Ar-*C_q*), 137.41 (Ar-*C_q*), 135.36 (Ar-*C_q*), 134.90 (Ar-*C_q*), 133.43 (Ar-*C_q*), 133.12 (Ar-*C_q*), 128.80 (Ar-*C*), 128.77 (Ar-*C*), 128.55 (Ar-*C*), 128.53 (Ar-*C*), 128.40 (Ar-*C*), 128.30 (Ar-*C*), 128.23 (Ar-*C*), 128.17 (Ar-*C*), 128.01 (Ar-*C*), 127.96 (Ar-*C*), 127.85 (Ar-*C*), 127.83 (Ar-*C*), 127.76 (Ar-*C*), 126.52 (Ar-*C*), 126.10 (Ar-*C*), 125.99 (Ar-*C*), 125.91 (Ar-*C*), 101.07 (C-1''), 98.81 (C-1'), 90.82 (C-1), 79.79 (C-3''), 77.99 (C-4'/C-5/C-3/C-4''/C-5'), 77.41 (C-4), 76.91 (C-3'), 75.41 (C-5''), 75.33 (Ar-C*H$_2$*), 75.22 (C-4'/C-5/C-3/C-4''/C-5'), 74.98 (C-4'/C-5/C-3/C-4''/C-5'), 74.87 (Ar-C*H$_2$*), 72.41 (Ar-C*H$_2$*), 71.13 (Ar-C*H$_2$*), 67.83 (C-4'/C-5/C-3/C-4''/C-5'), 67.41 (Ar-C*H$_2$*), 66.75 (C-4'/C-5/C-3/C-4''/C-5'), 61.59 (C-2''), 60.28 (C-2'), 51.85 (C-2), 17.16 (C-6'), 16.99 (C-6). **ESI HRMS**: $[M+Na]^+$ calculated for $C_{59}H_{60}Cl_3N_7O_{13}Na$: 1202.32124; found 1202.32069

(Benzyl (2-azido-3,4-di-*O*-benzyl-2-deoxy-β-D-mannopyranosiduronsyl)-(1→4)-2-azido-2-deoxy 3-*O*-(2-naphthylmethyl)-α-L-fucopyranosyl-(1→3)-4-*O*-benzyl-2-deoxy-2-*N*-trichloroacetamide-1-*O*-(*N*-phenyl-2,2,2-trifluoroacetimidoyl)-α-D-fucopyranose (20)

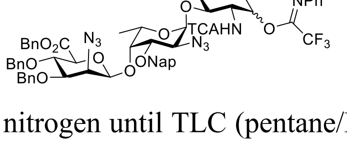

Hemiacetal **19** (352 mg, 0.298 mmol,) was co-evaporated with toluene (x3) before being dissolved in dry acetone (1.5 mL, 0.2 M). K_2CO_3 (82 mg, 0.596 mmol, 2 equiv.) was added to the solution followed by $CF_3C(NPh)Cl$ (0.1 mL, 0.596 mmol, 2 equiv.). The reaction mixture was stirred overnight under nitrogen until TLC (pentane/EtOAc, 7:3) showed full conversion. The mixture was filtered and concentrated *in vacuo*. Column chromatography (pentane/EtOAc 9:1 → 7:3) yielded imidate donor **20** in 96% yield (387 mg, 0.286 mmol). **^{1}H NMR (500 MHz, CD$_3$CN)** δ 7.90 – 7.72 (m, 7H), 7.53 – 7.39 (m, 8H), 7.39 – 7.24 (m, 19H), 7.24 – 7.21 (m, 1H), 7.20 – 7.04 (m, 11H), 6.81 (s, 2H), 5.23 (s, 1H), 4.96 – 4.87 (m, 2H), 4.87 – 4.69

(m, 8H), 4.69 – 4.61 (m, 3H), 4.46 – 4.33 (m, 5H), 4.30 – 4.20 (m, 3H), 4.10 – 4.03 (m, 2H), 4.00 (q, *J* = 6.9 Hz, 1H), 3.94 – 3.86 (m, 3H), 3.86 – 3.82 (m, 3H), 3.81 – 3.73 (m, 3H), 1.25 – 1.21 (m, 6H). **^{13}C NMR (126 MHz, CD_3CN)** δ 168.73, 163.36, 138.68, 136.89, 134.40, 133.53, 129.89, 129.41, 129.36, 129.18, 129.03, 128.85, 128.82, 128.71, 128.60, 127.15, 126.87, 118.32, 101.79, 99.54, 80.61, 79.00, 76.69, 76.42, 75.80, 75.67, 72.34, 71.00, 70.79, 68.44, 67.88, 62.29, 60.95, 52.15, 29.71, 17.11, 16.90. **ESI HRMS**: $[M+Na]^+$ calculated for $C_{67}H_{64}Cl_3F_3N_8O_{13}Na$: 1375.34787; found 1375.34717

5-(Benzyl(benzyloxycarbonyl)amino)pentyl (Benzyl (2-azido-3,4-di-*O*-benzyl-2-deoxy-β-D-mannopyranosiduronsyl)-(1→4)-2-azido-2-deoxy-3-*O*-(2-naphthylmethyl)-α-L-fucopyranosyl-(1→3)-4-*O*-benzyl-2-deoxy-2-*N*-trichloroacetamide-α-D-fucopyranoside (22)

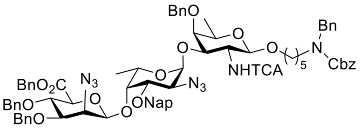

Donor **20** (169 mg, 0.125 mmol, 1 equiv.) and acceptor **21** (53 mg, 0.163 mmol, 1.3 equiv.) were co-evaporated with toluene (x3) before being dissolved in DCM/MeCN (1:1, 1.3 mL, 0.1 M). Activated 3Å molecular sieves were added and the solution was stirred for 30 min under argon at rt. The reaction mixture was cooled to -50 °C, followed by addition of TBSOTf (6 µL, 0.025 mmol, 0.2 equiv.). The mixture was stirred for 1 h while warming to -40 °C until TLC (pentane/EtOAc, 7:3) showed full conversion of the donor. The reaction was quenched with NEt_3 and diluted in EtOAc. The organic phase was washed with water (x1) and brine (x1), dried over Na_2SO_4, filtered and concentrated *in vacuo*. Column chromatography (pentane/EtOAc, 7:3 → 6:4) and size exclusion chromatography yielded trisaccharide **22** in 73% yield (136 mg, 0.91 mmol) as the sole β anomer. **^{1}H NMR (400 MHz, $CDCl_3$)** δ 7.84 – 7.76 (m, 4H), 7.53 (dd, *J* = 8.5, 1.6 Hz, 1H), 7.46 (dd, *J* = 6.3, 3.3 Hz, 2H), 7.41 – 7.27 (m, 19H), 7.24 – 7.11 (m, 6H), 7.13 – 7.02 (m, 4H), 5.18 (d, *J* = 9.5 Hz, 2H), 4.96 (d, *J* = 3.6 Hz, 1H), 4.90 (d, *J* = 11.4 Hz, 1H), 4.87 – 4.78 (m, 2H), 4.75 (d, *J* = 3.9 Hz, 1H), 4.73 – 4.66 (m, 5H), 4.63 (d, *J* = 1.1 Hz, 1H), 4.52 – 4.40 (m, 4H), 4.35 – 4.26 (m, 1H), 4.10 (t, *J* = 9.4 Hz, 1H), 4.04 (d, *J* = 3.2 Hz, 1H), 3.98 (s, 1H), 3.93 (dd, *J* = 10.6, 3.7 Hz, 1H), 3.92 – 3.77 (m, 4H), 3.77 (d, *J* = 9.7 Hz, 2H), 3.75 – 3.71 (m, 1H), 3.62 (q, *J* = 6.3 Hz, 1H), 3.59 – 3.52 (m, 2H), 3.45 – 3.31 (m, 1H), 3.20 (dt, *J* = 30.7, 7.8 Hz, 2H), 1.60 – 1.42 (m, 4H), 1.33 – 1.25 (m, 5H), 1.14 (d, *J* = 6.5 Hz, 3H). **^{13}C NMR (101 MHz, $CDCl_3$)** δ 167.22, 162.08, 138.51, 137.99, 137.94, 137.34, 135.43, 134.86, 133.35, 133.06, 128.80, 128.73, 128.62, 128.54, 128.39, 128.36, 128.26, 128.15, 128.12, 127.96, 127.94, 127.81, 127.78, 127.71, 127.50, 127.34, 127.25, 100.99, 99.29, 99.22, 79.83, 79.30, 78.00, 77.48, 77.16, 76.84, 75.40, 75.30, 75.15, 74.99, 72.29, 70.84, 70.65, 69.76, 67.40, 67.22, 66.94, 61.34, 59.25, 56.82, 50.57, 50.31, 47.21, 46.24, 29.26, 28.00, 27.48, 17.20, 17.13. **ESI HRMS**: $[M+Na]^+$ calculated for $C_{79}H_{83}Cl_3N_8O_{15}Na$: 1511.49412; found 1511.49357

5-(Benzyl(benzyloxycarbonyl)amino)pentyl (Benzyl (2-azido-3,4-di-*O*-benzyl-2-deoxy-β-D-mannopyranosiduronsyl)-(1→4)-3-*O*-acetyl-2-azido-2-deoxy-α-L-fucopyranosyl-(1→3)- 4-*O*-benzyl-2-deoxy-2-*N*-trichloroacetamide-α-D-fucopyranoside (23)

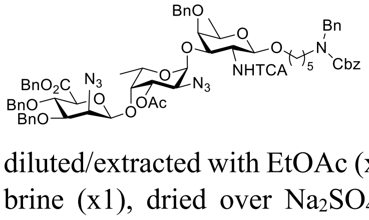

22 (138 mg, 0.0926 mmol) was dissolved in DCM/H_2O (4:1, 1.85 mL, 0.05 M) and added DDQ (42 mg, 0.185 mmol, 2 equiv.). The reaction was stirred at rt under nitrogen for 6 h until TLC (pentane, EtOAc, 6:4) showed full conversion. The solution was quenched with $Na_2S_2O_3$ (sat. aq.) and diluted/extracted with EtOAc (x3). The combined organic phases were washed with $NaHCO_3$ (sat. aq.; x4) and brine (x1), dried over Na_2SO_4, filtrated and concentrated *in vacuo*. The crude was used without further purification. The residue was dissolved in pyridine (2 mL) and cooled to 0 °C and added Ac_2O (0.3 mL) and DMAP (catalytic amount) and stirred at rt under nitrogen overnight until TLC (pentane/acetone, 7:3) showed full conversion. The mixture was dissolved in EtOAc, washed with 1 M HCl (x1), $NaHCO_3$ (sat. aq.; x1) and brin (x1), dried over Na_2SO_4 and concentrated *in vacuo*. Column chromatography (pentane/EtOAc, 7:3 → 6:4) yielded trisaccharide **23** in 92% yield (119 mg, 0.0856 mmol).**^{1}H NMR (400 MHz, $CDCl_3$)** δ 7.40 – 7.27 (m, 27H), 7.26 – 7.07 (m, 9H), 5.24 – 5.10 (m, 5H), 5.03 – 4.94 (m, 3H), 4.91 – 4.80 (m, 1H), 4.80 – 4.75 (m, 3H), 4.75 – 4.62 (m, 3H), 4.50 – 4.39 (m, 5H), 4.39 – 4.31 (m, 1H), 4.08 – 4.00 (m, 3H), 4.00 (dd, *J* = 3.7, 1.0 Hz, 2H), 3.98 – 3.88 (m, 2H), 3.82 (q, *J* = 11.3, 9.6 Hz, 3H), 3.72 (dd, *J* = 9.8, 6.0 Hz, 1H), 3.63 (q, *J* = 6.4 Hz,

1H), 3.58 – 3.49 (m, 2H), 3.46 – 3.31 (m, 1H), 3.28 – 3.04 (m, 2H), 2.04 (s, 4H), 1.62 – 1.40 (m, 5H), 1.33 – 1.28 (m, 5H), 1.26 (d, *J* = 1.8 Hz, 4H), 1.05 (d, *J* = 6.5 Hz, 3H). **^{13}C NMR (101 MHz, $CDCl_3$)** δ 170.62, 167.58, 138.22, 137.99, 137.82, 137.29, 134.85, 128.87, 128.74, 128.62, 128.52, 128.42, 128.29, 127.96, 127.94, 127.90, 127.82, 127.66, 127.33, 101.05, 99.79, 99.01, 79.54, 78.59, 78.07, 75.65, 75.50, 75.30, 75.12, 72.30, 70.73, 70.28, 69.75, 69.61, 67.71, 67.23, 66.28, 61.06, 57.66, 55.97, 53.88, 31.03, 29.37, 29.25, 23.51, 23.15, 20.86, 17.29, 16.52. **ESI HRMS**: $[M+NH_4]^+$ calculated for $C_{70}H_{77}Cl_3N_8O_{16}NH_4$:1408.48669; found 1408.48614

5-aminopentyl 2-*N*-acetamide-2-deoxy-β-D-mannopyranosiduronsyl-(1→4)-2-*N*-acetamide-3-*O*-acetyl-2-deoxy-α-L-fucopyranosyl-(1→3)-2-*N*-acetamide-2-deoxy-β-D-fucopyranoside (1)

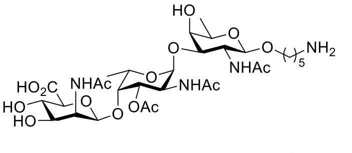

23 (106 mg, 0.0762 mmol) was dissolved in THF (distilled, 3 mL) and added zinc powder (mg, mmol, 300 equiv.), AcOH (1 mL) and Ac_2O (0.5 mL). The resulting mixture was stirred at 50 °C overnight until TLC (DCM/MeOH, 95:5) showed full conversion. The cooled mixture was filtered through Celite, evaporated *in vacuo* and co-evaporated with toluene (x3). The crude product was first purified by column chromatography (DCM/MeOH, 98:2 → 90:10) followed by HPLC given **24** in 18% yield (18 mg, 0.0136 mmol). The product **24** (13 mg, 0.00976 mmol) was dissolved in *t*-BuOH (1.5 mL) and added AcOH (1 mL, 0.1 mL in 100 mL MilliQ). Another 1 mL *t*-BuOH was added to dissolve the compound. The solution was birched with argon for 20 min and then $Pd(OH)_2$/C (catalytic amount) was added. The reaction was again birched with argon for 5 minutes before the atmosphere was changed for H_2. The mixture was stirred for 3 days under H_2 atmosphere, after which it was filtered over a Whatman filter and lyophilized. Purification by a HW40 column with NH_4OAc followed by lyophilization gave **1** in 56% yield (4 mg, 0.0054 mmol). **^{1}H NMR (600 MHz, D2O)** δ 5.02 (dd, *J* = 11.6, 3.0 Hz, 1H, H'-3), 5.00 (d, *J* = 3.9 Hz, 1H, H'-1), 4.74 (d, *J* = 1.4 Hz, 1H, H''-1), 4.59 (dd, *J* = 4.3, 1.4 Hz, 1H, H-1), 4.40 (d, *J* = 8.6 Hz, 1H, H'-2), 4.37 (dd, *J* = 11.6, 3.9 Hz, 1H, H'-4), 4.21 (d, *J* = 3.1 Hz, 1H, H'-5), 4.18 (q, *J* = 6.4 Hz, 1H, H-2), 3.98 (dd, *J* = 10.3, 8.6 Hz, 1H, C*H*2-Linker), 3.88 (dt, *J* = 10.1, 6.0 Hz, 1H), 3.82 – 3.74 (m, 4H, H''-3, H-3, H'-4, H-5), 3.64 (t, *J* = 9.7 Hz, 1H, H''-4), 3.61 – 3.54 (m, 2H, H''-5, C*H*2-Linker), 2.99 (t, *J* = 7.7 Hz, 2H, C*H*2-Linker), 2.13 (s, 3H, COC*H*3), 2.08 (s, 3H, COC*H*3), 2.00 (s, 3H, COC*H*3), 1.97 (s, 3H, COC*H*3), 1.67 (p, *J* = 7.7 Hz, 2H, C*H*2-Linker), 1.63 – 1.55 (m, 2H, C*H*2-Linker), 1.39 (pd, *J* = 7.1, 2.0 Hz, 2H, C*H*2-Linker), 1.27 (d, *J* = 6.4 Hz, 3H, H-6, H'-6), 1.24 (d, *J* = 6.5 Hz, 3H, H-6/ H'-6). **^{13}C NMR (151 MHz, D2O)** δ 176.53 (C=O), 176.15 (C=O), 175.18 (C=O), 175.02 (C=O), 174.73 (C=O), 102.44, 100.75, 99.94, 79.26, 78.03, 76.95, 72.54, 71.62, 71.35, 70.98, 70.74, 70.34, 67.74, 53.93, 52.23, 48.05, 40.24, 29.08, 27.32, 23.10, 23.06, 22.95, 22.89, 21.24, 16.27 (C-6/ C'-6), 16.18 (C-6/ C'-6). **ESI HRMS**: $[M+H]^+$ calculated for $C_{31}H_{52}N_4O_{16}H$: 737.34566; found 737.34497

***Tert*-butyldiphenylsilyl (Benzyl (2-azido-2-deoxy-3-*O*-benzyl-4-*O*-*p*-methoxybenzyl-β-D-mannopyranosiduronsyl)-(1→4)-2-azido-2-deoxy-3-*O*-(2-naphthylmethyl)-α-L-fucopyranosyl-(1→3)-4-*O*-benzyl-2-deoxy-2-*N*-trichloroacetamide-β-D-fucopyranoside (6)**

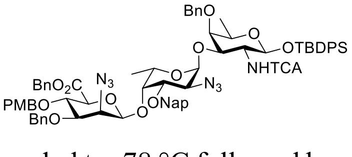

Acceptor **17** (417 mg, 0.44 mmol, 1 equiv.) and donor **10** (404 mg, 0.66 mmol, 1.5 equiv.) were co-evaporated trice with toluene before being dissolved in dry DCM 3 (mL, 0.15 M). Activated 3Å molecular sieves were added and the solution was stirred for 30 min under argon at rt. The reaction mixture was cooled to -78 °C followed by addition of NIS (198 mg, 0.88 mmol, 2 equiv.) and TBSOTf (20 µL, 0.088 mmol, 0.2 equiv.). The reaction mixture was allowed to warm to -10 °C and stirred for 4 h under argon until TLC (toluene/EtOAc, 8:2) showed full conversion. The reaction was quenched with NEt_3 and diluted in EtOAc. The organic phase was washed with $Na_2S_2O_3$ (sat. aq.; x1), $NaHCO_3$ (sat. aq.; x1) and brine (x1), dried over Na_2SO_4, filtrated and concentrated *in vacuo*. Column chromatography (pentane/EtOAc, 90:10 → 75:25) yielded 82% of trisaccharide **6** (α: 155 mg, 0.107 mmol; β: 367 mg, 0.253 mmol) in a α/β = 30:70. NMR reported for the β-anomer. **^{1}H NMR (500 MHz, $CDCl_3$)** δ 7.82 – 7.75 (m, 3H, Ar-*H*), 7.73 – 7.68 (m, 2H, Ar-*H*), 7.65 – 7.61 (m, 2H, Ar-*H*), 7.50 (dd, *J* = 8.5, 1.6 Hz, 1H, Ar-*H*), 7.47 – 7.43 (m, 2H, Ar-*H*), 7.38 – 7.26 (m, 17H, Ar-*H*), 7.19 – 7.13 (m, 4H, Ar-*H*, *H*N(CO)CCl_3), 7.07 – 7.03 (m, 2H, Ar-*H*), 6.99 – 6.94 (m, 1H, Ar-

H), 6.78 – 6.73 (m, 2H, Ar-*H*), 4.92 – 4.84 (m, 3H, H-1', H-1, Ar-CH_2), 4.77 (d, *J* = 12.0 Hz, 1H, Ar-CH_2), 4.73 – 4.63 (m, 6H, Ar-CH_2), 4.59 (d, *J* = 1.1 Hz, 1H, *H*-1''), 4.41 (d, *J* = 11.2 Hz, 1H, Ar-CH_2), 4.34 (d, *J* = 10.2 Hz, 1H, Ar-CH_2), 4.13 – 4.07 (m, 1H, H-2), 4.04 (t, *J* = 9.5 Hz, 1H, H-4''), 4.01 – 3.96 (m, 3H, H-2'', H-4', H-3), 3.94 (dd, *J* = 10.6, 3.6 Hz, 1H, H-2'), 3.89 (q, *J* = 6.6 Hz, 1H, H-5'), 3.80 – 3.74 (m, 4H, H-3', OCH_3), 3.71 (d, *J* = 9.8 Hz, 1H, H-5''), 3.50 (dd, *J* = 9.2, 3.6 Hz, 1H, H-3''), 3.44 (d, *J* = 2.9 Hz, 1H, H-4), 1.08 (d, *J* = 6.7 Hz, 3H, H-6'), 1.05 (s, 9H, $(CH_3)_3$), 1.00 (d, *J* = 6.4 Hz, 3H, *H*-6). **^{13}C NMR (126 MHz, $CDCl_3$)** δ 167.26 (*C*=O), 161.93 (*C*=O), 138.80 (Ar-C_q), 137.47 (Ar-C_q), 136.21 (Ar-*C*), 135.98 (Ar-*C*), 135.54 (Ar-C_q), 134.98 (Ar-C_q), 133.77 (Ar-C_q), 133.53 (Ar-C_q), 133.44 (Ar-C_q), 133.11 (Ar-C_q), 130.17 (Ar-C_q), 129.72 (Ar-*C*), 129.69 (Ar-*C*), 129.57 (Ar-*C*), 128.82 (Ar-*C*), 128.77 (Ar-*C*), 128.58 (Ar-*C*), 128.46 (Ar-*C*), 128.28 (Ar-*C*), 128.21 (Ar-*C*), 128.14 (Ar-*C*), 127.98 (Ar-*C*), 127.82 (Ar-*C*), 127.65 (Ar-*C*), 127.52 (Ar-*C*), 127.28 (Ar-*C*), 126.53 (Ar-*C*), 126.06 (Ar-*C*), 126.05 (Ar-*C*), 113.82 (Ar-*C*), 101.06 (*C*-1''), 99.00 (C-1'), 95.22 (C-1), 79.89 (C-3''), 78.93 (C-3), 78.71 (C-4), 75.74 (C-3'), 75.49 (C-4'), 75.04 (Ar-CH_2), 74.98 (Ar-CH_2), 74.95 (C-4''), 74.95 (C-5''), 72.37 (Ar-CH_2), 70.84 (Ar-CH_2), 70.60 (C-5), 67.39 (Ar-CH_2), 67.17 (C-5'), 61.46 (C-2''), 59.58 (C-2'), 57.21 (C-2), 55.39 (OCH_3), 27.14 ($(CH_3)_3$), 19.36 ($C(CH_3)_3$), 17.15 (C-6'), 16.78 (C-6). **ESI HRMS**: $[M+Na]^+$ calculated for $C_{76}H_{80}Cl_3N_7O_{14}Na$:1470.44958; found 1470.44903

***Tert*-butyldiphenylsilyl (Benzyl (2-azido-2-deoxy-3-*O*-benzyl-4-*O*-*p*-methoxybenzyl-β-D-mannopyranosiduronsyl)-(1→4)-2-azido-2-deoxy-3-*O*-(2-naphthylmethyl)-α-L-fucopyranosyl-(1→3)-4-*O*-benzyl-2-deoxy-2-*N*-triflouroacetamide-β-D-fucopyranoside (8)**

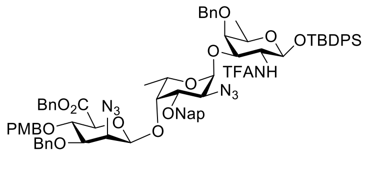

The thioglycoside donor **10** (1.15 g, 1.89 mmol, 2.0 eq) and acceptors **18** (849 mg, 0.944 mmol) were co-evaporated with toluene for three times. The residue was dissolved in dry DCM (9.5 mL, 0.1). The reaction mixture was cooled to -78 °C and followed by addition of NIS (850 mg, 3.78 mmol, 4.0 eq) and TBSOTf (50 mg, 0.19 mmol, 0.2 eq). The reaction was allowed to stir for 6 h from -78 °C to -10 °C until TLC analysis indicated the complete conversion of starting material. The reaction was quenched with Et_3N, diluted in DCM, washed with sat. $Na_2S_2O_3$, brine, and the organic phase was dried over Na_2SO_4, concentrated *in vacuo*. Purification by column chromatography (silica gel, petroleum ether/EtOAc, 4/1) give the product **8** (1.23 g, 93% yield). Characterization for α-anomer: **^{1}H NMR (400 MHz, $CDCl_3$)** δ 7.85 – 7.75 (m, 4H, ArH), 7.71 (dt, *J* = 6.7, 1.5 Hz, 2H, ArH), 7.62 (dt, *J* = 6.7, 1.5 Hz, 2H, ArH), 7.54 – 7.50 (m, 1H, ArH), 7.46 – 7.11 (m, 22H, ArH), 7.09 – 7.03 (m, 2H, ArH), 6.97 (d, *J* = 8.6 Hz, 2H, ArH), 6.75 (d, *J* = 8.7 Hz, 2H, ArH, NH), 4.89 (d, *J* = 11.0 Hz, 1H, ArCH_2), 4.78 (d, *J* = 3.2 Hz, 1H, H_{1L}), 4.81 – 4.73 (m, 1H, ArCH_2), 4.72 – 4.62 (m, 6H, ArCH_2), 4.59 (d, *J* = 1.2 Hz, 1H, H_{1M}), 4.58 (d, *J* = 3.3 Hz, 1H, H_{1D}), 4.45 (d, *J* = 11.1 Hz, 1H, ArCH_2), 4.34 (d, *J* = 10.1 Hz, 1H, ArCH_2), 4.24 (dt, *J* = 11.0, 8.1 Hz, 1H, H_{2D}), 4.03 – 3.96 (m, 4H, H_{4L}, H_{2L}, H_{2M}, H_{4M}), 3.90 (q, *J* = 6.5 Hz, 1H, H_{5D}), 3.77 – 3.65 (m, 6H, OCH_3, H_{3L}, H_{5M}, H_{3D}), 3.52 – 3.44 (m, 2H, H_{3M}, H_{4D}), 3.20 (q, *J* = 6.4 Hz, 1H, H_{5L}), 1.04 (d, *J* = 6.7 Hz, 15H, H_{6D}, H_{6L}, *t*Bu). **^{13}C NMR (101 MHz, $CDCl_3$)** δ 167.25 (C=O), 159.32 (ArC), 157.89 (CF_3C=O), 157.53 (CF_3C=O), 138.62, 137.43, 136.19, 136.16, 135.92, 135.49, 134.92, 133.86, 133.38, 133.10, 133.06, 130.10, 129.88, 129.68, 129.66, 128.80, 128.77, 128.71, 128.67, 128.59, 128.54, 128.41, 128.22, 128.19, 128.06, 127.92, 127.77, 127.65, 127.61, 127.58, 127.29, 127.28, 127.22, 126.60, 126.10, 126.01, 125.83, 113.75 (ArC-PMB), 100.95 (C_{1M}), 99.17 (C_{1L}), 95.76 (C_{1D}), 80.32 (C_{3D}), 79.81 (C_{3M}), 77.85 (C_{4D}), 76.09 (C_{3L}), 75.36 (C_{5M}), 75.04 (C_{4M}), 74.98 (ArCH_2), 74.93 (C_{4L}), 74.85 (ArCH_2), 72.33 (ArCH_2), 71.13 (ArCH_2), 70.62 (C_{5L}), 67.40 (C_{5D}), 67.36 (ArCH_2), 61.51 (C_{2M}), 59.92 (C_{2L}), 55.34 (OCH_3), 54.99 (C_{2D}), 26.89 (*t*Bu), 19.21 (*t*BuC), 17.03 (C_{6L}), 16.77 (C_{6D}). **ESI HRMS**: $[M + Na]^+$ calculated for $C_{76}H_{80}F_3N_7O_{14}SiNa$, 1422.5382; found 1422.5379. Characterization for α-anomer: **^{1}H NMR (500 MHz, $CDCl_3$)** δ 7.83 – 7.75 (m, 4H, ArH), 7.74 – 7.70 (m, 2H, ArH), 7.66 – 7.60 (m, 2H, ArH), 7.49 (dd, *J* = 8.4, 1.7 Hz, 1H, ArH), 7.46 – 7.06 (m, 25H, ArH), 6.79 (d, *J* = 8.7 Hz, 2H, ArH), 6.65 (d, *J* = 8.2 Hz, 1H, NH), 5.25 (d, *J* = 5.3 Hz, 1H, H_{1L}), 5.05 – 4.92 (m, 2H, ArCH_2), 4.78 (d, *J* = 1.0 Hz, 1H, H_{1M}), 4.77 (d, *J* = 12.0 Hz, 1H, ArCH_2), 4.73 (s, 2H, ArCH_2), 4.66 (d, *J* = 3.3 Hz, 1H, H_{1D}), 4.64 (d, *J* = 11.1 Hz, 1H, ArCH_2), 4.52 (d, *J* = 11.0 Hz, 1H, ArCH_2), 4.45 – 4.35 (m, 4H, ArCH_2, H_{4M}), 4.22 (dt, *J* = 11.2, 8.0 Hz, 1H, H_{2D}), 4.02 (t, *J* = 5.9 Hz, 1H, H_{3M}), 3.91 – 3.84 (m, 2H, H_{5D}, H_{4L}), 3.82 – 3.72

(m, 8H, OCH3, H_{2L}, H_{2M}, H_{3D}, H_{3L}, H_{5M}), 3.46 (d, *J* = 2.8 Hz, 1H, H_{4D}), 3.28 – 3.20 (m, 1H, H_{5L}), 1.11 (d, *J* = 6.5 Hz, 3H, H_{6D}), 1.06 (d, *J* = 3.3 Hz, 12H, H_{6L}, *t*Bu). **^{13}C NMR (126 MHz, CDCl$_3$)** δ 169.00 (C=O), 159.50 (ArC), 157.80 (CF_3C=O), 157.51 (CF_3C=O), 138.72, 137.36, 136.22, 135.95, 135.27, 134.78, 133.40, 133.19, 133.15, 129.92, 129.71, 129.67, 128.67, 128.55, 128.46, 128.44, 128.38, 128.34, 128.17, 128.03, 128.00, 127.80, 127.70, 127.66, 127.36, 127.33, 126.82, 126.17, 126.06, 125.88, 119.50 (CF_3), 117.20 (CF_3), 114.91 (CF_3), 113.94, 112.61 (CF_3), 99.51 (C_{1M}), 99.14 (C_{1L}), 95.57 (C_{1D}), 80.06 (C_{3D}), 78.31 (C_{4D}), 77.71 (C_{3L}), 77.07 (C_{5M}), 76.31 (C_{4L}), 74.97 (Ar*C*H$_2$), 74.74 (C_{3M}), 73.27 (Ar*C*H$_2$), 72.95 (C_{4M}), 72.93 (Ar*C*H$_2$), 72.47 (Ar*C*H$_2$), 70.72 (C_{5L}), 67.33 (C_{5D}), 67.15 (Ar*C*H$_2$), 60.64 (C_{2M}), 60.42 (C_{2L}), 55.38 (OCH_3), 55.38 (C_{2D}), 26.94 (*t*Bu), 19.26 (*t*BuC), 17.04 (C_{6L}), 16.83 (C_{6D}). **ESI HRMS:** $[M + Na]^+$ calculated for $C_{76}H_{80}F_3N_7O_{14}SiNa$, 1422.5382; found 1422.5378.

(Benzyl (2-azido-3-*O*-benzyl-2-deoxy-4-*O*-*p*-methoxybenzyl-β-D-mannopyranosiduronsyl)-(1→4)-2-azido-2-deoxy-3-*O*-(2-naphthylmethyl)-α-L-fucopyranosyl-(1→3)-4-*O*-benzyl-2-deoxy-2-*N*-trichloroacetamide-β-D-fucopyranose (25-OH)

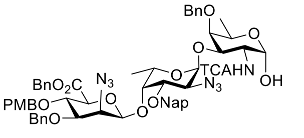

Trisaccharide **6** (297 mg, 0.205 mmol) was dissolved in THF (2 mL, 0.1 M) and cooled to 0 °C. Following, AcOH (24 µL, 0.41 mmol, 2 equiv.) and TBAF (1 M in THF; 0.4 mL, 0.41 mmol, 2 equiv.) were added. The reaction mixture was stirred overnight at rt under nitrogen atmosphere until TLC (pentane/EtOAc, 7:3) showed full conversion. The reaction was quenched by the addition of NH_4Cl (sat. aq.) and diluted in EtOAc. The organic phase was washed with water (x3) and brine (x1), dried over Na_2SO_4, filtrated and concentrated *in vacuo*. Column chromatography (pentane/EtOAc, 80:20 → 60:40) furnished hemiacetal **25-OH** in 83% yield (205 mg, 0.17 mmol). **^{1}H NMR (500 MHz, CDCl$_3$)** δ 7.86 – 7.76 (m, 4H, Ar-*H*), 7.70 (d, *J* = 6.4 Hz, 1H, *H*N(CO)CCl$_3$), 7.51 (dd, *J* = 8.3, 1.7 Hz, 1H, Ar-*H*), 7.48 – 7.42 (m, 2H, Ar-*H*), 7.39 – 7.27 (m, 9H, Ar-*H*), 7.22 – 7.13 (m, 3H, Ar-*H*), 7.04 – 7.00 (m, 2H, Ar-*H*), 6.98 – 6.93 (m, 2H, Ar-*H*), 6.78 – 6.73 (m, 2H, Ar-*H*), 5.60 (t, *J* = 3.6 Hz, 1H, H-1), 5.03 – 4.93 (m, 2H, *H*-1', Ar-C*H$_2$*), 4.85 (d, *J* = 11.7 Hz, 1H, Ar-C*H$_2$*), 4.74 – 4.60 (m, 7H, H-1'', Ar-C*H$_2$*), 4.48 – 4.43 (m, 1H, H-2), 4.41 (d, *J* = 10.9 Hz, 1H, Ar-C*H$_2$*), 4.33 (d, *J* = 10.1 Hz, 1H, Ar-C*H$_2$*), 4.17 – 4.00 (m, 7H, H-2', H-5, H-3, H-4', H-5', H-4'', H-2''), 3.88 (dd, *J* = 10.5, 2.9 Hz, 1H, H-3'), 3.77 (s, 3H, OC*H$_3$*), 3.72 (d, *J* = 9.7 Hz, 1H, H-5''), 3.69 (d, *J* = 2.6 Hz, 1H, H-4), 3.51 (dd, *J* = 9.2, 3.6 Hz, 1H, H-3''), 2.73 (dd, *J* = 3.6, 1.4 Hz, 1H, O*H*), 1.15 (d, *J* = 6.5 Hz, 3H, H-6), 1.10 (d, *J* = 6.6 Hz, 3H, H-6'). **^{13}C NMR (126 MHz, CDCl$_3$)** δ 167.23 (*C*=O), 162.35 (*C*=O), 138.35 (Ar-*C$_q$*), 137.46 (Ar-*C$_q$*), 135.37 (Ar-*C$_q$*), 134.91 (Ar-*C$_q$*), 133.40 (Ar-*C$_q$*), 133.05 (Ar-*C$_q$*), 130.12 (Ar-*C$_q$*), 129.73 (Ar-*C*), 128.82 (Ar-*C*), 128.78 (Ar-*C*), 128.57 (Ar-*C*), 128.53 (Ar-*C*), 128.30 (Ar-*C*), 128.23 (Ar-*C*), 128.17 (Ar-*C*), 127.98 (Ar-*C*), 127.86 (Ar-*C*), 127.83 (Ar-*C*), 127.77 (Ar-*C*), 126.52 (Ar-*C*), 126.09 (Ar-*C*), 125.99 (Ar-*C*), 125.91 (Ar-*C*), 113.81 (Ar-*C*), 101.09 (C-1''), 98.81 (C-1'), 90.82 (C-1), 79.80 (C-3''), 77.98 (C-3/C-4'/C-4''/C-5'), 77.51 (C-4), 76.54 (Ar-*C*H$_2$), 75.44 (C-3'), 75.06 (C-5''), 74.96 (C-3/C-4'/C-4''/C-5'), 74.96 (C-3/C-4'/C-4''/C-5'), 74.86 (Ar-*C*H$_2$), 72.44 (Ar-*C*H$_2$), 71.10 (Ar-*C*H$_2$), 67.83 (Ar-*C*H$_2$), 67.39 (Ar-*C*H$_2$), 66.75 (C-5'), 61.61 (C-2''), 60.28 (C-2'), 55.40 (O*C*H$_3$), 51.84 (C-2), 17.17 (C-6'), 16.99 (C-6). **ESI HRMS**: $[M+Na]^+$ calculated for $C_{60}H_{62}Cl_3N_7O_{14}Na$: 1232.33180; found 1232.33125

(Benzyl (2-azido-3-*O*-benzyl-2-deoxy-4-*O*-*p*-methoxybenzyl-β-D-mannopyranosiduronsyl)-(1→4)-2-azido-2-deoxy-3-*O*-(2-naphthylmethyl)-α-L-fucopyranosyl-(1→3)-4-*O*-benzyl-2-deoxy-2-*N*-tricfluoroacetamide-β-D-fucopyranose (26-OH)

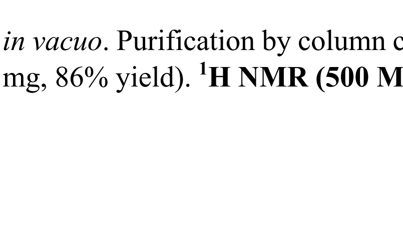

Trisaccharide **8** (1.24 g, 0.886 mmol) was dissolved in THF (8.9 mL, 0.1M), a catalytic amount of AcOH and TBAF (1.33 mL, 1.33 mmol, 1.5 eq) were added at 0 °C. The resulting mixture was stirred at room temperature for 2 h until TLC analysis indicated the complete conversion of starting material. The reaction was diluted in EtOAc and washed with sat. NH_4Cl, brine. The organic phase was dried over Na_2SO_4, concentrated *in vacuo*. Purification by column chromatography (silica gel, petroleum ether/acetone = 3/1) give **26-OH** (884 mg, 86% yield). **^{1}H NMR (500 MHz, CDCl$_3$)** δ 7.85 – 7.72 (m, 4H, ArH), 7.63 (d, *J* = 6.4 Hz, 1H, NH), 7.53

(dd, *J* = 8.4, 1.7 Hz, 1H, ArH), 7.46 – 7.40 (m, 2H, ArH), 7.37 – 7.11 (m, 15H, ArH), 7.09 – 7.04 (m, 2H, ArH), 6.97 (d, *J* = 8.7 Hz, 2H, ArH), 6.76 (d, *J* = 8.7 Hz, 2H, ArH), 5.51 (d, *J* = 3.5 Hz, 1H, H_{1D}), 4.94 (d, *J* = 3.8 Hz, 1H, H_{1L}), 4.90 (d, *J* = 11.1 Hz, 1H, Ar*CH2*), 4.82 – 4.71 (m, 3H, Ar*CH2*), 4.70 – 4.61 (m, 4H, Ar*CH2*), 4.63 (d, *J* = 1.1 Hz, 1H, H_{1M}), 4.52 (d, *J* = 11.2 Hz, 1H, Ar*CH2*), 4.42 (ddd, *J* = 10.4, 6.3, 3.5 Hz, 1H, H_{2D}), 4.35 (d, *J* = 10.2 Hz, 1H, Ar*CH2*), 4.12 (dd, *J* = 10.5, 3.6 Hz, 1H, H_{2L}), 4.08 – 4.00 (m, 4H, H_{5M}, H_{5D}, H_{2M}, H_{4L}), 3.99 – 3.91 (m, 2H, H_{5L}, H_{3D}), 3.82 – 3.77 (m, 1H, H_{3L}), 3.74 – 3.72 (m, 4H, OCH3, H_{4M}), 3.64 – 3.61 (m, 1H, H_{4D}), 3.52 (dd, *J* = 9.3, 3.5 Hz, 1H, H_{3M}), 2.56 (d, *J* = 16.4 Hz, 1H, OH), 1.11 (d, *J* = 6.5 Hz, 3H, H_{6L}), 1.07 (d, *J* = 6.5 Hz, 3H, H_{6D}). **^{13}C NMR (126 MHz, $CDCl_3$)** δ 167.16 (C=O), 159.48 (*t*PMBC), 157.97 (CF_3C=O), 157.68 (CF_3C=O), 157.39 (CF_3C=O), 157.10 (CF_3C=O), 138.27, 137.33, 135.30, 134.81, 133.26, 132.99, 129.95, 129.63, 128.72, 128.63, 128.45, 128.36, 128.14, 128.06, 128.04, 127.87, 127.69, 127.45, 126.57, 125.95, 125.78, 119.42 (CF_3), 117.11 (CF_3), 114.84 (CF_3), 113.67 (PMB-CH), 112.55 (CF_3), 100.85 (C_{1M}), 99.24 (C_{1L}), 90.38 (C_{1D}), 79.69 (C_{3M}), 78.35 (C_{3D}), 77.86 (C_{4D}), 76.27 (C_{3L}), 75.24 (C_{4M}), 75.19 (C_{4L}), 74.92 (Ar*C*H_2), 74.86 (C_{5M}), 74.81 (Ar*C*H_2), 72.26 (Ar*C*H_2), 71.26 (Ar*C*H_2), 67.66 (C_{5L}), 67.32 (Ar*C*H_2), 66.39 (C_{5D}), 61.50 (C_{2M}), 60.26 (C_{2L}), 55.24 (OCH_3), 50.63 (C_{2D}), 16.92 (C_{6L}), 16.80 (C_{6D}). **ESI HRMS:** $[M + Na]^+$ calculated for $C_{60}H_{62}F_3N_7O_{14}Na$, 1184.4205; found 1184.4211.

(Benzyl (2-azido-3-*O*-benzyl-2-deoxy-4-*O*-*p*-methoxybenzyl-β-D-mannopyranosiduronsyl)-(1→4)-2-azido-2-deoxy-3-*O*-(2-naphthylmethyl)-α-L-fucopyranosyl-(1→3)-4-*O*-benzyl-2-deoxy-2-*N*-tricfluoroacetamide-β-D-fucopyranose (27-OH)

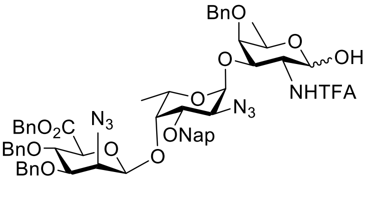

Trisaccharide **9** (600 mg, 0.438 mmol) was dissolved in THF (4.4 mL, 0.1M), a catalytic amount of AcOH and TBAF (0.876 mL, 0.876 mmol, 1.5 eq) were added at 0 °C. The resulting mixture was stirred at room temperature for 2 h until TLC analysis indicated the complete conversion of starting material. The reaction was diluted in EtOAc and washed with sat. NH_4Cl, brine. The organic phase was dried over Na_2SO_4, concentrated *in vacuo*. Purification by column chromatography (silica gel, petroleum ether/acetone = 3/1) give **27-OH** (406 mg, 82% yield). **^{1}H NMR (500 MHz, $CDCl_3$)** δ 7.84 – 7.72 (m, 5H, ArH), 7.61 (d, *J* = 6.5 Hz, 1H, NH), 7.55 – 7.50 (m, 1H, ArH), 7.46 – 7.40 (m, 3H, ArH), 7.35 – 7.27 (m, 7H, ArH), 7.26 – 7.21 (m, 4H, ArH), 7.18 – 7.09 (m, 4H, ArH), 7.07 – 7.00 (m, 4H, ArH), 5.51 (d, *J* = 3.6 Hz, 1H, H_{1D}), 4.93 (d, *J* = 3.8 Hz, 1H, H_{1L}), 4.91 – 4.87 (m, 1H, Ar*CH2*), 4.82 – 4.60 (m, 7H, Ar*CH2*), 4.63 (d, *J* = 1.1 Hz, 1H, H_{1M}), 4.53 (dd, *J* = 11.2, 2.4 Hz, 1H, Ar*CH2*), 4.46 – 4.37 (m, 2H, H_{2D}, Ar*CH2*), 4.16 – 3.90 (m, 7H, H_{2L}, H_{5M}, H_{2M}, H_{5D}, H_{4L}, H_{5L}, H_{3D}), 3.79 (dt, *J* = 10.5, 2.7 Hz, 1H, H_{3L}), 3.72 (ddd, *J* = 9.8, 3.5, 1.6 Hz, 1H, H_{4M}), 3.63 (d, *J* = 2.7 Hz, 1H, H_{4D}), 3.58 – 3.46 (m, 1H, H_{3M}), 1.13 – 1.09 (d, *J* = 6.6 Hz, 3H, H_{6L}), 1.09 – 1.05 (d, *J* = 6.6 Hz, 3H, H_{6L}). **^{13}C NMR (126 MHz, $CDCl_3$)** δ 167.32 (C=O), 158.04 (CF_3C=O), 157.74 (CF_3C=O), 157.45 (CF_3C=O), 157.16 (CF_3C=O), 138.32, 137.85, 137.82, 137.30, 135.32, 134.83, 133.32, 133.05, 128.74, 128.66, 128.48, 128.41, 128.30, 128.19, 128.10, 127.92, 127.88, 127.72, 127.69, 127.48, 126.62, 126.00, 125.83, 119.47, 117.17, 114.88, 112.60, 100.90 (C_{1M}), 99.30 (C_{1L}), 90.49 (C_{1D}), 79.75 (C_{3M}), 78.40 (C_{3D}), 77.92 (C_{4D}), 76.41 (C_{3L}), 75.31 (C_{4M}), 75.23 (C_{4L}), 75.21 (C_{5M}), 74.90 (Ar*C*H_2), 74.87 (Ar*C*H_2), 72.31 (Ar*C*H_2), 71.38 (Ar*C*H_2), 67.72 (C_{5L}), 67.38 (Ar*C*H_2), 66.51(C_{5D}), 61.56 (C_{2M}), 60.33 (C_{2L}), 50.65 (C_{2D}), 16.96 (C_{6L}), 16.84 (C_{6D}). **ESI HRMS**: $[M + Na]^+$ calculated for $C_{59}H_{60}F_3N_7O_{13}Na$, 1154.4099; found 1154.4093

(Benzyl (2-azido-3-*O*-benzyl-2-deoxy-4-*O*-*p*-methoxybenzyl-β-D-mannopyranosiduronsyl)-(1→4)-2-azido-2-deoxy-3-*O*-(2-naphthylmethyl)-α-L-fucopyranosyl-(1→3)-4-*O*-benzyl-2-deoxy-2-*N*-trichloroacetamide-1-*O*-(*N*-phenyl-2,2,2-trifluoroacetimidoyl)-β-D-fucopyranose (25)

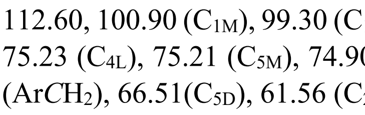

Hemiacetal **25-OH** (205 mg, 0.17 mmol) was co-evaporated with toluene (x3) before being dissolved in dry acetone (1.7 mL, 0.1 M). K_2CO_3 (47 g, 0.339 mmol, 2 equiv.) and CF_3C(NPh)Cl (0.06 mL, 0.339 mmol, 2 equiv.) were added and the reaction mixture was stirred overnight under nitrogen until TLC (pentane/EtOAc, 7:3) showed full conversion. The mixture was filtered and concentrated *in vacuo*. Column chromatography (pentane/EtOAc, 9:1 → 7:3) yielded imidate donor **25** in 97% yield (227 mg, 0.164 mmol).

^{1}H NMR (500 MHz, CD_3CN) δ 7.89 – 7.76 (m, 6H), 7.53 – 7.46 (m, 3H), 7.46 – 7.41 (m, 3H), 7.40 – 7.27 (m, 13H), 7.26 – 7.16 (m, 5H), 7.15 – 7.06 (m, 4H), 7.03 – 6.97 (m, 2H), 6.86 – 6.76 (m, 5H), 5.24 (s, 1H), 4.96 – 4.88 (m, 1H), 4.87 – 4.75 (m, 6H), 4.70 – 4.59 (m, 4H), 4.48 – 4.40 (m, 2H), 4.35 (dd, *J* = 3.6, 1.2 Hz, 1H), 4.33 – 4.28 (m, 2H), 4.27 – 4.22 (m, 2H), 4.09 – 4.03 (m, 2H), 3.95 – 3.91 (m, 1H), 3.90 – 3.86 (m, 2H), 3.85 – 3.81 (m, 3H), 3.77 – 3.75 (m, 2H), 3.74 (s, 3H), 1.23 (d, *J* = 6.3 Hz, 6H). **^{13}C NMR (126 MHz, CD_3CN)** δ 168.93, 163.36, 160.28, 144.65, 139.61, 139.16, 136.90, 136.29, 134.24, 133.90, 131.24, 130.60, 129.91, 129.44, 129.39, 129.37, 129.31, 129.25, 129.16, 129.03, 128.87, 128.84, 128.71, 128.63, 127.17, 127.06, 126.91, 126.87, 118.34, 114.51, 101.80, 99.57, 80.68, 79.02, 78.01, 76.71, 76.50, 76.35, 76.11, 75.89, 75.34, 72.38, 71.05, 70.81, 68.47, 67.88, 62.37, 60.99, 55.86, 52.18, 29.73, 17.13, 16.94. **ESI HRMS** (found for the hemiacetal): $[M+Na]^+$ calculated for $C_{60}H_{62}Cl_3N_7O_{14}Na$: 1234.32885; found 1234.32680.

(Benzyl (2-azido-3-*O*-benzyl-2-deoxy-4-*O*-*p*-methoxybenzyl-β-D-mannopyranosiduronsyl)-(1→4)-2-azido-2-deoxy-3-*O*-(2-naphthylmethyl)-α-L-fucopyranosyl-(1→3)-4-*O*-benzyl-2-deoxy-2-*N*-trifluoroacetamide-1-*O*-(*N*-phenyl-2,2,2-trifluoroacetimidoyl)-β-D-fucopyranose (26)

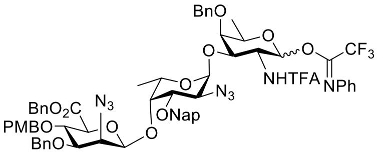

The hemiacetal **26-OH** wasdissolved in acetone, and K_2CO_3 (1.5 eq) and 2,2,2-Trifluoro-*N*-phenylacetimidoyl Chloride (2.0 eq) were added at 0 °C. The resulting mixture was stirred from 0 °C to room temperature for 14 h until completion. The reaction was quenched by Et_3N, filtered, concentrated *in vacuo*. Due to the compound is labile for silica gel during purification, the crude **26** was used for the next step without further purification.

(Benzyl (2-azido-3-*O*-benzyl-2-deoxy-4-*O*-*p*-methoxybenzyl-β-D-mannopyranosiduronsyl)-(1→4)-2-azido-2-deoxy-3-*O*-(2-naphthylmethyl)-α-L-fucopyranosyl-(1→3)-4-*O*-benzyl-2-deoxy-2-*N*-trifluoroacetamide-1-*O*-(*N*-phenyl-2,2,2-trifluoroacetimidoyl)-β-D-fucopyranose (27)

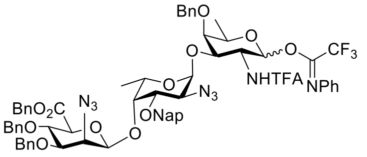

The hemiacetal **27-OH** wasdissolved in acetone, and K_2CO_3 (1.5 eq) and 2,2,2-Trifluoro-*N*-phenylacetimidoyl Chloride (2.0 eq) were added at 0 °C. The resulting mixture was stirred from 0 °C to room temperature for 14 h until completion. The reaction was quenched by Et_3N, filtered, concentrated *in vacuo*. Due to the compound is labile for silica gel during purification, the crude **27** was used for the next step without further purification.

5-(Benzyl(benzyloxycarbonyl)amino)pentyl (Benzyl (2-azido-3-*O*-benzyl-2-deoxy-4-*O*-*p*-methoxybenzyl-β-D-mannopyranosiduronsyl)-(1→4)-2-azido-2-deoxy-3-*O*-(2-naphthylmethyl)-α-L-fucopyranosyl-(1→3)-4-*O*-benzyl-2-deoxy-2-*N*-trichloroacetamide-α-D-fucopyranoside (29)

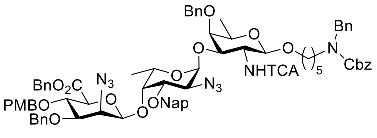

Donor **25** (256 mg, 0.185 mmol, 1 equiv.) and acceptor **21** (79 mg, 0.241 mmol, 1.3 equiv.) were co-evaporated with toluene (x3) before being dissolved in DCM/MeCN (1.9 mL, 1:1; 0.1 M). Activated 3Å molecular sieves were added and the solution was stirred for 30 min under argon at rt. The reaction mixture was cooled to -50 °C, followed by addition of TBSOTf (8.5 µL, 0.037 mmol, 0.2 equiv.). The mixture was allowed to warm to -30 °C and stirred for 1 h until TLC (pentane/EtOAc, 7:3) showed full conversion. The reaction was quenched with NEt_3 and diluted in EtOAc, washed with water (x1) and brine (x1), dried over Na_2SO_4, filtered and concentrated *in vacuo*. Column chromatography (pentane/EtOAc, 7:3 → 6:4) and size exclusion chromatography yielded trisaccharide **29** in 80% yield (225 mg, 0.148 mmol) as the sole β-anomer. **^{1}H NMR (500 MHz, $CDCl_3$)** δ 7.78 – 7.74 (m, 3H, Ar-*H*), 7.50 (dd, *J* = 8.5, 1.6 Hz, 1H, Ar-*H*), 7.45 – 7.42 (m, 2H, Ar-*H*), 7.36 – 7.12 (m, 25H, Ar-*H*), 7.08 – 7.06 (m, 1H, Ar-*H*), 7.00 – 6.96 (m, 2H, Ar-*H*), 6.79 – 6.74 (m, 2H, Ar-*H*), 5.15 (d, *J* = 10.9 Hz, 2H, C*H₂*Ph-linker), 4.94 (d, *J* = 3.8 Hz, 1H, H-1'), 4.87 (d, *J* = 11.5 Hz, 1H, Ar-C*H₂*), 4.85 – 4.78 (m, 2H, *H*-1, Ar-C*H₂*), 4.75 – 4.62 (m, 6H, Ar-C*H₂*), 4.59 (d, *J* = 1.1 Hz, 1H, H-1''), 4.46 (d, *J* = 7.9 Hz, 2H, C*H₂*Ph-linker), 4.42 (d, *J* = 11.4 Hz, 1H, Ar-C*H₂*), 4.35 (d, *J* = 10.2 Hz, 1H, Ar-C*H₂*), 4.28 (s, 1H, H-3), 4.05 (t, *J* = 9.5 Hz, 1H, H-4''), 4.01 (d, *J* = 3.2 Hz, 1H, H-2''), 3.95 (d, *J* = 2.9 Hz, 1H, H-4'), 3.90 (dd, *J* = 10.5, 3.8 Hz, 1H, H-2'), 3.82 (q, *J* = 6.7 Hz, 2H, H-5, H-2), 3.76 (s,

3H, OCH_3), 3.74 – 3.68 (m, 2H, H-5'', H-3'), 3.60 (q, *J* = 6.4 Hz, 1H, H-5'), 3.55 – 3.49 (m, 2H, H-3'', H-4), 3.37 (d, *J* = 21.3 Hz, 1H, CH_2-linker), 3.18 (d, *J* = 35.4 Hz, 2H, CH_2-linker), 1.58 – 1.40 (m, 4H, CH_2-linker), 1.26 (q, *J* = 14.5, 10.5 Hz, 12H, CH_2-linker, H-6'), 1.11 (d, *J* = 6.6 Hz, 3H, H-6). **^{13}C NMR (126 MHz, $CDCl_3$)** δ 167.25 (*C*=O), 162.09 (*C*=O), 159.36 (*C*=O), 138.53 (Ar-C_q), 138.02 (Ar-C_q), 137.43 (Ar-C_q), 135.45 (Ar-C_q), 134.94 (Ar-C_q), 133.37 (Ar-C_q), 133.08 (Ar-C_q), 130.13 (Ar-C_q), 129.70 (Ar-*C*), 128.80 (Ar-*C*), 128.74 (Ar-*C*), 128.62 (Ar-*C*), 128.55 (Ar-*C*), 128.40 (Ar-*C*), 128.25 (Ar-*C*), 128.15 (Ar-*C*), 128.13 (Ar-*C*), 127.95 (Ar-*C*), 127.79 (Ar-*C*), 127.71 (Ar-*C*), 127.51 (Ar-*C*), 126.57 (Ar-*C*), 126.07 (Ar-*C*), 126.02 (Ar-*C*), 125.88 (Ar-*C*), 113.79 (Ar-*C*), 101.01 (C-1''), 99.31 (C-1'), 99.24 (C-1), 79.90 (C-3''/C-4), 79.34 (C-3''/C-4), 78.02 (C-3), 75.47 (C-5''/C-3'), 75.31 (C-5''/C-3'), 75.28 (Ar-CH_2), 75.01 (Ar-CH_2), 74.91 (C-4''/C-4'), 74.91 (C-4''/C-4'), 72.34 (Ar-CH_2), 70.86 (Ar-CH_2), 70.67 (C-5'), 67.38 (Ar-CH_2), 67.23 (CH_2Ph-linker), 66.97 (C-5), 61.42 (C-2''), 59.28 (C-2'), 55.98 (C-2), 55.36 (OCH_3), 50.61 (CH_2Ph-linker), 50.34 (CH_2Ph-linker), 48.21 (CH_2-linker), 46.44 (CH_2-linker), 29.28 (CH_2-linker), 27.51 (CH_2-linker), 23.44 (CH_2-linker), 23.35 (CH_2-linker), 17.21 (C-6'), 17.14 (C-6). **ESI HRMS**: $[M+NH_4]^+$ calculated for $C_{80}H_{85}Cl_3N_8O_{16}NH_4$:1536.54929; found 1536.54874

1-allylbutanyl (Benzyl (2-azido-3-*O*-benzyl-2-deoxy-4-*O*-*p*-methoxybenzyl-β-D-mannopyranosiduronsyl)-(1→4)-2-azido-2-deoxy-3-*O*-(2-naphthylmethyl)-α-L-fucopyranosyl-(1→3)-4-*O*-benzyl-2-deoxy-2-*N*-trifluoroacetamide-α-D-fucopyranoside (31)

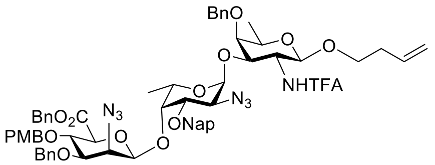

The imidate donor **26** (500 mg, 0.375 mmol) and allylcarbinol **28** (163 mg, 2.25 mmol) were co-evaporated with toluene for three times. The residue was dissolved in dry DCM/ MeCN (22.5 mL, 0.1 M). The reaction mixture was cooled to -78 °C and followed by adding TBSOTf (20 mg, 0.075 mmol 0.2 eq), and the reaction was allowed to stir for 15 min at -78 °C until TLC analysis indicated the complete conversion of starting material. The reaction was quenched with Et_3N, diluted in DCM, washed with brine, and the organic phase was dried over Na_2SO_4, concentrated *in vacuo*. Purification by column chromatography (silica gel, petroleum ether/EtOAc, 6/1) give the product **31** (360 mg, 79% yield). **^{1}H NMR (500 MHz, $CDCl_3$)** δ 7.83 – 7.74 (m, 4H, ArH), 7.52 (dd, *J* = 8.5, 1.6 Hz, 1H, ArH), 7.47 – 7.41 (m,3H, ArH), 7.37 – 7.12 (m, 13H, ArH), 7.11 – 7.04 (m, 2H, ArH), 6.98 (d, *J* = 8.7 Hz, 2H, ArH), 6.91 (d, *J* = 7.4 Hz, 1H, NH), 6.76 (d, *J* = 8.6 Hz, 2H, ArH), 5.80 – 5.68 (m, 1H, *CH*=CH_2), 5.08 – 4.97 (m, 2H, CH=CH_2), 4.89 (d, *J* = 3.9 Hz, 1H, H_{1L}), 4.87 (d, *J* = 11.0 Hz, 1H, ArCH_2), 4.81 (d, *J* = 12.0 Hz, 1H, ArCH_2), 4.89 (d, *J* = 3.3 Hz, 1H, H_{1D}), 4.75 – 4.63 (m, 6H, ArCH_2), 4.60 (d, *J* = 1.1 Hz, 1H, H_{1M}), 4.45 (d, *J* = 11.3 Hz, 1H, ArCH_2), 4.35 (d, *J* = 10.2 Hz, 1H, ArCH_2), 4.12 – 4.02 (m, 2H, H_{3D}, H_{5M}), 4.01 – 3.89 (m, 5H, H_{2M}, H_{2L}, H_{4L}, H_{2D}, OCH_2), 3.85 – 3.80 (m, 1H, H_{5D}), 3.76 (s, 3H, OCH_3), 3.73 – 3.67 (m, 2H, H_{3L}, H_{4M}), 3.62 – 3.57 (m, 1H, H_{5L}), 3.55 (d, *J* = 2.8 Hz, 1H, H_{4D}), 3.53 – 3.41 (m, 2H, H_{3M}, OCH_2), 2.35 – 2.24 (m, 2H, CH_2CH=CH_2), 1.26 (d, *J* = 5.6 Hz, 3H, H_{6L}), 1.08 (d, *J* = 6.6 Hz, 3H, H_{6D}). **^{13}C NMR (126 MHz, $CDCl_3$)** δ 167.31 (C=O), 159.37, 157.95 (CF_3C=O), 157.66 (CF_3C=O), 138.52, 137.46, 135.47, 134.96, 134.83 (*C*H=CH_2), 133.40, 133.11, 130.14, 129.71, 128.84, 128.76, 128.58, 128.42, 128.26, 128.17, 128.15, 127.97, 127.81, 127.73, 127.41, 126.65, 126.09, 125.90, 116.70 (CH=CH_2), 113.80 (PMBCH), 100.99 (C_{1M}), 99.79 (C_{1D}), 99.54 (C_{1L}), 79.90 (C_{3M}), 79.39 (C_{3D}), 78.83 (C_{4D}), 75.70 (C_{3L}), 75.43 (C_{4M}), 75.20 (ArCH_2), 75.07 (C_{5M}), 75.02 (ArCH_2), 74.96 (C_{4L}), 72.38 (ArCH_2), 71.08 (ArCH_2), 70.78 (C_{5L}), 68.99 (OCH_2), 67.42 (ArCH_2), 67.23 (C_{5D}), 61.52 (C_{2M}), 59.69 (C_{2L}), 55.38 (OCH_3), 54.32 (C_{2D}), 33.92 (CH_2), 17.17 (C_{6L}), 17.11 (C_{6D}). **ESI HRMS:** $[M + Na]^+$ calculated for $C_{64}H_{68}F_3N_7O_{14}Na$, 1238.4674; found 1238.4678.

5-(Benzyl(benzyloxycarbonyl)amino)pentyl (Benzyl (2-azido-3-*O*-benzyl-2-deoxy-β-D-mannopyranosiduronsyl)-(1→4)-2-azido-2-deoxy-3-*O*-(2-naphthylmethyl)-α-L-fucopyranosyl-(1→3)-4-*O*-benzyl-2-deoxy-2-*N*-trichloroacetamide-α-D-fucopyranoside (30)

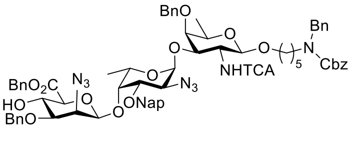

Trisaccharide **29** (268 mg, 0.176 mmol) was dissolved in DCM (1.8 mL, 0.1 M) and cooled to 0 °C after which TES (0.14 mL, 0.881 mmol, 5 equiv.) and HCl in HFIP (0.2 M, 0.26 mL, 0.3 equiv.) were added to the solution. The reaction was stirred for 1 h at 0 °C until TLC (pentane/EtOAc, 6:4) showed

full conversion. The reaction mixture was quenched by the addition of $NaHCO_3$ (sat. aq.) and diluted in EtOAc. The organic phase was washed with $NaHCO_3$ (sat. aq.; x1) and brine (x1), dried over Na_2SO_4, filtered and concentrated *in vacuo*. Column chromatography (pentane/EtOAc, 7:3 → 5:5) afforded acceptor **30** in 78% yield (192 mg, 0.137 mmol). **^{1}H NMR (500 MHz, $CDCl_3$)** δ 7.83 – 7.77 (m, 1H, Ar-*H*), 7.77 – 7.71 (m, 3H, Ar-*H*), 7.50 (dd, *J* = 8.4, 1.7 Hz, 1H, Ar-*H*), 7.42 – 7.26 (m, 17H, Ar-*H*), 7.26 – 7.14 (m, 6H, Ar-*H*), 7.13 – 7.08 (m, 2H, Ar-*H*), 5.17 (d, *J* = 11.5 Hz, 2H, C*H₂*Ph-linker), 4.95 (s, 1H, H-1'), 4.93 – 4.88 (m, 2H, Ar-C*H₂*), 4.83 (d, *J* = 8.3 Hz, 1H, H-1), 4.79 – 4.68 (m, 4H, Ar-C*H₂*), 4.66 (s, 1H, H-1''), 4.64 (s, 1H, Ar-C*H₂*), 4.48 (d, *J* = 6.6 Hz, 2H, C*H₂*Ph-linker), 4.43 (d, *J* = 11.4 Hz, 1H, Ar-C*H₂*), 4.28 (d, *J* = 10.7 Hz, 1H, H-3), 4.18 (t, *J* = 9.4 Hz, 1H, H-4''), 4.03 (d, *J* = 3.6 Hz, 1H, H-2''), 4.00 (d, *J* = 2.9 Hz, 1H, H-4'), 3.91 (dd, *J* = 10.6, 3.7 Hz, 1H, H-2'), 3.87 – 3.78 (m, 2H, H-2, H-5), 3.74 – 3.69 (m, 2H, H-3', H-5''), 3.61 (q, *J* = 6.6 Hz, 1H, H-5'), 3.53 (d, *J* = 2.8 Hz, 1H, H-4), 3.45 (s, 1H, H-3''), 3.41 – 3.32 (m, 1H, C*H₂*-linker), 3.28 – 3.12 (m, 2H, C*H₂*-linker), 1.59 – 1.43 (m, 4H, C*H₂*-linker), 1.35 – 1.22 (m, 6H, C*H₂*-linker), H-6'), 1.14 (d, *J* = 6.6 Hz, 3H, H-6). **^{13}C NMR (126 MHz, $CDCl_3$)** δ 168.34 (*C*=O), 162.09 (*C*=O), 138.52 (Ar-C_q), 137.97 (Ar-C_q), 137.53 (Ar-C_q), 135.40 (Ar-C_q), 134.64 (Ar-C_q), 133.30 (Ar-C_q), 133.04 (Ar-C_q), 128.88 (Ar-*C*), 128.82 (Ar-*C*), 128.72 (Ar-*C*), 128.61 (Ar-*C*), 128.51 (Ar-*C*), 128.37 (Ar-*C*), 128.23 (Ar-*C*), 128.10 (Ar-*C*), 128.04 (Ar-*C*), 127.95 (Ar-*C*), 127.92 (Ar-*C*), 127.77 (Ar-*C*), 127.69 (Ar-*C*), 127.55 (Ar-*C*), 127.52 (Ar-*C*), 126.68 (Ar-*C*), 126.09 (Ar-*C*), 126.03 (Ar-*C*), 125.90 (Ar-*C*), 100.82 (C-1''), 99.24 (C-1'), 99.24 (C-1''), 79.22 (C-4), 78.64 (C-3''), 78.02 (C-3), 75.19 (Ar-*C*H₂), 75.12 (C-3'/C-5''), 75.03 (C-3'/C-5''), 74.69 (C-4'), 72.63 (Ar-*C*H₂), 70.72 (Ar-*C*H₂), 70.65 (C-5'), 69.86 (*C*H₂Ph-linker), 69.73 (*C*H₂Ph-linker), 68.01 (C-4''), 67.61 (N Ar-*C*H₂), 67.22 (*C*H₂Ph-linker), 66.91 (C-5), 61.33 (C-2''), 59.22 (C-2'), 55.88 (C-2), 50.58 (*C*H₂Ph-linker), 50.31 (*C*H₂-linker), 47.20 (*C*H₂-linker), 46.24 (*C*H₂-linker), 29.77 (*C*H₂-linker), 29.25 (*C*H₂-linker), 27.46 (*C*H₂-linker), 23.39 (*C*H₂-linker), 17.19 (C-6'), 17.13 (C-6). **ESI HRMS**: $[M+NH_4]^+$ calculated for $C_{72}H_{77}Cl_3N_8O_{15}NH_4$:1415.49177; found 1416.49122

1-allylbutanyl (Benzyl (2-azido-3-*O*-benzyl-2-deoxy-β-D-mannopyranosiduronsyl)-(1→4)-2-azido-2-deoxy-3-*O*-(2-naphthylmethyl)-α-L-fucopyranosyl-(1→3)-4-*O*-benzyl-2-deoxy-2-*N*-trifluoroacetamide-α-D-fucopyranoside (32)

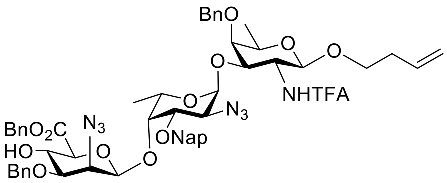

31 (340 mg, 0.28 mmol) and triethylsilane (163 mg, 1.4 mmol 1.0 eq) were dissolved in DCM:HFIP (hexafluoro-iso-propanol) (3 mL, 1:1, 0.1 M) . Then 0.2 M HCl in HFIP (0.28 mL, 0.056 mmol) was added to the mixture. The reaction was stirred until TLC-analysis indicated complete consumption of the starting material (30 min.). Then the mixture was diluted with DCM and the reaction quenched with saturated $NaHCO_3$. The organic phase was washed with water and brine, dried with anhydrous Na_2SO_4, filtered and concentrated *in vacuo*. Purification by column chromatography (silica gel, petroleum ether/acetone, 3/1) give the product **32** (230 mg, 75% yield). **^{1}H NMR (400 MHz, $CDCl_3$)** δ 7.82 – 7.72 (m, 4H, ArH), 7.52 – 7.42 (m, 3H, ArH), 7.40 – 7.14 (m, 13H, ArH), 7.11 – 7.05 (m, 2H, ArH), 6.84 (d, *J* = 7.3 Hz, 1H, NH), 5.75 (ddt, *J* = 17.0, 10.2, 6.7 Hz, 1H, *CH*=CH₂), 5.08 – 4.97 (m, 2H, CH=*CH₂*), 4.90 (dd, *J* = 11.6, 7.9 Hz, 2H, Ar*CH₂*), 4.86 (d, *J* = 3.7 Hz, 1H, H_{1L}), 4.71 (d, *J* = 3.3 Hz, 1H, H_{1D}), 4.77 – 4.65 (m, 5H, Ar*CH₂*), 4.63 (d, *J* = 1.2 Hz, 1H, H_{1M}), 4.42 (d, *J* = 11.3 Hz, 1H, Ar*CH₂*), 4.20 – 4.12 (m, 1H, C_{5M}), 4.07 (dd, *J* = 11.1, 2.8 Hz, 1H, H_{3D}), 4.02 – 3.85 (m, 5H, H_{2L}, H_{2D}, H_{2M}, OCH_3, H_{4L}), 3.84 – 3.76 (m, 1H, H_{5M}), 3.71 – 3.64 (m, 2H, H_{3L}, H_{4M}), 3.59 (q, *J* = 6.2 Hz, 1H, H_{5L}), 3.54 (d, *J* = 2.7 Hz, 1H, H4D), 3.47 (dt, *J* = 9.6, 7.1 Hz, 1H, OCH_2), 3.42 – 3.33 (m, 1H, H_{3M}), 2.95 (d, *J* = 2.8 Hz, 1H, OH), 2.35 – 2.24 (m, 2H, *CH₂*CH=CH₂), 1.26 (d, *J* = 4.5 Hz, 3H, H_{6L}), 1.09 (d, *J* = 6.6 Hz, 3H, H_{6D}). **^{13}C NMR (101 MHz, $CDCl_3$)** δ 168.36 (C=O), 157.98 (CF_3C=O), 157.62 (CF_3C=O), 138.52, 137.58, 135.47, 134.82 (*C*H=CH₂), 134.65, 133.34, 133.09, 128.77, 128.71, 128.69, 128.66, 128.41, 128.27, 128.10, 127.99, 127.82, 127.72, 127.43, 126.74, 126.13, 126.11, 125.94, 116.72 (CH=*C*H₂), 100.83 (C_{1M}), 99.74 (C_{1D}), 99.51 (C_{1L}), 79.40 (C_{3D}), 78.72 (C_{4D}), 78.56 (C_{3M}), 75.45 (C_{3L}), 75.14 (Ar*C*H₂), 75.10 (C_{4M}), 74.59 (C_{4L}), 72.70 (Ar*C*H₂), 70.88 (Ar*C*H₂), 70.78 (C_{5L}), 68.99 (O*C*H₂), 68.10 (C_{5M}),

67.68 (Ar*C*H_2), 67.18 (C_{5D}), 61.43 (C_{2M}), 59.64 (C_{2L}), 54.34 (C_{2D}), 33.91 (CH_2), 17.18 (C_{6L}), 17.16 (C_{6D}). **ESI HRMS:** $[M + Na]^+$ calculated for $C_{56}H_{60}F_3N_7O_{13}Na$, 1118.4099; found 1118.4095.

Hexasaccharide-protected with ONap on L-Fuc, Bn on D-Man and *N*-TCA (33)

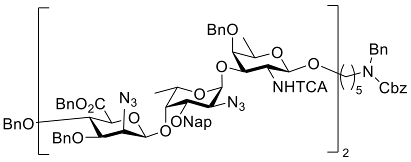

Acceptor **30** (57 mg, 0.041 mmol, 1 equiv.) and donor **20** (74 mg, 0.054 mmol, 1.3 equiv.) were co-evaporated with toluene (x3) before being dissolved in dry DCM/ MeCN (1 mL, 2:1; 0.04 M). Activated 3Å molecular sieves were added and the solution was stirred for 30 min under argon at rt. The mixture was cooled to -78 °C, after which TBSOTf (2 µL, mmol, 0.2 equiv.) was added. The reaction mixture was stirred at -78 °C for 1 h until TLC (pentane/EtOAc, 6.5:3.5) showed full conversion. The reaction mixture was quenched with NEt_3 and diluted in EtOAc. The organic phase was washed with $NaHCO_3$ (sat. aq.; x1) and brine (x1), dried over Na_2SO_4, filtered and concentrated *in vacuo*. Size exclusion column chromatography furnished hexamer **33** in 84% yield (88 mg, 0.0342 mmol) as the sole β-anomer. **^{1}H NMR (500 MHz, $CDCl_3$)** δ 7.78 (tdd, *J* = 14.4, 5.1, 3.2 Hz, 8H), 7.55 – 7.39 (m, 7H), 7.39 – 7.26 (m, 26H), 7.25 – 7.12 (m, 16H), 7.07 (dddd, *J* = 20.2, 8.5, 6.4, 2.0 Hz, 7H), 6.78 (d, *J* = 8.6 Hz, 1H), 5.17 (d, *J* = 13.1 Hz, 2H), 4.92 (dd, *J* = 11.9, 2.9 Hz, 3H), 4.84 (ddd, *J* = 12.1, 8.7, 5.9 Hz, 3H), 4.80 – 4.71 (m, 5H), 4.71 – 4.60 (m, 9H), 4.57 (d, *J* = 11.9 Hz, 1H), 4.51 – 4.45 (m, 3H), 4.42 (dd, *J* = 10.7, 3.5 Hz, 2H), 4.39 – 4.21 (m, 4H), 4.17 (ddd, *J* = 11.8, 9.4, 7.5 Hz, 1H), 4.12 – 4.03 (m, 2H), 4.01 (td, *J* = 8.3, 3.7 Hz, 3H), 3.97 (d, *J* = 3.1 Hz, 1H), 3.94 (t, *J* = 6.6 Hz, 1H), 3.88 (tdd, *J* = 10.3, 7.0, 3.7 Hz, 3H), 3.81 (dt, *J* = 10.4, 3.4 Hz, 3H), 3.77 – 3.72 (m, 2H), 3.72 – 3.66 (m, 1H), 3.65 – 3.58 (m, 1H), 3.57 – 3.50 (m, 2H), 3.50 – 3.44 (m, 1H), 3.43 (s, 2H), 3.39 – 3.31 (m, 1H), 3.23 (s, 1H), 3.16 (q, *J* = 6.4 Hz, 1H), 1.50 (d, *J* = 29.3 Hz, 3H), 1.30 – 1.25 (m, 5H), 1.18 (d, *J* = 6.4 Hz, 3H), 1.11 (td, *J* = 11.2, 10.1, 6.4 Hz, 6H). **^{13}C NMR (126 MHz, $CDCl_3$)** δ 167.96, 167.24, 162.07, 161.92, 138.63, 138.53, 138.31, 138.02, 137.96, 137.40, 135.49, 135.47, 134.92, 134.83, 133.39, 133.37, 133.06, 129.31, 128.85, 128.81, 128.78, 128.75, 128.73, 128.65, 128.52, 128.50, 128.42, 128.40, 128.35, 128.25, 128.21, 128.17, 128.15, 128.08, 128.06, 127.96, 127.94, 127.90, 127.85, 127.82, 127.79, 127.77, 127.72, 127.59, 127.54, 127.48, 127.36, 127.25, 127.21, 127.16, 126.72, 126.49, 126.05, 126.02, 126.00, 125.89, 125.83, 100.97, 100.71, 99.94, 99.24, 99.02, 80.21, 79.87, 79.24, 78.05, 77.16, 76.91, 76.03, 75.38, 75.31, 75.27, 75.23, 75.12, 74.93, 74.48, 74.11, 73.84, 72.38, 71.17, 70.90, 70.68, 70.66, 69.81, 69.79, 67.64, 67.36, 67.23, 66.94, 62.56, 61.58, 59.55, 59.20, 55.94, 54.05, 50.65, 50.38, 29.80, 29.26, 23.38, 17.20, 17.16, 17.09, 17.01. **ESI HRMS**: $[M+NH_4]^+$ calculated for $C_{113}H_{123}Cl_6N_{15}O_{29}NH_4$:2384.70901; found 2385.70722

CP5-*O*-Ac-Hexasaccharide (2)

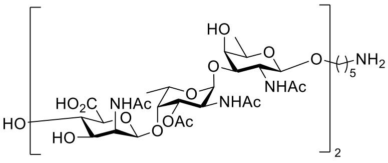

33 (129 mg, 0.051 mmol) was dissolved in DCM/H_2O (4:1, 5 mL, 0.01 M) and added DDQ (46 mg, 0.202 mmol, 4 equiv.). The reaction was stirred at rt under nitrogen for 5 h until TLC (pentane, EtOAc, 6:4) showed full conversion. The solution was quenched with $Na_2S_2O_3$ (sat. aq.) and diluted/extracted with EtOAc (x3). The combined organic phases were washed with $NaHCO_3$ (sat. aq.; x4) and brine (x1), dried over Na_2SO_4, filtrated and concentrated *in vacuo*. The crude was used without further purification. The residue was dissolved in pyridine (2 mL) and cooled to 0 °C and added Ac_2O (0.3 mL) and DMAP (catalytic amount) and stirred at rt under nitrogen overnight until TLC (pentane/acetone, 7:3) showed full conversion. The mixture was dissolved in EtOAc, washed with 1 M HCl (x1), $NaHCO_3$ (sat. aq.; x1) and brine (x1), dried over Na_2SO_4 and concentrated *in vacuo*. Column chromatography (pentane/acetone, 7:3) yielded the acetylated product in 73% yield (87 mg, 0.037 mmol). **^{1}H NMR (500 MHz, $CDCl_3$)** δ 7.51 – 7.28 (m, 44H), 7.24 – 7.04 (m, 13H), 6.77 (dd, *J* = 17.0, 8.2 Hz, 1H), 5.27 – 5.21 (m, 2H), 5.20 – 5.11 (m, 6H), 5.04 – 4.94 (m, 4H), 4.93 – 4.73 (m, 10H), 4.72 – 4.65 (m, 5H), 4.52 (d, *J* = 8.1 Hz, 1H), 4.50 – 4.40 (m, 7H), 4.37 – 4.21 (m, 4H), 4.20 – 3.93 (m, 12H), 3.91 – 3.76 (m, 6H), 3.75 – 3.59 (m, 6H), 3.60 – 3.40 (m, 7H), 3.28 – 3.09 (m, 4H), 2.04 – 2.01 (m, 6H), 1.78 – 1.38 (m, 10H), 1.31 (d, *J* = 6.4 Hz, 4H), 1.27 (dd, *J* = 7.8, 2.6 Hz, 4H), 1.19 (dd, *J* = 8.6, 6.3 Hz, 5H), 1.07 – 1.02 (m, 6H). **^{13}C NMR (126 MHz, $CDCl_3$)** δ 170.60, 170.34, 167.81, 167.60, 162.03, 138.28, 138.06, 137.81, 137.32, 135.08, 134.86,

133.00, 129.51, 128.97, 128.90, 128.86, 128.77, 128.64, 128.56, 128.53, 128.46, 128.44, 128.39, 128.32, 127.99, 127.96, 127.85, 127.75, 127.70, 127.56, 127.35, 101.12, 100.77, 99.10, 99.02, 98.99, 98.87, 79.57, 79.17, 78.62, 78.05, 77.94, 77.41, 75.75, 75.33, 75.27, 75.14, 74.99, 73.40, 72.39, 70.78, 70.60, 70.22, 67.94, 67.76, 67.26, 66.53, 62.00, 61.19, 57.77, 57.67, 56.05, 54.84, 50.60, 50.34, 47.17, 46.49, 29.24, 23.48, 20.88, 17.32, 17.18, 16.54.

The acetylated product (62 mg, 0.026 mmol) was dissolved in THF (distilled, 3 mL) and added zinc powder (510 mg, 7.804 mmol, 300 equiv.), AcOH (1 mL) and Ac_2O (0.5 mL). The resulting mixture was stirred at 50 °C overnight until TLC (DCM/MeOH, 95:5) showed full conversion. The cooled mixture was filtered through Celite, evaporated *in vacuo* and co-evaporated with toluene (x3). The crude product was first purified by column chromatography (DCM/MeOH, 98:2 → 90:10) followed by HPLC given intermediate in 11% yield (6.2 mg, 0.00279 mmol). The intermediate (5.2 mg, 0.00234 mmol) was dissolved in *t*-BuOH (1.5 mL) and added AcOH (1 mL, 0.1 mL in 100 mL MilliQ). Another 1 mL *t*-BuOH was added to dissolve the compound. The solution was birched with argon for 20 min and then $Pd(OH)_2/C$ (catalytic amount) was added. The reaction was again birched with argon for 5 minutes before the atmosphere was changed for H_2. The mixture was stirred for 3 days under H_2 atmosphere, after which it was filtered over a Whatman filter and lyophilized until NMR showed full conversion. Purification by a HW40 column with NH_4OAc followed by lyophilization gave **2** in 47% yield (1.5 mg, 0.0011 mmol). **^{1}H NMR (600 MHz, D_2O)** δ 5.03 – 4.96 (m, 4H), 4.74 (d, *J* = 1.4 Hz, 1H), 4.71 (s, 1H), 4.63 (dd, *J* = 4.4, 1.2 Hz, 1H), 4.59 (dd, *J* = 4.3, 1.4 Hz, 1H), 4.43 – 4.32 (m, 4H), 4.20 (d, *J* = 3.1 Hz, 2H), 4.16 (q, *J* = 6.5 Hz, 2H), 4.01 – 3.91 (m, 2H), 3.91 – 3.84 (m, 2H), 3.85 – 3.74 (m, 8H), 3.67 – 3.59 (m, 2H), 3.59 – 3.53 (m, 2H), 2.98 (t, *J* = 7.7 Hz, 2H), 2.13 (s, 3H), 2.12 (s, 3H), 2.07 (s, 3H), 2.04 (s, 3H), 1.99 (s, 6H), 1.98 (s, 3H), 1.97 (s, 3H), 1.67 (p, *J* = 7.7 Hz, 2H), 1.61 – 1.54 (m, 2H), 1.42 – 1.34 (m, 2H), 1.27 (dd, *J* = 8.2, 6.4 Hz, 6H), 1.23 (dd, *J* = 6.6, 3.2 Hz, 6H). **^{13}C NMR (151 MHz, D_2O)** δ 176.53, 176.45, 175.94, 175.47, 175.20, 175.16, 174.99, 174.72, 174.61, 174.39, 102.43, 102.34, 100.81, 100.75, 100.01, 99.93, 79.96, 79.05, 78.11, 77.96, 77.90, 76.99, 76.52, 72.54, 71.66, 71.63, 71.32, 71.24, 71.03, 70.96, 70.80, 70.61, 70.27, 67.71, 53.91, 52.89, 52.22, 47.98, 40.24, 29.07, 27.31, 23.37, 23.09, 23.05, 22.97, 22.94, 22.89, 21.23, 21.15, 16.26, 16.22, 16.16, 16.09. **ESI HRMS**: $[M+H]^+$ calculated for $C_{57}H_{91}N_7O_{31}H$: 1370.58377; found 1370.58355

Hexasaccharide-protected with ONap on L-Fuc, PMB on D-Man and *N*-TCA (34)

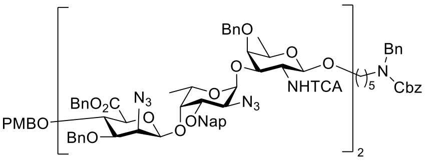

Acceptor **30** (61 mg, 0.0435 mmol) and donor **25** (91 mg, 0.0655 mmol, 1.5 equiv.) were co-evaporated with toluene (x3) before being dissolved in dry DCM/ MeCN (1 mL, 2:1; 0.04 M). Activated 3Å molecular sieves were added and the solution was stirred for 30 min under argon at rt. The mixture was cooled to -78 °C, after which TBSOTf (2 µL, 0.0087 mmol, 0.2 equiv.) was added. The reaction mixture was stirred at -78 °C for 1 h until TLC (pentane/EtOAc, 7:3) showed full conversion. The reaction mixture was quenched with Et_3N and diluted in EtOAc. The organic phase was washed with $NaHCO_3$ (sat. aq.; x1) and brine (x1), dried over Na_2SO_4, filtered and concentrated *in vacuo*. Size exclusion column chromatography furnished hexamer **34** in 80% yield (85 mg, 0.0329 mmol) as the sole β-anomer. **^{1}H NMR (500 MHz, $CDCl_3$)** δ 7.78 (ddd, *J* = 17.7, 8.9, 3.8 Hz, 10H), 7.54 – 7.39 (m, 9H), 7.41 – 7.12 (m, 52H), 7.13 – 7.03 (m, 6H), 7.00 – 6.93 (m, 3H), 6.77 (dd, *J* = 8.5, 5.3 Hz, 4H), 5.16 (d, *J* = 12.1 Hz, 2H), 4.91 (dd, *J* = 11.6, 3.0 Hz, 4H), 4.87 – 4.80 (m, 4H), 4.76 (dq, *J* = 15.6, 9.0, 7.9 Hz, 5H), 4.72 – 4.59 (m, 13H), 4.56 (d, *J* = 11.9 Hz, 1H), 4.47 (t, *J* = 8.8 Hz, 4H), 4.39 – 4.29 (m, 4H), 4.26 (dt, *J* = 14.1, 8.7 Hz, 3H), 4.20 – 4.13 (m, 1H), 4.08 – 3.97 (m, 7H), 3.96 (s, 1H), 3.92 (t, *J* = 6.5 Hz, 1H), 3.90 – 3.79 (m, 6H), 3.77 (s, 5H), 3.74 – 3.65 (m, 5H), 3.61 (t, *J* = 6.4 Hz, 1H), 3.55 – 3.49 (m, 3H), 3.48 – 3.32 (m, 5H), 3.25 – 3.21 (m, 1H), 3.15 (q, *J* = 6.4 Hz, 2H), 1.58 – 1.42 (m, 4H), 1.28 (d, *J* = 6.2 Hz, 4H), 1.17 (d, *J* = 6.4 Hz, 4H), 1.15 – 1.06 (m, 9H). **^{13}C NMR (126 MHz, $CDCl_3$)** δ 167.96, 167.26, 161.93, 159.74, 138.63, 138.32, 138.01, 137.46, 135.49, 134.96, 134.81, 133.39, 133.06, 130.12, 129.69, 129.33, 128.78, 128.74, 128.66, 128.63, 128.54, 128.52, 128.42, 128.41, 128.36, 128.26, 128.18, 128.08, 127.95, 127.86, 127.77, 127.73, 127.60, 127.55, 127.16, 126.73, 126.49, 126.01, 125.90, 125.83, 113.78, 100.99, 100.72, 99.97, 99.25, 99.02, 80.23,

79.88, 78.04, 77.41, 76.02, 75.42, 75.30, 75.25, 75.10, 74.45, 74.14, 73.86, 72.40, 71.15, 70.68, 70.64, 67.66, 67.39, 67.35, 66.93, 62.56, 61.62, 59.55, 59.19, 55.95, 55.37, 54.03, 17.21, 17.10, 17.02 **ESI HRMS**: $[M+NH_4]^+$ calculated for $C_{132}H_{137}Cl_6N_{15}O_{28}NH_4$: 2610.82364; found 2611.82279

Hexasaccharide-protected with ONap on L-Fuc, PMB on D-Man and *N*-TFA (36)

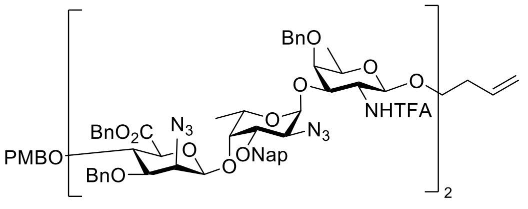

The imidate donor **26** (600 mg, 0.45 mmol, 1.5 eq) and acceptor **32** (328 mg, 0.3 mmol) were co-evaporated with toluene for three times. The residue was dissolved in dry DCM/MeCN (3 mL, 0.1 M). The reaction mixture was cooled to -78 °C and followed by adding TBSOTf (16 mg, 0.06 mmol 0.2 eq), and the reaction was allowed to stir for 30 min at -78 °C until TLC analysis indicated the complete conversion of starting material. The reaction was quenched with Et_3N, diluted in DCM, washed with brine, and the organic phase was dried over Na_2SO_4, concentrated *in vacuo*. Purification by size-exclusion and column chromatography (silica gel, petroleum ether/acetone, 3/1) give the product **36** (500 mg, 75% yield). Characterization for β-anomer: **^{1}H NMR (500 MHz, $CDCl_3$)** δ 7.85 – 7.70 (m, 9H, ArH), 7.53 – 7.41 (m, 7H, ArH), 7.40 – 7.12 (m, 24H, ArH), 7.11 – 7.03 (m, 5H, ArH), 6.99 – 6.92 (m, 2H, ArH), 6.83 (d, *J* = 7.4 Hz, 1H, NH), 6.78 – 6.71 (m, 2H, ArH), 6.44 (d, *J* = 8.6 Hz, 1H, NH), 5.80 – 5.69 (m, 1H, *CH*=CH_2), 5.08 – 4.97 (m, 2H, CH=*CH_2*), 4.90 – 4.81 (m, 4H, $ArCH_2$, H_{1L}, $H_{1L'}$), 4.81 – 4.62 (m, 14H, $ArCH_2$, H_{1D}), 4.62 – 4.56 (m, 3H, $ArCH_2$, H_{1M}, $H_{1M'}$), 4.46 (dd, *J* = 19.0, 11.4 Hz, 2H, $ArCH_2$,), 4.35 (dd, *J* = 17.5, 10.6 Hz, 2H, $ArCH_2$,), 4.16 – 3.86 (m, 13H), 3.97 (d, *J* = 5.5 Hz, 1H, $H_{1D'}$), 3.79 – 3.73 (m, 5H, OCH_3), 3.72 – 3.63 (m, 3H), 3.59 (d, *J* = 6.5 Hz, 1H, H5), 3.54 (d, *J* = 2.8 Hz, 1H), 3.51 – 3.31 (m, 5H), 3.17 (dd, *J* = 11.0, 2.7 Hz, 1H), 3.07 (d, *J* = 6.5 Hz, 1H, H5), 2.37 – 2.24 (m, 2H, CH_2), 1.28 – 1.22 (m, 3H, H_6), 1.12 (d, *J* = 6.3 Hz, 3H, H_6), 1.06 (t, *J* = 6.5 Hz, 6H, 2xH_6). **^{13}C NMR (126 MHz, $CDCl_3$)** δ 167.87, 167.29, 159.32, 157.88, 157.62, 138.54, 138.48, 138.17, 137.45, 135.49, 135.47, 135.04, 134.95, 134.80, 133.38, 133.36, 133.06, 130.09, 129.68, 129.45, 128.82, 128.73, 128.65, 128.55, 128.53, 128.41, 128.24, 128.20, 128.17, 128.07, 128.04, 127.93, 127.84, 127.82, 127.76, 127.73, 127.69, 127.60, 127.37, 127.11, 126.72, 126.61, 126.10, 126.06, 126.01, 125.99, 125.90, 125.82, 116.70 (CH=*CH_2*), 113.76, 100.93 (C_{1M}), 100.68 (C_{1M}), 100.60 ($C_{1D'}$), 99.64 (C_{1D}), 99.51 (C_{1L}), 99.23 (C_{1L}), 81.03, 79.83, 79.27, 78.80, 77.40, 76.20, 75.43, 75.35, 75.16 (Ar*CH_2*), 75.07, 74.99 (Ar*CH_2*), 74.97, 74.86 (Ar*CH_2*), 74.49, 73.68 (Ar*CH_2*), 72.39 (Ar*CH_2*), 71.41 (Ar*CH_2*), 70.79, 70.77 (Ar*CH_2*), 70.60, 68.95 (OCH_2), 67.58, 67.53 (Ar*CH_2*), 67.37 (Ar*CH_2*), 67.10, 62.44 (C_{2M}), 61.67 (C_{2M}), 59.82 (C_{2L}), 59.54 (C_{2L}), 55.36 (OCH_3), 54.41 (C_{2D}), 52.23 (C_{2D}), 33.88 (CH_2), 17.17 (C_6), 17.13 (C_6), 17.05 (C_6), 16.94 (C_6). **ESI HRMS:** $[M + Na]^+$ calculated for $C_{116}H_{120}F_6N_{14}O_{26}Na$, 2261.8300; found 2261.8310. Characterization for α-anomer: **^{1}H NMR (500 MHz, $CDCl_3$)** δ 7.85 – 7.70 (m, 9H, ArH), 7.62 (d, *J* = 6.2 Hz, 1H, ArH), 7.51 (ddd, *J* = 13.9, 8.4, 1.7 Hz, 2H, ArH), 7.47 – 7.41 (m, 4H, ArH), 7.39 – 7.12 (m, 25H, ArH), 7.08 (ddd, *J* = 8.3, 6.2, 1.9 Hz, 4H, ArH), 6.98 (d, *J* = 8.6 Hz, 2H, ArH), 6.85 (d, *J* = 7.4 Hz, 1H), 6.76 (d, *J* = 8.6 Hz, 2H, ArH), 5.75 (ddt, *J* = 17.0, 10.2, 6.7 Hz, 1H), 5.58 (d, *J* = 3.6 Hz, 1H, $H_{1D\alpha}$), 5.08 – 4.97 (m, 2H), 4.95 – 4.84 (m, 5H), 4.80 (dd, *J* = 12.0, 10.4 Hz, 3H), 4.76 – 4.59 (m, 15H), 4.52 (d, *J* = 11.2 Hz, 1H), 4.43 (d, *J* = 11.4 Hz, 1H), 4.35 (d, *J* = 10.1 Hz, 1H), 4.18 – 3.86 (m, 15H), 3.84 – 3.63 (m, 10H), 3.59 (d, *J* = 6.7 Hz, 1H), 3.55 – 3.44 (m, 4H), 3.40 (dd, *J* = 9.2, 3.6 Hz, 1H), 2.35 – 2.21 (m, 2H), 1.27 – 1.24 (m, 3H), 1.14 (d, *J* = 6.5 Hz, 3H), 1.08 (dd, *J* = 6.6, 6.6 Hz, 6H). **^{13}C NMR (126 MHz, $CDCl_3$)** δ 167.31, 166.99, 159.37, 138.52, 138.33, 137.49, 136.83, 135.48, 135.26, 134.95, 134.82, 133.40, 133.10, 130.11, 129.72, 128.88, 128.84, 128.78, 128.72, 128.70, 128.67, 128.58, 128.47, 128.41, 128.29, 128.22, 128.17, 128.10, 127.98, 127.81, 127.53, 127.39, 127.13, 126.76, 126.66, 126.11, 126.05, 125.88, 116.73, 113.80, 101.15, 101.04, 99.76, 99.49, 99.04, 96.67 ($C_{1D\alpha}$), 79.77, 79.66, 79.47, 78.80, 78.75, 76.42, 75.80, 75.75, 75.60, 75.36, 75.17, 75.05, 75.00, 74.68, 72.48, 71.49, 71.46, 71.18, 70.78, 69.65, 69.00, 67.73, 67.69, 67.63, 67.41, 67.20, 61.80, 60.61, 60.24, 59.70, 55.39, 54.30, 33.91, 17.18, 17.08, 16.65. **ESI HRMS:** $[M + Na]^+$ $C_{116}H_{120}F_6N_{14}O_{26}Na$, 2261.8300; found 2261.8311.

Hexasaccharide-protected with ONap on L-Fuc and *N*-TCA as acceptor (35)

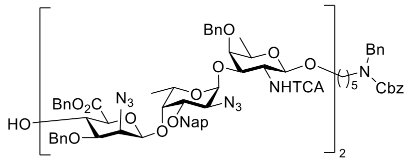

Hexasaccharide **34** (179 mg, 0.0692 mmol) was dissolved in DCM (1.4 mL, 0.05 M) and cooled to 0 °C after which TES (0.06 mL, 0.346 mmol, 5 equiv.) and HCl in HFIP (0.2 M, 0.1 mL, 0.2 equiv.) were added to the solution. The reaction was stirred for 1 h at 0 °C until TLC (pentane/EtOAc, 6:4) showed full conversion. The reaction mixture was quenched by the addition of $NaHCO_3$ (sat. aq.) and diluted in EtOAc. The organic phase was washed with $NaHCO_3$ (sat. aq.; x1) and brine (x1), dried over Na_2SO_4, filtered and concentrated *in vacuo*. Column chromatography (pentane/EtOAc, 6:4 → 5:5) afforded acceptor **35** in 78% yield (133 mg, 0.054 mmol). **^{1}H NMR (500 MHz, $CDCl_3$)** δ 7.84 – 7.68 (m, 9H), 7.50 – 7.42 (m, 7H), 7.40 – 7.28 (m, 22H), 7.26 – 7.10 (m, 20H), 7.07 (ddd, *J* = 7.8, 6.0, 2.3 Hz, 3H), 6.76 (dd, *J* = 8.7, 4.3 Hz, 1H), 5.16 (d, *J* = 12.6 Hz, 2H), 4.97 – 4.89 (m, 4H), 4.88 – 4.78 (m, 5H), 4.78 – 4.71 (m, 5H), 4.71 – 4.58 (m, 9H), 4.54 (d, *J* = 11.8 Hz, 1H), 4.45 (dd, *J* = 19.2, 10.1 Hz, 4H), 4.33 – 4.18 (m, 4H), 4.18 – 4.08 (m, 3H), 4.08 – 3.97 (m, 5H), 3.95 (d, *J* = 3.6 Hz, 1H), 3.93 – 3.87 (m, 2H), 3.87 – 3.74 (m, 6H), 3.71 (dd, *J* = 9.5, 2.5 Hz, 1H), 3.69 – 3.62 (m, 3H), 3.60 (q, *J* = 6.2 Hz, 1H), 3.51 (d, *J* = 2.8 Hz, 1H), 3.47 – 3.31 (m, 5H), 3.22 (s, 1H), 3.14 (q, *J* = 6.4 Hz, 2H), 2.90 (d, *J* = 2.8 Hz, 1H), 1.43 (q, *J* = 5.3, 4.4 Hz, 5H), 1.31 – 1.24 (m, 8H), 1.15 (dd, *J* = 10.6, 6.3 Hz, 5H), 1.10 (dd, *J* = 6.6, 3.8 Hz, 6H). **^{13}C NMR (126 MHz, $CDCl_3$)** δ 168.31, 167.99, 161.94, 138.65, 138.32, 138.02, 137.58, 135.51, 134.80, 134.69, 133.34, 133.04, 129.34, 128.77, 128.67, 128.64, 128.59, 128.51, 128.42, 128.38, 128.27, 128.17, 128.11, 128.05, 127.99, 127.97, 127.88, 127.79, 127.62, 127.59, 127.57, 127.34, 127.20, 126.74, 126.60, 126.07, 126.04, 125.88, 100.81, 100.72, 99.99, 99.25, 98.97, 80.24, 79.27, 78.56, 78.05, 75.81, 75.29, 75.08, 74.89, 74.72, 74.42, 74.18, 73.87, 72.72, 71.00, 68.11, 67.60, 67.28, 66.94, 62.55, 61.53, 59.49, 59.18, 55.95, 54.01, 29.83, 17.20, 17.04. **ESI HRMS**: $[M+NH_4]^+$ calculated for $C_{124}H_{129}Cl_6N_{15}O_{27}NH_4$: 2490.76613; found 2491.76488

Hexasaccharide-protected with ONap on L-Fuc and *N*-TFA as acceptor (37)

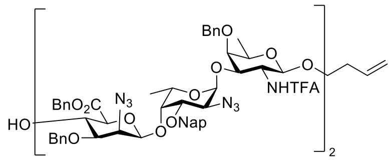

37 (498 mg, 0.223 mmol) and triethylsilane (129 mg, 1.113 mmol, 1.0 eq) were dissolved in DCM:HFIP (11 mL, 1:1, 0.1 M). Then 0.2 M HCl in HFIP (0.223 mL, 0.0446 mmol) was added to the mixture. The reaction was stirred until TLC-analysis indicated complete consumption of the starting material (30 min.). Then the mixture was diluted with DCM and the reaction quenched with saturated $NaHCO_3$. The organic phase was washed with water and brine, dried with anhydrous Na_2SO_4, filtered and concentrated *in vacuo*. Purification by column chromatography (silica gel, petroleum ether/acetone, 5/2) give the product **37** (325 mg, 69% yield). **^{1}H NMR (400 MHz, $CDCl_3$)** δ 7.85 – 7.65 (m, 8H), 7.53 – 7.41 (m, 6H), 7.40 – 7.12 (m, 25H), 7.11 – 6.99 (m, 5H), 6.84 (d, *J* = 7.3 Hz, 1H, NH), 6.45 (d, *J* = 8.7 Hz, 1H, NH), 5.75 (ddt, *J* = 17.0, 10.2, 6.7 Hz, 1H, *CH*=CH_2), 5.09 – 4.97 (m, 2H, CH=$\mathit{CH_2}$), 4.92 – 4.55 (m, 17H, Ar$\mathit{CH_2}$, H_{1L}, $H_{1L'}$, H_{1D}, H_{1M}, $H_{1M'}$), 4.45 (dd, *J* = 11.4, 5.8 Hz, 2H, Ar$\mathit{CH_2}$), 4.36 (d, *J* = 11.2 Hz, 1H, Ar$\mathit{CH_2}$), 4.18 – 3.84 (m, 13H), 4.02 (d, *J* = 5.5 Hz, 1H, $H_{1D'}$), 3.84 – 3.57 (m, 8H), 3.55 – 3.29 (m, 6H), 3.16 (dd, *J* = 11.0, 2.7 Hz, 1H), 3.07 (d, *J* = 6.6 Hz, 1H, H5), 2.90 (s, 1H, OH), 2.36 – 2.23 (m, 2H, CH_2), 1.26 (d, *J* = 6.7 Hz, 3H, H_6), 1.09 (dd, *J* = 19.3, 6.4 Hz, 9H, 2xH_6). **^{13}C NMR (126 MHz, $CDCl_3$)** δ 168.23, 167.80, 158.11 (CF_3C=O), 157.82 (CF_3C=O), 157.53 (CF_3C=O), 157.24 (CF_3C=O), 138.51, 138.46, 138.12, 137.54, 135.45, 134.98, 134.74, 134.65, 133.30, 133.27, 133.00, 132.97, 129.32, 128.65, 128.57, 128.53, 128.47, 128.34, 128.14, 128.09, 128.04, 127.94, 127.89, 127.73, 127.69, 127.64, 127.60, 127.51, 127.29, 127.05, 126.65, 126.62, 126.05, 125.96, 125.83, 125.78, 117.23 (CF_3), 116.97 (CF_3), 116.60 (CH=*C*H_2), 114.94 (CF_3), 114.68 (CF_3), 100.69 (C_{1M}), 100.61 (C_{1M}), 100.46 ($C_{1D'}$), 99.67 (C_{1D}), 99.41 (C_{1L}), 99.14 (C_{1L}), 80.82, 79.32, 78.70, 78.62, 77.50, 75.88, 75.42, 75.25, 75.08 (Ar*C*H_2), 75.06, 74.95, 74.57 (Ar*C*H_2), 74.81, 74.78 (Ar*C*H_2), 74.49, 73.54 (Ar*C*H_2), 72.59 (Ar*C*H_2), 71.12 (Ar*C*H_2), 70.76 (Ar*C*H_2), 70.69, 70.55, 68.82 (OCH_2), 67.95, 67.47 (Ar*C*H_2), 67.41, 67.06, 62.35 (C_{2M}), 61.50 (C_{2M}), 59.68 (C_{2L}), 59.53 (C_{2L}), 54.13 (C_{2D}), 52.25 (C_{2D}), 33.81 (CH_2), 17.08 (C_6), 17.01 (C_6), 16.97 (C_6), 16.86 (C_6). **ESI HRMS:** $[M + Na]^+$ calculated for $C_{108}H_{112}F_6N_{14}O_{25}Na$, 2141.7725; found 2141.7734.

Nonasaccharide-protected with ONap on L-Fuc, Bn on D-Man and *N*-TCA (38)

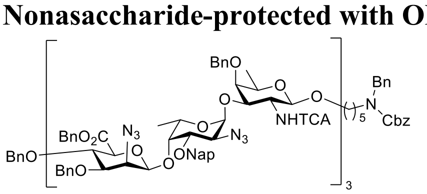

Acceptor **35** (129 mg, 0.0524 mmol) and donor **20** (106 mg, 0.0787 mmol, 1.5 equiv.) were co-evaporated with toluene (x3) before being dissolved in dry DCM/MeCN (0.7 mL, 2:1; 0.075 M). Activated 3Å molecular sieves were added and the solution was stirred for 30 min under argon at rt. The mixture was cooled to -78 °C, after which TBSOTf (2.4 µL, 0.0105 mmol, 0.2 equiv.) was added. The reaction mixture was stirred at -78 °C for 4 h until TLC (pentane/acetone, 6.5:3.5) showed full conversion. The reaction mixture was quenched with Et_3N and diluted in EtOAc. The organic phase was washed with $NaHCO_3$ (sat. aq.; x1) and brine (x1), dried over Na_2SO_4, filtered and concentrated *in vacuo*. Size exclusion column chromatography furnished nonamer **38** in 74% yield (141 mg, 0.0388 mmol) as the sole β-anomer. **^{1}H NMR (600 MHz, CDCl$_3$)** δ 7.85 – 7.71 (m, 11H), 7.56 – 7.42 (m, 9H), 7.42 – 7.28 (m, 23H), 7.24 – 7.11 (m, 19H), 7.11 – 6.99 (m, 8H), 6.80 – 6.73 (m, 2H), 5.17 (d, *J* = 15.4 Hz, 2H), 4.96 – 4.87 (m, 5H), 4.88 – 4.79 (m, 5H), 4.79 – 4.58 (m, 18H), 4.55 (d, *J* = 11.7 Hz, 1H), 4.48 (td, *J* = 7.9, 3.6 Hz, 4H), 4.41 (d, *J* = 10.7 Hz, 1H), 4.35 (t, *J* = 11.1 Hz, 2H), 4.31 – 4.20 (m, 5H), 4.20 – 4.12 (m, 2H), 4.11 – 3.95 (m, 9H), 3.95 – 3.66 (m, 13H), 3.61 (q, *J* = 6.3 Hz, 1H), 3.53 (dd, *J* = 8.9, 3.4 Hz, 2H), 3.46 (dd, *J* = 7.7, 4.4 Hz, 2H), 3.43 – 3.31 (m, 5H), 3.25 – 3.10 (m, 4H), 1.50 (ddd, *J* = 39.9, 14.5, 7.3 Hz, 4H), 1.28 (d, *J* = 5.6 Hz, 6H), 1.20 – 1.07 (m, 13H). **^{13}C NMR (151 MHz, CDCl$_3$)** δ 167.95, 167.91, 167.24, 161.96, 161.93, 138.60, 138.57, 138.50, 138.29, 138.28, 138.00, 137.93, 137.37, 135.54, 135.44, 134.88, 134.81, 134.78, 133.37, 133.34, 133.05, 133.01, 129.31, 129.29, 128.76, 128.73, 128.64, 128.62, 128.52, 128.44, 128.42, 128.40, 128.35, 128.26, 128.20, 128.16, 128.13, 128.08, 128.05, 128.04, 127.96, 127.94, 127.91, 127.85, 127.80, 127.76, 127.60, 127.55, 127.19, 127.15, 126.71, 126.63, 126.48, 126.05, 126.02, 125.98, 125.89, 125.83, 100.96, 100.70, 100.66, 99.99, 99.94, 99.29, 99.22, 99.00, 98.90, 80.19, 79.83, 79.18, 78.02, 77.91, 77.37, 76.02, 75.71, 75.35, 75.28, 75.19, 75.09, 74.92, 74.89, 74.44, 74.12, 73.88, 73.84, 72.37, 71.16, 70.82, 70.67, 70.63, 69.88, 69.74, 67.64, 67.58, 67.38, 67.34, 67.26, 67.21, 66.93, 62.64, 62.53, 61.56, 59.52, 59.40, 59.17, 55.90, 53.99, 50.60, 50.32, 47.22, 46.26, 29.80, 29.23, 23.32, 17.20, 17.16, 17.13, 17.08, 17.00. **ESI HRMS**: $[M+2H]^+$ calculated for $C_{183}H_{187}Cl_9N_{22}O_{39}H_2$: 3639.06871 (1819.53435); found 1819.53301

Nonasaccharide-protected with ONap on L-Fuc, Bn on D-Man and *N*-TFA (39)

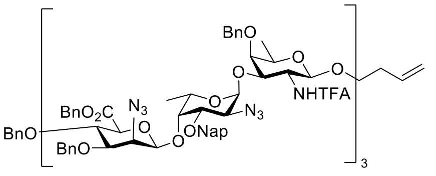

The imidate donor **27** (160 mg, 0.123 mmol) and acceptor **37** (170 mg, 0.082 mmol) were co-evaporated with toluene for three times. The residue was dissolved in dry DCM/MeCN (1.6 mL, 0.05 M). The reaction mixture was cooled to -78 °C and followed by adding TBSOTf (0.2 eq), and the reaction was allowed to stir for 30 min -78 °C until TLC analysis indicated the complete conversion of starting material. The reaction was quenched with Et_3N, diluted in DCM, washed with brine, and the organic phase was dried over Na_2SO_4, concentrated *in vacuo*. Purification by size-exclusion and column chromatography (silica gel, petroleum ether/acetone, 2/1) give the product **39** (172 mg, 65% yield). **^{1}H NMR (500 MHz, CDCl$_3$)** δ 7.85 – 7.68 (m, 11H), 7.54 – 7.39 (m, 8H), 7.38 – 7.11 (m, 44H), 7.11 – 6.98 (m, 8H), 6.85 (d, *J* = 7.5 Hz, 1H, NH), 6.44 (t, *J* = 7.9 Hz, 1H, 2xNH), 5.74 (ddt, *J* = 17.0, 10.3, 6.7 Hz, 1H, *CH*=CH$_2$), 5.08 – 4.97 (m, 2H, CH=*CH$_2$*), 4.90 – 4.82 (m, 5H, Ar*CH$_2$*, H_{1L}, $H_{1L'}$), 4.81 – 4.54 (m, 22H, Ar*CH$_2$*, $H_{1L''}$, H_{1D}, H_{1M}, $H_{1M'}$, $H_{1M''}$), 4.52 – 4.34 (m, 7H), 4.17 – 4.02 (m, 6H), 4.00 (d, *J* = 5.5 Hz, 2H, $H_{1D'}$, $H_{1D''}$), 4.02 – 3.81 (m, 12H), 3.80 – 3.62 (m, 7H), 3.58 (q, *J* = 6.5 Hz, 1H), 3.54 – 3.33 (m, 8H), 3.21 – 3.13 (m, 1H), 3.06 (t, *J* = 6.2 Hz, 1H), 2.29 (ddd, *J* = 13.1, 7.3, 4.1 Hz, 1H), 1.31 – 1.19 (m, 6H, 2xH_6), 1.15 – 0.97 (m, 12H, 4xH_6). **^{13}C NMR (126 MHz, CDCl$_3$)** δ 167.86 (C=O), 167.84 (C=O), 167.28 (C=O), 158.18 (CF_3C=O) , 157.89 (CF_3C=O), 157.60 (CF_3C=O), 157.30 (CF_3C=O), 138.54, 138.51, 138.49, 138.18, 138.16, 137.95, 137.39, 135.56, 135.48, 135.46, 135.10, 135.04, 134.91, 134.79, 133.38, 133.36, 133.06, 129.39, 128.77, 128.70, 128.62, 128.52, 128.50, 128.40, 128.36, 128.33, 128.22, 128.18, 128.14, 128.07, 127.94, 127.88, 127.81, 127.74, 127.66, 127.34, 127.12, 127.08, 126.70, 126.62, 126.09, 126.04, 125.99, 125.89, 125.81, 117.27 (CF_3), 116.98 (CF_3), 116.66 (CH=*CH$_2$*), 114.97 (CF_3), 114.69 (CF_3),

100.89 (C_{1M}), 100.67 ($C_{1M'}$), 100.64 ($C_{1M''}$), 100.54 (C_{1D}), 100.48 ($C_{1D'}$), 99.66 ($C_{1D''}$), 99.51 (C_{1L}), 99.24 ($C_{1L'}$), 99.21 ($C_{1L''}$), 80.95, 80.88, 79.83, 79.75, 79.29, 78.83, 76.21, 75.89, 75.45, 75.37, 75.31, 75.23 (Ar*C*H_2), 75.16 (Ar*C*H_2), 74.86 (Ar*C*H_2), 74.76, 74.65 (Ar*C*H_2), 74.57, 73.67 (Ar*C*H_2), 73.63 (Ar*C*H_2), 72.36 (Ar*C*H_2), 71.43 (Ar*C*H_2), 71.11 (Ar*C*H_2), 70.82 (O*C*H_2), 70.77, 70.61, 68.92 (Ar*C*H_2), 67.58, 67.50 (Ar*C*H_2), 67.47 (Ar*C*H_2), 67.37 (Ar*C*H_2), 67.10, 62.59 ($C_{1M''}$), 62.44 ($C_{1M'}$), 61.65 (C_{1M}), 59.82 ($C_{1L''}$), 59.71 ($C_{1L'}$), 59.57 (C_{1L}), 54.37 ($C_{1D''}$), 52.31 ($C_{1D'}$), 52.27 (C_{1D}), 33.87, 17.15 (2xC_6), 17.10 (C_6), 17.07 (C_6), 17.01 (2xC_6), 16.92 (3xC_6). **ESI HRMS:** $[(M + 2Na)]^+$ calculated for $C_{167}H_{170}F_9N_{21}O_{37}Na_2$, 2691.9106 (1639.0859); found 1639.0871.

CP5-*O*-Ac-Nonasaccharide (3)

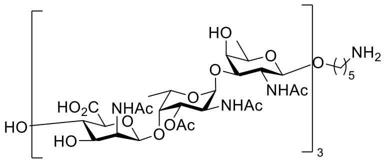

38 (91 mg, 0.025 mmol) was dissolved in DCM/H_2O (4:1, 2.5 mL, 0.01 M) and added DDQ (34 mg, 0.151 mmol, 6 equiv.). The reaction was stirred at rt under nitrogen for 6 h until TLC (pentane, EtOAc, 6.5:3.5) showed full conversion. The solution was quenched with $Na_2S_2O_3$ (sat. aq.) and extracted with EtOAc (x3). The combined organic phases were washed with $NaHCO_3$ (sat. aq.; x4) and brine (x1), dried over Na_2SO_4, filtrated and concentrated *in vacuo*. The crude was used without further purification. The residue was dissolved in pyridine (2 mL) and cooled to 0 °C and added Ac_2O (0.3 mL) and DMAP (catalytic amount) and stirred at rt under nitrogen overnight until TLC (pentane/acetone, 7:3) showed full conversion. The mixture was dissolved in EtOAc, washed with 1 M HCl (x1), $NaHCO_3$ (sat. aq.; x1) and brine (x1), dried over Na_2SO_4 and concentrated *in vacuo*. Column chromatography (pentane/acetone, 8:2 → 5:5) yielded the acetylated product in 74% yield (62 mg, 0.0186 mmol). The substrate (62 mg, 0.0186 mmol) was dissolved in THF (distilled, 3 mL) and added zinc powder (366 mg, 5.59 mmol, 300 equiv.), AcOH (1 mL) and Ac_2O (0.5 mL). The resulting mixture was stirred at 50 °C overnight until TLC (DCM/MeOH, 95:5) showed full conversion. The cooled mixture was filtered through Celite, evaporated *in vacuo* and co-evaporated with toluene (x3). The crude product was first purified by column chromatography (DCM/MeOH, 98:2 → 90:10) followed by HPLC given the acetamide product in 16% yield (9.6 mg, 0.0031 mmol). The substrate (9 mg, 0.00288 mmol) was dissolved in *t*-BuOH (1.5 mL) and added AcOH (1 mL, 0.1 mL in 100 mL MilliQ). Another 1 mL *t*-BuOH was added to dissolve the compound. The solution was birched with argon for 20 min and then $Pd(OH)_2/C$ (catalytic amount) was added. The reaction was again birched with argon for 5 minutes before the atmosphere was changed for H_2. The mixture was stirred for 3 days under H_2 atmosphere, after which it was filtered over a Whatman filter and lyophilized until NMR showed full conversion. Purification by a HW40 column with NH_4OAc followed by lyophilization gave **3** in 49% yield (2.8 mg, 0.0014 mmol). **^{1}H NMR (850 MHz, D_2O)** δ 5.18 (d, J = 17.4 Hz, 1H), 4.99 (td, J = 16.8, 14.2, 7.5 Hz, 5H), 4.72 (d, J = 28.1 Hz, 3H), 4.65 – 4.61 (m, 1H), 4.59 (d, J = 4.4 Hz, 1H), 4.46 – 4.31 (m, 5H), 4.24 – 4.09 (m, 7H), 4.09 – 3.92 (m, 6H), 3.92 – 3.67 (m, 17H), 3.66 – 3.55 (m, 5H), 2.99 (t, J = 7.8 Hz, 2H), 2.17 – 1.94 (m, 56H), 1.67 (q, J = 7.8 Hz, 2H), 1.58 (d, J = 8.7 Hz, 2H), 1.41 – 1.36 (m, 2H), 1.29 – 1.20 (m, 19H). **^{13}C NMR (214 MHz, D_2O)** δ 177.69, 175.21, 174.20, 173.67, 101.48, 101.37, 99.85, 99.80, 99.06, 98.97, 79.03, 78.30, 77.32, 77.00, 76.06, 75.57, 71.62, 70.71, 70.67, 70.36, 70.30, 70.11, 70.01, 69.86, 69.64, 69.38, 66.76, 52.98, 51.94, 51.27, 47.01, 39.29, 28.11, 26.35, 22.42, 22.10, 22.02, 21.94, 20.96, 20.28, 20.19, 15.26, 15.20, 15.13. **ESI HRMS**: $[M+2H]^+$ calculated for $C_{83}H_{130}N_{10}O_{46}H_2$: 2004.82189 (1002.410945); found 1002.41545

CP5-de-*O*-Ac-Nonasaccharide (4)

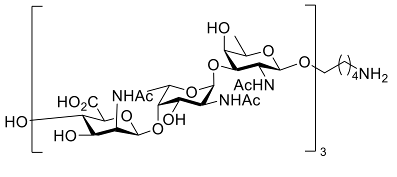

To a solution of **38** in 3 mL THF was added 1 mL AcOH and 0.5 mL Ac_2O, followed by addition of Zn dust (300 eq.). The resulting suspension was stirred at 50 °C until TLC analysis indicated the complete conversion of starting material (DCM/CH_3OH, 20/1). The reaction was filtered through a celite pad, concentrated *in vacuo*. Purification by silica gel column chromatography (DCM/CH_3OH, 50/1 to 20/1 to 10/1) delivered the compound as a white solid. The substrate

was dissolved in *t*BuOH/THF/H_2O (6 mL/1 mL/6 mL), 0.3 ml AcOH was added, followed by addition of $Pd(OH)_2$/C (200 mg). The reaction mixture was purged by hydrogen for 3 times and stirred under hydrogen atmosphere (hydrogen balloon) for 3 d until LC-MS indicated the complete conversion of starting material. Palladium was filtered through a celite pad, and the solution was concentrated *in vacuo* and purified by gel filtration (HW-40, 0.15M NH_4OAc in H_2O). The product containing fractions were pooled and lyophilized to yield **4** (9.3 mg, 60% yield over 2 steps). **^{1}H NMR (850 MHz, D_2O)** δ 5.00 – 4.88 (m, 6H, 6xH_1), 4.67 (dd, *J* = 4.6, 4.6 Hz, 3H, 3xH_2), 4.46 – 4.38 (m, 3H, 3xH_1), 4.31 – 4.24 (m, 1H), 4.20 – 4.03 (m, 11H), 4.01 – 3.90 (m, 9H), 3.90 – 3.69 (m, 20H), 3.66 – 3.52 (m, 3H), 2.99 (t, *J* = 7.7 Hz, 2H), 2.12 (s, 3H), 2.11 (s, 3H), 2.11 (s, 3H), 2.04 (s, 3H), 2.04 (s, 3H), 2.03 (s, 3H), 1.99 (s, 3H), 1.99 (s, 3H), 1.99 (s, 3H), 1.74 – 1.65 (m, 2H), 1.62 – 1.54 (m, 2H), 1.43 – 1.36 (m, 2H), 1.34 – 1.17 (m, 20H). **^{13}C NMR (214 MHz, D_2O)** δ 176.54, 176.43, 175.33, 175.29, 175.27, 175.25, 174.95, 102.43 (C_1), 102.12 (C_1), 102.11 (C_1), 100.33 (C_1), 100.30 (C_1), 100.13 (C_1), 100.10 (C_1), 100.05 (C_1), 99.89 (C_1), 80.46, 80.41, 80.31, 78.86, 78.83, 77.80, 77.67, 77.62, 76.83, 72.56, 71.65, 71.60, 71.59, 71.34, 71.24, 71.19, 71.17, 70.95, 69.67, 68.13, 68.10, 67.76, 67.69, 67.64, 53.73, 52.81, 52.80, 52.21, 52.10, 51.02, 50.95, 40.22, 29.04, 27.28, 23.30, 23.09, 23.08, 23.07, 23.06, 23.03, 22.89, 22.87, 22.86, 16.23, 16.22, 16.17. **ESI HRMS:** $[M+H]^+$ calculated for $C_{77}H_{121}N_{10}O_{43}$, 1877.7902; found 1877.7903.

CP5-Zwiterionic-Nonasaccharide (3)

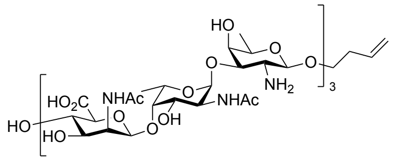

To a solution of compound **39** (50 mg, 0.0154 mmol) in 2 mL THF was added 1 mL methanol and 0.5 mL 1 M NaOH solution at 0 °C. The resulting mixture was stirred at rt for 48 h until TLC analysis indicated the complete conversion of carboxylate ester to carboxylic acid (petroleum ether/acetone, 2/1). The reaction was neutralized by addition of 1 M HCl, pH was adjusted to 6. Then the mixture was diluted in 30 mL EtOAc, washed with water, brine. The organic phase was dried with anhydrous Na_2SO_4, filtered and concentrated *in vacuo*. The carboxylic acid compounds were purified by silica gel column chromatography (DCM/CH_3OH, 50/1 – 10/1). The obtained carboxylic acid substrates were dissolved in a mixture of pyridine (3 mL) and H_2O (1 mL), followed by addition of triethylamine (40 eq/azide) and 1,3-propanedithiol (30 eq/azide) at room temperature under argon. The resulting mixture was shaded by aluminum foil and stirred for 24 h until TLC analysis indicated the complete conversion of azide to amine (EtOAc/CH_3OH/H_2O, 7/2/1). The solvents were evaporated *in vacuo*, the resulting residue was co-evaporated with toluene for 3 times. The given white solid was used for the next step without further purification. To a solution of amine substrates in THF (3 mL) and water (1 mL) was added $NaHCO_3$ (50 eq per amine), followed by addition of Ac_2O (30 eq per amine). The reaction mixture was stirred at room temperature for 24 h until TLC analysis indicated the complete conversion of starting material (DCM/CH_3OH, 10/1). The reaction was diluted in DCM (30 mL), washed with 1M HCl solution, water, brine. The organic phase was dried with anhydrous Na_2SO_4, filtered and concentrated *in vacuo*. The amide compounds were purified by silica gel column chromatography (DCM/CH_3OH, 50/1 – 10/1, 0.5% Et_3N). Ammonia (20 ml) was condensed at -70 °C, the amide substrates were dissolved in THF (2 ml) and tert-butanol (1 ml) and slowly added to reaction flask containing ammonia. Additive (allylcarbinol, 200 ul) was added to the reaction mixture. Small pieces of sodium were added to the reaction mixture to keep deep blue for 40 min. Then, ammonia acetate (100 mg) was added to the reaction mixture and the solution was allowed to reach room temperature and stirred until all of ammonia was evaporated. Then the solution was concentrated *in vacuo* and purified by gel filtration (HW-40, 0.15M NH_4OAc in H_2O). The product containing fractions were pooled and lyophilized to yield the final products as a white solid. The white solid was dissolved in 35% ammonia solution (5 mL), the flask was sealed, and the reaction mixture was stirred at 50 °C for 7 d until LC-MS indicated the complete conversion of starting material. The solution was concentrated *in vacuo* and purified by gel filtration (HW-40, 0.15M NH_4OAc in H_2O). The product containing fractions were pooled and lyophilized to yield the final product **5** (10.0 mg, 38% yield over 5 steps). **^{1}H NMR (850 MHz, D_2O)** δ 5.84 (ddt, *J* = 17.1, 10.3, 6.7 Hz, 1H), 5.16 – 5.07 (m, 1H), 4.99 – 4.94 (m, 3H, 3xH_1), 4.90 (s, 2H, 2xH_1), 4.86 (d, *J* = 1.5 Hz, 2H, 2xH_1), 4.67 – 4.63 (m, 3H, 2xH_1, H_2), 4.61 – 4.57 (m, 2H, H_2), 4.29 (q, *J* = 6.6 Hz, 3H), 4.17 (tt, *J* = 8.6, 3.0 Hz, 3H), 4.06 (d, *J* = 3.1 Hz, 5H), 4.00 – 3.84 (m, 13H), 3.83 – 3.66 (m, 11H), 3.65 – 3.51 (m, 3H), 3.28 (t, *J* = 9.7 Hz, 3H), 2.40 – 2.34 (m, 2H), 2.08 (s, 3H), 2.08 (s, 3H), 2.08 (s, 3H), 2.02 (s, 3H), 2.02 (s, 3H), 2.01 (s, 3H), 1.26 – 1.21 (m, 9H), 1.21 – 1.16 (m, 9H)**. ^{13}C NMR (214 MHz, D_2O)** δ 175.82, 175.58,

175.55, 175.12, 174.73, 174.67, 174.33, 135.13, 116.89, 100.29, 100.27, 99.02, 98.69, 98.51, 98.49, 79.36, 79.20, 78.96, 77.12, 74.56, 71.65, 71.31, 71.12, 70.71, 69.86, 69.71, 69.14, 69.08, 67.89, 66.56, 66.50, 52.85, 52.13, 51.44, 51.28, 50.28, 50.22, 33.12, 21.92, 21.91, 15.31, 15.29, 15.24. **ESI HRMS:** $[M+H]^+$ calculated for $C_{70}H_{114}N_9O_{40}$, 1720.7147; found 1720.7150.

SPR experiments

Immobilization of CP5-biotin on SA sensor chip:

Biotinylated CP5 CPS with concentration of 20 µg/mL was immobilized on a streptavidin-coated sensor chip (GE Healthcare) through a streptavidin–biotin capture using 1 M NaCl, 50 mM NaOH buffer for surface activation, and 1 M NaCl, 50 mM NaOH, and 50% isopropanol buffer to deactivate remaining active groups on the chip surface and remove noncovalently bound ligand. Biotinylated CP5 CPS was used at 20 µg/mL, reaching an immobilized surface density of 311.7 resonance units.

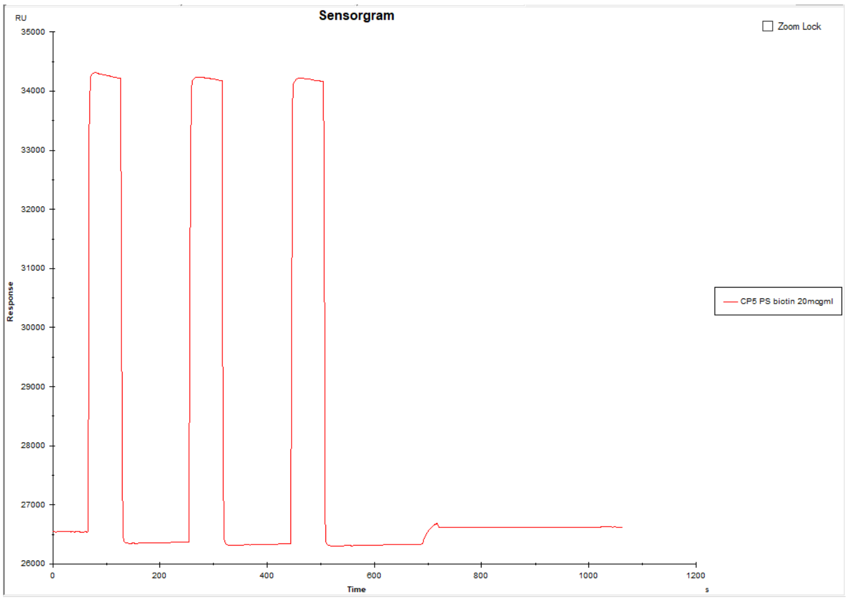

Figure S1. Sensorgram of CP5 CPS biotin immobilization on SA chip

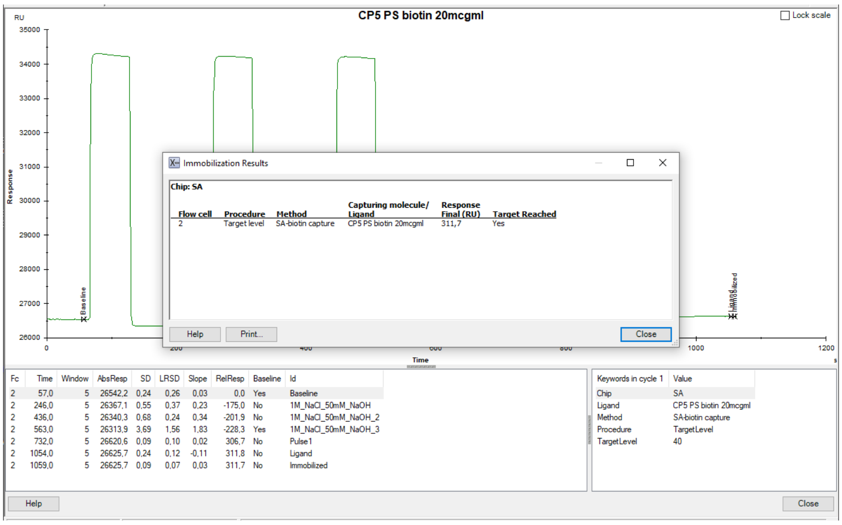

Figure S2. Immobilization level in terms of Resonance Unit response of CP5 CPS biotin

Binding analysis of CP5 oligosaccharides:

Binding was determined by SPR using a BIACORE X100 system. Binding competition was performed by incubating each competitor with the anti CP5 rat mAb before injection. The anti-CP5 antibody (a functional anti-CP5 rat mAb) was generated in rats via hybridoma monoclonal antibody production following immunization with the conjugated polysaccharide 5. For each sample, the experiment was performed using a constant concentration of mAb and decreasing concentrations (2-fold dilutions) of competitor. The ability of the competitor to inhibit the mAb binding to immobilized CP5 CPS is expressed as a percentage or reduction of the binding level compared to not-competitive mAb. The calculation of IC50 values were performed with GraphPad Prism software using Kruskal-Wallis with Dunn's multiple comparisons.

	**Tri 1**	**Hexa 2**	**Nona 3**	**Nona deOAc 4**	**Nona NH_3^+ 5**	**CP5 PS**
**Competitor concentrations (µg/mL)**	1000	20	5	250	800	0.0781
	500	10	2.5	125	400	0.0391
	250	5	1.25	62.5	200	0.0195
	125	2.5	0.625	31.25	100	0.0977
	62.5	1.25	0.313	15.63	50	0.00488
	31.25	0.625	0.156	7.81	25	0.00244
	15.63	0.313	0.0781	3.91	12.5	0.00122
	7.81	0.156	0.0391	1.95	6.25	0.000610
	3.91	0.0781	0.0195	0.977	3.125	0.000305
	1.95	0.0391	0.00977	0.488	1.56	0.000153
	0.977	0.0195	0.00488	0.244	0.78	7.629E-05
	0.488	0.00977	0.00244	0.122	0.39	3.815E-05
	0	0	0	0	0	0

Table S2. Concentrations of oligo- and polysaccharide inhibitors used in the competitive SPR experiments

Figure S3. Sensorgrams of competitive SPR analyses:

1) Sensorgrams competitive SPR of **<u>Tri 1</u>** (in duplicate)

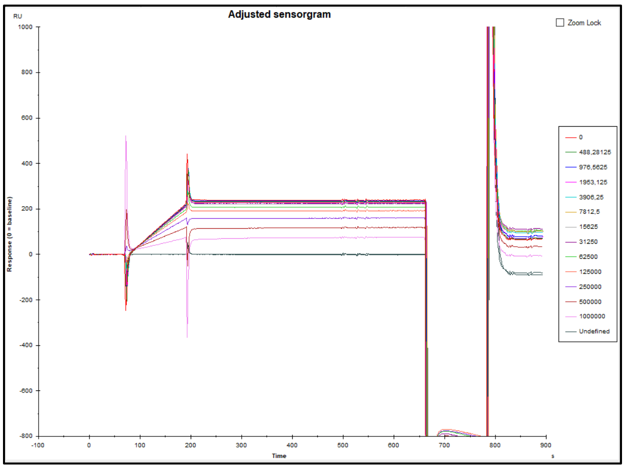

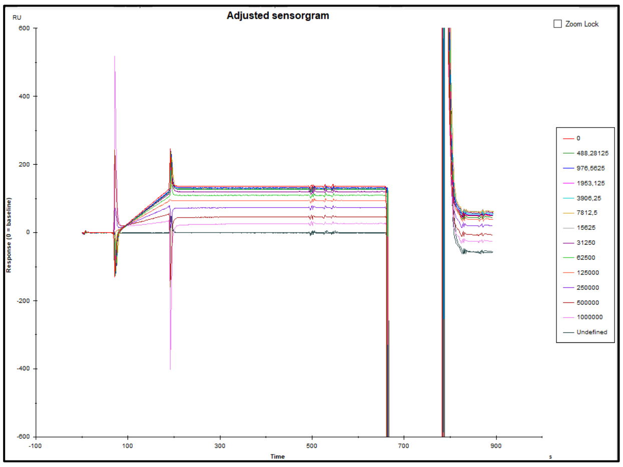

Binding levels:

**Conc. Tri 1 (µg/mL)**	**Binding level (RU) replicate 1**	**Binding level (RU) replicate 2**
1000	66,9	31,8
500	108,5	50,5
250	143,2	69,9
125	168,7	85,6
62.5	180,7	97,1
31.25	193,3	105,2
15.63	192,2	111,3
7.81	197,8	113,3
3.91	201,1	111,3
1.95	191,9	108,8
0.977	199,5	111,8
0.488	195,7	107,7
0	198,9	114,7

2) Sensorgrams competitive SPR of **<u>Hexa 2</u>** (in duplicate)

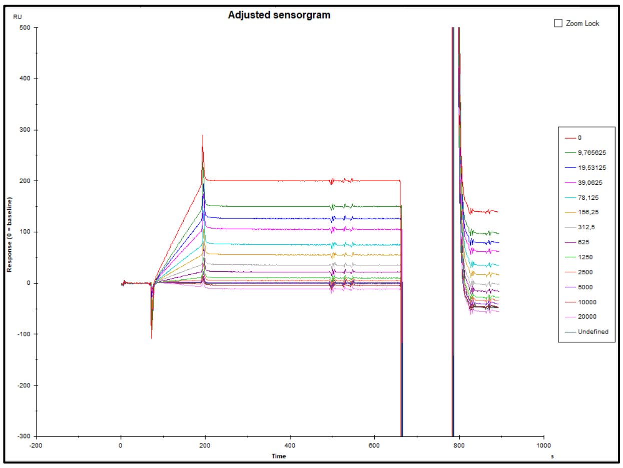

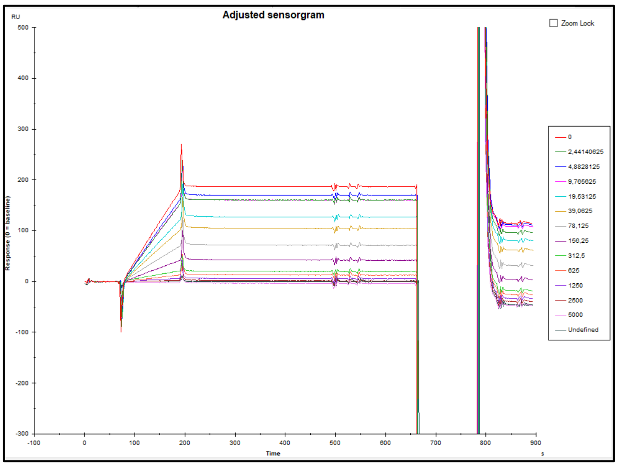

Binding levels:

**Conc. Hexa 2 (µg/mL)**	**Binding level (RU) replicate 1**	**Binding level (RU) replicate 2**
20	0	-
10	0	-
5	2	-2.9
2.5	5.6	2.2
1.25	10.0	5.8
0.625	20.5	12.2
0.313	33.4	19.2
0.156	50.6	38.5
0.0781	68.1	64.8
0.0391	93.7	93.4
0.0195	112.2	112.1
0.00977	131.4	140.7
0.00488	-	148.2
0.00244	-	139.6
0	173.9	162.5

3) Sensorgrams competitive SPR of **<u>Nona 3</u>** (in duplicate)

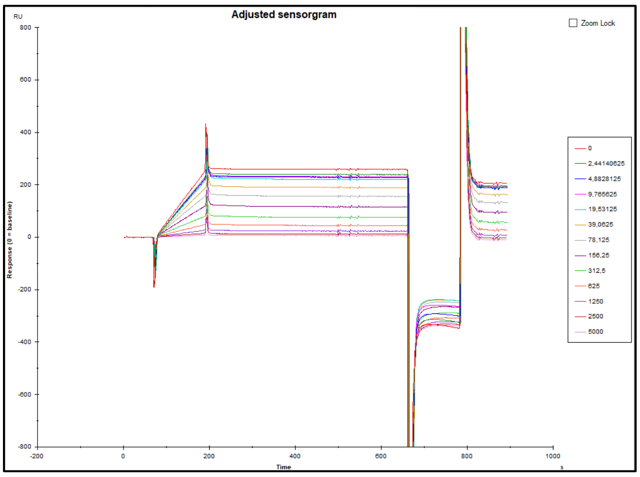

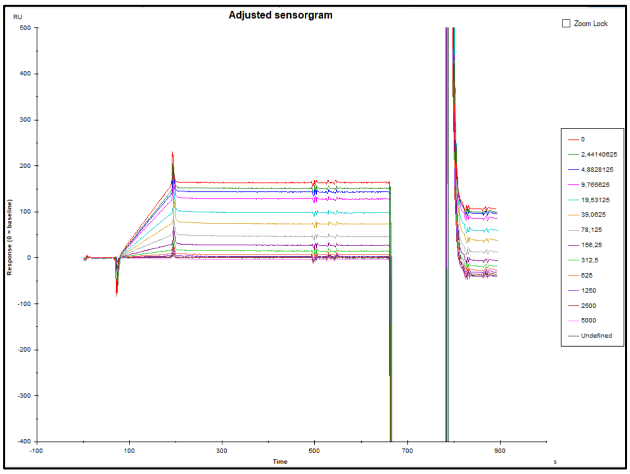

Binding levels:

**Conc. Nona 3 (µg/mL)**	**Binding level (RU) replicate 1**	**Binding level (RU) replicate 2**
5	6.8	0
2.5	14	1
1.25	24.8	4.2
0.625	44.9	7.7
0.313	73.8	15.2
0.156	109.2	27
0.0781	145.0	45.1
0.0391	173.5	68.7
0.0195	198.6	89.3
0.00977	206.3	114.5
0.00488	203.2	127.3
0.00244	210.3	132.9
0	226.4	141.9

4) Sensorgrams competitive SPR of <u>**Nona deOAc 4**</u> (in triplicate)

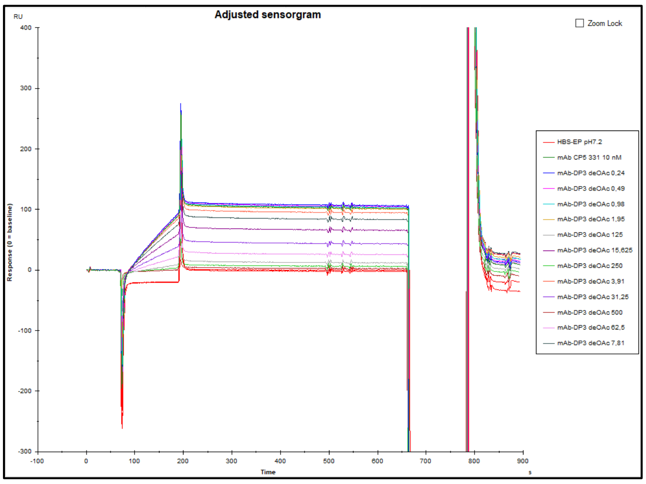

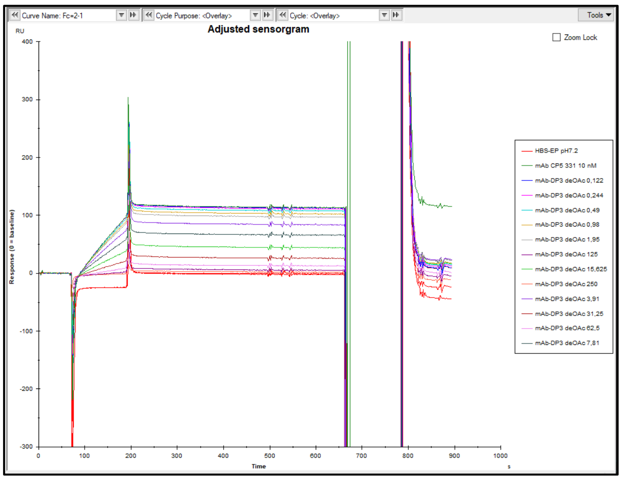

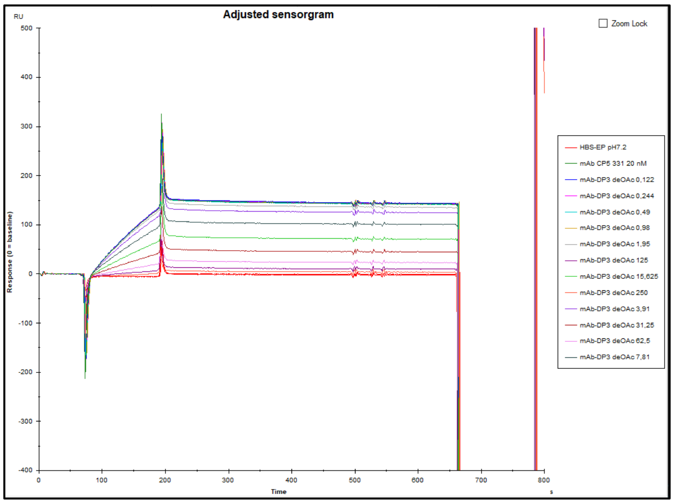

Binding levels:

**Conc. Nona deOAc 4 (µg/mL)**	**Binding level (RU) replicate 1**	**Binding level (RU) replicate 2**	**Binding level (RU) replicate 3**
250	0.1	-1.4	0.1
125	2.7	1.5	6.0
62.5	7.6	7.4	18.1
31.25	19.3	18.8	38.3
15.63	35.4	34.7	61.6
7.81	54.2	53.4	88.4
3.91	69.3	68.5	110.3
1.95	79.0	79.6	119.7
0.977	83.4	83.5	127.6
0.488	85.2	87.1	124.7

0.244	85.5	89.0	124.8
0.122	86.3	89.8	125.7
0	82.3	89.2	123.6

5) Sensorgrams competitive SPR of **<u>Nona NH_3^+ 5</u>** (in triplicate)

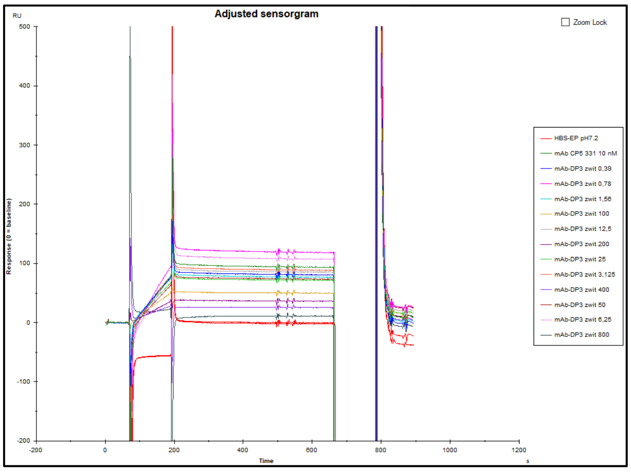

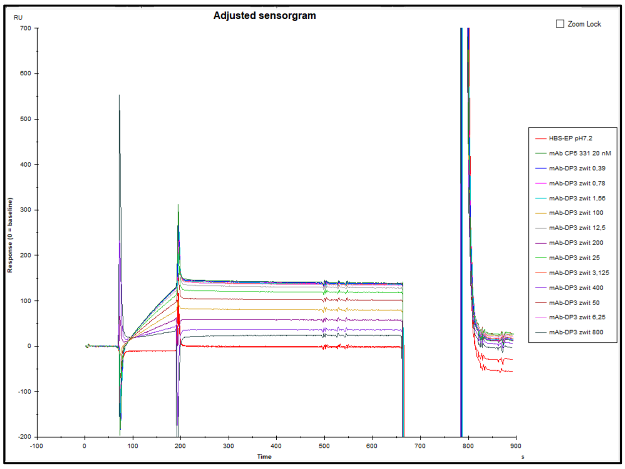

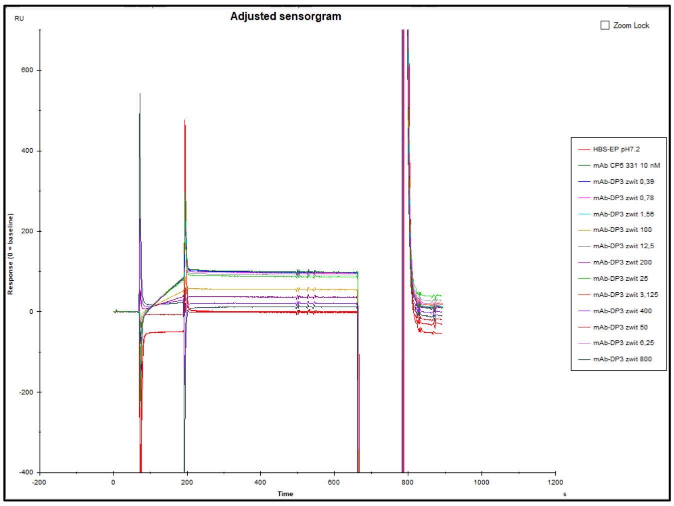

Binding levels:

**Conc. Nona NH_3^+ 5 (µg/mL)**	**Binding level (RU) replicate 1**	**Binding level (RU) replicate 2**	**Binding level (RU) replicate 3**
800	21.9	21.7	31.2
400	27.2	26.2	40.9
200	34.4	32.4	54.5
100	46.7	45.3	71.9
50	-6.8*	57.4	90.2
25	71.7	61.5	103.8

12.5	74.6	70.2	113.9
6.25	77.6	71.8	118.8
3.125	78.0	73.2	119.2
1.56	76.8	69.6	119.7
0.78	76.1	86.2	119.7
0.39	75.8	69.8	119.3
0	77	71.3	120.7

6) Sensorgram competitive SPR of <u>CP5 CPS</u>

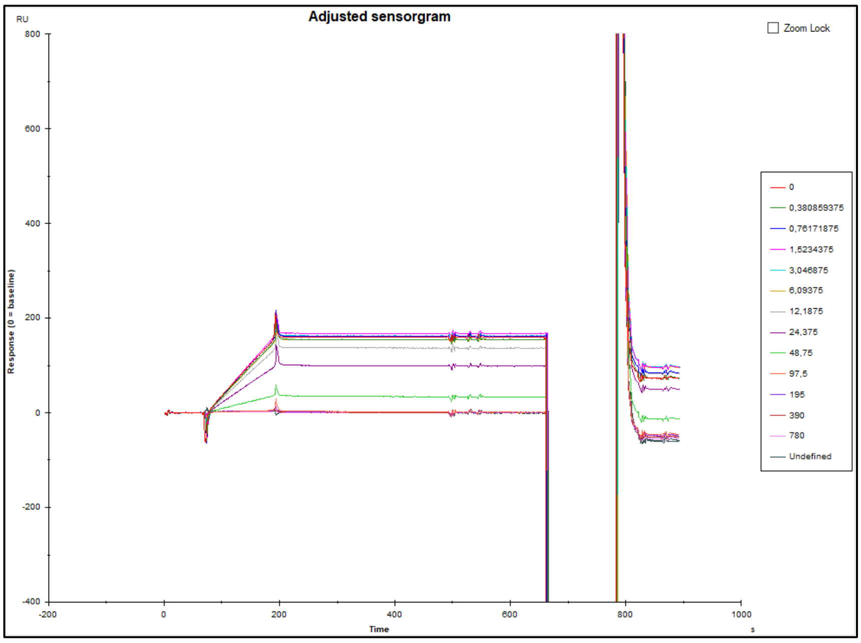

Binding levels:

**Conc. CP5 CPS**	**Binding level (RU) replicate 1**
<0.0781	2,5
0.0391	3,4
0.0195	4,4
0.0977	4,8
0.00488	33,3
0.00244	88,9
0.00122	122,2

0.000610	136,4
0.000305	144,1
0.000153	147
7.629E-05	142,1
3.815E-05	136,6
0	139,5

Structural conformation

Structure and conformational studies

NMR methods. NMR experiments were performed in a Bruker Avance III 800 MHz spectrometer equipped with a TCI cryoprobe. Samples were dissolved in D_2O at 1.0 mM concentration. Experiments were acquired at the temperature of 298 K.

^{1}H and ^{13}C NMR resonances of the molecules **1**, **2**, and **3** were assigned through standard 2D-TOCSY, 2D-ROESY, 2D-NOESY, 2D ^{1}H-^{13}C-HSQC. 2D-TOCSY experiments were acquired with 60 to 80 ms mixing time, 1.5 s of relaxation delay, 16 scans, and 2048x384 (F2xF1) points with a spectral width of ca. 10.0 ppm. 2D-ROESY experiment was acquired with mixing time of 200 ms, 1.5 s of relaxation delay, 16 to 24 scans, and 2048x512 (F2xF1) points with a spectral width of ca. 10.0 ppm. 2D-NOESY experiments were acquired with mixing times between 100 and 300 ms, 1.0 s of relaxation delay, 32 scans, and 4096x256 (F2xF1) points with a spectral width of ca. 10.0 ppm. 2D ^{1}H,^{13}C-HSQC experiments were acquired with 1.5 s of relaxation delay, 16 scans, and 2048x512 (F2xF1) points with a spectral width of ca. 12.0 ppm (F2) and 130.1 ppm (F1). The data were processed with Topspin 4.2 (Bruker Biospin) using a 90° shifted qsine window function to a total of 16K × 2K data points (F2 × F1), followed by automated baseline and phase correction.

NMR spectra and NMR signal assignment.

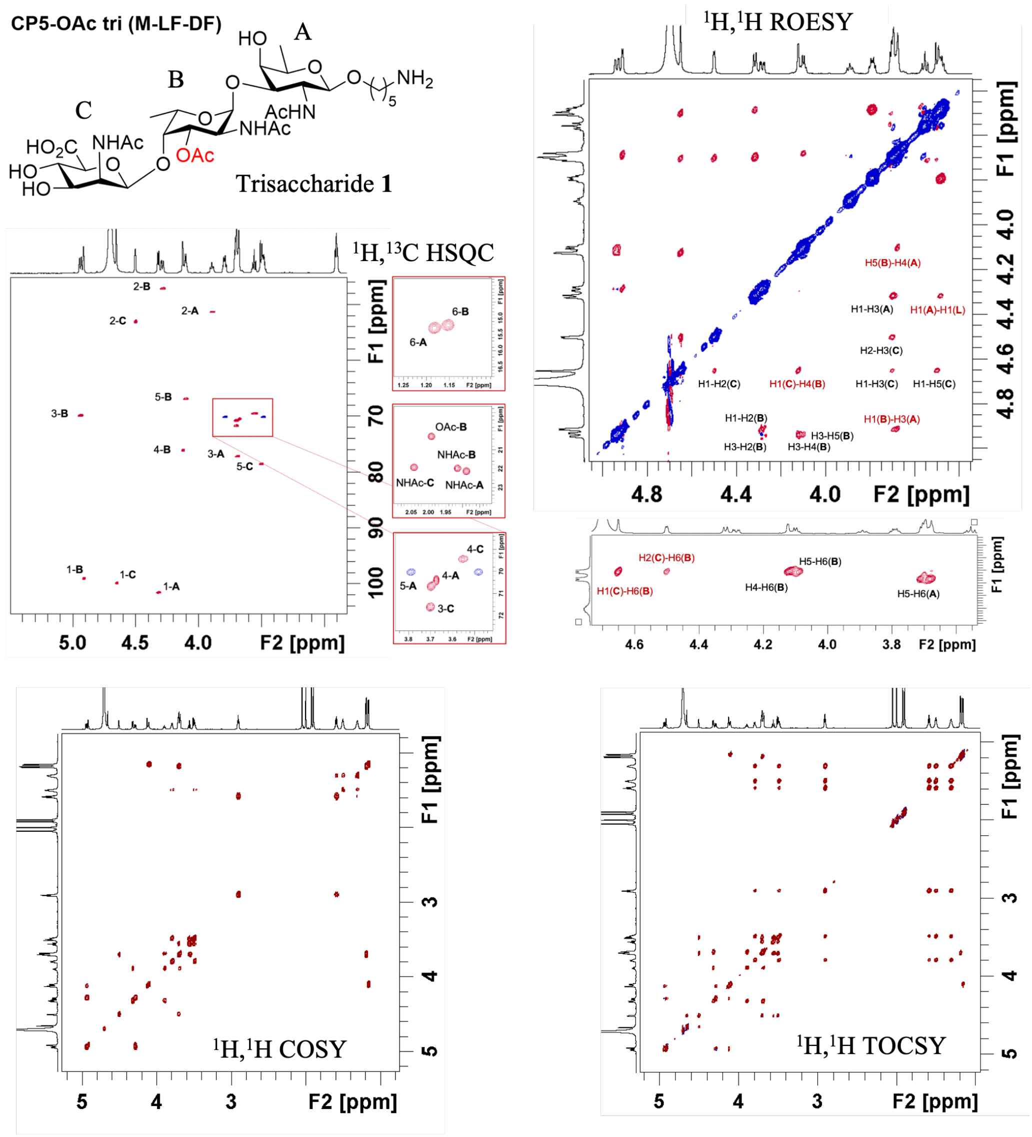

Figure S4: Trisaccharide 1 NMR characterization. The 1H and 13C NMR signals of the trisaccharide 1 were assigned through the combined use of herein reported 2D NMR spectra.

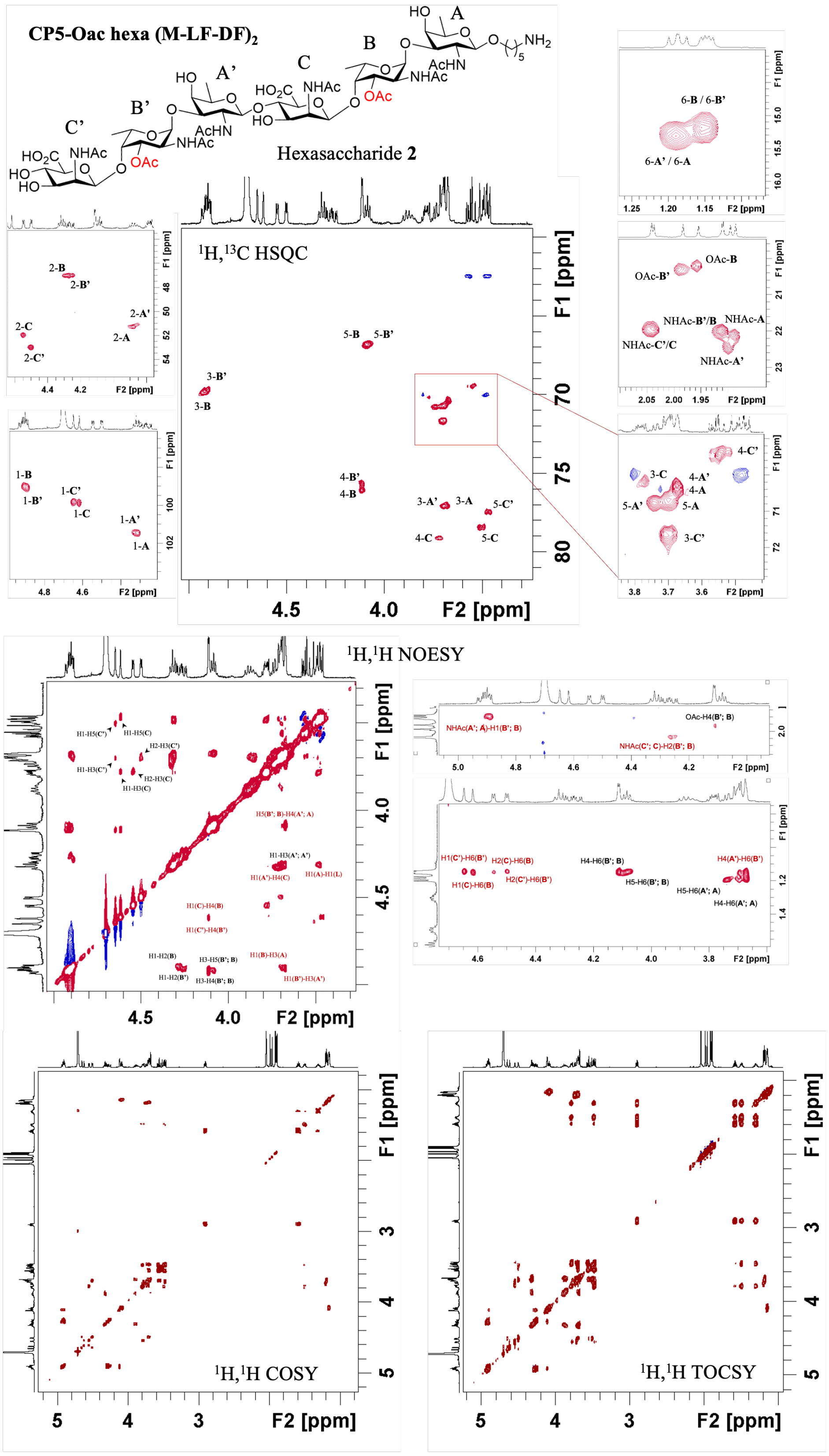

Figure S5: Hexasaccharide **2** NMR characterization. The ^{1}H and ^{13}C NMR signals of the hexasaccharide **2** were assigned through the combined use of herein reported 2D NMR spectra.

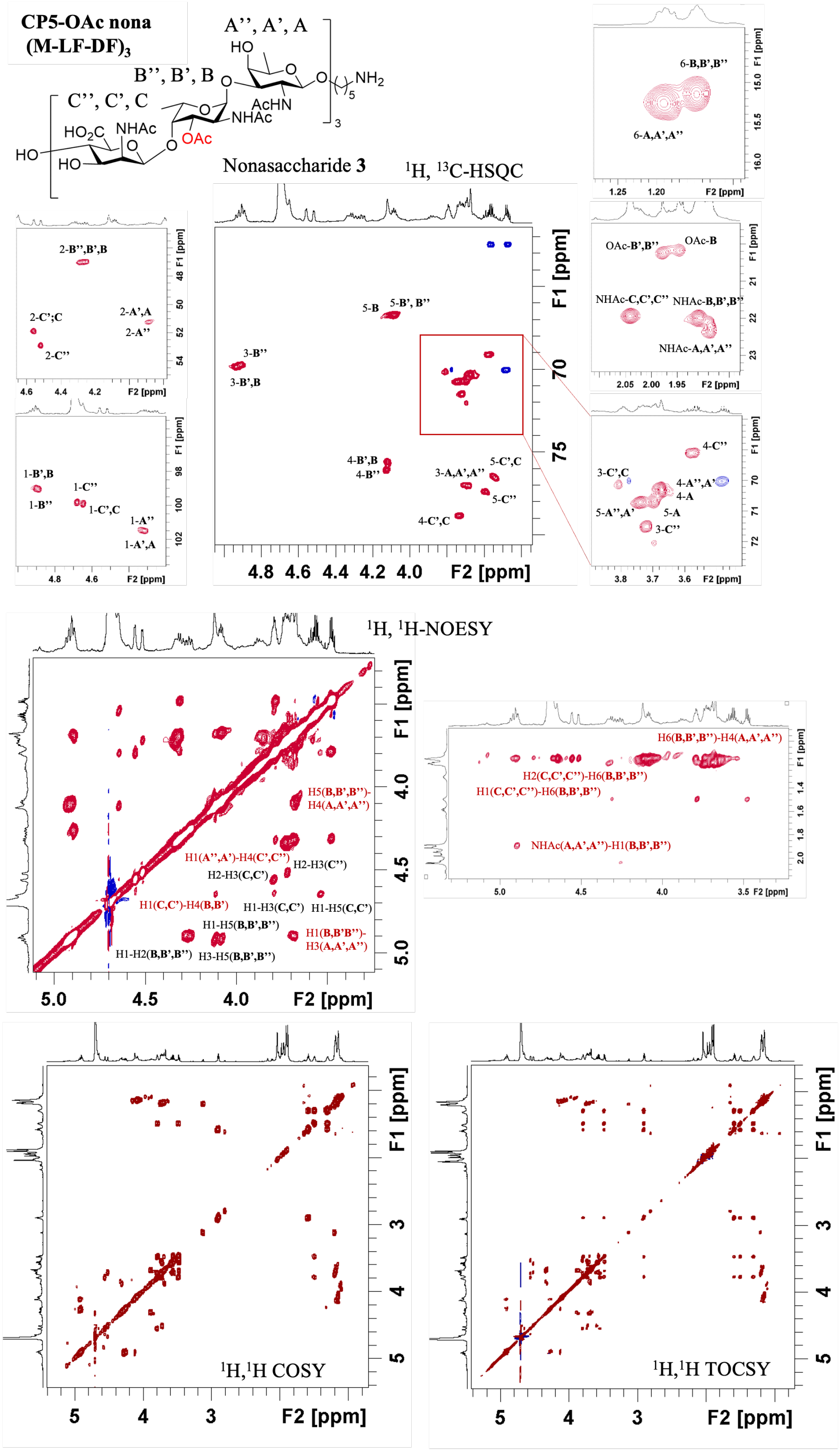

Figure S6: Nonasaccharide **3** NMR characterization. The ^{1}H and ^{13}C NMR signals of the nonasaccharide **3** were assigned through the combined use of herein reported 2D NMR spectra.

Molecular Mechanics Calculations. The geometry optimization was performed by using the Jaguar/Schroedinger package (version 13.5) and the AMBER* force field, with the GB/SA continuum solvent model for water. The glycosidic torsion angles were defined as ϕ (H1'-C1'-Ox-Cx) and ψ (C1'-Ox-Cx-Hx). Extended nonbonded cut-off distances (van der Waals cut-off of 8.0 Å and electrostatic cut-off of 20.0 Å) were used. The conformers for the tri-, the hexa-, and nona-saccharide molecules **1-3** were generated employing geometric restrictions to respect the exo-anomeric effect. The possible staggered rotamers around ψ were selected and minimized. The coordinates of the obtained local minima were employed to measure the key inter-proton distances that were then compared to those obtained experimentally by the ROESY and NOESY NMR experiments through integration of the observed NOEs cross peaks using the ISPA approximation. The resulting conformations and NOE distances analysis are reported in Table S1-S3 for the trisaccharide **1**.

Trisaccharide **1**

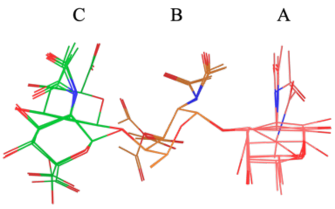

Table S1. Selected structures and derived Potential Energy values as function of the explored conformational space around the glycosidic linkages.

Structure	E (Kcal·mol^{-1})	ϕ C-B	ψ C-B	ϕ B-A	ψ B-A
1	0.00	+53	+7	+46	-15
2	0.55	+54	+6	+45	+1
3	1.03	+54	+6	+21	-57
4	2.27	+23	-50	+46	-15
5	2.15	+56	+7	+40	+30

Table S2. Analysis of the experimental NMR-NOEs data and comparison with those derived from the modeled conformations for the C-B glycosidic linkage. The main conformation has been determined by NOEs based calculated interatomic distances.

Atom Pair	Calculated Distance for selected conformer (Å)			Observed NOE intensity	Experimental Distance (Å)
	exo-*syn*-ϕ/*syn*(+)-ψ	exo-*syn*-ϕ/*syn*(-)-ψ	exo-*syn*-ϕ/anti-ψ		
H1(C)-H4(B)	2.7	2.4	3.5	strong	2.3
H1(C)-H6(B)	2.0	4.6	3.7-4.6	strong	2.6
H2(C)-H6(B)	2.8	4.3	5.3	medium/strong	2.8
H5(C)-OAc(B)	7.0-7.8	1.8-2.3	7.5	very weak	3.5
H1(C)-H2(B)	4.4	3.6	1.0	No	-
H2(C)-H2(B)	3.8	5.0	2.5	No	-
H3(C)-H2(B)	6.0	5.6	2.6	No	-
H5(C)-H1(B)	7.4	6.5	2.6	No	-

Table S3. Analysis of the experimental NMR-NOEs data and comparison with those derived from the modeled conformations for the B-A glycosidic linkage. The main conformation has been determined by NOEs based calculated interatomic distances.

Atom Pair	Calculated Distance for selected conformer (Å)			Observed NOE intensity	Experimental Distance (Å)
	exo-*syn*-ϕ/*syn*(+)-ψ	exo-*syn*-ϕ/*syn*(-)-ψ	exo-*syn*-ϕ/anti-ψ		
H1(B)-H3(A)	3.0	2.5	3.6	strong	2.2
H5(B)-H4(A)	3.2	2.9	5.0	medium/strong	2.5
H5(B)-H3(A)	2.8	4.0	3.6	medium/weak	2.8
H1(B)-H2(A)	2.9	4.6	2.7	No	-
H6(B)-H4(A)	4.7	2.7	6.5	No	-
H1(B)-H4(A)	4.5	2.5	2.8	No	-
H6(B)-H2(A)	6.2	4.7	2.6	No	-
H5(B)-H2(A)	5.0	3.4	2.8	No	-

Spectra

1,2,3,4-tetra-*O*-acetyl-D-fucopyranose (S1)

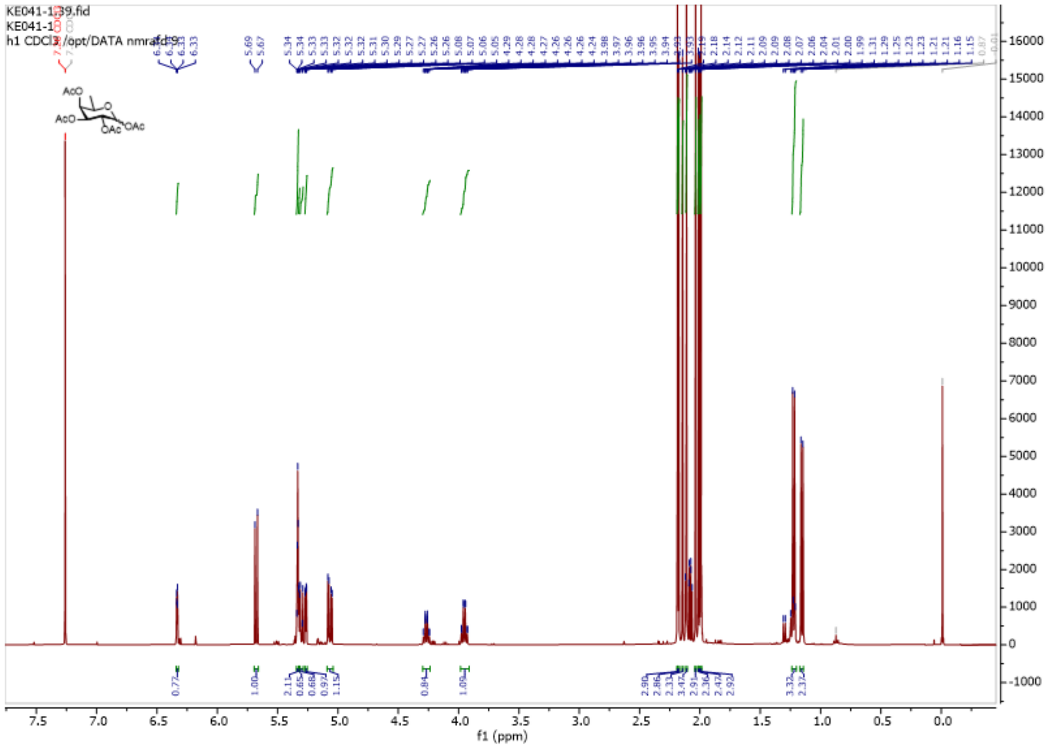

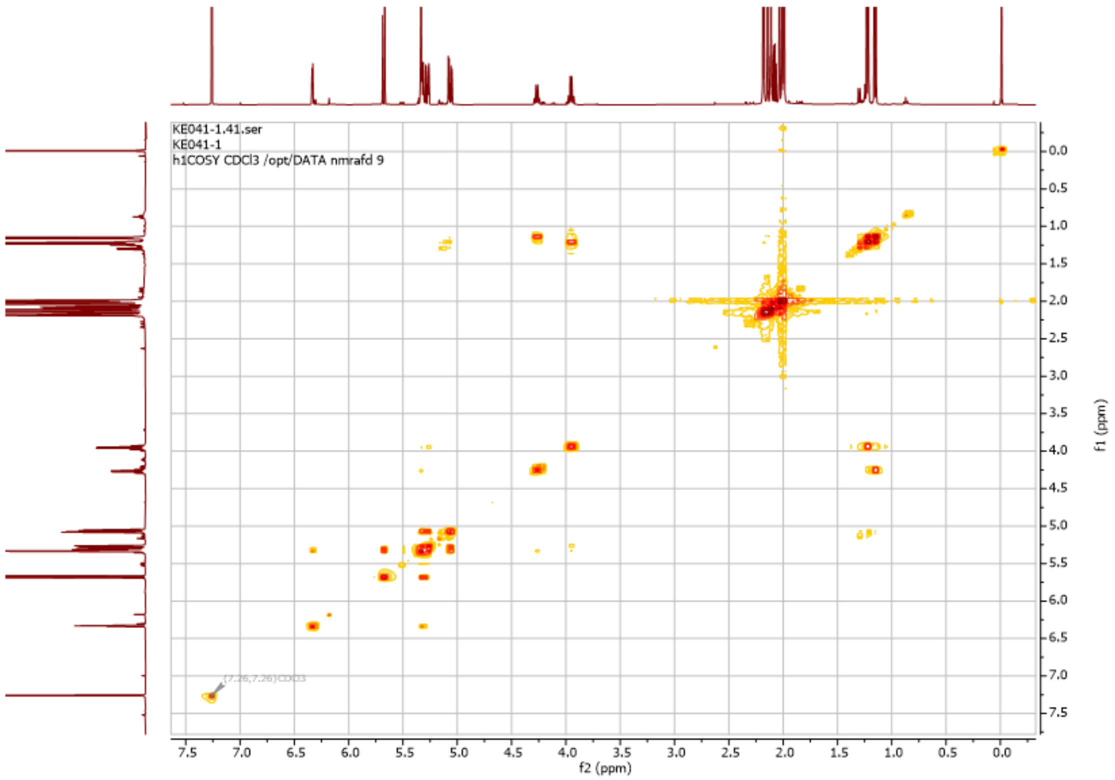

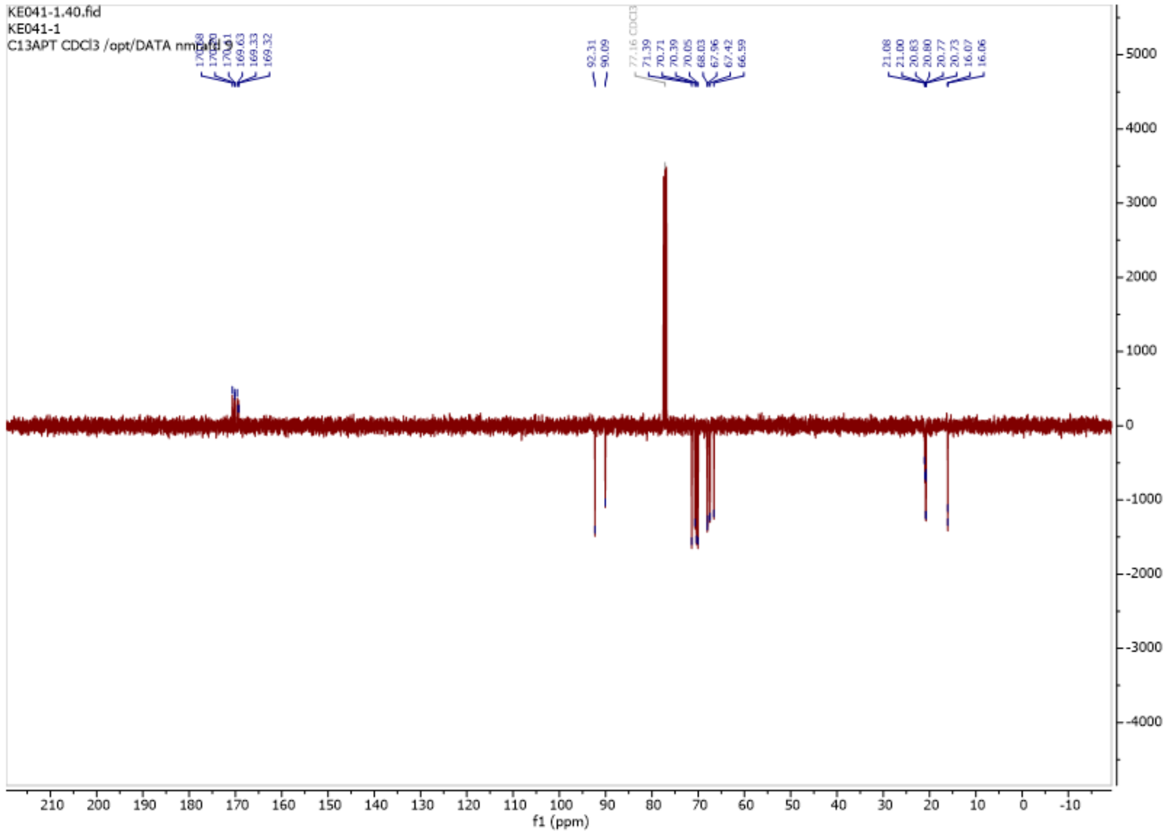
KE041-1.40.fid
KE041-1
C13APT CDCl3 /opt/DATA nmrafd 9
169.63
169.33
169.32
92.31
90.09
77.16 CDCl3
71.39
70.71
70.39
70.05
68.03
67.96
67.42
66.59
21.08
21.00
20.83
20.80
20.77
20.73
16.07
16.06
f1 (ppm)

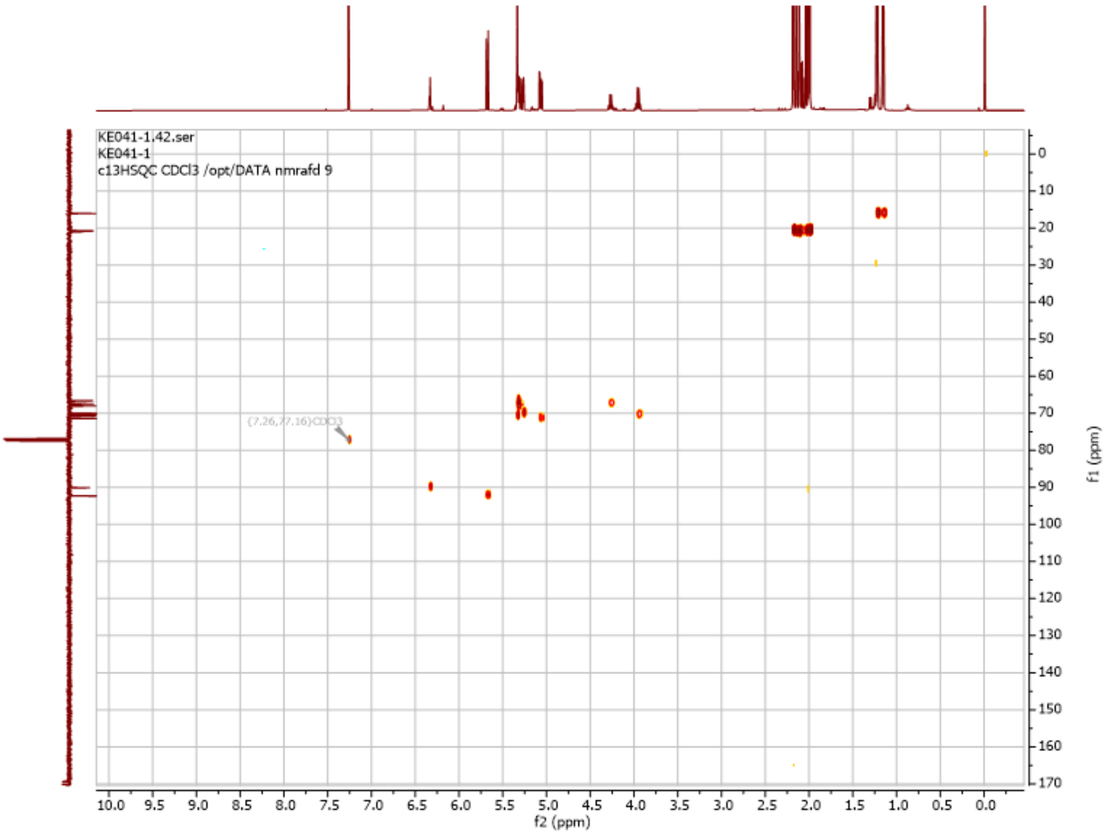
KE041-1.42.ser
KE041-1
c13HSQC CDCl3 /opt/DATA nmrafd 9
(7.26,77.16)CDCl3
f2 (ppm)
f1 (ppm)

3,4-di-*O*-acetyl-D-fucal (S2)

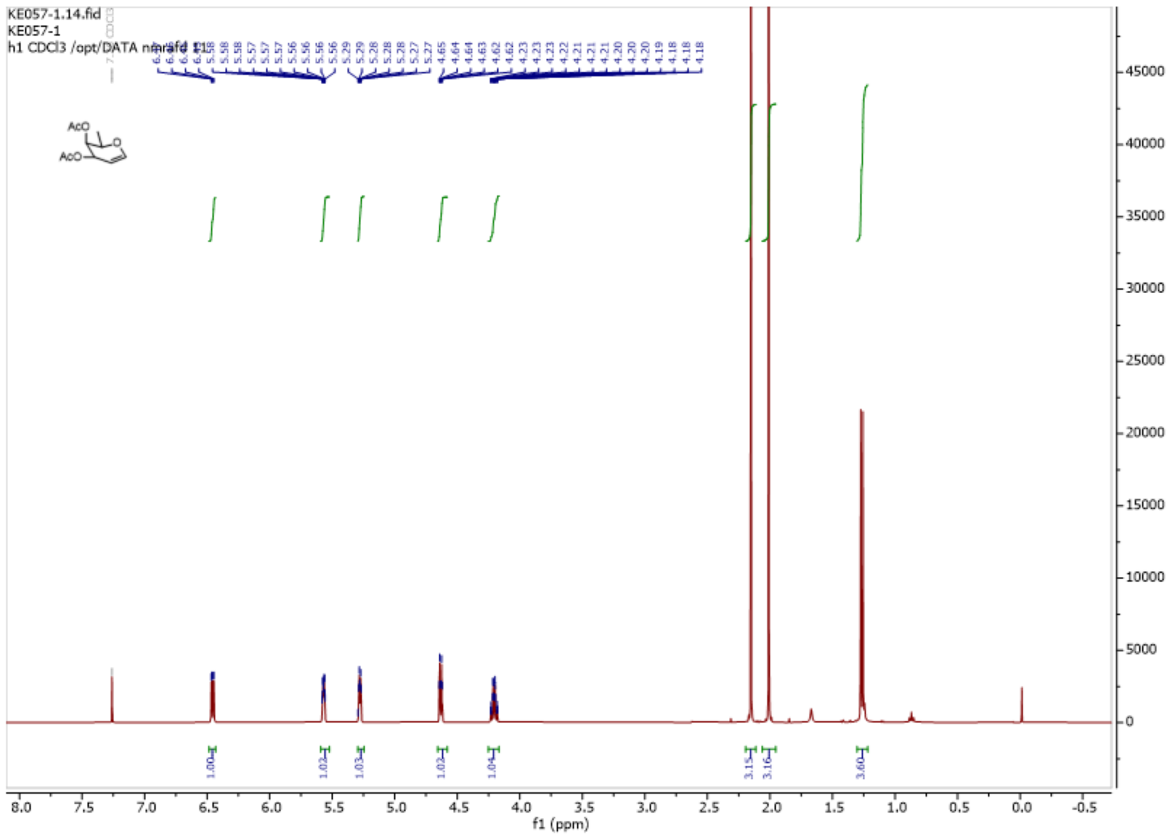

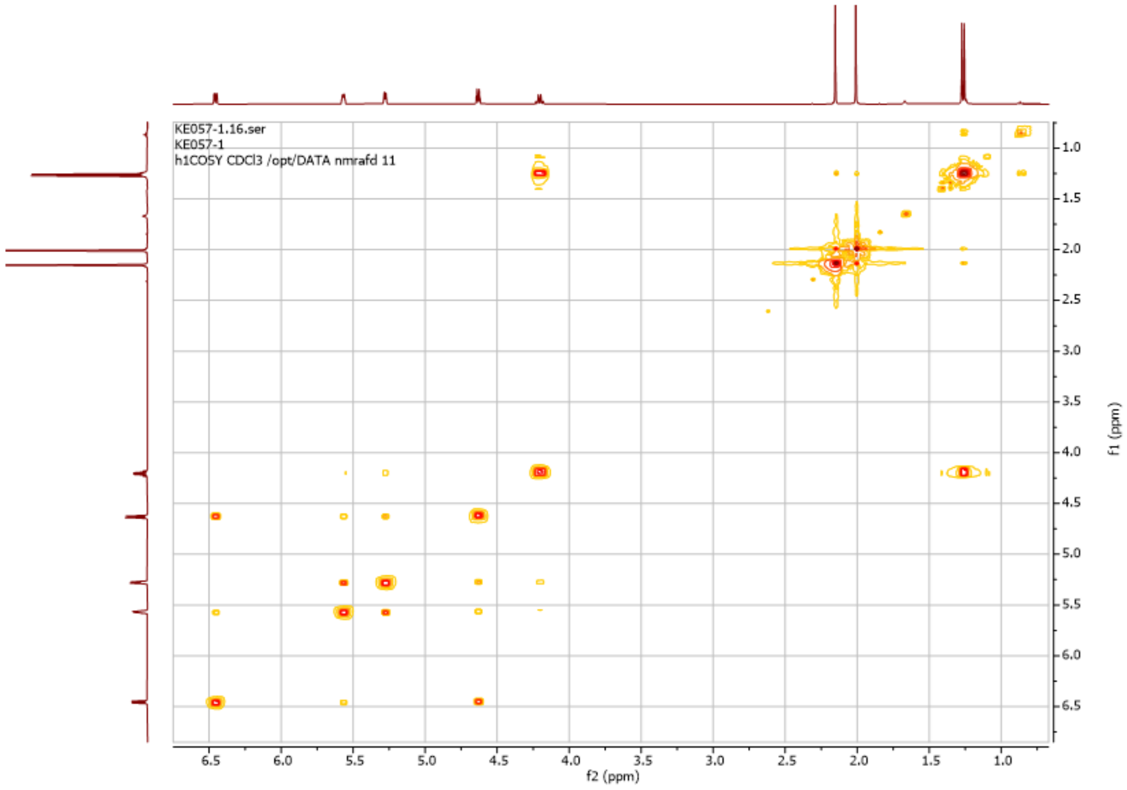
KE057-1.16.ser
KE057-1
h1COSY CDCl3 /opt/DATA nmrafd 11
f2 (ppm)
f1 (ppm)

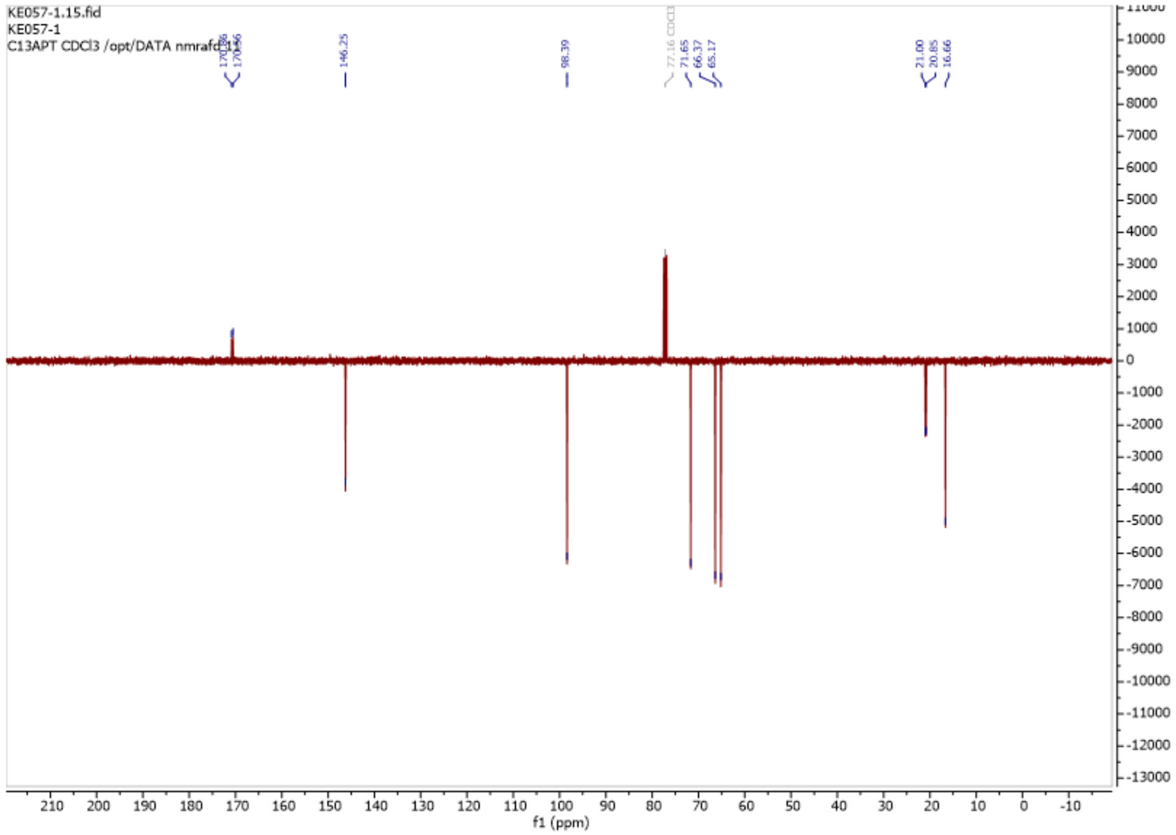
KE057-1.15.fid
KE057-1
C13APT CDCl3 /opt/DATA nmrafd 11
146.25
98.39
77.16 CDCl3
71.65
66.37
65.17
21.00
20.85
16.66
f1 (ppm)

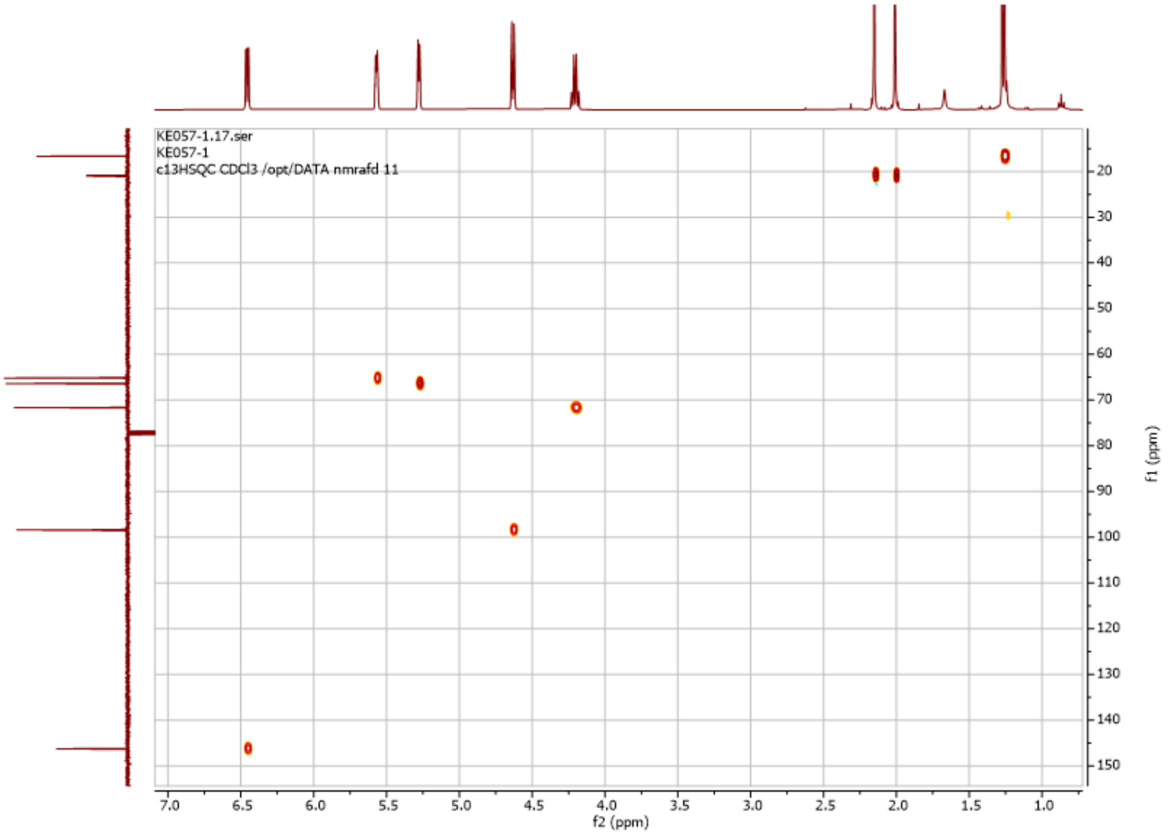

Phenyl 2-azido-2-deoxy-1-seleno-α-D-fucopyranoside (S3)

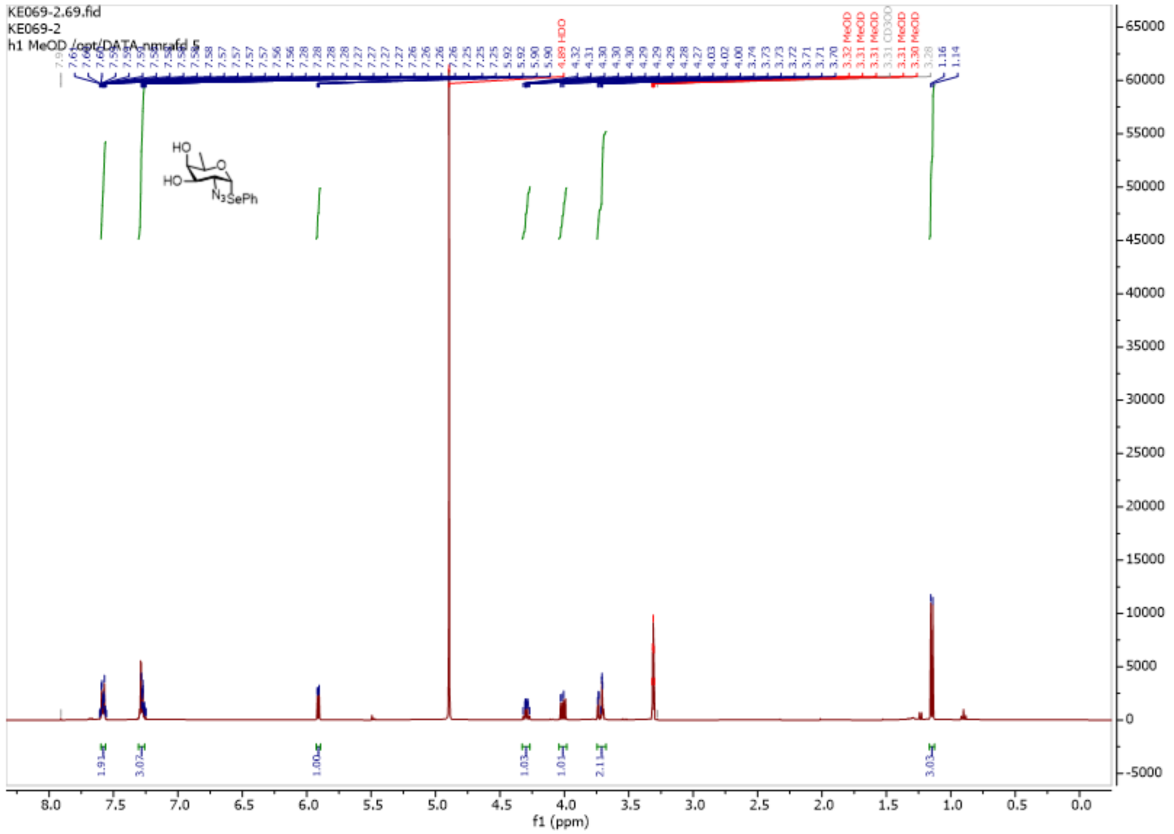
KE069-2.69.fid
KE069-2
h1 MeOD /opt/DATA nmrafd 5
HO
HO
O
N3
SePh
f1 (ppm)

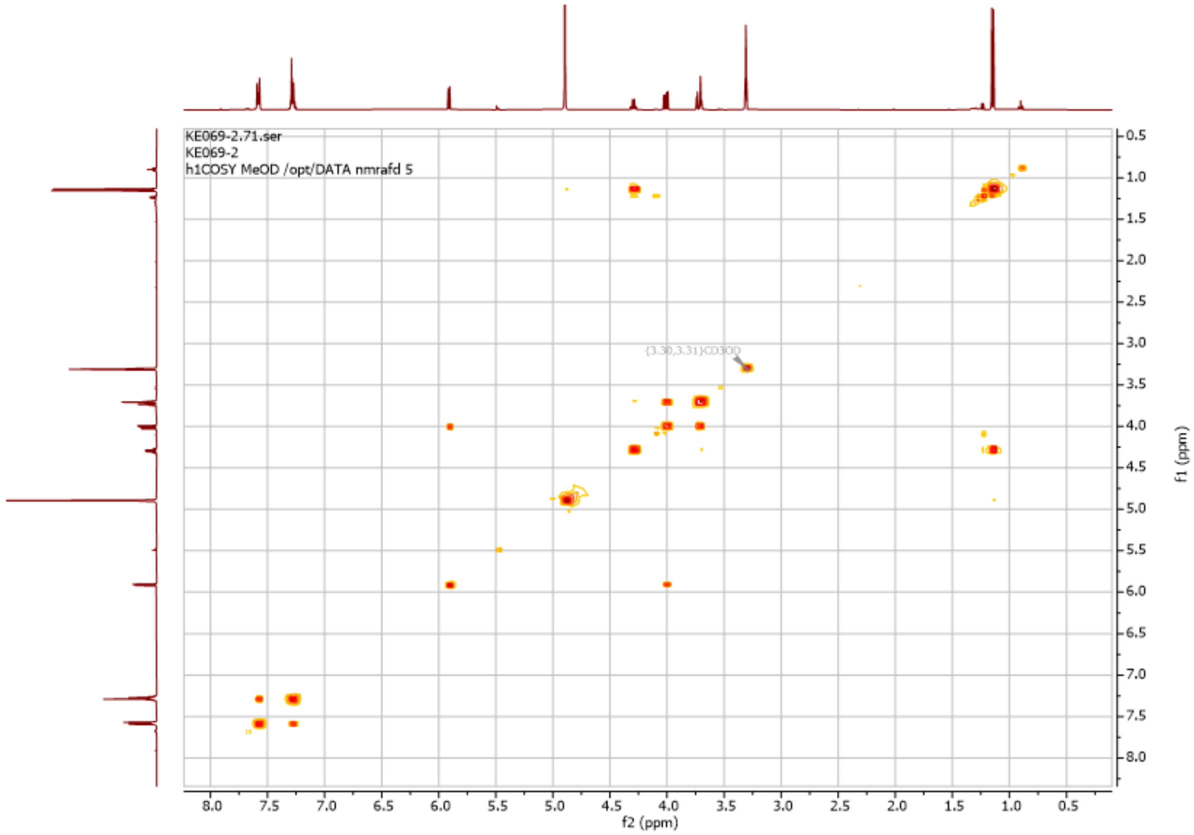
KE069-2.71.ser
KE069-2
h1COSY MeOD /opt/DATA nmrafd 5
f2 (ppm)
f1 (ppm)

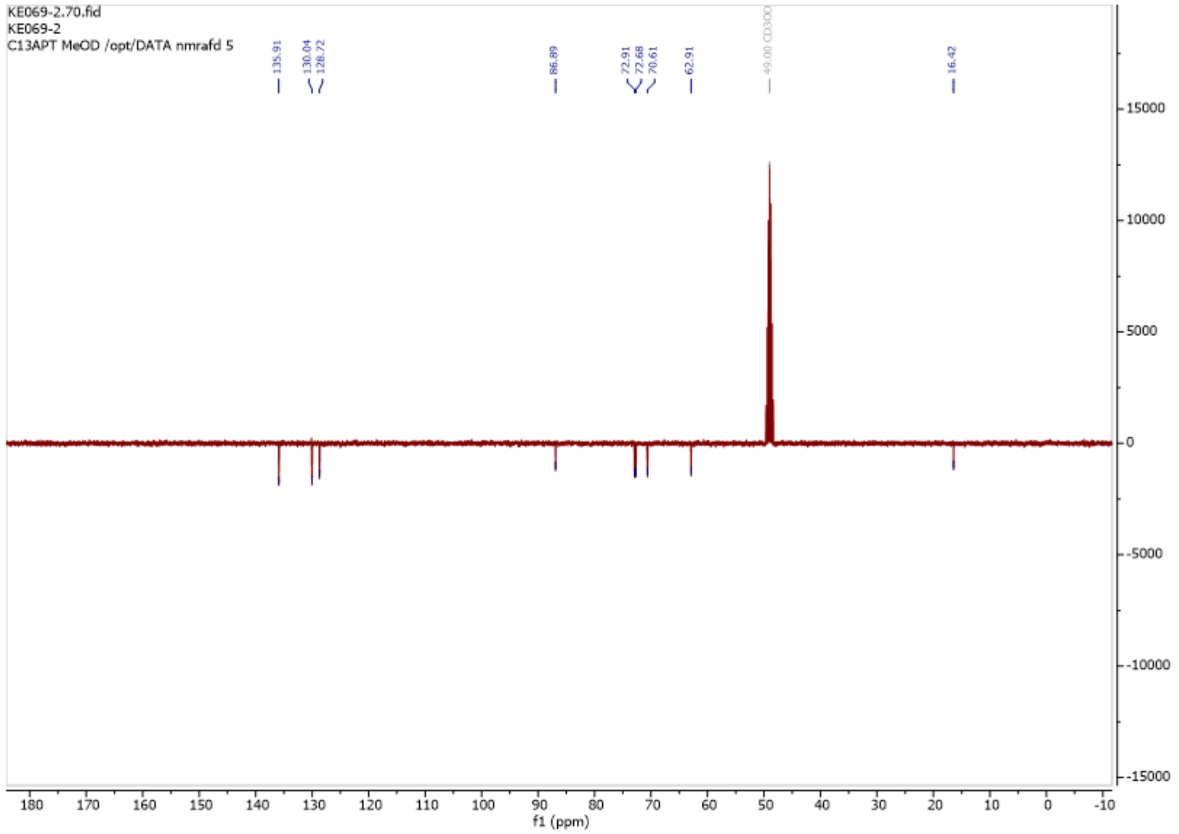
KE069-2.70.fid
KE069-2
C13APT MeOD /opt/DATA nmrafd 5
135.91
130.04
128.72
86.89
72.91
72.68
70.61
62.91
49.00 CD3OD
16.42
15000
10000
5000
0
-5000
-10000
-15000
180
170
160
150
140
130
120
110
100
90
80
70
60
50
40
30
20
10
0
-10
f1 (ppm)

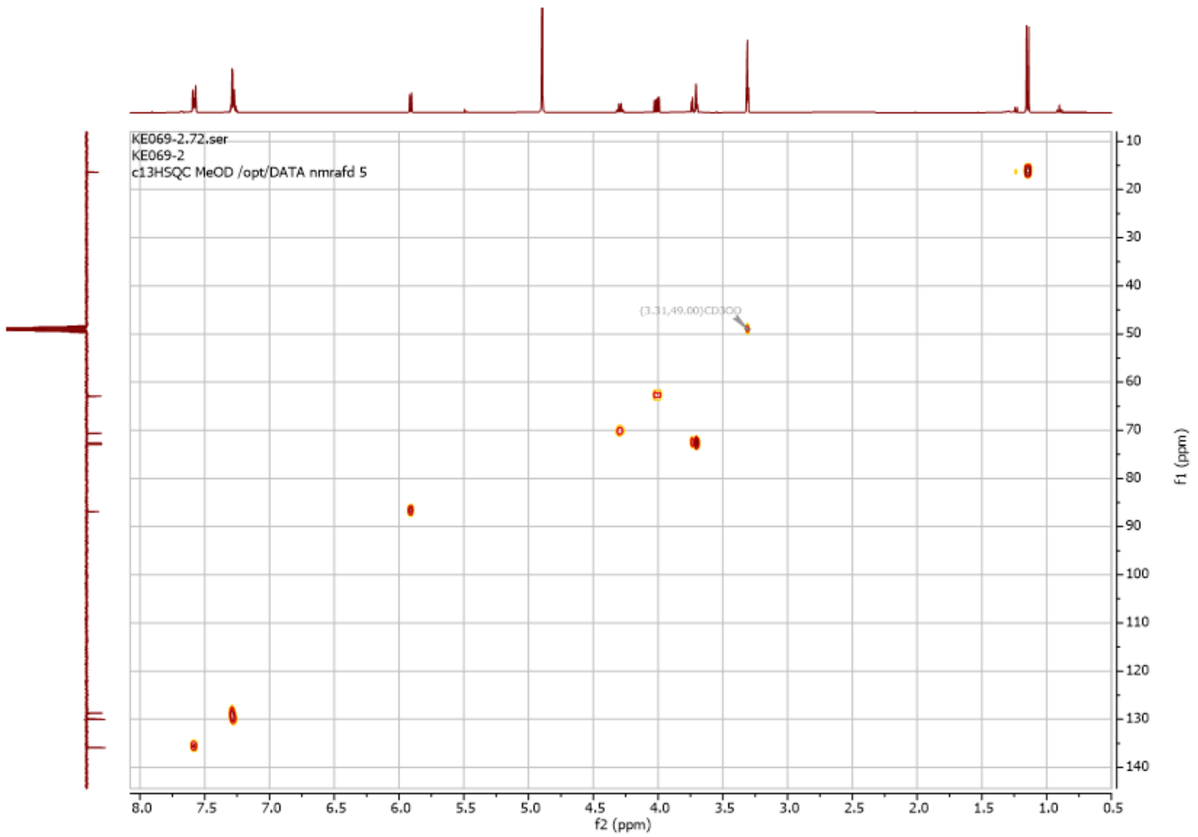
KE069-2.72.ser
KE069-2
c13HSQC MeOD /opt/DATA nmrafd 5
(3.31,49.00)CD3OD
10
20
30
40
50
60
70
80
90
100
110
120
130
140
f1 (ppm)
8.0
7.5
7.0
6.5
6.0
5.5
5.0
4.5
4.0
3.5
3.0
2.5
2.0
1.5
1.0
0.5
f2 (ppm)

Phenyl 2-azido-2-deoxy-3-*O*-(2-naphthylmethyl)-1-seleno-α-D-fucopyranoside (S4)

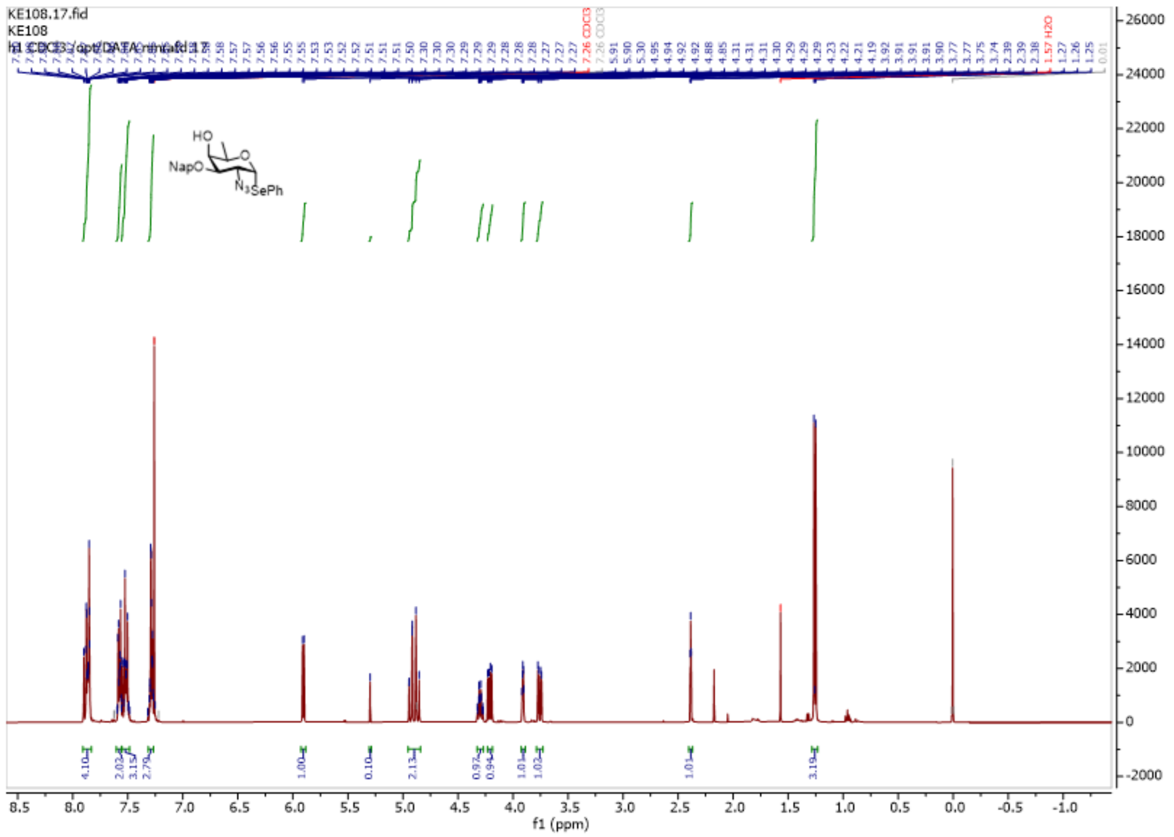

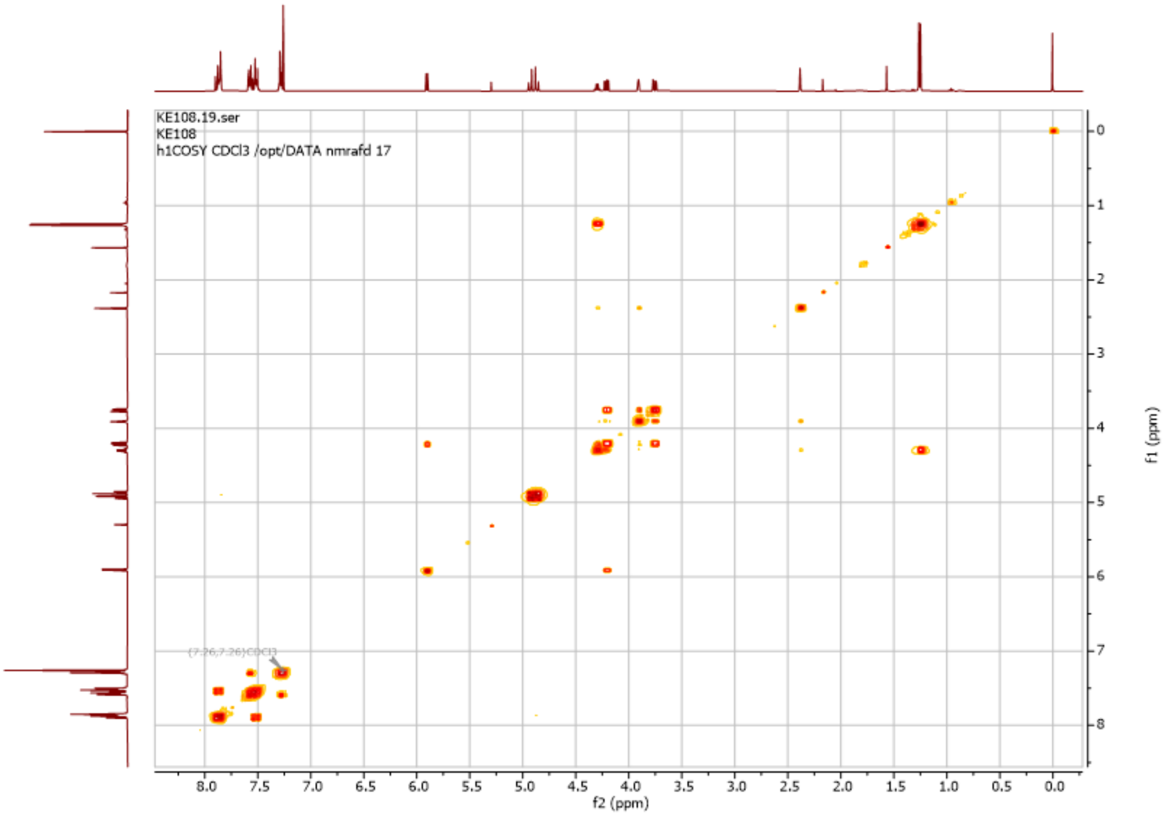
KE108.19.ser
KE108
h1COSY CDCl3 /opt/DATA nmrafd 17
f2 (ppm)
f1 (ppm)

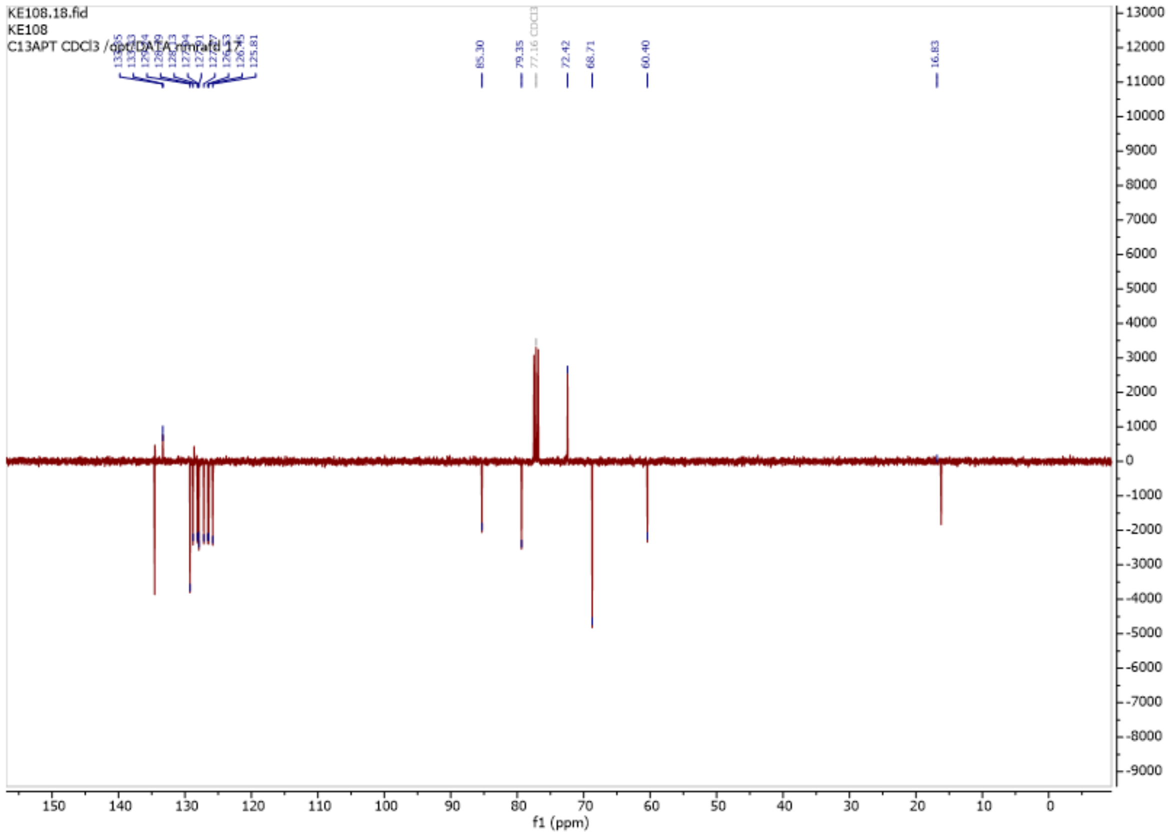
KE108.18.fid
KE108
C13APT CDCl3 /opt/DATA nmrafd 17
125.81
85.30
79.35
77.16 CDCl3
72.42
68.71
60.40
16.83
f1 (ppm)

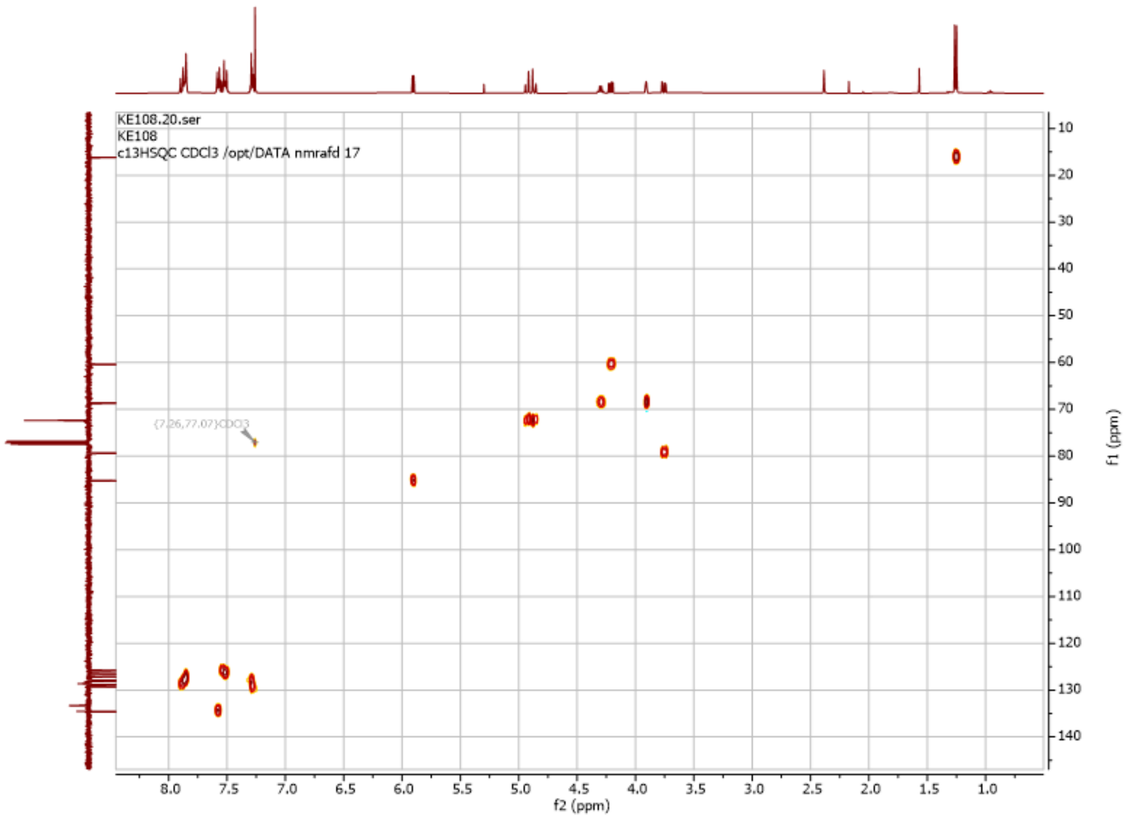
KE108.20.ser
KE108
c13HSQC CDCl3 /opt/DATA nmrafd 17
{7.26,77.07}CDCl3
f2 (ppm)
f1 (ppm)

Phenyl 2-azido-4-*O*-benzyl-2-deoxy-3-*O*-(2-naphthylmethyl)-1-seleno-α-D-fucopyranoside (S5)

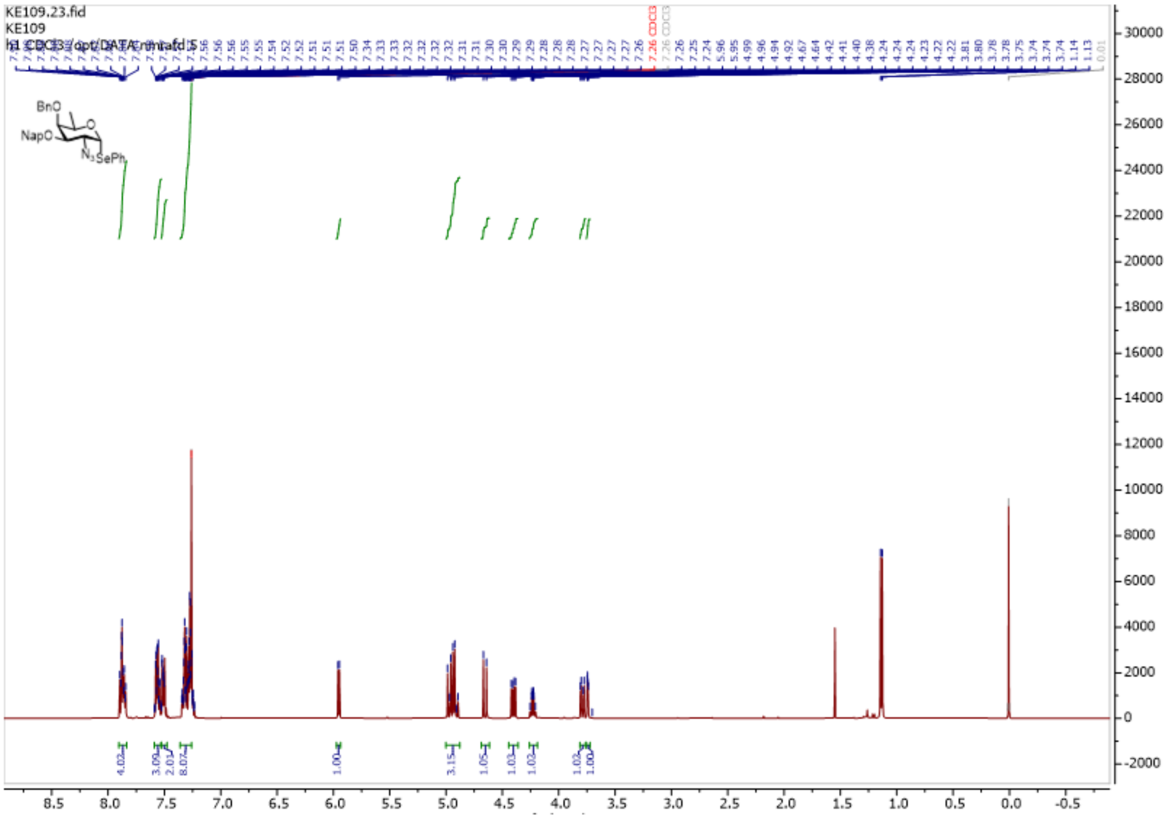

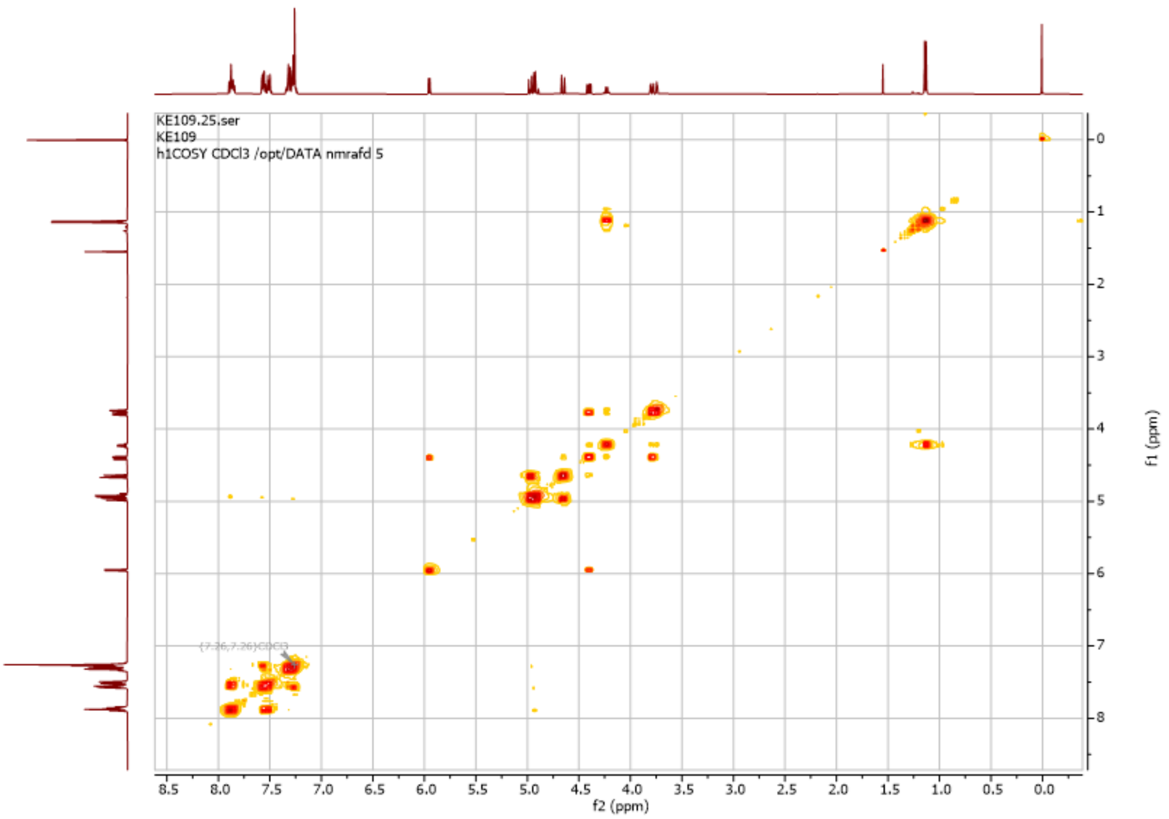
KE109.25.ser
KE109
h1COSY CDCl3 /opt/DATA nmrafd 5
f2 (ppm)
f1 (ppm)

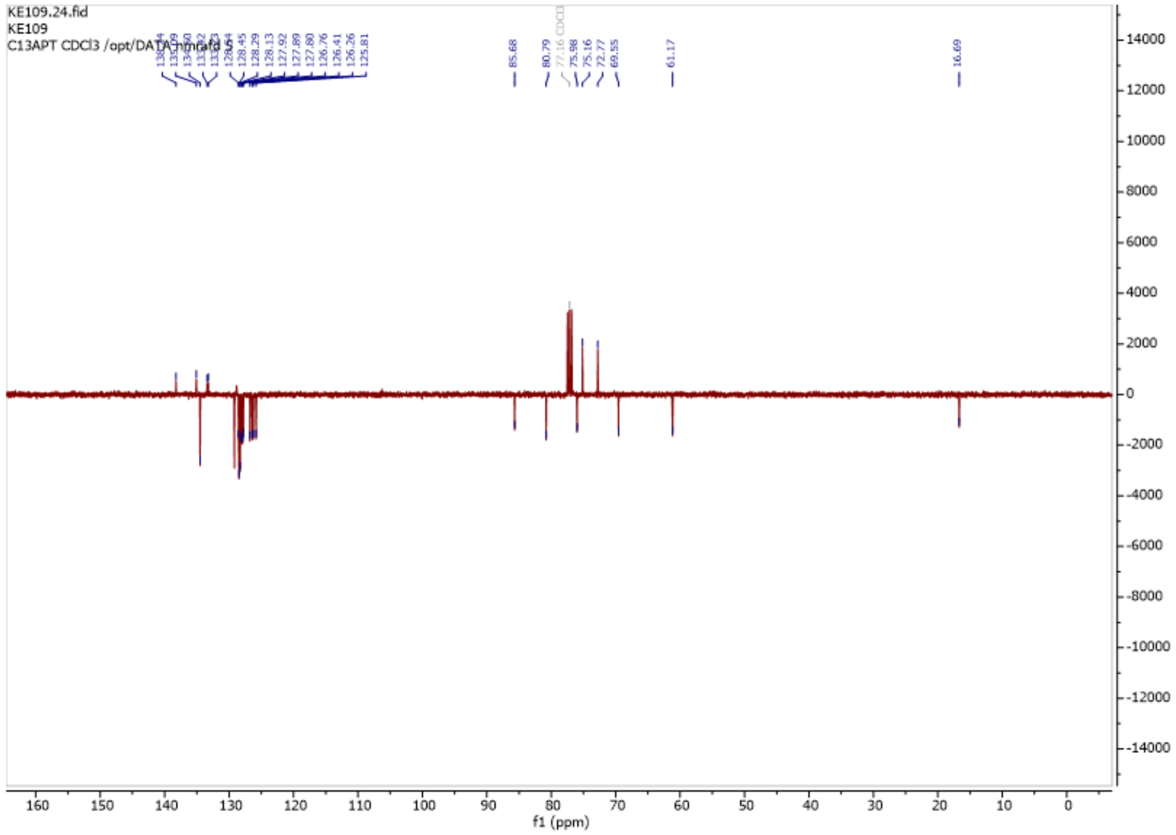
KE109.24.fid
KE109
C13APT CDCl3 /opt/DATA nmrafd 5
128.45
128.29
128.13
127.92
127.89
127.80
126.76
126.41
126.26
125.81
85.68
80.79
77.16 CDCl3
75.98
75.16
72.77
69.55
61.17
16.69
f1 (ppm)

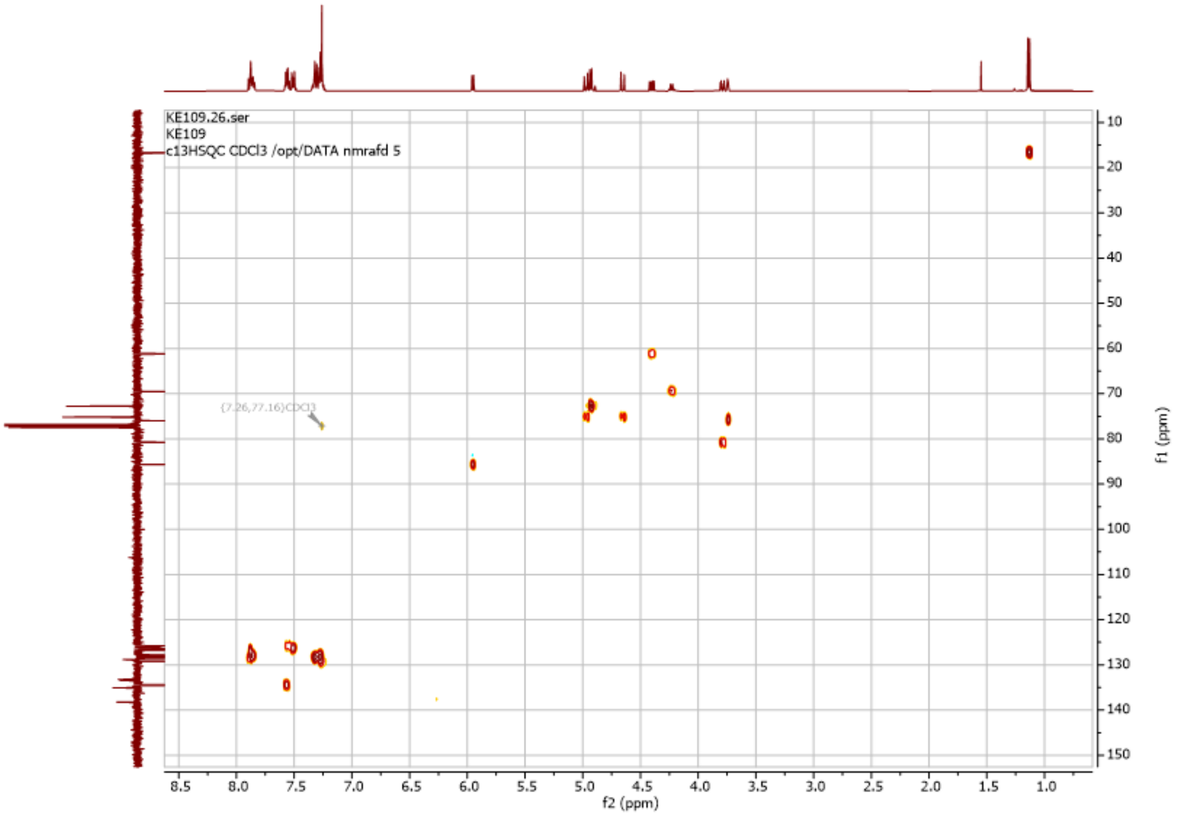

Phenyl 4-*O*-benzyl-2-deoxy-3-*O*-(2-naphthylmethyl)-2-*N*-trichloroacetamide-1-seleno-α-D-fucopyranoside (S6)

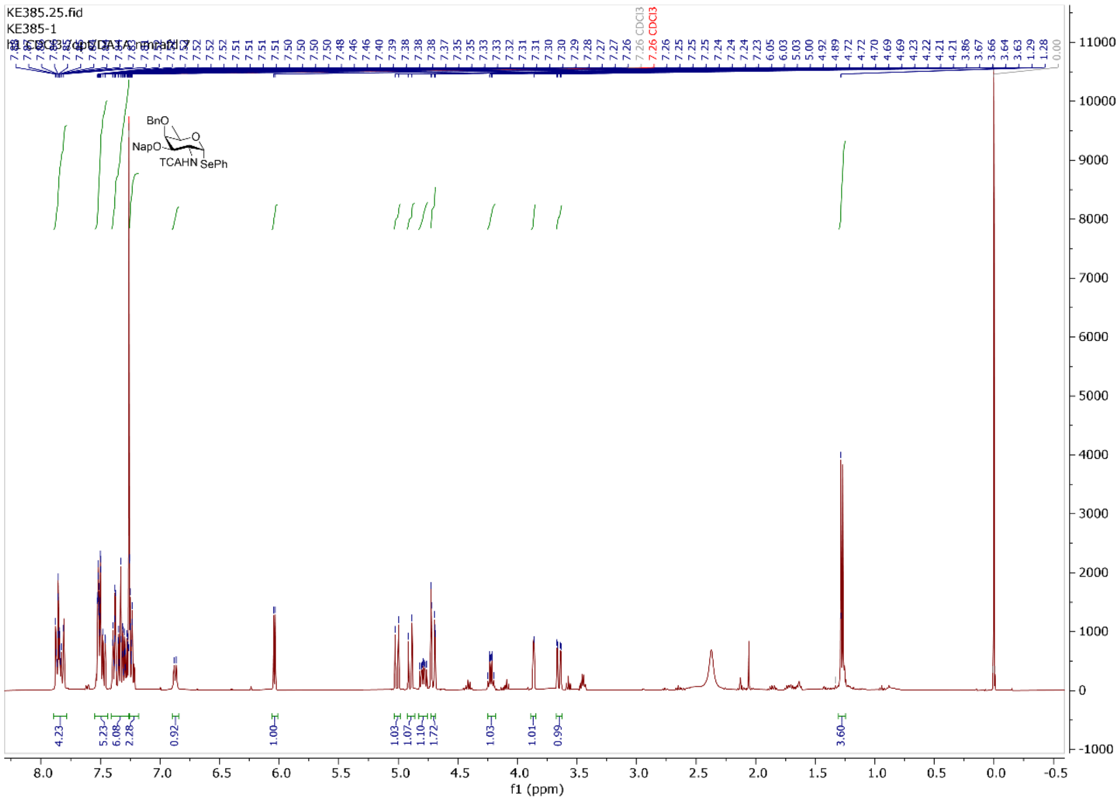
KE385.25.fid
KE385-1
BnO
NapO
TCAHN
SePh
f1 (ppm)

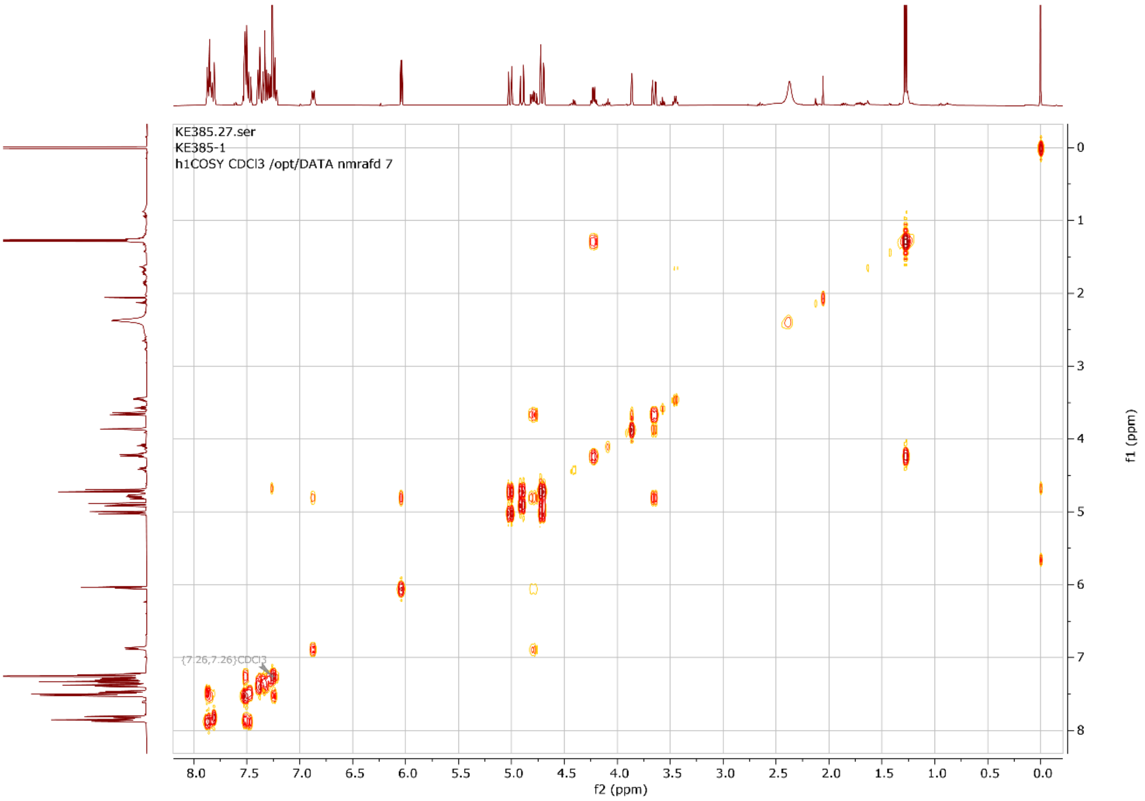
KE385.27.ser
KE385-1
h1COSY CDCl3 /opt/DATA nmrafd 7
f2 (ppm)
f1 (ppm)

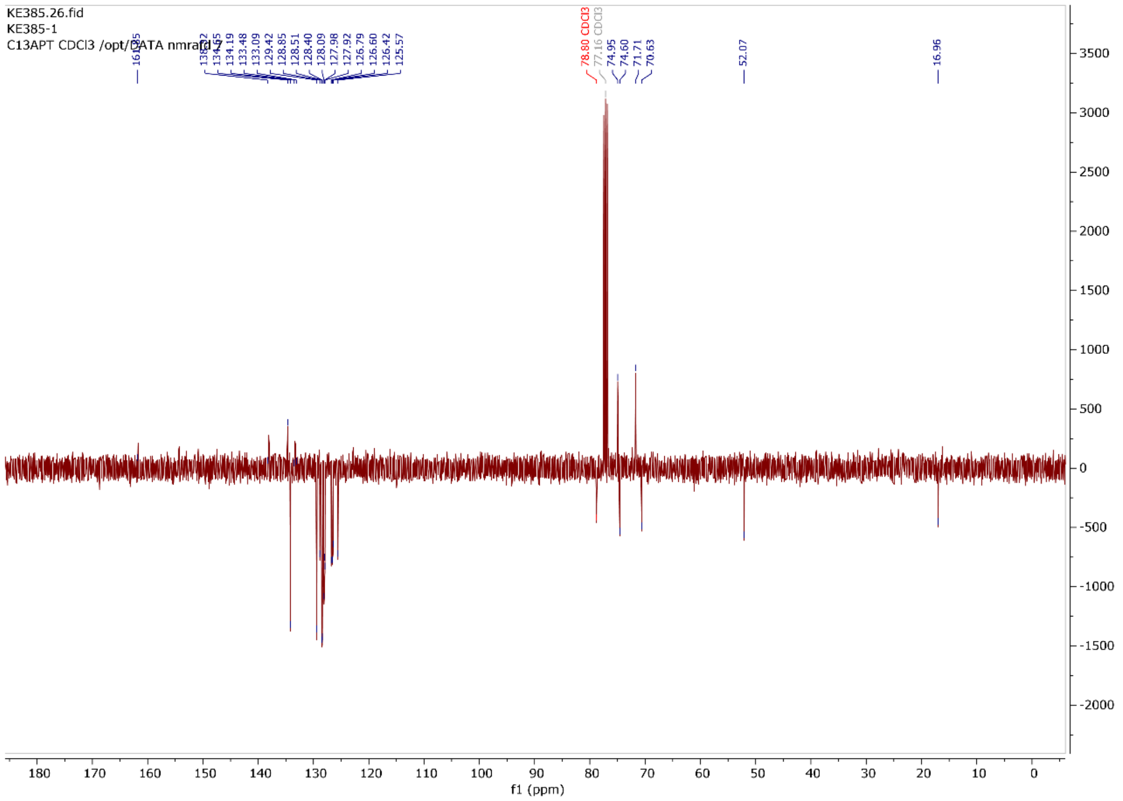
KE385.26.fid
KE385-1
C13APT CDCl3 /opt/DATA nmrafd 7
134.19
133.48
133.09
129.42
128.85
128.51
128.40
128.09
127.98
127.92
126.79
126.60
126.42
125.57
78.80 CDCl3
77.16 CDCl3
74.95
74.60
71.71
70.63
52.07
16.96
f1 (ppm)

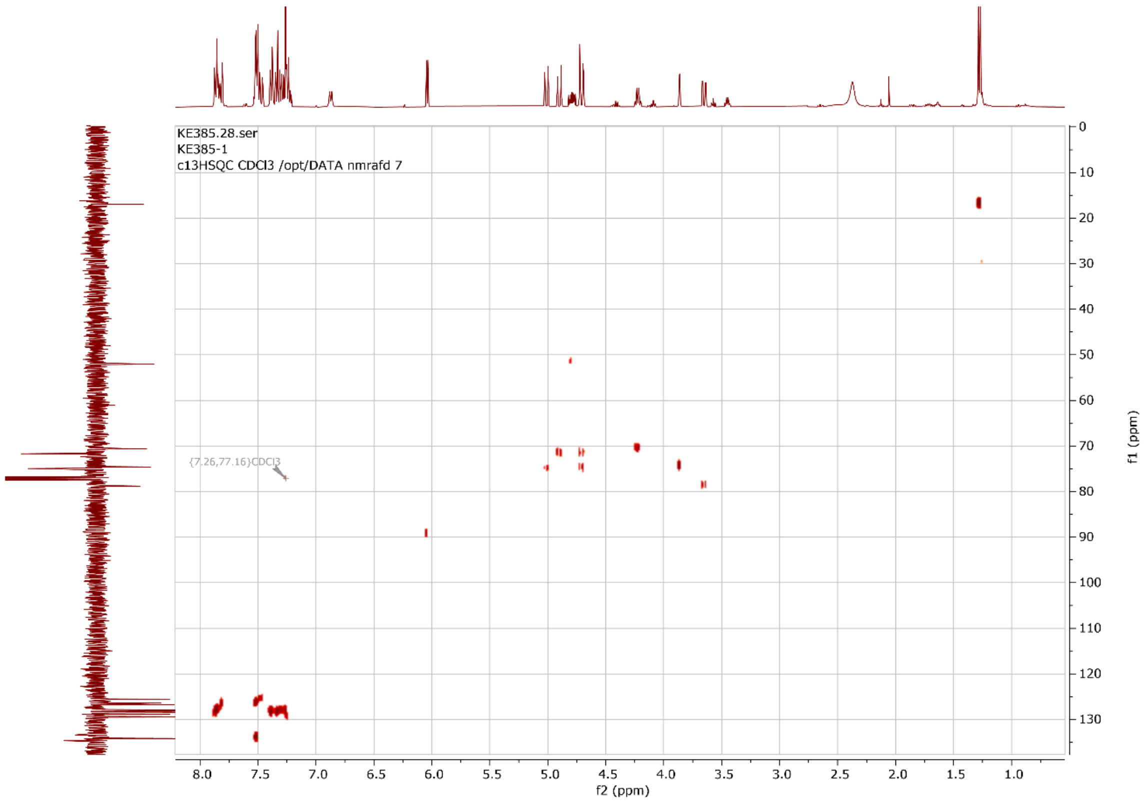
KE385.28.ser
KE385-1
c13HSQC CDCl3 /opt/DATA nmrafd 7
{7.26,77.16}CDCl3
f2 (ppm)
f1 (ppm)

4-*O*-benzyl-2-deoxy-3-*O*-(2-naphthylmethyl)-2-*N*-trichloroacetamide-α-D-fucopyranose (S7)

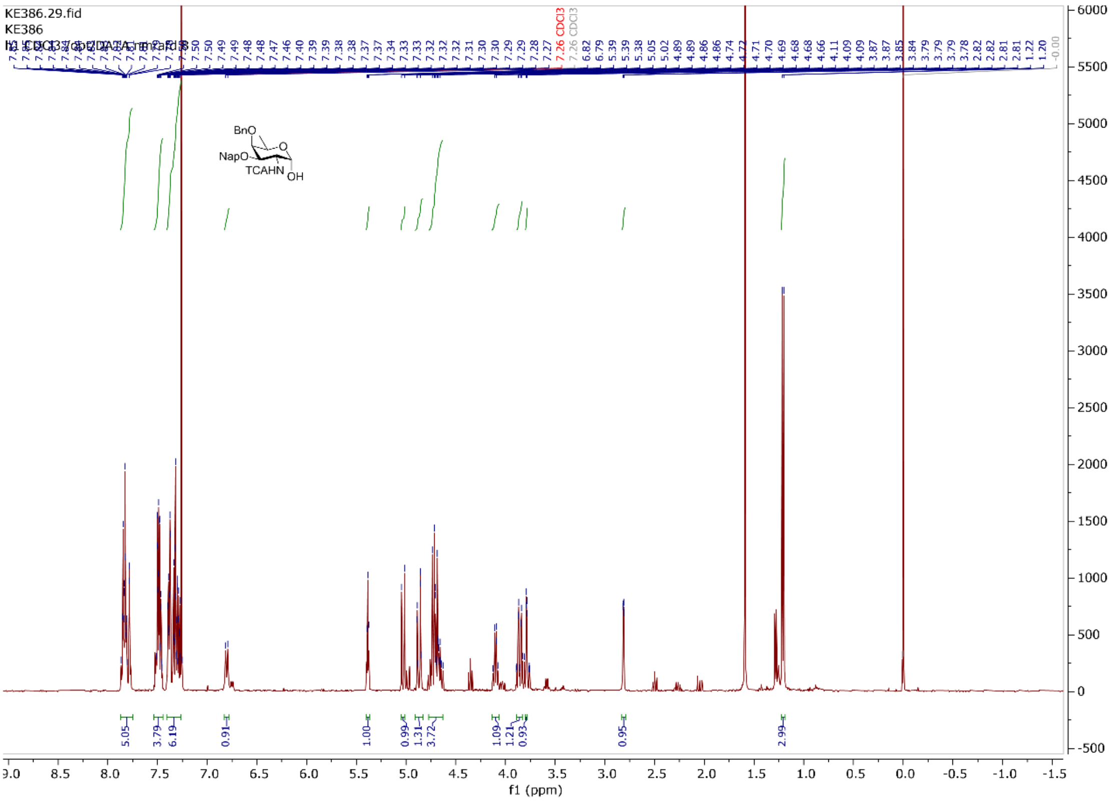

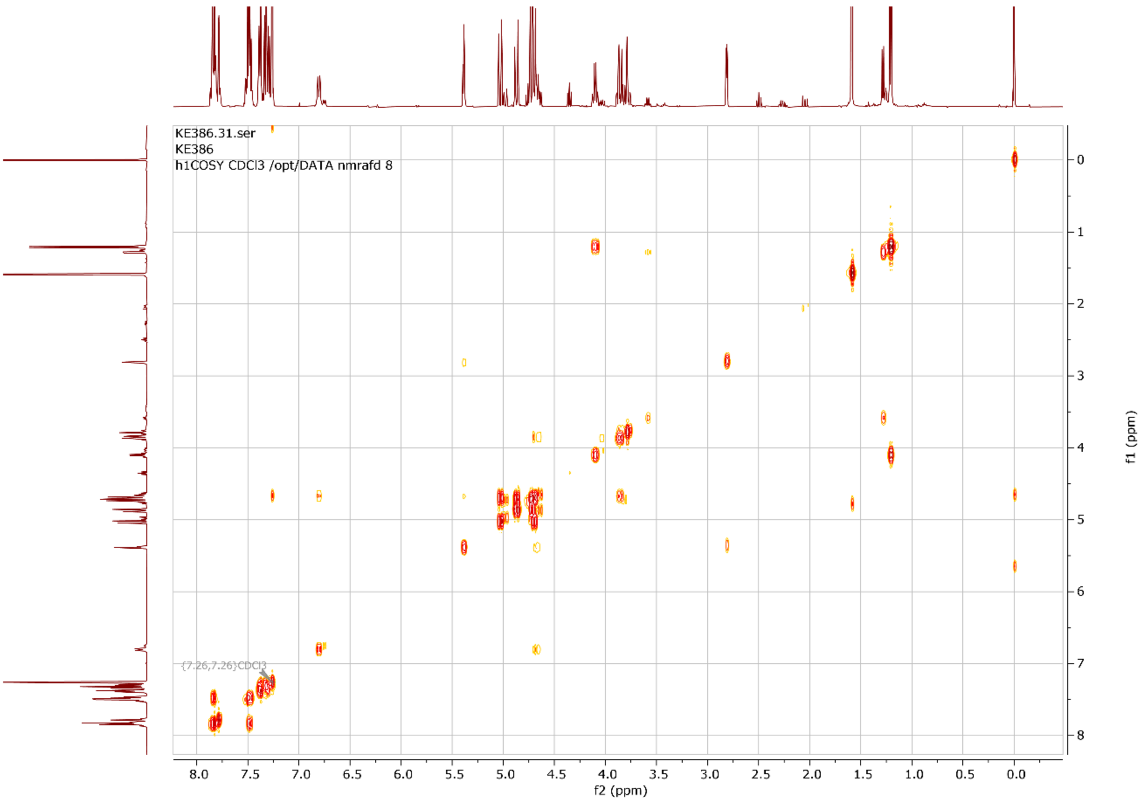
KE386.31.ser
KE386
h1COSY CDCl3 /opt/DATA nmrafd 8
{7.26,7.26}CDCl3
f2 (ppm)
f1 (ppm)

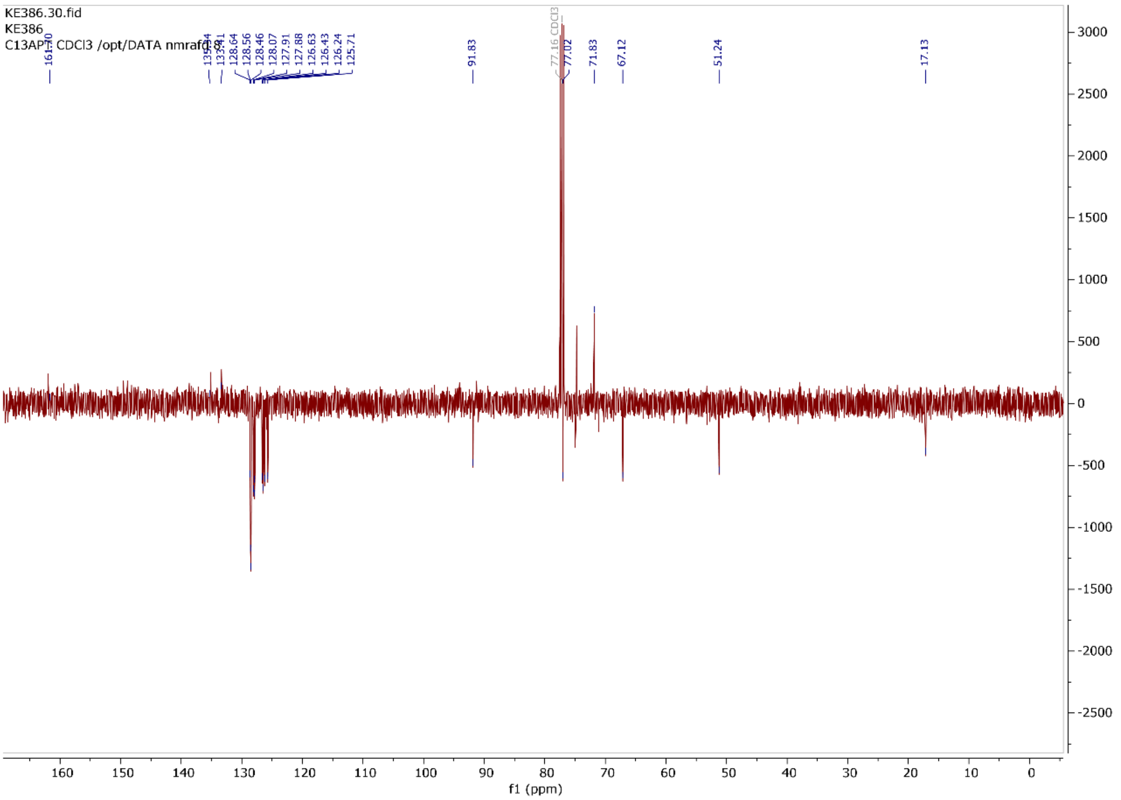
KE386.30.fid
KE386
C13APT CDCl3 /opt/DATA nmrafd 8
128.64
128.56
128.46
128.07
127.91
127.88
126.63
126.43
126.24
125.71
91.83
77.16 CDCl3
77.02
71.83
67.12
51.24
17.13
f1 (ppm)

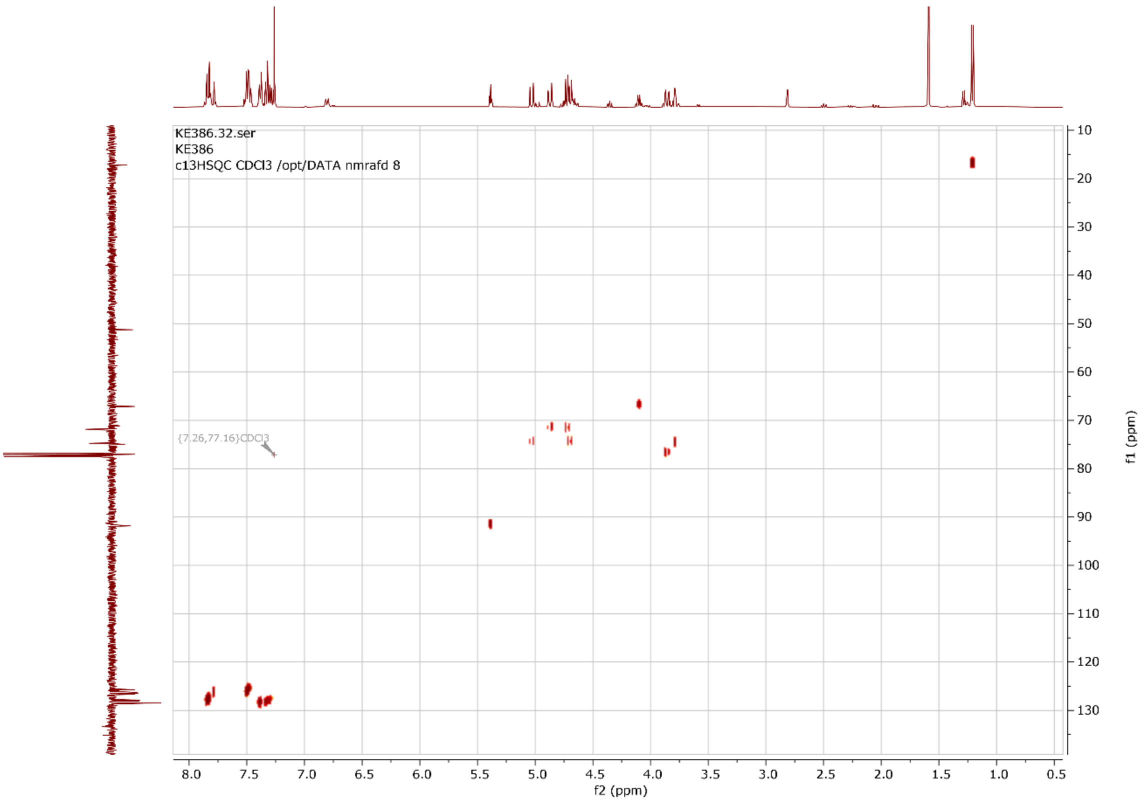

***Tert*-butyldiphenylsilyl 4-*O*-benzyl-2-deoxy-3-*O*-(2-naphthylmethyl)-2-*N*-trichloroacetamide-β-D-fucopyranoside (S8)**

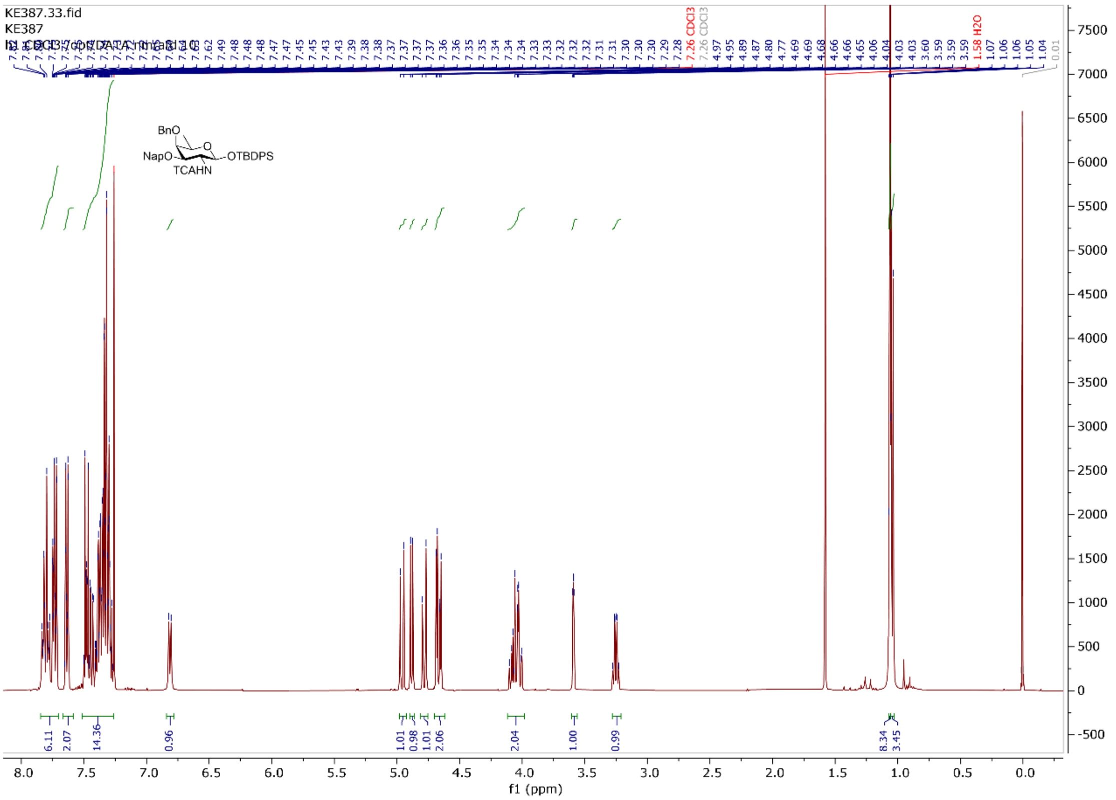
KE387.33.fid
KE387
BnO
NapO
O
OTBDPS
TCAHN
f1 (ppm)

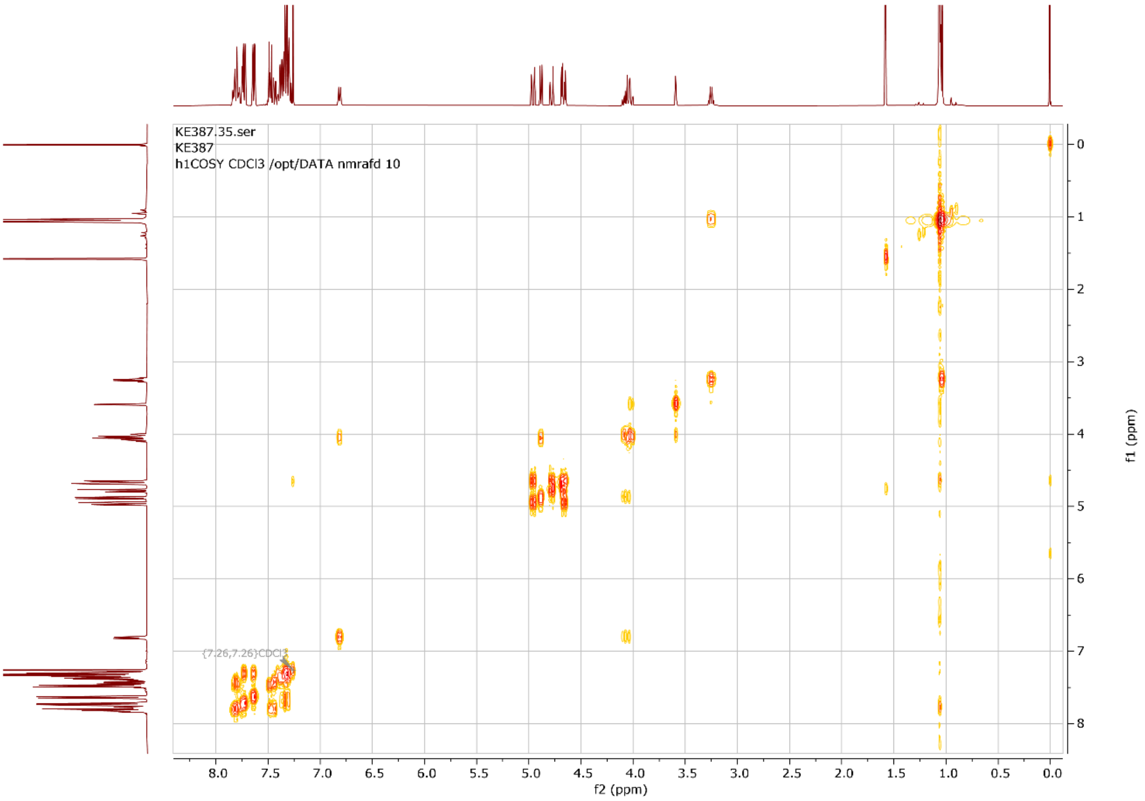
KE387.35.ser
KE387
h1COSY CDCl3 /opt/DATA nmrafd 10
f2 (ppm)
f1 (ppm)

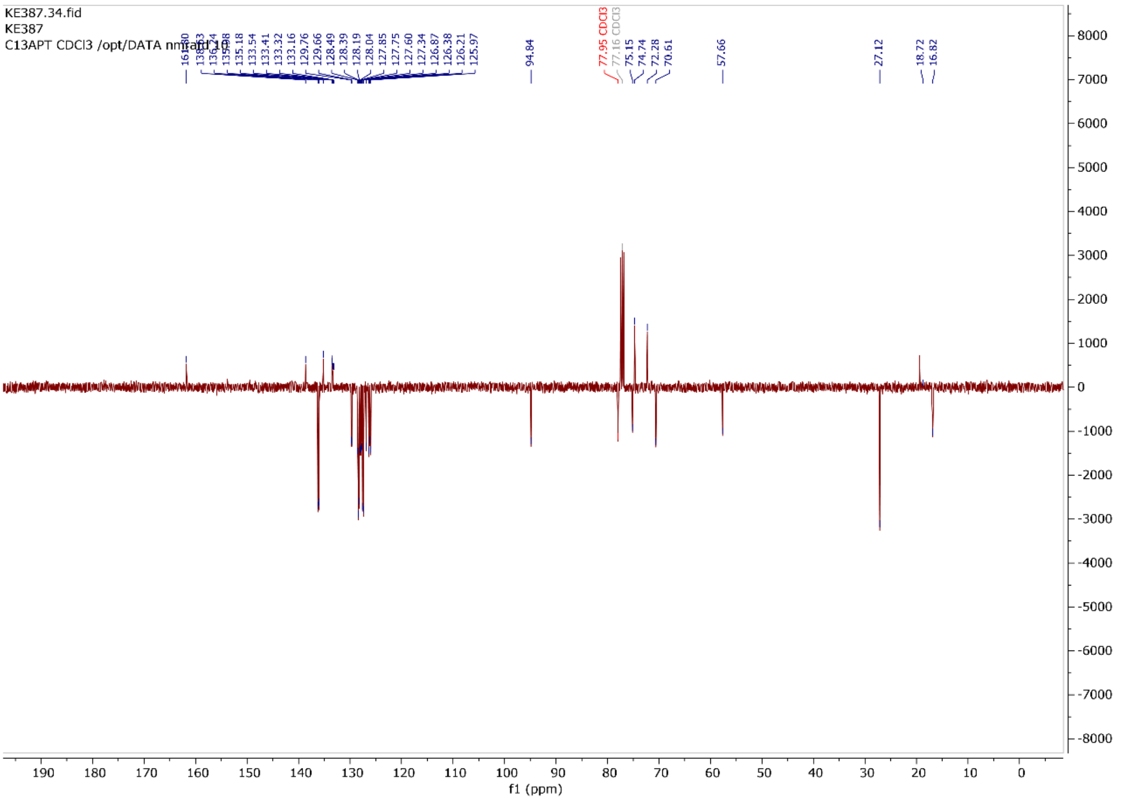

KE387.34.fid
KE387
C13APT CDCl3 /opt/DATA nmrafd 10
136.24
135.18
133.54
133.41
133.32
133.16
129.76
129.66
128.49
128.39
128.19
128.04
127.85
127.75
127.60
127.34
126.87
126.38
126.21
125.97
94.84
77.95 CDCl3
77.16 CDCl3
75.15
74.74
72.28
70.61
57.66
27.12
18.72
16.82
f1 (ppm)

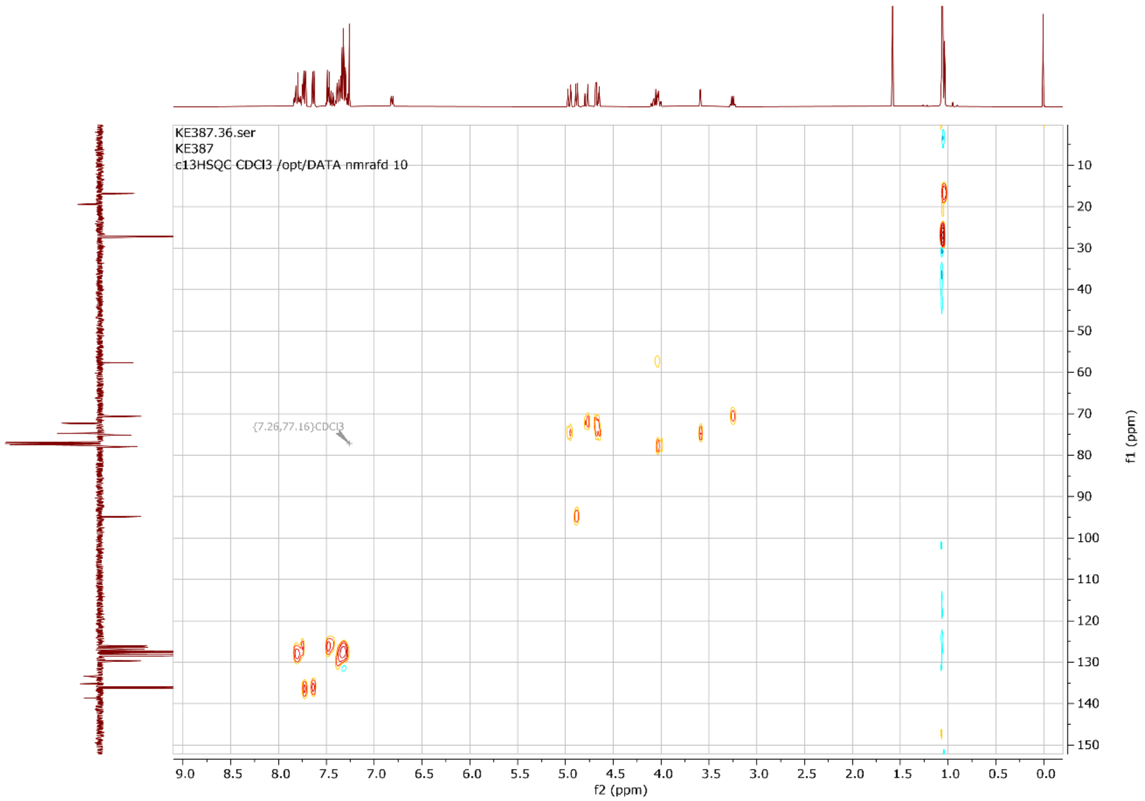

KE387.36.ser
KE387
c13HSQC CDCl3 /opt/DATA nmrafd 10
{7.26,77.16}CDCl3
f2 (ppm)
f1 (ppm)

***Tert*-butyldiphenylsilyl 4-*O*-benzyl-2-deoxy-2-*N*-trichloroacetamide-β-D-fucopyranoside (13)**

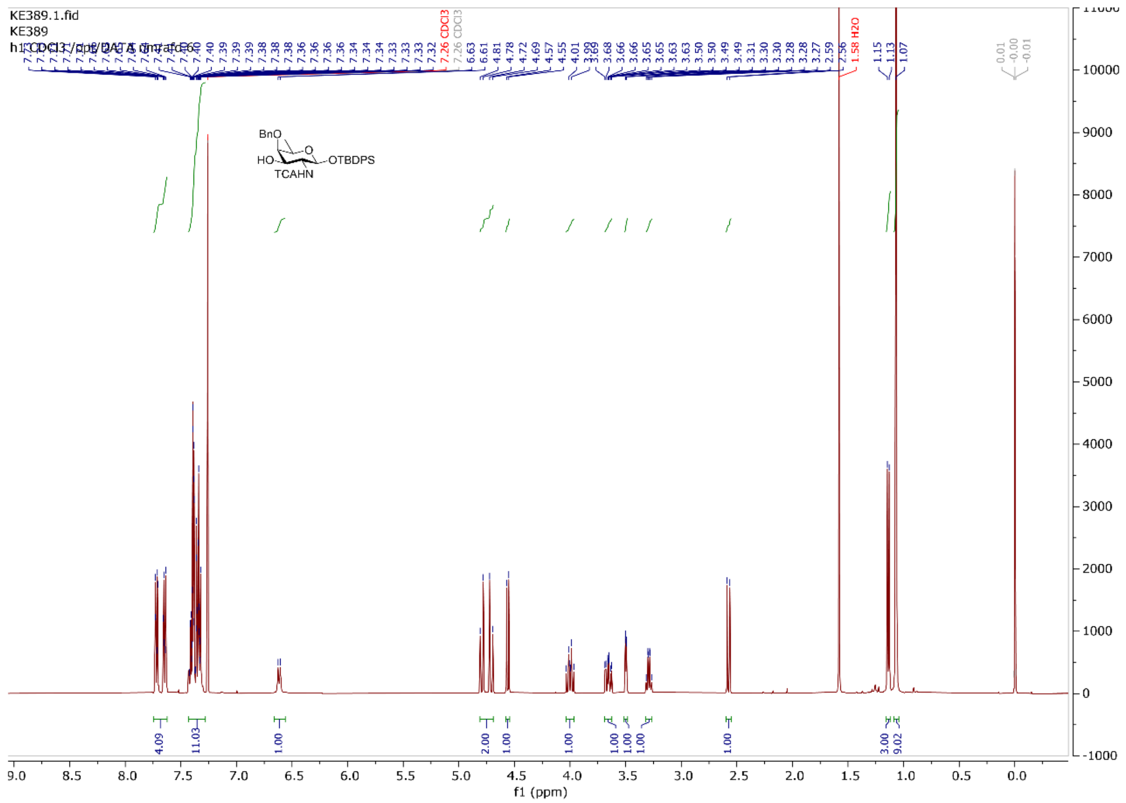

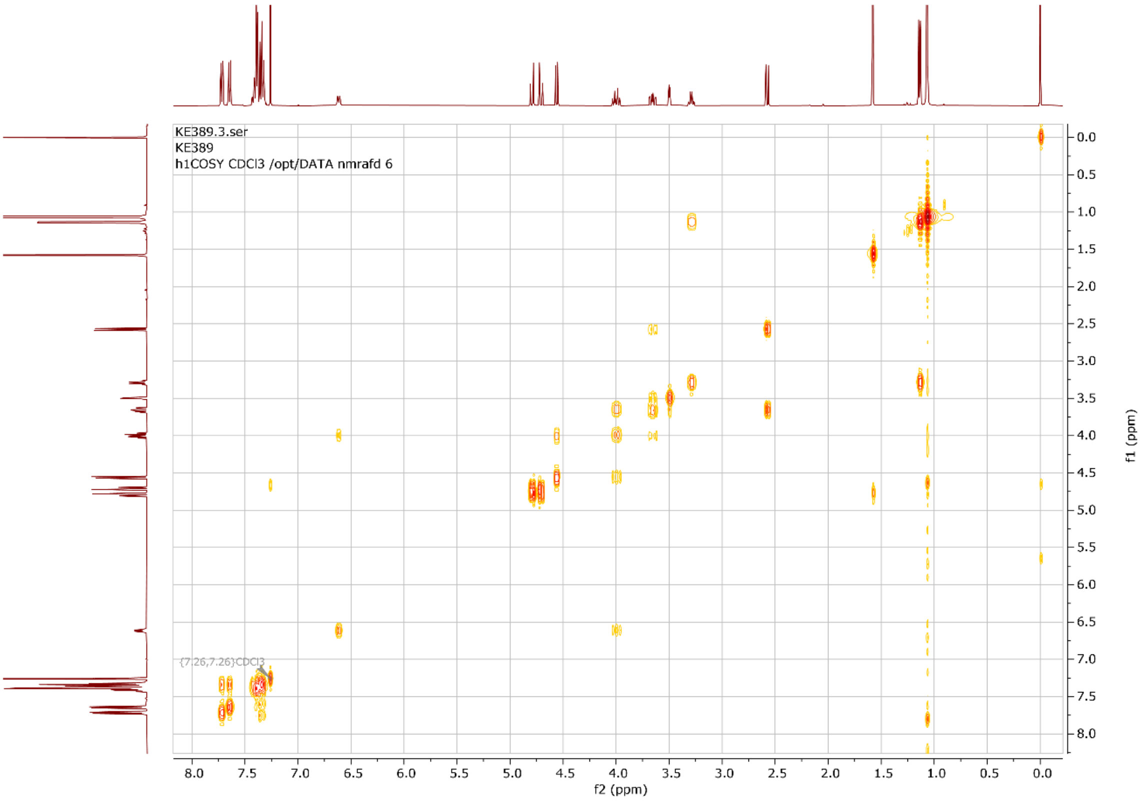
KE389.3.ser
KE389
h1COSY CDCl3 /opt/DATA nmrafd 6
{7.26,7.26}CDCl3
f2 (ppm)
f1 (ppm)

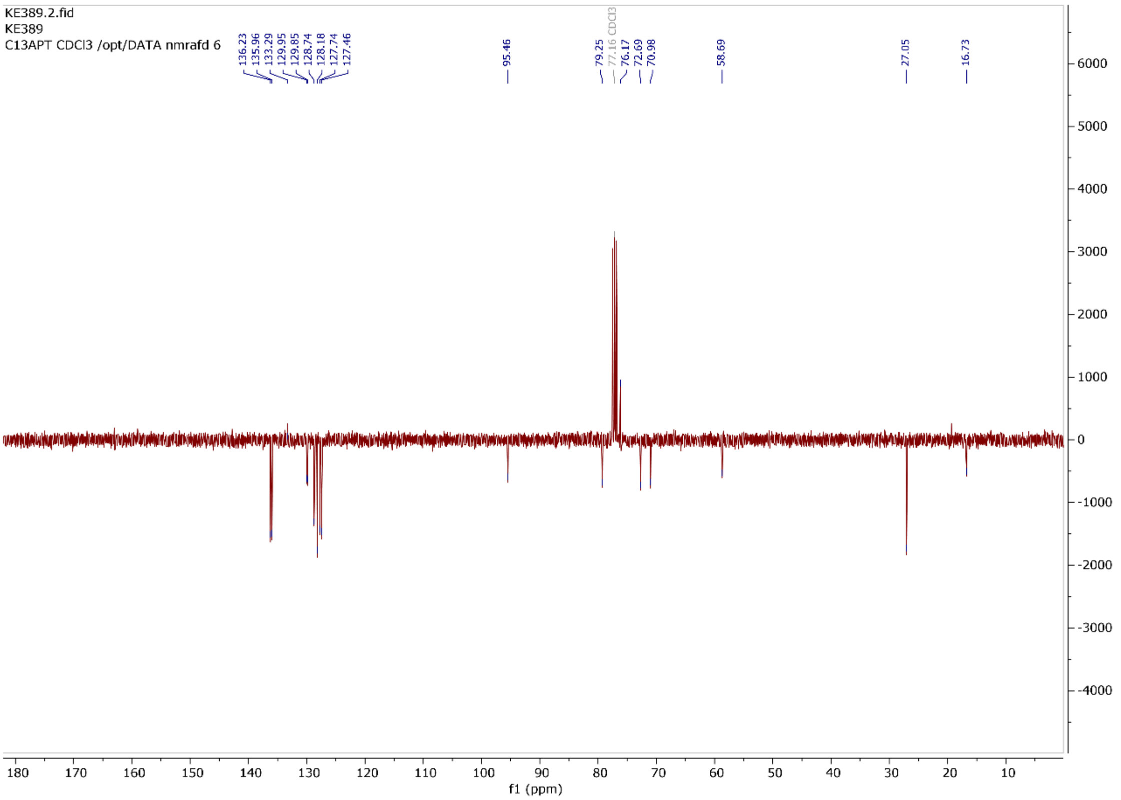
KE389.2.fid
KE389
C13APT CDCl3 /opt/DATA nmrafd 6
136.23
135.96
133.29
129.95
129.85
128.74
128.18
127.74
127.46
95.46
79.25
77.16 CDCl3
76.17
72.69
70.98
58.69
27.05
16.73
f1 (ppm)

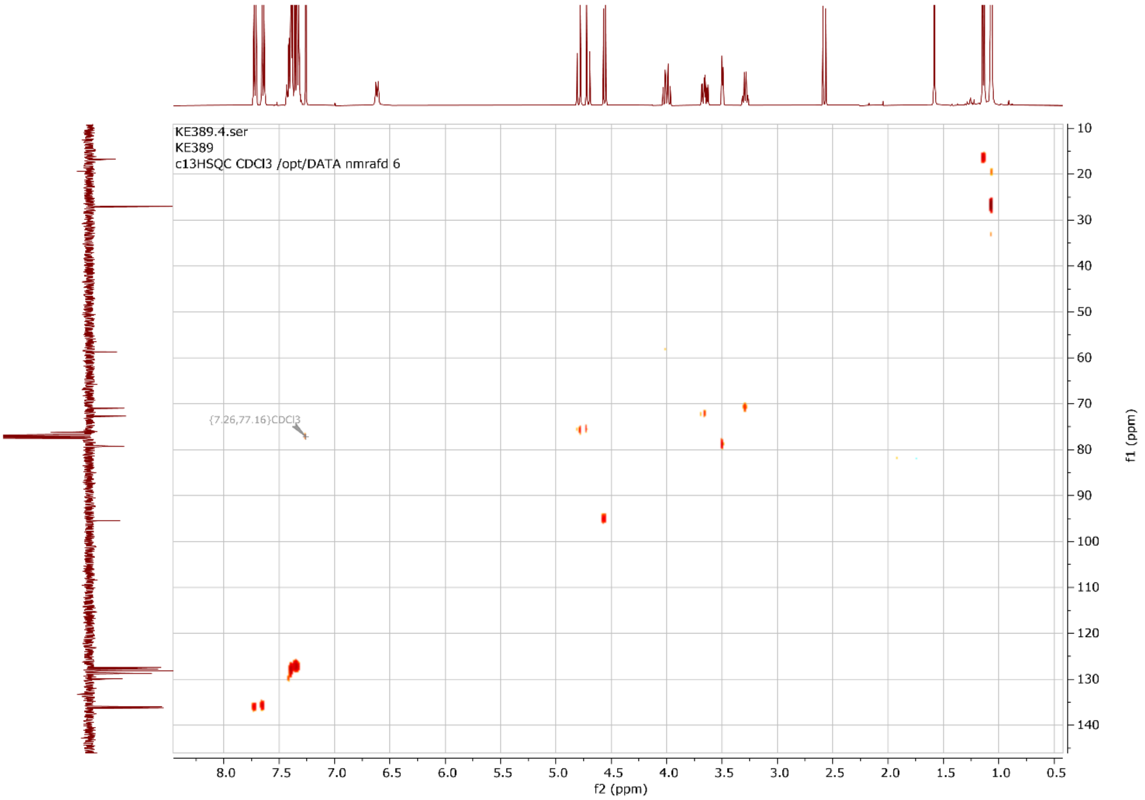

Phenyl 2-azido-2-deoxy-3-*O*-(*p*-methoxybenzyl)-1-seleno-α-D-fucopyranoside (S9)

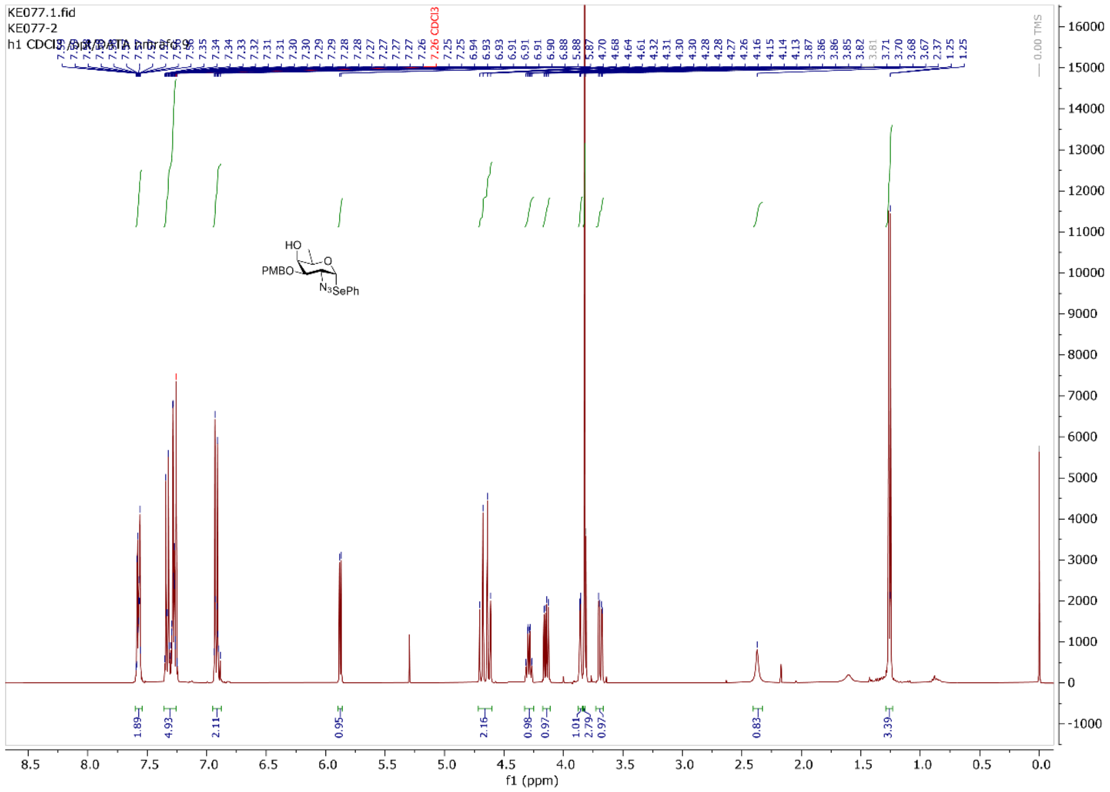
KE077.1.fid
KE077-2
h1 CDCl3 /opt/DATA nmrafd 9
HO
PMBO
O
N3
SePh
f1 (ppm)

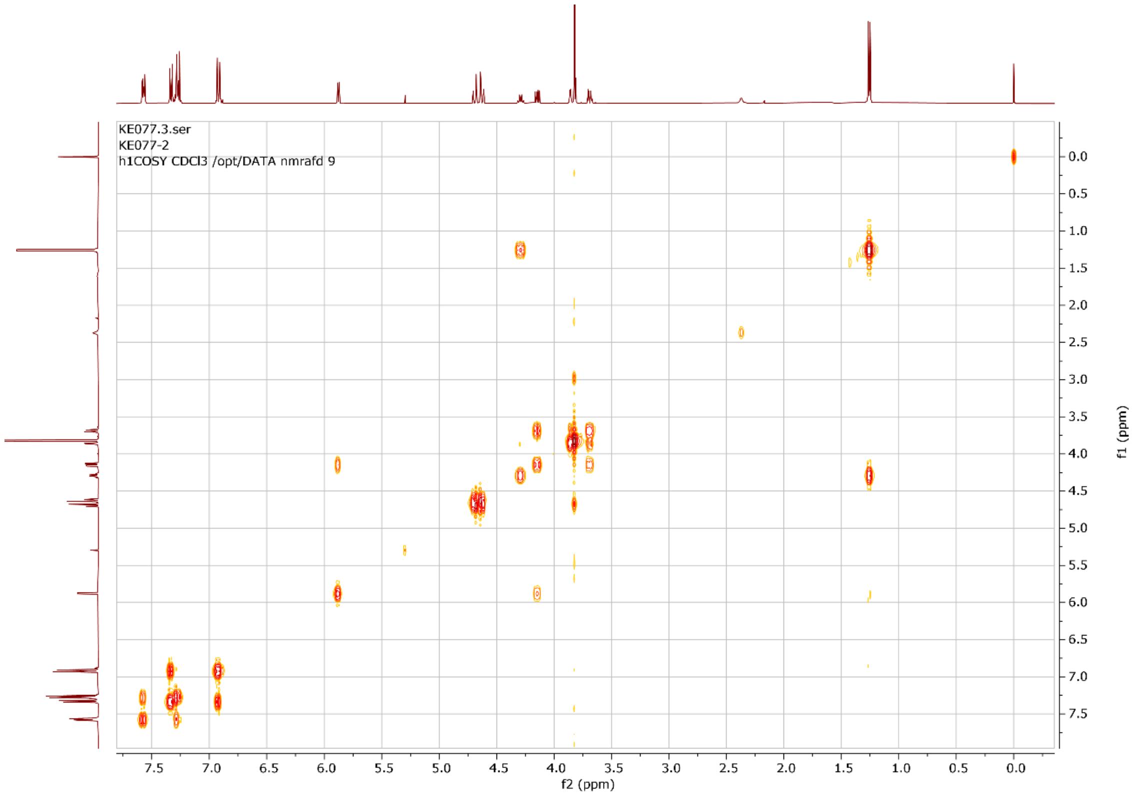
KE077.3.ser
KE077-2
h1COSY CDCl3 /opt/DATA nmrafd 9
f2 (ppm)
f1 (ppm)

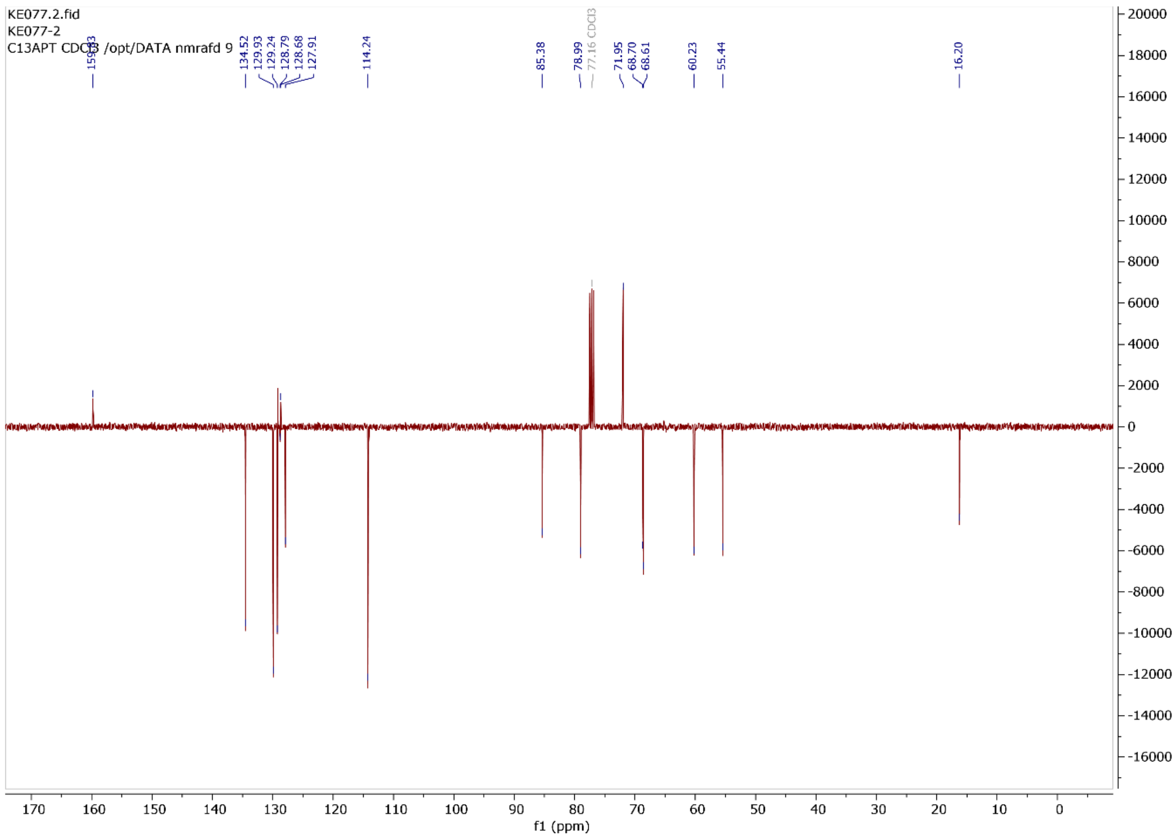
KE077.2.fid
KE077-2
C13APT CDCl3 /opt/DATA nmrafd 9
159.93
134.52
129.93
129.24
128.79
128.68
127.91
114.24
85.38
78.99
77.16 CDCl3
71.95
68.70
68.61
60.23
55.44
16.20
f1 (ppm)

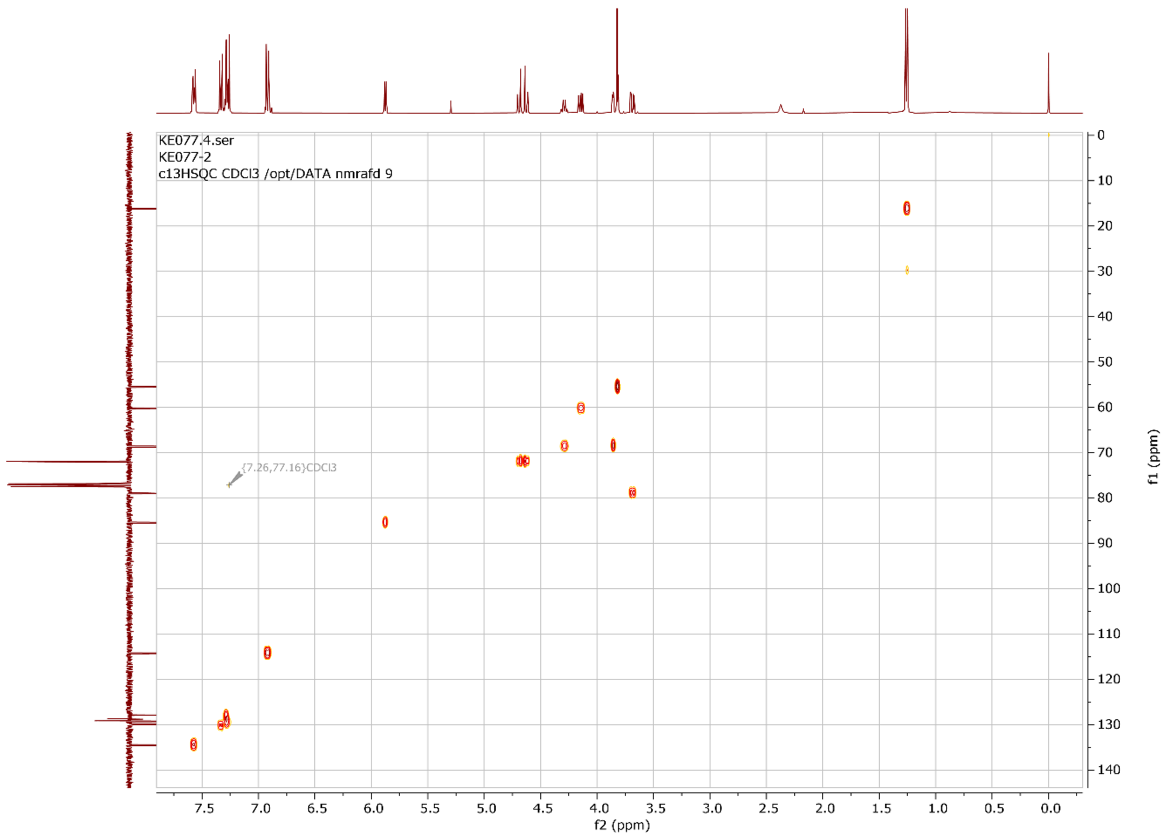
KE077.4.ser
KE077-2
c13HSQC CDCl3 /opt/DATA nmrafd 9
{7.26,77.16}CDCl3
f2 (ppm)
f1 (ppm)

Phenyl 2-azido-4-*O*-benzyl-2-deoxy-3-*O*-(*p*-methoxybenzyl)-1-seleno-α-D-fucopyranoside (S10)

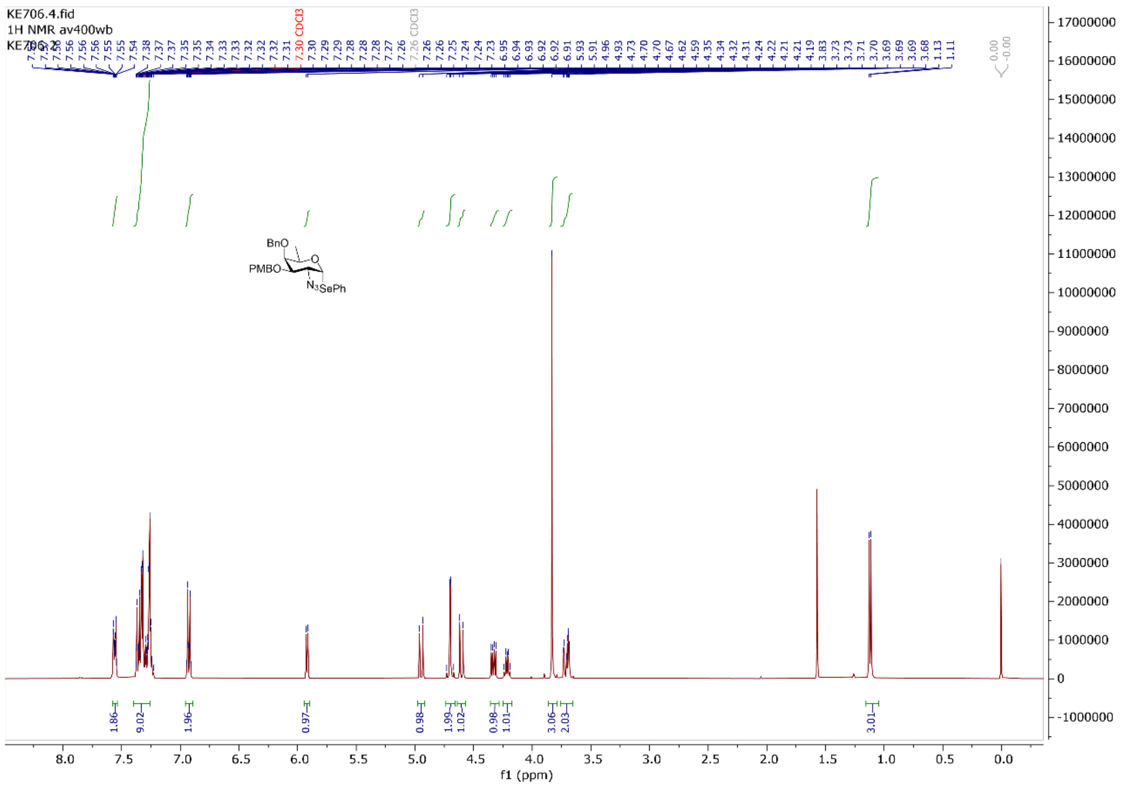

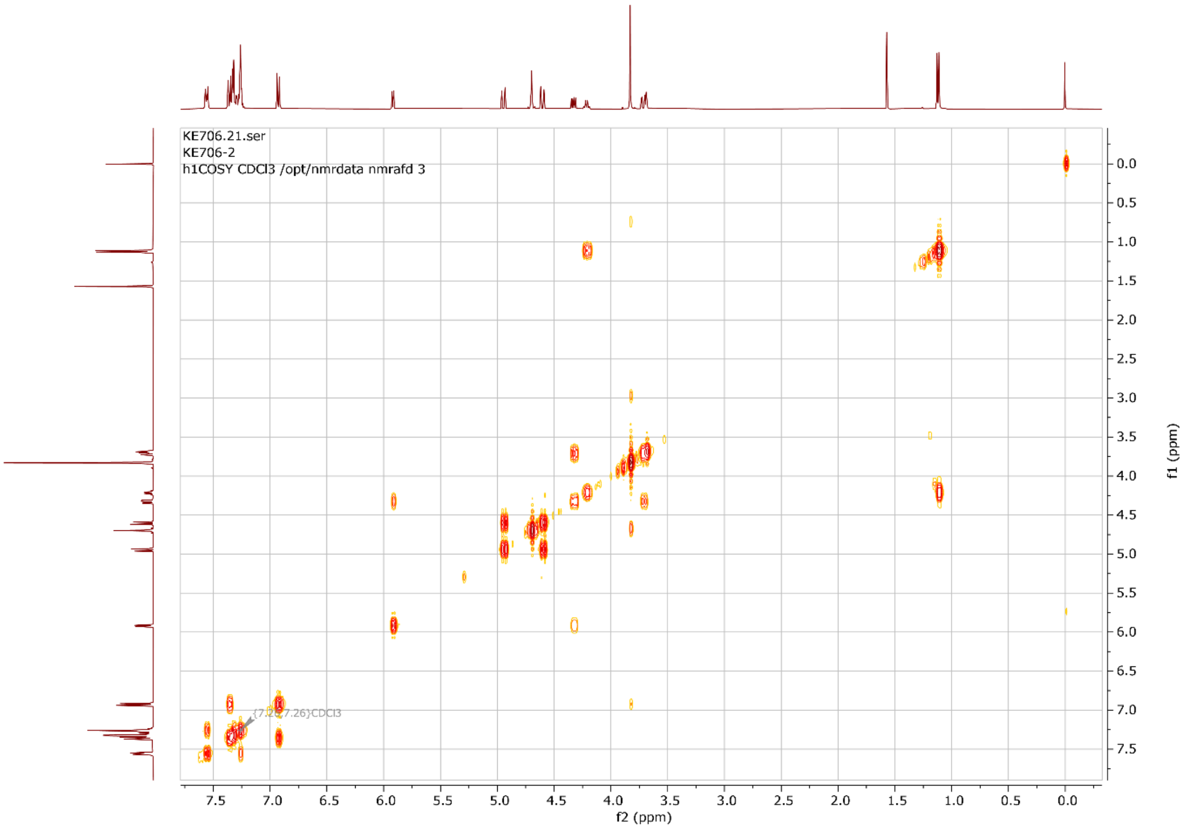

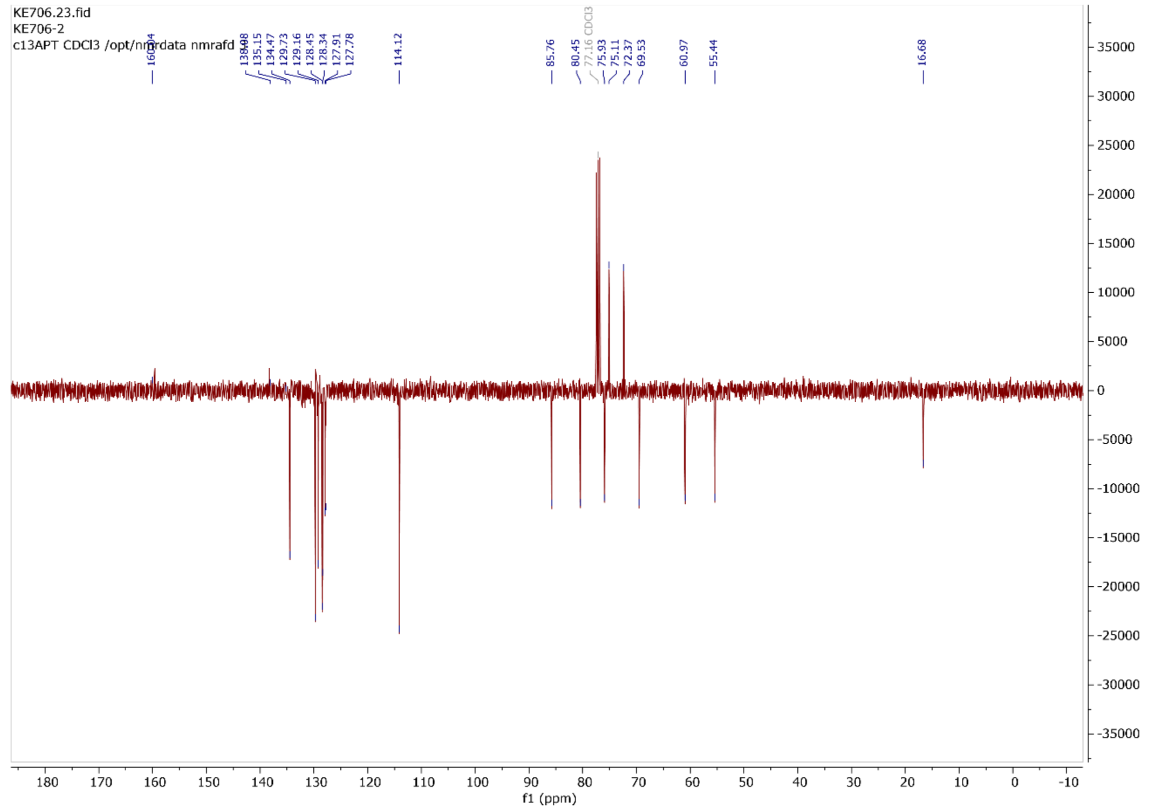
KE706.23.fid
KE706-2
c13APT CDCl3 /opt/nmrdata nmrafd 3
160.04
138.08
135.15
134.47
129.73
129.16
128.45
128.34
127.91
127.78
114.12
85.76
80.45
77.16 CDCl3
75.93
75.11
72.37
69.53
60.97
55.44
16.68
f1 (ppm)

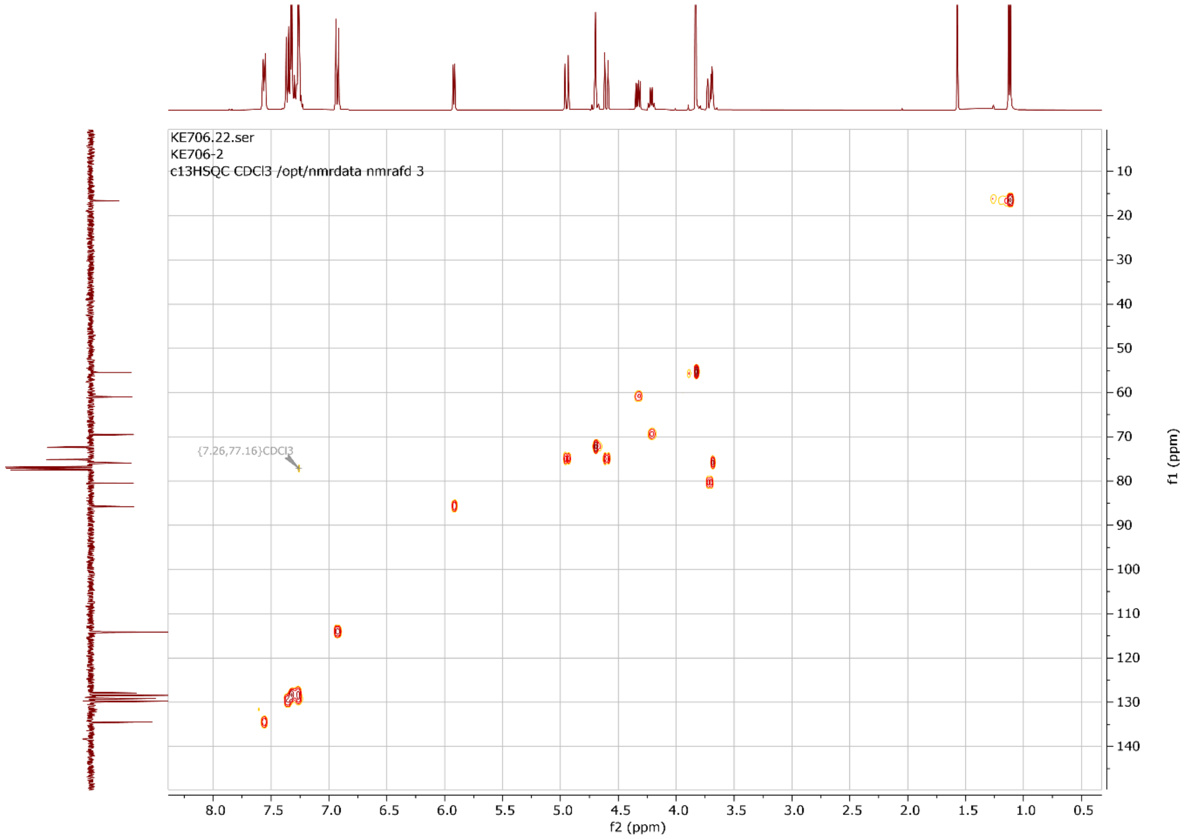
KE706.22.ser
KE706-2
c13HSQC CDCl3 /opt/nmrdata nmrafd 3
{7.26,77.16}CDCl3
f2 (ppm)
f1 (ppm)

Phenyl 4-*O*-benzyl-2-deoxy-3-*O*-(*p*-methoxybenzyl)-2-*N*-trifluoroacetamide-1-seleno-α-D-fucopyranoside (S11)

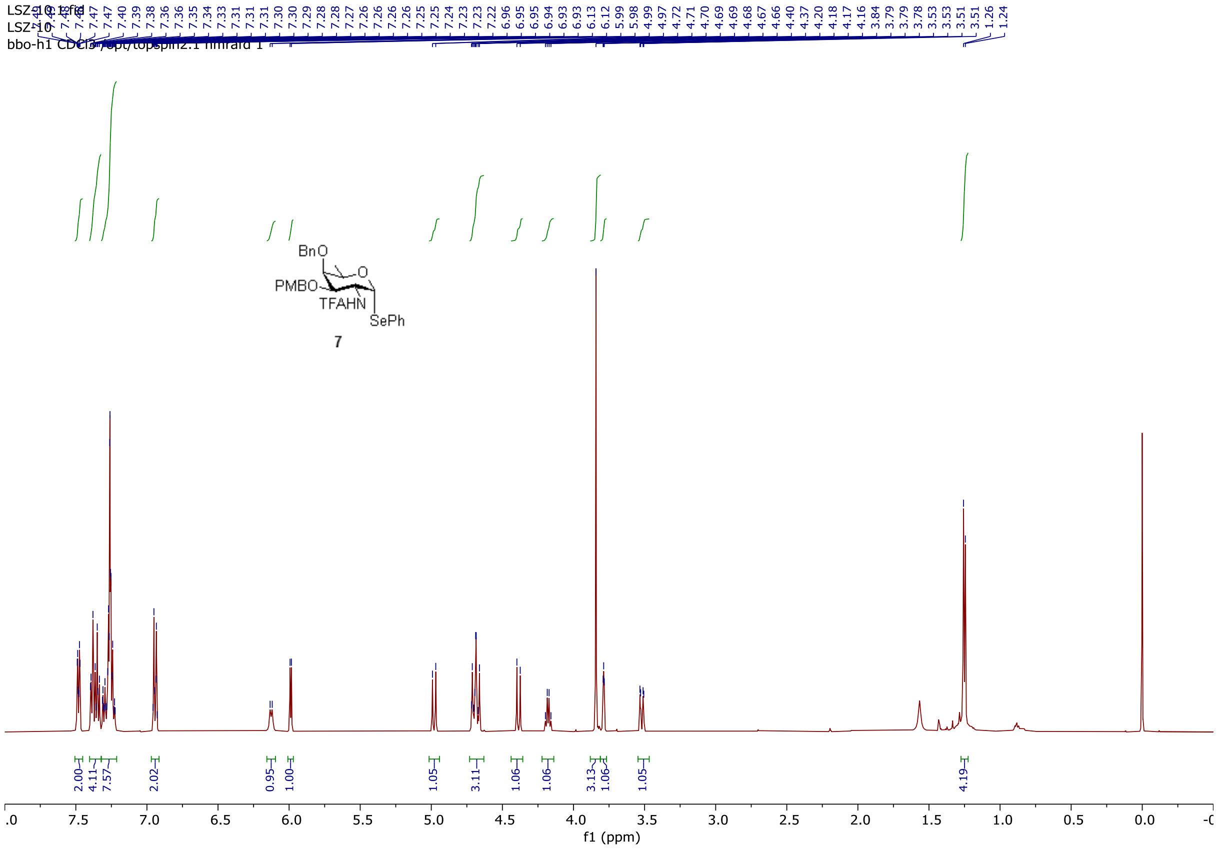

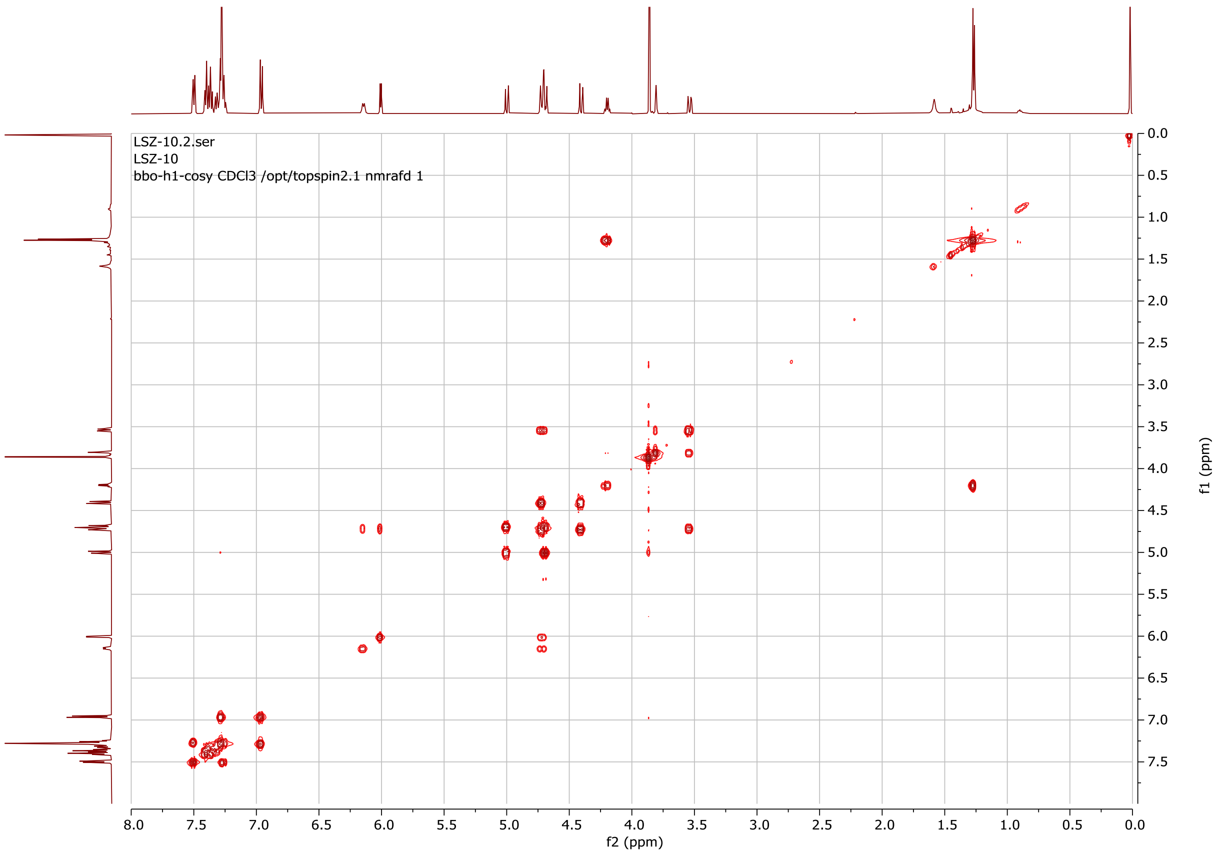
LSZ-10.2.ser
LSZ-10
bbo-h1-cosy CDCl3 /opt/topspin2.1 nmrafd 1
f2 (ppm)
f1 (ppm)

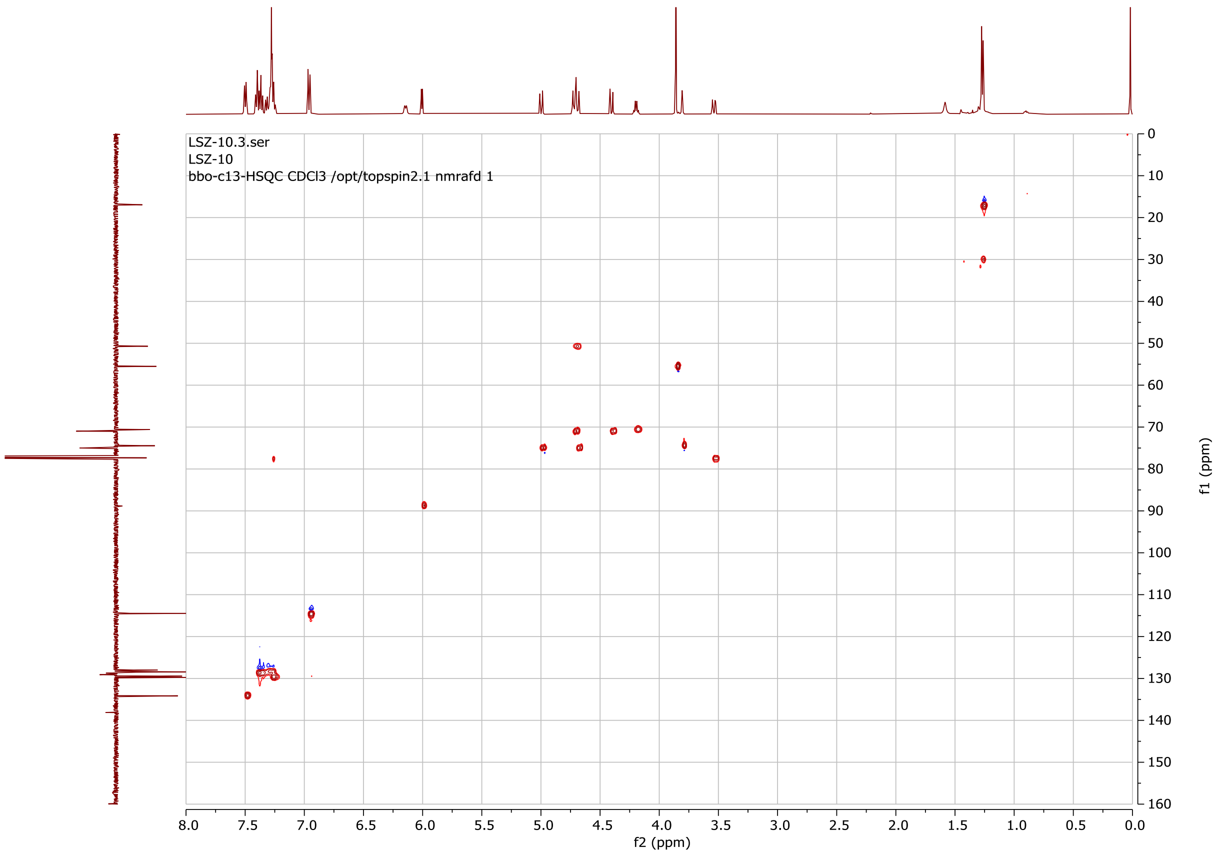
LSZ-10.3.ser
LSZ-10
bbo-c13-HSQC CDCl3 /opt/topspin2.1 nmrafd 1
f2 (ppm)
f1 (ppm)

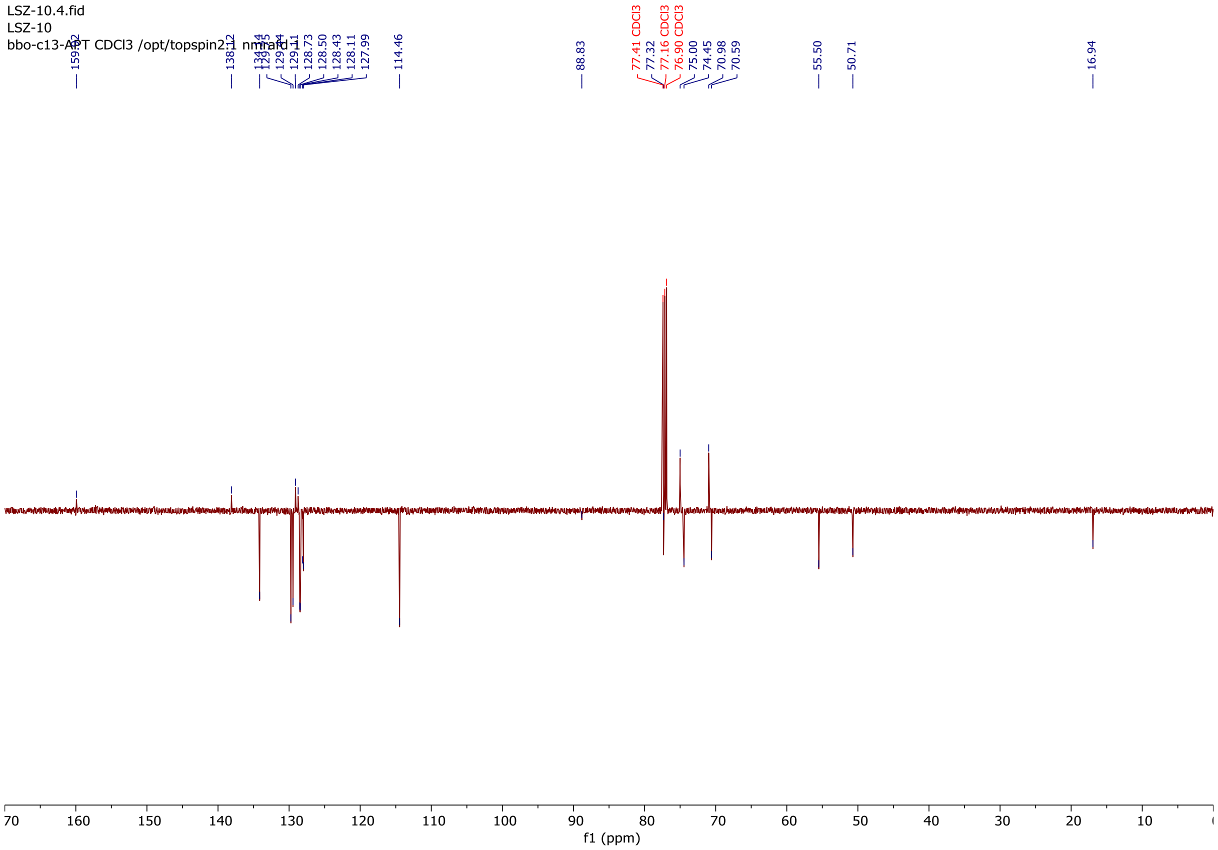

***Tert*-butyldiphenylsilyl 4-*O*-benzyl-2-deoxy-3-*O*-(*p*-methoxybenzyl)-2-*N*-trifluoroacetamide-β-D-fucopyranoside (S13)**

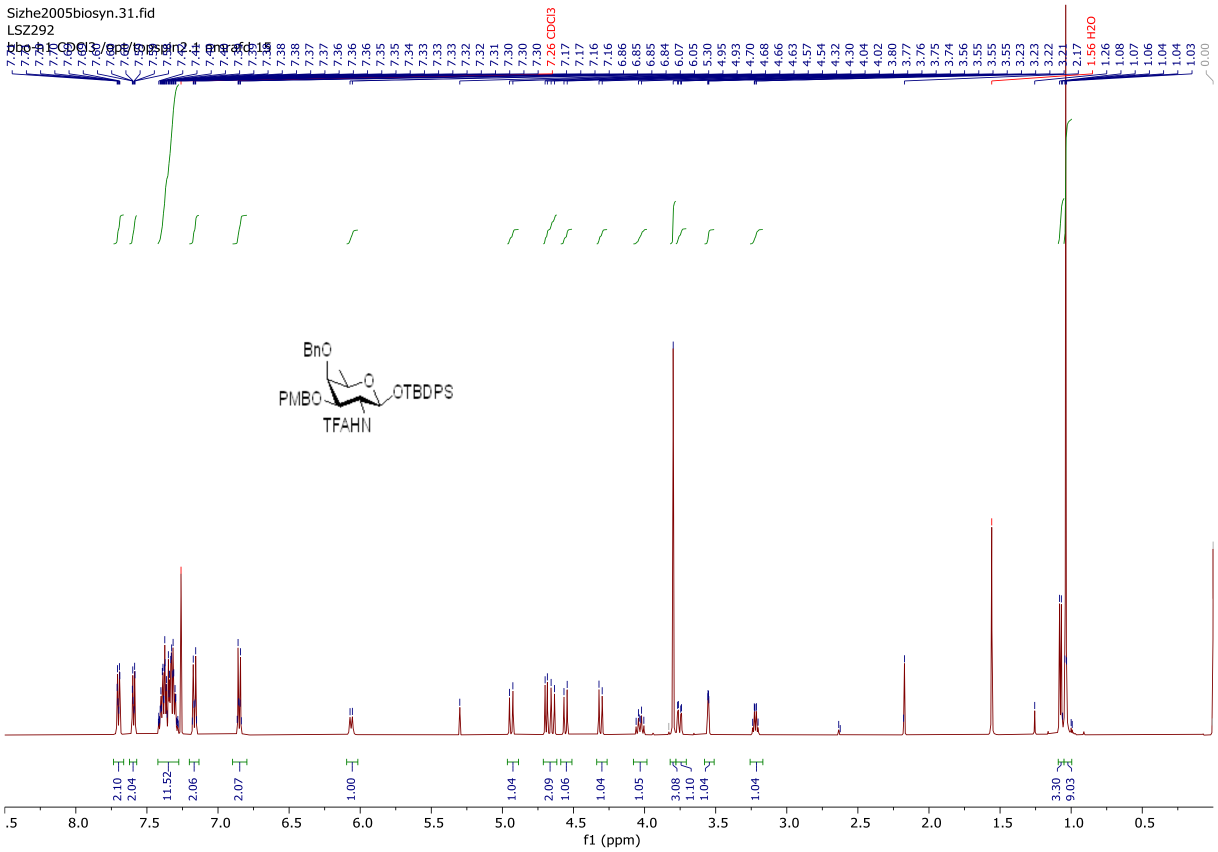
Sizhe2005biosyn.31.fid
LSZ292
bbo-h1 CDCl3 /opt/topspin2.1 nmrafd 15
7.26 CDCl3
1.56 H2O
BnO
PMBO
O
OTBDPS
TFAHN
2.10
2.04
11.52
2.06
2.07
1.00
1.04
2.09
1.06
1.04
1.05
3.08
1.10
1.04
1.04
3.30
9.03
f1 (ppm)

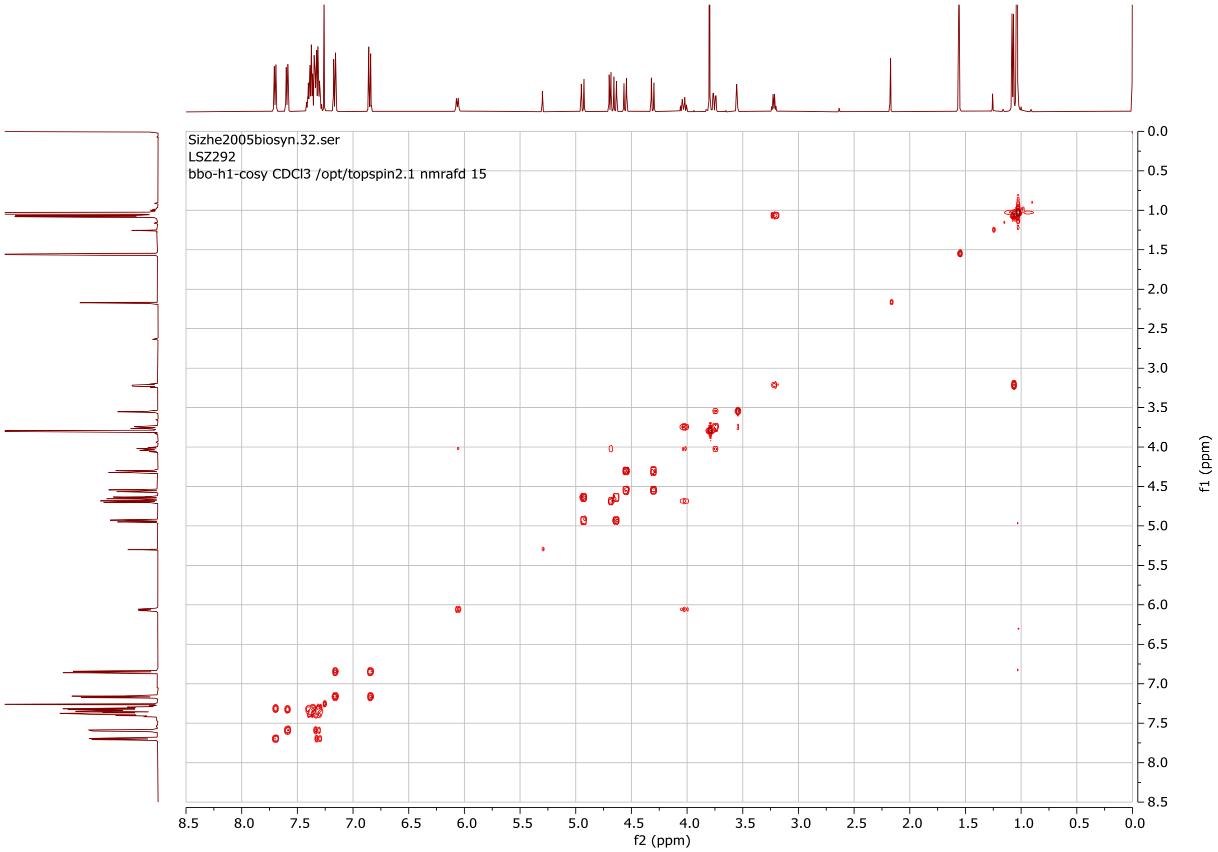
Sizhe2005biosyn.32.ser
LSZ292
bbo-h1-cosy CDCl3 /opt/topspin2.1 nmrafd 15
f2 (ppm)
f1 (ppm)

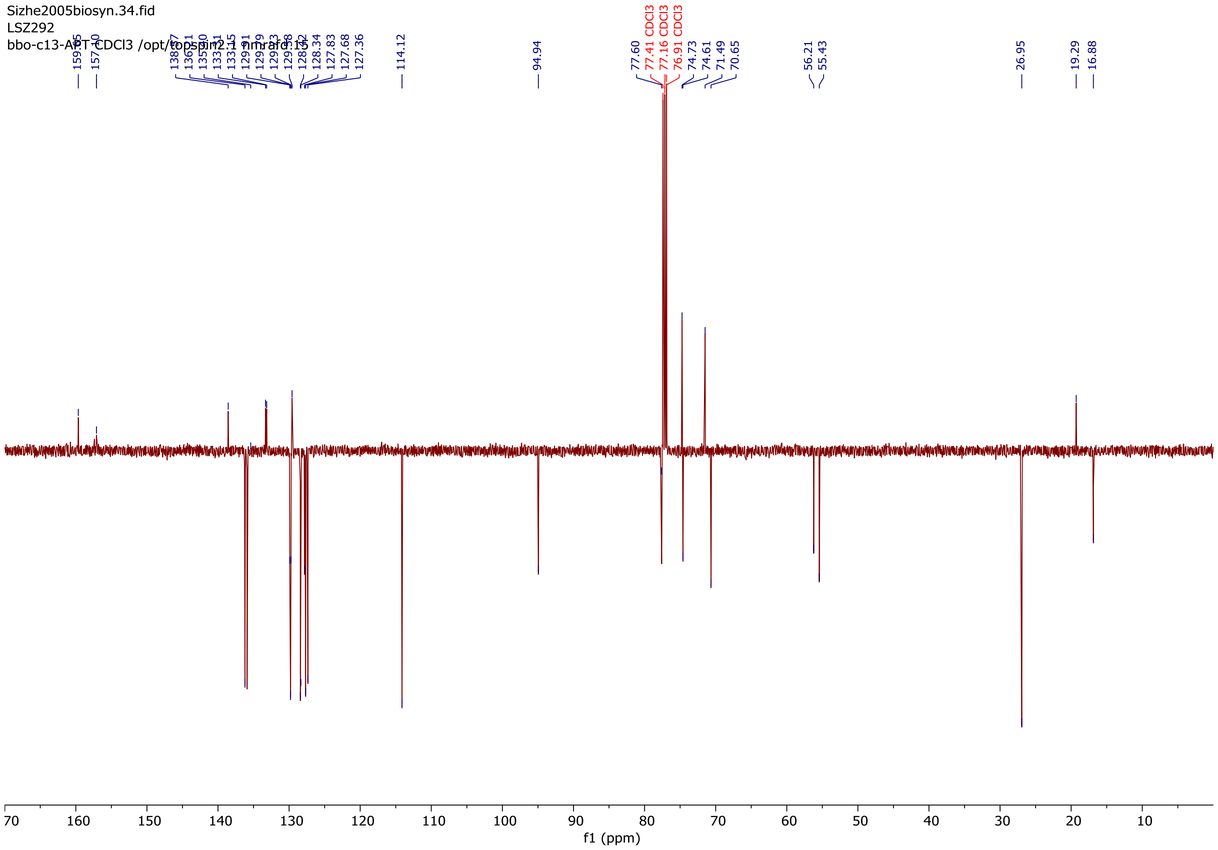

Sizhe2005biosyn.34.fid
LSZ292
bbo-c13-APT CDCl3 /opt/topspin2.1 nmrafd 15
128.34
127.83
127.68
127.36
114.12
94.94
77.60
77.41 CDCl3
77.16 CDCl3
76.91 CDCl3
74.73
74.61
71.49
70.65
56.21
55.43
26.95
19.29
16.88
f1 (ppm)

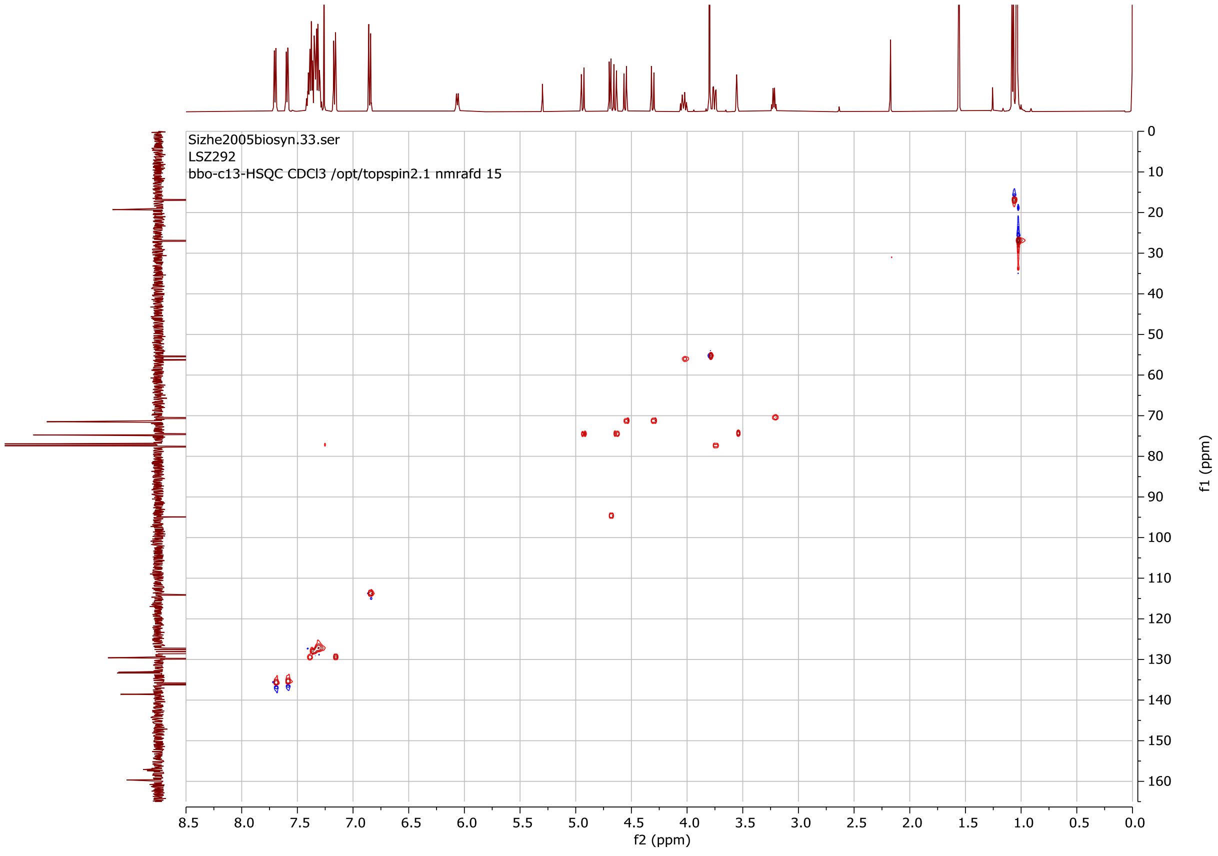

Sizhe2005biosyn.33.ser
LSZ292
bbo-c13-HSQC CDCl3 /opt/topspin2.1 nmrafd 15
f2 (ppm)
f1 (ppm)

***Tert*-butyldiphenylsilyl 4-*O*-benzyl-2-deoxy-2-*N*-trifluoroacetamide-β-D-fucopyranoside (14)**

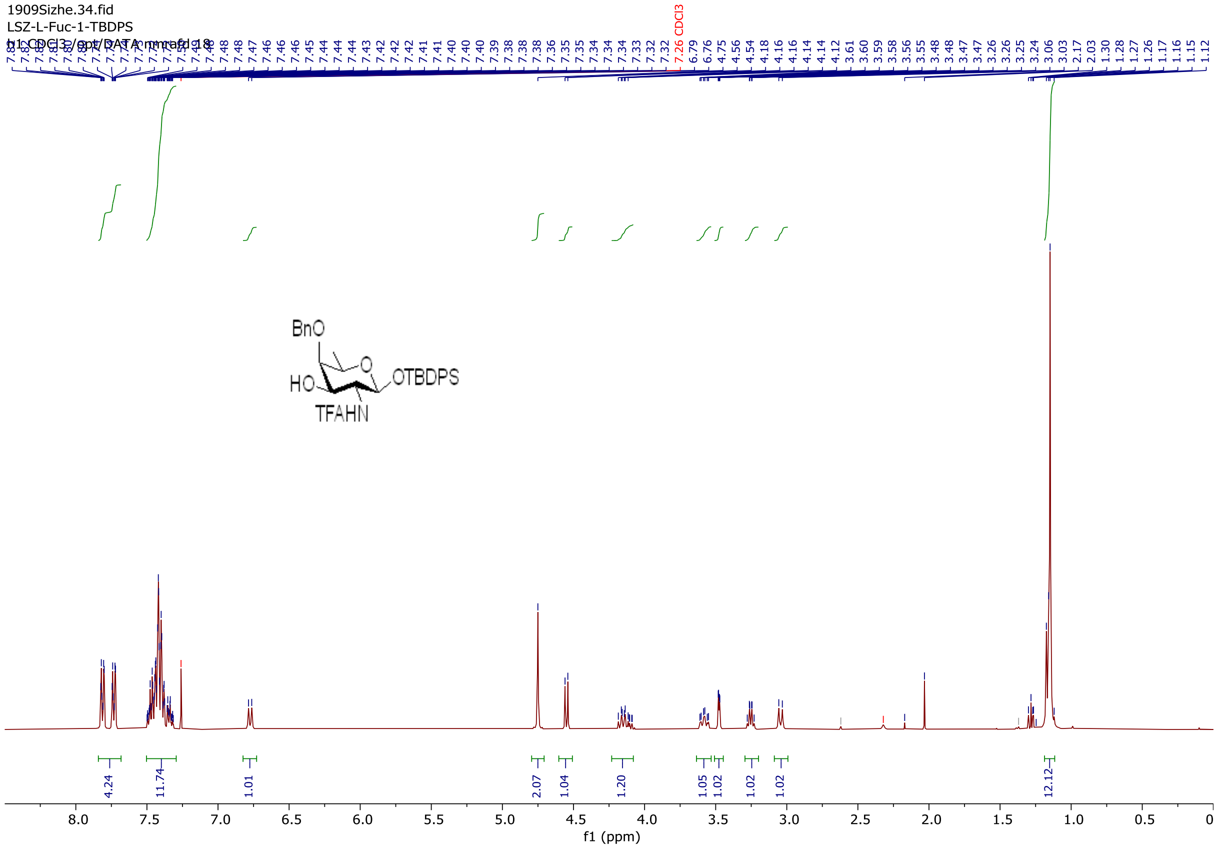

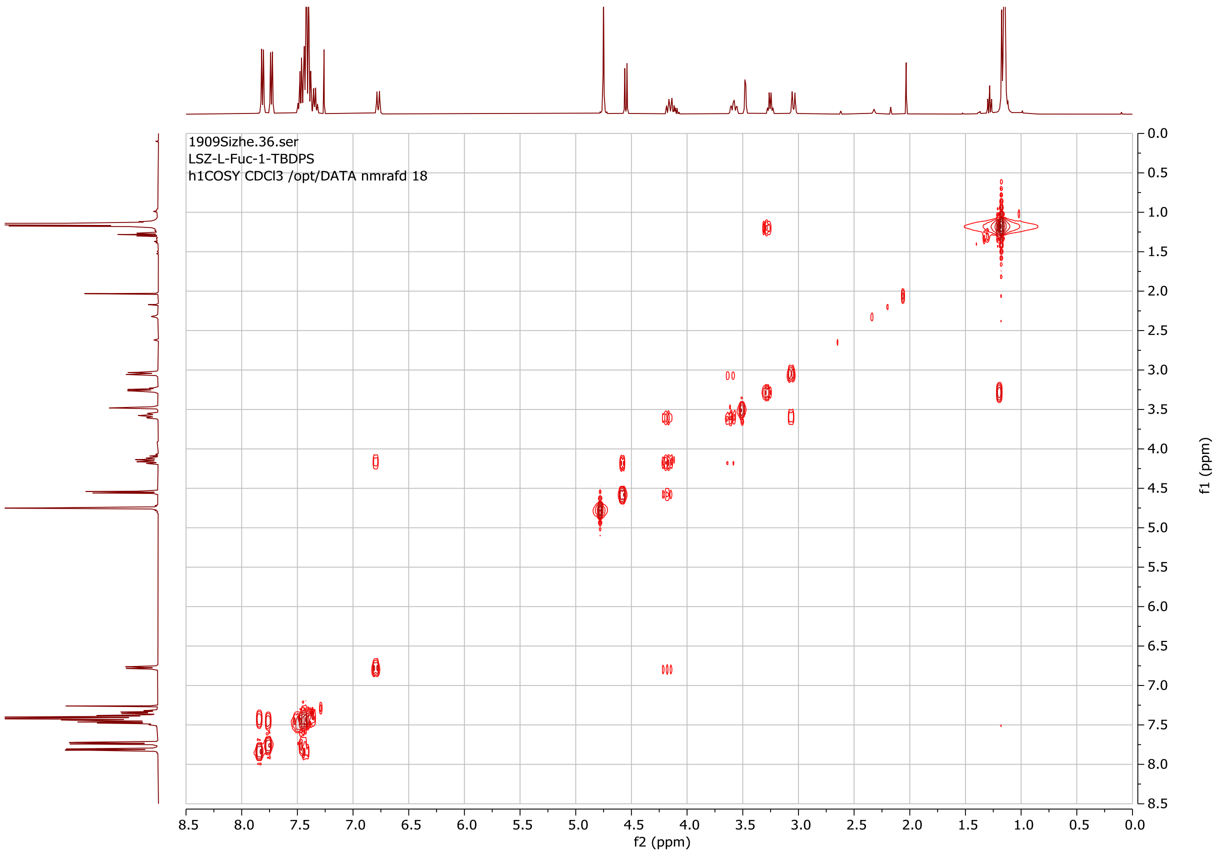
1909Sizhe.36.ser
LSZ-L-Fuc-1-TBDPS
h1COSY CDCl3 /opt/DATA nmrafd 18
f2 (ppm)
f1 (ppm)

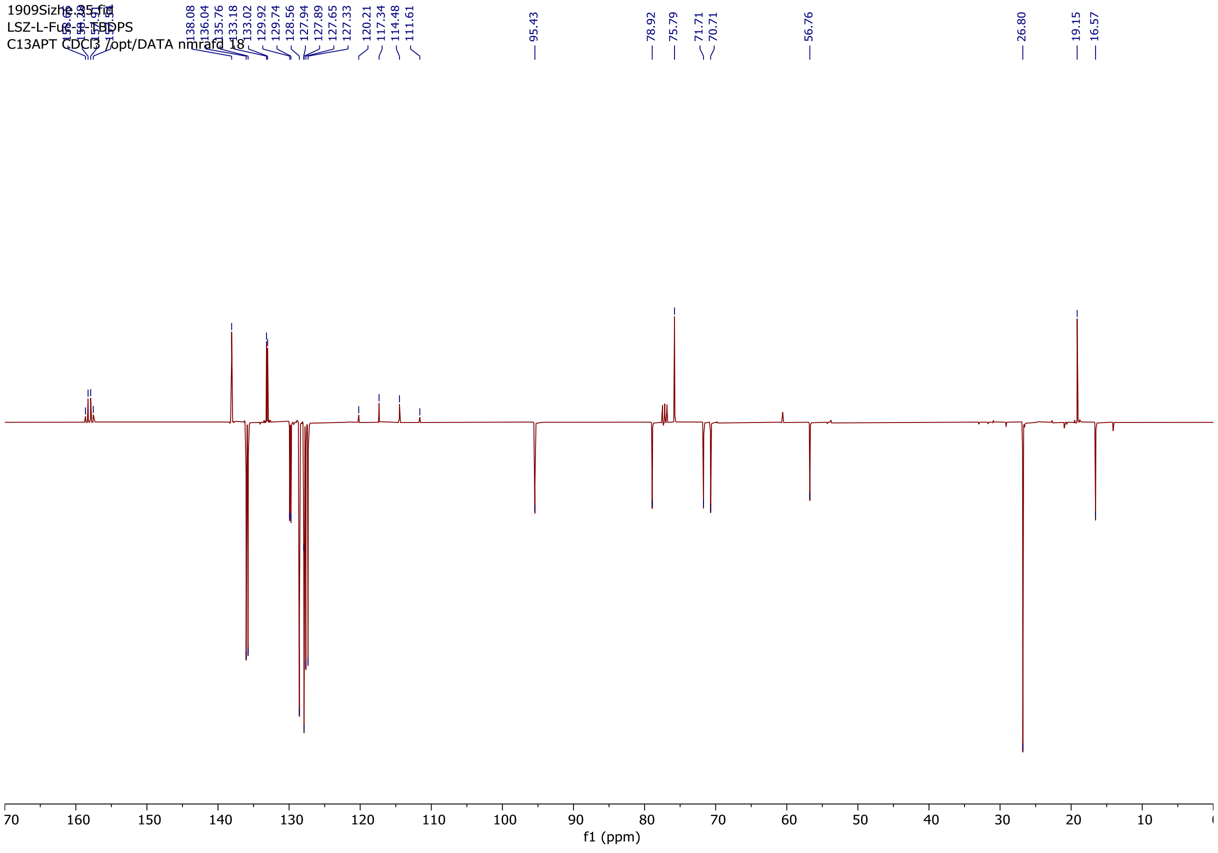
1909Sizhe.35.fid
LSZ-L-Fuc-1-TBDPS
C13APT CDCl3 /opt/DATA nmrafd 18
138.08
136.04
135.76
133.18
133.02
129.92
129.74
128.56
127.94
127.89
127.65
127.33
120.21
117.34
114.48
111.61
95.43
78.92
75.79
71.71
70.71
56.76
26.80
19.15
16.57
f1 (ppm)

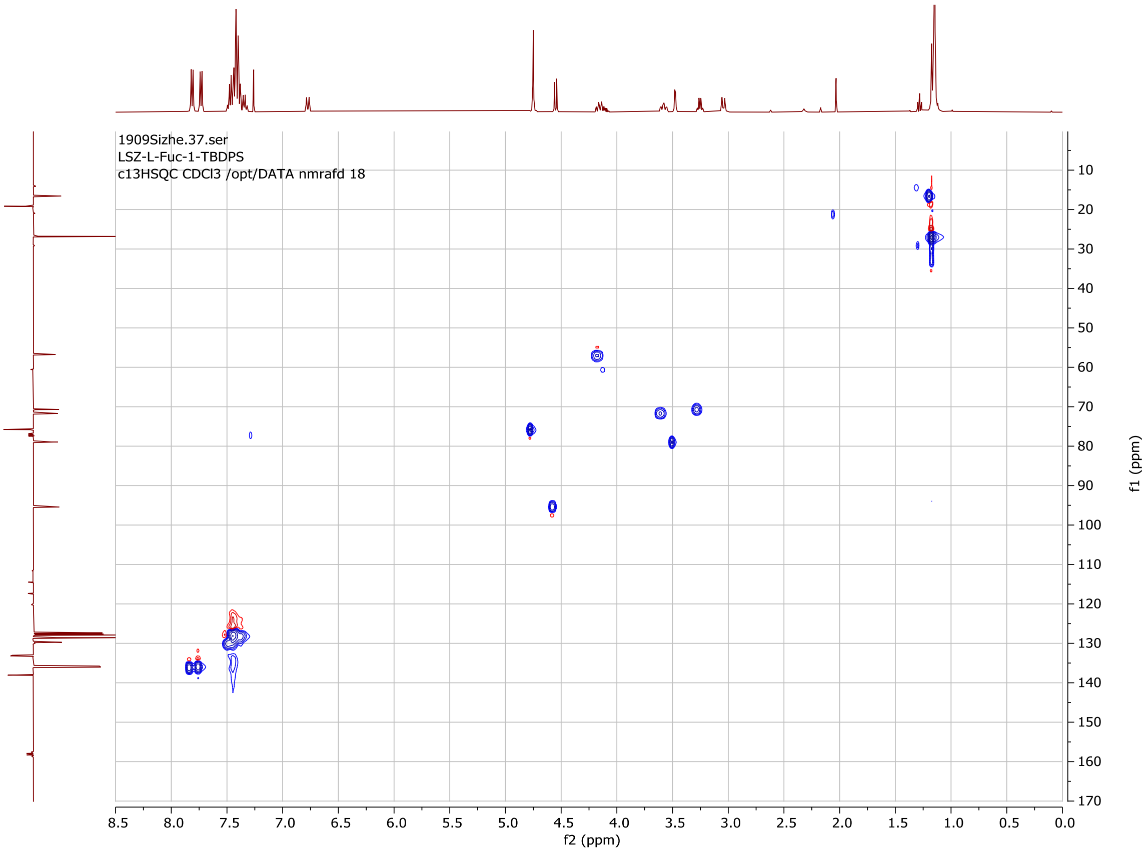

Phenyl 3,4-di-*O*-acetyl-2-azido-2-deoxy-1-thio-α/β-L-fucopyranoside (S15)

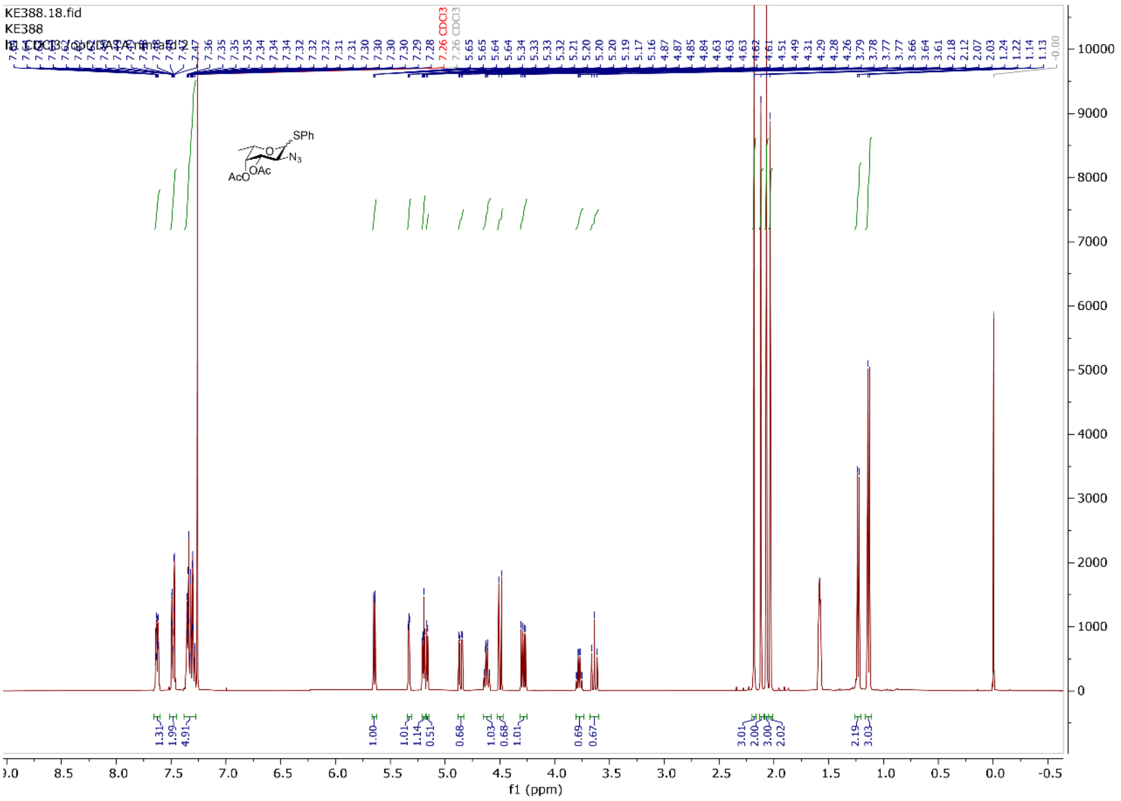
KE388.18.fid
KE388
SPh
N3
AcO
OAc
f1 (ppm)

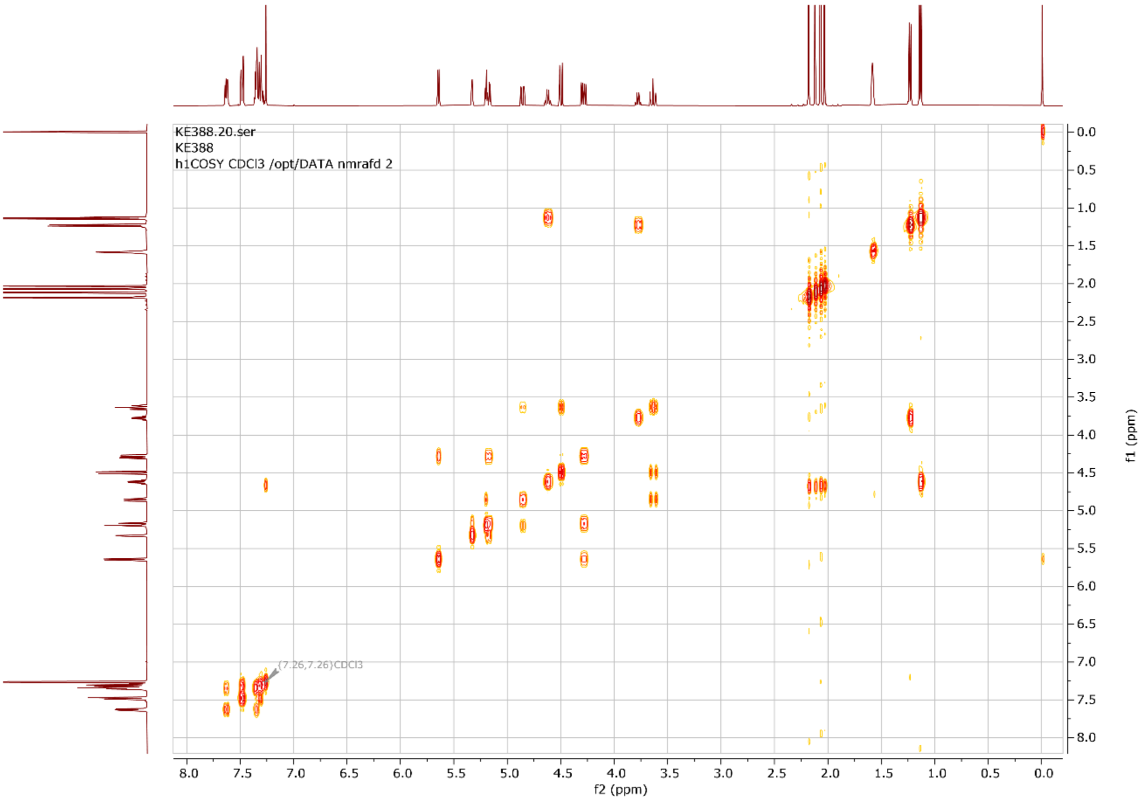
KE388.20.ser
KE388
h1COSY CDCl3 /opt/DATA nmrafd 2
(7.26,7.26)CDCl3
f2 (ppm)
f1 (ppm)

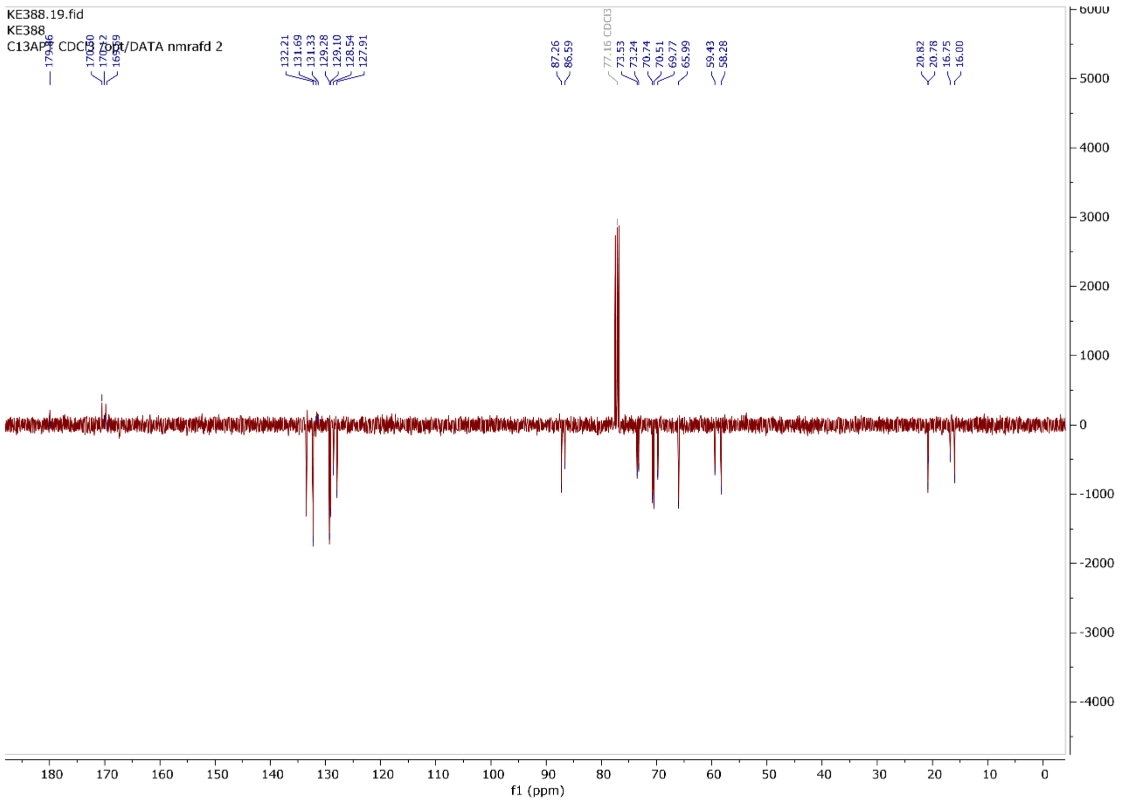
KE388.19.fid
KE388
C13APT CDCl3 /opt/DATA nmrafd 2
179.86
170.50
169.59
132.21
131.69
131.33
129.28
129.10
128.54
127.91
87.26
86.59
77.16 CDCl3
73.53
73.24
70.74
70.51
69.77
65.99
59.43
58.28
20.82
20.78
16.75
16.00
f1 (ppm)

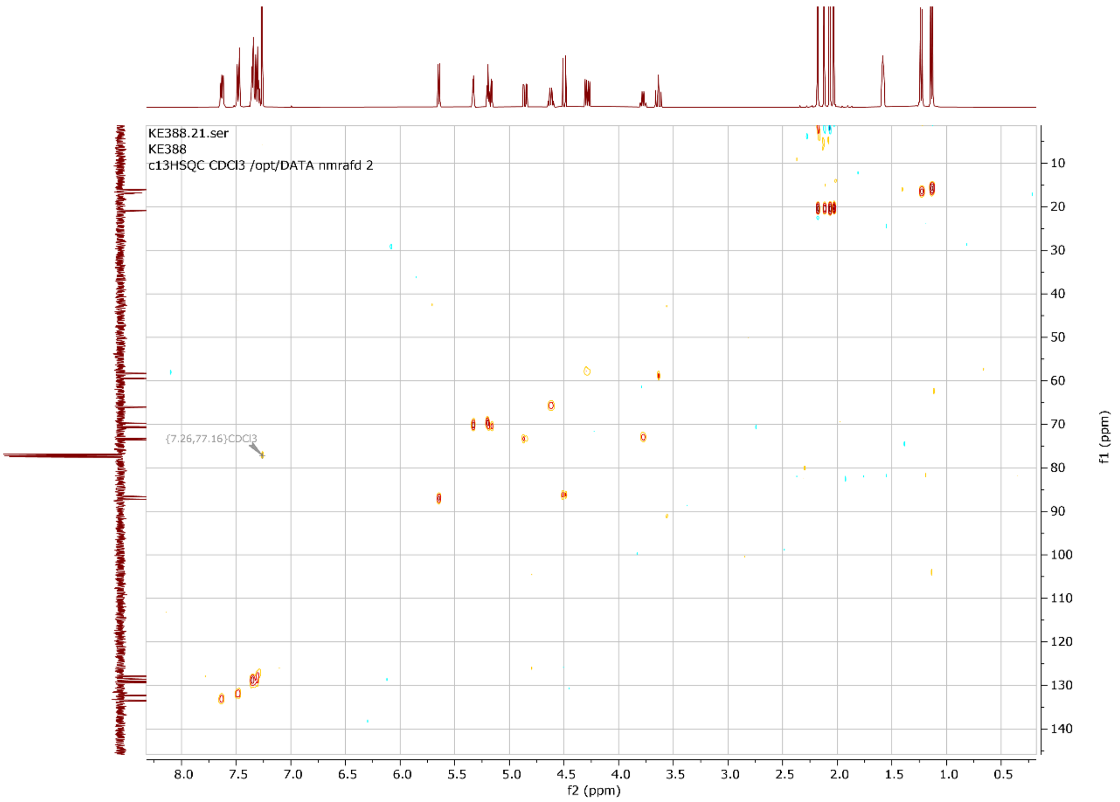
KE388.21.ser
KE388
c13HSQC CDCl3 /opt/DATA nmrafd 2
{7.26,77.16}CDCl3
f2 (ppm)
f1 (ppm)

Phenyl 2-azido-2-deoxy-1-thio-α/β-L-fucopyranoside (S16)

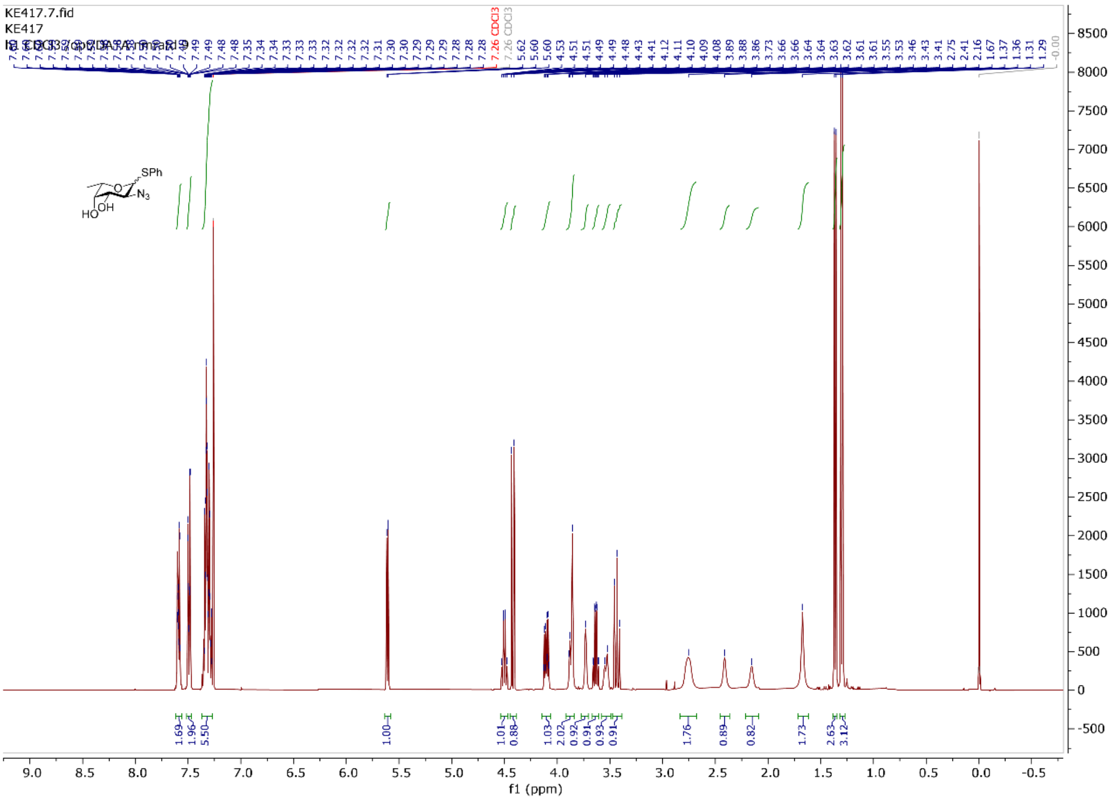

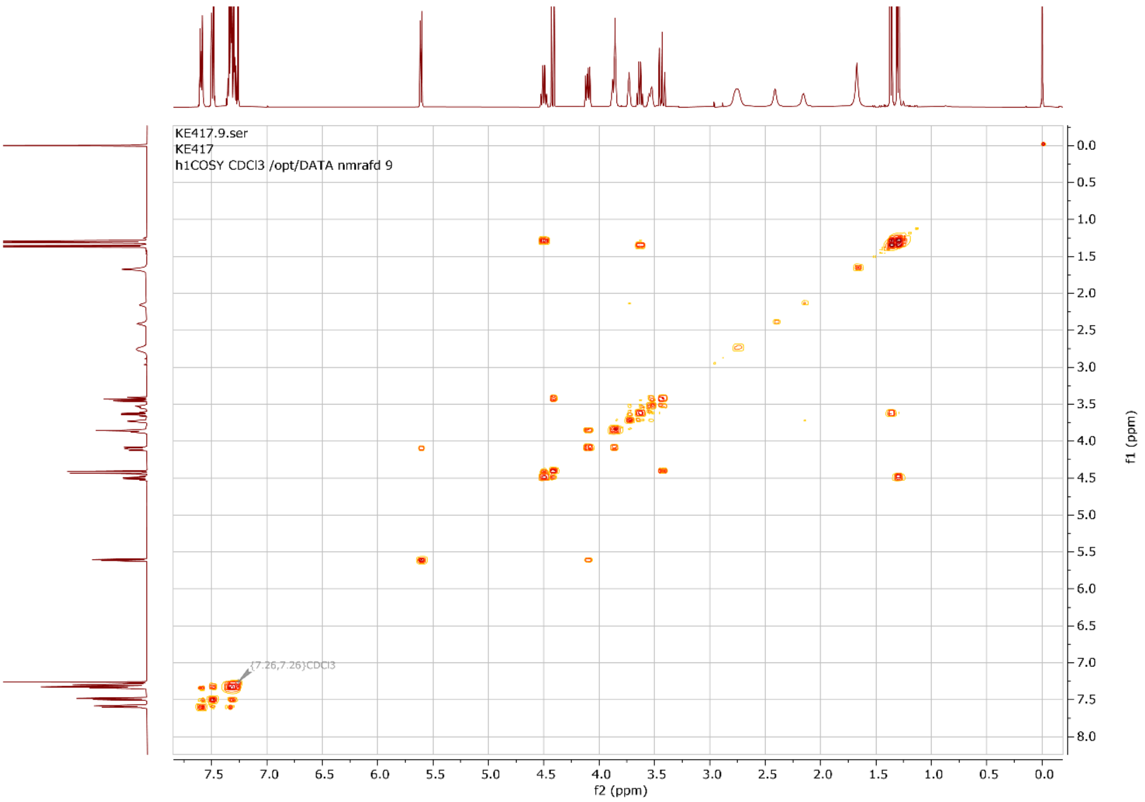

KE417.9.ser
KE417
h1COSY CDCl3 /opt/DATA nmrafd 9
(7.26,7.26)CDCl3
f2 (ppm)
f1 (ppm)

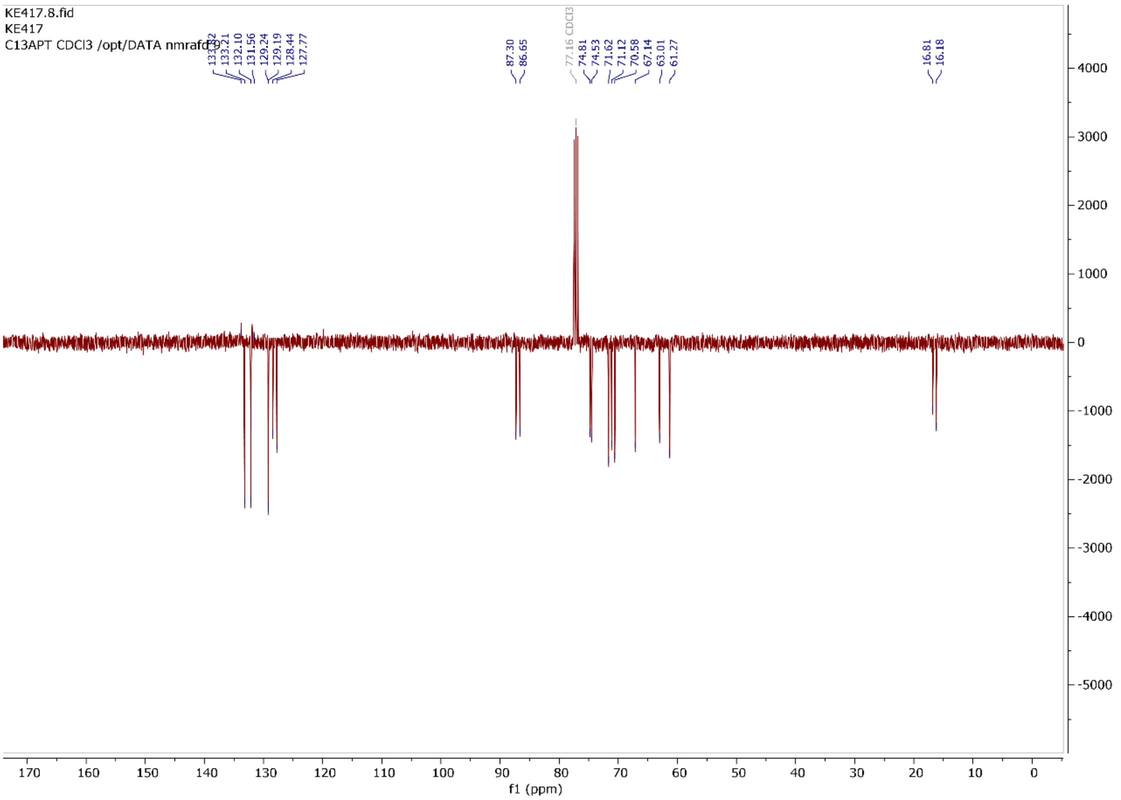

KE417.8.fid
KE417
C13APT CDCl3 /opt/DATA nmrafd 9
133.32
133.21
132.10
131.56
129.24
129.19
128.44
127.77
87.30
86.65
77.16 CDCl3
74.81
74.53
71.62
71.12
70.58
67.14
63.01
61.27
16.81
16.18
f1 (ppm)

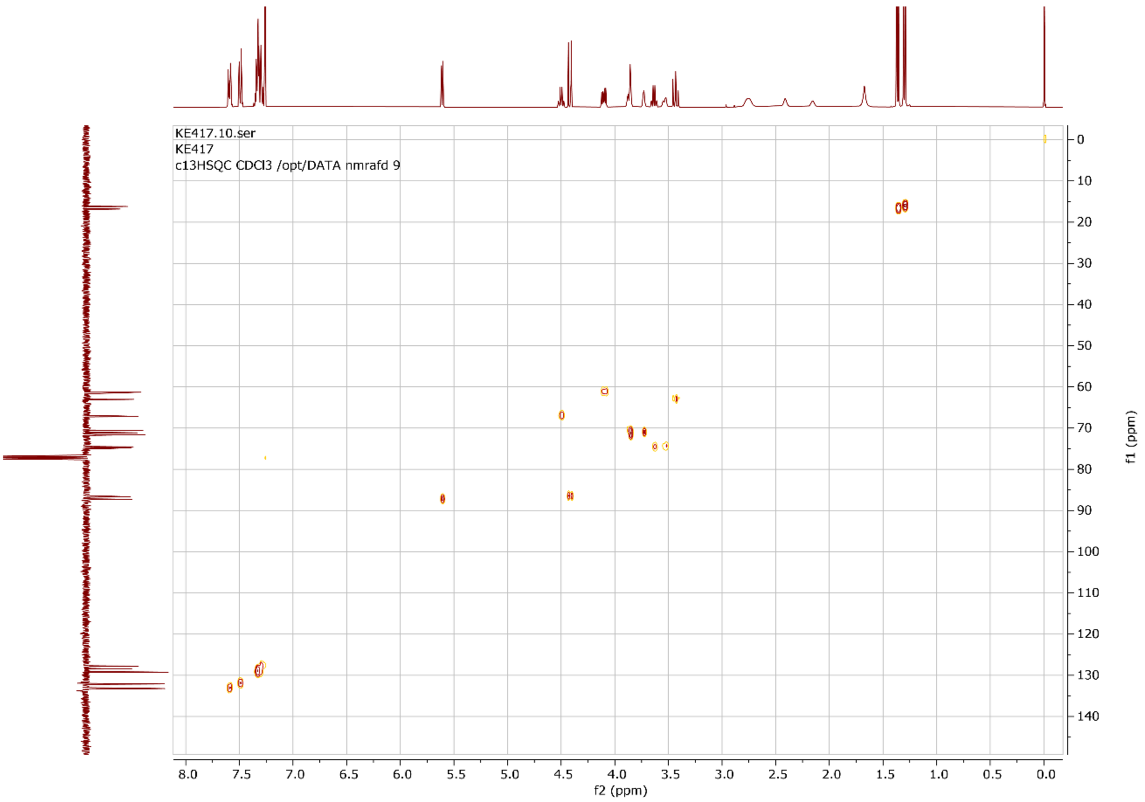

Phenyl 2-azido-2-deoxy-3-*O*-(2-naphthylmethyl)-1-thio-α/β-L-fucopyranoside (S17)

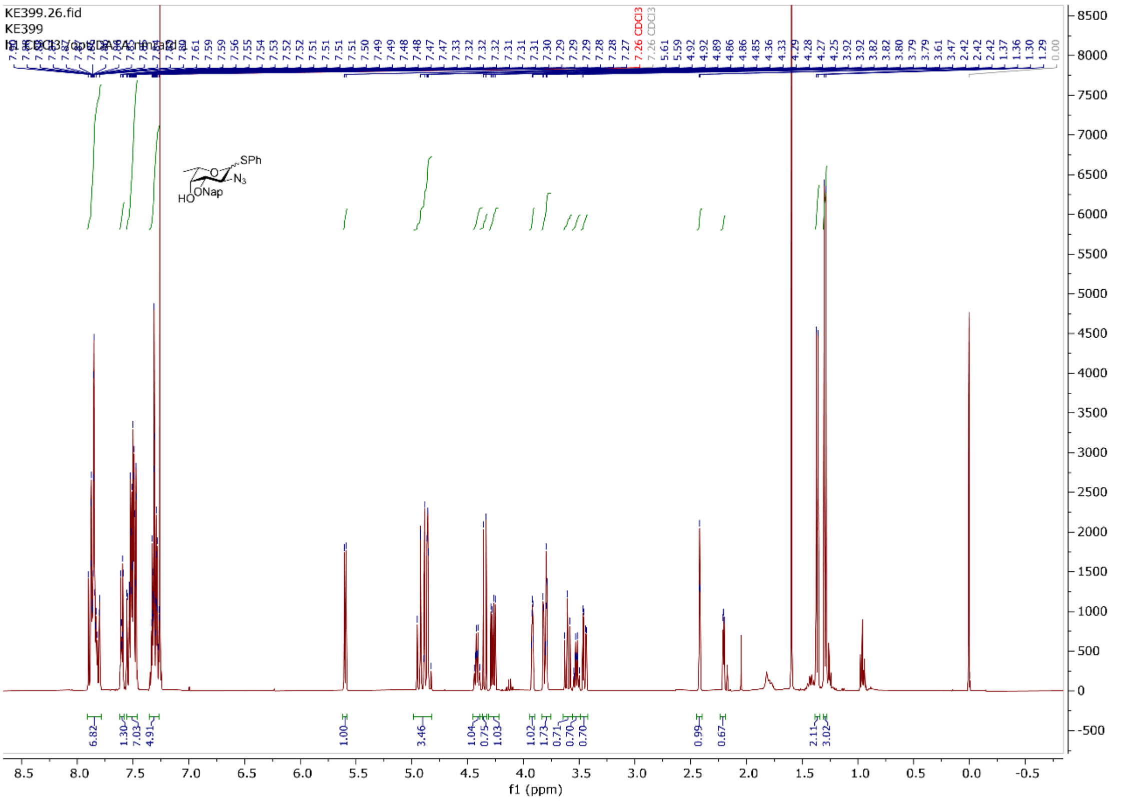
KE399.26.fid
KE399
SPh
N3
HO
ONap
f1 (ppm)

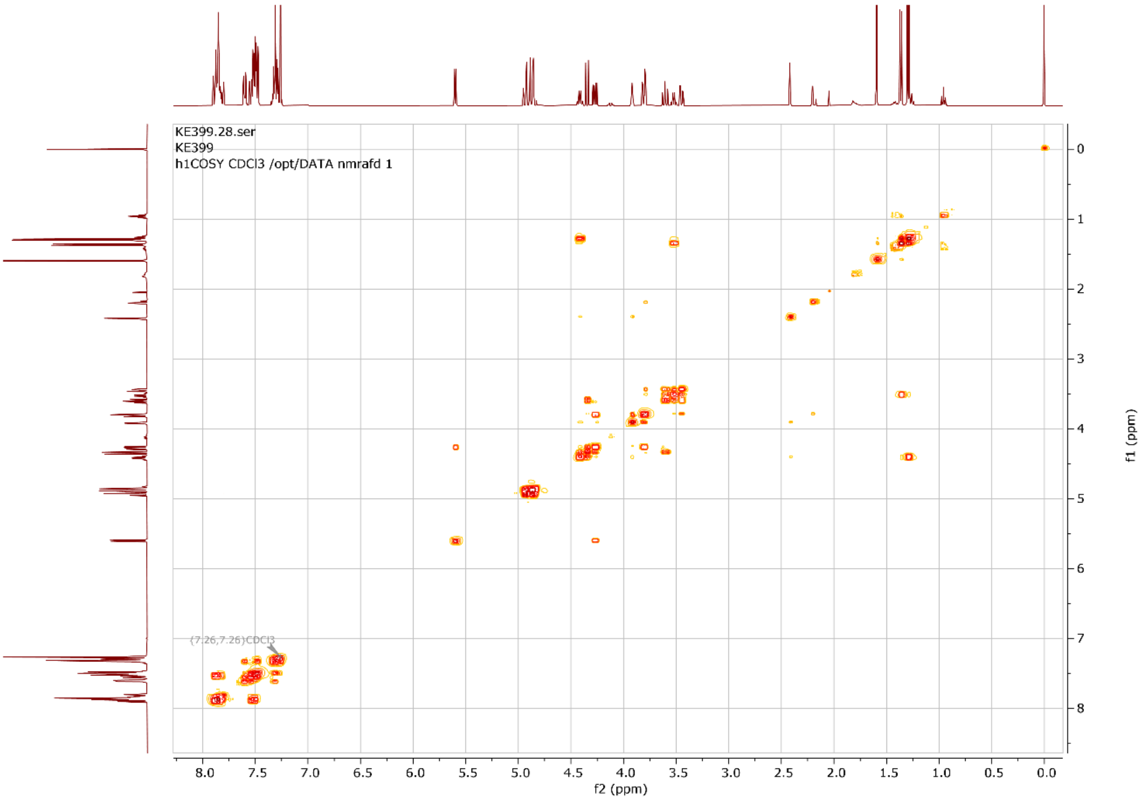
KE399.28.ser
KE399
h1COSY CDCl3 /opt/DATA nmrafd 1
f2 (ppm)
f1 (ppm)

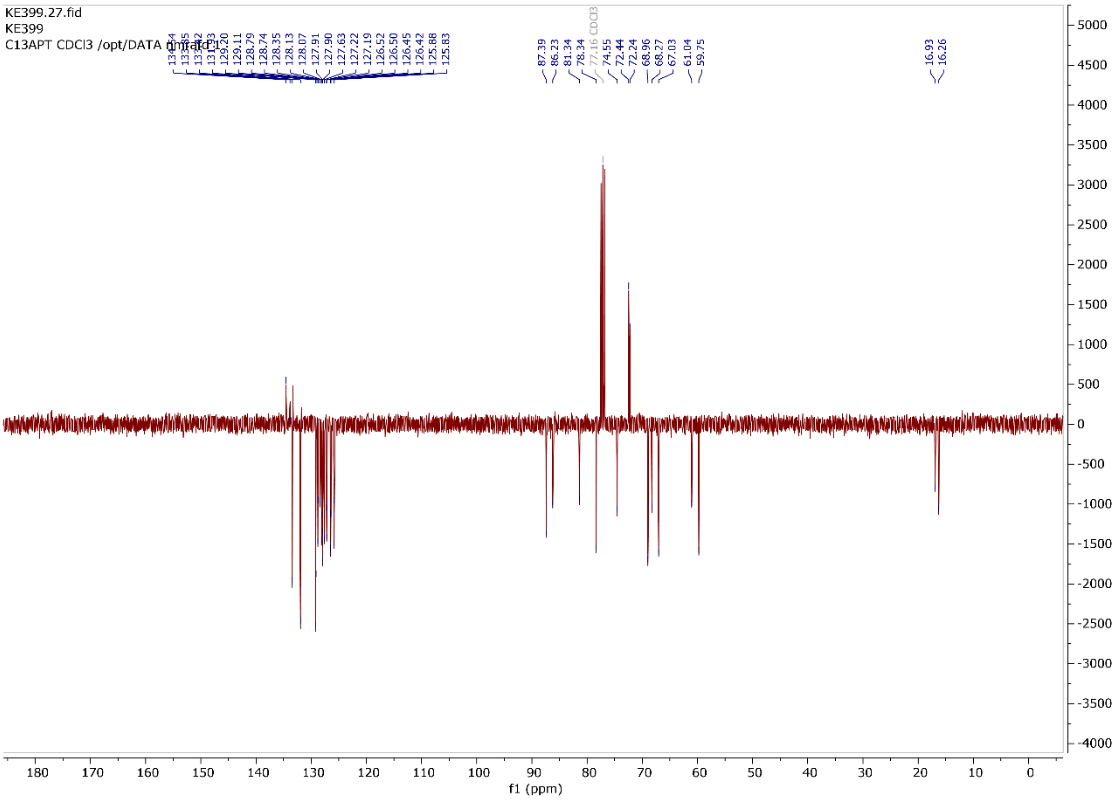
KE399.27.fid
KE399
C13APT CDCl3 /opt/DATA nmrafd 1
131.93
129.20
129.11
128.79
128.74
128.35
128.13
128.07
127.91
127.90
127.63
127.22
127.19
126.52
126.50
126.45
126.42
125.88
125.83
87.39
86.23
81.34
78.34
77.16 CDCl3
74.55
72.44
72.24
68.96
68.27
67.03
61.04
59.75
16.93
16.26
f1 (ppm)

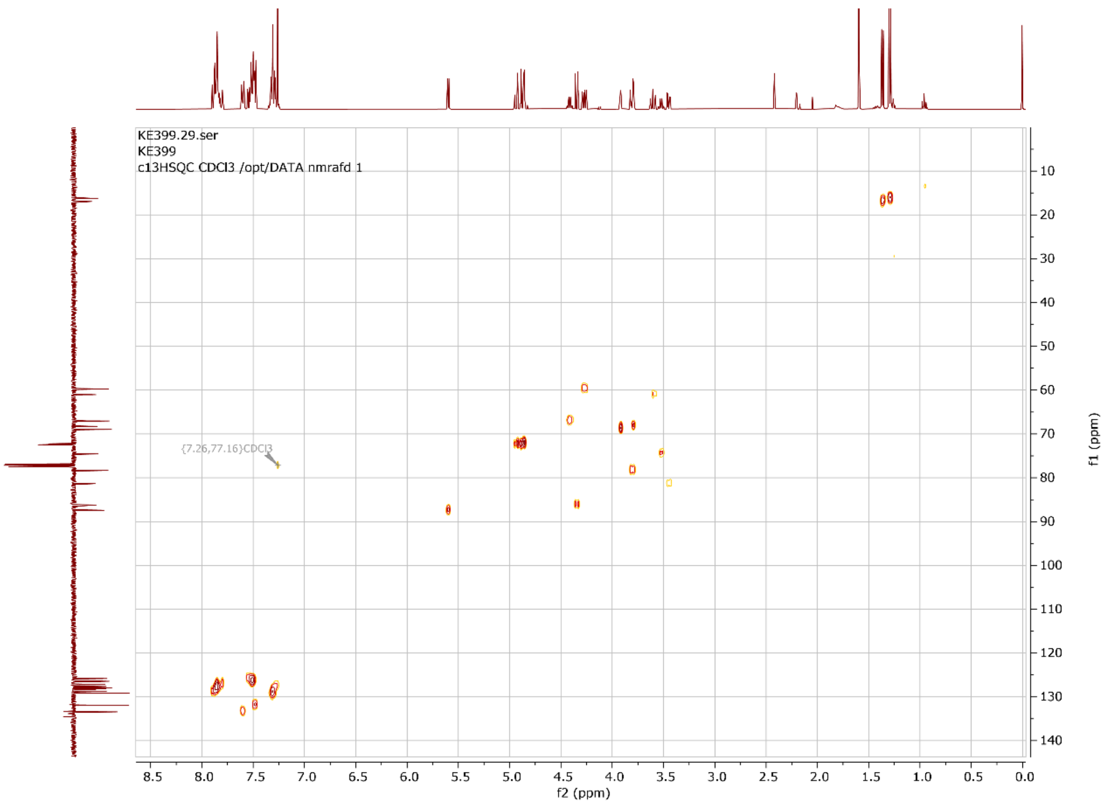
KE399.29.ser
KE399
c13HSQC CDCl3 /opt/DATA nmrafd 1
{7.26,77.16}CDCl3
f2 (ppm)
f1 (ppm)

Phenyl 2-azido-4-*O*-benzoyl-2-deoxy-3-*O*-(2-naphthylmethyl)-1-thio-α-L-fucopyranoside (12a)

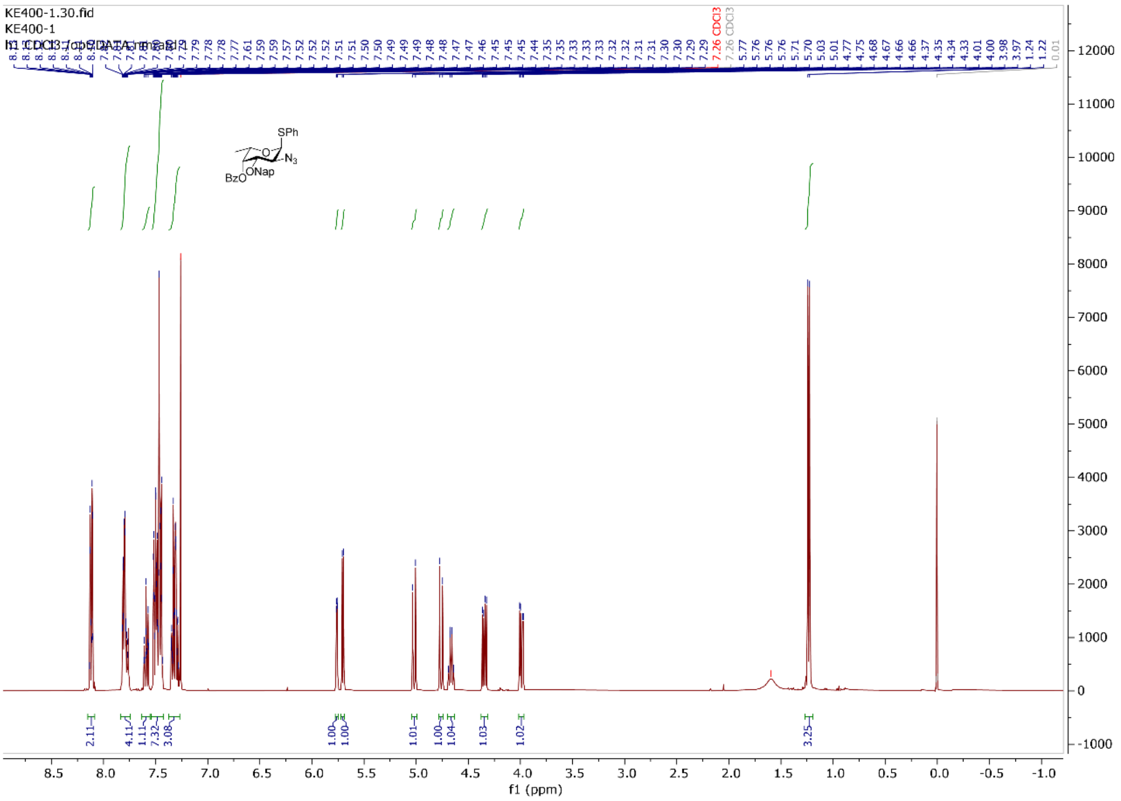
KE400-1.30.fid
KE400-1
SPh
N3
BzO
ONap
f1 (ppm)

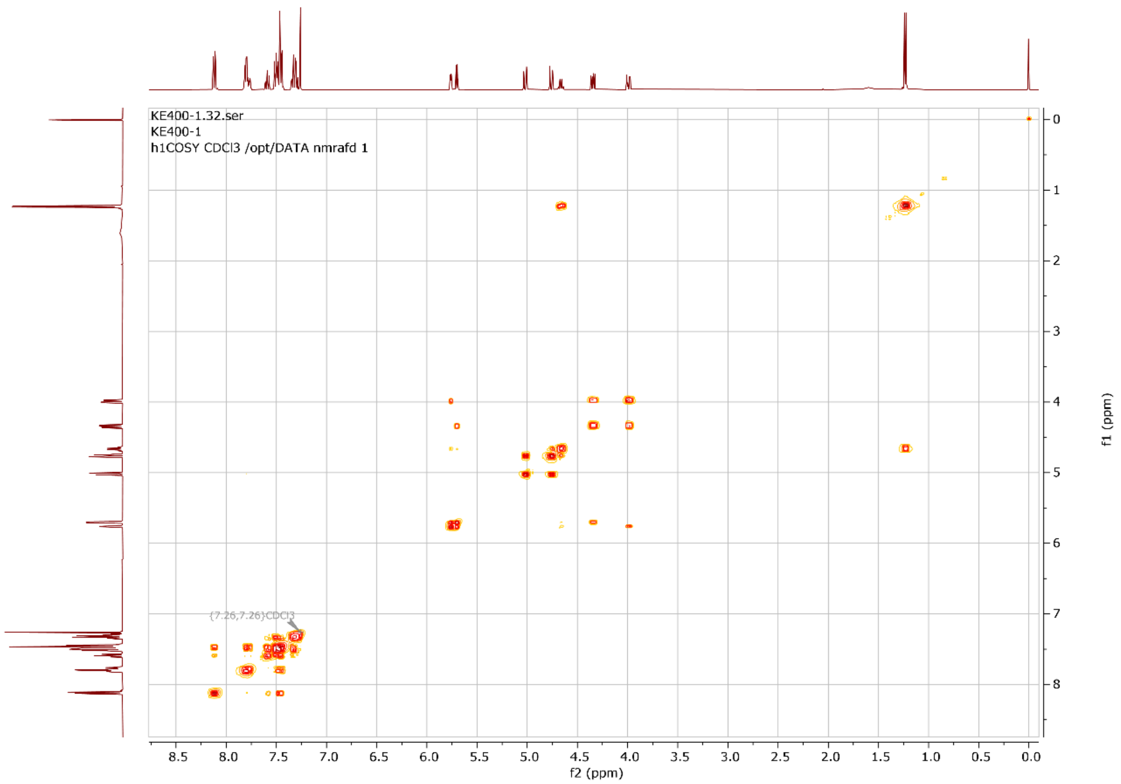
KE400-1.32.ser
KE400-1
h1COSY CDCl3 /opt/DATA nmrafd 1
(7.26,7.26)CDCl3
f2 (ppm)
f1 (ppm)

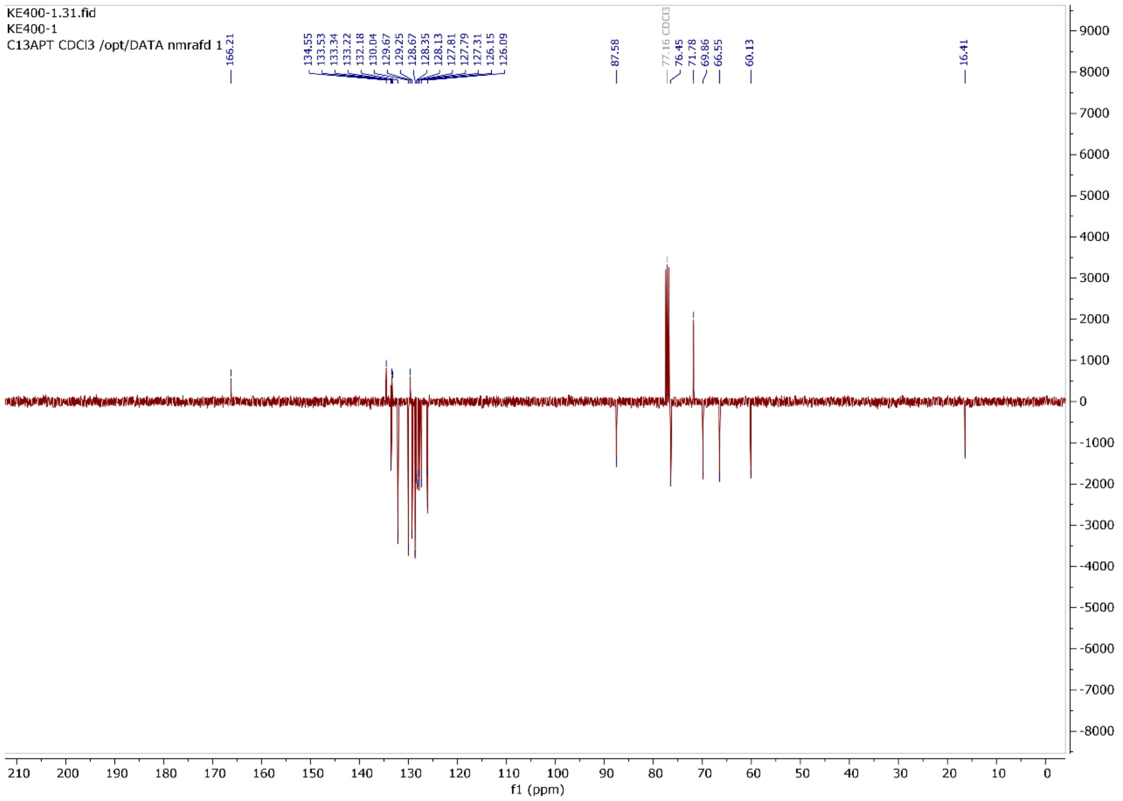
KE400-1.31.fid
KE400-1
C13APT CDCl3 /opt/DATA nmrafd 1
166.21
134.55
133.53
133.34
133.22
132.18
130.04
129.67
129.25
128.67
128.35
128.13
127.81
127.79
127.31
126.15
126.09
87.58
77.16 CDCl3
76.45
71.78
69.86
66.55
60.13
16.41
f1 (ppm)

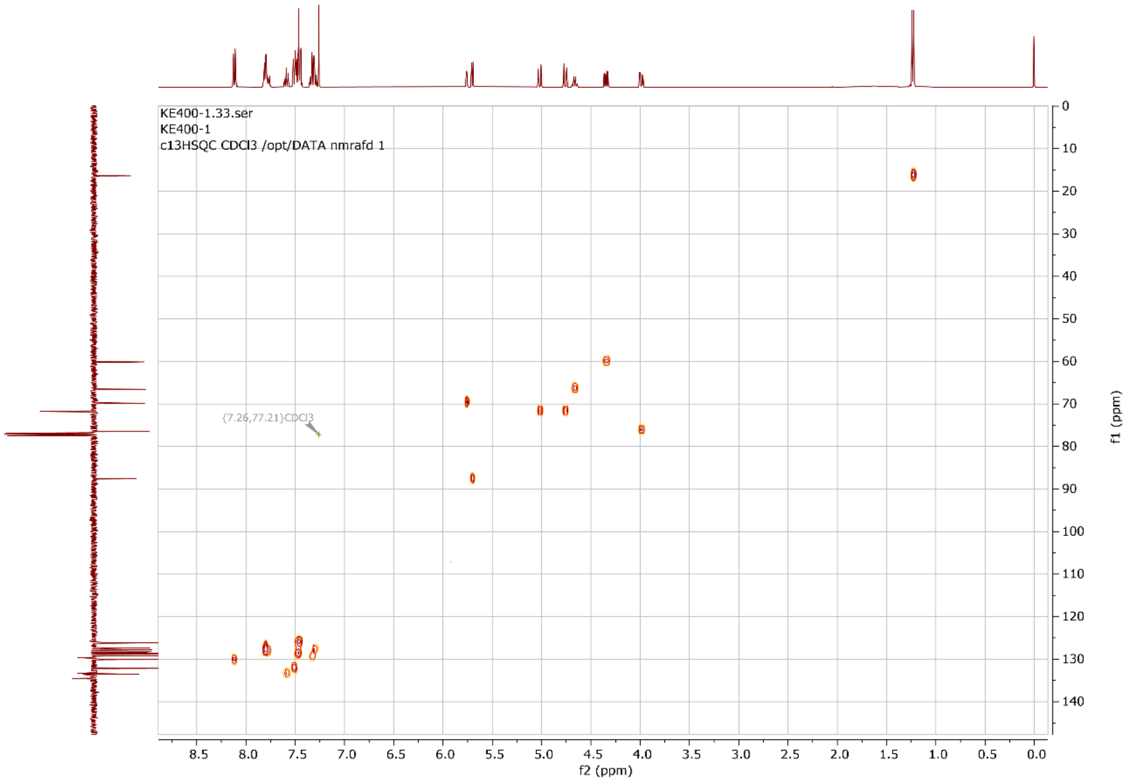
KE400-1.33.ser
KE400-1
c13HSQC CDCl3 /opt/DATA nmrafd 1
{7.26,77.21}CDCl3
f2 (ppm)
f1 (ppm)

Phenyl 2-azido-4-*O*-benzoyl-2-deoxy-3-*O*-(2-naphthylmethyl)-1-thio-β-L-fucopyranoside (12a)

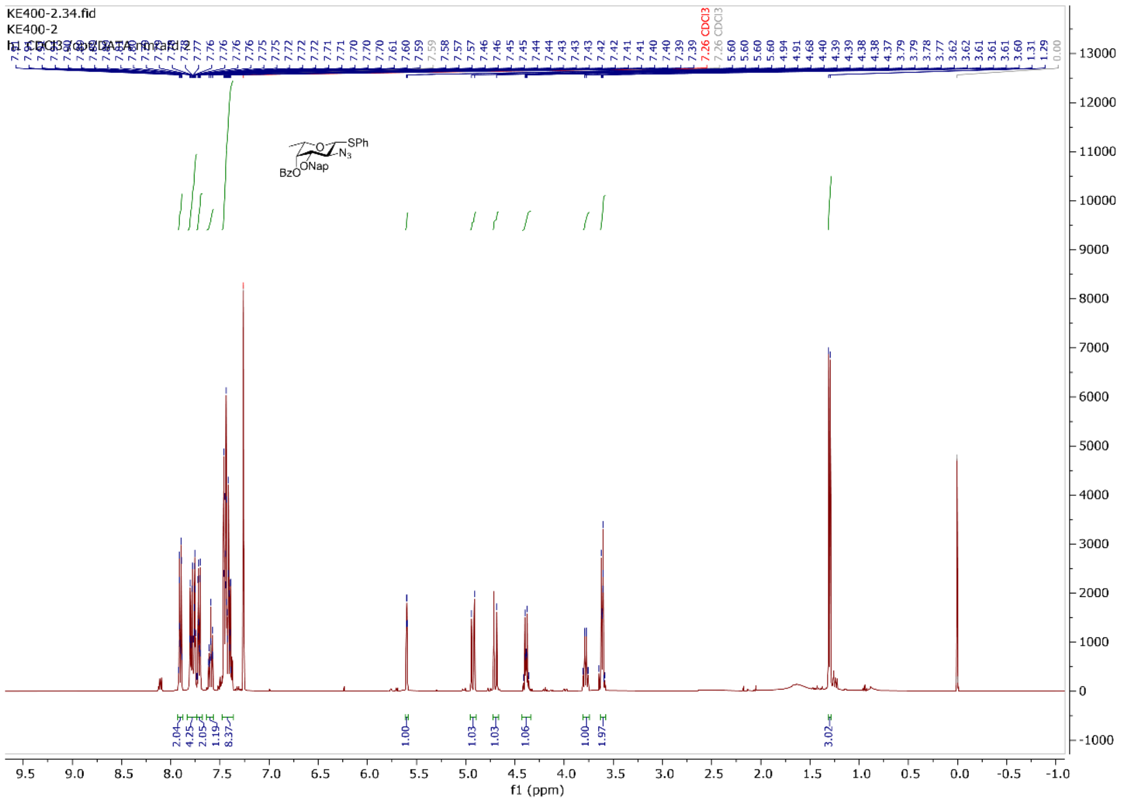

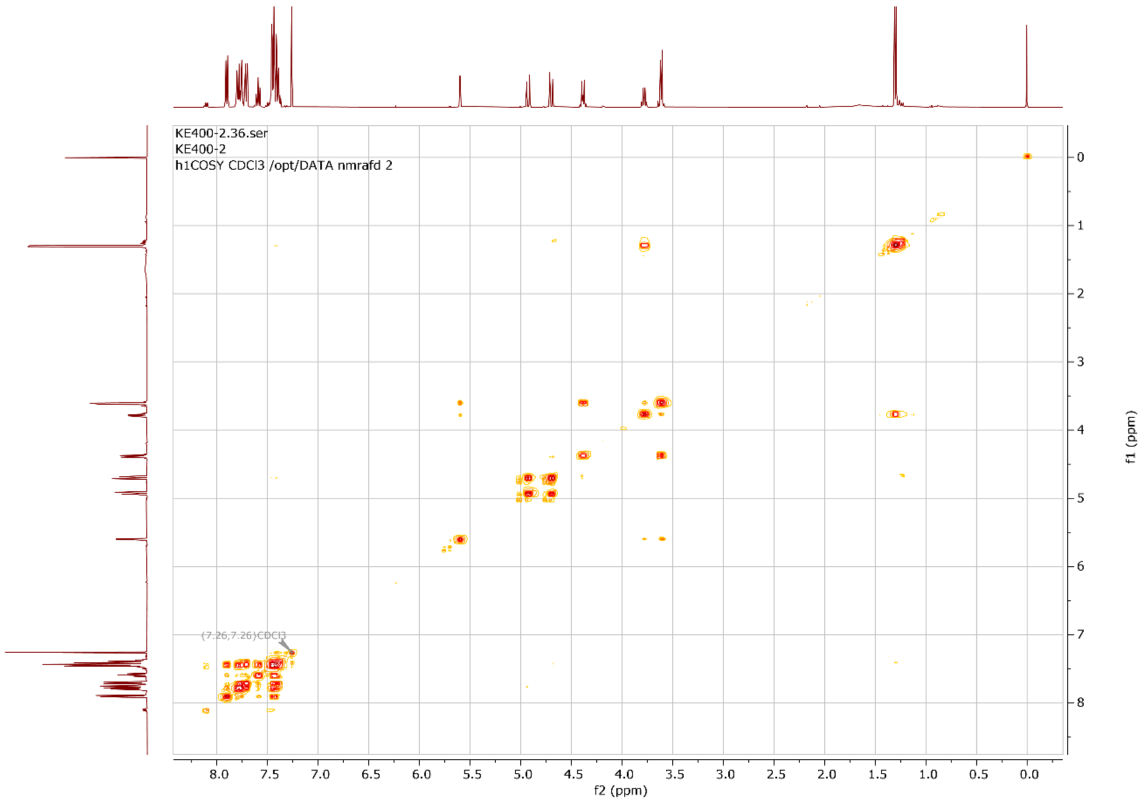
KE400-2.36.ser
KE400-2
h1COSY CDCl3 /opt/DATA nmrafd 2
(7.26,7.26)CDCl3
f2 (ppm)
f1 (ppm)

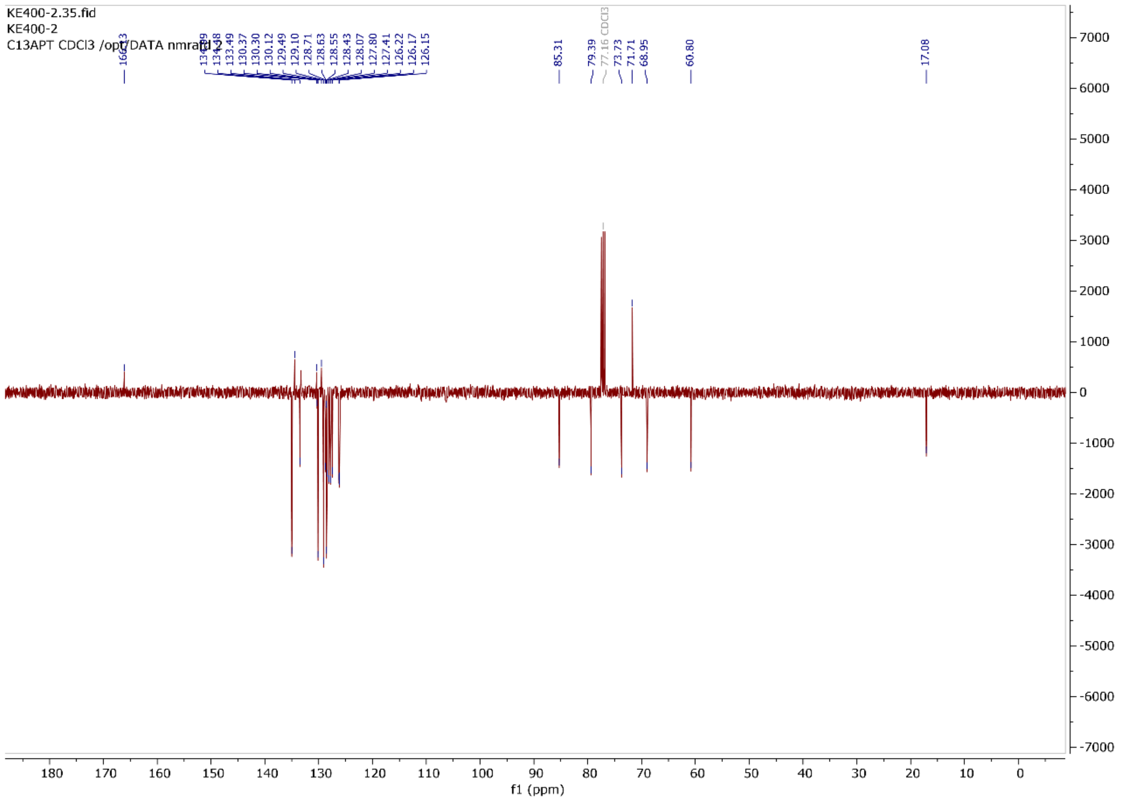

KE400-2.35.fid
KE400-2
C13APT CDCl3 /opt/DATA nmrafd 2
134.48
133.49
130.37
130.30
130.12
129.49
129.10
128.71
128.63
128.55
128.43
128.07
127.80
127.41
126.22
126.17
126.15
85.31
79.39
77.16 CDCl3
73.73
71.71
68.95
60.80
17.08
f1 (ppm)

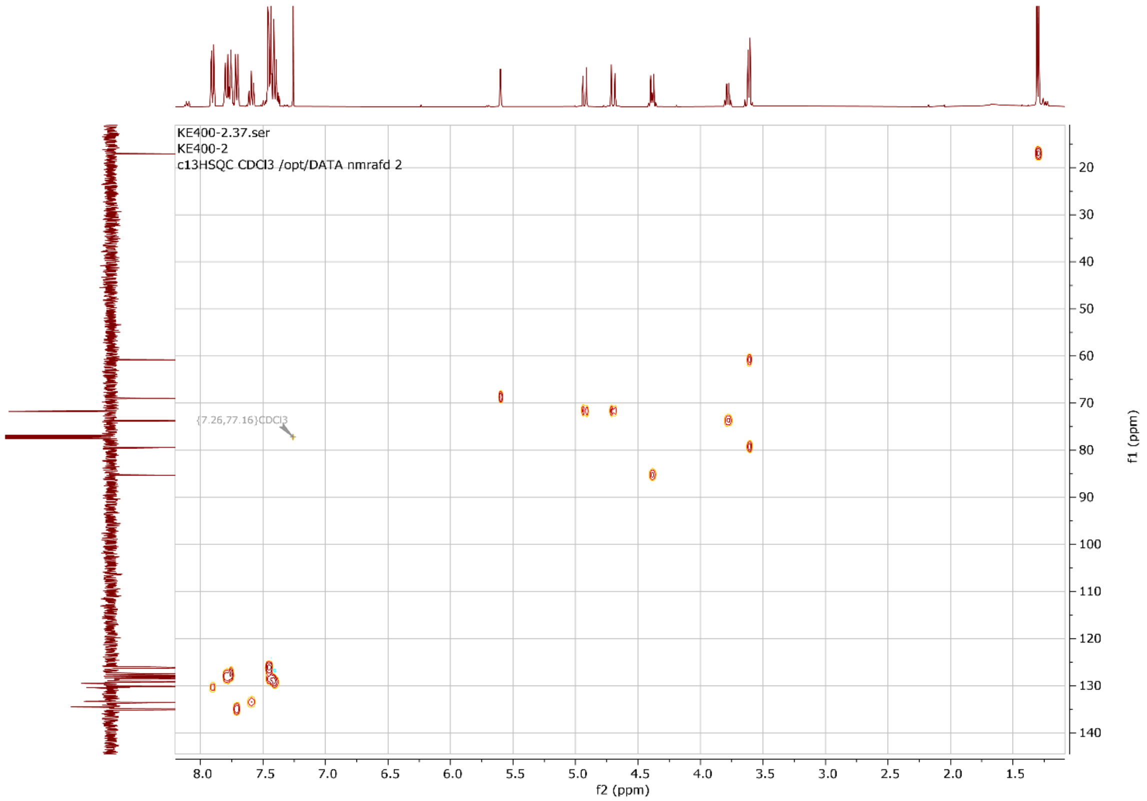

KE400-2.37.ser
KE400-2
c13HSQC CDCl3 /opt/DATA nmrafd 2
{7.26,77.16}CDCl3
f2 (ppm)
f1 (ppm)

1,3,4,6 Tetra-*O*-acetyl-α/β-D-mannopyranose (S18)

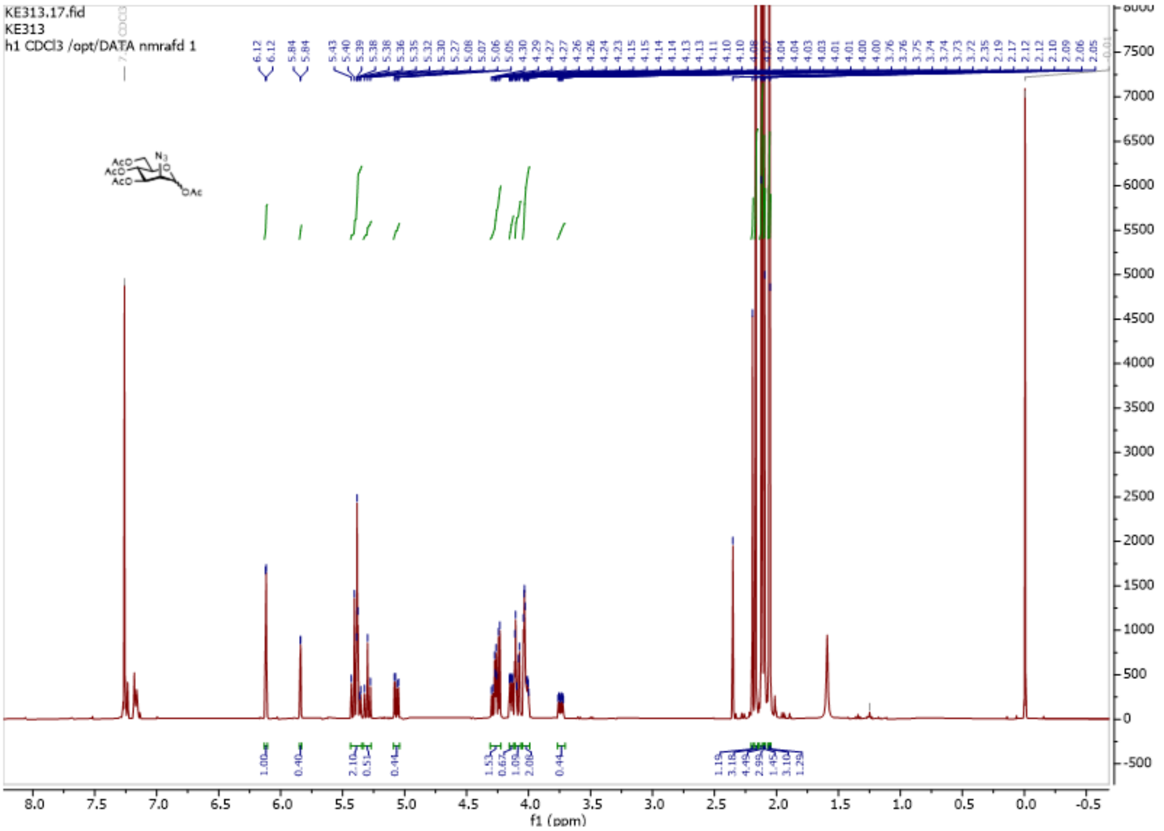

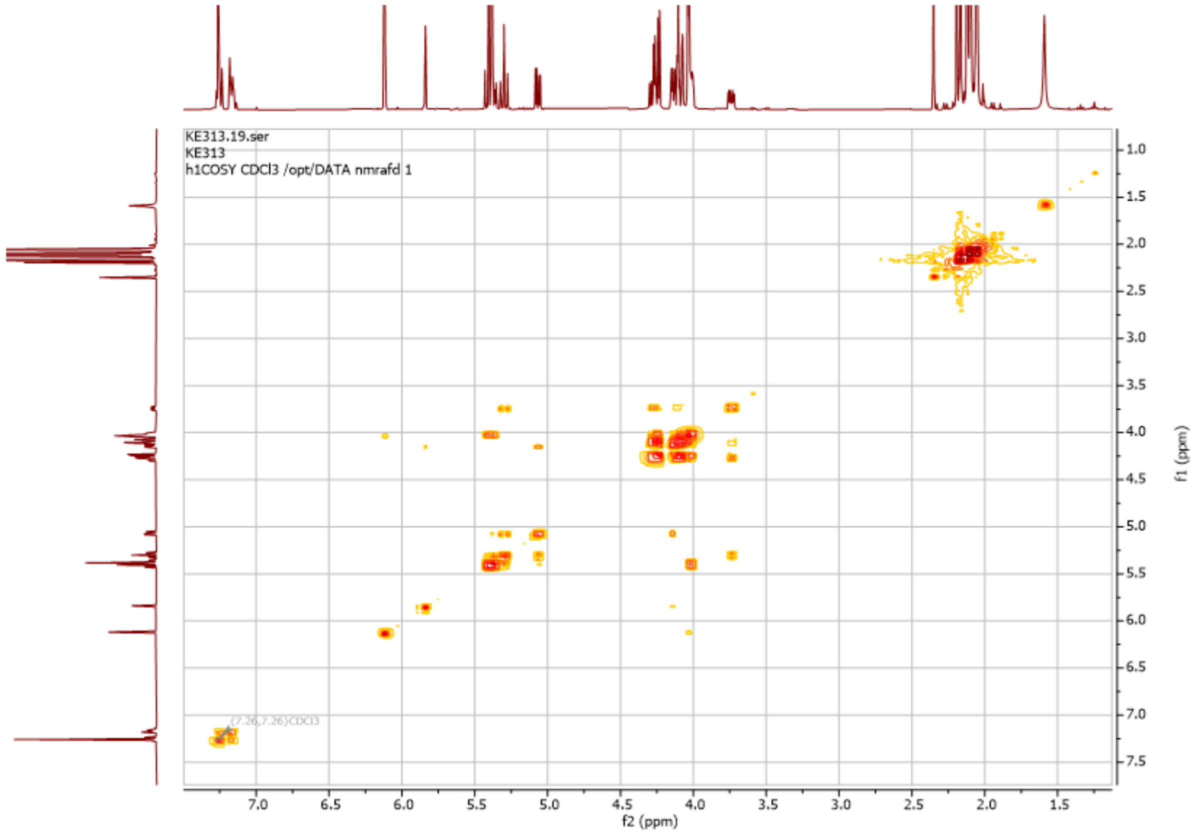
KE313.19.ser
KE313
h1COSY CDCl3 /opt/DATA nmrafd 1
f2 (ppm)
f1 (ppm)

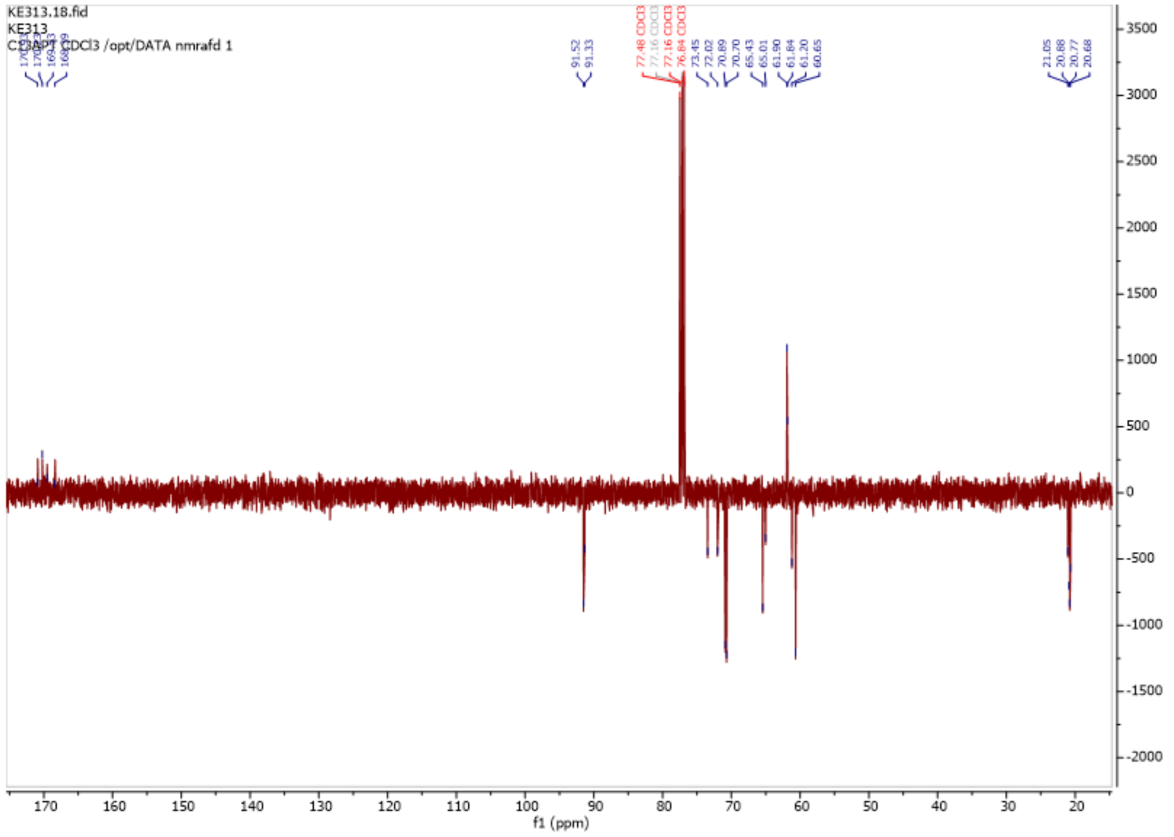
KE313.18.fid
KE313
C13APT CDCl3 /opt/DATA nmrafd 1
91.52
91.33
77.48 CDCl3
77.16 CDCl3
76.84 CDCl3
73.45
72.02
70.89
70.70
65.43
65.01
61.90
61.84
61.20
60.65
21.05
20.88
20.77
20.68
f1 (ppm)

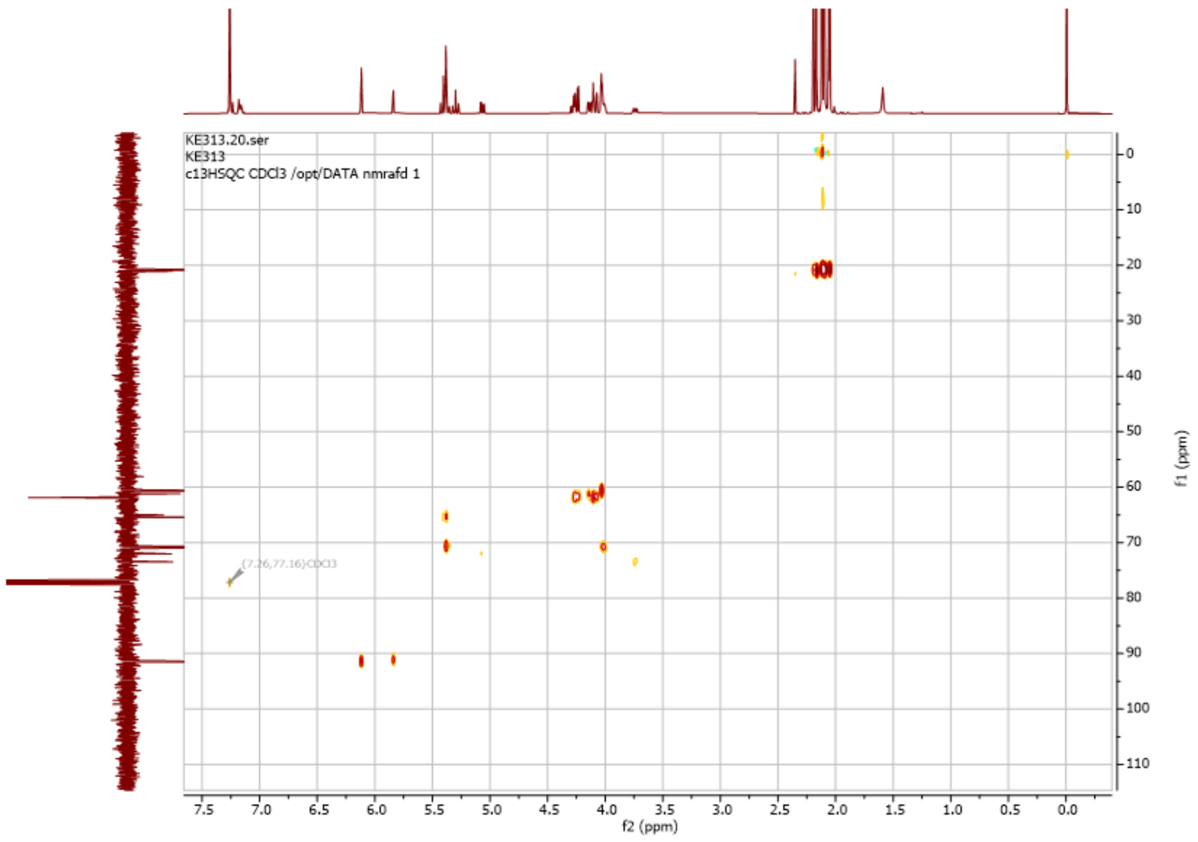

Phenyl 3,4,6 tri-*O*-acetyl-2-azido-2-deoxy-1-thio-α/β-D-mannopyranoside (S19)

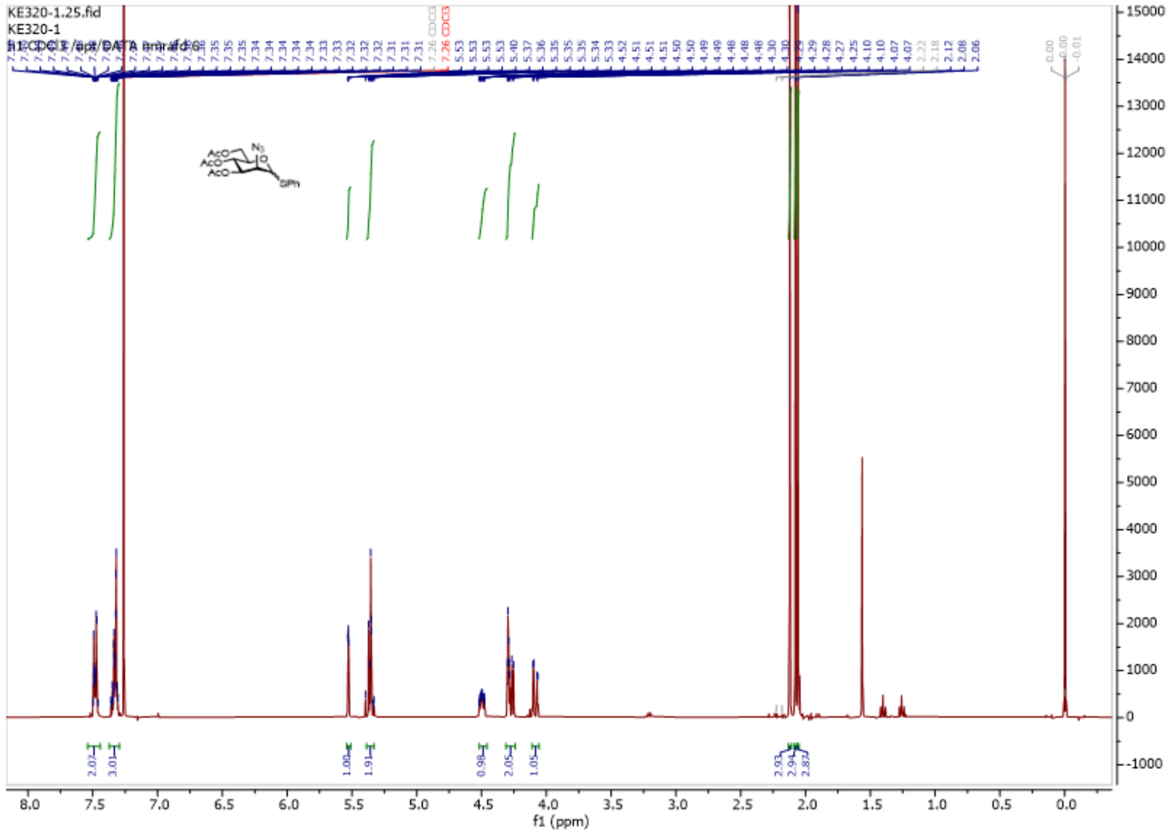
KE320-1.25.fid
KE320-1
f1 (ppm)

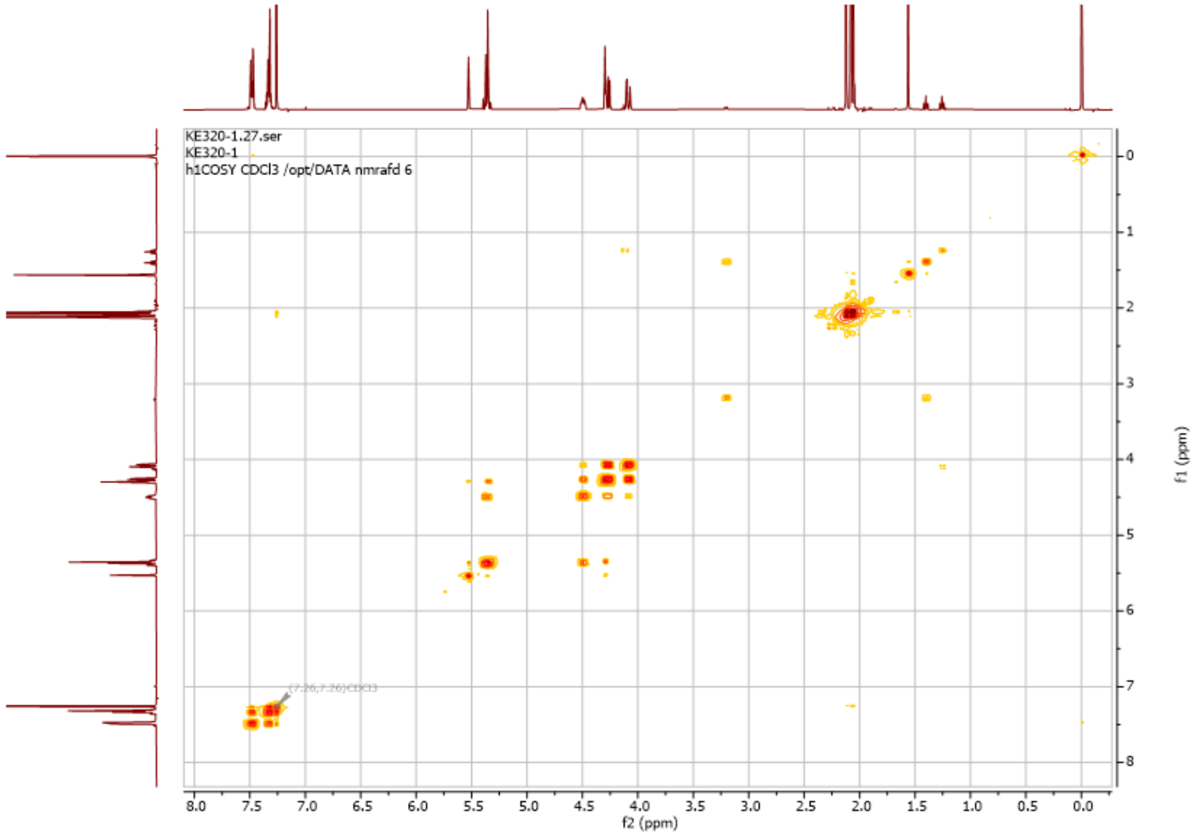
KE320-1.27.ser
KE320-1
h1COSY CDCl3 /opt/DATA nmrafd 6
f2 (ppm)
f1 (ppm)

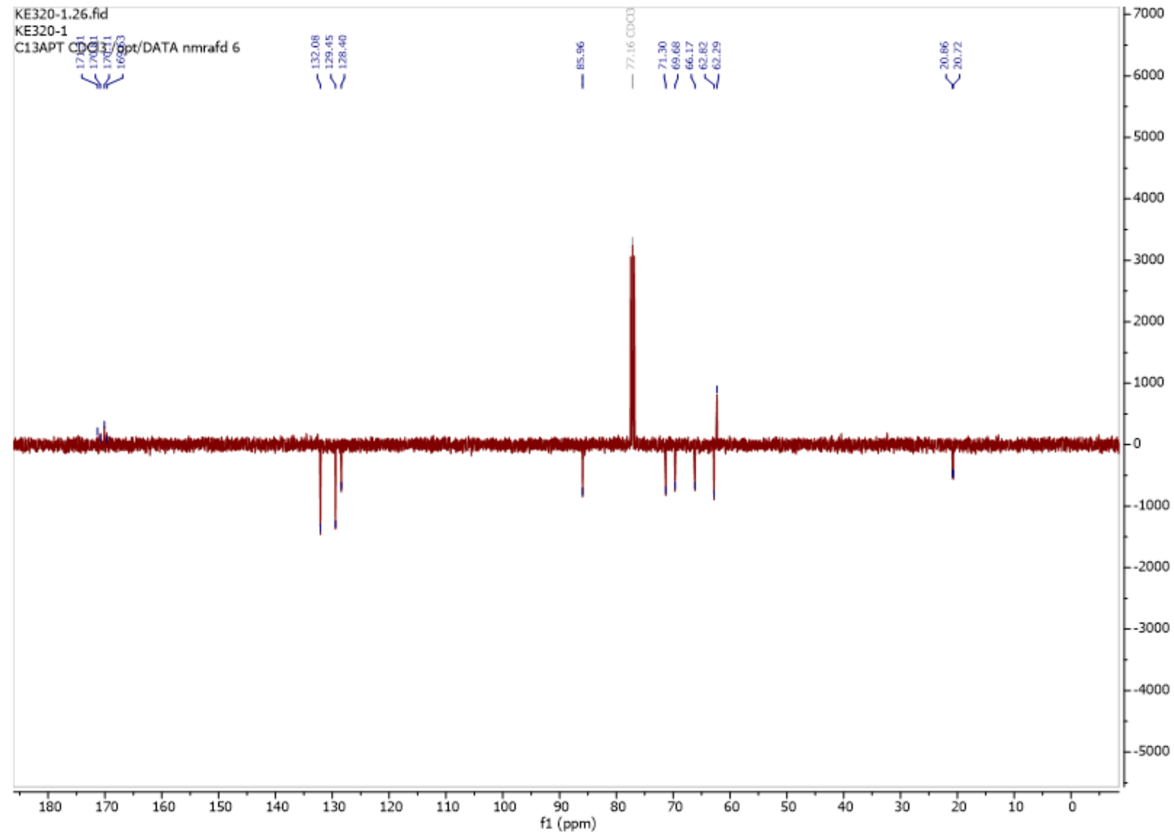
KE320-1.26.fid
KE320-1
C13APT CDCl3 /opt/DATA nmrafd 6
132.08
129.45
128.40
85.96
77.16 CDCl3
71.30
69.68
66.17
62.82
62.29
20.86
20.72
f1 (ppm)

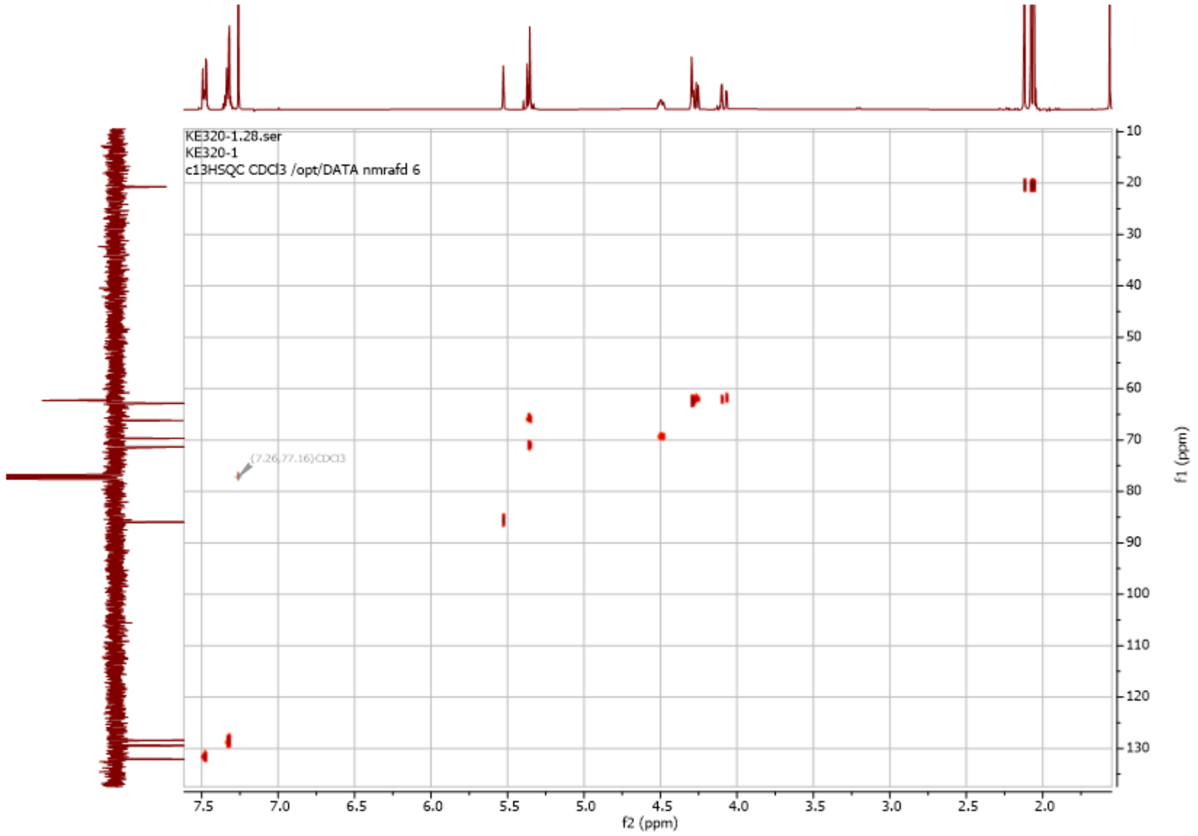
KE320-1.28.ser
KE320-1
c13HSQC CDCl3 /opt/DATA nmrafd 6
f2 (ppm)
f1 (ppm)

Phenyl 2-azido-2-deoxy-1-thio-α-D-mannopyranoside (S20)

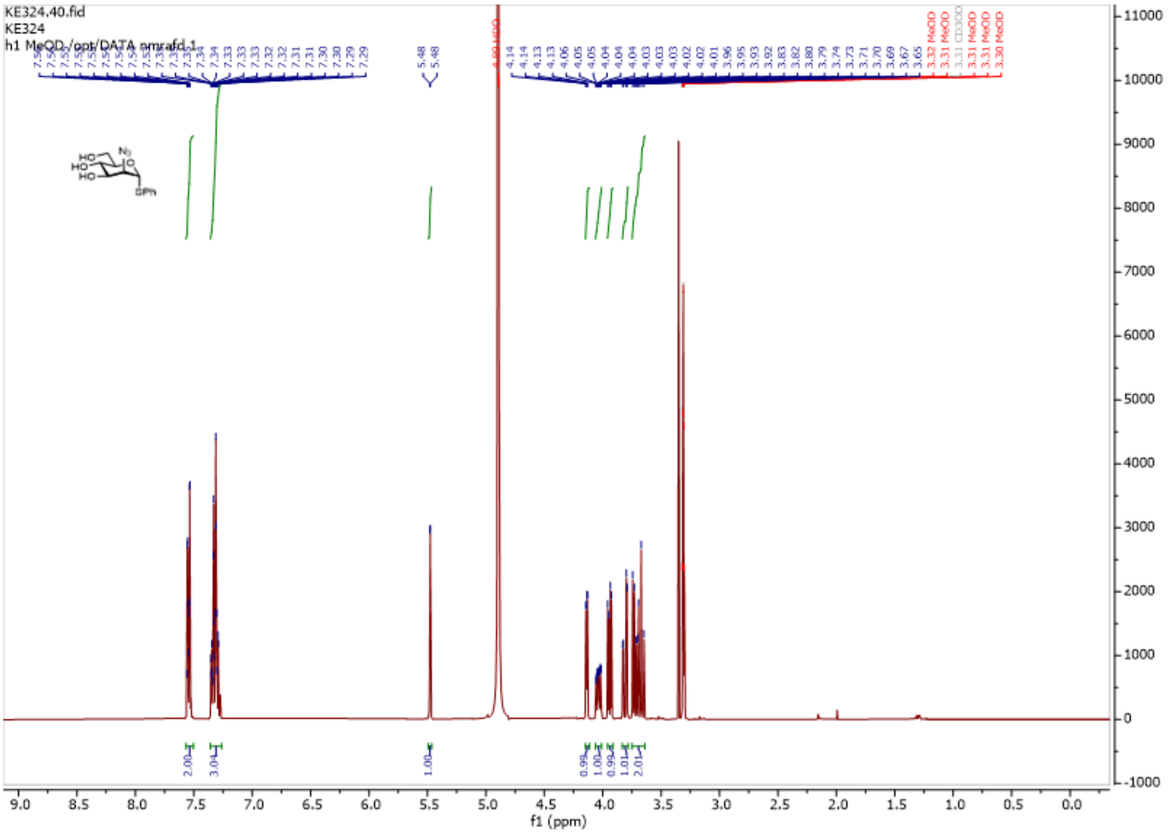

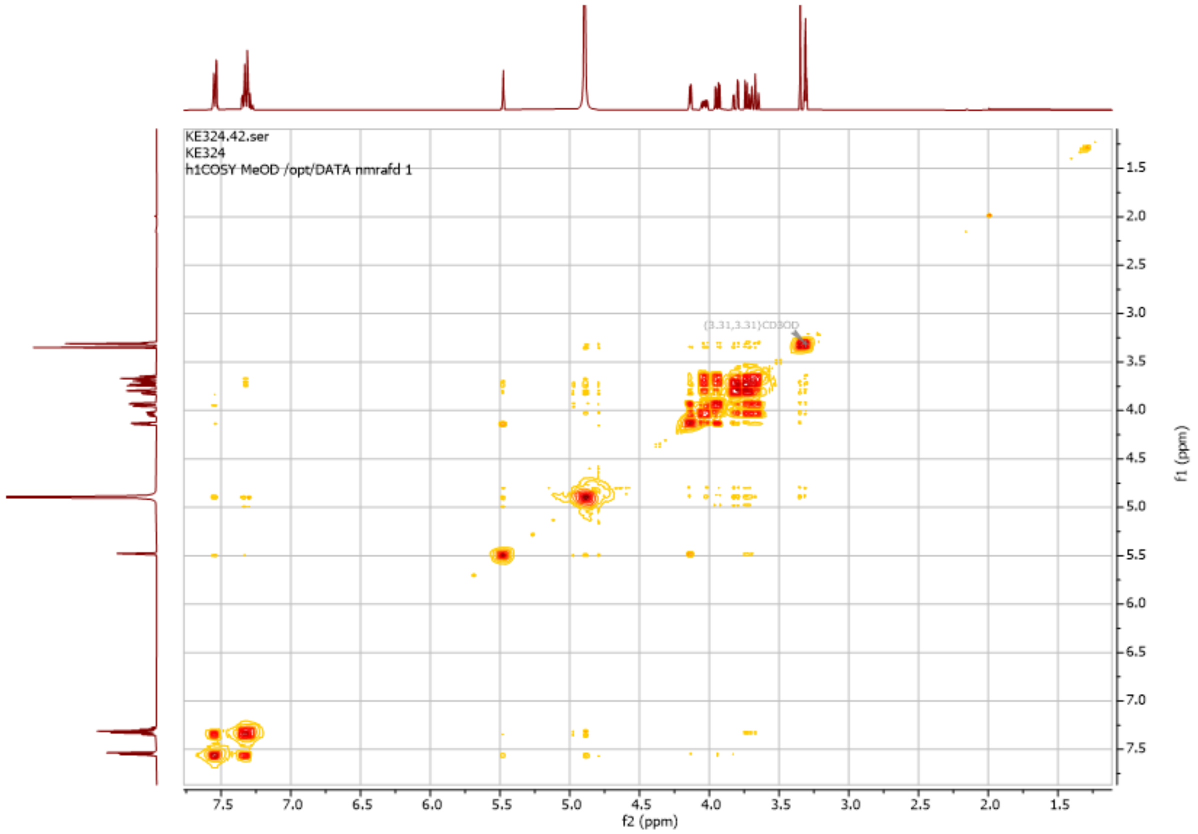
KE324.42.ser
KE324
h1COSY MeOD /opt/DATA nmrafd 1
f2 (ppm)
f1 (ppm)

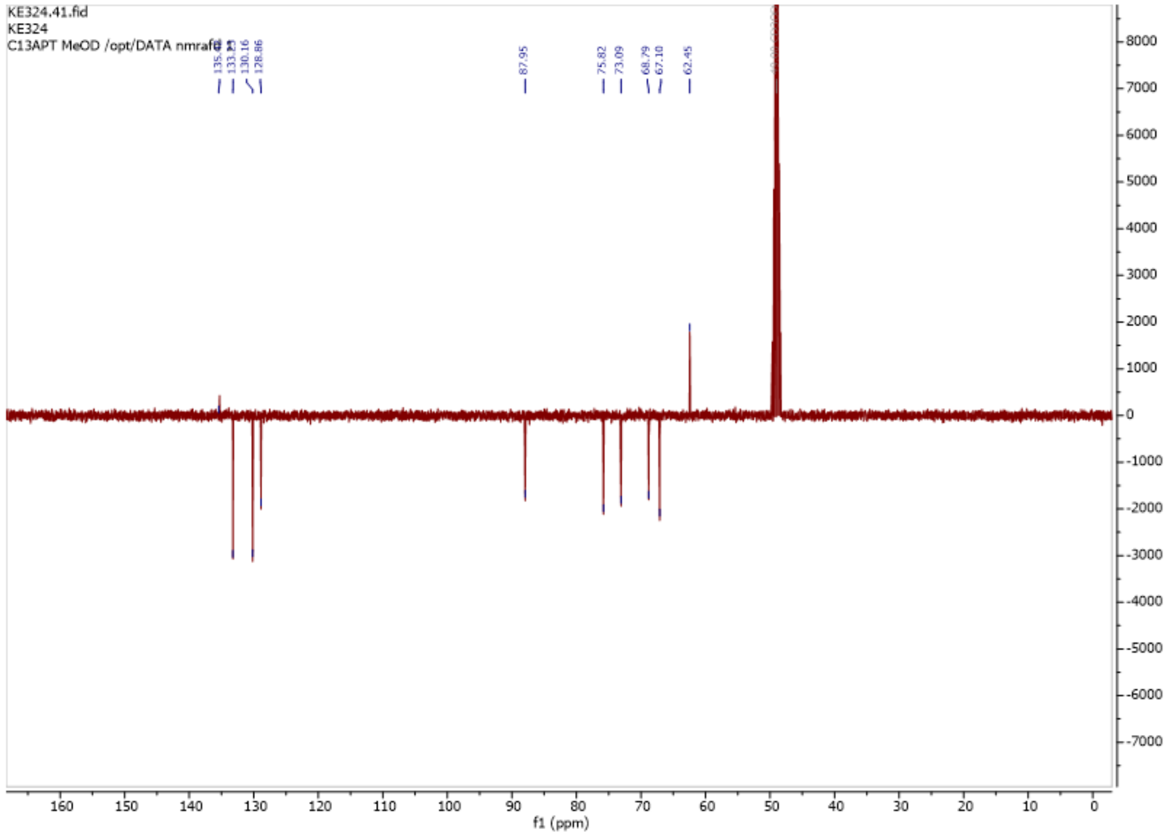
KE324.41.fid
KE324
C13APT MeOD /opt/DATA nmrafd 1
130.16
128.86
87.95
75.82
73.09
68.79
67.10
62.45
f1 (ppm)

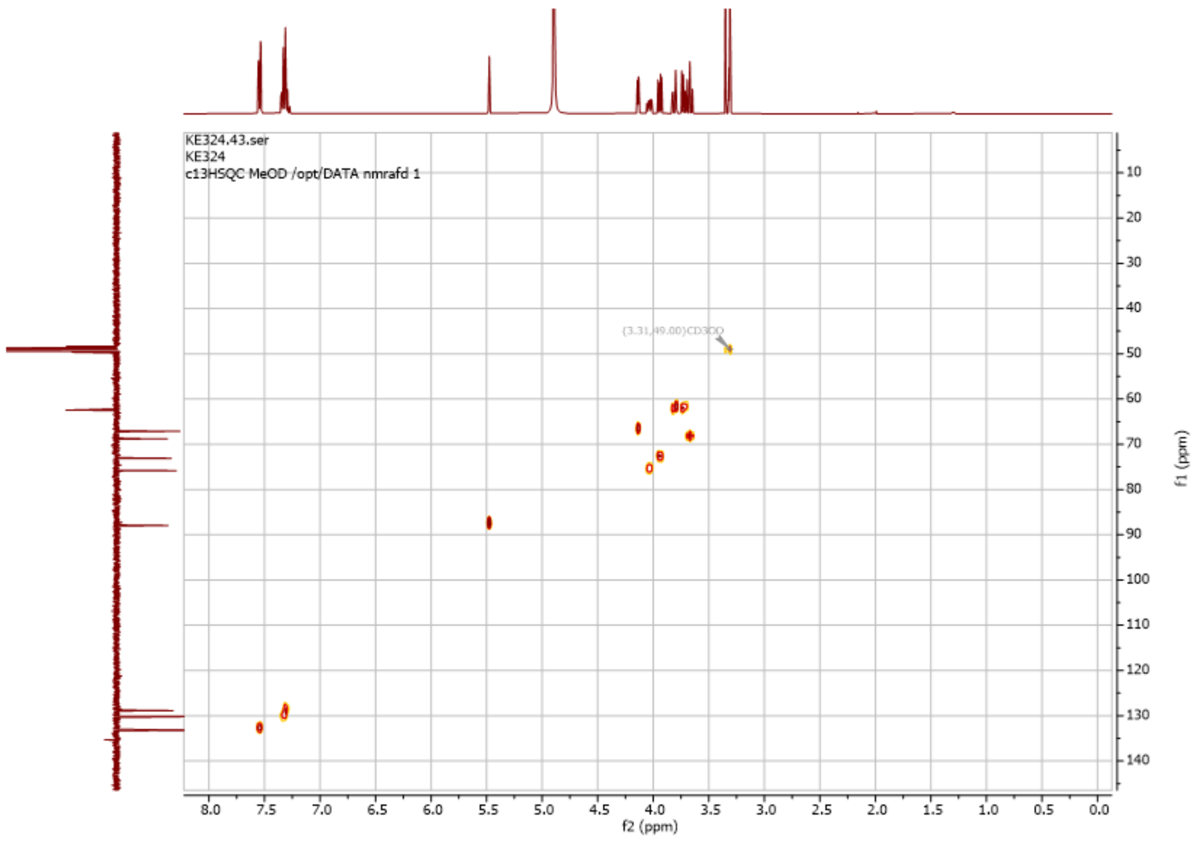

Phenyl 2-azido-2-deoxy-4,6-*O*-(*p*-methoxybenzylidene)-1-thio-α-D-mannopyranoside (S21)

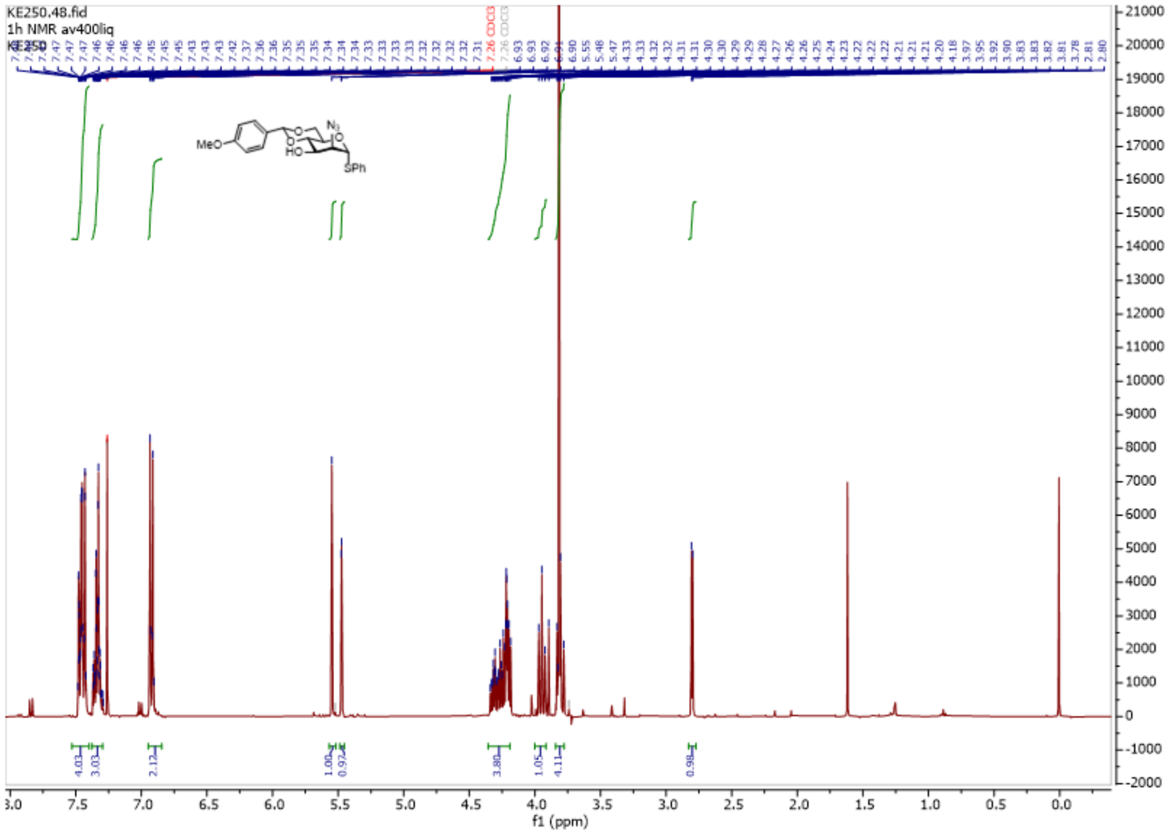

KE250.48.fid
1h NMR av400liq
KE250
MeO
HO
N3
SPh
f1 (ppm)

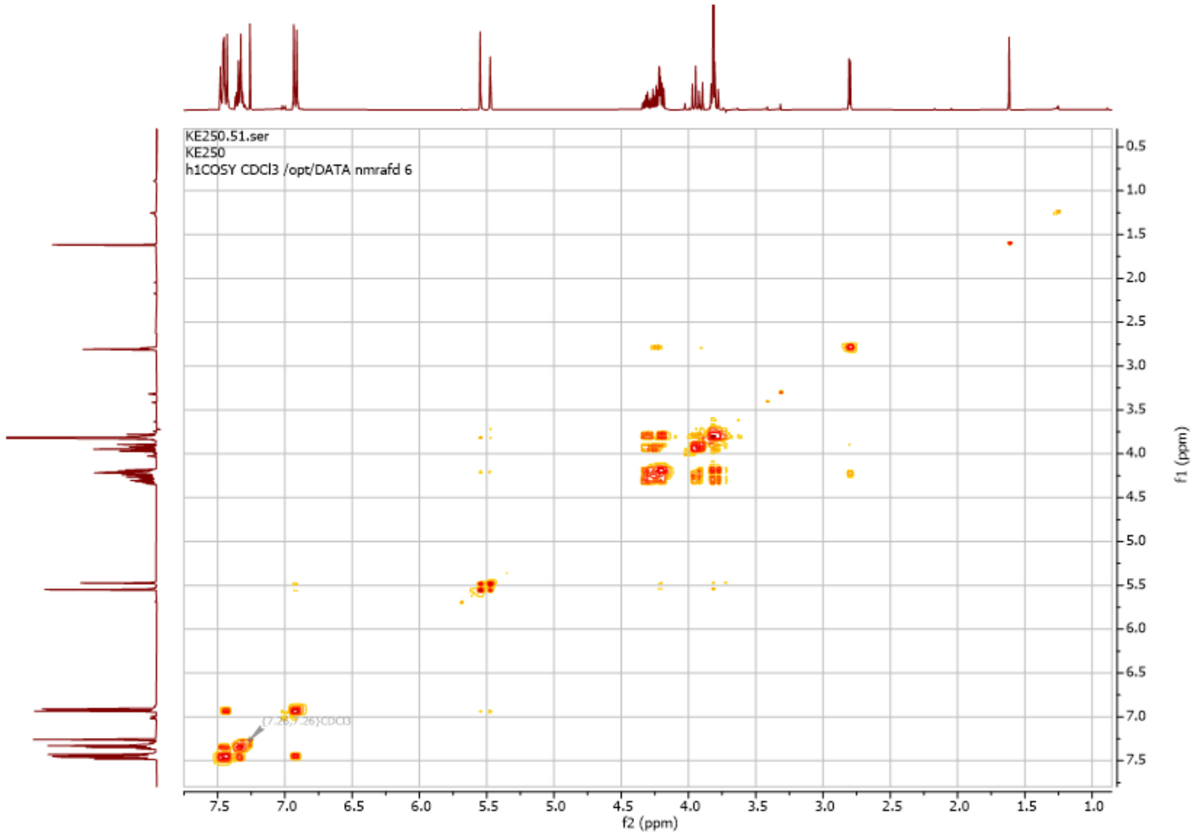

KE250.51.ser
KE250
h1COSY CDCl3 /opt/DATA nmrafd 6
f2 (ppm)
f1 (ppm)

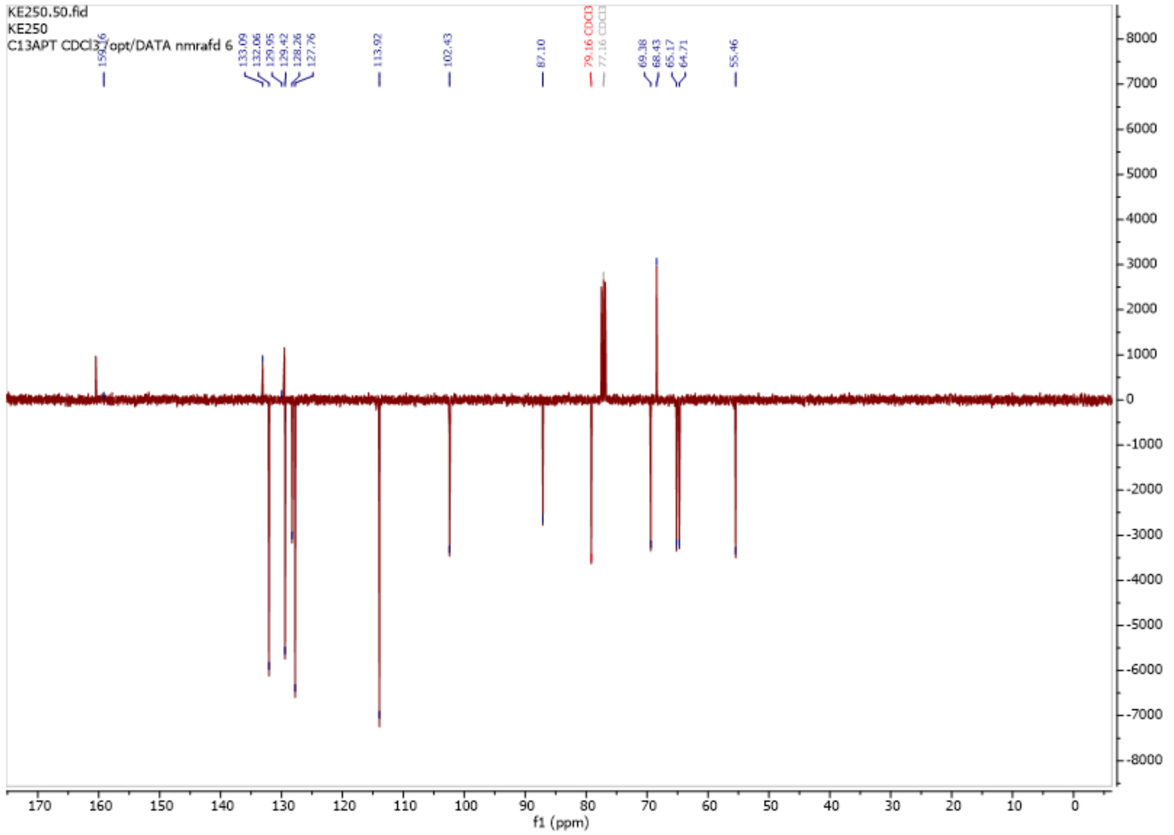
KE250.50.fid
KE250
C13APT CDCl3 /opt/DATA nmrafd 6
159.76
133.09
132.06
129.95
129.42
128.26
127.76
113.92
102.43
87.10
79.16 CDCl3
77.16 CDCl3
69.38
68.43
65.17
64.71
55.46
f1 (ppm)

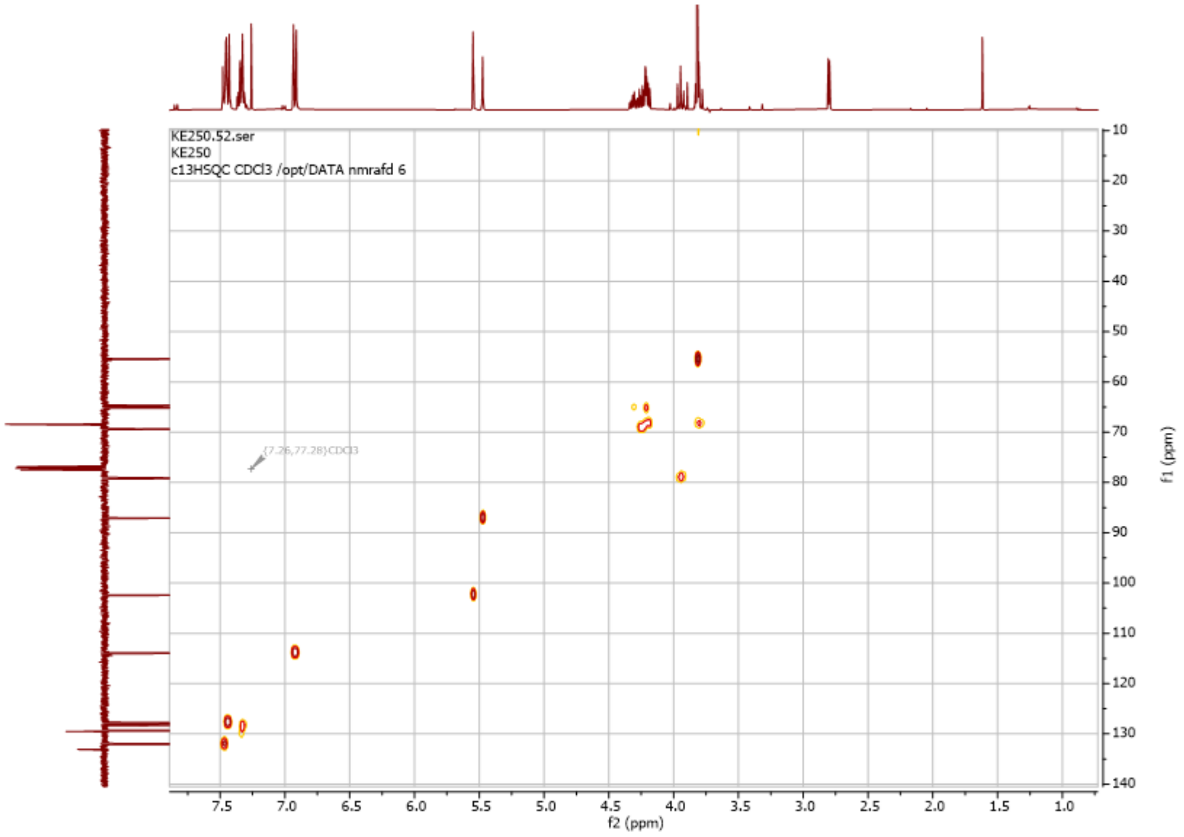
KE250.52.ser
KE250
c13HSQC CDCl3 /opt/DATA nmrafd 6
{7.26,77.28}CDCl3
f2 (ppm)
f1 (ppm)

Phenyl 2-azido-2-deoxy-4,6-*O*-*p*-methoxybenzylidene-3-*O*-benzyl-1-thio-α-D-mannopyranoside (S22)

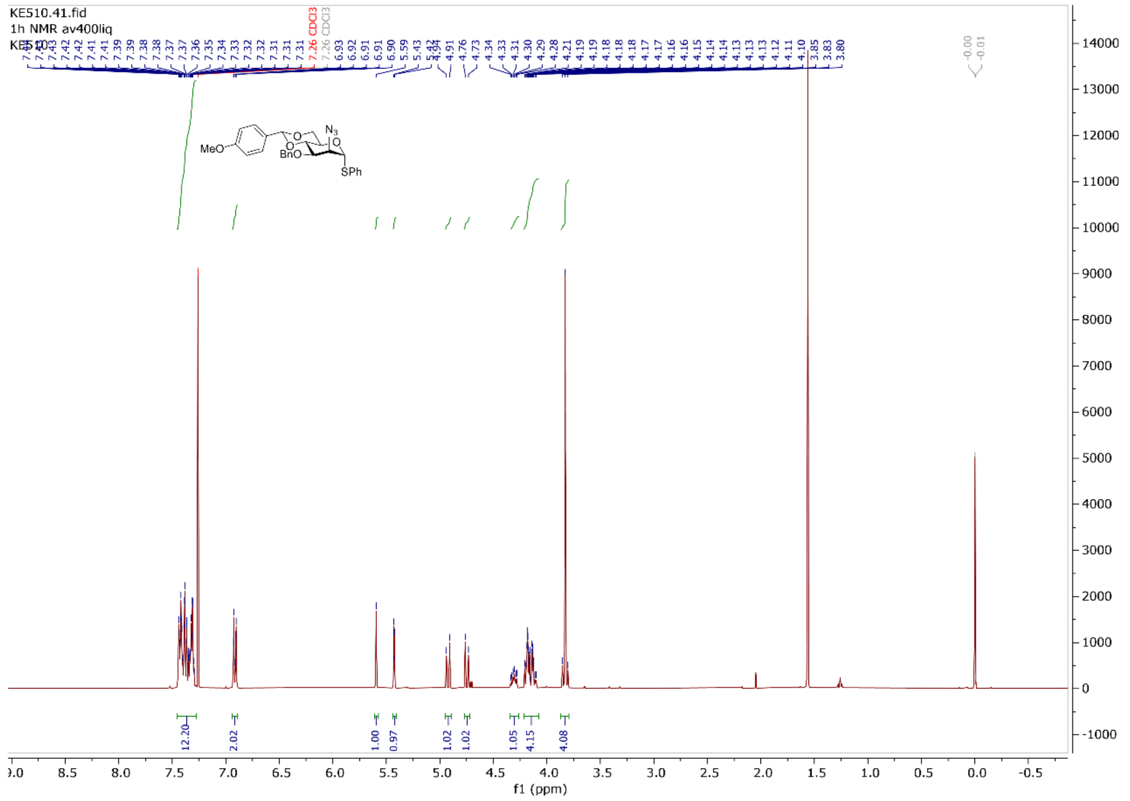

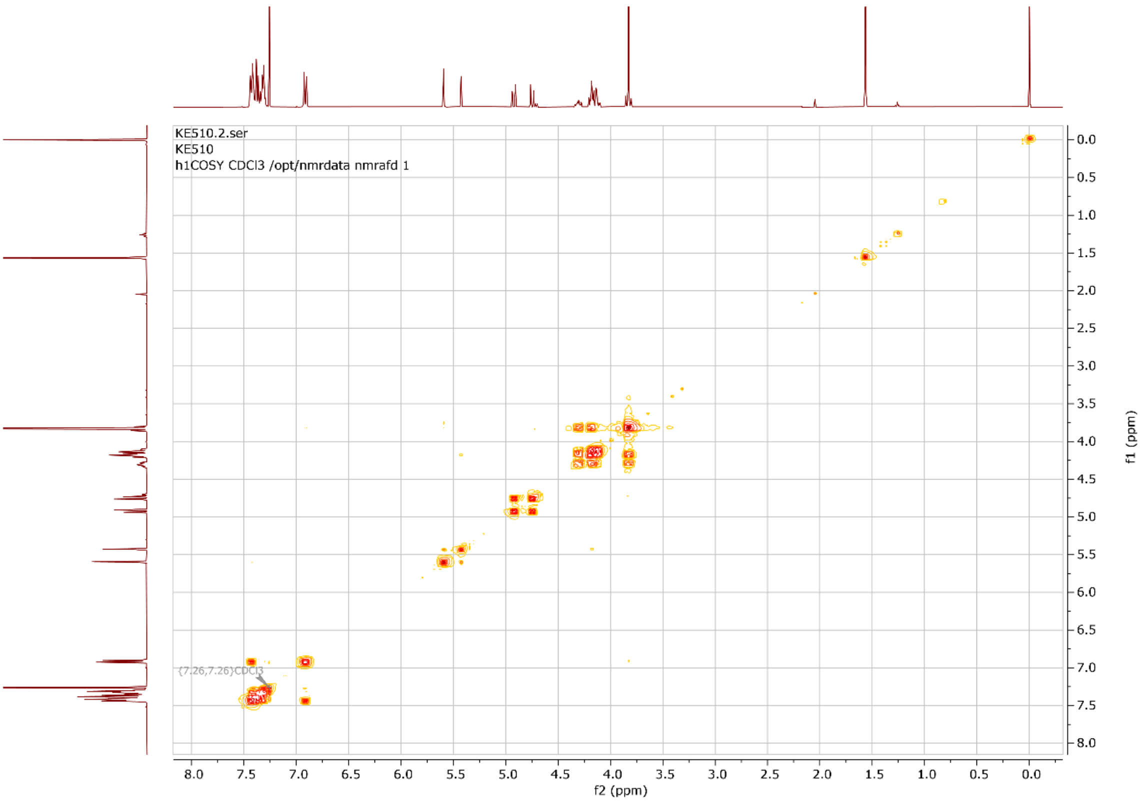
KE510.2.ser
KE510
h1COSY CDCl3 /opt/nmrdata nmrafd 1
{7.26,7.26}CDCl3
f2 (ppm)
f1 (ppm)

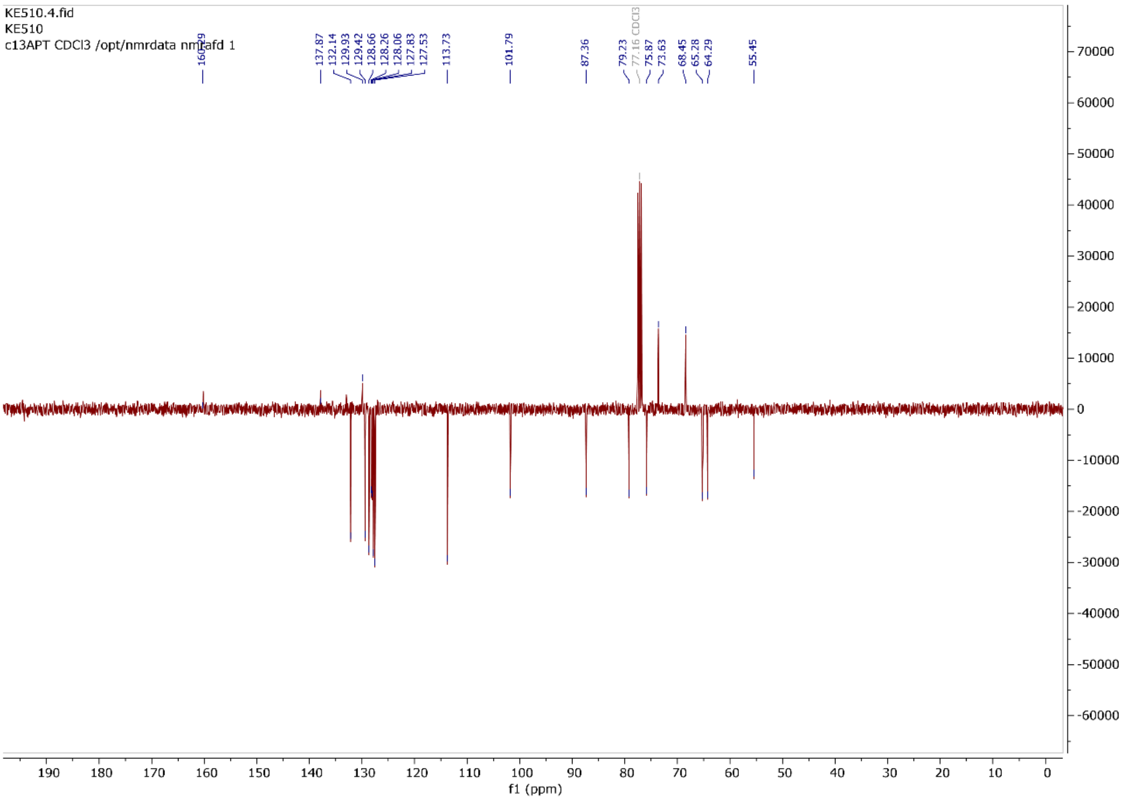
KE510.4.fid
KE510
c13APT CDCl3 /opt/nmrdata nmrafd 1
160.29
137.87
132.14
129.93
129.42
128.66
128.26
128.06
127.83
127.53
113.73
101.79
87.36
79.23
77.16 CDCl3
75.87
73.63
68.45
65.28
64.29
55.45
f1 (ppm)

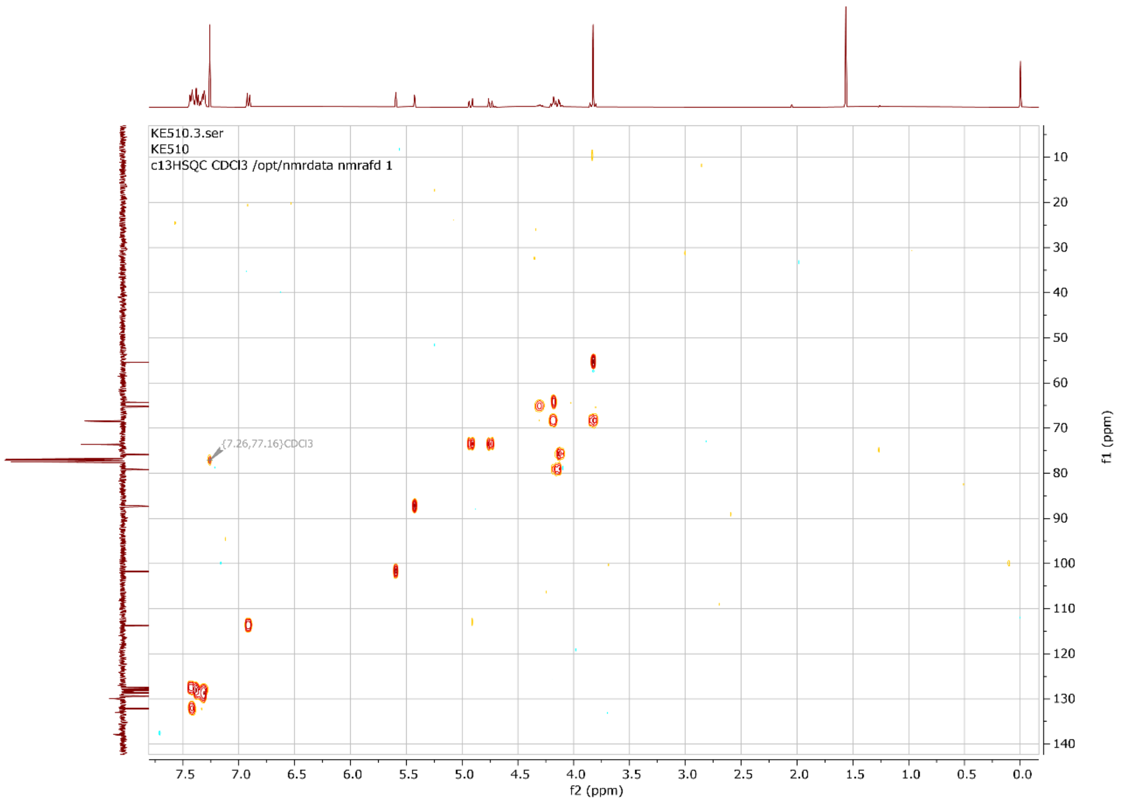

Phenyl 2-azido-2-deoxy-3-*O*-benzyl-4-*O*-*p*-methoxybenzyl-1-thio-α-D-mannopyranoside (S23)

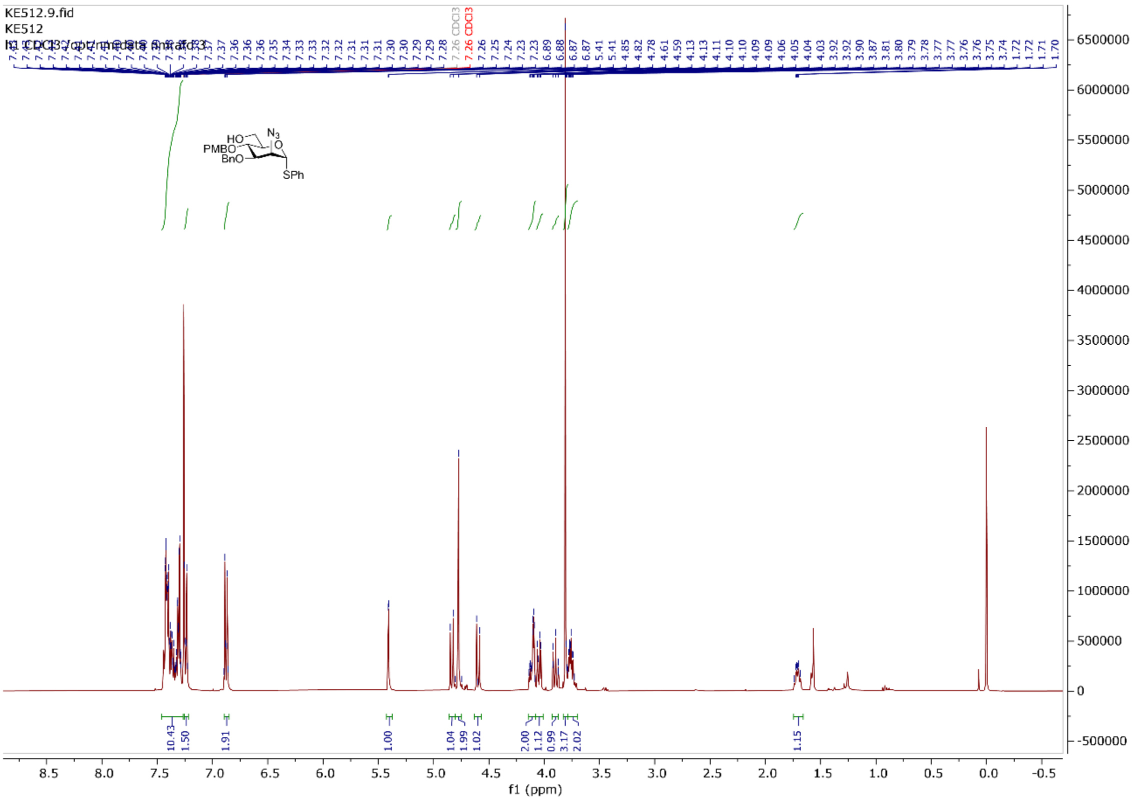
KE512.9.fid
KE512
h1 CDCl3 /opt/nmrdata nmrafd 3
HO
N3
PMBO
BnO
O
SPh
f1 (ppm)

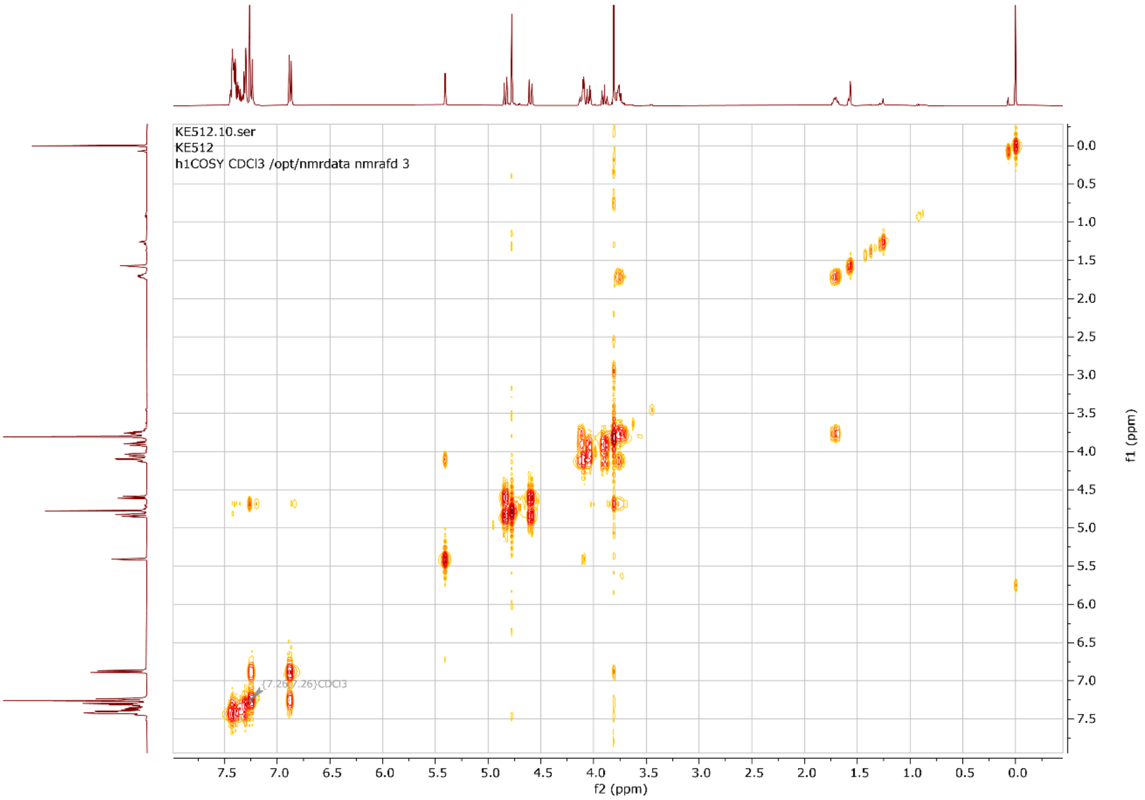
KE512.10.ser
KE512
h1COSY CDCl3 /opt/nmrdata nmrafd 3
f2 (ppm)
f1 (ppm)

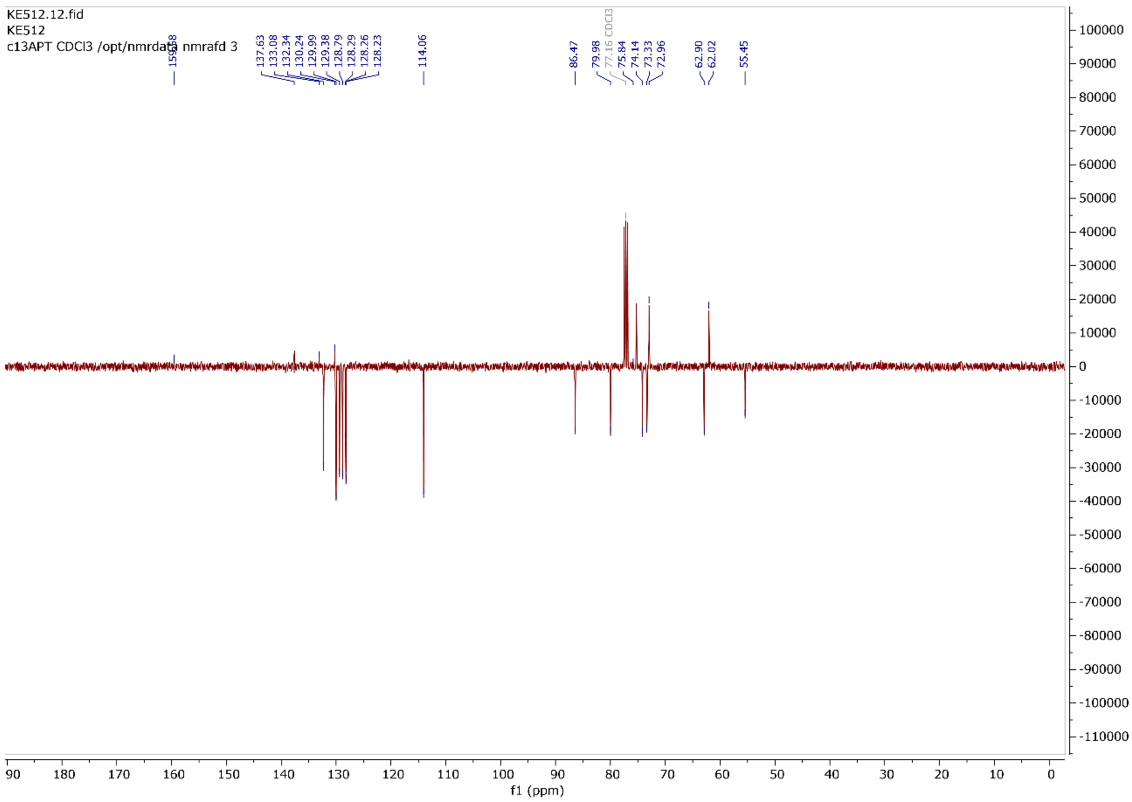
KE512.12.fid
KE512
c13APT CDCl3 /opt/nmrdata nmrafd 3
137.63
133.08
132.34
130.24
129.99
129.38
128.79
128.29
128.26
128.23
114.06
86.47
79.98
77.16 CDCl3
75.84
74.14
73.33
72.96
62.90
62.02
55.45
f1 (ppm)

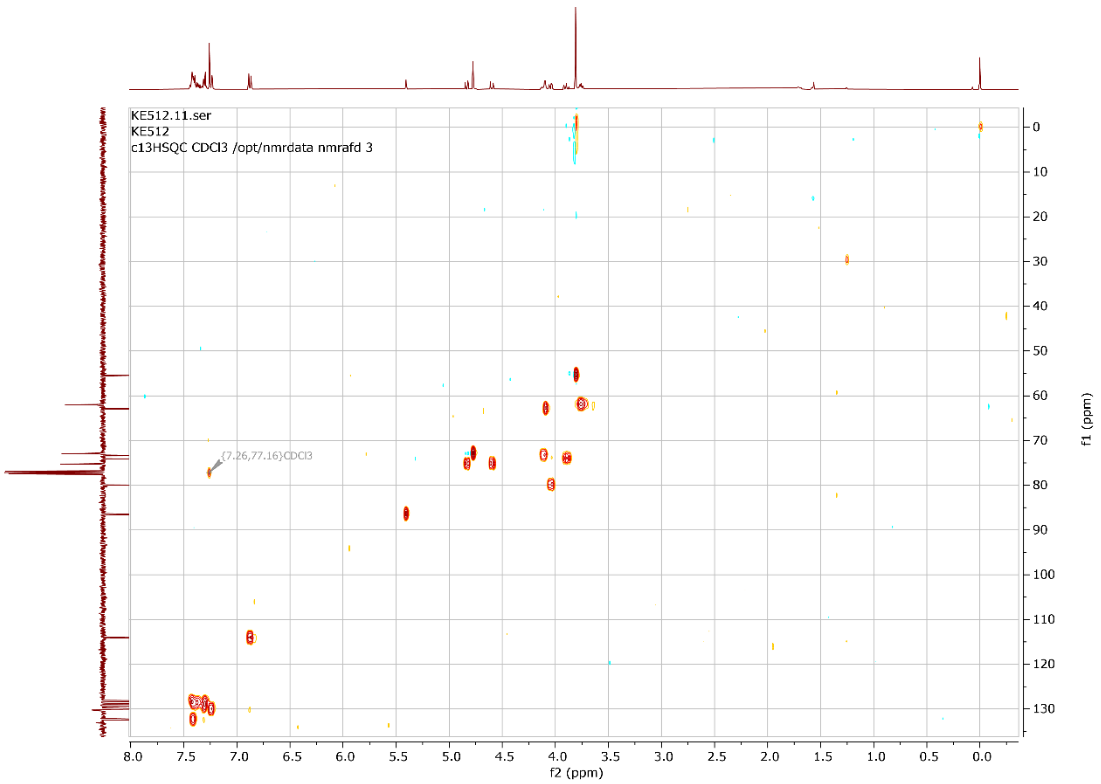
KE512.11.ser
KE512
c13HSQC CDCl3 /opt/nmrdata nmrafd 3
{7.26,77.16}CDCl3
f2 (ppm)
f1 (ppm)

Benzyl (phenyl 2-azido-3-*O*-benzyl-2-deoxy-4-*O*-*p*-methoxybenzyl-1-thio-α-D-mannopyranosiduronate) (10)

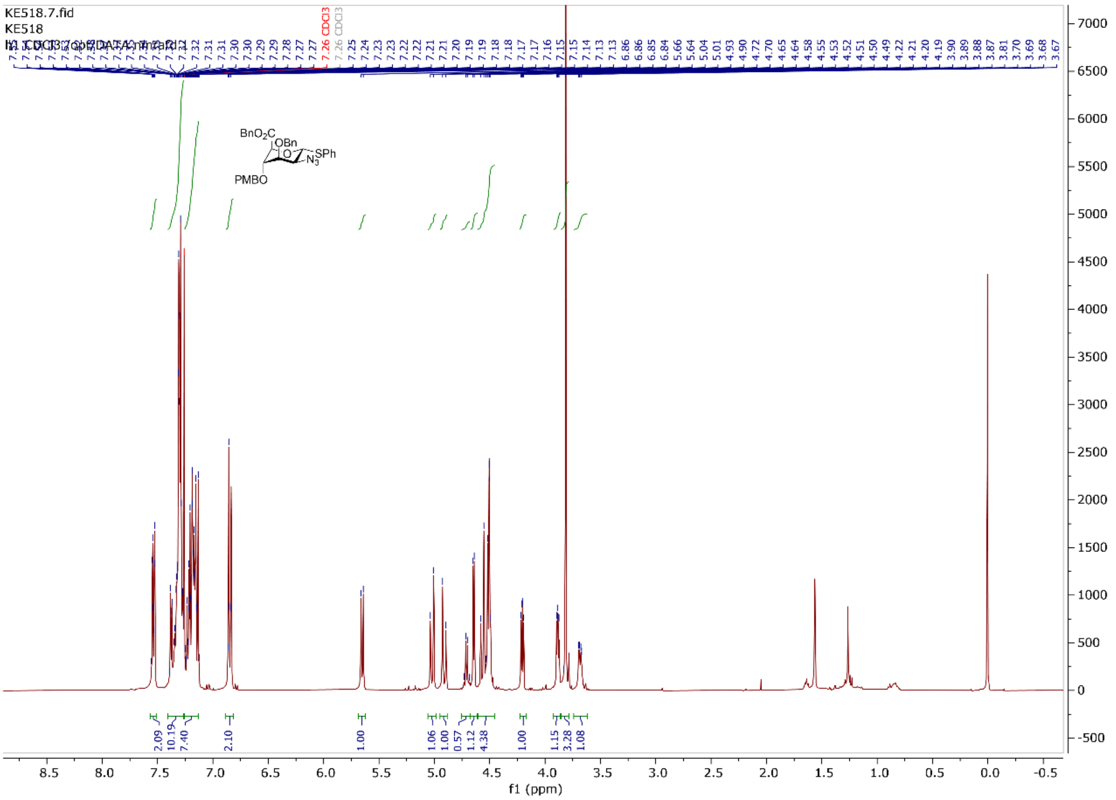

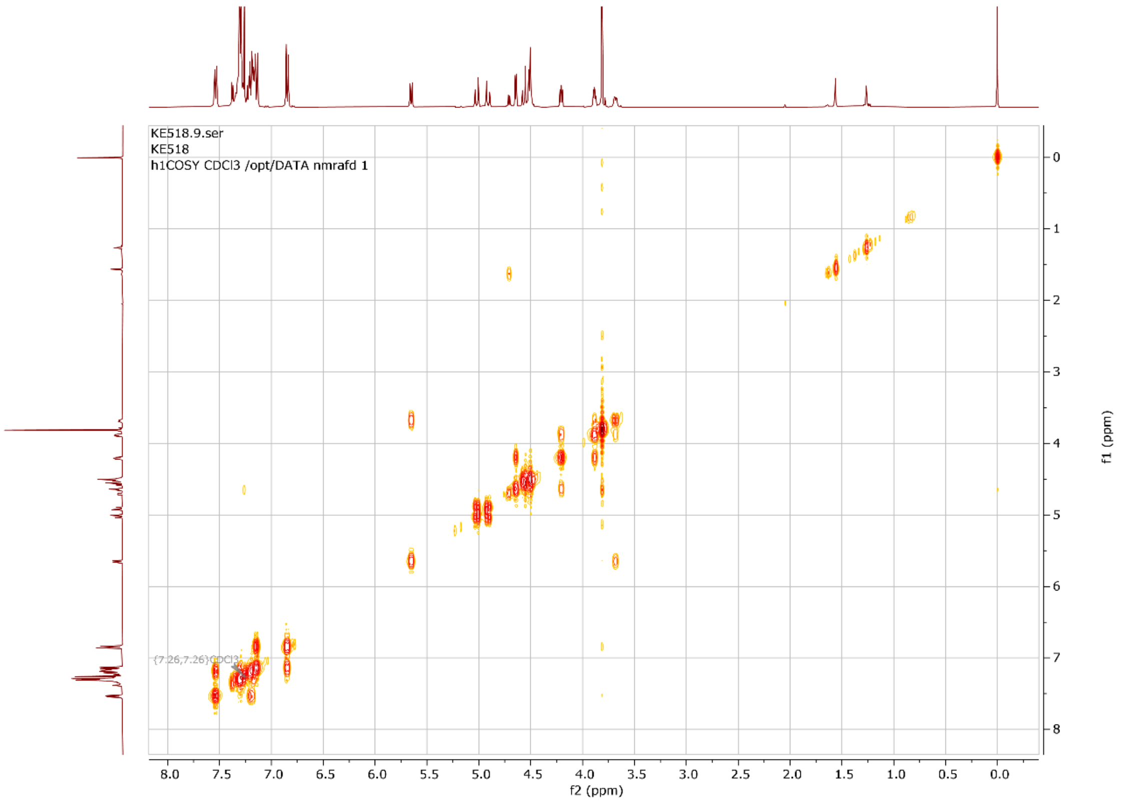

KE518.9.ser
KE518
h1COSY CDCl3 /opt/DATA nmrafd 1
f2 (ppm)
f1 (ppm)

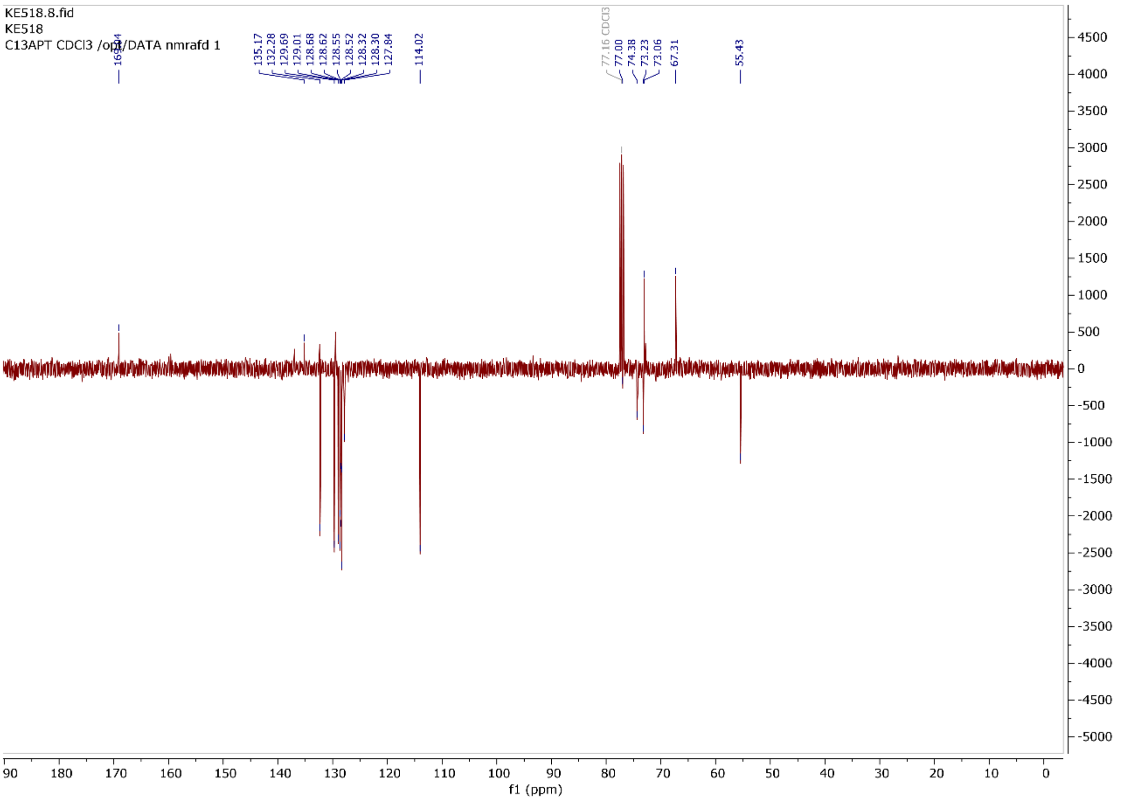

KE518.8.fid
KE518
C13APT CDCl3 /opt/DATA nmrafd 1
135.17
132.28
129.69
129.01
128.68
128.62
128.55
128.52
128.32
128.30
127.84
114.02
77.16 CDCl3
77.00
74.38
73.23
73.06
67.31
55.43
f1 (ppm)

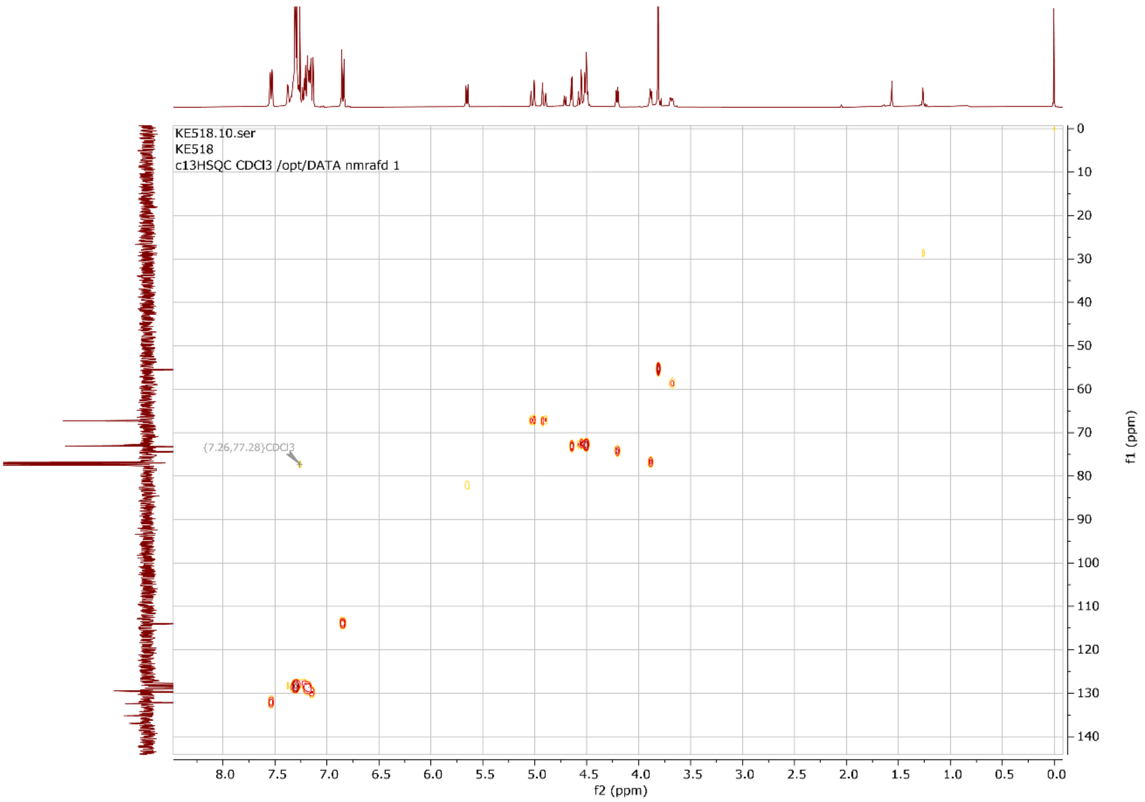

Phenyl 2-azido-4,6-*O*-benzylidene-2-deoxy-1-thio-α-D-mannopyranoside (S24)

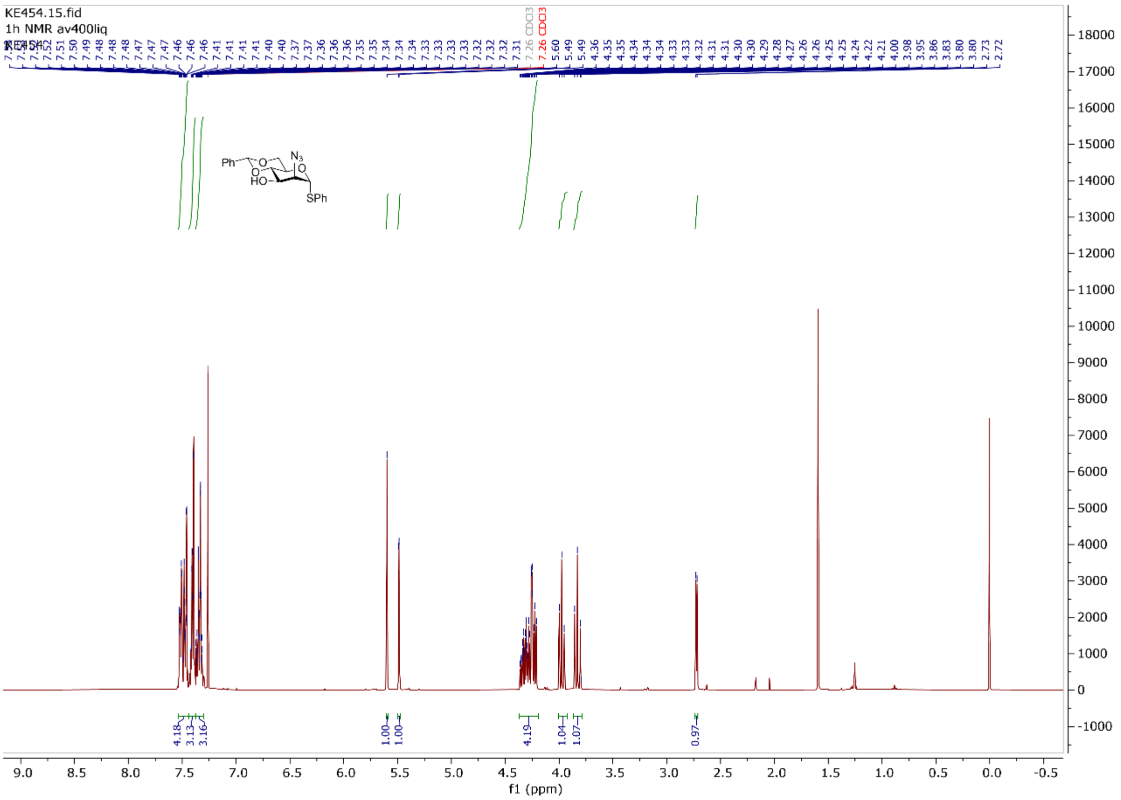
KE454.15.fid
1h NMR av400liq
KE454
f1 (ppm)

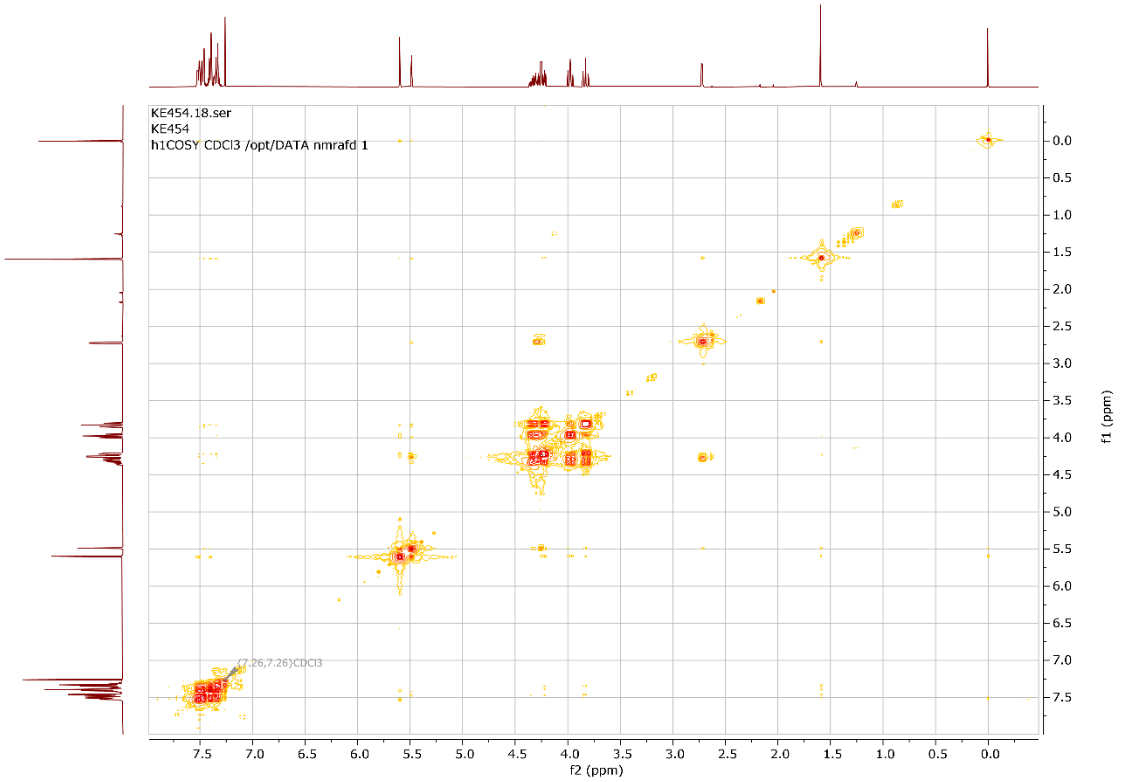
KE454.18.ser
KE454
h1COSY CDCl3 /opt/DATA nmrafd 1
f2 (ppm)
f1 (ppm)

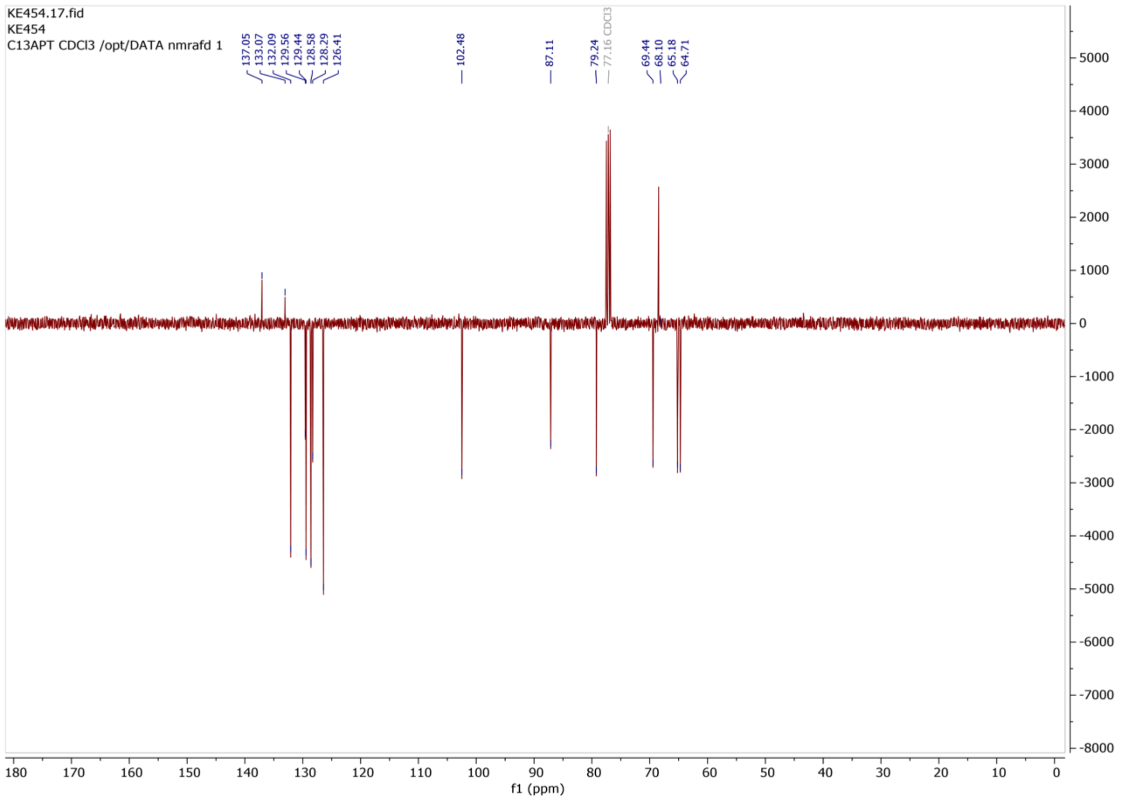
KE454.17.fid
KE454
C13APT CDCl3 /opt/DATA nmrafd 1
137.05
133.07
132.09
129.56
129.44
128.58
128.29
126.41
102.48
87.11
79.24
77.16 CDCl3
69.44
68.10
65.18
64.71
f1 (ppm)

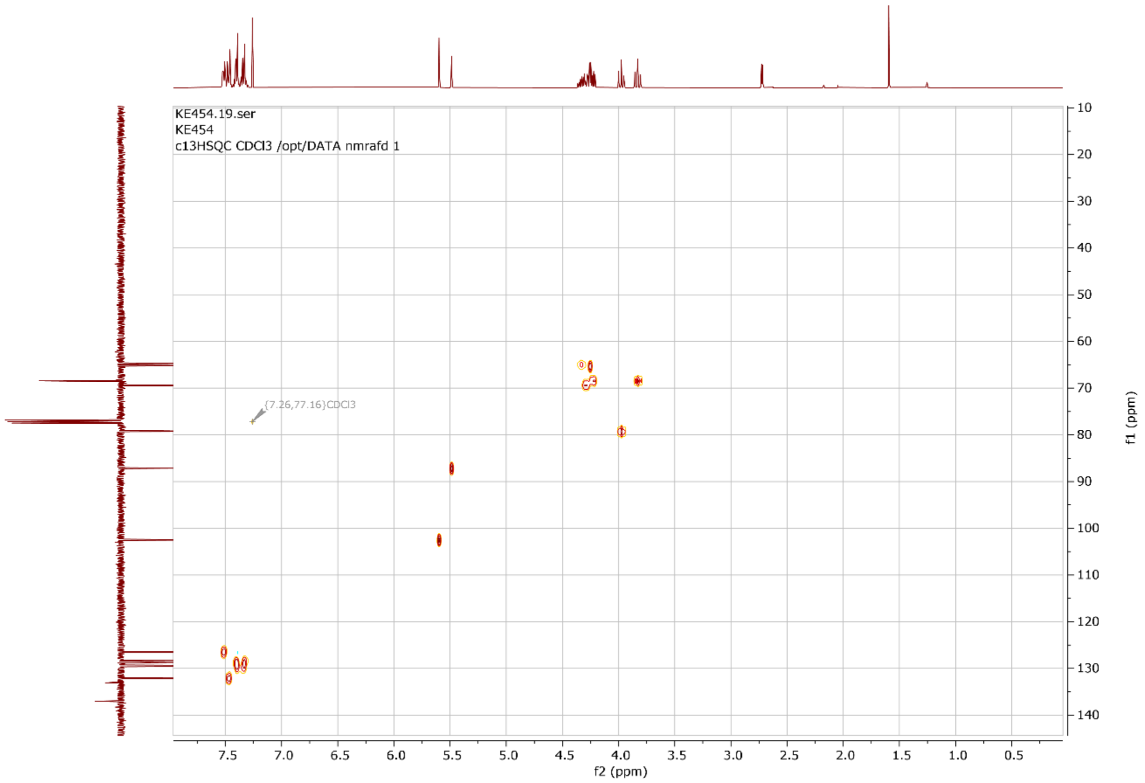
KE454.19.ser
KE454
c13HSQC CDCl3 /opt/DATA nmrafd 1
{7.26,77.16}CDCl3
f2 (ppm)
f1 (ppm)

Phenyl 2-azido-4,6-*O*-benzylidene-3-*O*-benzyl-2-deoxy-1-thio-α-D-mannopyranoside (S25)

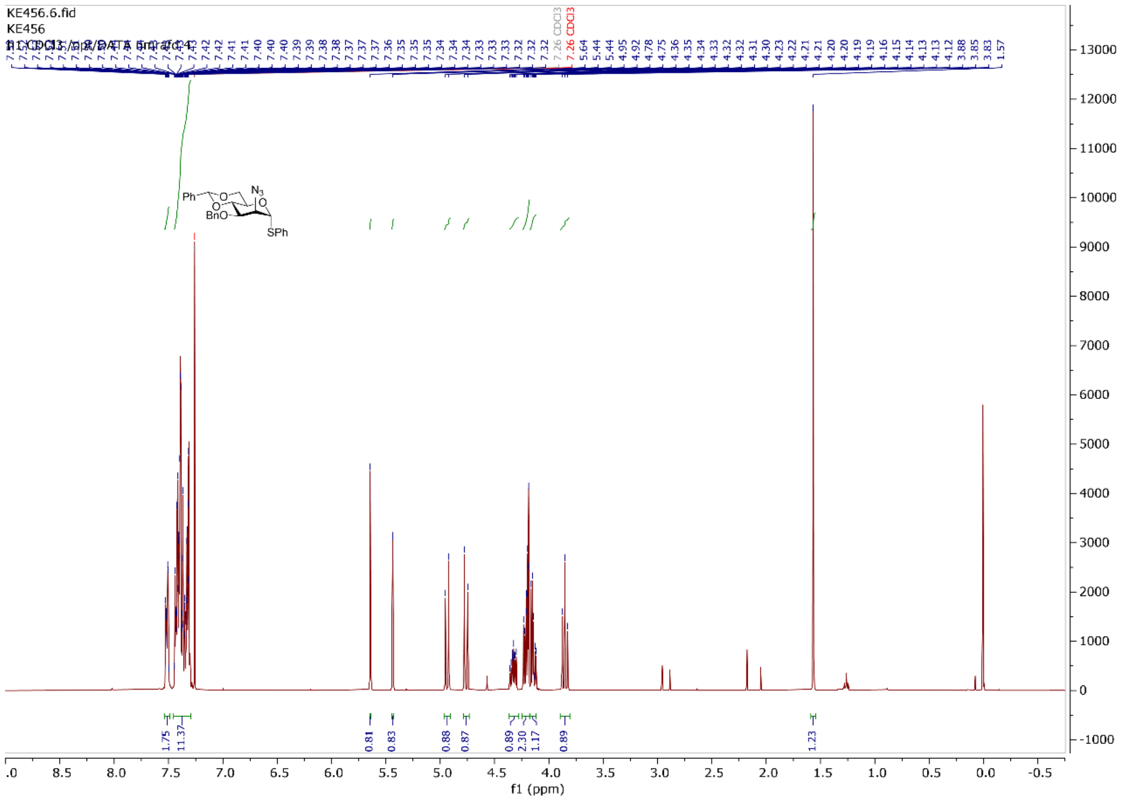

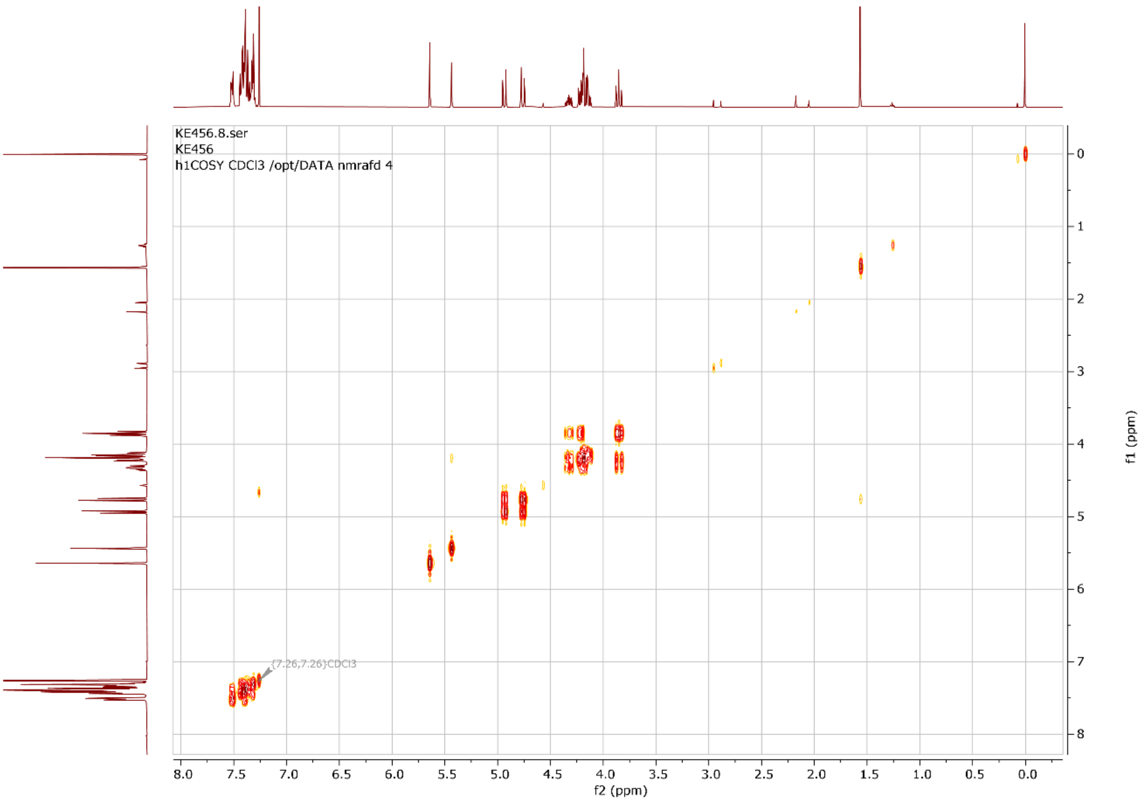
KE456.8.ser
KE456
h1COSY CDCl3 /opt/DATA nmrafd 4
{7.26,7.26}CDCl3
f2 (ppm)
f1 (ppm)

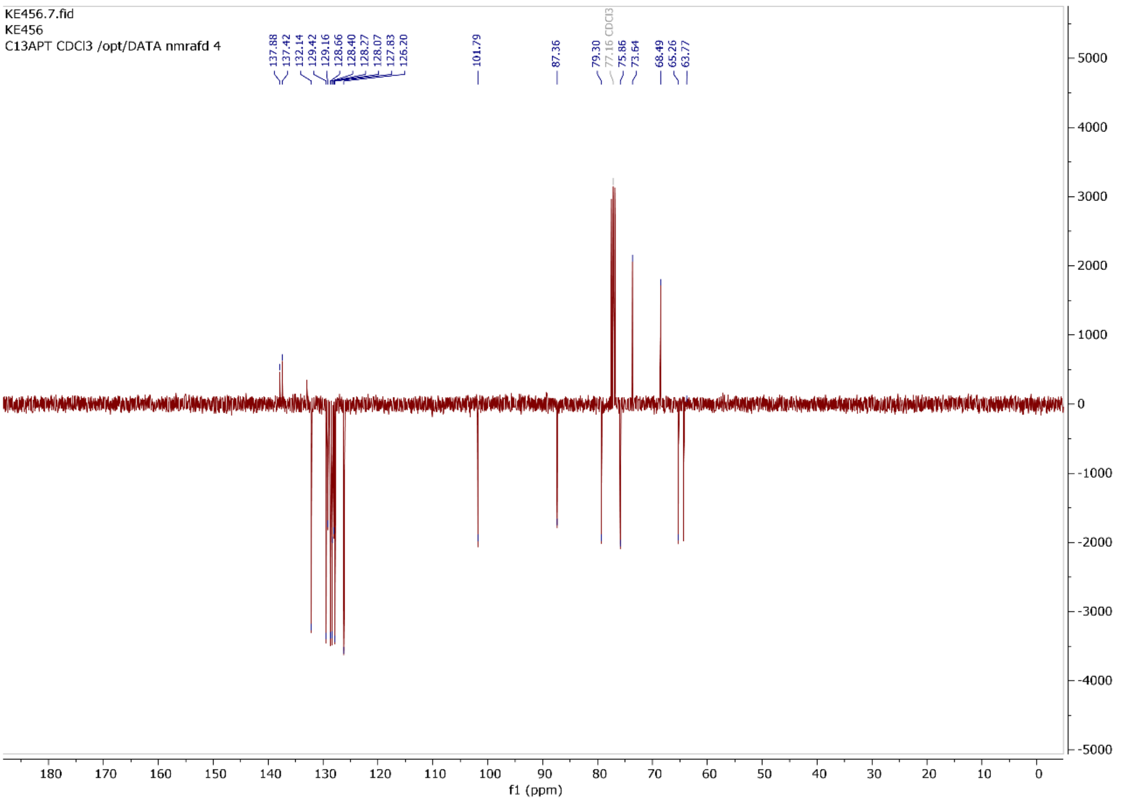
KE456.7.fid
KE456
C13APT CDCl3 /opt/DATA nmrafd 4
137.88
137.42
132.14
129.42
129.16
128.66
128.40
128.27
128.07
127.83
126.20
101.79
87.36
79.30
77.16 CDCl3
75.86
73.64
68.49
65.26
63.77
f1 (ppm)

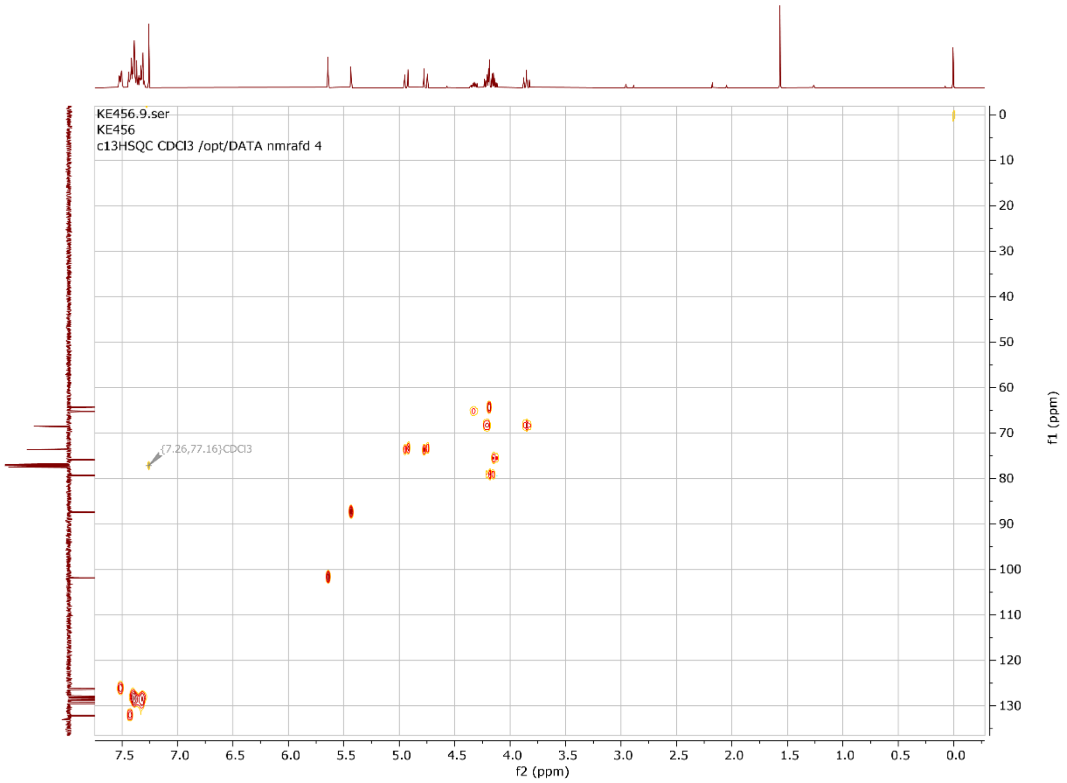

Phenyl 2-azido-2-deoxy-3,4-di-*O*-benzyl-1-thio-α-D-mannopyranoside (S26)

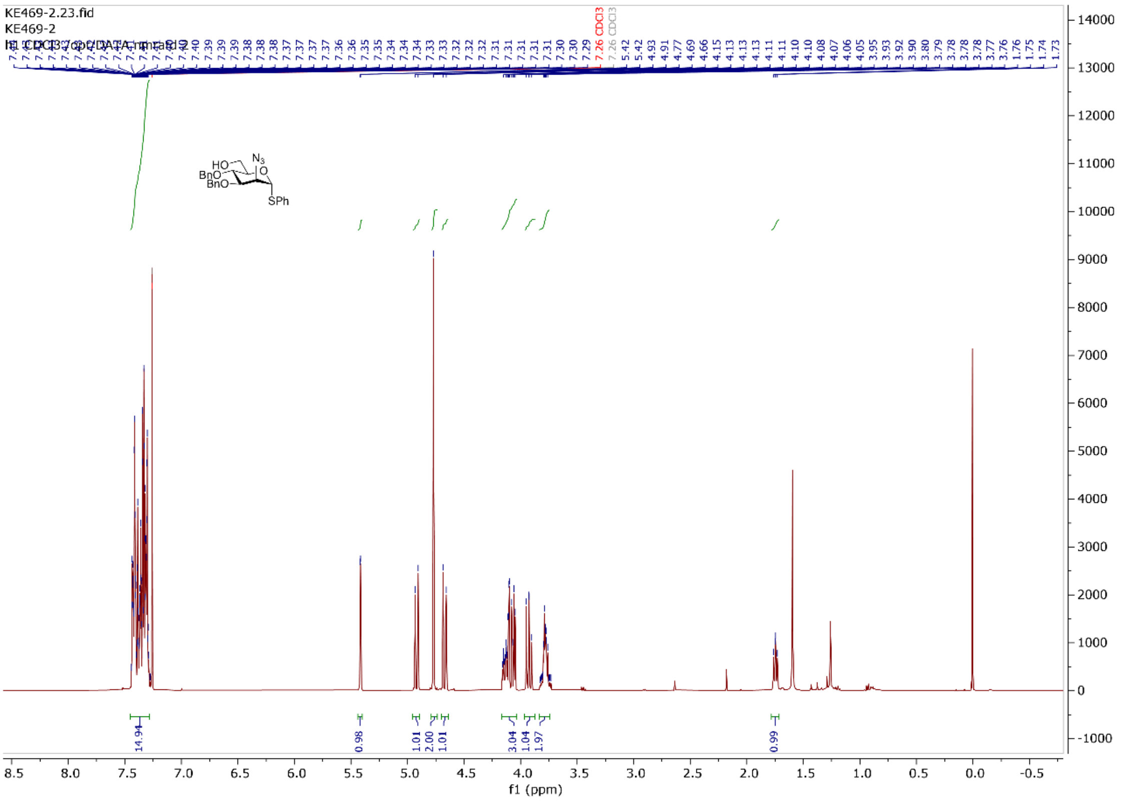
KE469-2.23.fid
KE469-2
HO
BnO
BnO
N_3
O
SPh
f1 (ppm)

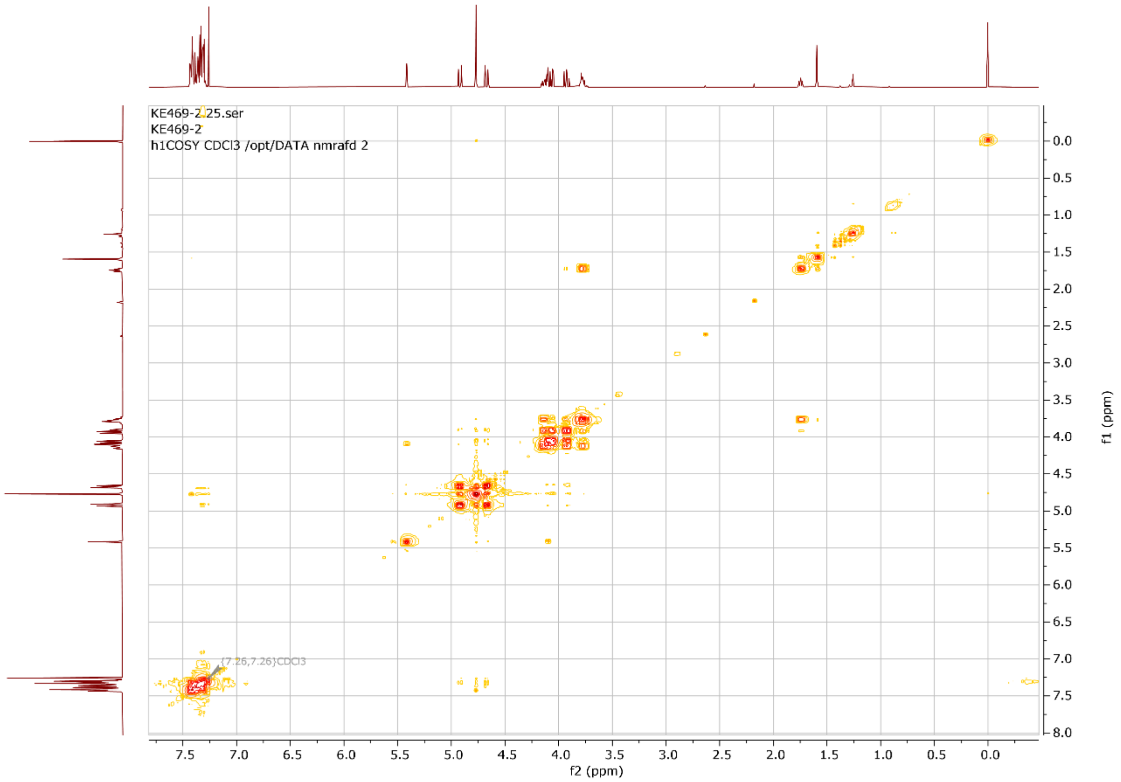
KE469-2.25.ser
KE469-2
h1COSY CDCl3 /opt/DATA nmrafd 2
f2 (ppm)
f1 (ppm)

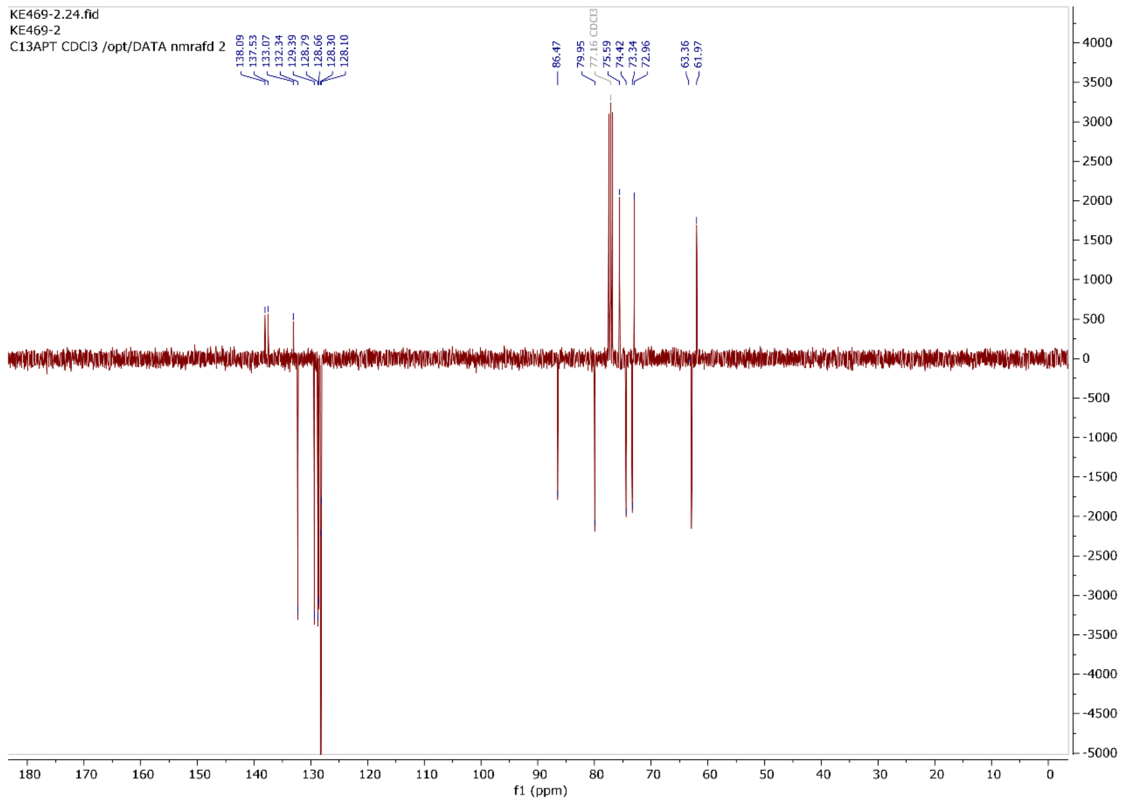
KE469-2.24.fid
KE469-2
C13APT CDCl3 /opt/DATA nmrafd 2
138.09
137.53
133.07
132.34
129.39
128.79
128.66
128.30
128.10
86.47
79.95
77.16 CDCl3
75.59
74.42
73.34
72.96
63.36
61.97
f1 (ppm)

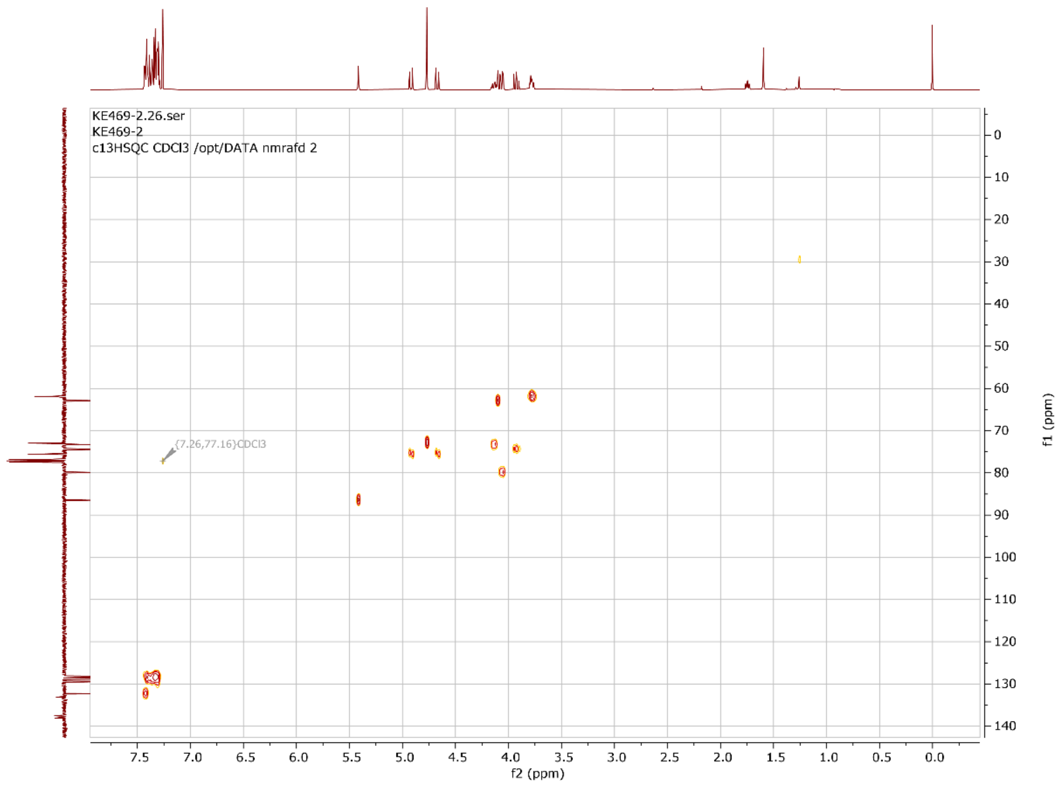
KE469-2.26.ser
KE469-2
c13HSQC CDCl3 /opt/DATA nmrafd 2
{7.26,77.16}CDCl3
f2 (ppm)
f1 (ppm)

Benzyl (phenyl 2-azido-3,4-di-*O*-benzyl-2-deoxy-1-thio-α-D-mannopyranosiduronate) (11)

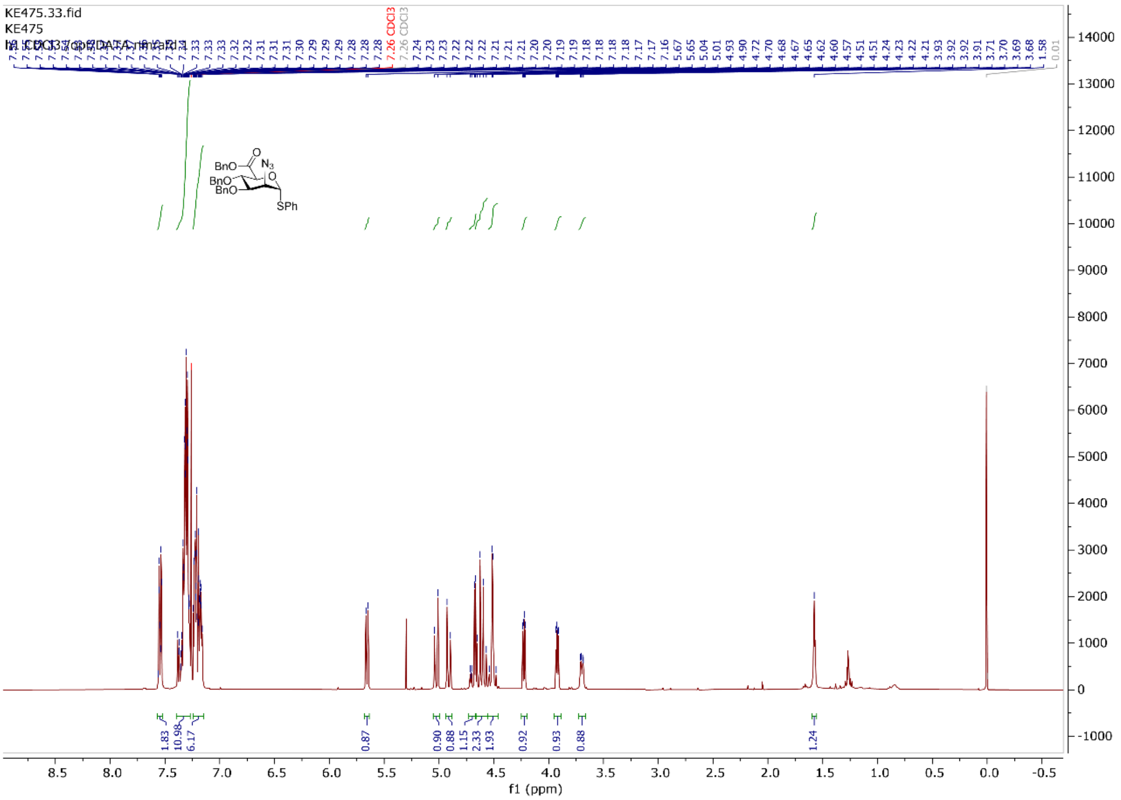

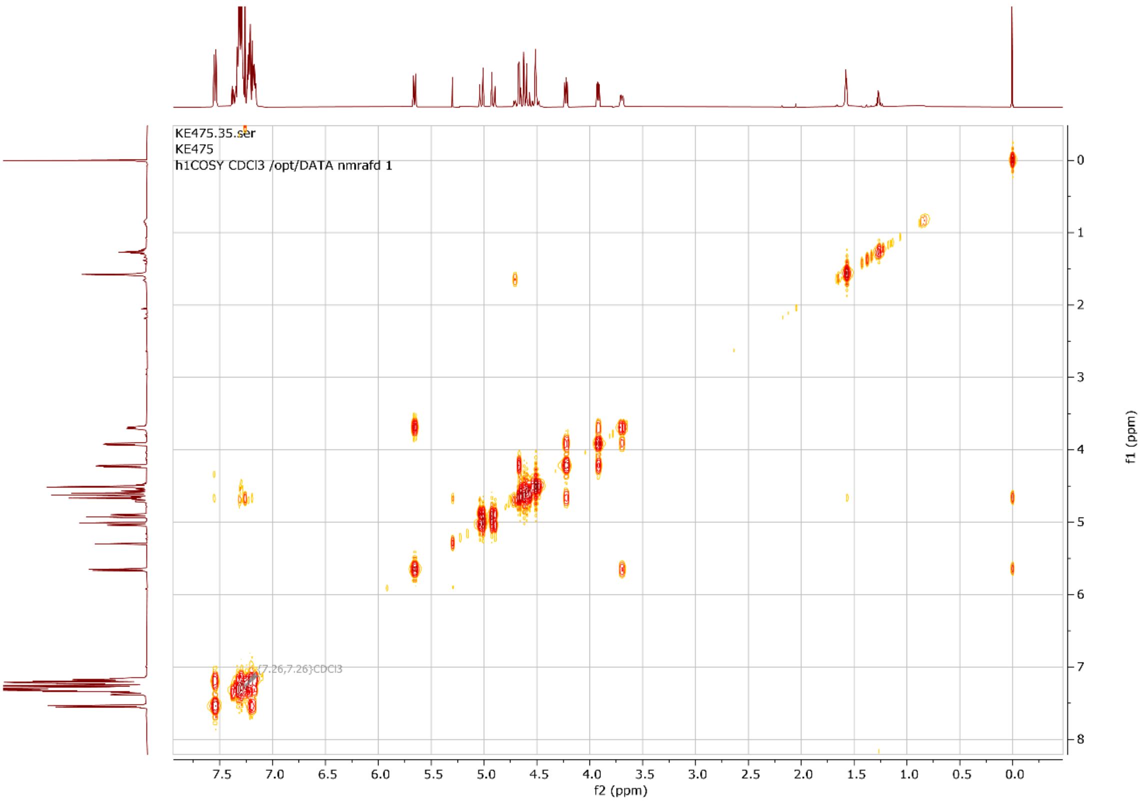
KE475.35.ser
KE475
h1COSY CDCl3 /opt/DATA nmrafd 1
{7.26,7.26}CDCl3
f2 (ppm)
f1 (ppm)

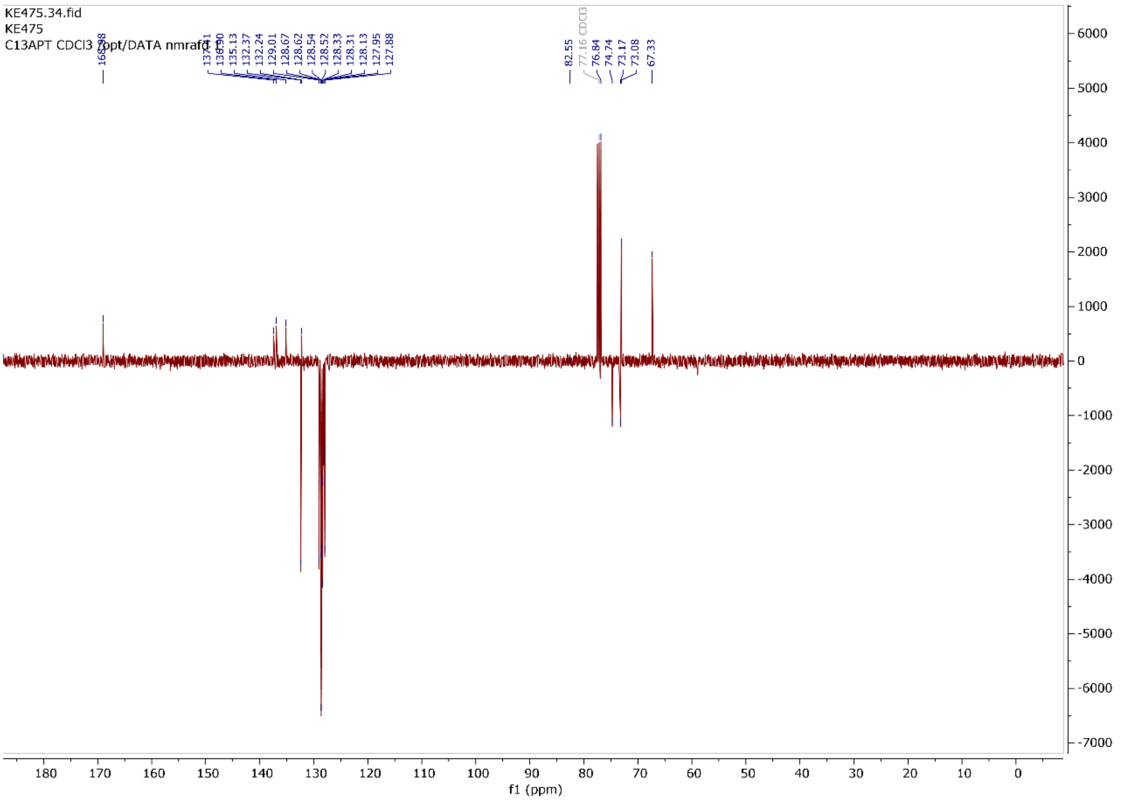
KE475.34.fid
KE475
C13APT CDCl3 /opt/DATA nmrafd 1
136.90
135.13
132.37
132.24
129.01
128.67
128.62
128.54
128.52
128.33
128.31
128.13
127.95
127.88
82.55
77.16 CDCl3
76.84
74.74
73.17
73.08
67.33
f1 (ppm)

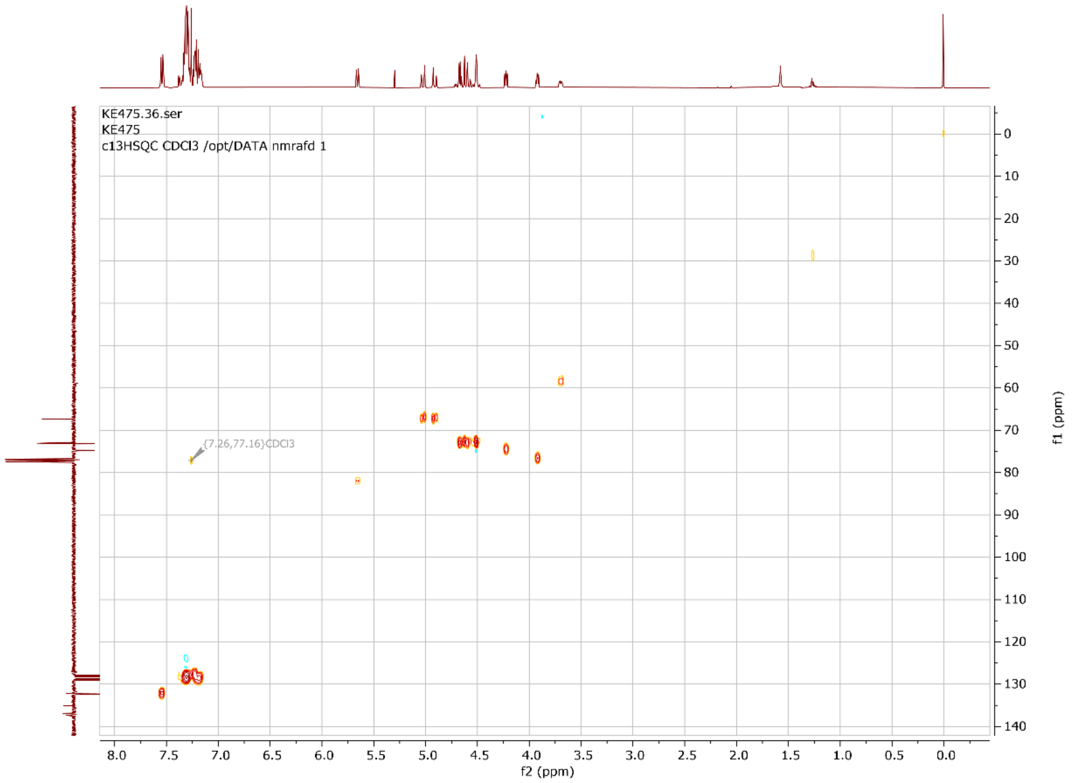

Tert-butyldiphenylsilyl 2-azido-4-_O_-benzoyl-2-deoxy-3-_O_-(2-naphthylmethyl)-α-L-fucopyranosyl-(1→3)-2-deoxy-2-_N_-trichloroacetamide-4-_O_-benzyl-β-D-fucopyranoside (15)

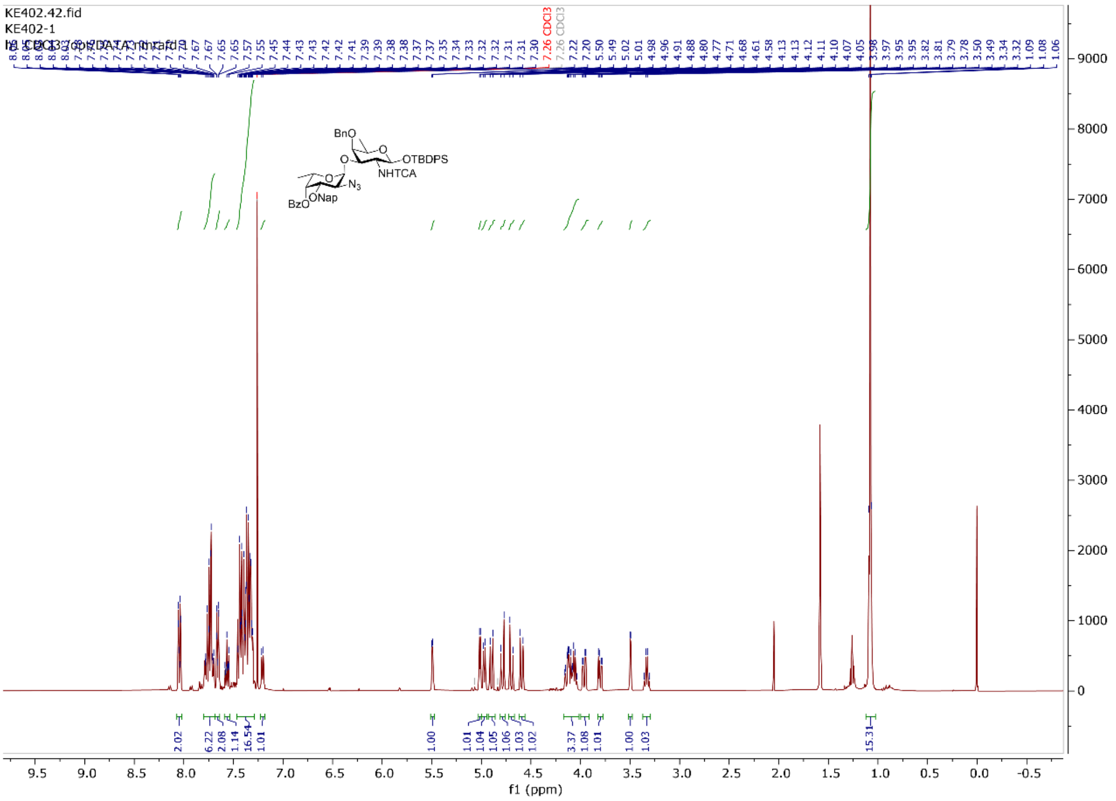
KE402.42.fid
KE402-1
BnO
O
OTBDPS
NHTCA
N_3
BzO
ONap
7.26 CDCl3
f1 (ppm)

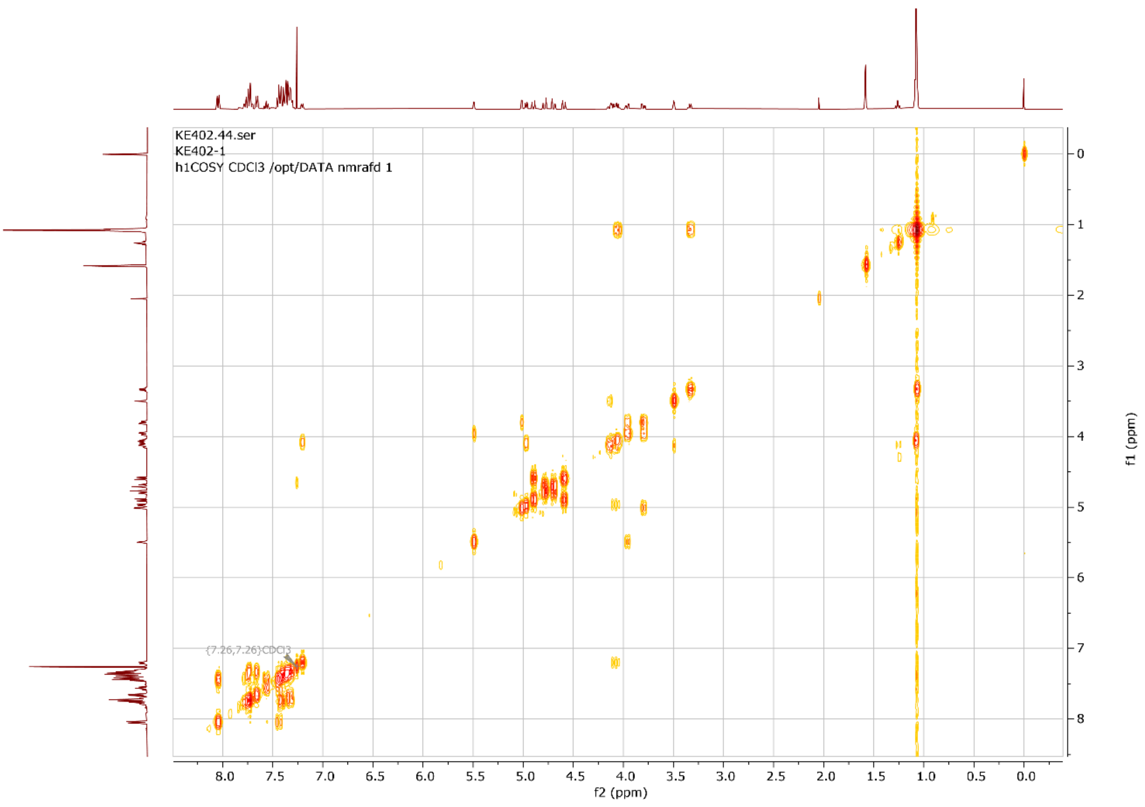
KE402.44.ser
KE402-1
h1COSY CDCl3 /opt/DATA nmrafd 1
(7.26,7.26)CDCl3
f2 (ppm)
f1 (ppm)

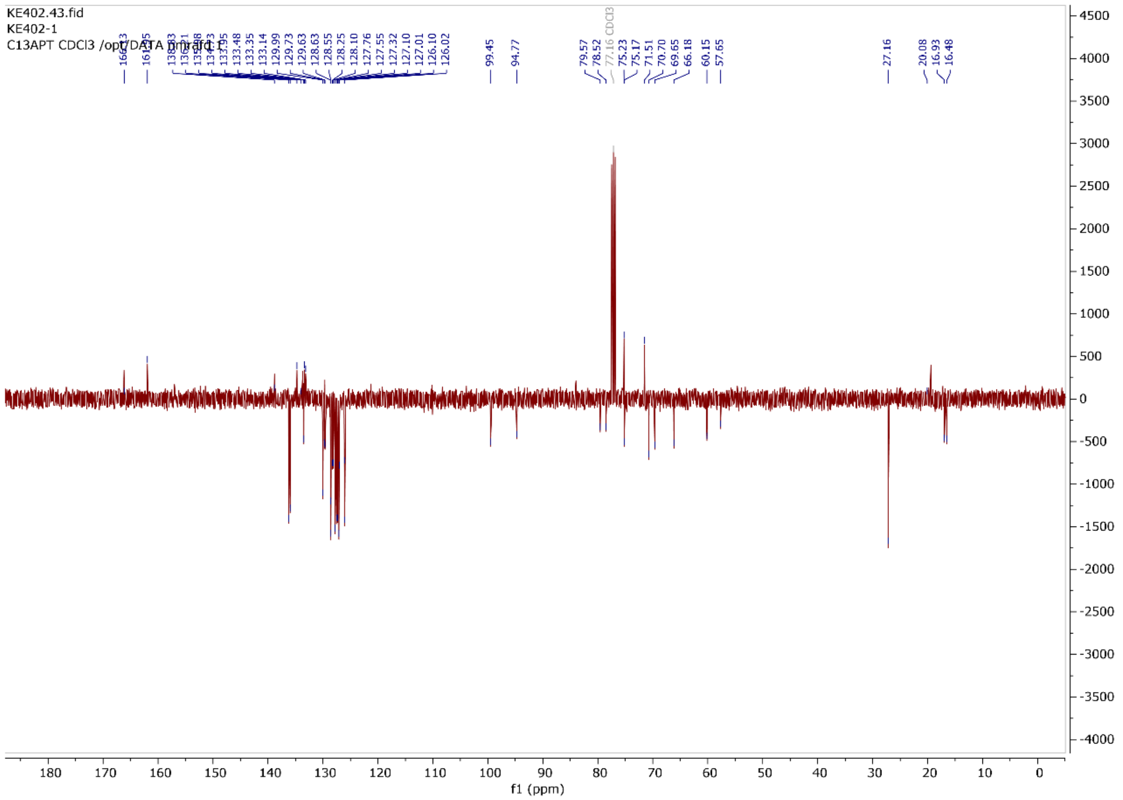
KE402.43.fid
KE402-1
C13APT CDCl3 /opt/DATA nmrafd 1
f1 (ppm)

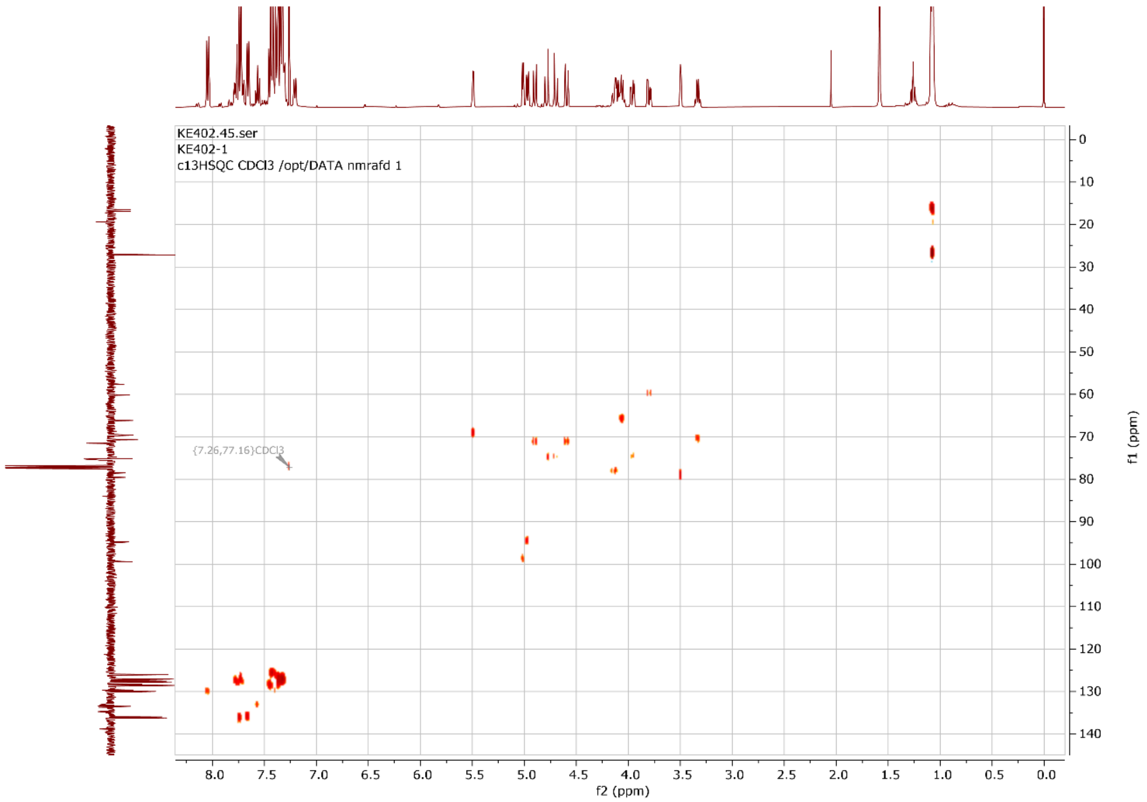
KE402.45.ser
KE402-1
c13HSQC CDCl3 /opt/DATA nmrafd 1
f2 (ppm)
f1 (ppm)

Tert-butyldiphenylsilyl 2-azido-4-_O_-benzoyl-2-deoxy-3-_O_-(2-naphthylmethyl)-α-L-fucopyranosyl-(1→3)-2-deoxy-2-_N_-triflouroacetamide-4-_O_-benzyl-β-D-fucopyranoside (16)

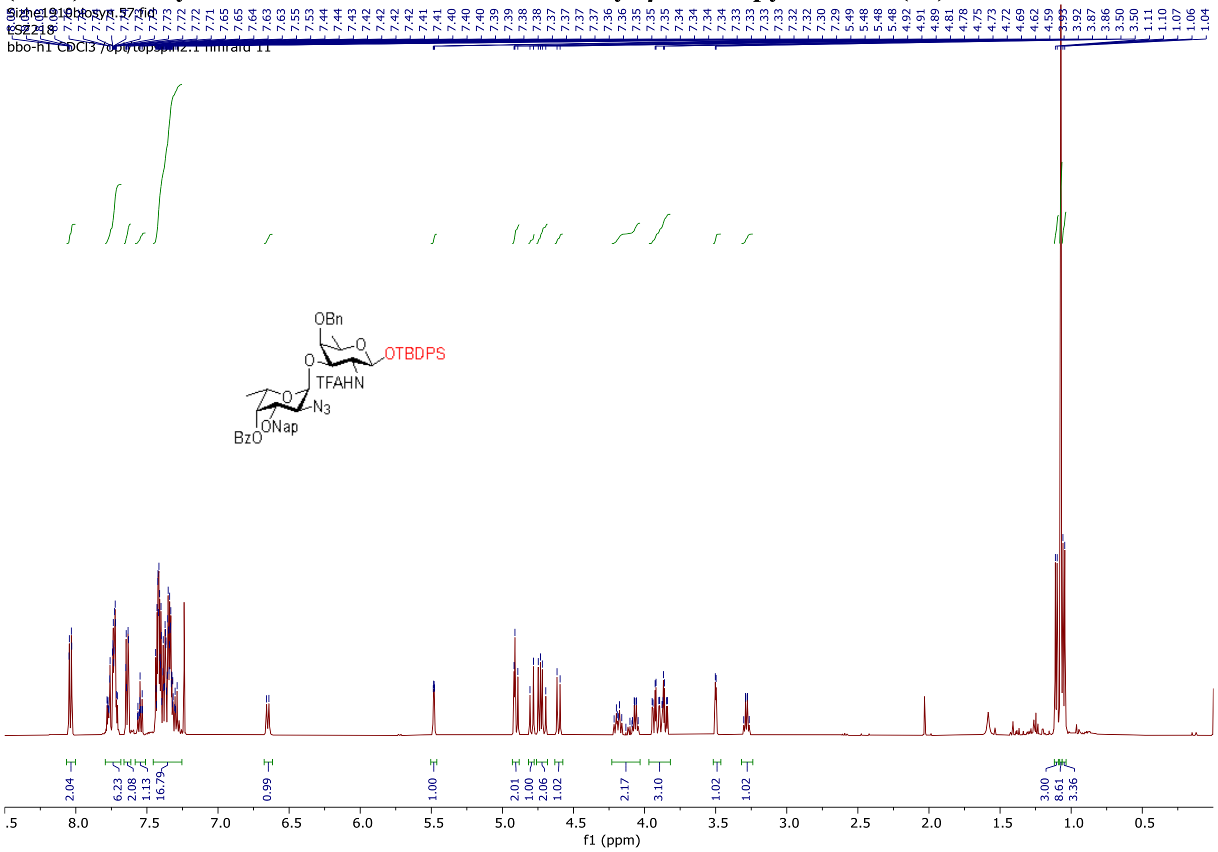

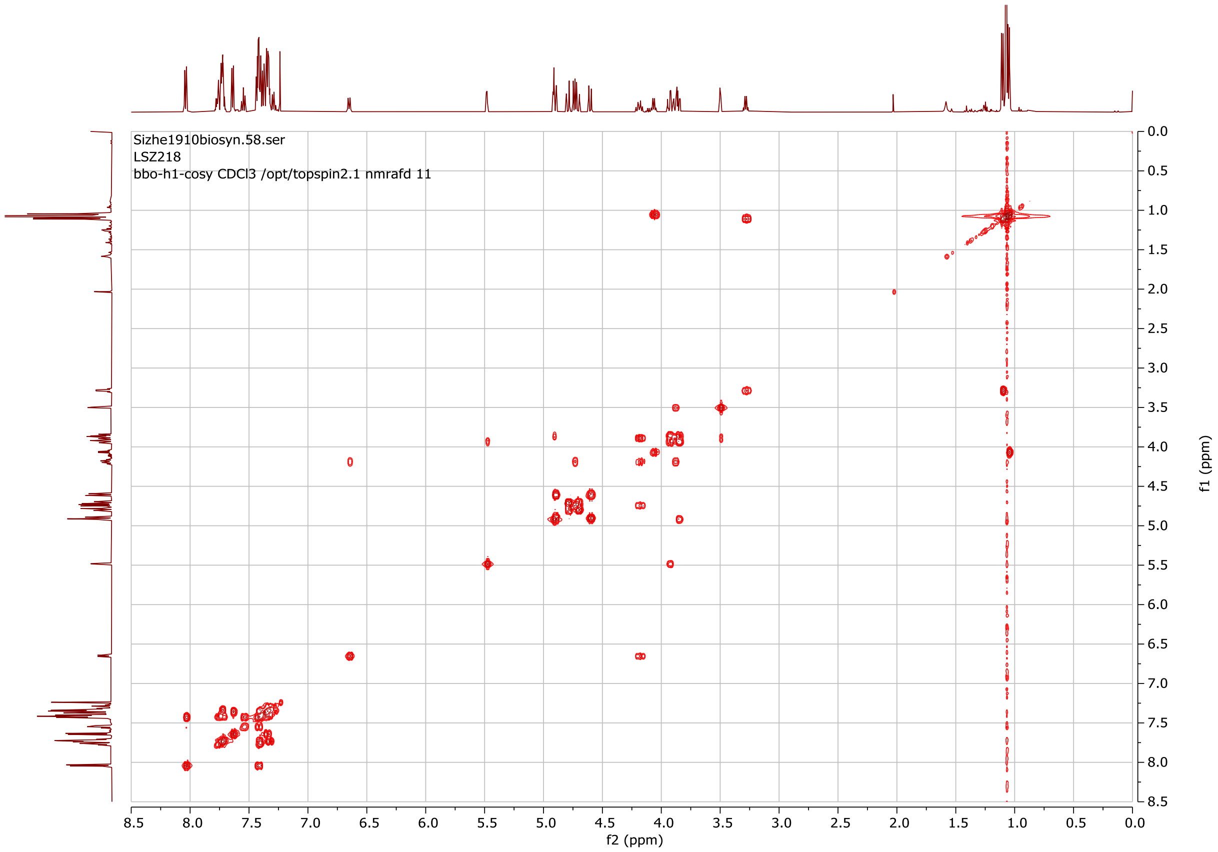
Sizhe1910biosyn.58.ser
LSZ218
bbo-h1-cosy CDCl3 /opt/topspin2.1 nmrafd 11
f2 (ppm)
f1 (ppm)

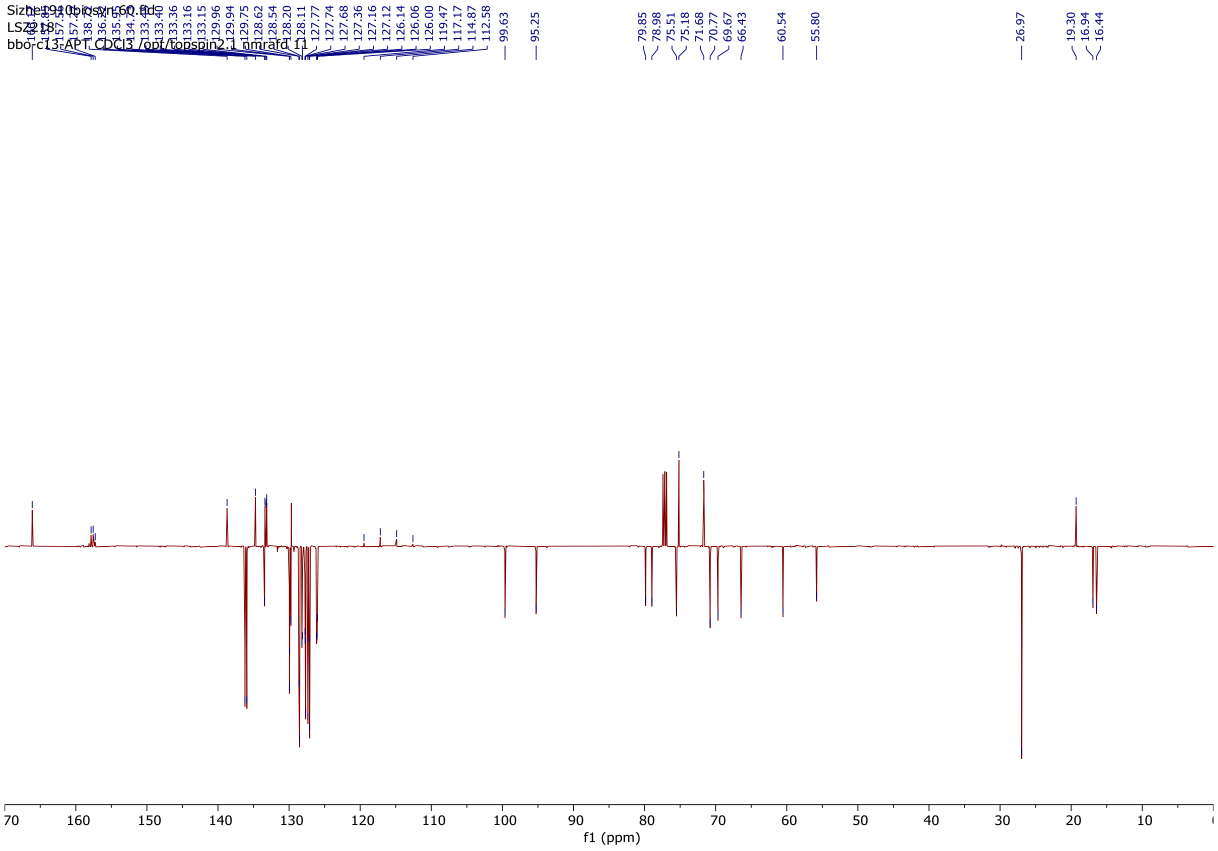
LSZ218
bbo-c13-APT CDCl3 /opt/topspin2.1 nmrafd 11
133.40
133.36
133.16
133.15
129.96
129.94
129.75
128.62
128.54
128.20
128.11
127.77
127.74
127.68
127.36
127.16
127.12
126.14
126.06
126.00
119.47
117.17
114.87
112.58
99.63
95.25
79.85
78.98
75.51
75.18
71.68
70.77
69.67
66.43
60.54
55.80
26.97
19.30
16.94
16.44
f1 (ppm)

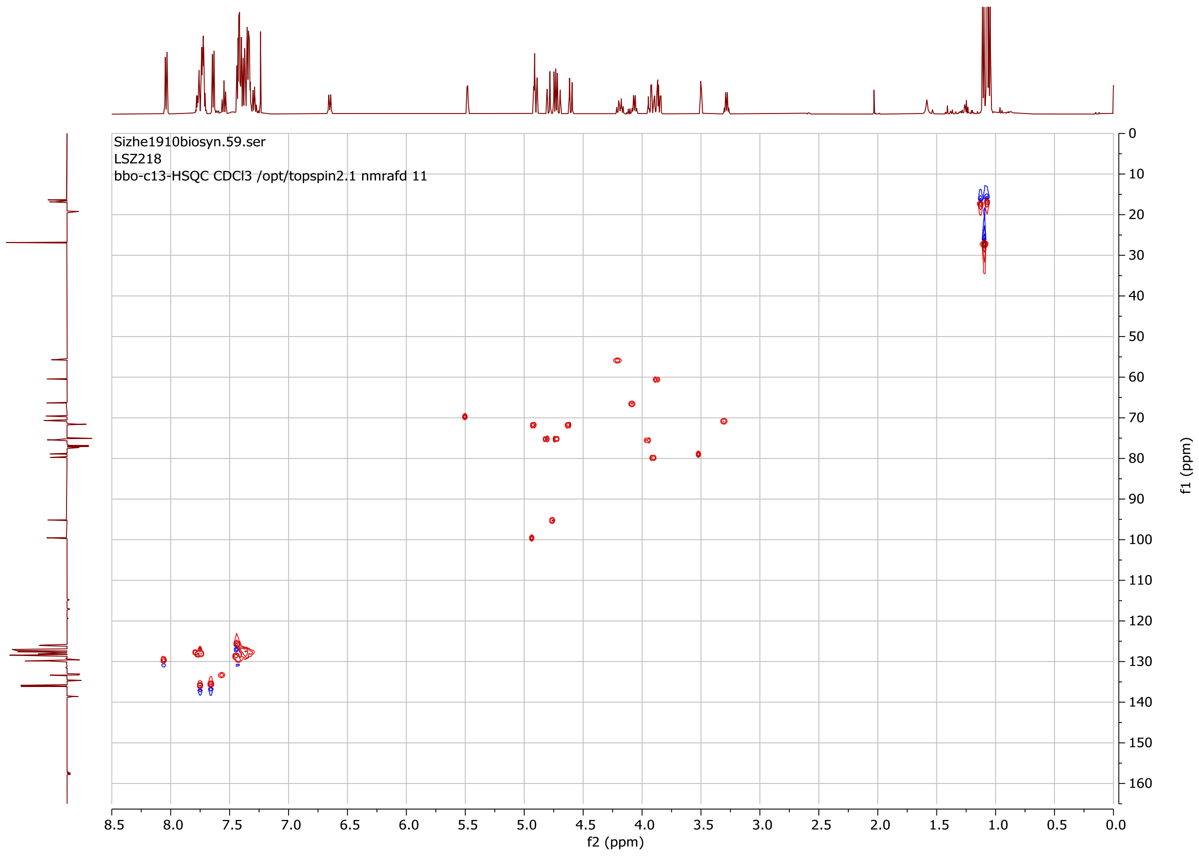

***Tert*-butyldiphenylsilyl 2-azido-2-deoxy-3-*O*-(2-naphthylmethyl)-α-L-fucopyranosyl-(1→3)-2-deoxy-2-*N*-trichloroacetamide-4-*O*-benzyl-β-D-fucopyranoside (17)**

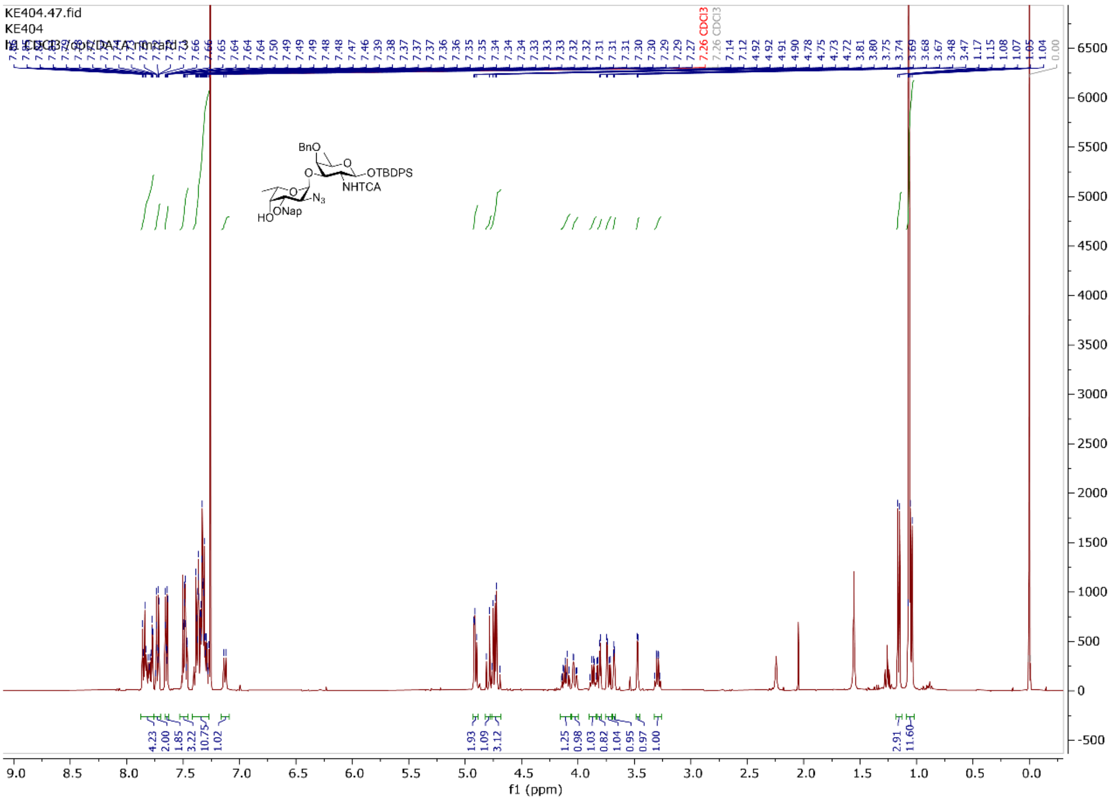
KE404.47.fid
KE404
BnO
O
OTBDPS
NHTCA
N3
HO
ONap
f1 (ppm)

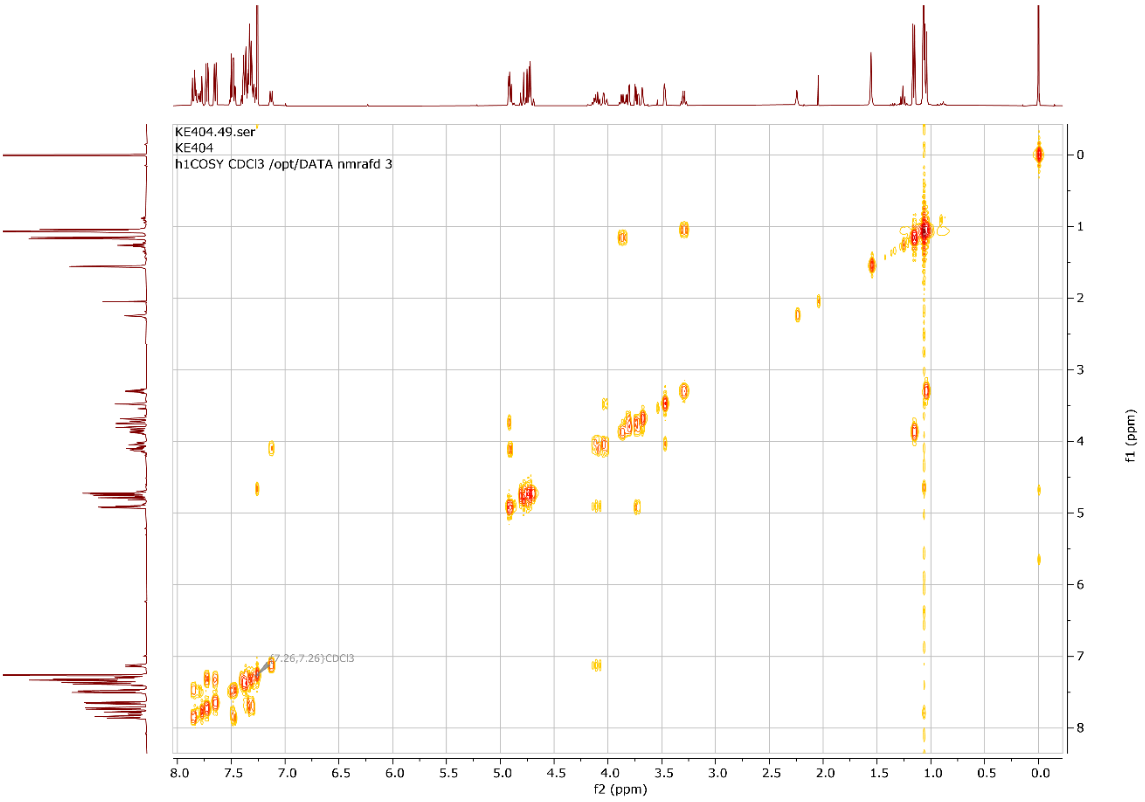
KE404.49.ser
KE404
h1COSY CDCl3 /opt/DATA nmrafd 3
f2 (ppm)
f1 (ppm)

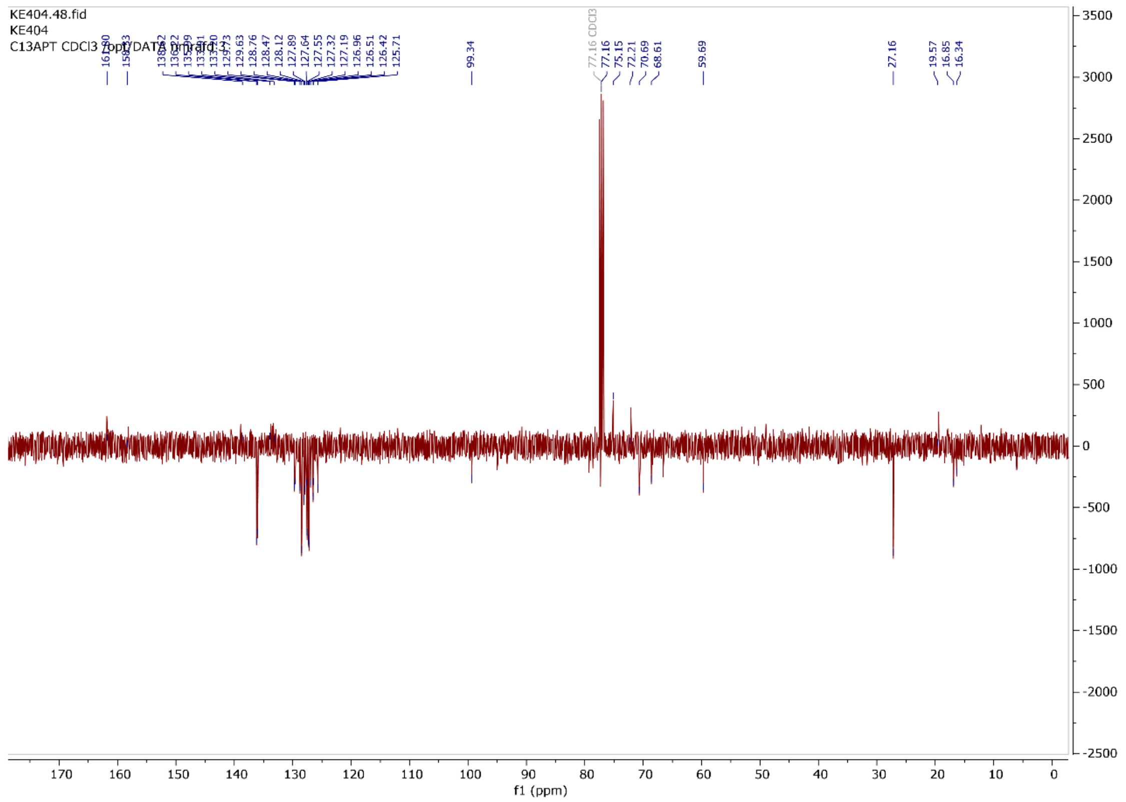
KE404.48.fid
KE404
C13APT CDCl3 /opt/DATA nmrafd 3
99.34
77.16 CDCl3
77.16
75.15
72.21
70.69
68.61
59.69
27.16
19.57
16.85
16.34
f1 (ppm)

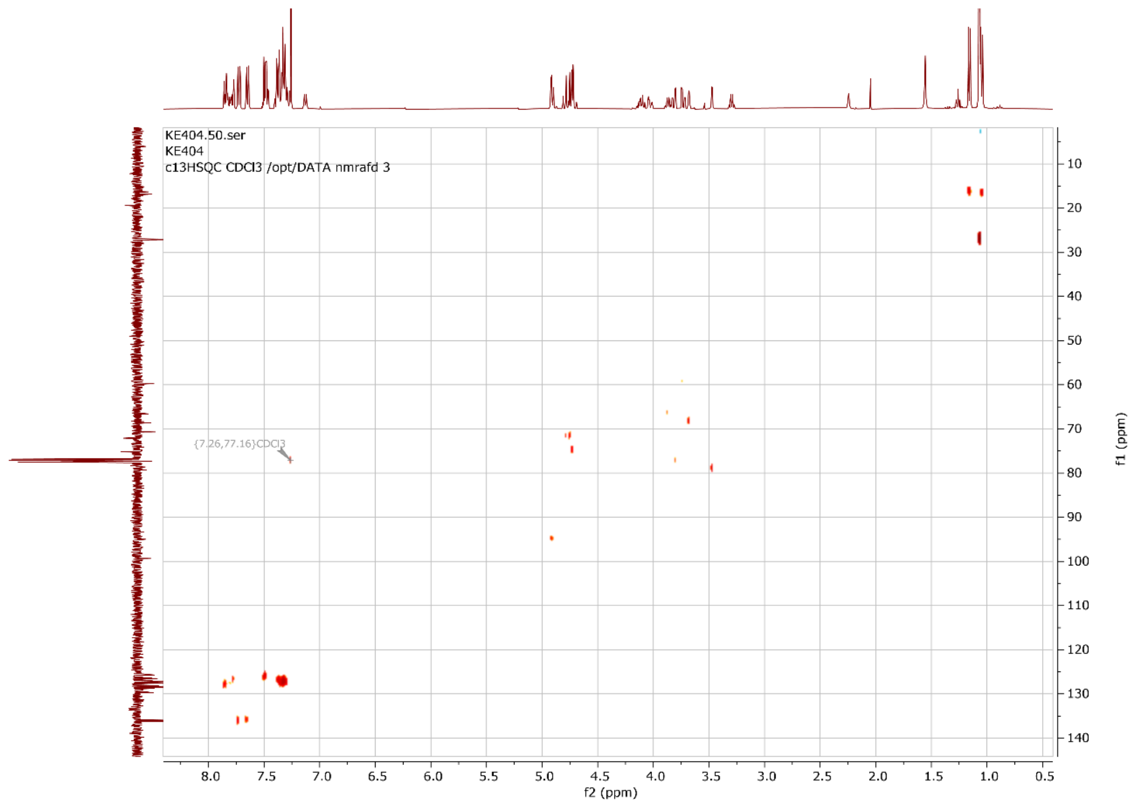
KE404.50.ser
KE404
c13HSQC CDCl3 /opt/DATA nmrafd 3
{7.26,77.16}CDCl3
f2 (ppm)
f1 (ppm)

Tert-butyldiphenylsilyl 2-azido-2-deoxy-α-L-fucopyranosyl-(1→3)-2-deoxy-2-_N_-trichloroacetamide-4-_O_-benzyl-β-D-fucopyranoside (18)

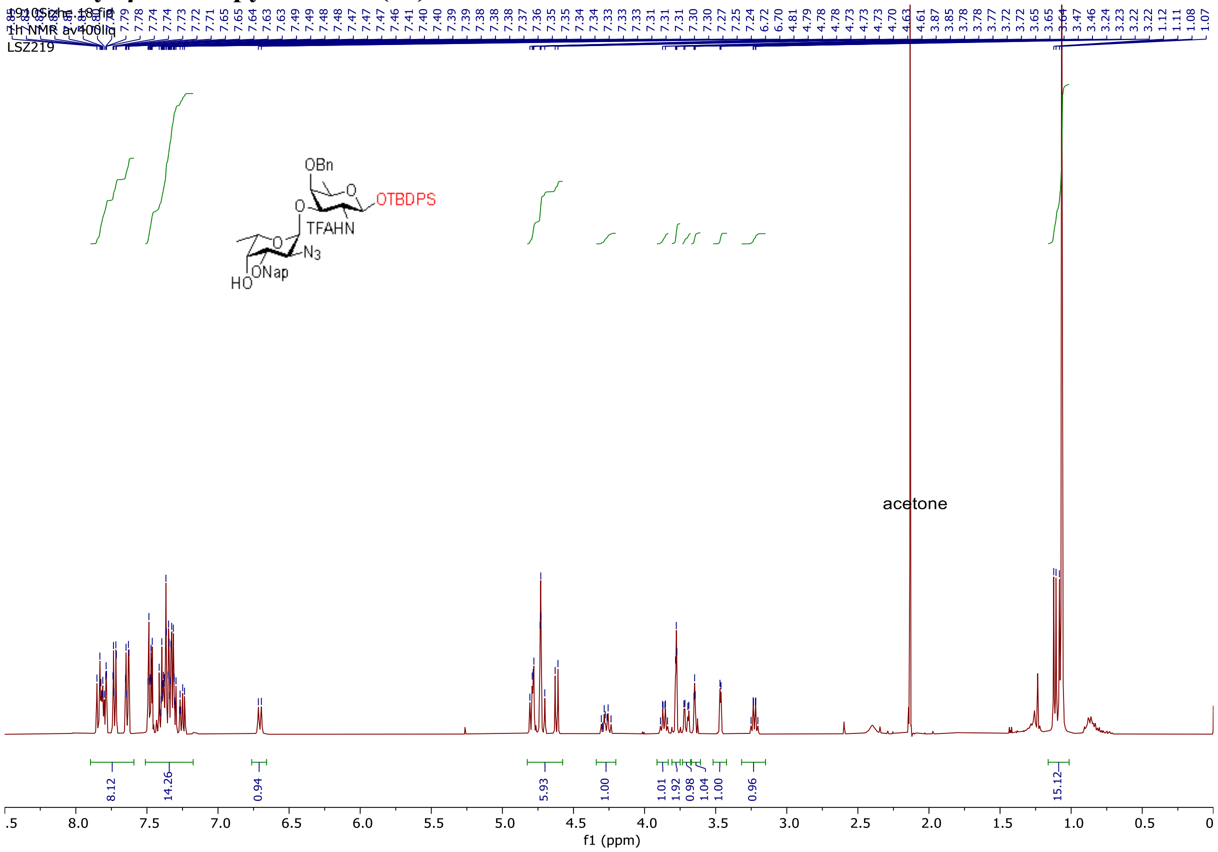

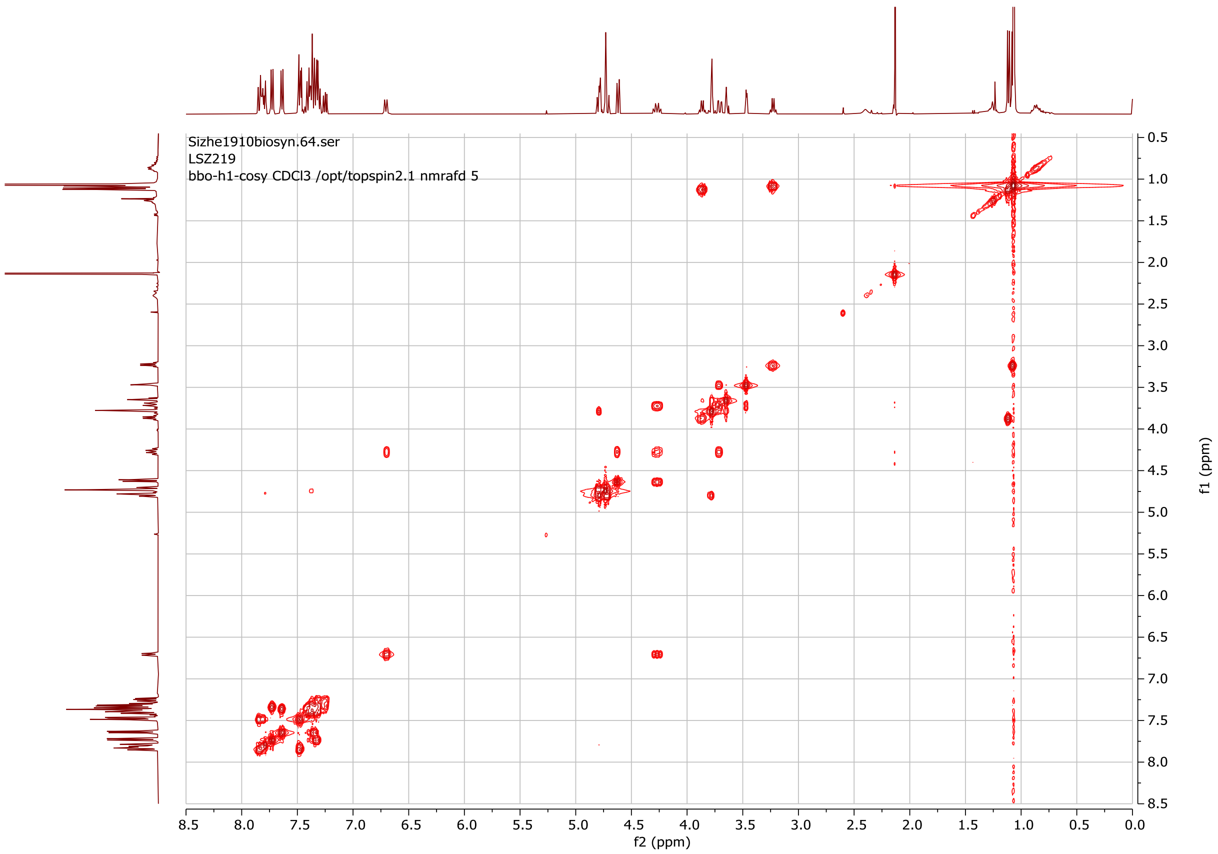
Sizhe1910biosyn.64.ser
LSZ219
bbo-h1-cosy CDCl3 /opt/topspin2.1 nmrafd 5
f2 (ppm)
f1 (ppm)

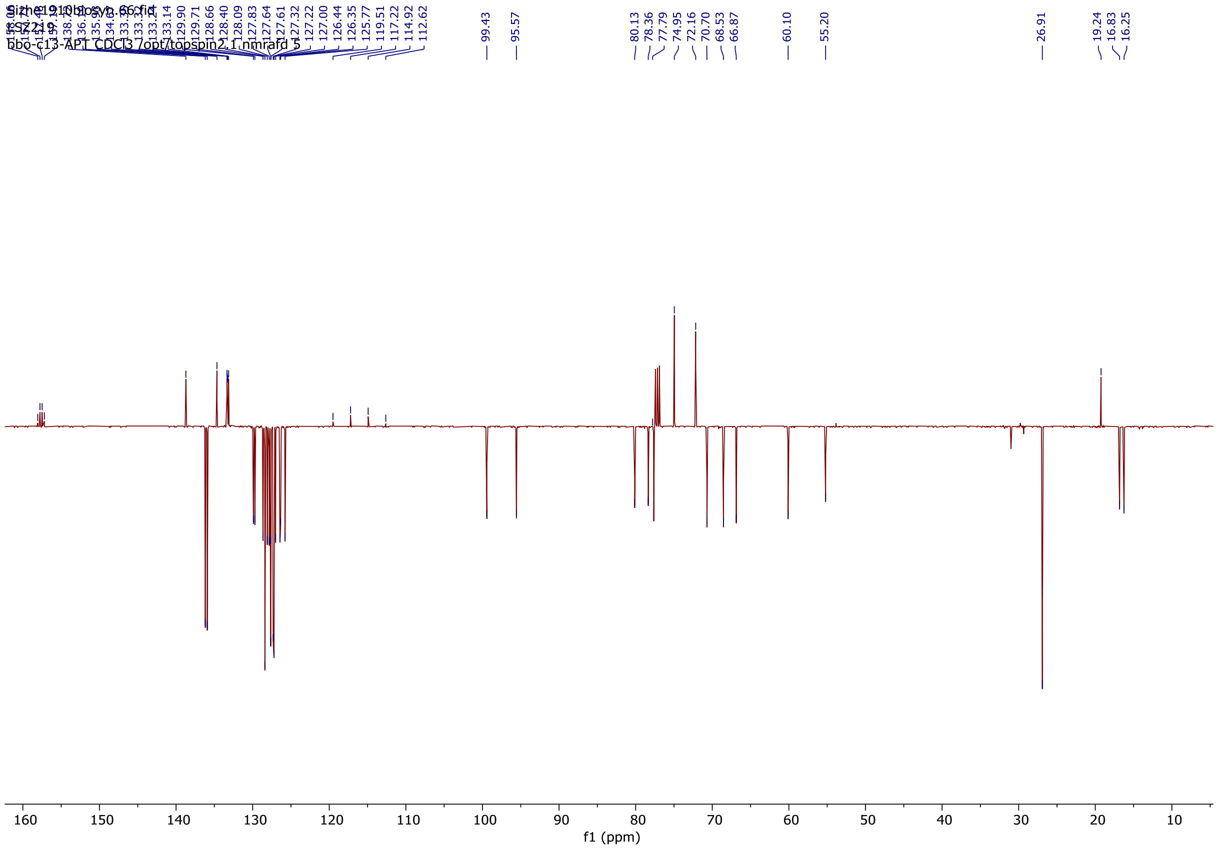
LSZ219
bbo-c13-APT CDCl3 /opt/topspin2.1 nmrafd 5
133.14
129.90
129.71
128.66
128.40
128.09
127.83
127.64
127.61
127.32
127.22
127.00
126.44
126.35
125.77
119.51
117.22
114.92
112.62
99.43
95.57
80.13
78.36
77.79
74.95
72.16
70.70
68.53
66.87
60.10
55.20
26.91
19.24
16.83
16.25
f1 (ppm)

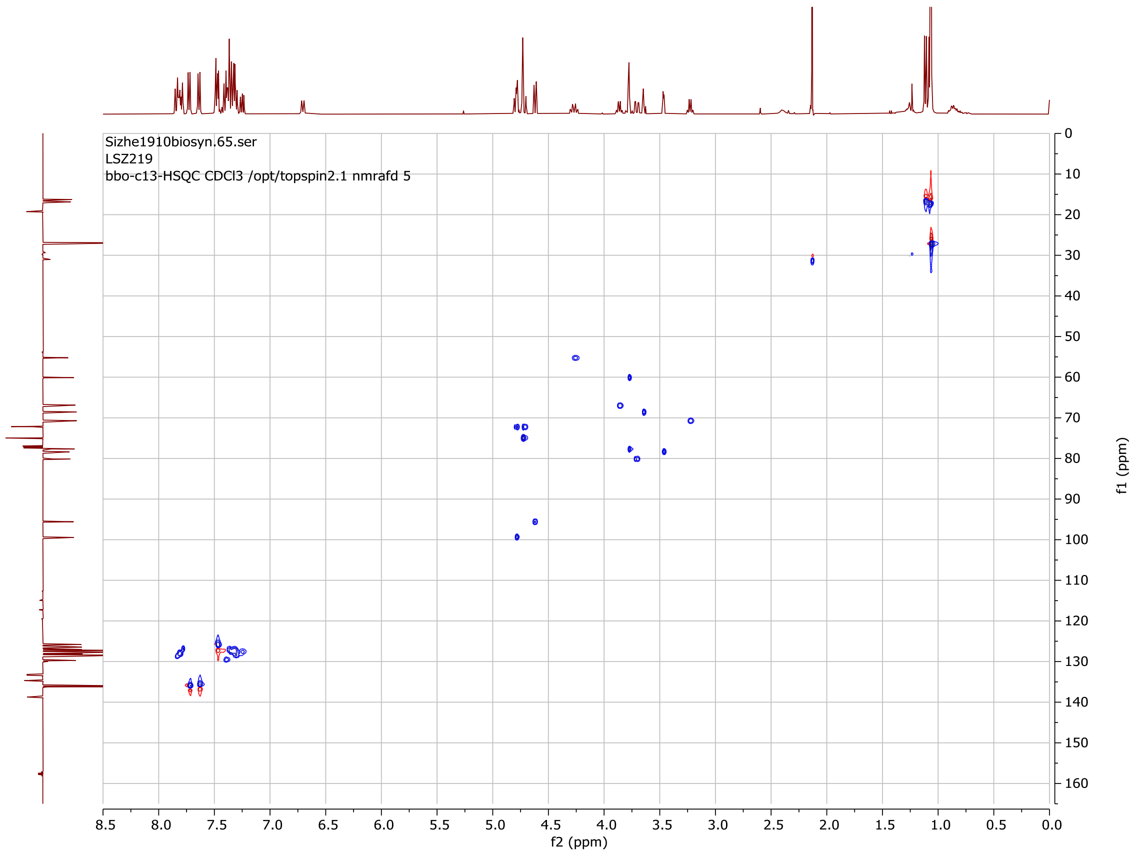

Tert-butyldiphenylsilyl (Benzyl (2-azido-3,4-di-_O_-benzyl-2-deoxy-β-D-mannopyranosiduronsyl)-(1→4)-2-azido-2-deoxy-3-_O_-(2-naphthylmethyl)-α-L-fucopyranosyl-(1→3)-4-_O_-benzyl-2-deoxy-2-_N_-trichloroacetamide-β-D-fucopyranoside (7)

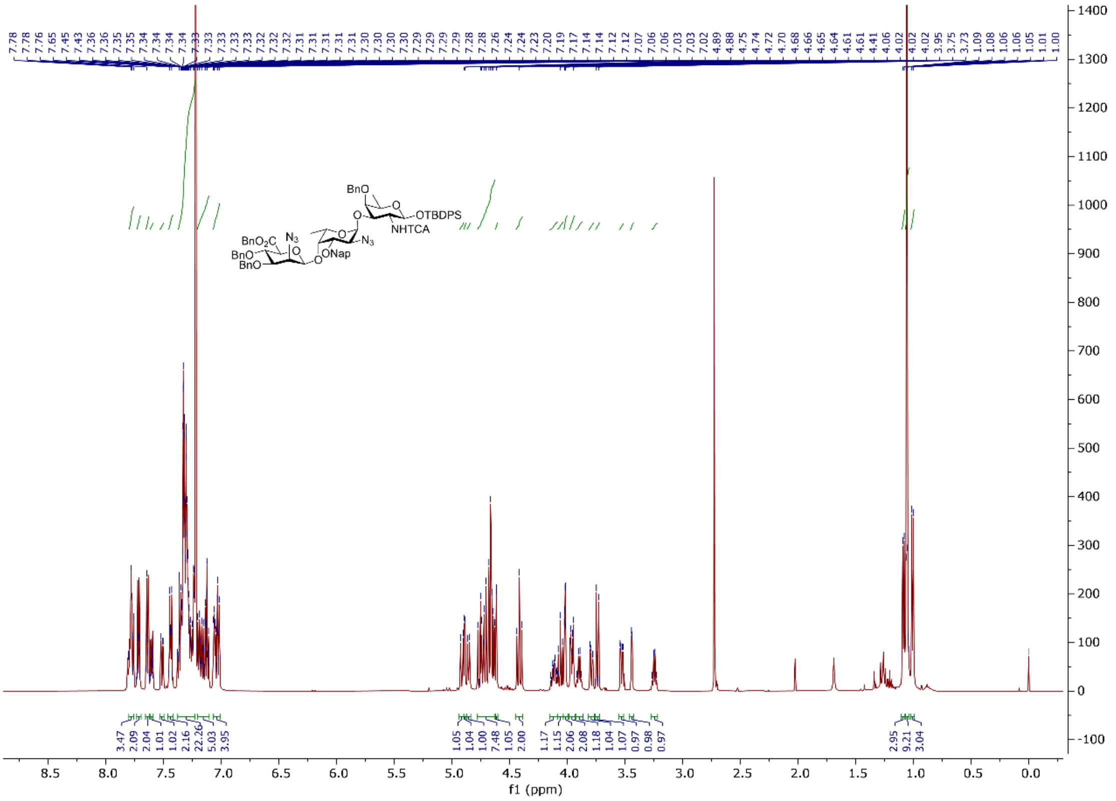
BnO
OTBDPS
NHTCA
BnO_2C
N_3
BnO
BnO
ONap
f1 (ppm)

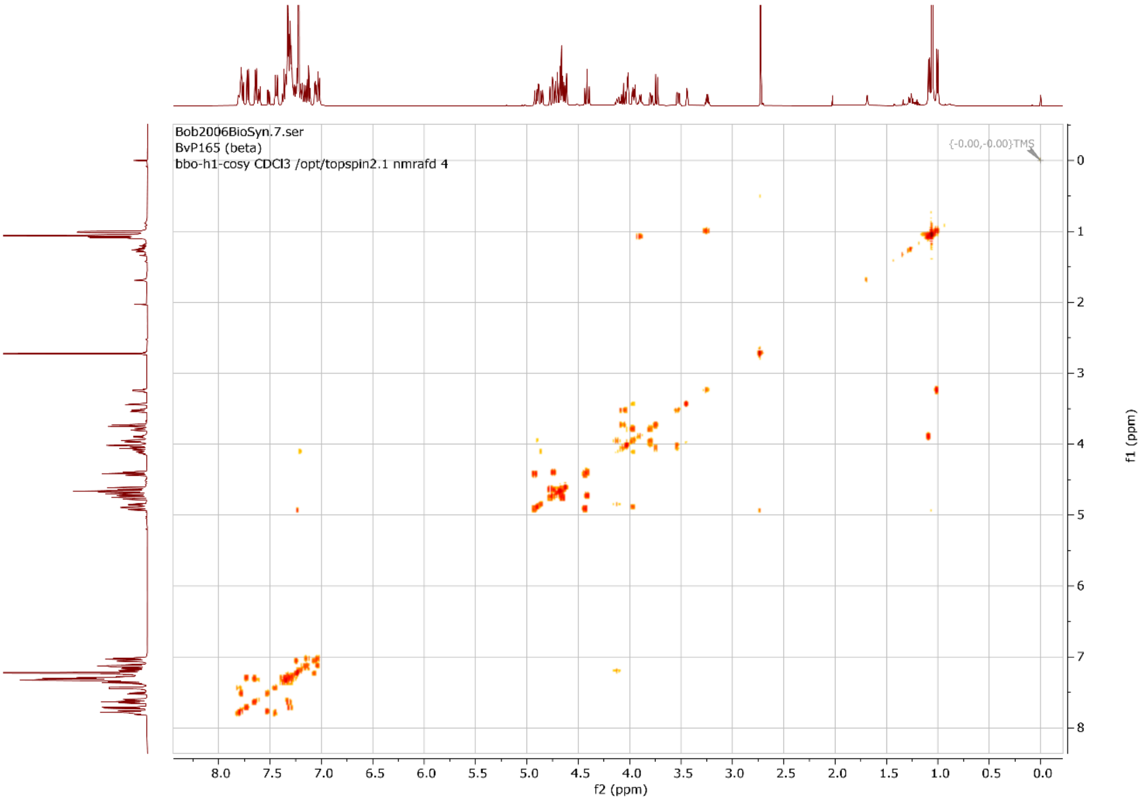
Bob2006BioSyn.7.ser
BvP165 (beta)
bbo-h1-cosy CDCl3 /opt/topspin2.1 nmrafd 4
{-0.00,-0.00}TMS
f2 (ppm)
f1 (ppm)

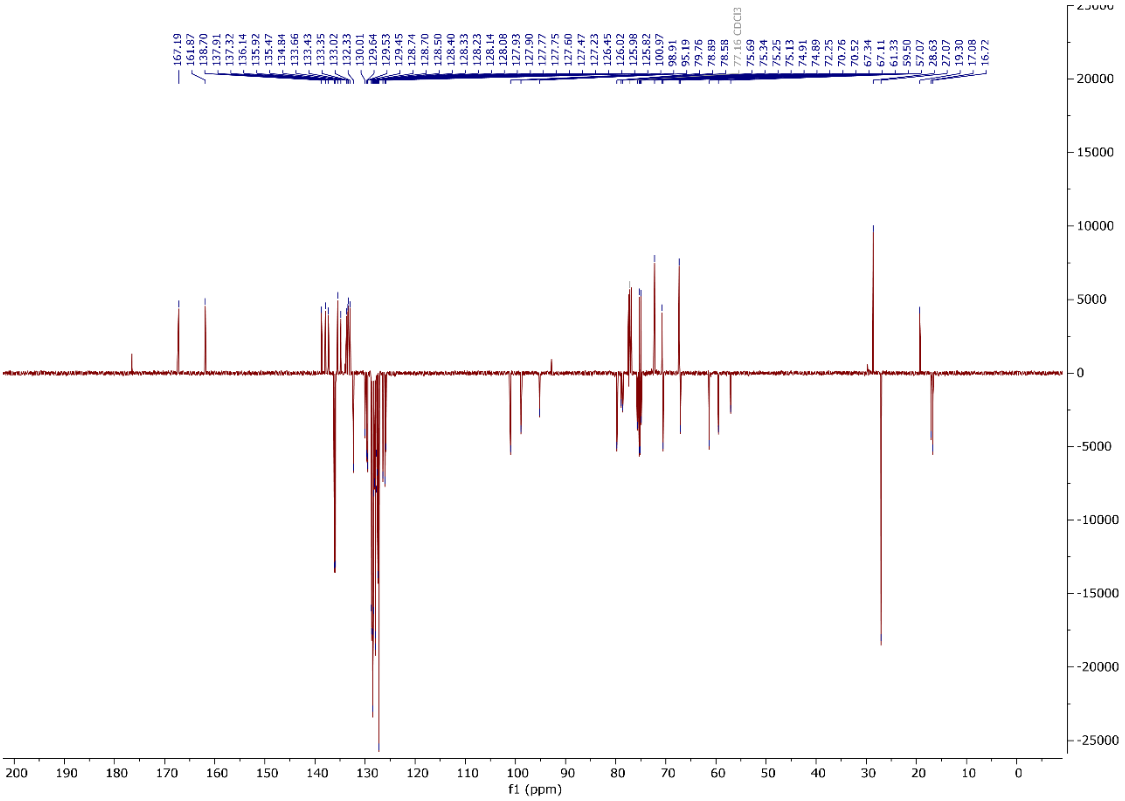
167.19
161.87
138.70
137.91
137.32
136.14
135.92
135.47
134.84
133.66
133.43
133.35
133.02
132.33
130.01
129.64
129.53
129.45
128.74
128.70
128.50
128.40
128.33
128.23
128.14
128.08
127.93
127.90
127.77
127.75
127.60
127.47
127.23
126.45
126.02
125.98
125.82
100.97
98.91
95.19
79.76
78.89
78.58
77.16 CDCl3
75.69
75.34
75.25
75.13
74.91
74.89
72.25
70.76
70.52
67.34
67.11
61.33
59.50
57.07
28.63
27.07
19.30
17.08
16.72
f1 (ppm)

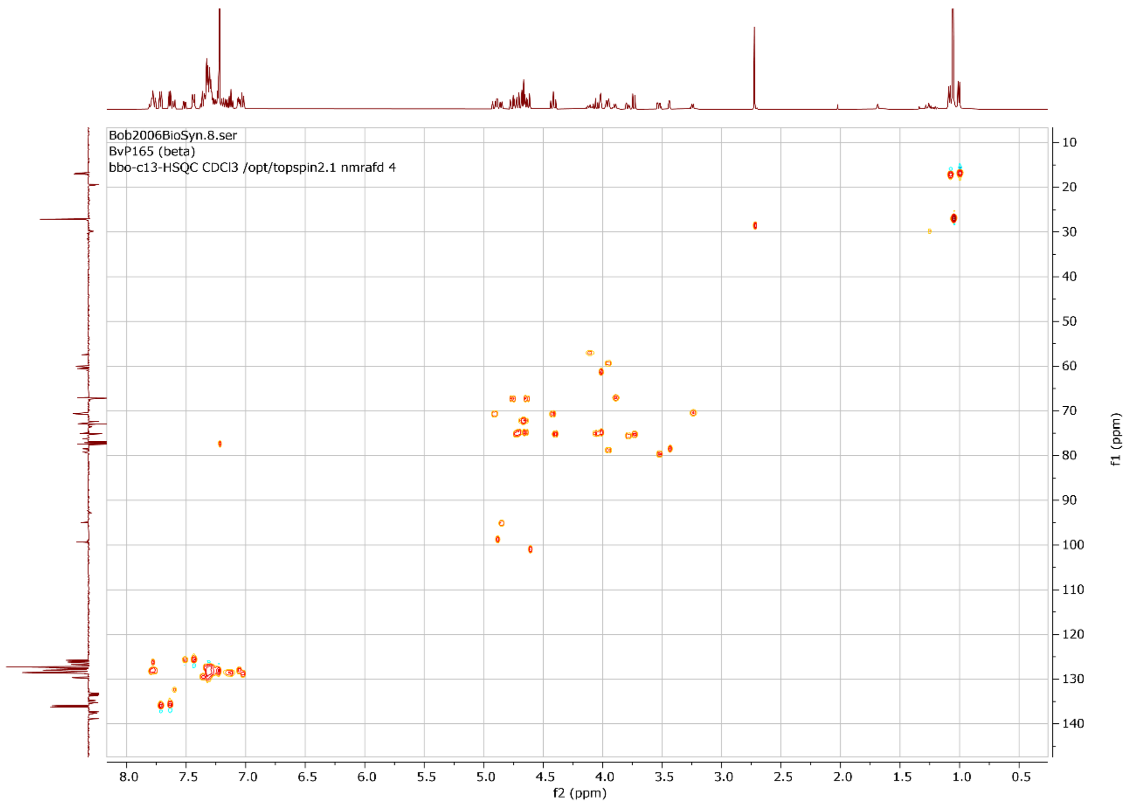
Bob2006BioSyn.8.ser
BvP165 (beta)
bbo-c13-HSQC CDCl3 /opt/topspin2.1 nmrafd 4
f2 (ppm)
f1 (ppm)

(Benzyl (2-azido-3,4-di-*O*-benzyl-2-deoxy-β-D-mannopyranosiduronsyl)-(1→4)-2-azido-2-deoxy 3-*O*-(2-naphthylmethyl)-α-L-fucopyranosyl-(1→3)-4-*O*-benzyl-2-deoxy-2-*N*-trichloroacetamide-α-D-fucopyranose (19)

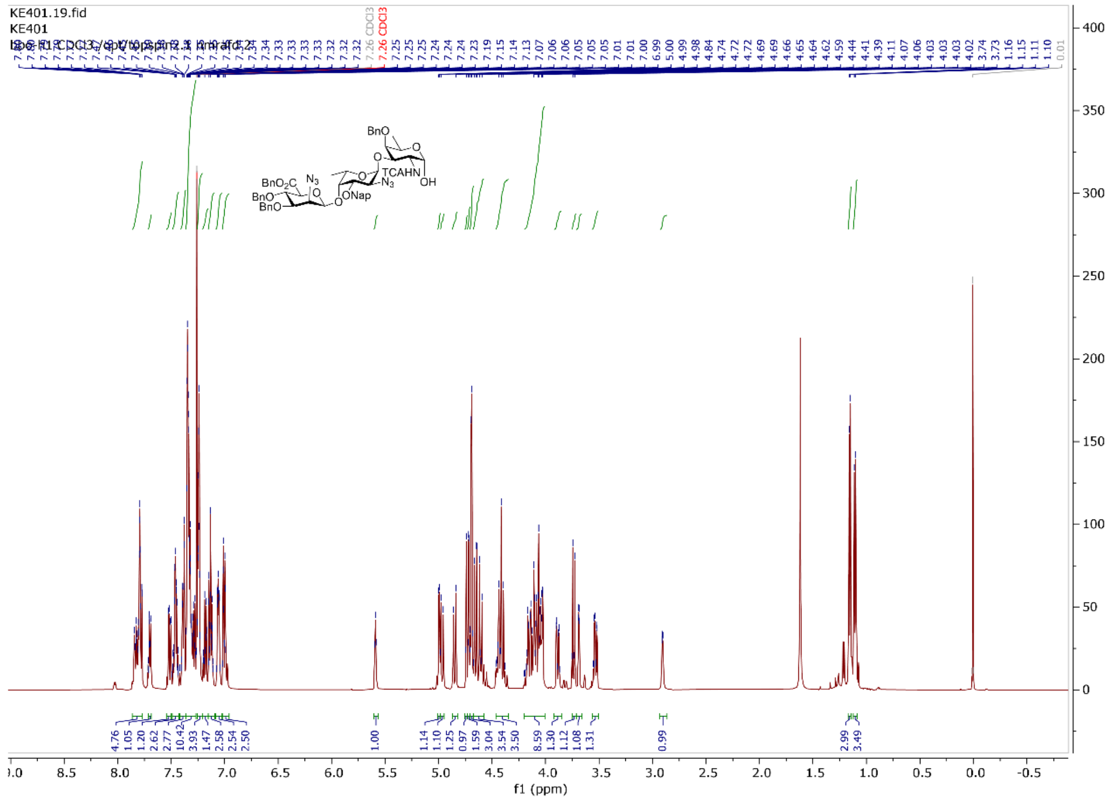

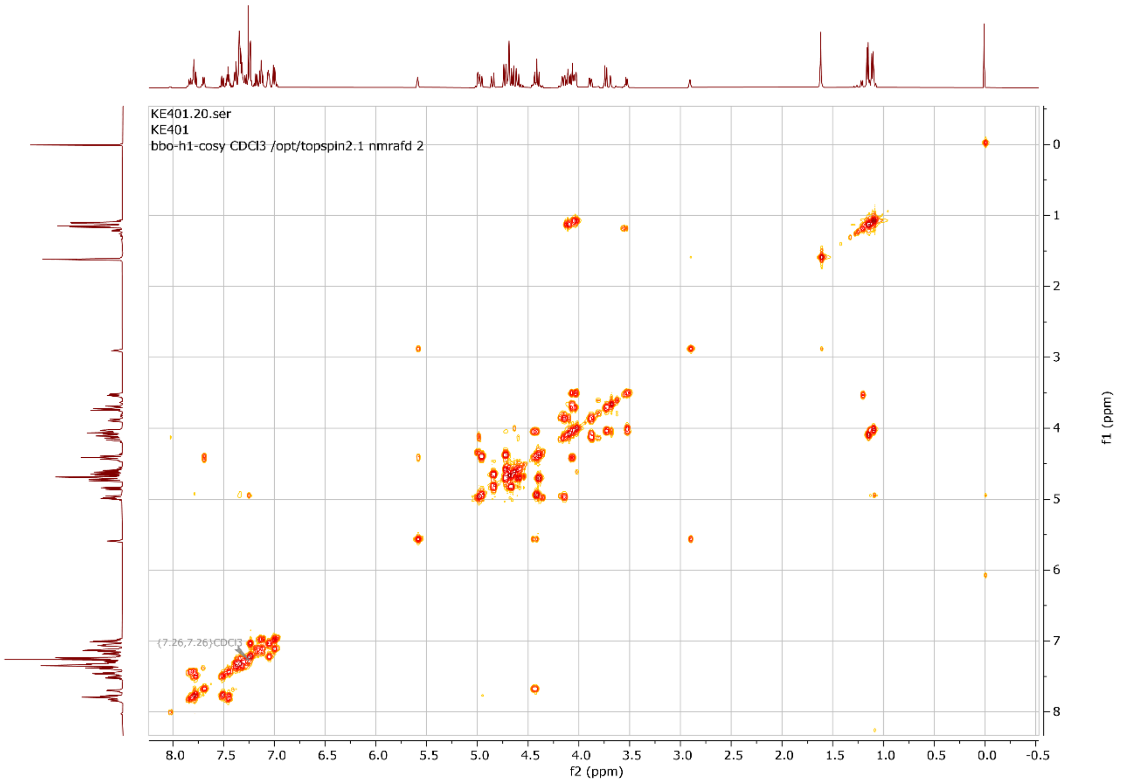
KE401.20.ser
KE401
bbo-h1-cosy CDCl3 /opt/topspin2.1 nmrafd 2
(7.26,7.26)CDCl3
f2 (ppm)
f1 (ppm)

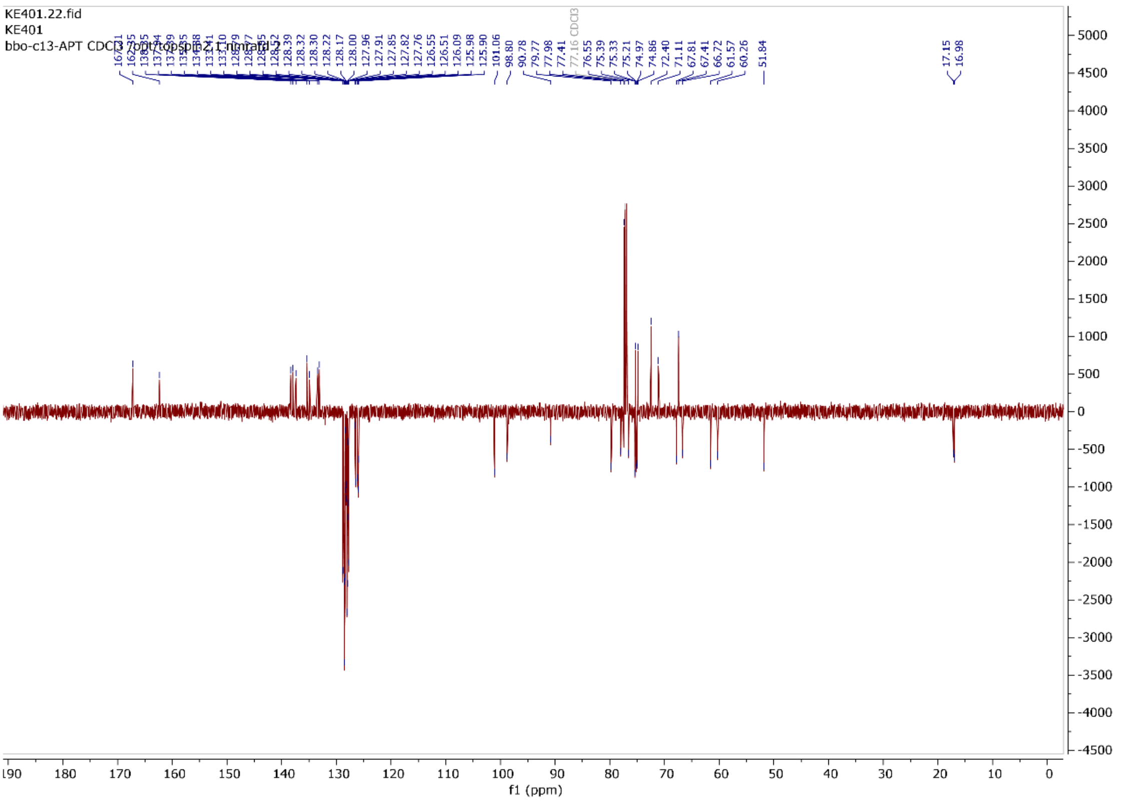
KE401.22.fid
KE401
bbo-c13-APT CDCl3 /opt/topspin2.1 nmrafd 2
128.39
128.32
128.30
128.22
128.17
128.00
127.96
127.91
127.85
127.82
127.76
126.55
126.51
126.09
125.98
125.90
101.06
98.80
90.78
79.77
77.98
77.41
77.16 CDCl3
76.55
75.39
75.33
75.21
74.97
74.86
72.40
71.11
67.81
67.41
66.72
61.57
60.26
51.84
17.15
16.98
f1 (ppm)

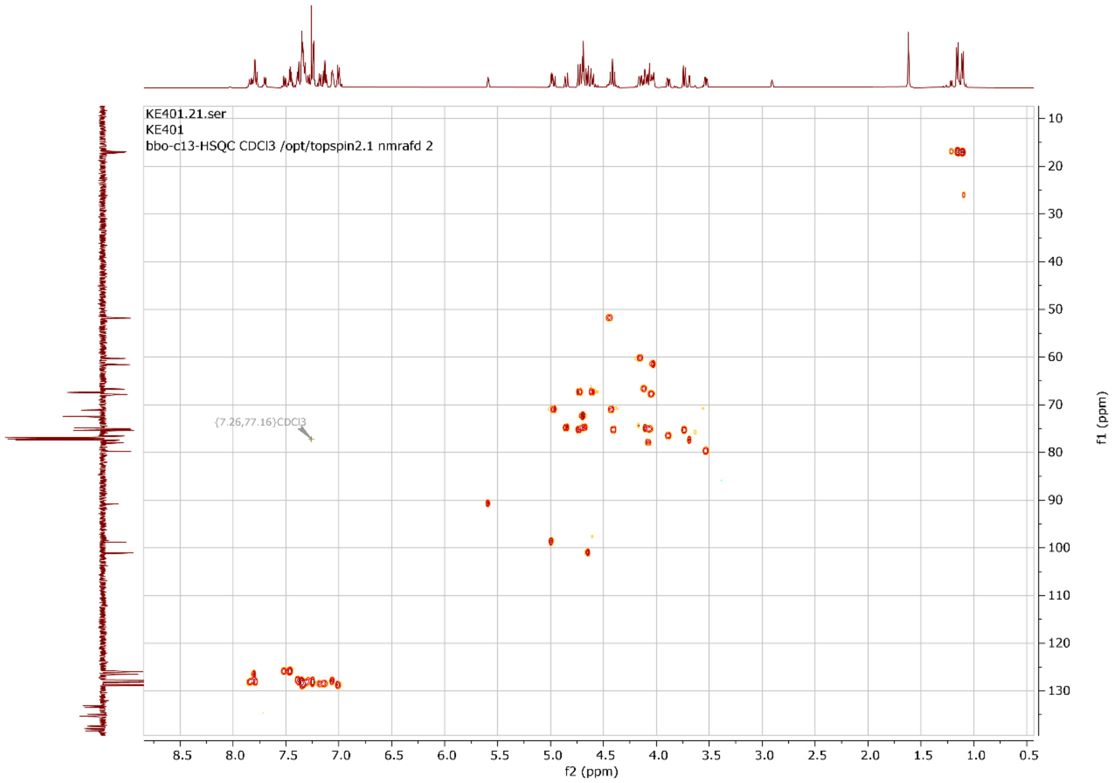

(Benzyl (2-azido-3,4-di-*O*-benzyl-2-deoxy-β-D-mannopyranosiduronsyl)-(1→4)-2-azido-2-deoxy 3-*O*-(2-naphthylmethyl)-α-L-fucopyranosyl-(1→3)-4-*O*-benzyl-2-deoxy-2-*N*-trichloroacetamide-1-*O*-(*N*-phenyl-2,2,2-trifluoroacetimidoyl)-α-D-fucopyranose (20)

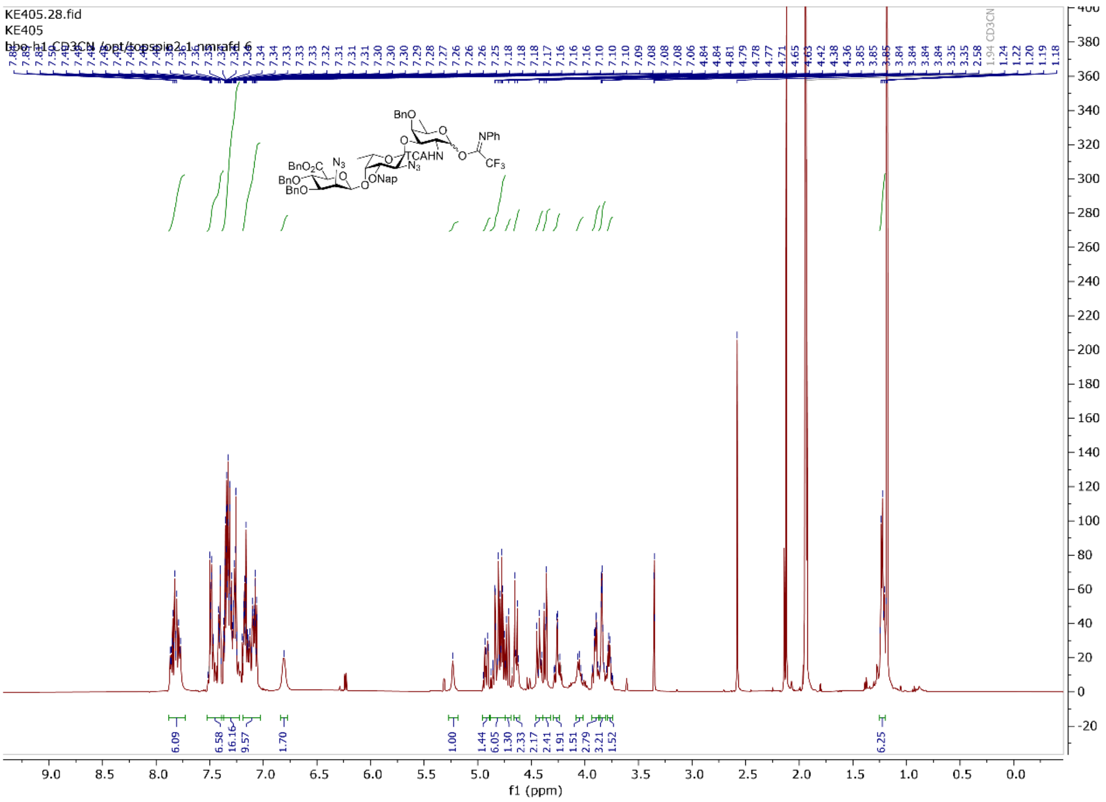
KE405.28.fid
KE405
bbo-h1 CD3CN /opt/topspin2.1 nmrafd 6
f1 (ppm)

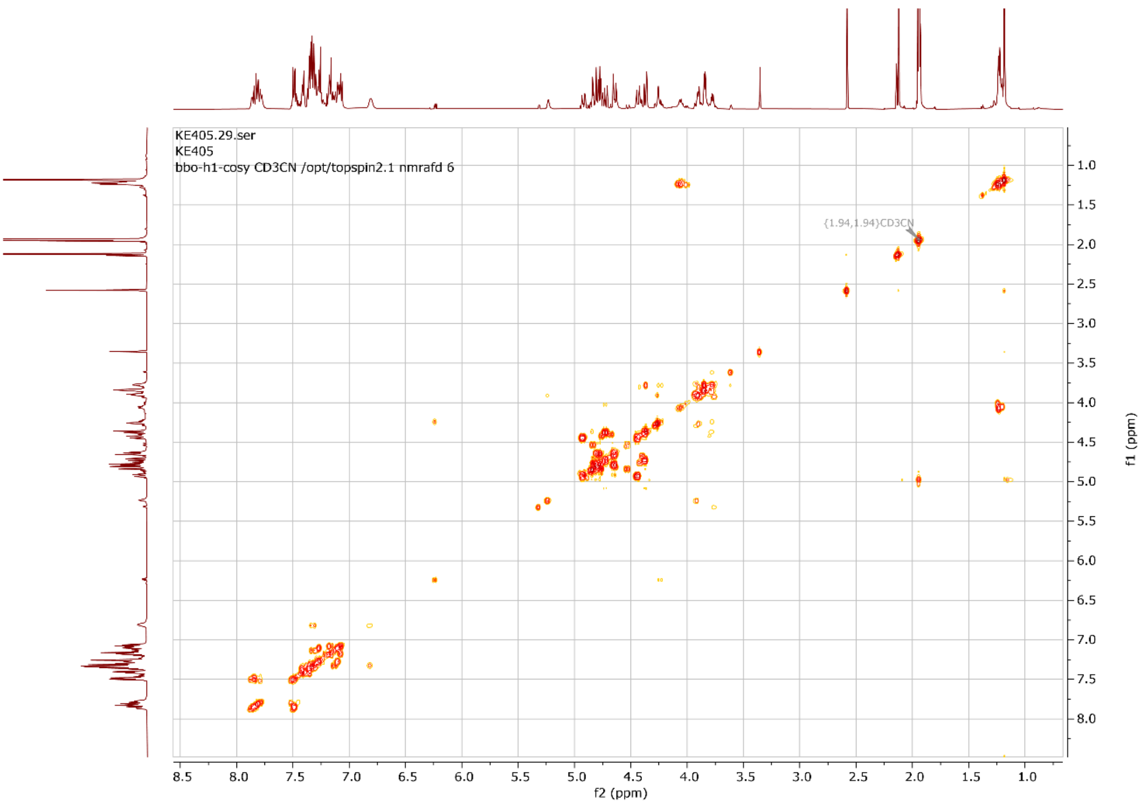
KE405.29.ser
KE405
bbo-h1-cosy CD3CN /opt/topspin2.1 nmrafd 6
{1.94,1.94}CD3CN
f2 (ppm)
f1 (ppm)

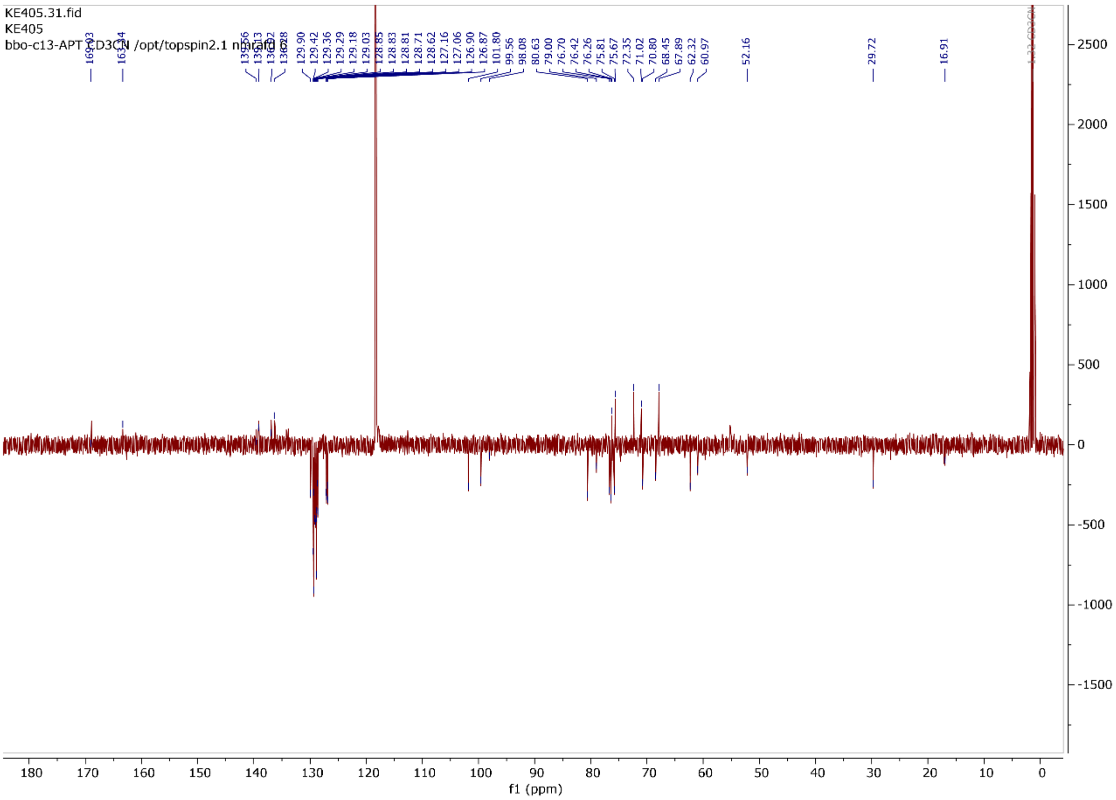
KE405.31.fid
KE405
bbo-c13-APT CD3CN /opt/topspin2.1 nmrafd 6
52.16
29.72
16.91
f1 (ppm)

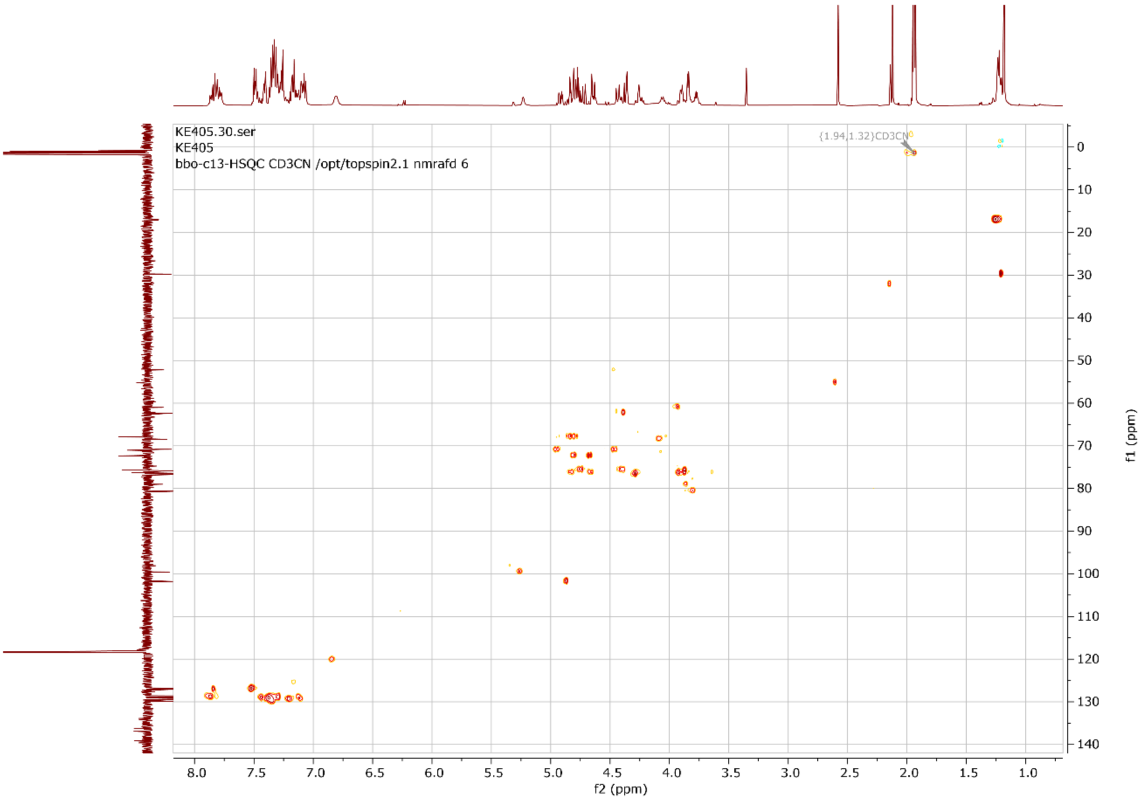
KE405.30.ser
KE405
bbo-c13-HSQC CD3CN /opt/topspin2.1 nmrafd 6
f2 (ppm)
f1 (ppm)

5-(Benzyl(benzyloxycarbonyl)amino)pentyl (Benzyl (2-azido-3,4-di-*O*-benzyl-2-deoxy-β-D-mannopyranosiduronsyl)-(1→4)-2-azido-2-deoxy-3-*O*-(2-naphthylmethyl)-α-L-fucopyranosyl-(1→3)-4-*O*-benzyl-2-deoxy-2-*N*-trichloroacetamide-α-D-fucopyranoside (22)

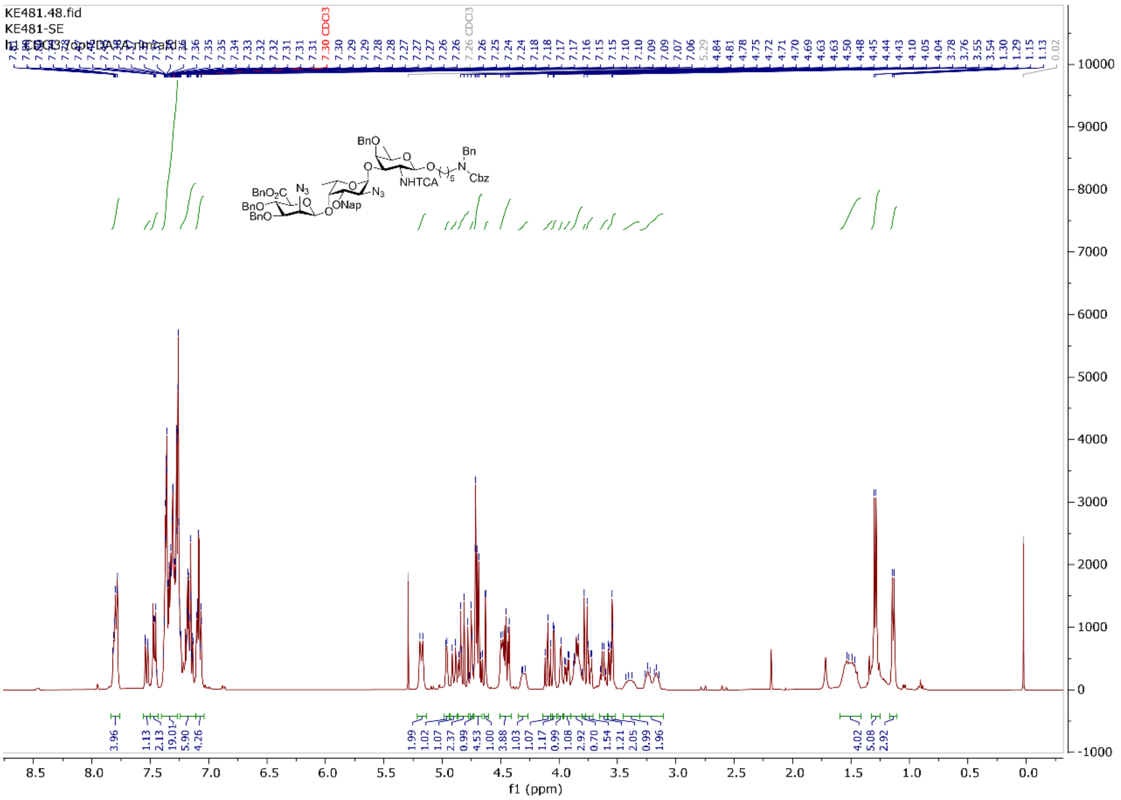

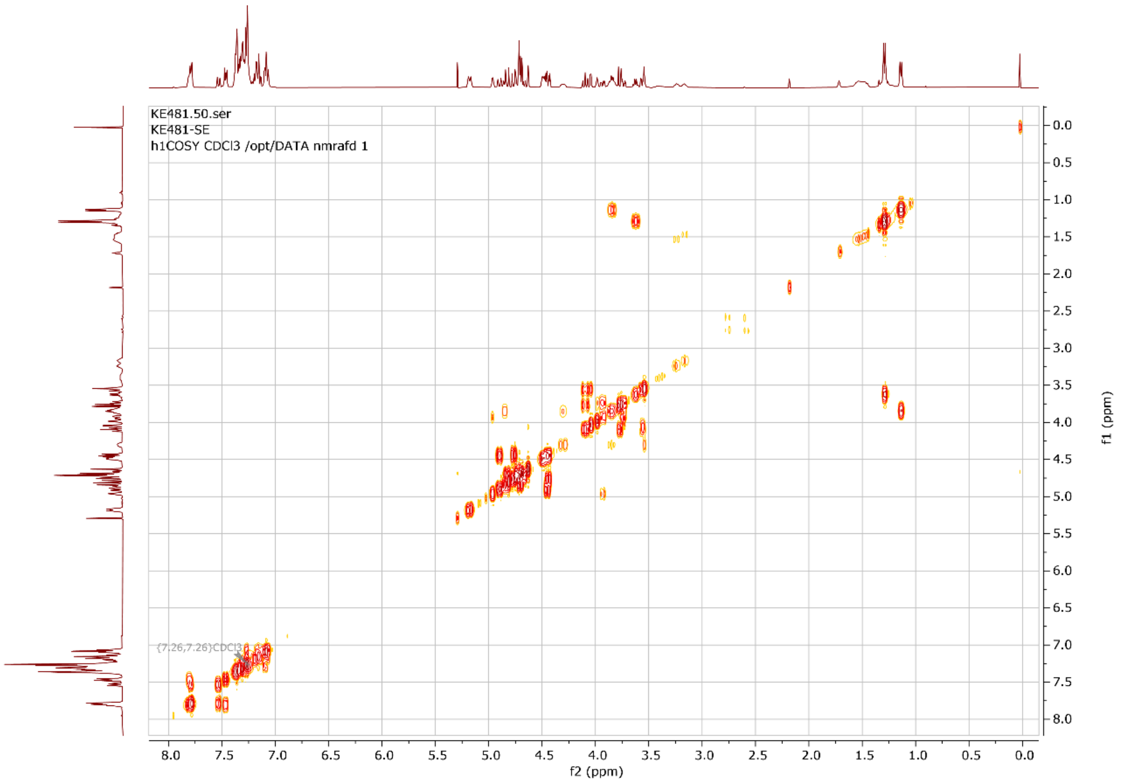
KE481.50.ser
KE481-SE
h1COSY CDCl3 /opt/DATA nmrafd 1
{7.26,7.26}CDCl3
f2 (ppm)
f1 (ppm)

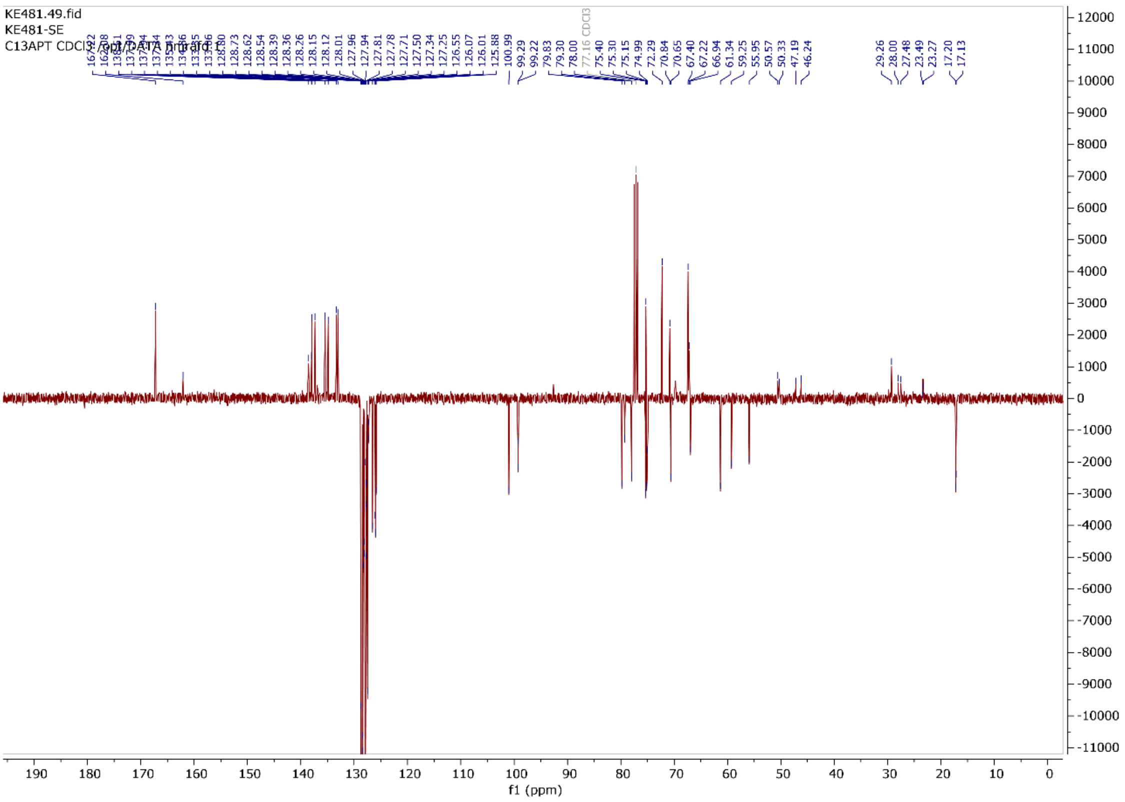
KE481.49.fid
KE481-SE
C13APT CDCl3 /opt/DATA nmrafd 1
128.80
128.73
128.62
128.54
128.39
128.36
128.26
128.15
128.12
128.01
127.96
127.94
127.81
127.78
127.71
127.50
127.34
127.25
126.55
126.07
126.01
125.88
100.99
99.29
99.22
79.83
79.30
78.00
77.16 CDCl3
75.40
75.30
75.15
74.99
72.29
70.84
70.65
67.40
67.22
66.94
61.34
59.25
55.95
50.57
50.33
47.19
46.24
29.26
28.00
27.48
23.49
23.27
17.20
17.13
f1 (ppm)

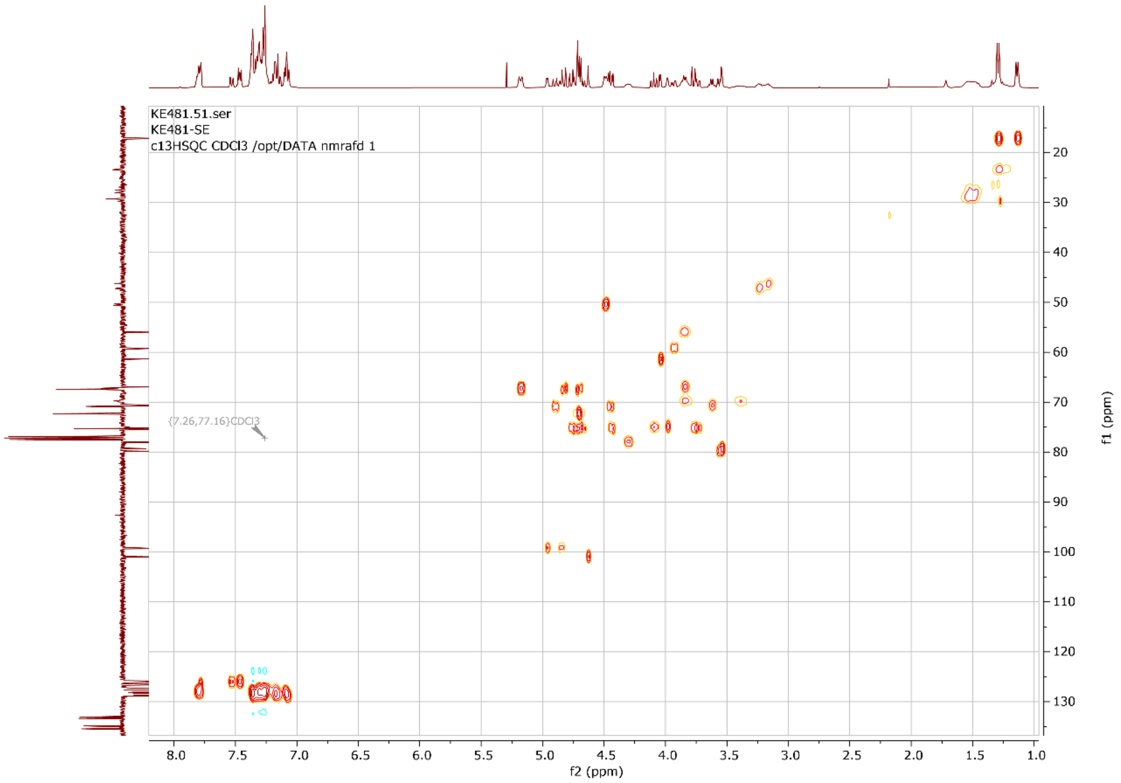
KE481.51.ser
KE481-SE
c13HSQC CDCl3 /opt/DATA nmrafd 1
{7.26,77.16}CDCl3
f2 (ppm)
f1 (ppm)

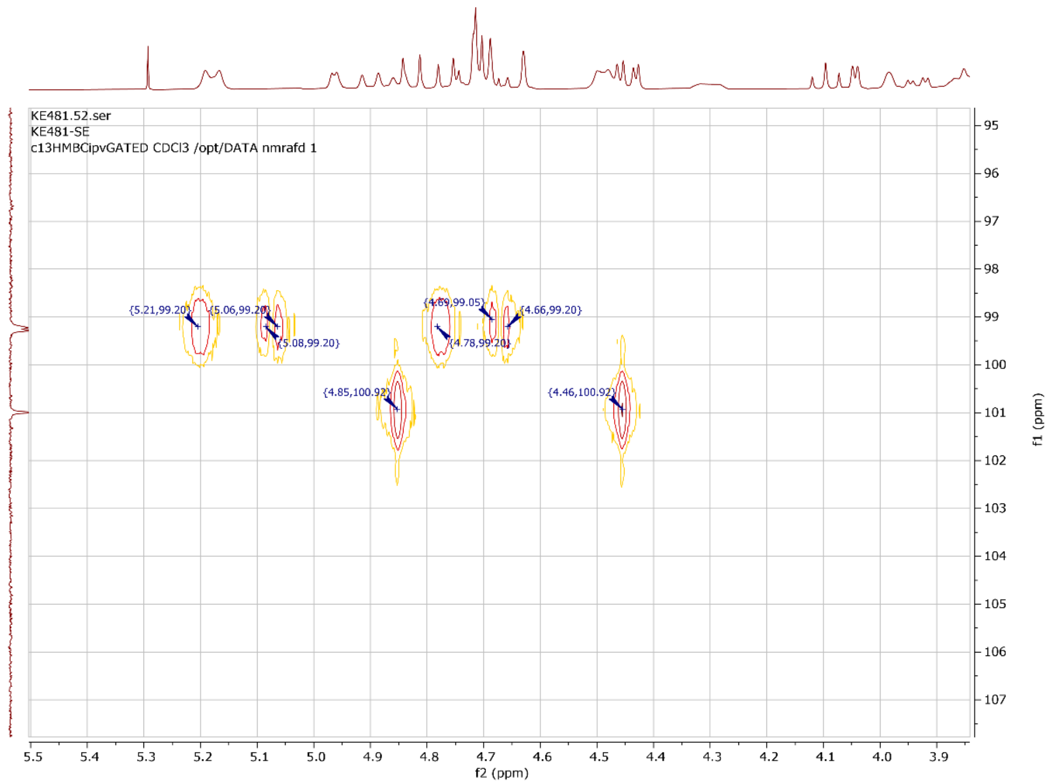
KE481.52.ser
KE481-SE
c13HMBCipvGATED CDCl3 /opt/DATA nmrafd 1
{5.21,99.20}
{5.06,99.20}
{5.08,99.20}
{4.69,99.05}
{4.66,99.20}
{4.78,99.20}
{4.85,100.92}
{4.46,100.92}
f2 (ppm)
f1 (ppm)

5-(Benzyl(benzyloxycarbonyl)amino)pentyl (Benzyl (2-azido-3,4-di-*O*-benzyl-2-deoxy-β-D-mannopyranosiduronsyl)-(1→4)-3-*O*-acetyl-2-azido-2-deoxy-α-L-fucopyranosyl-(1→3)- 4-*O*-benzyl-2-deoxy-2-*N*-trichloroacetamide-α-D-fucopyranoside (23)

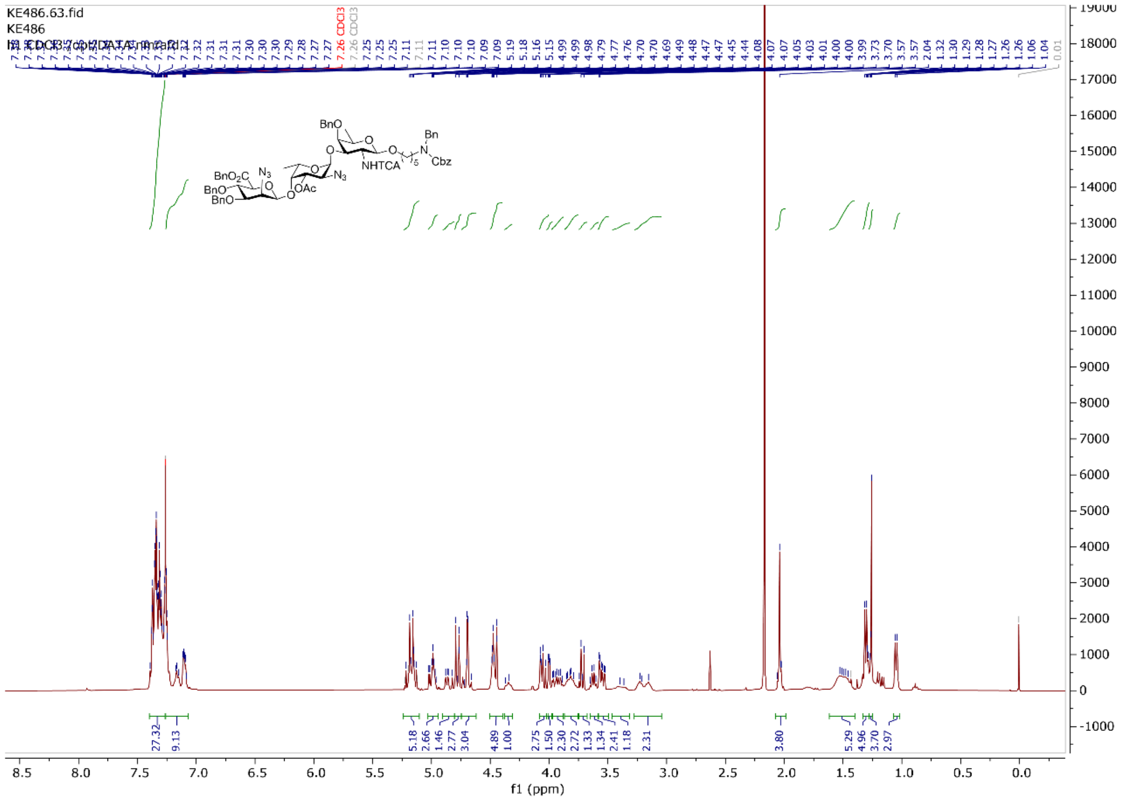

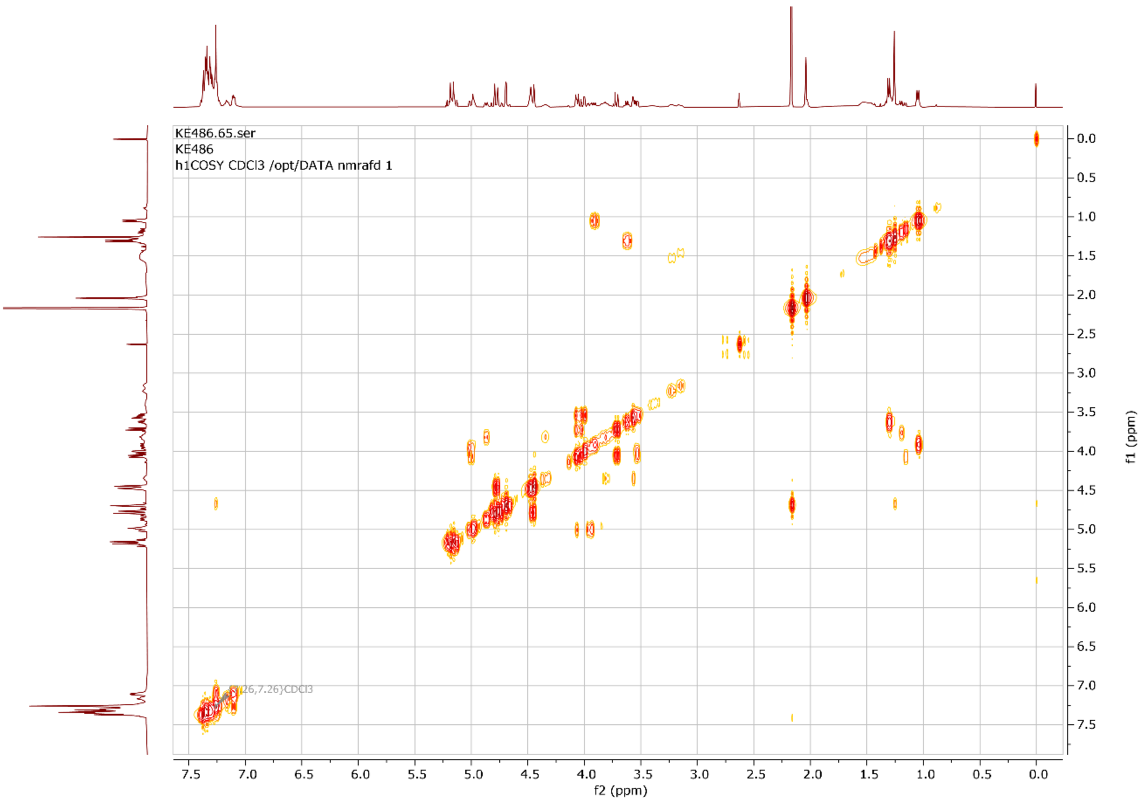
KE486.65.ser
KE486
h1COSY CDCl3 /opt/DATA nmrafd 1
f2 (ppm)
f1 (ppm)

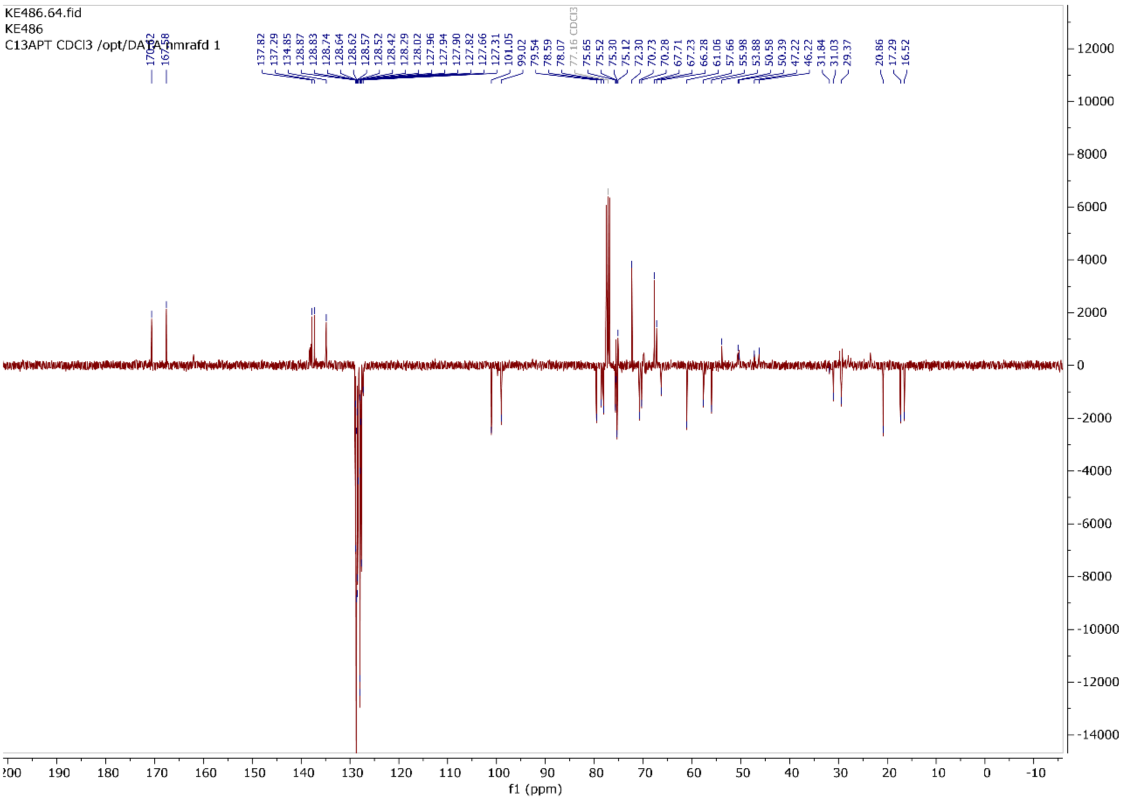
KE486.64.fid
KE486
C13APT CDCl3 /opt/DATA nmrafd 1
137.82
137.29
134.85
128.87
128.83
128.74
128.64
128.62
128.57
128.52
128.42
128.29
128.02
127.96
127.94
127.90
127.82
127.66
127.31
101.05
99.02
79.54
78.59
78.07
77.16 CDCl3
75.65
75.52
75.30
75.12
72.30
70.73
70.28
67.71
67.23
66.28
61.06
57.66
55.98
53.88
50.58
50.39
47.22
46.22
31.84
31.03
29.37
20.86
17.29
16.52
f1 (ppm)

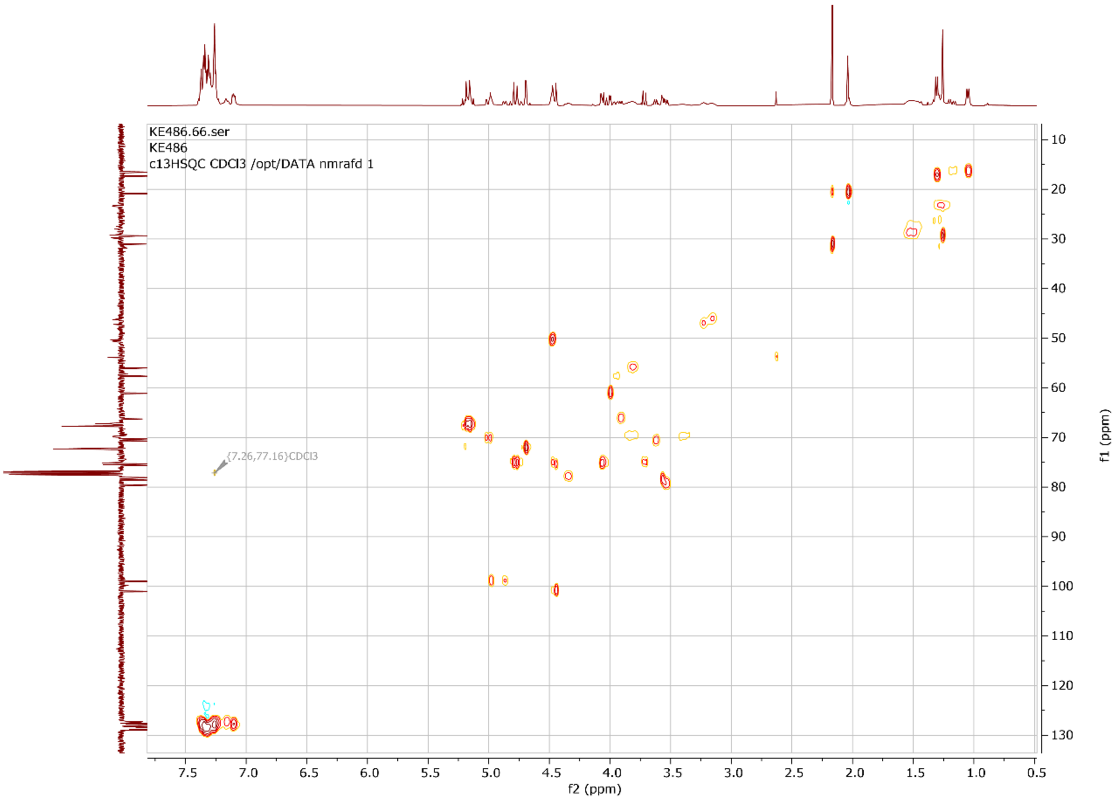

5-aminopentyl 2-*N*-acetamide-2-deoxy-β-D-mannopyranosiduronsyl-(1→4)-2-*N*-acetamide-3-*O*-acetyl-2-deoxy-α-L-fucopyranosyl-(1→3)-2-*N*-acetamide-2-deoxy-β-D-fucopyranoside (1)

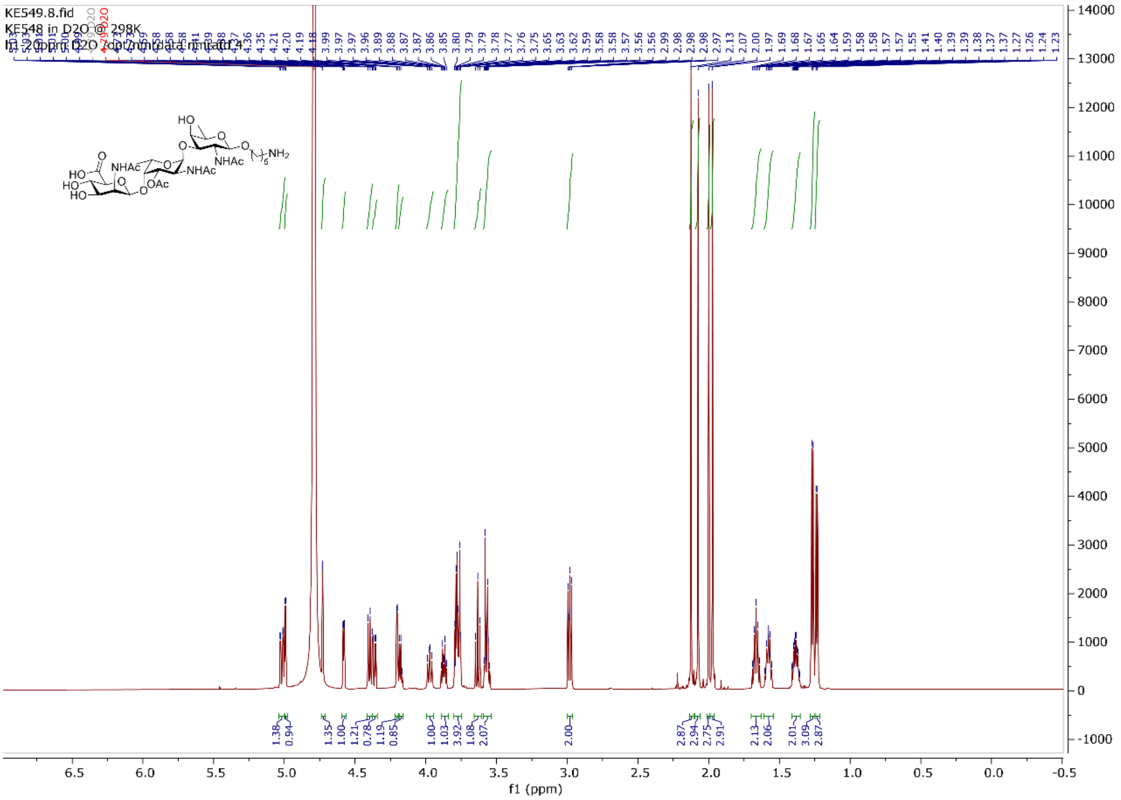
KE549.8.fid
KE548 in D2O @ 298K
h1-20ppm D2O /opt/nmrdata nmrafd 4
f1 (ppm)

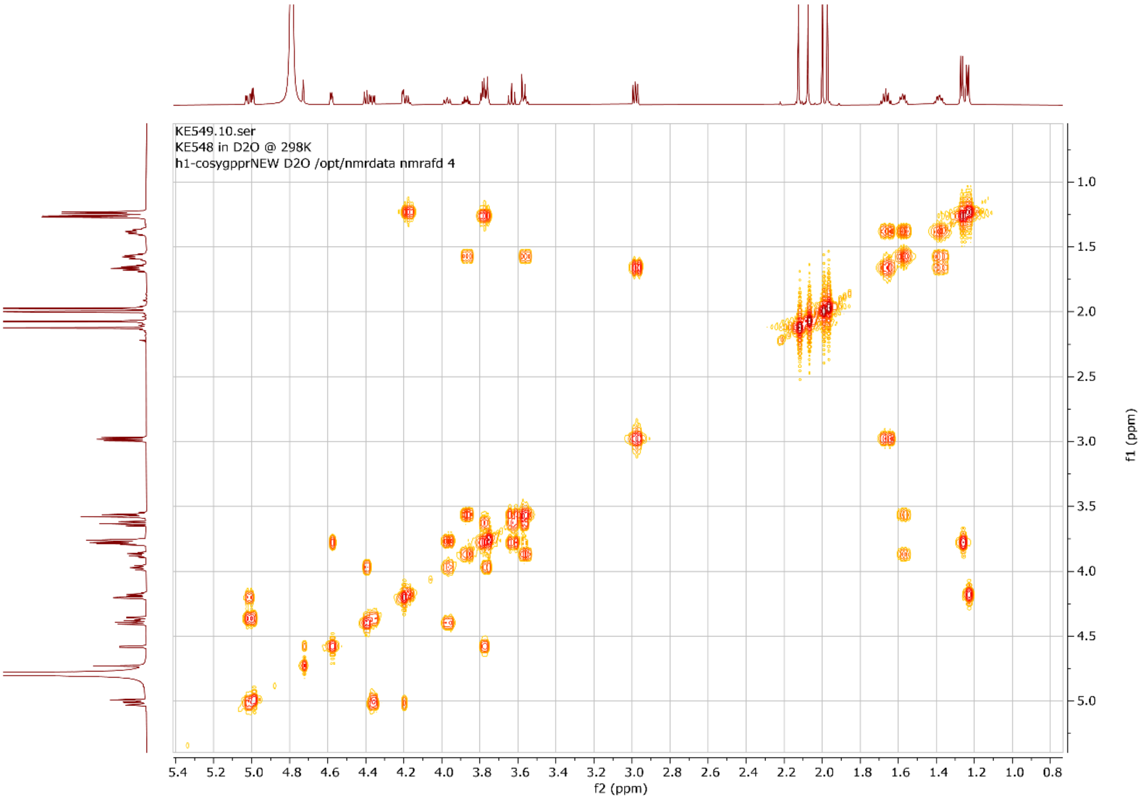
KE549.10.ser
KE548 in D2O @ 298K
h1-cosygpprNEW D2O /opt/nmrdata nmrafd 4
f2 (ppm)
f1 (ppm)

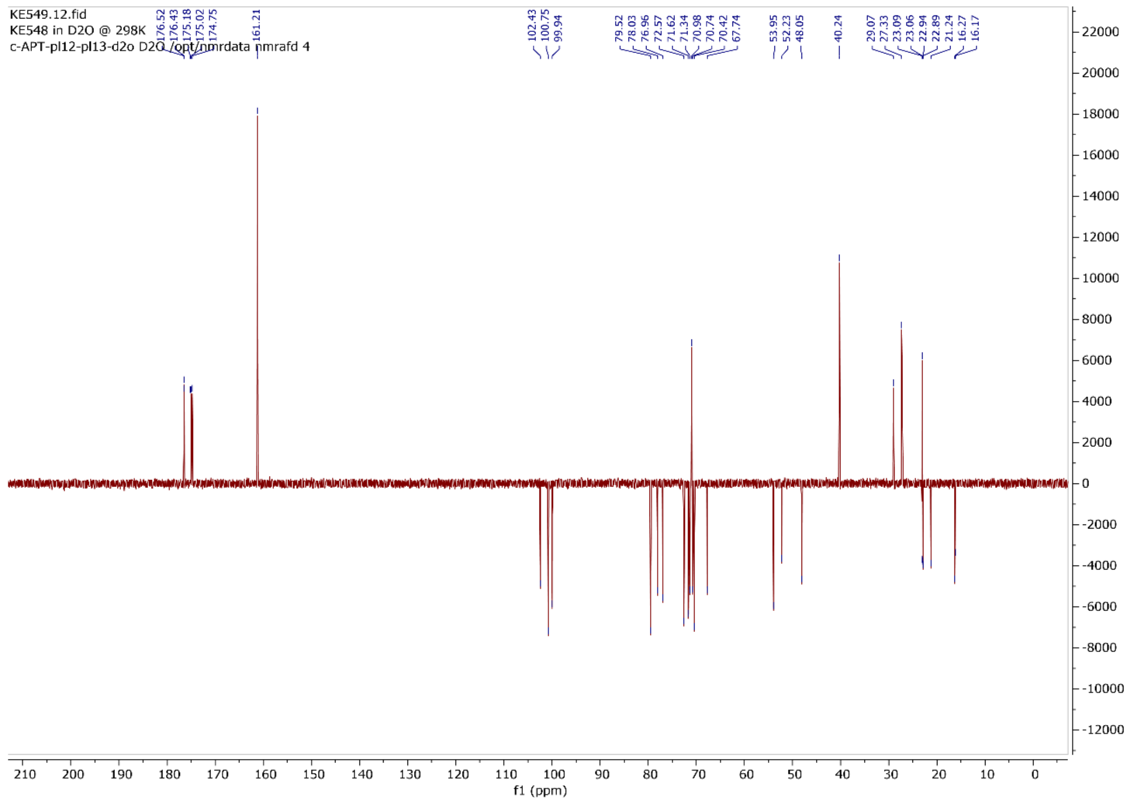
KE549.12.fid
KE548 in D2O @ 298K
c-APT-pl12-pl13-d2o D2O /opt/nmrdata nmrafd 4
176.52
176.43
175.18
175.02
174.75
161.21
102.43
100.75
99.94
79.52
78.03
76.96
72.57
71.62
71.34
70.98
70.74
70.42
67.74
53.95
52.23
48.05
40.24
29.07
27.33
23.09
23.06
22.94
22.89
21.24
16.27
16.17
f1 (ppm)

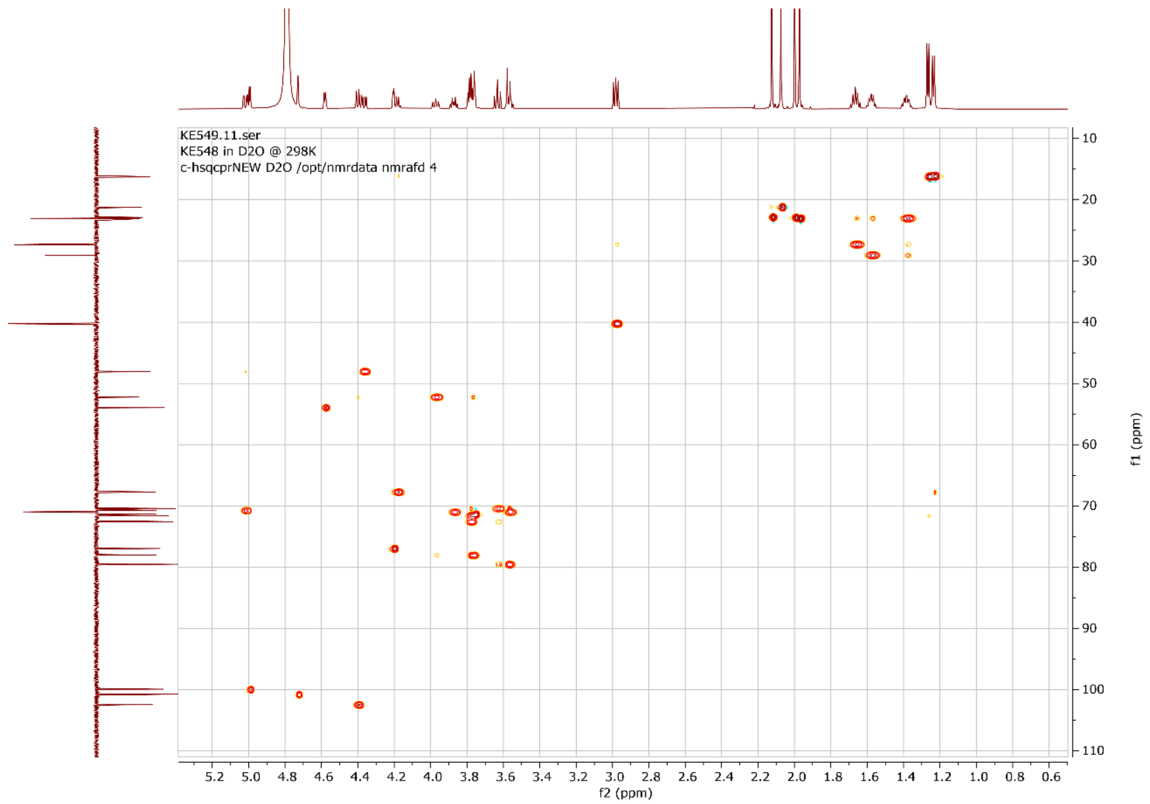
KE549.11.ser
KE548 in D2O @ 298K
c-hsqcprNEW D2O /opt/nmrdata nmrafd 4
f2 (ppm)
f1 (ppm)

***Tert*-butyldiphenylsilyl (Benzyl (2-azido-2-deoxy-3-*O*-benzyl-4-*O*-*p*-methoxybenzyl-β-D-mannopyranosiduronsyl)-(1→4)-2-azido-2-deoxy-3-*O*-(2-naphthylmethyl)-α-L-fucopyranosyl-(1→3)-4-*O*-benzyl-2-deoxy-2-*N*-trichloroacetamide-β-D-fucopyranoside (6)**

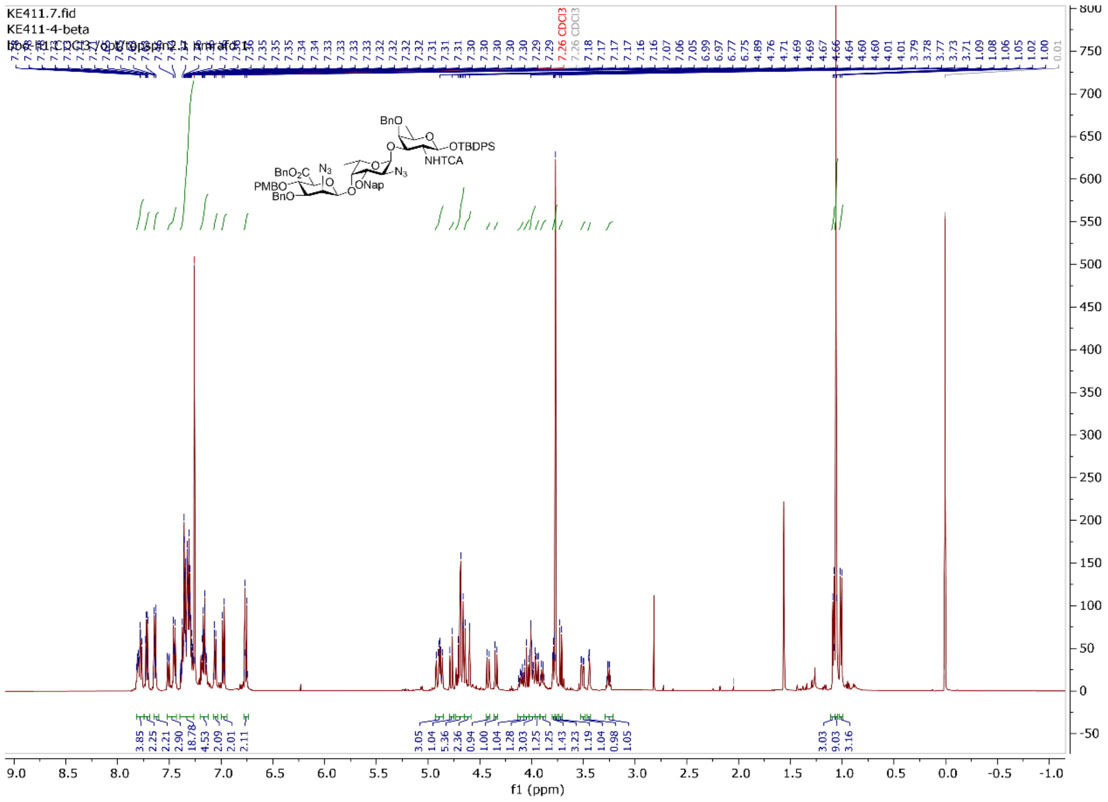

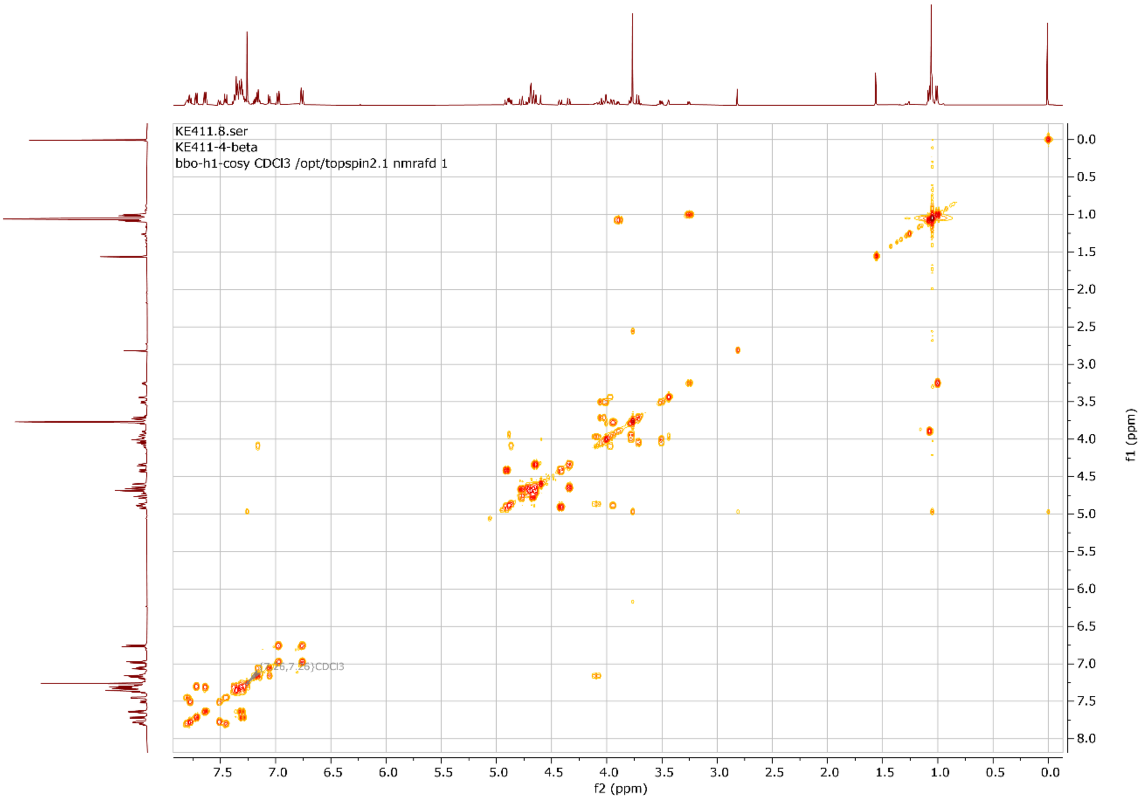
KE411.8.ser
KE411-4-beta
bbo-h1-cosy CDCl3 /opt/topspin2.1 nmrafd 1
f2 (ppm)
f1 (ppm)

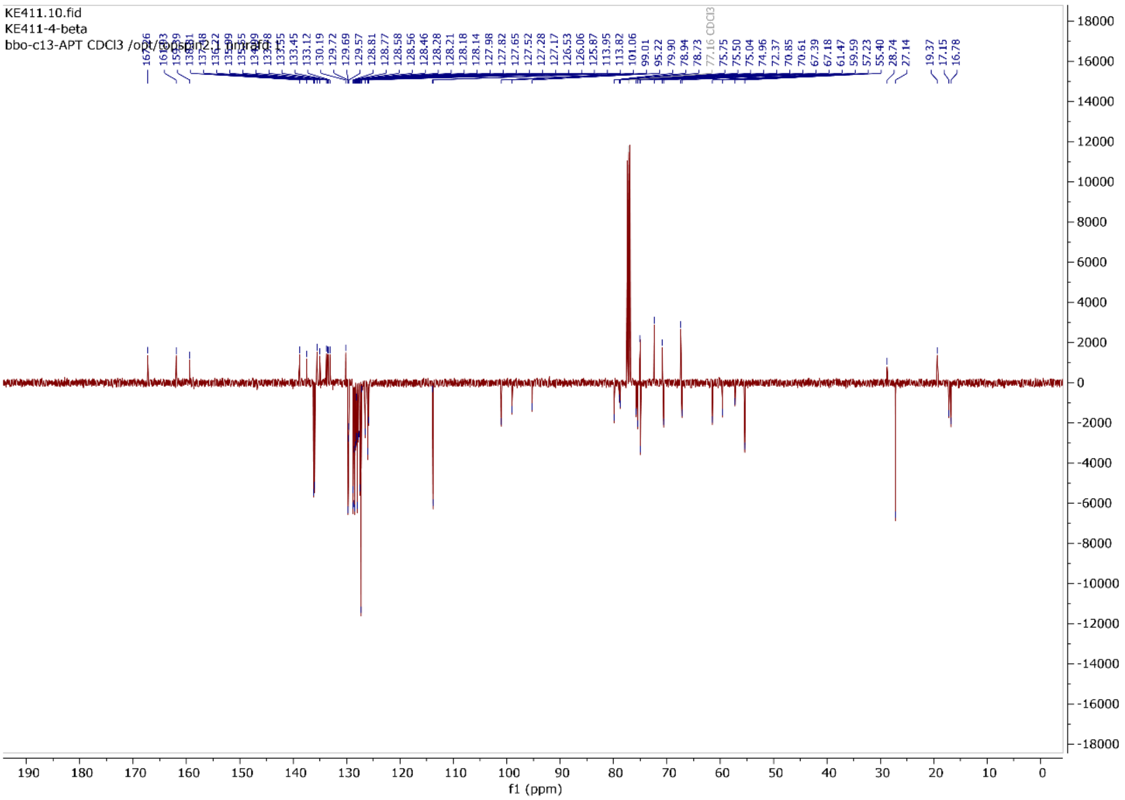
KE411.10.fid
KE411-4-beta
bbo-c13-APT CDCl3 /opt/topspin2.1 nmrafd 1
77.16 CDCl3
f1 (ppm)

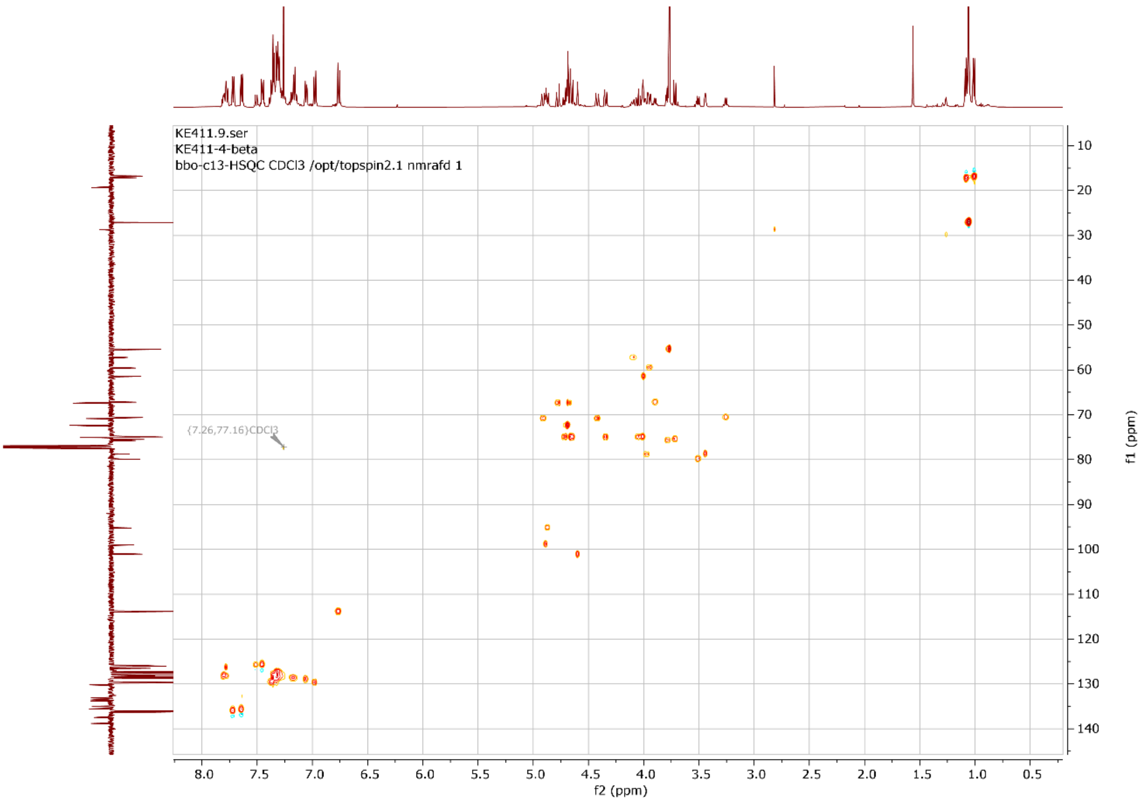
KE411.9.ser
KE411-4-beta
bbo-c13-HSQC CDCl3 /opt/topspin2.1 nmrafd 1
{7.26,77.16}CDCl3
f2 (ppm)
f1 (ppm)

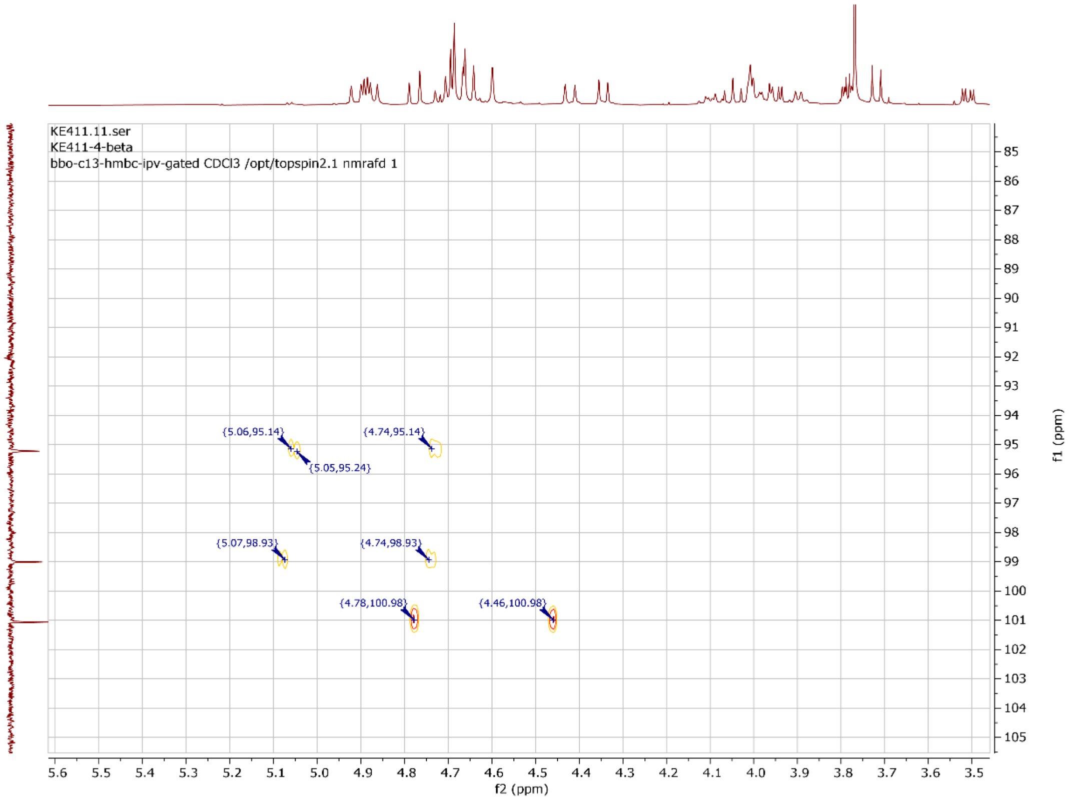
KE411.11.ser
KE411-4-beta
bbo-c13-hmbc-ipv-gated CDCl3 /opt/topspin2.1 nmrafd 1
{5.06,95.14}
{5.05,95.24}
{4.74,95.14}
{5.07,98.93}
{4.74,98.93}
{4.78,100.98}
{4.46,100.98}
f2 (ppm)
f1 (ppm)

Tert-butyldiphenylsilyl (Benzyl (2-azido-2-deoxy-3-_O_-benzyl-4-_O_-_p_-methoxybenzyl-β-D-mannopyranosiduronsyl)-(1→4)-2-azido-2-deoxy-3-_O_-(2-naphthylmethyl)-α-L-fucopyranosyl-(1→3)-4-_O_-benzyl-2-deoxy-2-_N_-triflouroacetamide-β-D-fucopyranoside (8)

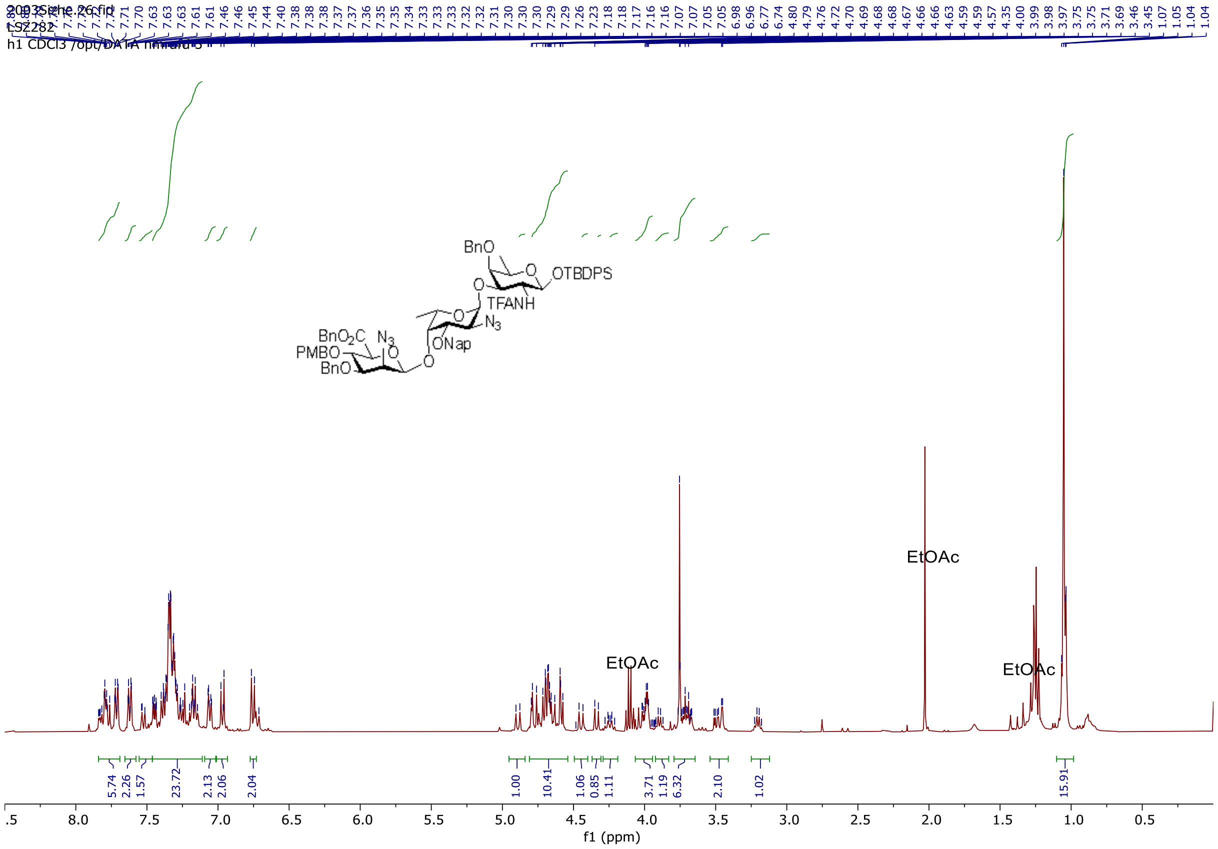

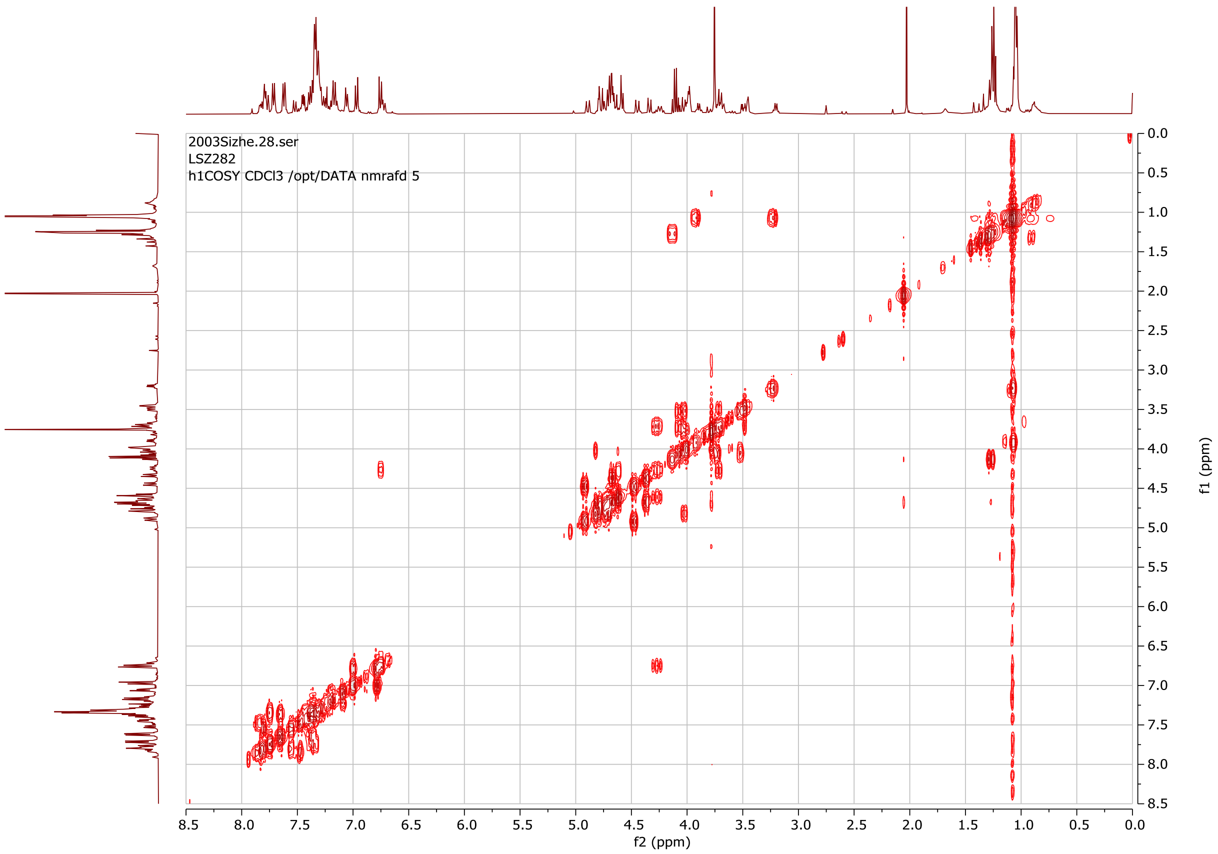

2003Sizhe.28.ser
LSZ282
h1COSY CDCl3 /opt/DATA nmrafd 5
f2 (ppm)
f1 (ppm)

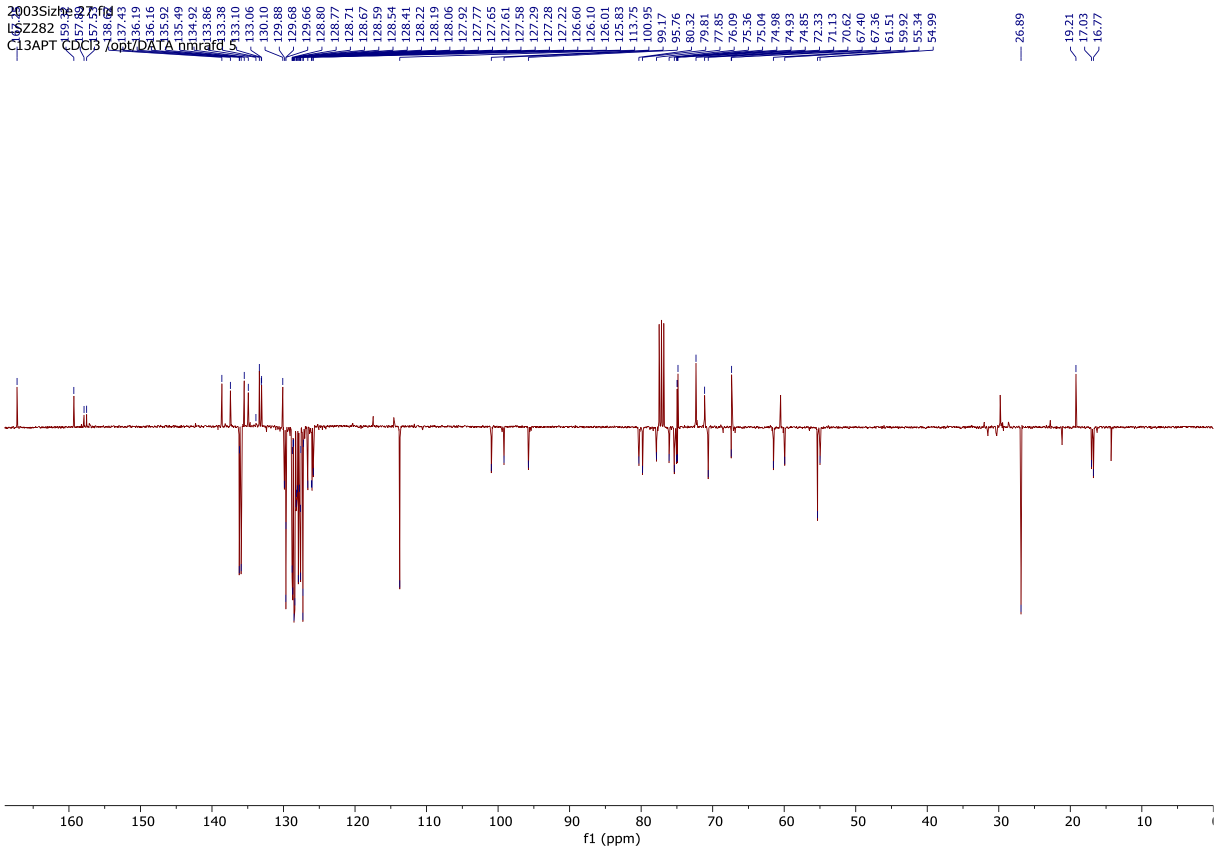

2003Sizhe.27.fid
LSZ282
C13APT CDCl3 /opt/DATA nmrafd 5
137.43
136.19
136.16
135.92
135.49
134.92
133.86
133.38
133.10
133.06
130.10
129.88
129.68
129.66
128.80
128.77
128.71
128.67
128.59
128.54
128.41
128.22
128.19
128.06
127.92
127.77
127.65
127.61
127.58
127.29
127.28
127.22
126.60
126.10
126.01
125.83
113.75
100.95
99.17
95.76
80.32
79.81
77.85
76.09
75.36
75.04
74.98
74.93
74.85
72.33
71.13
70.62
67.40
67.36
61.51
59.92
55.34
54.99
26.89
19.21
17.03
16.77
f1 (ppm)

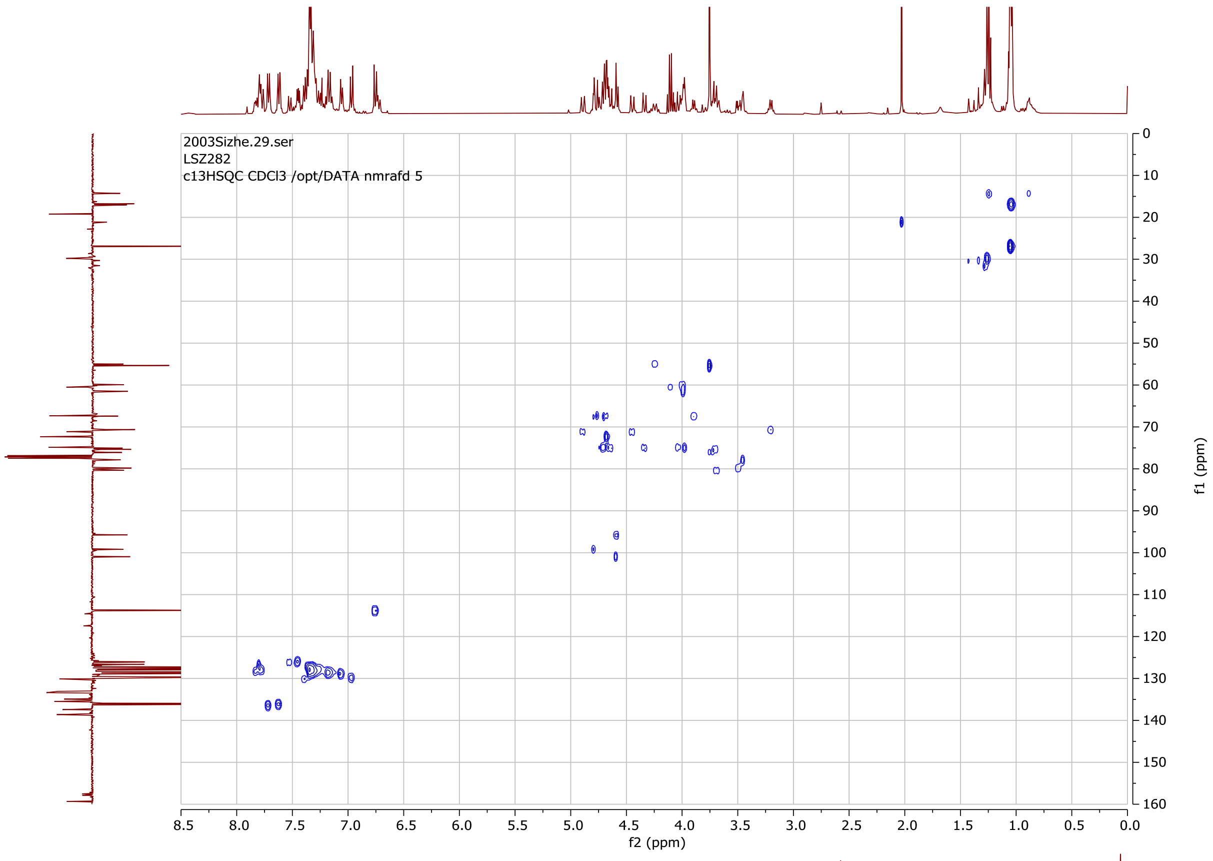
2003Sizhe.29.ser
LSZ282
c13HSQC CDCl3 /opt/DATA nmrafd 5
f2 (ppm)
f1 (ppm)

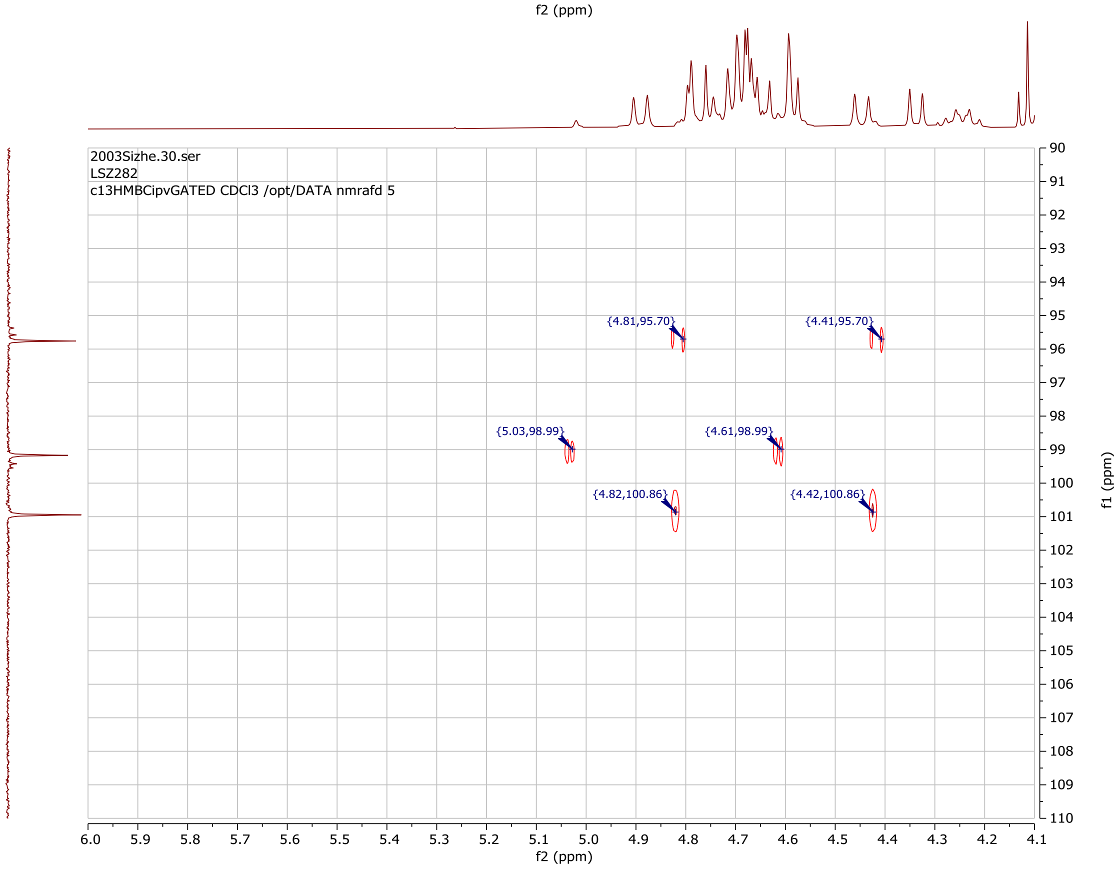
2003Sizhe.30.ser
LSZ282
c13HMBCipvGATED CDCl3 /opt/DATA nmrafd 5
{4.81,95.70}
{4.41,95.70}
{5.03,98.99}
{4.61,98.99}
{4.82,100.86}
{4.42,100.86}
f2 (ppm)
f1 (ppm)

α-anomer

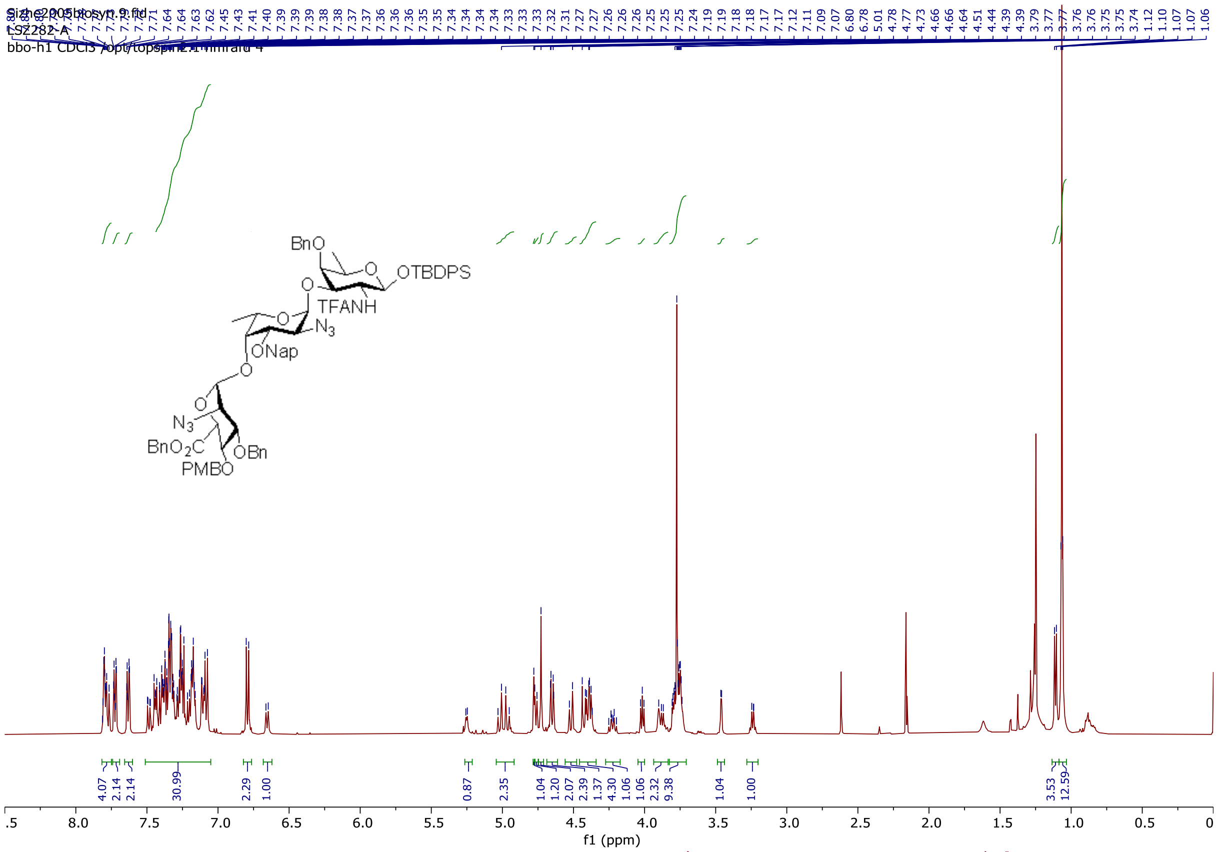
LSZ282-A
bbo-h1 CDCl3 /opt/topspin2.1 nmrafd 4
BnO
OTBDPS
TFANH
N3
ONap
BnO2C
OBn
PMBO
f1 (ppm)

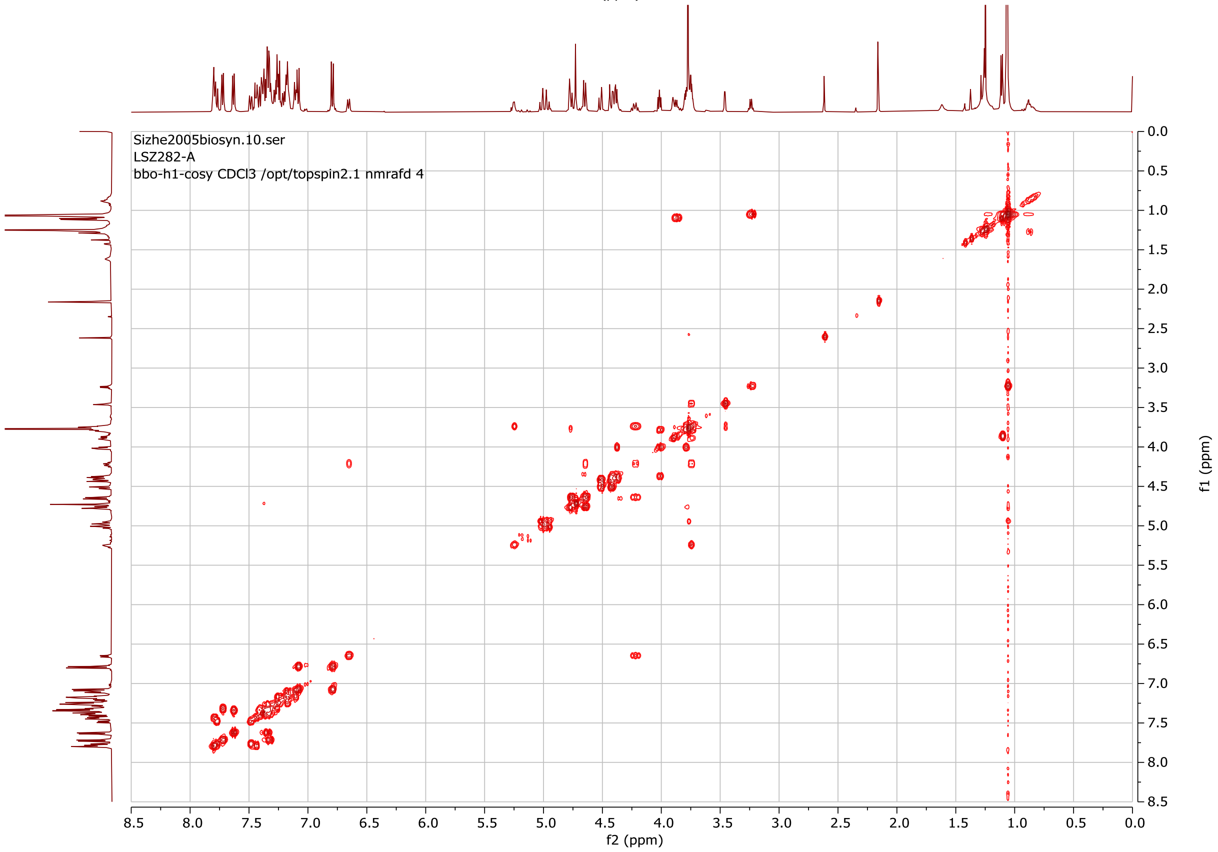
Sizhe2005biosyn.10.ser
LSZ282-A
bbo-h1-cosy CDCl3 /opt/topspin2.1 nmrafd 4
f2 (ppm)
f1 (ppm)

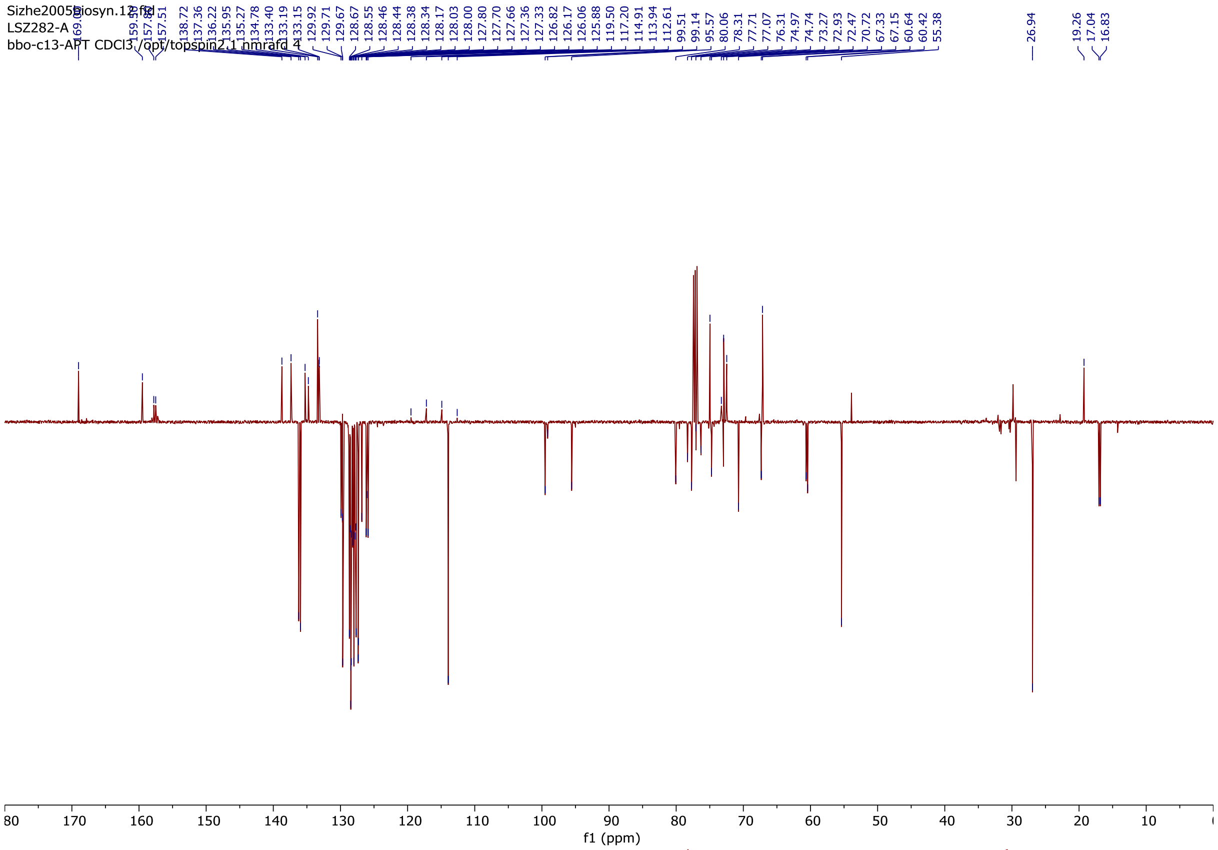
Sizhe2005biosyn.12.fid
LSZ282-A
bbo-c13-APT CDCl3 /opt/topspin2.1 nmrafd 4
169.00
159.50
157.80
157.51
138.72
137.36
136.22
135.95
135.27
134.78
133.40
133.19
133.15
129.92
129.71
129.67
128.67
128.55
128.46
128.44
128.38
128.34
128.17
128.03
128.00
127.80
127.70
127.66
127.36
127.33
126.82
126.17
126.06
125.88
119.50
117.20
114.91
113.94
112.61
99.51
99.14
95.57
80.06
78.31
77.71
77.07
76.31
74.97
74.74
73.27
72.93
72.47
70.72
67.33
67.15
60.64
60.42
55.38
26.94
19.26
17.04
16.83
f1 (ppm)

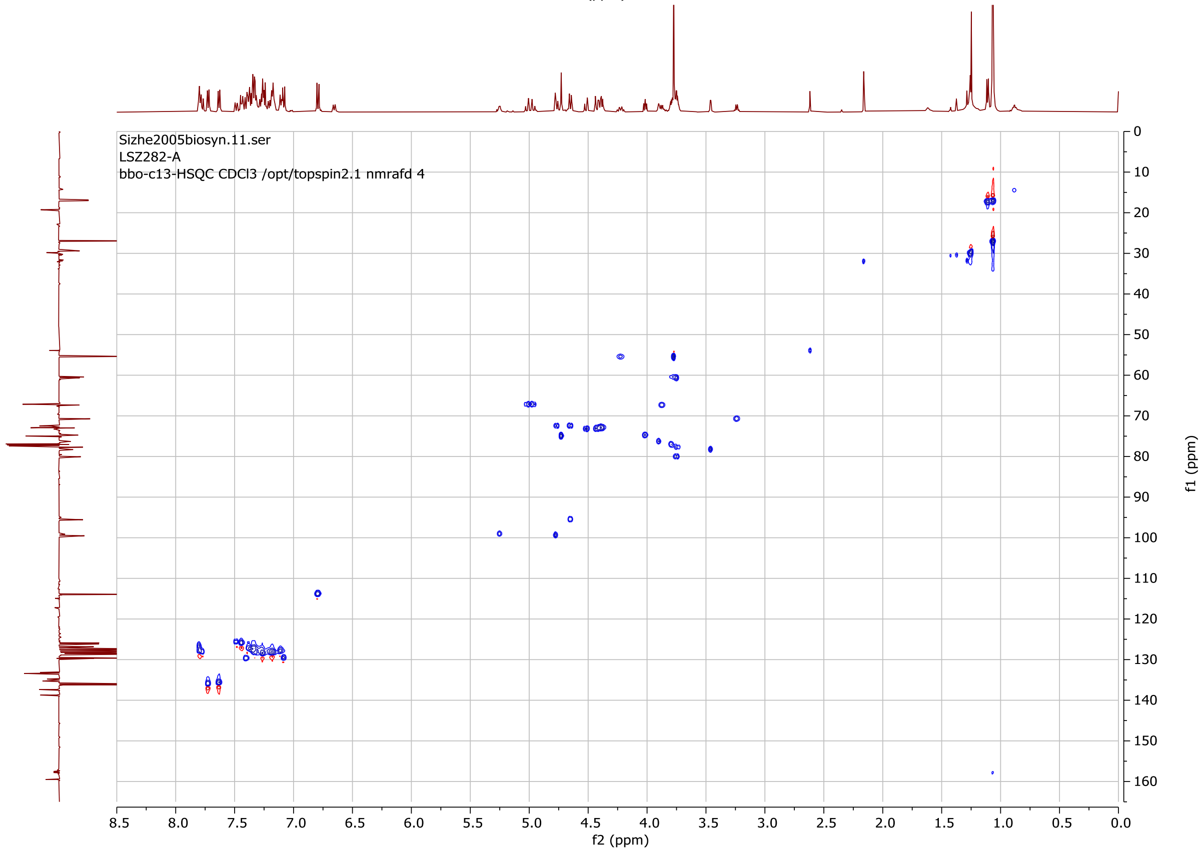
Sizhe2005biosyn.11.ser
LSZ282-A
bbo-c13-HSQC CDCl3 /opt/topspin2.1 nmrafd 4
f2 (ppm)
f1 (ppm)

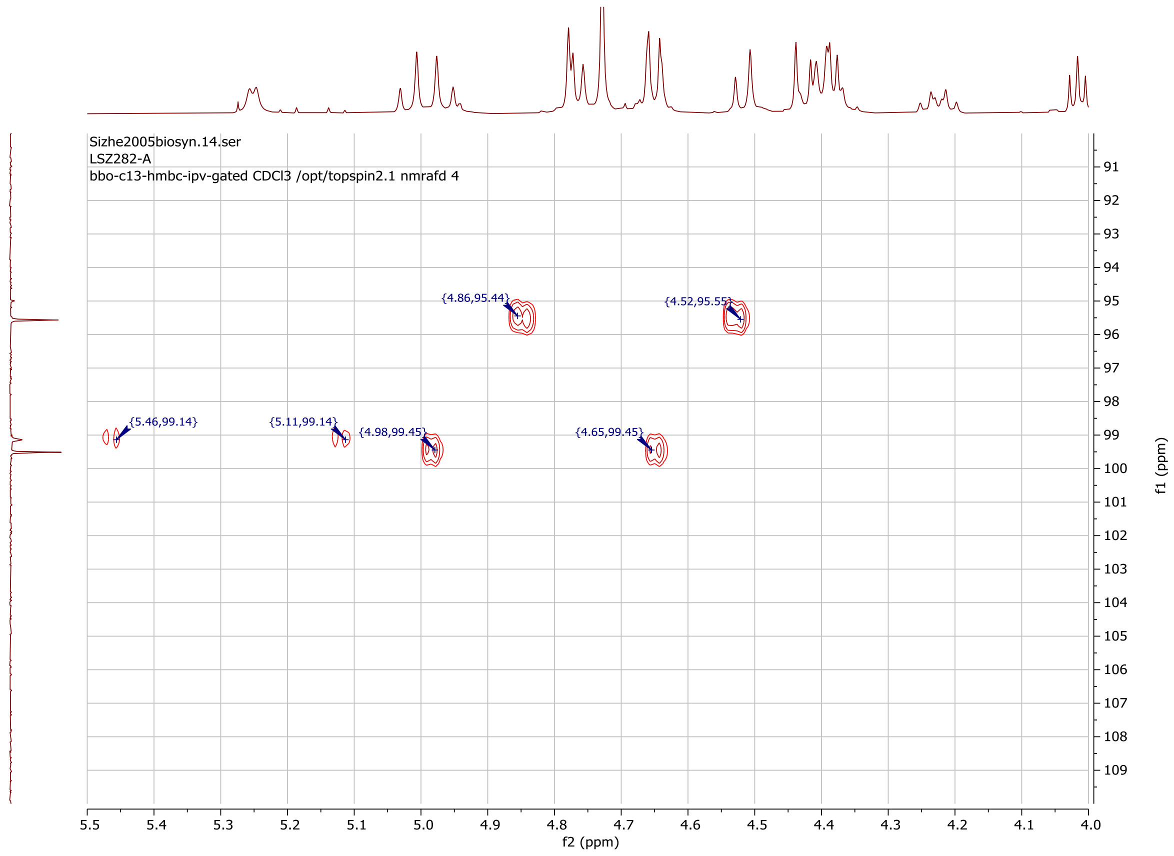

***Tert*-butyldiphenylsilyl (Benzyl (2-azido-3,4-di-*O*-benzyl-2-deoxy-β-D-mannopyranosiduronsyl)-(1→4)-2-azido-2-deoxy-3-*O*-(2-naphthylmethyl)-α-L-fucopyranosyl-(1→3)-4-*O*-benzyl-2-deoxy-2-*N*-trichloroacetamide-β-D-fucopyranoside (9)**

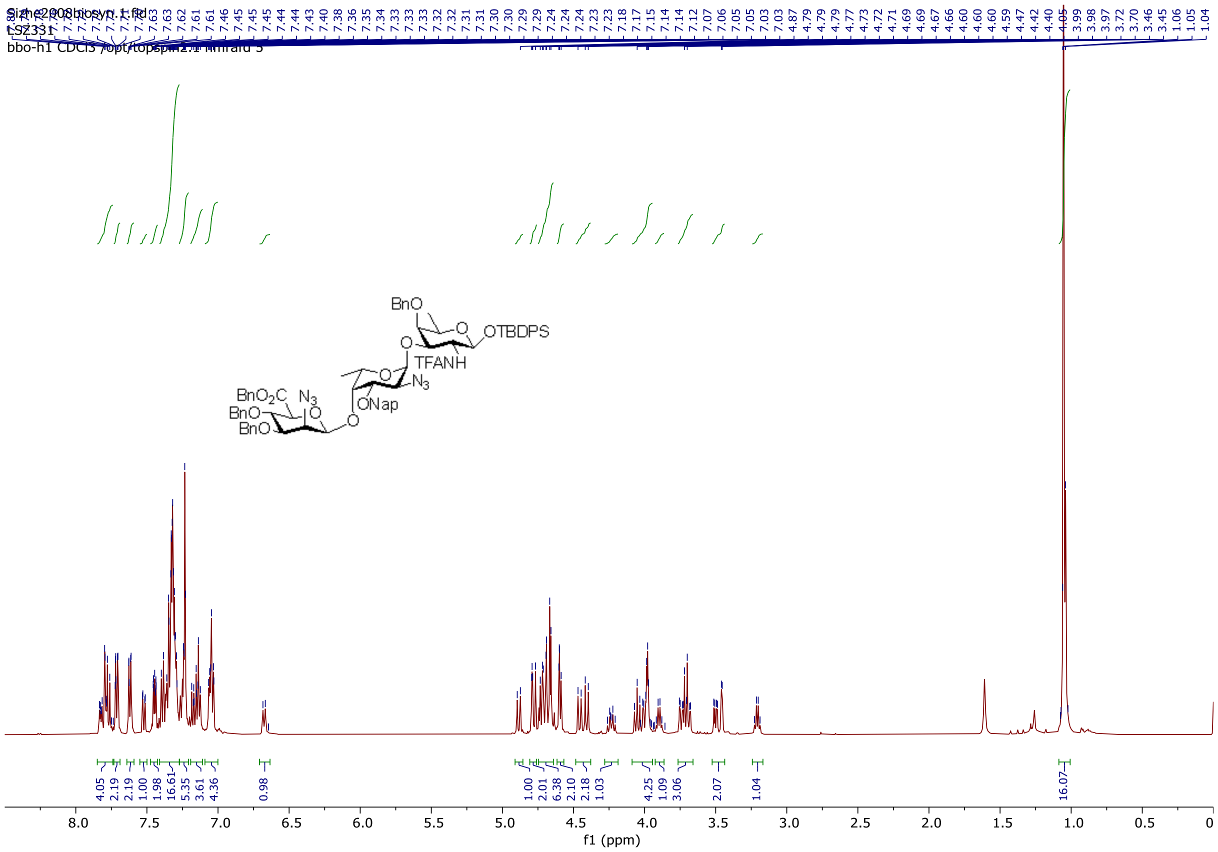
Sizhe2008biosyn.1.fid
LSZ331
bbo-h1 CDCl3 /opt/topspin2.1 nmrafd 5
BnO
OTBDPS
TFANH
N_3
ONap
BnO_2C
BnO
f1 (ppm)

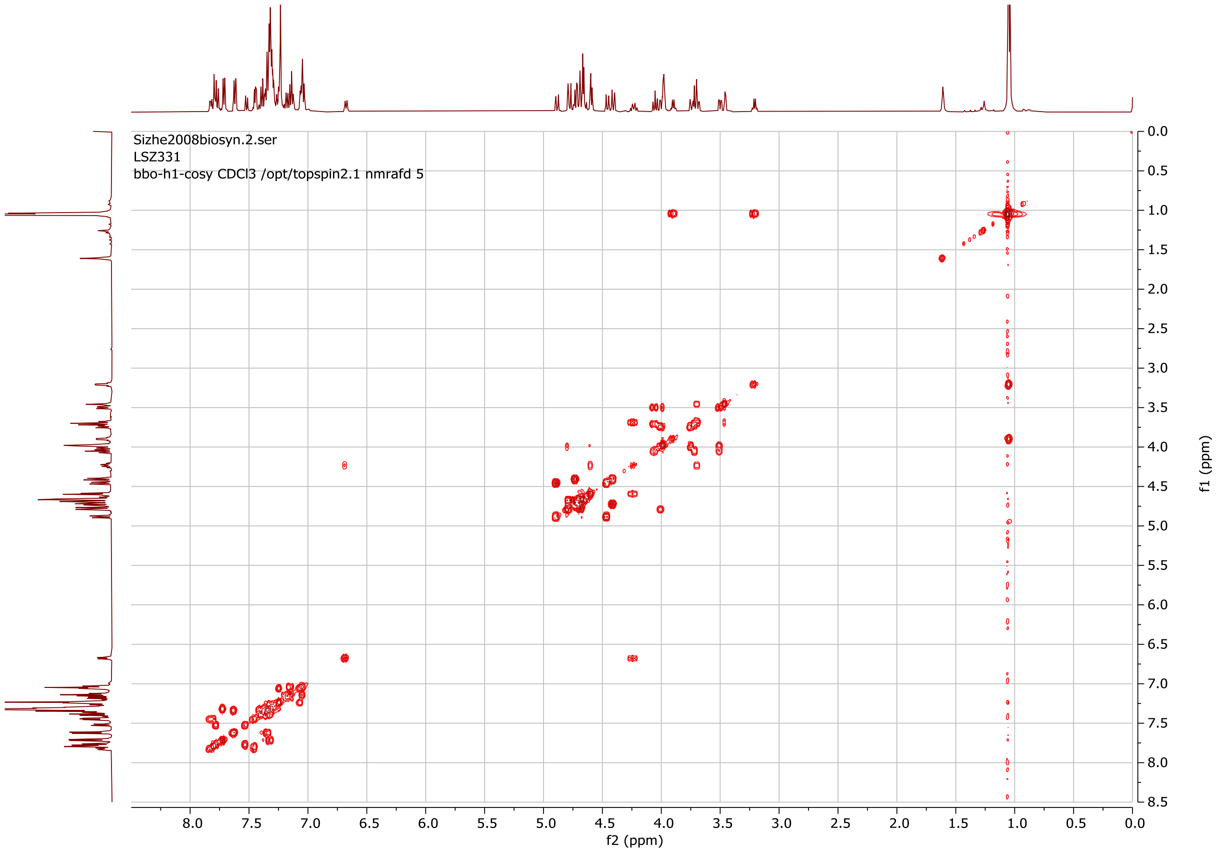
Sizhe2008biosyn.2.ser
LSZ331
bbo-h1-cosy CDCl3 /opt/topspin2.1 nmrafd 5
f2 (ppm)
f1 (ppm)

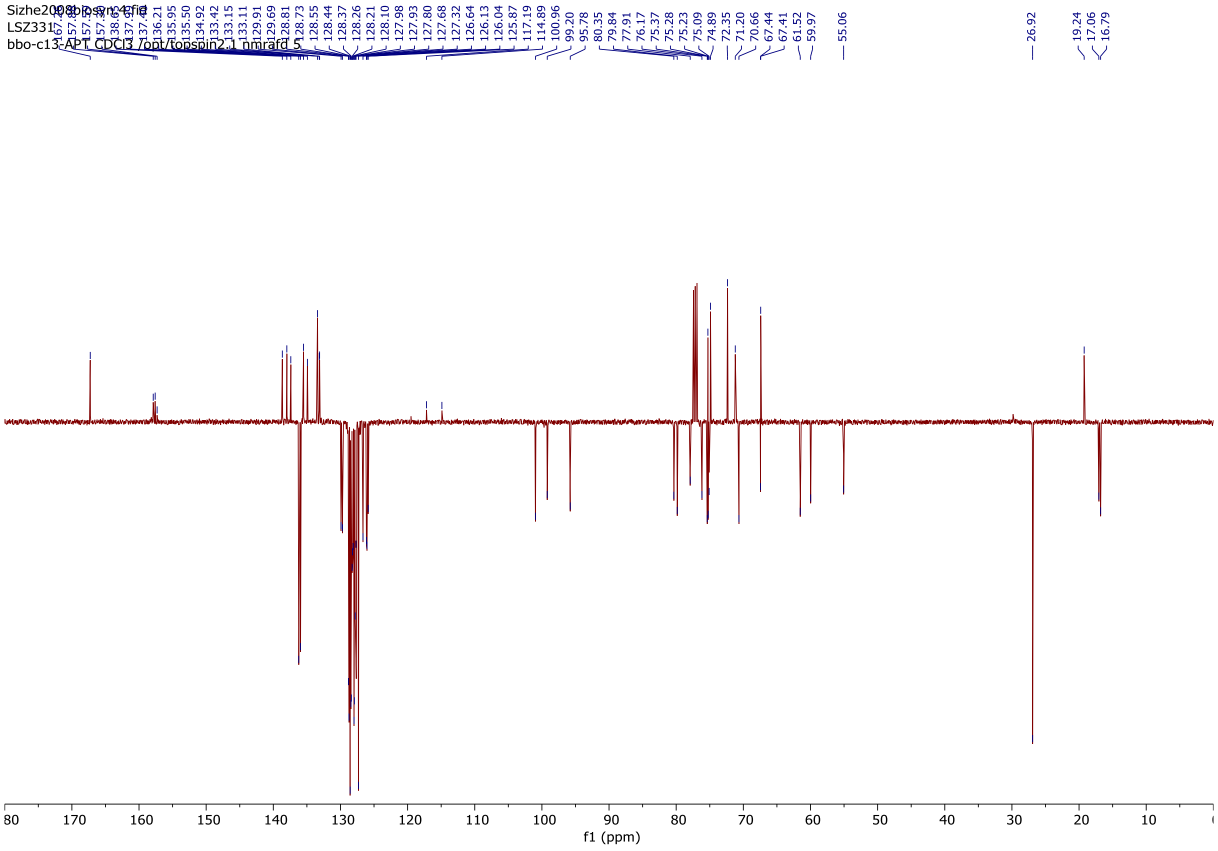
Sizhe2008biosyn.4.fid
LSZ331
bbo-c13-APT CDCl3 /opt/topspin2.1 nmrafd 5
136.21
135.95
135.50
134.92
133.42
133.15
133.11
129.91
129.69
128.81
128.73
128.55
128.44
128.37
128.26
128.21
128.10
127.98
127.93
127.80
127.68
127.32
126.64
126.13
126.04
125.87
117.19
114.89
100.96
99.20
95.78
80.35
79.84
77.91
76.17
75.37
75.28
75.23
75.09
74.89
72.35
71.20
70.66
67.44
67.41
61.52
59.97
55.06
26.92
19.24
17.06
16.79
f1 (ppm)

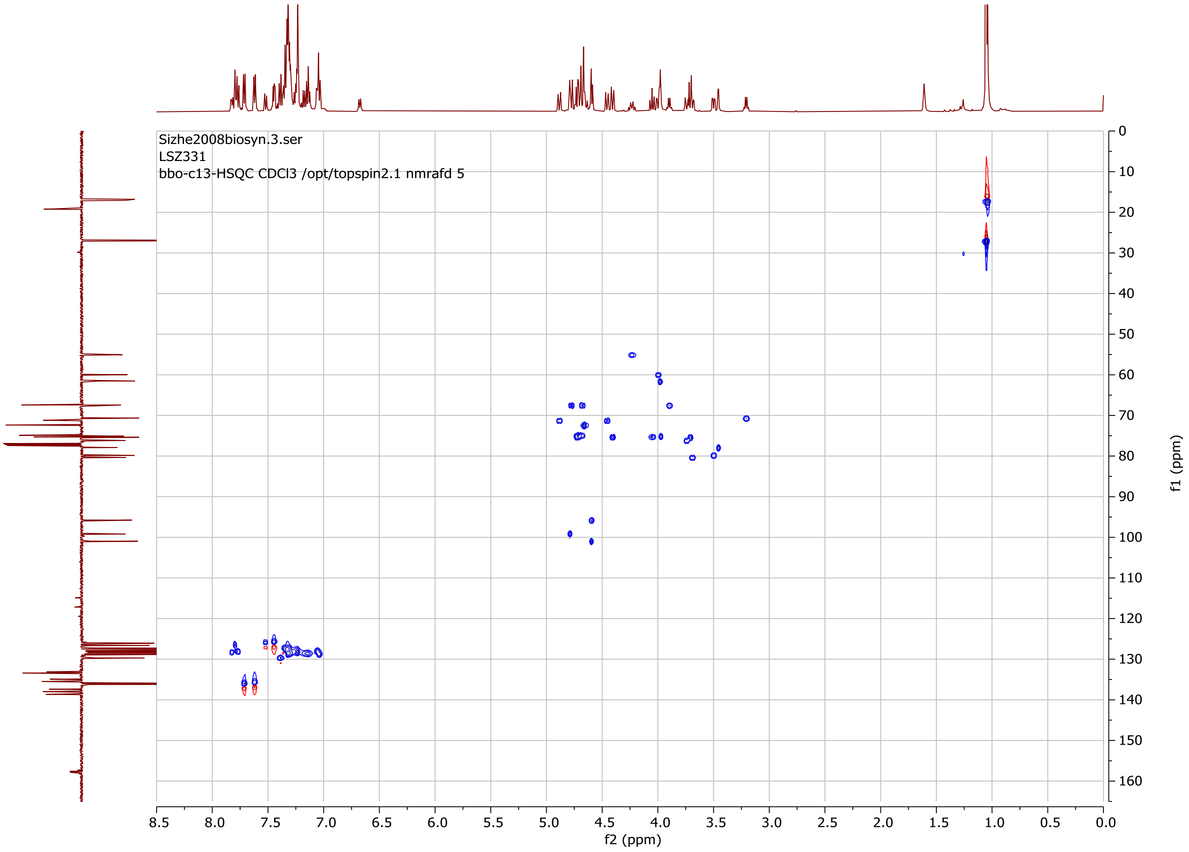
Sizhe2008biosyn.3.ser
LSZ331
bbo-c13-HSQC CDCl3 /opt/topspin2.1 nmrafd 5
f2 (ppm)
f1 (ppm)

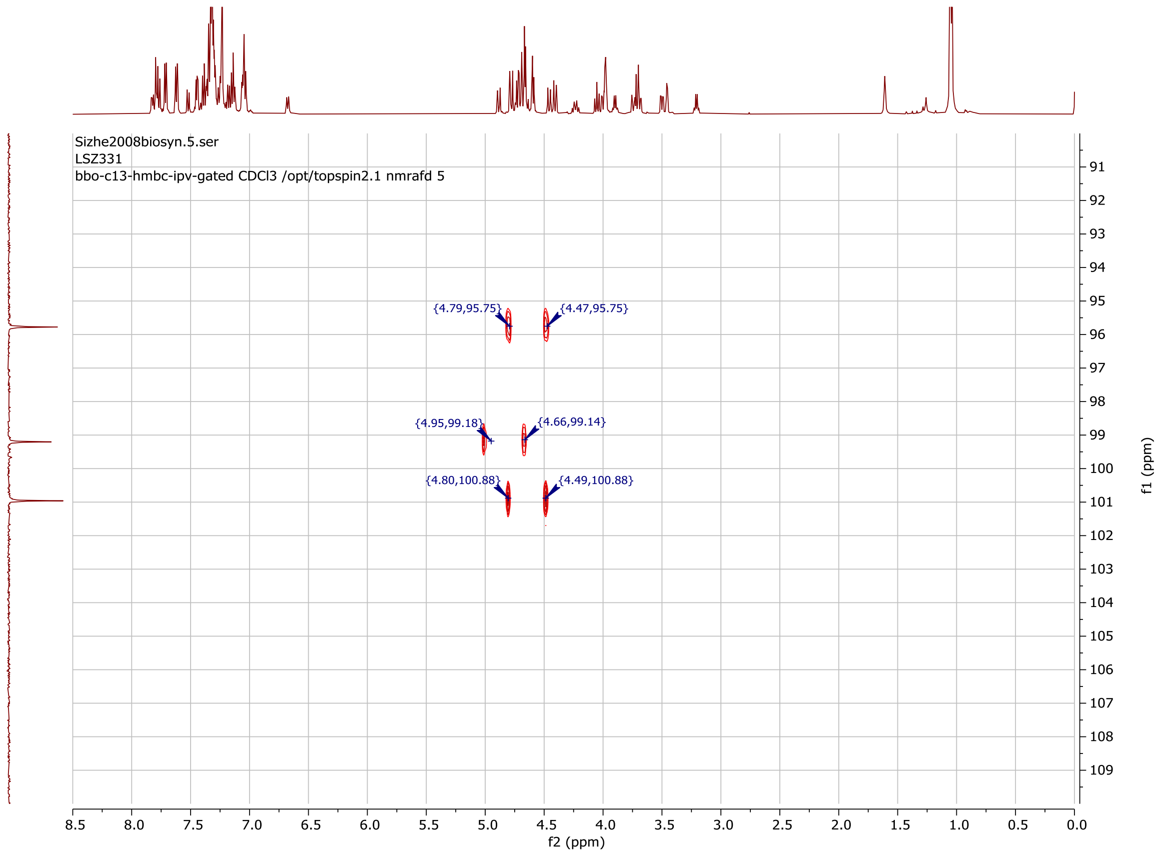

(Benzyl (2-azido-3-*O*-benzyl-2-deoxy-4-*O*-*p*-methoxybenzyl-β-D-mannopyranosiduronsyl)-(1→4)-2-azido-2-deoxy-3-*O*-(2-naphthylmethyl)-α-L-fucopyranosyl-(1→3)-4-*O*-benzyl-2-deoxy-2-*N*-trichloroacetamide-β-D-fucopyranose (25-OH)

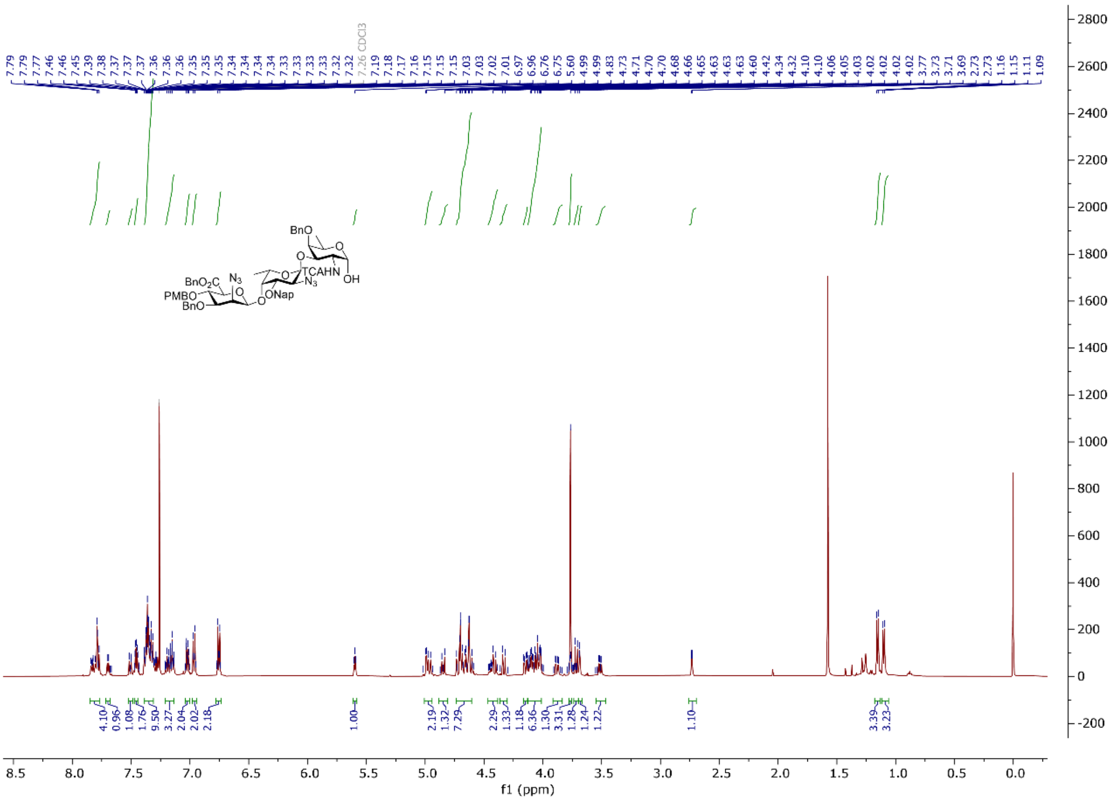
7.26 CDCl3
BnO
TCAHN
OH
BnO2C
N3
PMBO
BnO
ONap
f1 (ppm)

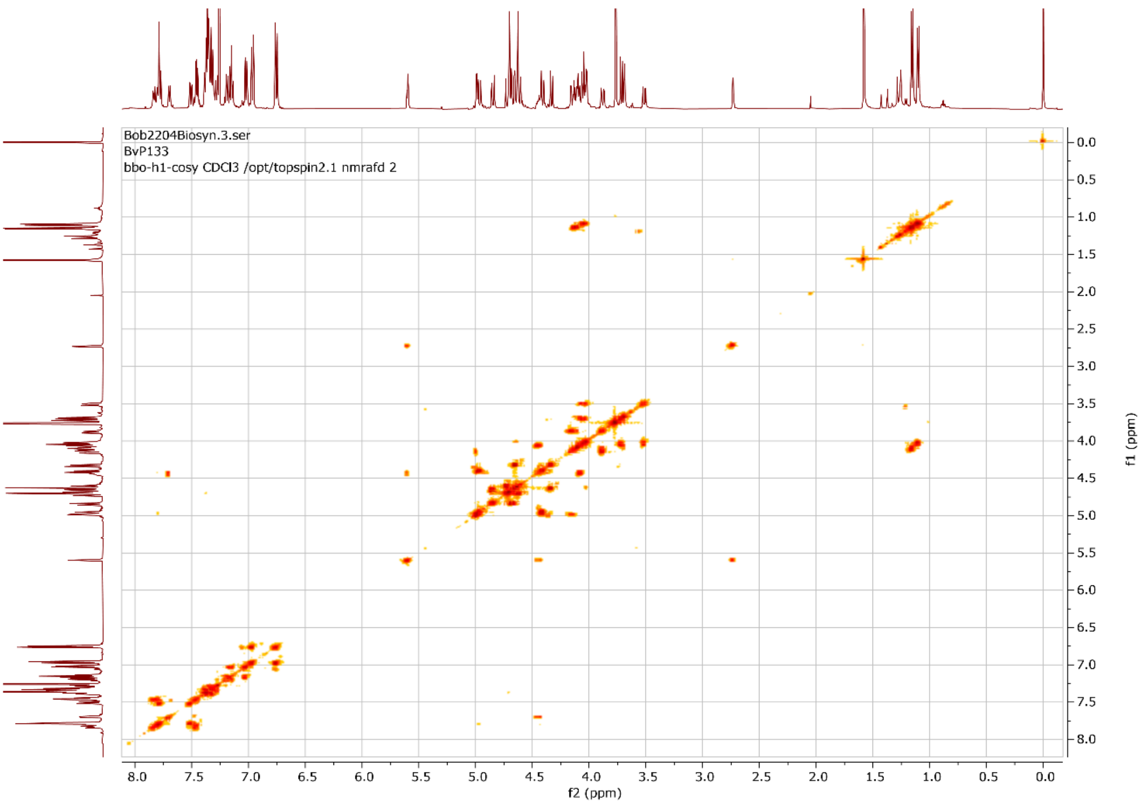
Bob2204Biosyn.3.ser
BvP133
bbo-h1-cosy CDCl3 /opt/topspin2.1 nmrafd 2
f2 (ppm)
f1 (ppm)

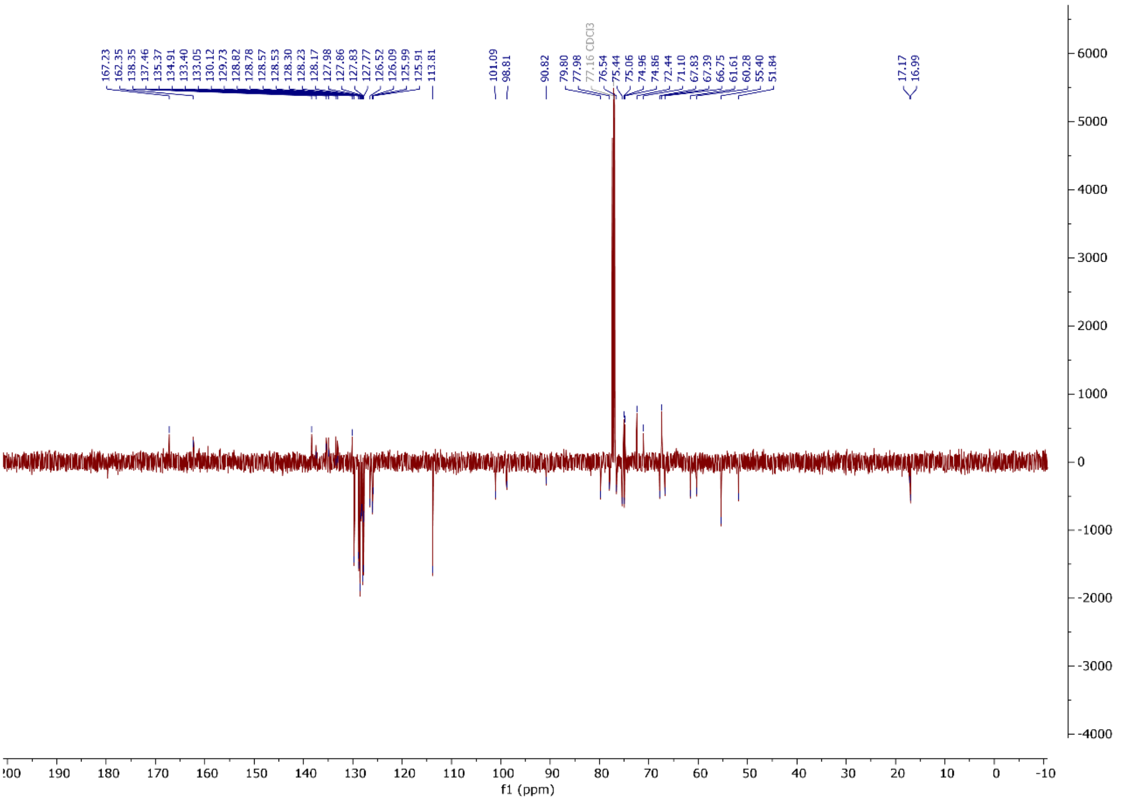
167.23
162.35
138.35
137.46
135.37
134.91
133.40
133.05
130.12
129.73
128.82
128.78
128.57
128.53
128.30
128.23
128.17
127.98
127.86
127.83
127.77
126.52
126.09
125.99
125.91
113.81
101.09
98.81
90.82
79.80
77.98
77.16 CDCl3
76.54
75.44
75.06
74.96
74.86
72.44
71.10
67.83
67.39
66.75
61.61
60.28
55.40
51.84
17.17
16.99
f1 (ppm)

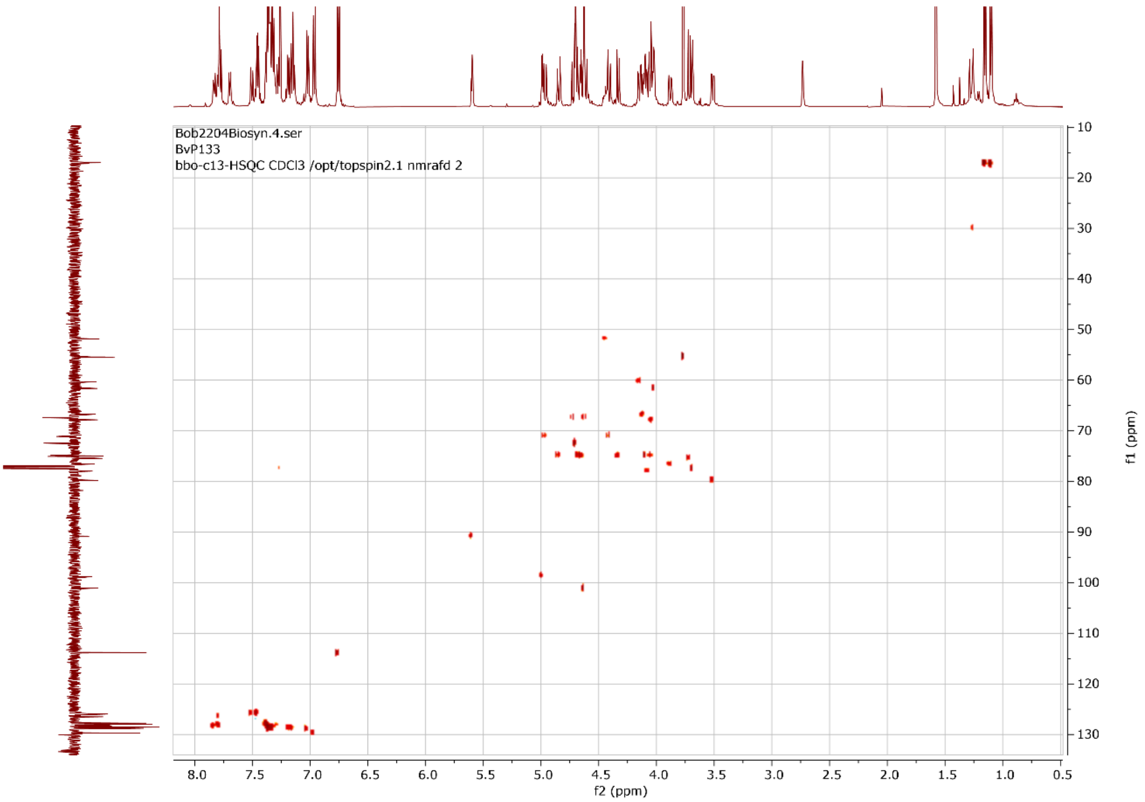
Bob2204Biosyn.4.ser
BvP133
bbo-c13-HSQC CDCl3 /opt/topspin2.1 nmrafd 2
f2 (ppm)
f1 (ppm)

(Benzyl (2-azido-3-*O*-benzyl-2-deoxy-4-*O*-*p*-methoxybenzyl-β-D-mannopyranosiduronsyl)-(1→4)-2-azido-2-deoxy-3-*O*-(2-naphthylmethyl)-α-L-fucopyranosyl-(1→3)-4-*O*-benzyl-2-deoxy-2-*N*-tricfluoroacetamide-β-D-fucopyranose (26-OH)

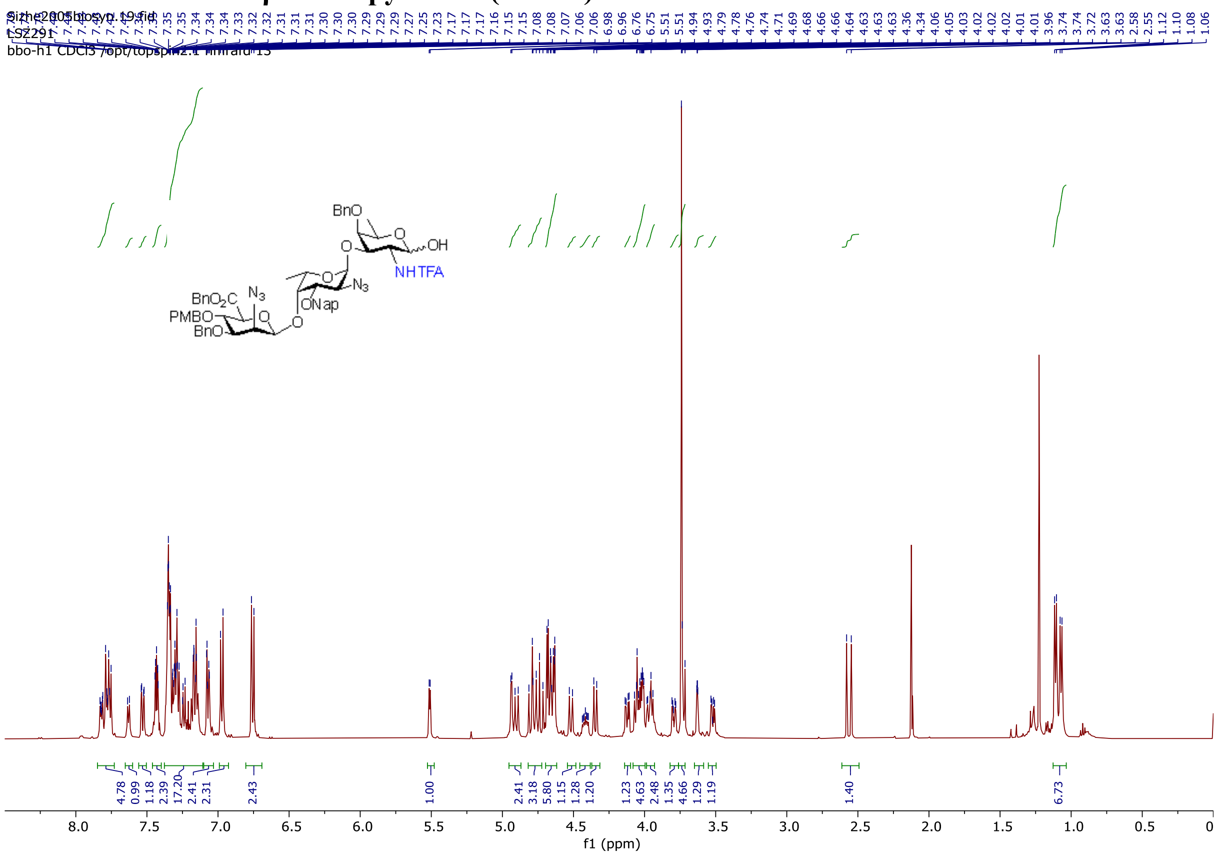

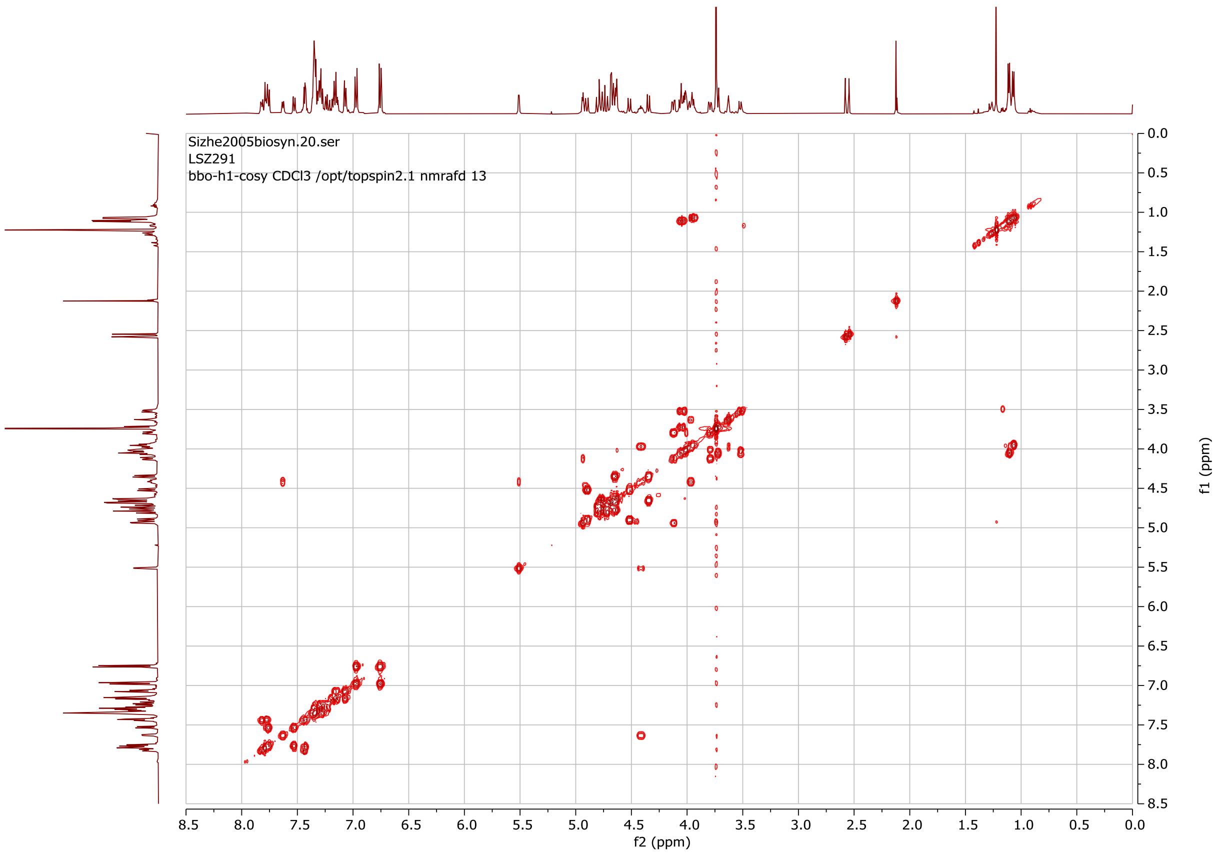
Sizhe2005biosyn.20.ser
LSZ291
bbo-h1-cosy CDCl3 /opt/topspin2.1 nmrafd 13
f2 (ppm)
f1 (ppm)

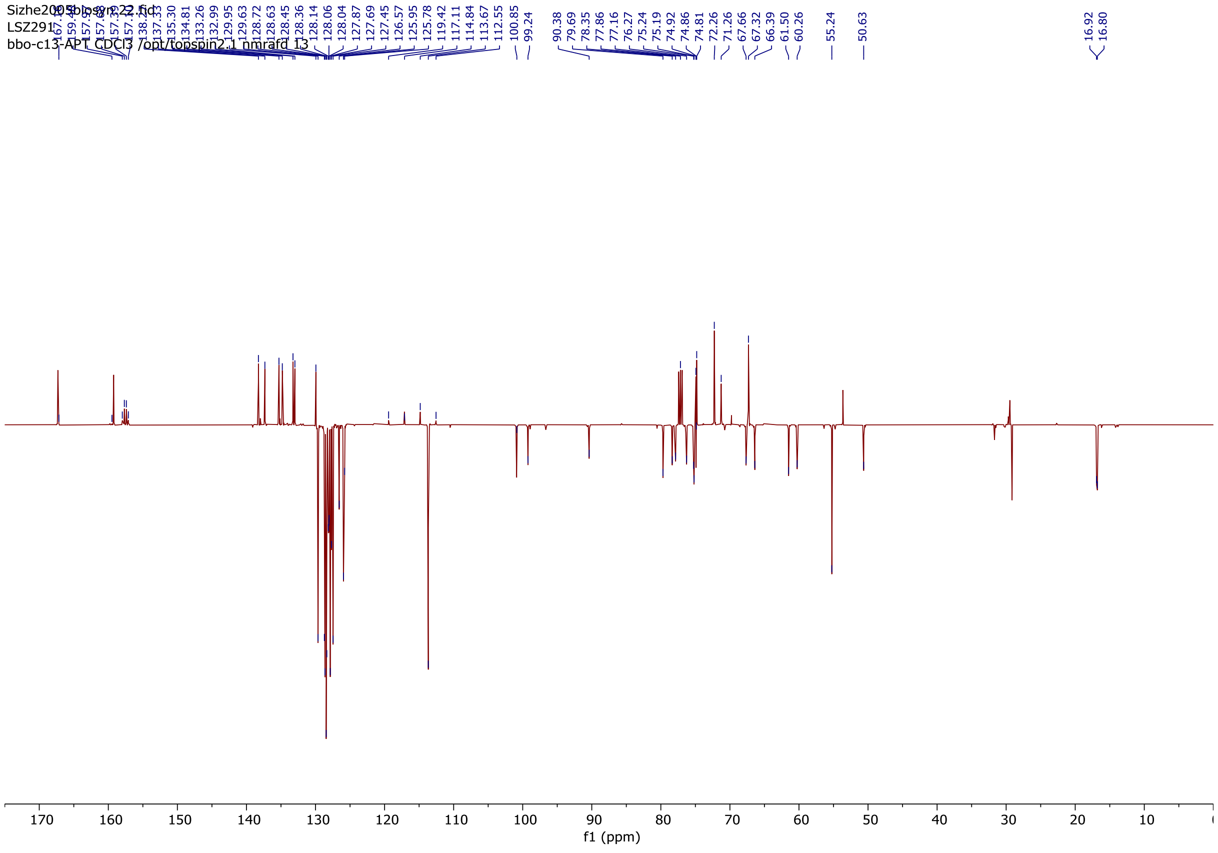
LSZ291
bbo-c13-APT CDCl3 /opt/topspin2.1 nmrafd 13
135.30
134.81
133.26
132.99
129.95
129.63
128.72
128.63
128.45
128.36
128.14
128.06
128.04
127.87
127.69
127.45
126.57
125.95
125.78
119.42
117.11
114.84
113.67
112.55
100.85
99.24
90.38
79.69
78.35
77.86
77.16
76.27
75.24
75.19
74.92
74.86
74.81
72.26
71.26
67.66
67.32
66.39
61.50
60.26
55.24
50.63
16.92
16.80
f1 (ppm)

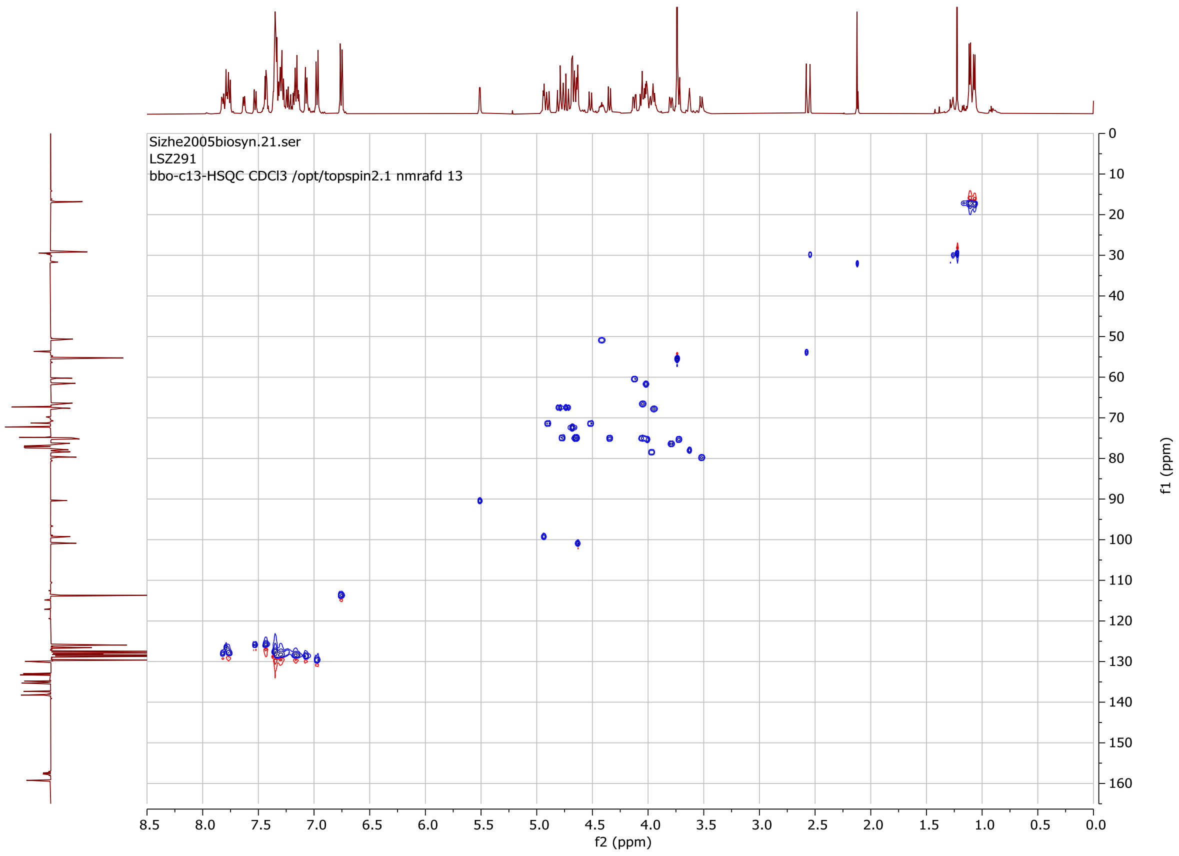

(Benzyl (2-azido-3-*O*-benzyl-2-deoxy-4-*O*-*p*-methoxybenzyl-β-D-mannopyranosiduronsyl)-(1→4)-2-azido-2-deoxy-3-*O*-(2-naphthylmethyl)-α-L-fucopyranosyl-(1→3)-4-*O*-benzyl-2-deoxy-2-*N*-trichloroacetamide-1-*O*-(*N*-phenyl-2,2,2-trifluoroacetimidoyl)-β-D-fucopyranose (25)

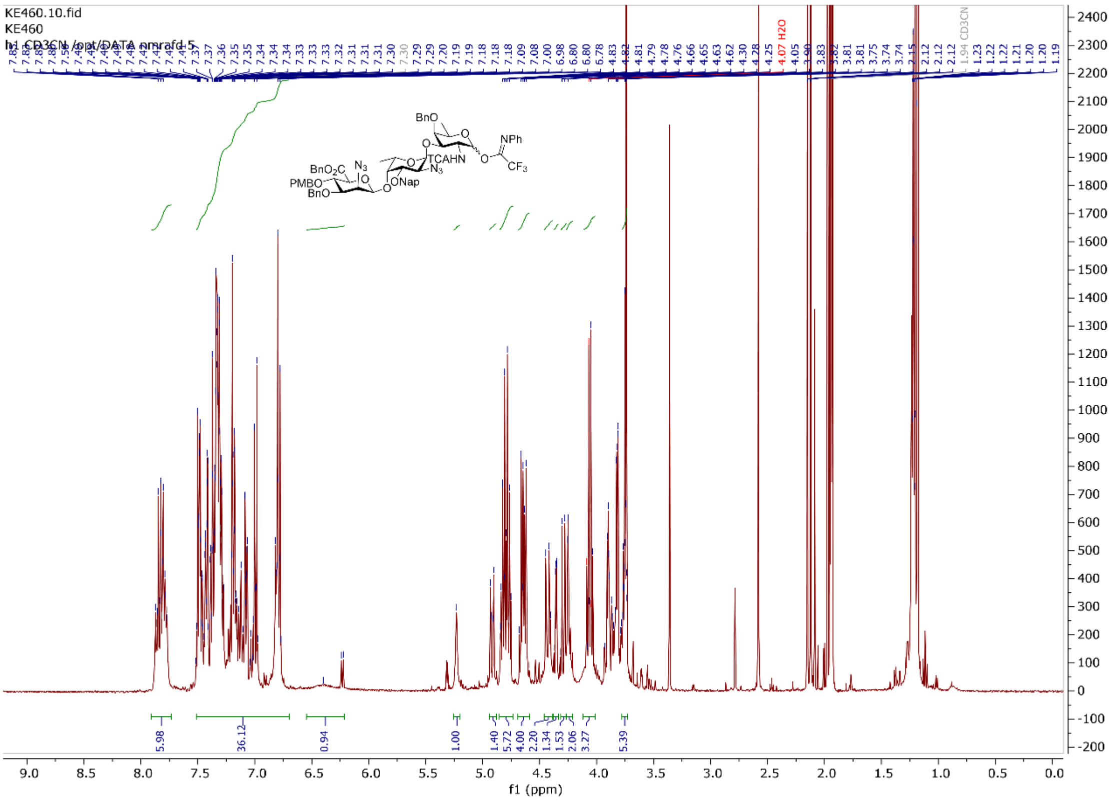
KE460.10.fid
KE460
h1 CD3CN /opt/DATA nmrafd 5
4.07 H2O
1.94 CD3CN
f1 (ppm)

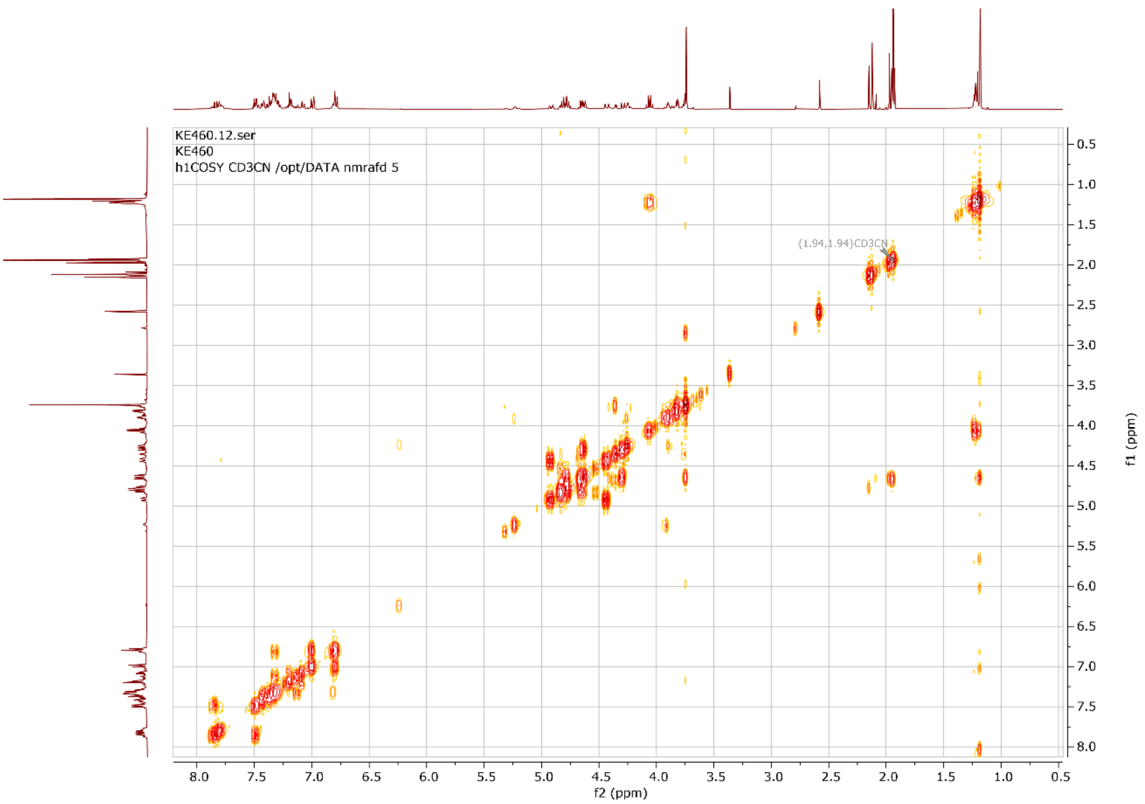
KE460.12.ser
KE460
h1COSY CD3CN /opt/DATA nmrafd 5
{1.94,1.94}CD3CN
f2 (ppm)
f1 (ppm)

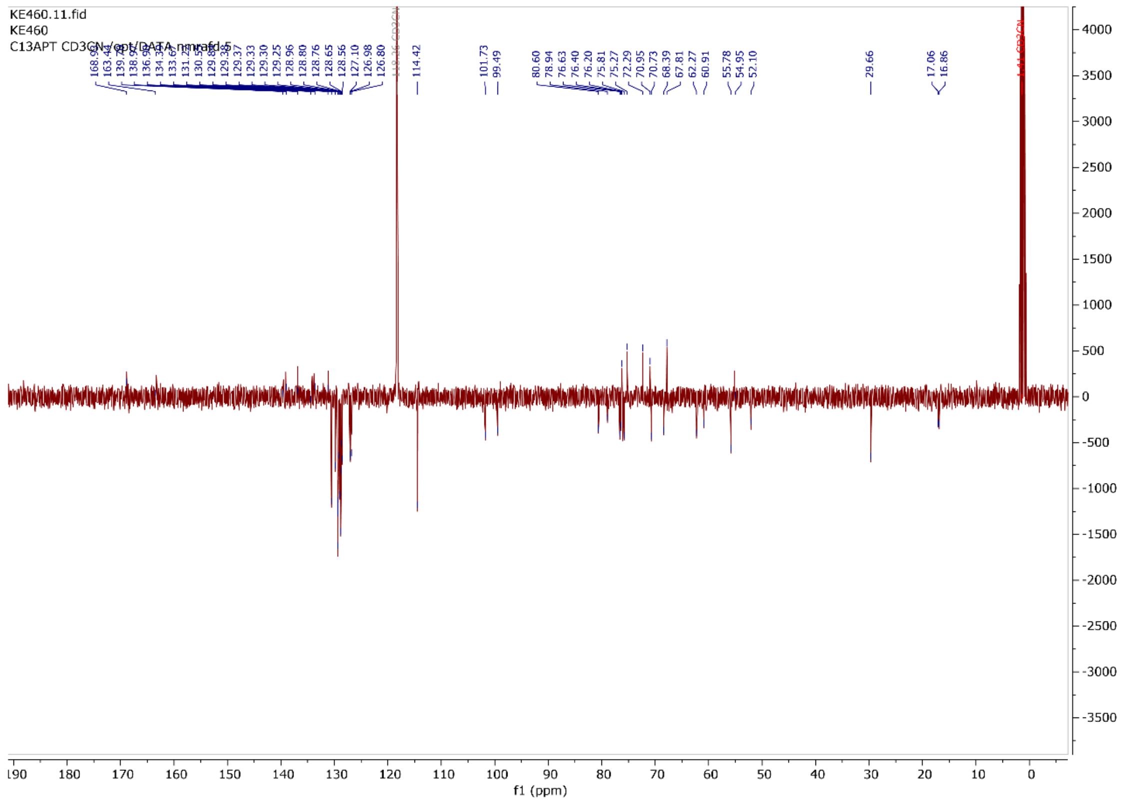

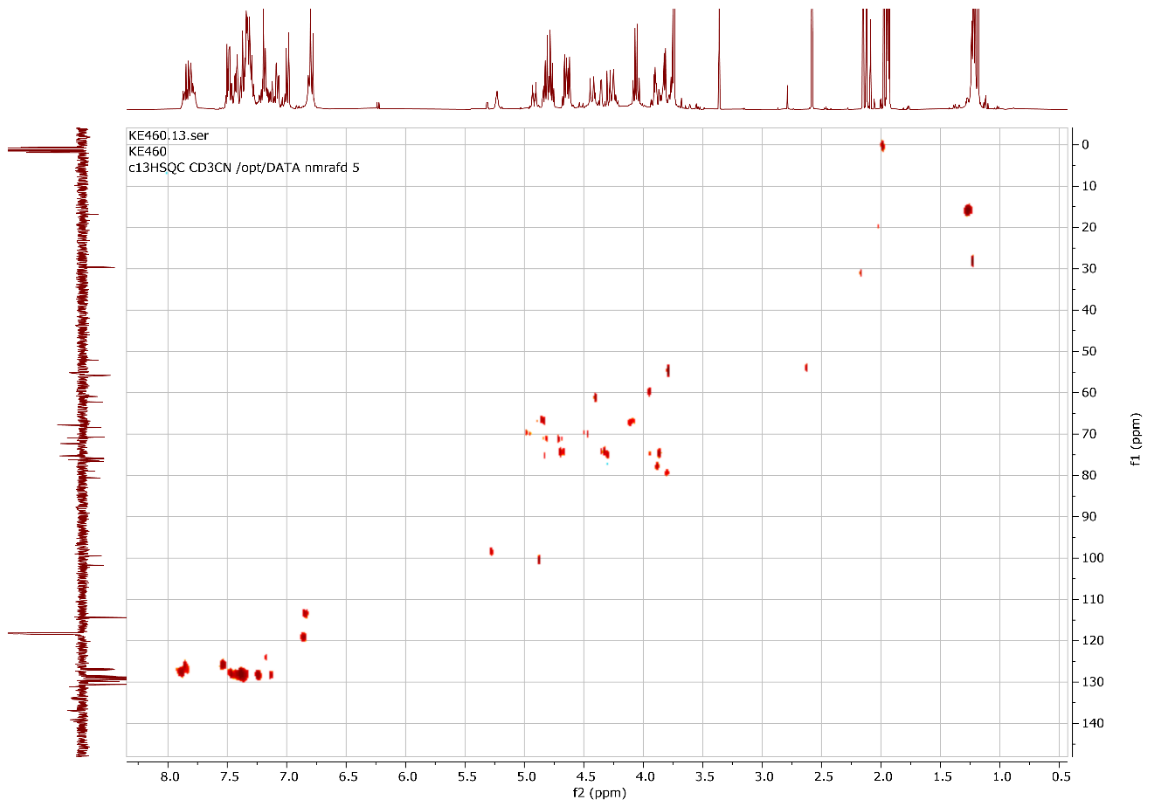

(Benzyl (2-azido-3,4-di-*O*-benzyl-2-deoxy-β-D-mannopyranosiduronsyl)-(1→4)-2-azido-2-deoxy-3-*O*-(2-naphthylmethyl)-α-L-fucopyranosyl-(1→3)-4-*O*-benzyl-2-deoxy-2-*N*-tricfluoroacetamide-β-D-fucopyranose (27-OH)

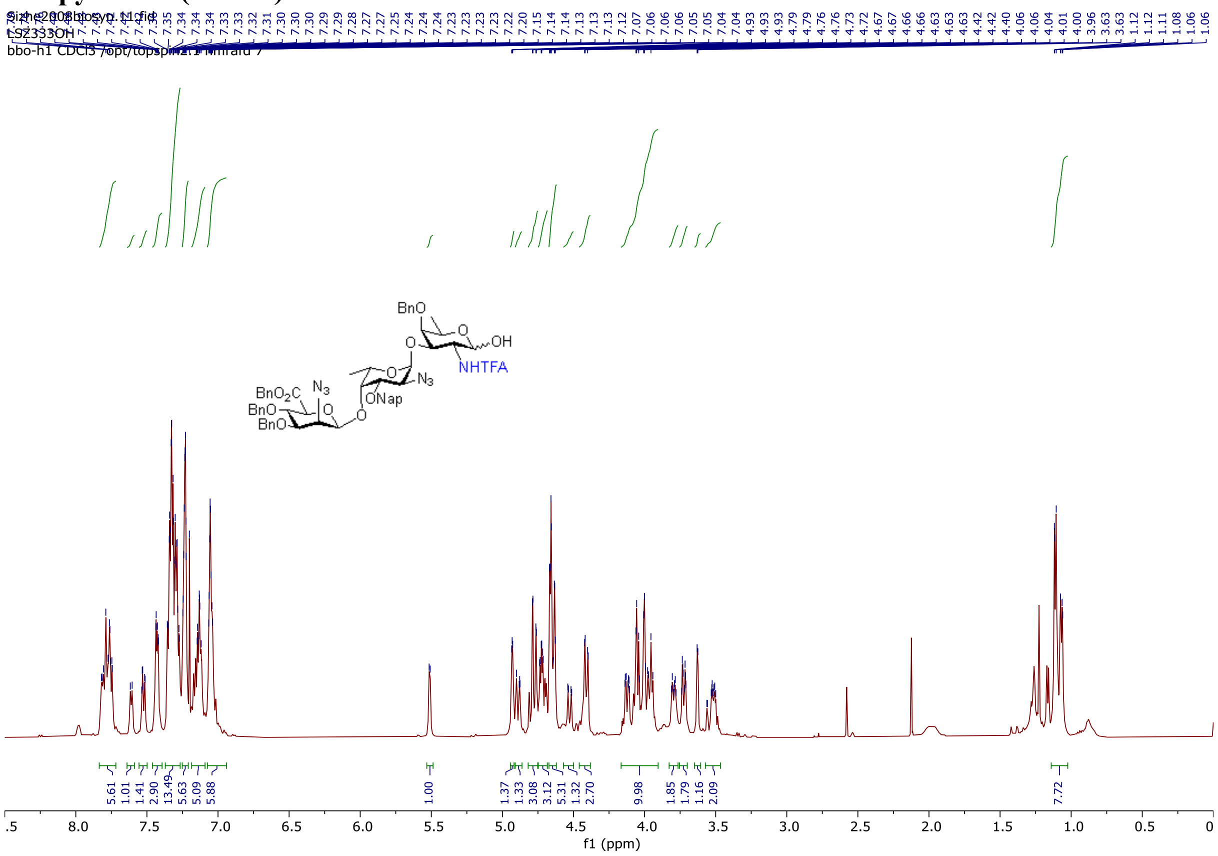

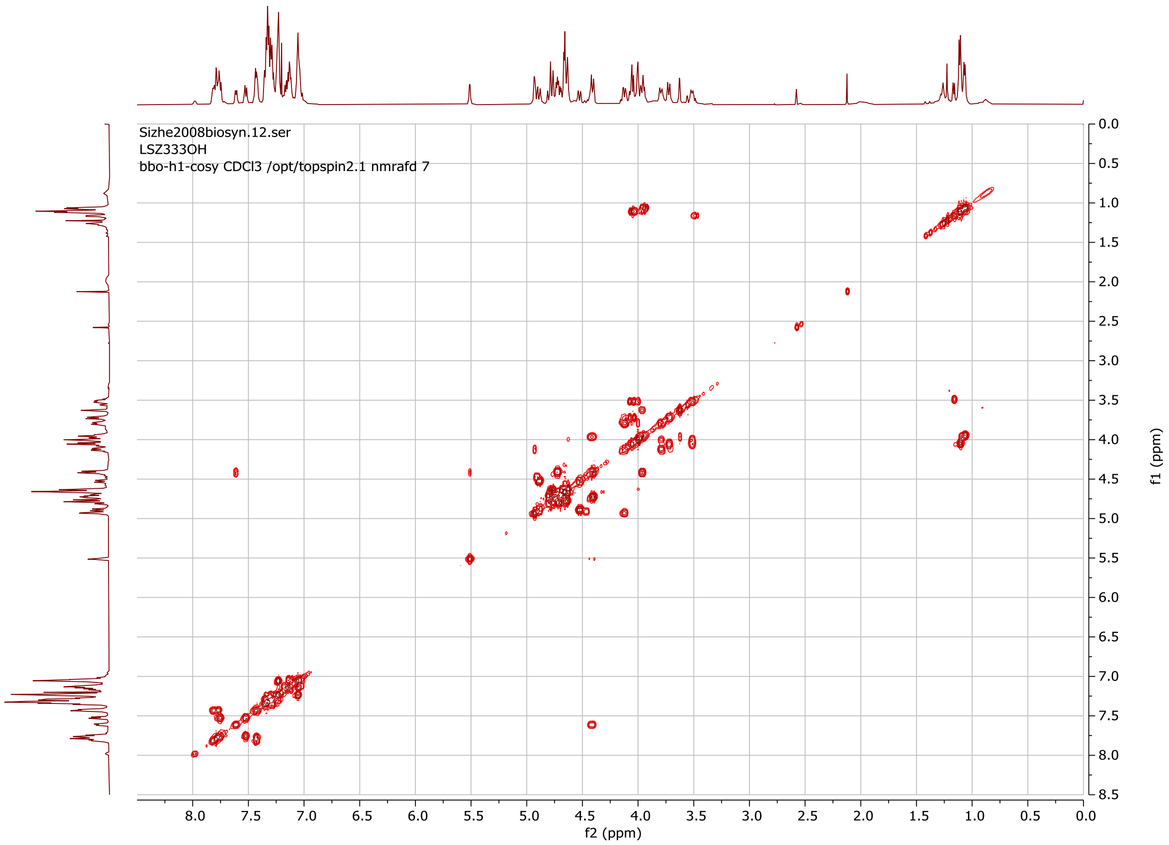

Sizhe2008biosyn.12.ser
LSZ333OH
bbo-h1-cosy CDCl3 /opt/topspin2.1 nmrafd 7
f2 (ppm)
f1 (ppm)

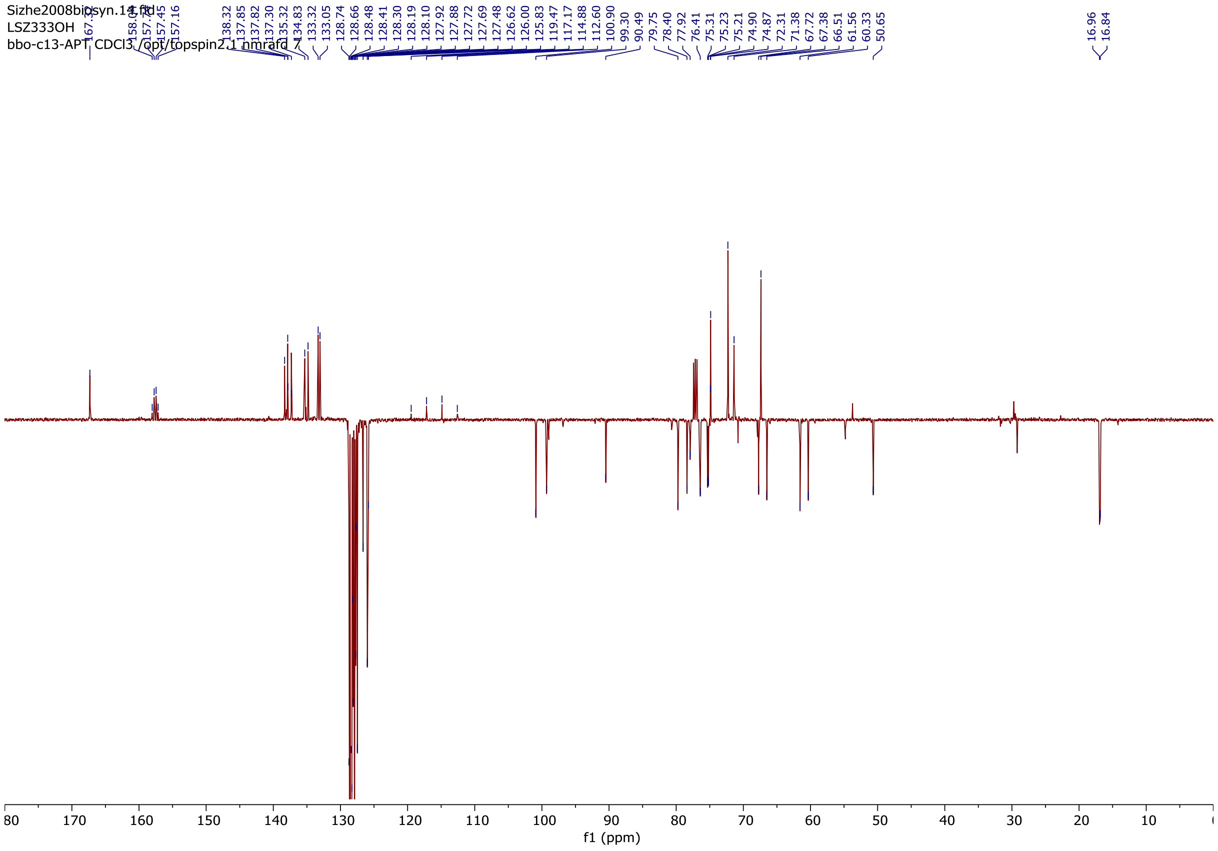

Sizhe2008biosyn.14.fid
LSZ333OH
bbo-c13-APT CDCl3 /opt/topspin2.1 nmrafd 7
157.45
157.16
138.32
137.85
137.82
137.30
135.32
134.83
133.32
133.05
128.74
128.66
128.48
128.41
128.30
128.19
128.10
127.92
127.88
127.72
127.69
127.48
126.62
126.00
125.83
119.47
117.17
114.88
112.60
100.90
99.30
90.49
79.75
78.40
77.92
76.41
75.31
75.23
75.21
74.90
74.87
72.31
71.38
67.72
67.38
66.51
61.56
60.33
50.65
16.96
16.84
f1 (ppm)

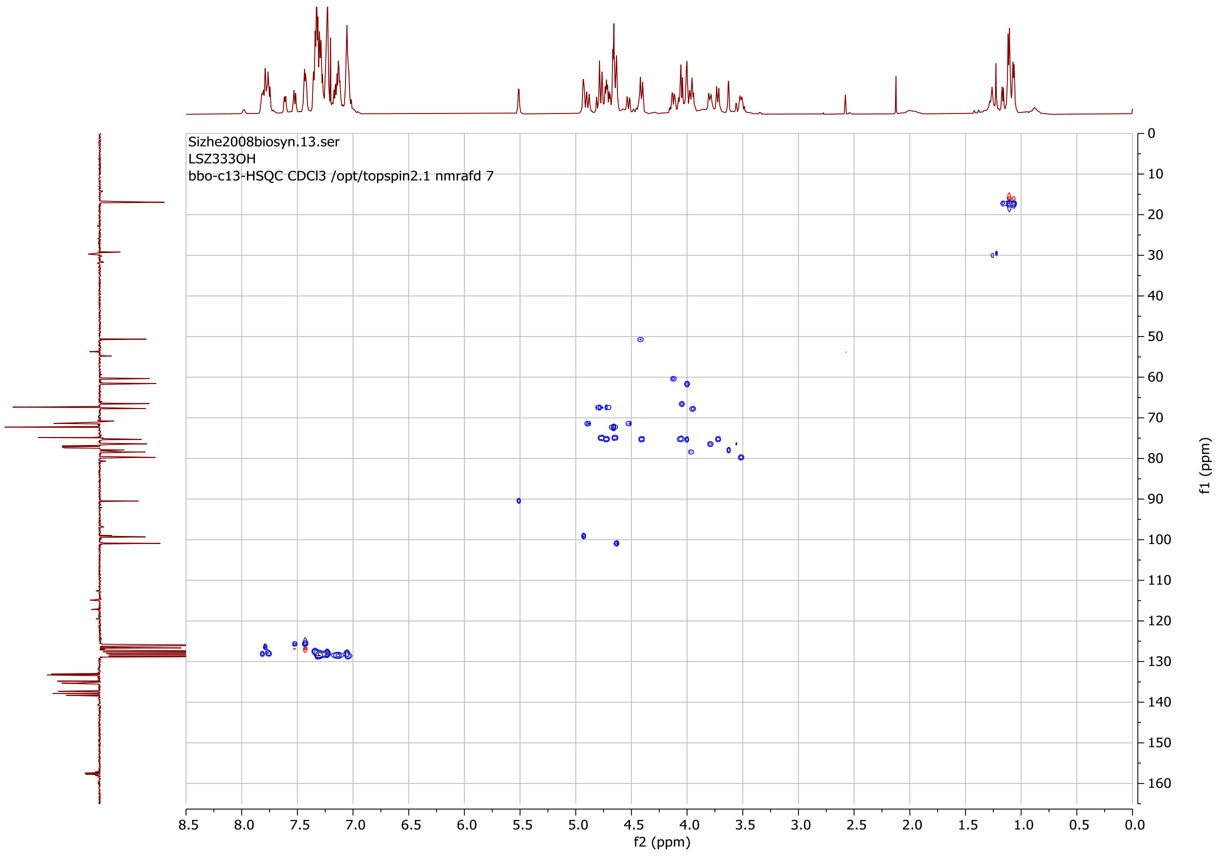

5-(Benzyl(benzyloxycarbonyl)amino)pentyl (Benzyl (2-azido-3-*O*-benzyl-2-deoxy-4-*O*-*p*-methoxybenzyl-β-D-mannopyranosiduronsyl)-(1→4)-2-azido-2-deoxy-3-*O*-(2-naphthylmethyl)-α-L-fucopyranosyl-(1→3)-4-*O*-benzyl-2-deoxy-2-*N*-trichloroacetamide-α-D-fucopyranoside (29)

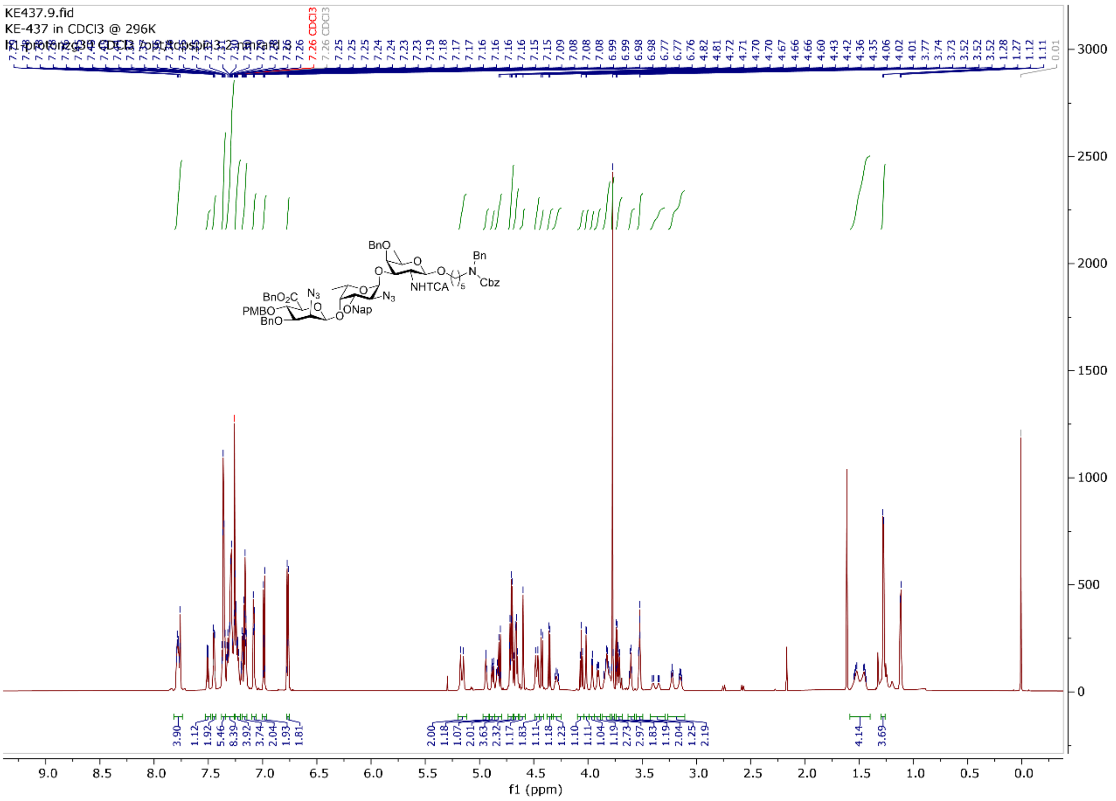
KE437.9.fid
KE-437 in CDCl3 @ 296K
f1 (ppm)

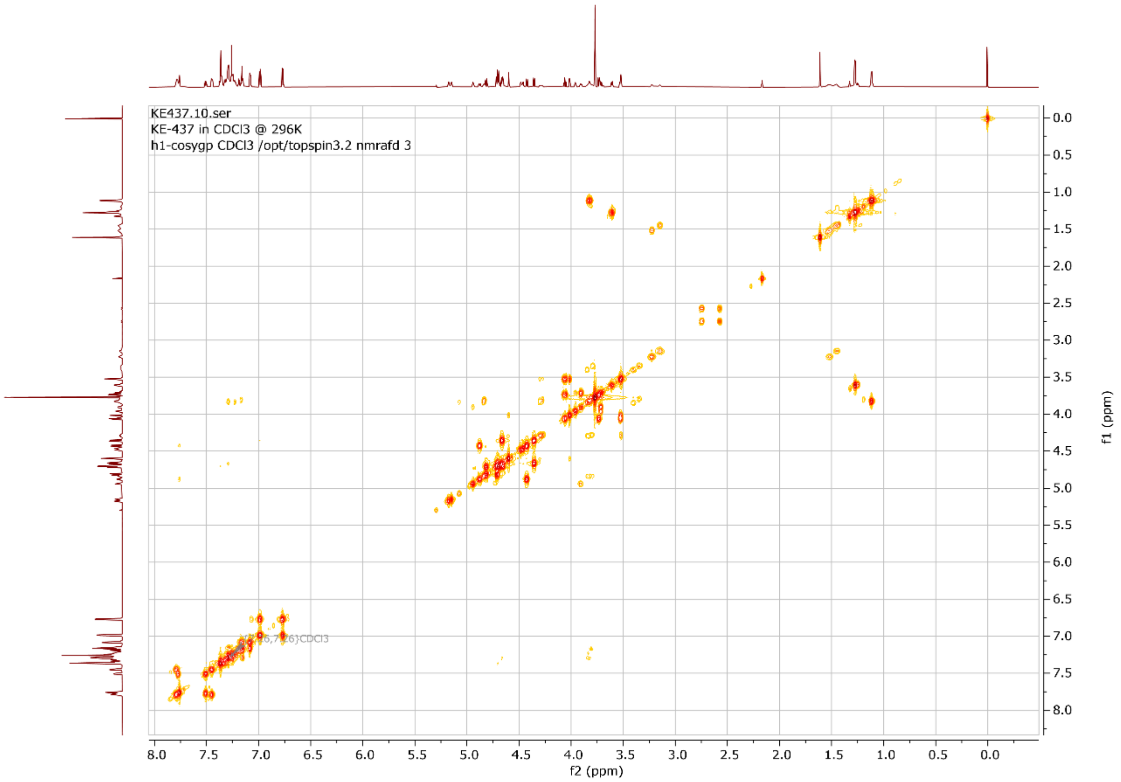
KE437.10.ser
KE-437 in CDCl3 @ 296K
h1-cosygp CDCl3 /opt/topspin3.2 nmrafd 3
f2 (ppm)
f1 (ppm)

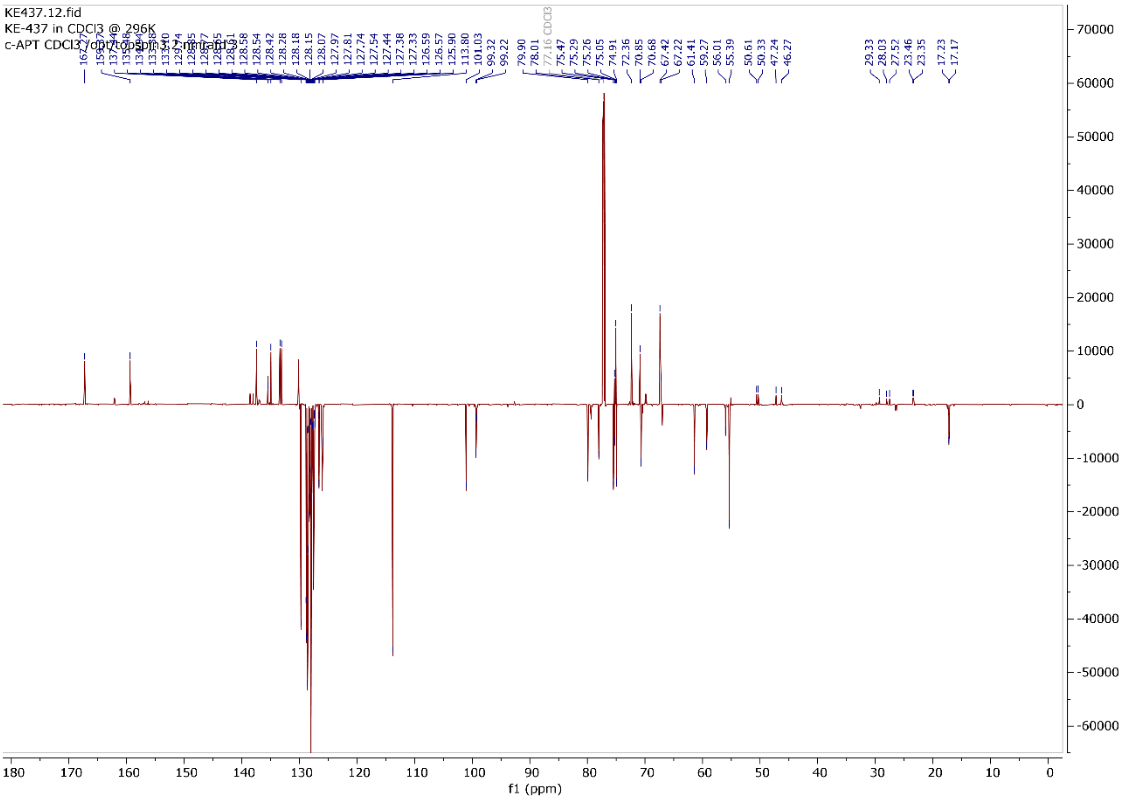
KE437.12.fid
KE-437 in CDCl3 @ 296K
c-APT CDCl3 /opt/topspin3.2 nmrafd 3
128.58
128.54
128.42
128.28
128.18
128.15
128.07
127.97
127.81
127.74
127.54
127.44
127.38
127.33
126.59
126.57
125.90
113.80
101.03
99.32
99.22
79.90
78.01
77.16 CDCl3
75.47
75.29
75.26
75.05
74.91
72.36
70.85
70.68
67.42
67.22
61.41
59.27
56.01
55.39
50.61
50.33
47.24
46.27
29.33
28.03
27.52
23.46
23.35
17.23
17.17
f1 (ppm)

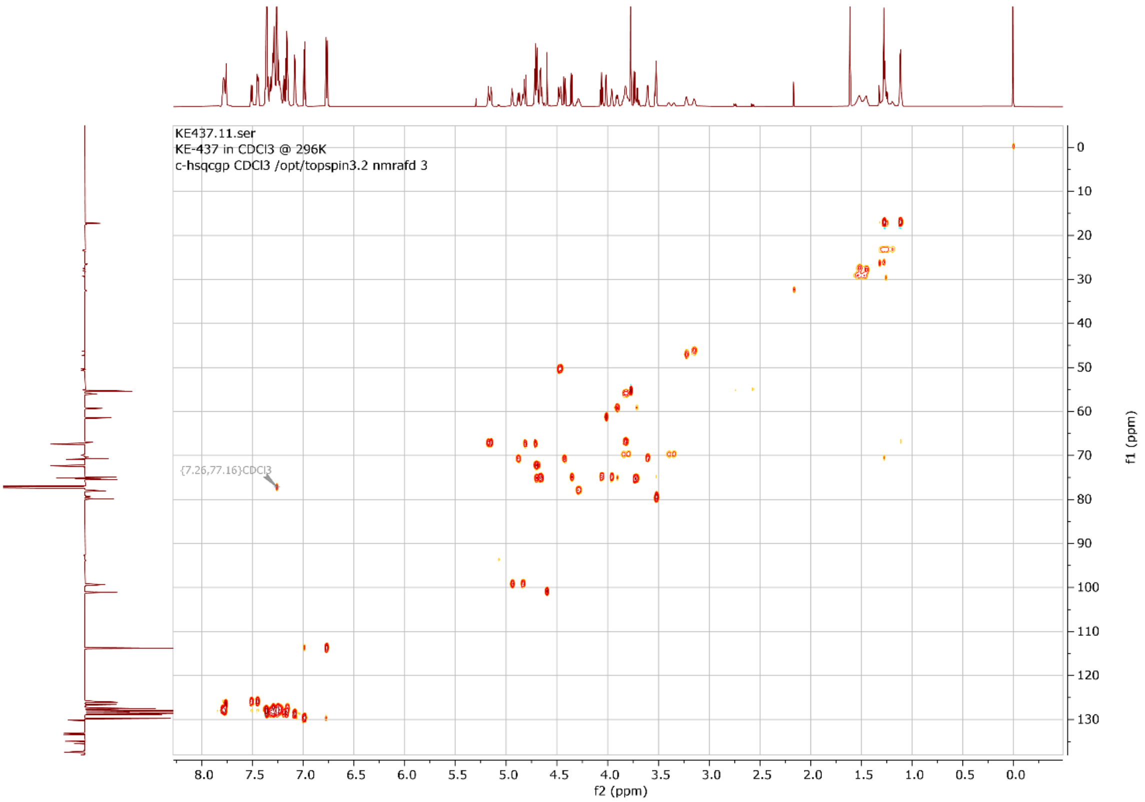
KE437.11.ser
KE-437 in CDCl3 @ 296K
c-hsqcgp CDCl3 /opt/topspin3.2 nmrafd 3
{7.26,77.16}CDCl3
f2 (ppm)
f1 (ppm)

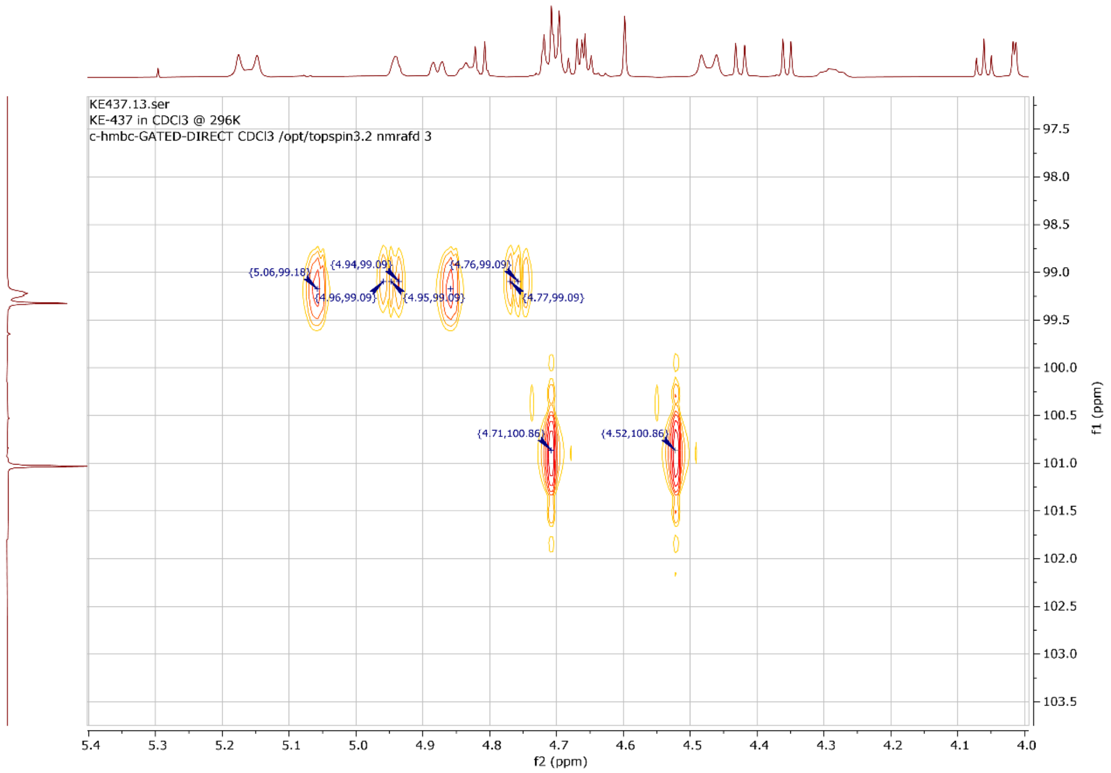

1-allylbutanyl (Benzyl (2-azido-3-*O*-benzyl-2-deoxy-4-*O*-*p*-methoxybenzyl-β-D-mannopyranosiduronsyl)-(1→4)-2-azido-2-deoxy-3-*O*-(2-naphthylmethyl)-α-L-fucopyranosyl-(1→3)-4-*O*-benzyl-2-deoxy-2-*N*-trifluoroacetamide-α-D-fucopyranoside (31)

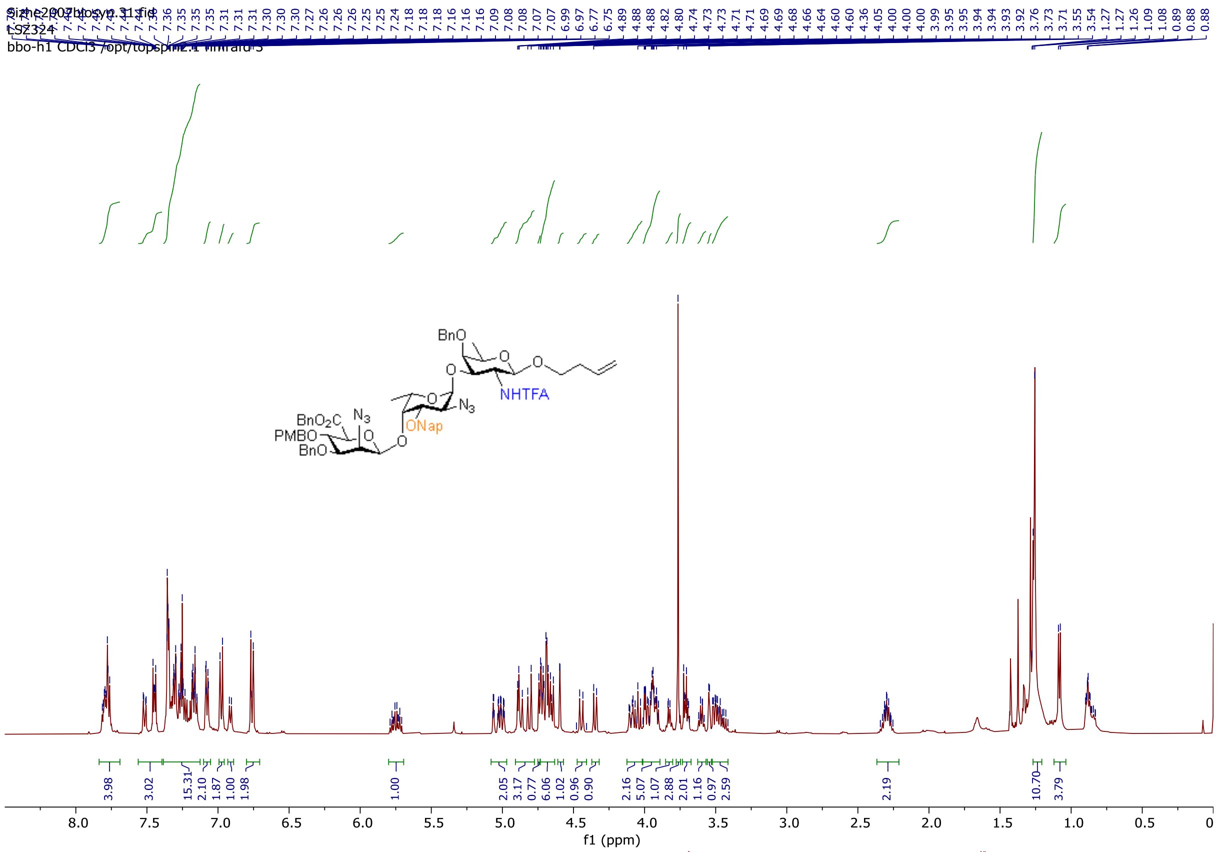
Sizhe2007biosyn.31.fid
LSZ324
bbo-h1 CDCl3 /opt/topspin2.1 nmrafd 3
7.36 7.35 7.35 7.35 7.31 7.31 7.31 7.30 7.30 7.30 7.27 7.26 7.26 7.26 7.25 7.25 7.24 7.18 7.18 7.16 7.16 7.16 7.09 7.08 7.08 7.07 7.07 6.99 6.97 6.77 6.75 4.89 4.88 4.88 4.82 4.80 4.74 4.73 4.73 4.71 4.71 4.69 4.69 4.68 4.66 4.64 4.60 4.60 4.36 4.05 4.00 4.00 4.00 3.99 3.95 3.95 3.94 3.94 3.93 3.92 3.76 3.73 3.71 3.55 3.54 1.27 1.27 1.26 1.09 1.08 0.89 0.88 0.88
BnO
O
O
NHTFA
O
N3
BnO2C
N3
PMBO
BnO
O
O
ONap
3.98
3.02
15.31
2.10
1.87
1.00
1.98
1.00
2.05
3.17
0.77
6.06
1.02
0.96
0.90
2.16
5.07
1.07
2.88
2.01
1.16
0.97
2.59
2.19
10.70
3.79
8.0 7.5 7.0 6.5 6.0 5.5 5.0 4.5 4.0 3.5 3.0 2.5 2.0 1.5 1.0 0.5 0
f1 (ppm)

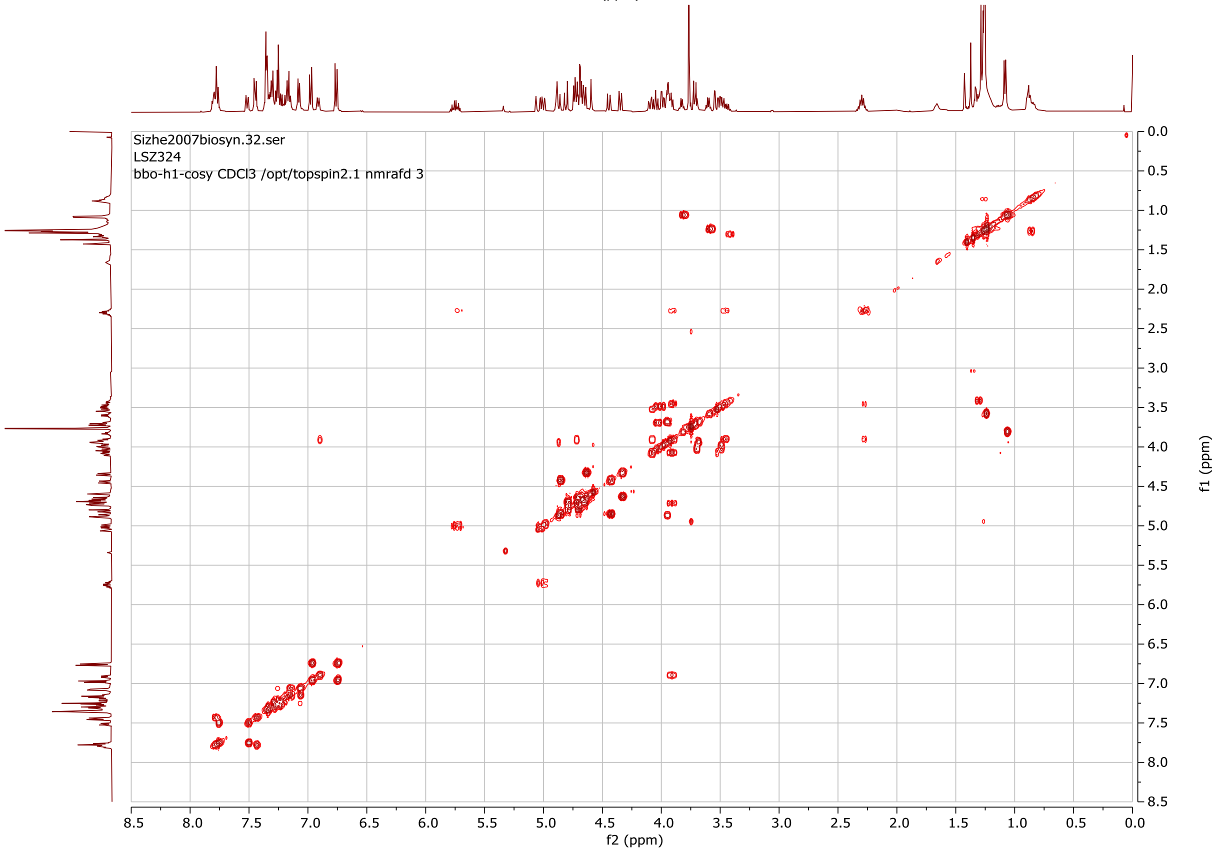
Sizhe2007biosyn.32.ser
LSZ324
bbo-h1-cosy CDCl3 /opt/topspin2.1 nmrafd 3
8.5 8.0 7.5 7.0 6.5 6.0 5.5 5.0 4.5 4.0 3.5 3.0 2.5 2.0 1.5 1.0 0.5 0.0
f2 (ppm)
f1 (ppm)

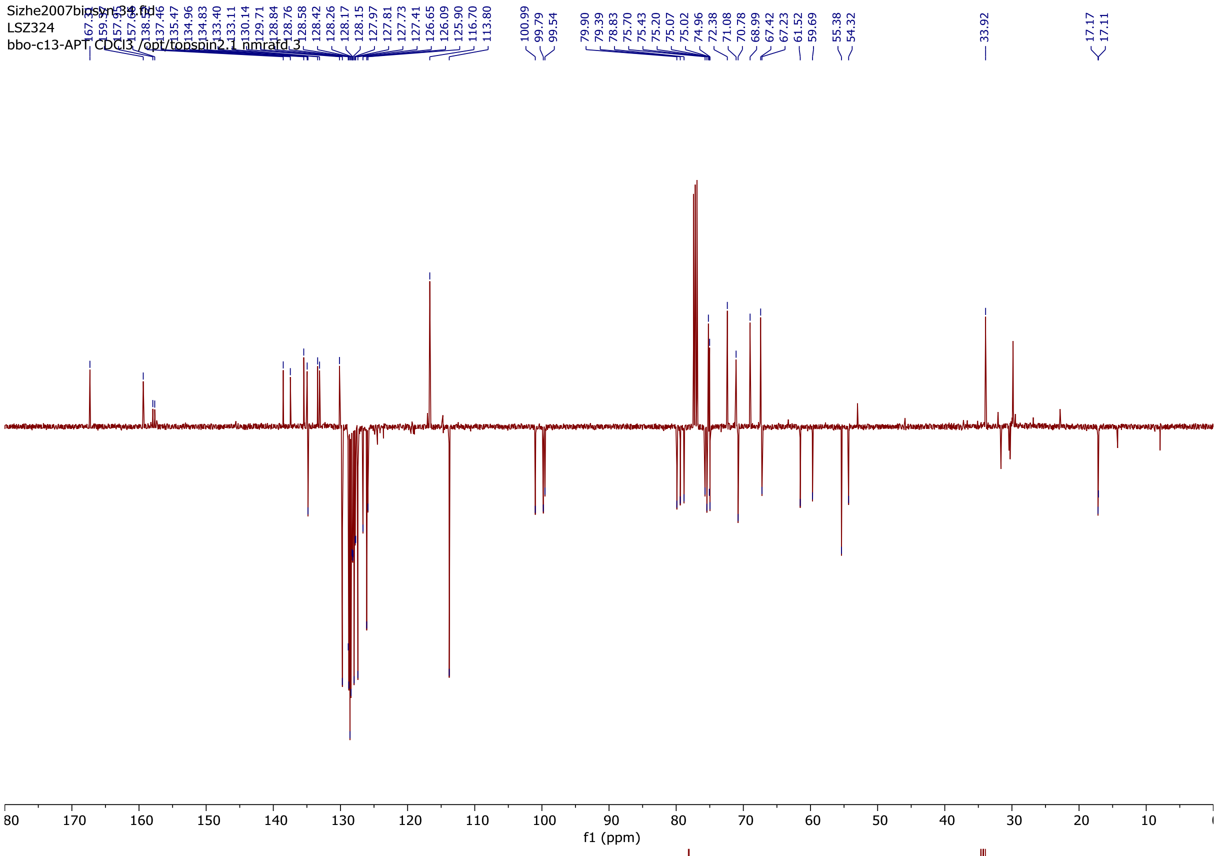
LSZ324
bbo-c13-APT CDCl3 /opt/topspin2.1 nmrafd 3
137.46
135.47
134.96
134.83
133.40
133.11
130.14
129.71
128.84
128.76
128.58
128.42
128.26
128.17
128.15
127.97
127.81
127.73
127.41
126.65
126.09
125.90
116.70
113.80
100.99
99.79
99.54
79.90
79.39
78.83
75.70
75.43
75.20
75.07
75.02
74.96
72.38
71.08
70.78
68.99
67.42
67.23
61.52
59.69
55.38
54.32
33.92
17.17
17.11
f1 (ppm)

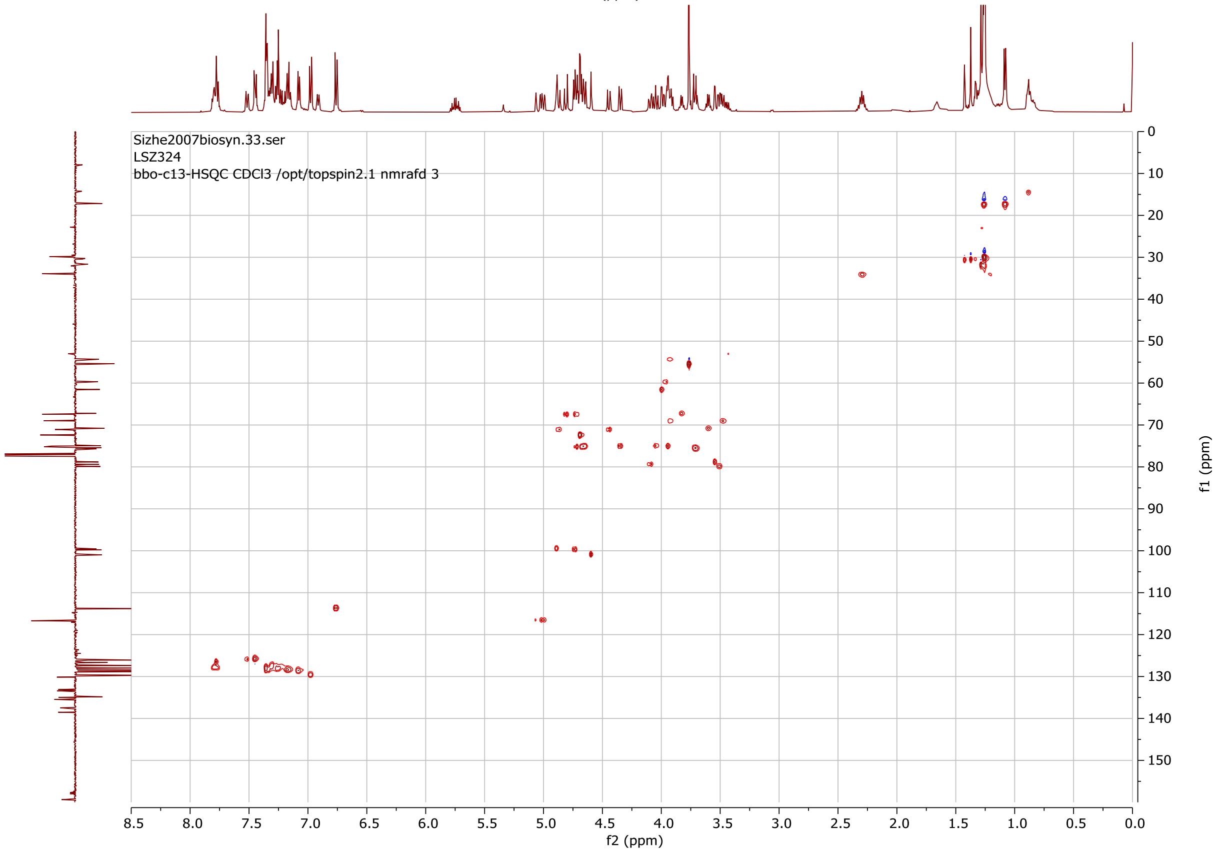
Sizhe2007biosyn.33.ser
LSZ324
bbo-c13-HSQC CDCl3 /opt/topspin2.1 nmrafd 3
f2 (ppm)
f1 (ppm)

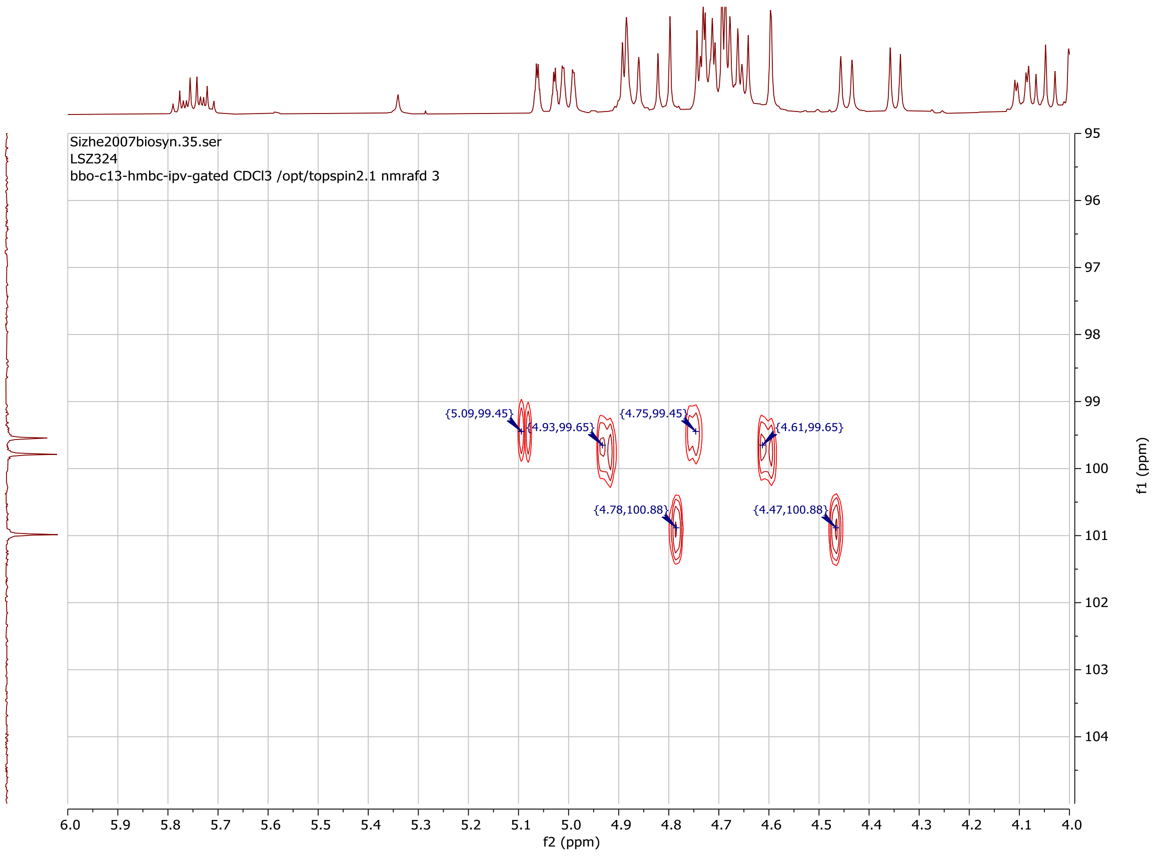

5-(Benzyl(benzyloxycarbonyl)amino)pentyl (Benzyl (2-azido-3-*O*-benzyl-2-deoxy-β-D-mannopyranosiduronsyl)-(1→4)-2-azido-2-deoxy-3-*O*-(2-naphthylmethyl)-α-L-fucopyranosyl-(1→3)-4-*O*-benzyl-2-deoxy-2-*N*-trichloroacetamide-α-D-fucopyranoside (30)

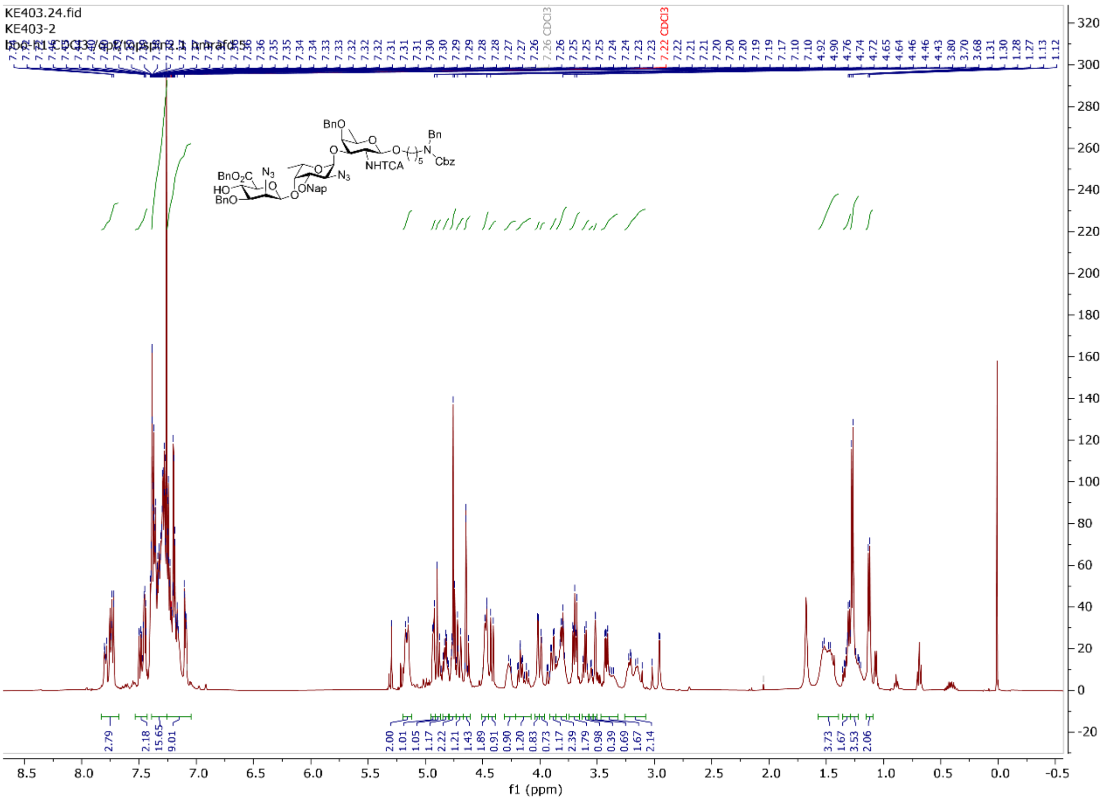
KE403.24.fid
KE403-2
f1 (ppm)
BnO
Bn
NHTCA
Cbz
BnO$_2$C
N_3
HO
BnO
ONap

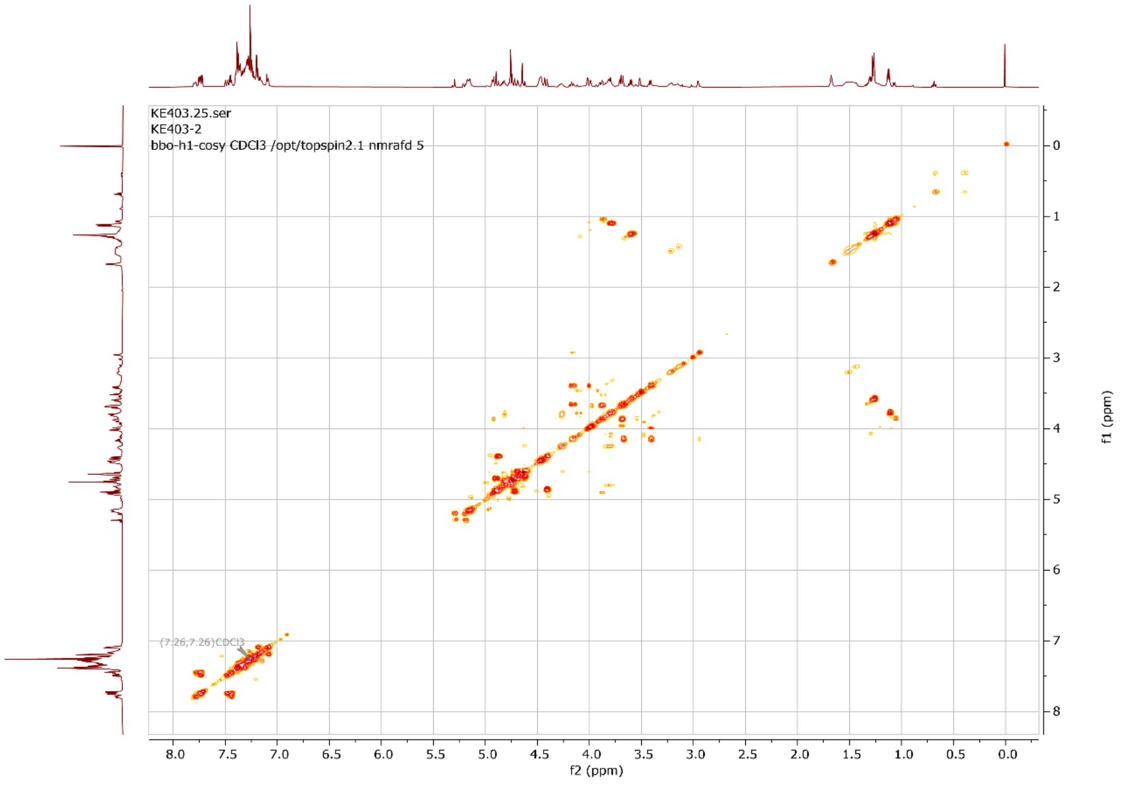
KE403.25.ser
KE403-2
bbo-h1-cosy CDCl3 /opt/topspin2.1 nmrafd 5
f2 (ppm)
f1 (ppm)

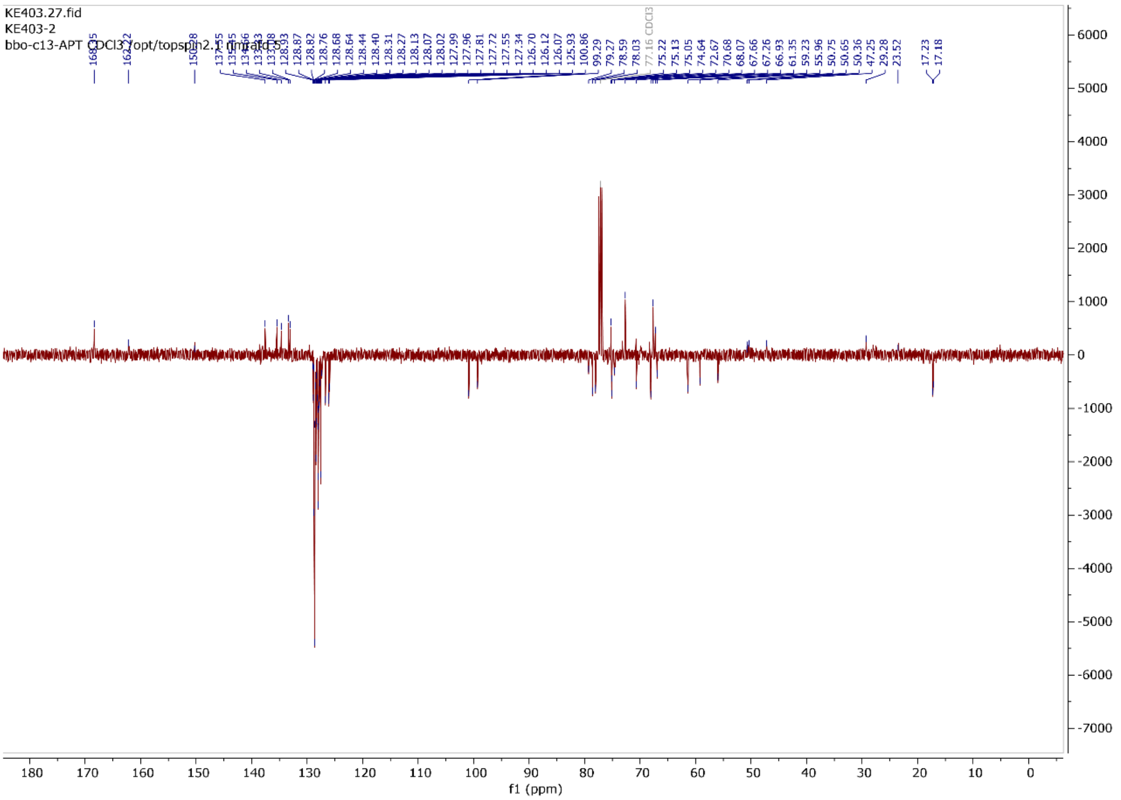

KE403.27.fid
KE403-2
bbo-c13-APT CDCl3 /opt/topspin2.1 nmrafd 5
162.22
137.55
128.93
128.87
128.82
128.76
128.68
128.64
128.44
128.40
128.31
128.27
128.13
128.07
128.02
127.99
127.96
127.81
127.72
127.55
127.34
126.70
126.12
126.07
125.93
100.86
99.29
79.27
78.59
78.03
77.16 CDCl3
75.22
75.13
75.05
74.64
72.67
70.68
68.07
67.66
67.26
66.93
61.35
59.23
55.96
50.75
50.65
50.36
47.25
29.28
23.52
17.23
17.18
f1 (ppm)

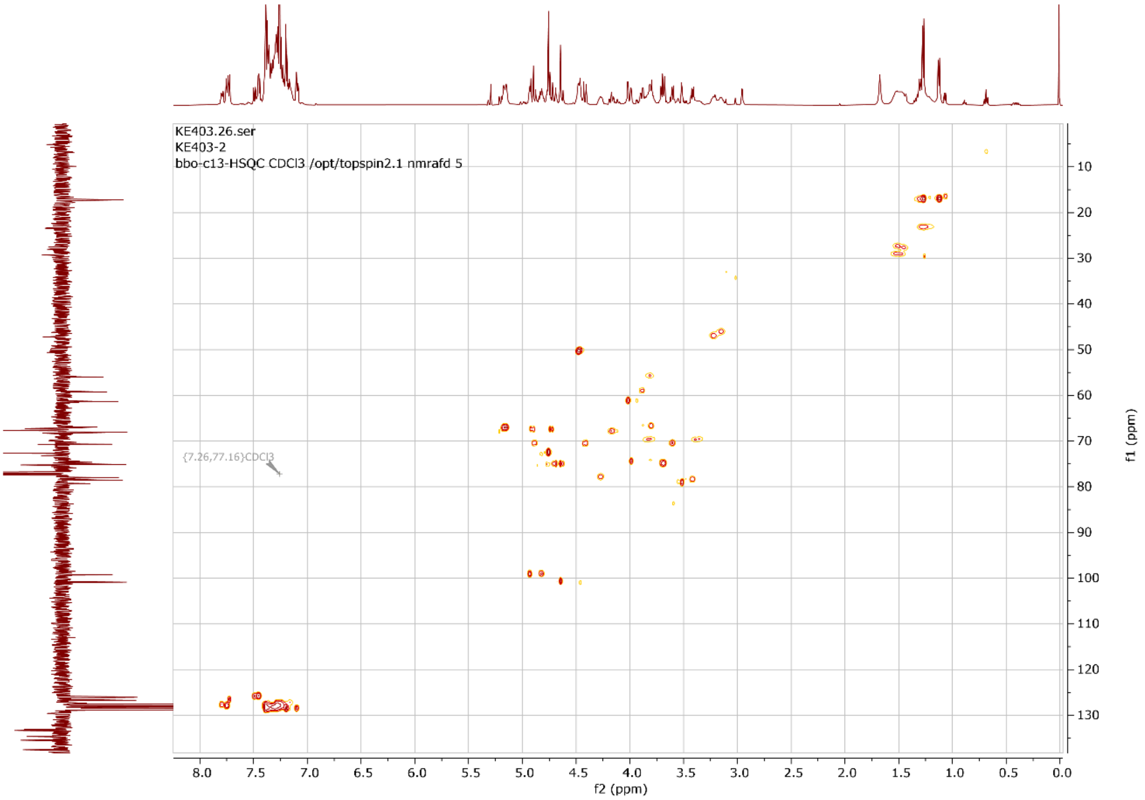

KE403.26.ser
KE403-2
bbo-c13-HSQC CDCl3 /opt/topspin2.1 nmrafd 5
{7.26,77.16}CDCl3
f2 (ppm)
f1 (ppm)

1-allylbutanyl (Benzyl (2-azido-3-*O*-benzyl-2-deoxy-β-D-mannopyranosiduronsyl)-(1→4)-2-azido-2-deoxy-3-*O*-(2-naphthylmethyl)-α-L-fucopyranosyl-(1→3)-4-*O*-benzyl-2-deoxy-2-*N*-trifluoroacetamide-α-D-fucopyranoside (32)

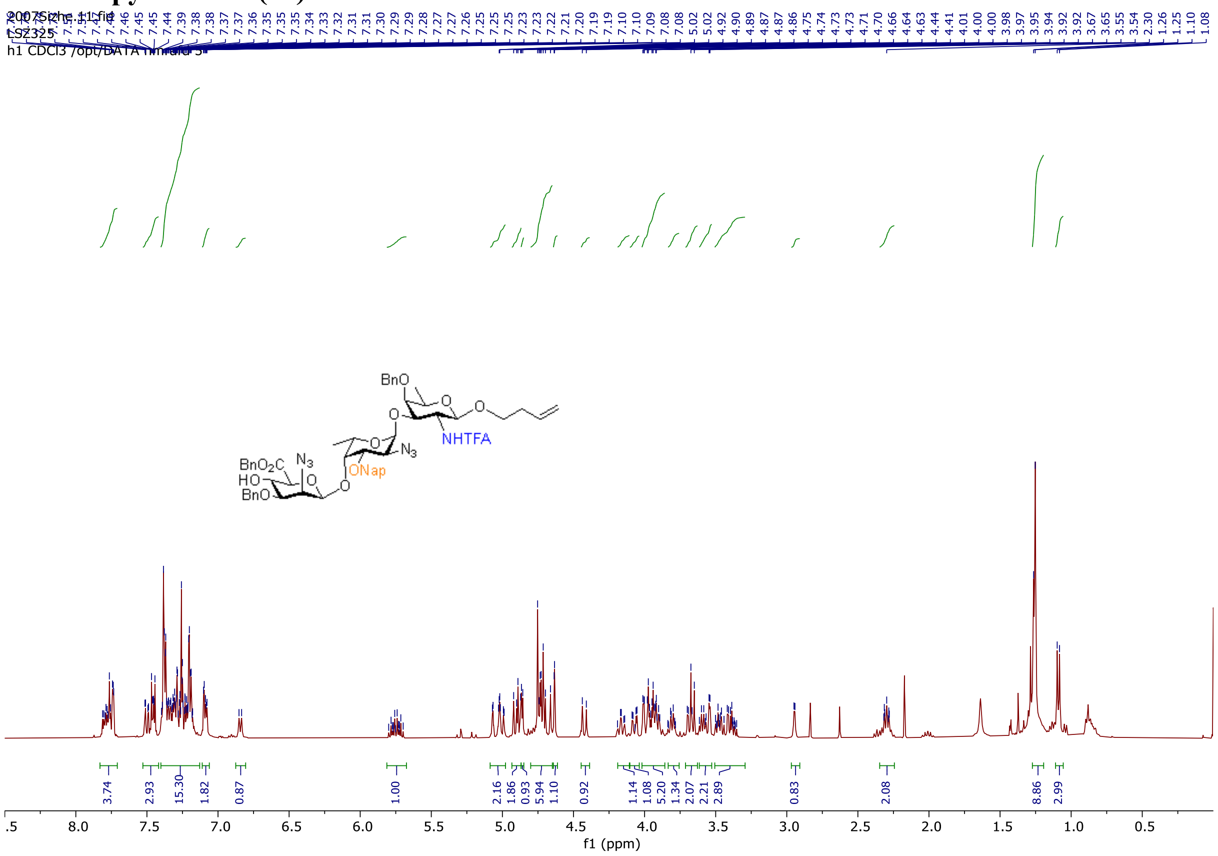

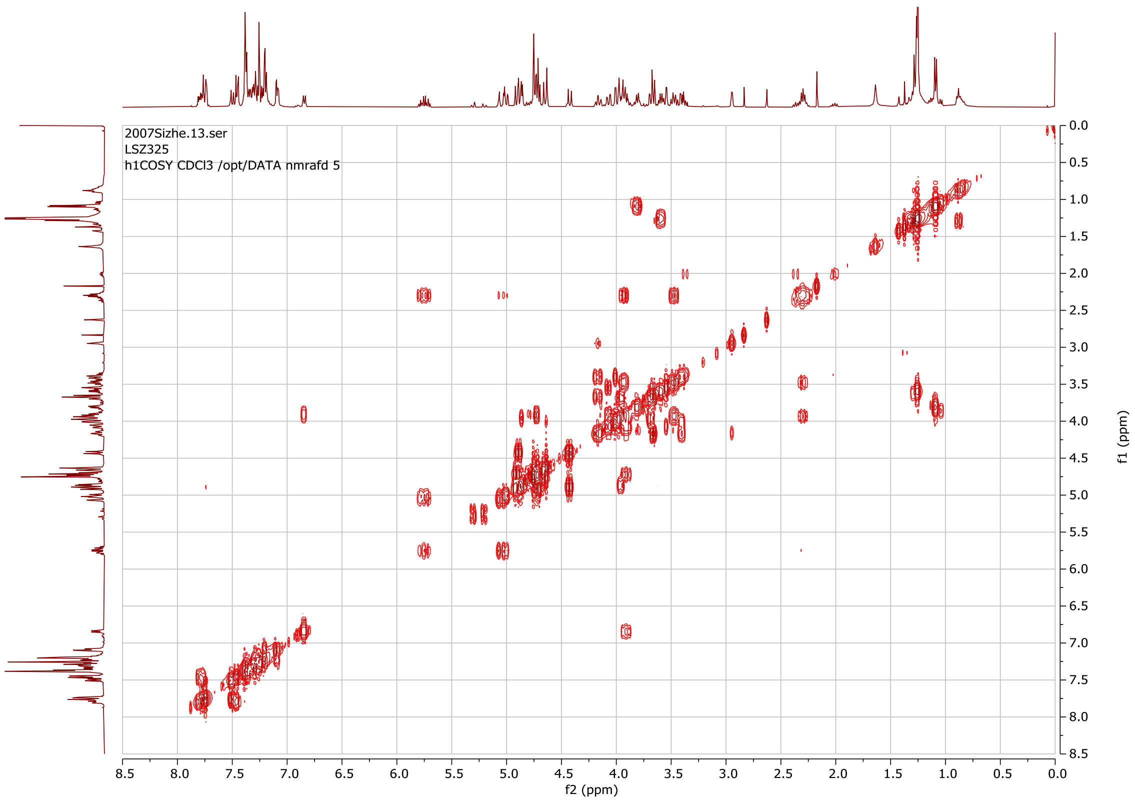
2007Sizhe.13.ser
LSZ325
h1COSY CDCl3 /opt/DATA nmrafd 5
f2 (ppm)
f1 (ppm)

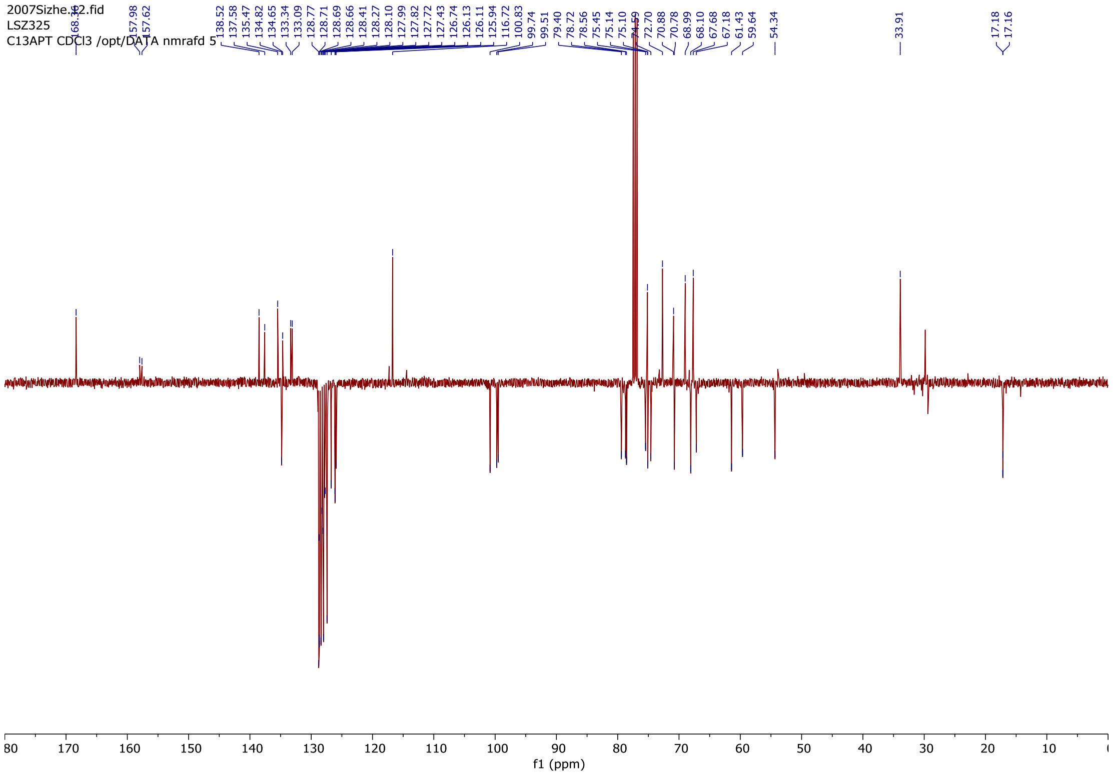
2007Sizhe.12.fid
LSZ325
C13APT CDCl3 /opt/DATA nmrafd 5
157.98
157.62
138.52
137.58
135.47
134.82
134.65
133.34
133.09
128.77
128.71
128.69
128.66
128.41
128.27
128.10
127.99
127.82
127.72
127.43
126.74
126.13
126.11
125.94
116.72
100.83
99.74
99.51
79.40
78.72
78.56
75.45
75.14
75.10
72.70
70.88
70.78
68.99
68.10
67.68
67.18
61.43
59.64
54.34
33.91
17.18
17.16
f1 (ppm)

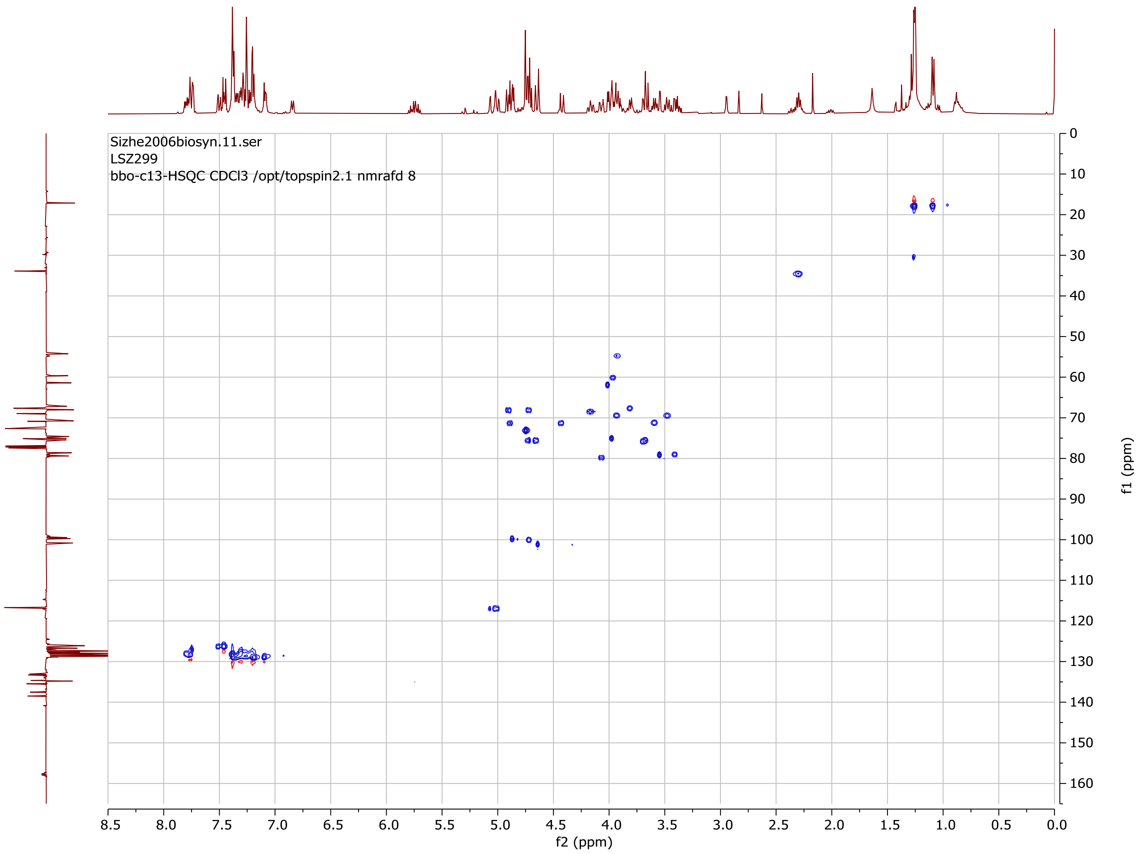

Hexasaccharide-protected with ONap on L-Fuc, Bn on D-Man and *N*-TCA (33)

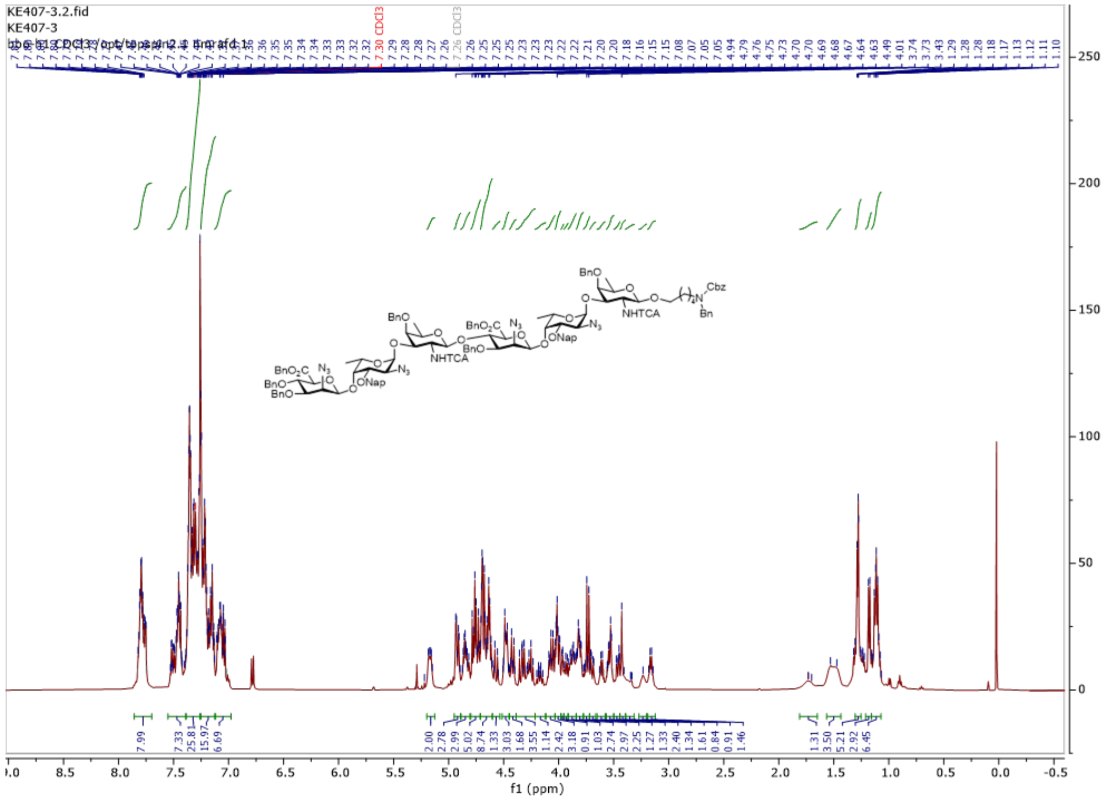

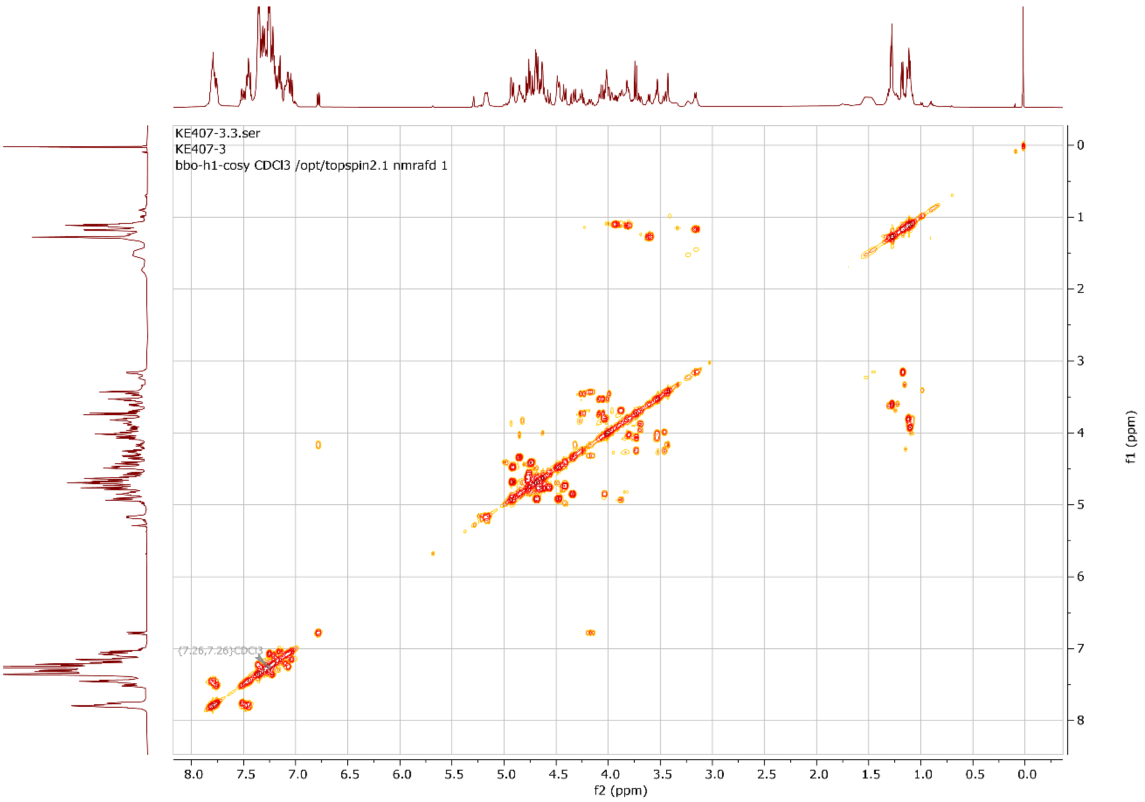
KE407-3.3.ser
KE407-3
bbo-h1-cosy CDCl3 /opt/topspin2.1 nmrafd 1
f2 (ppm)
f1 (ppm)

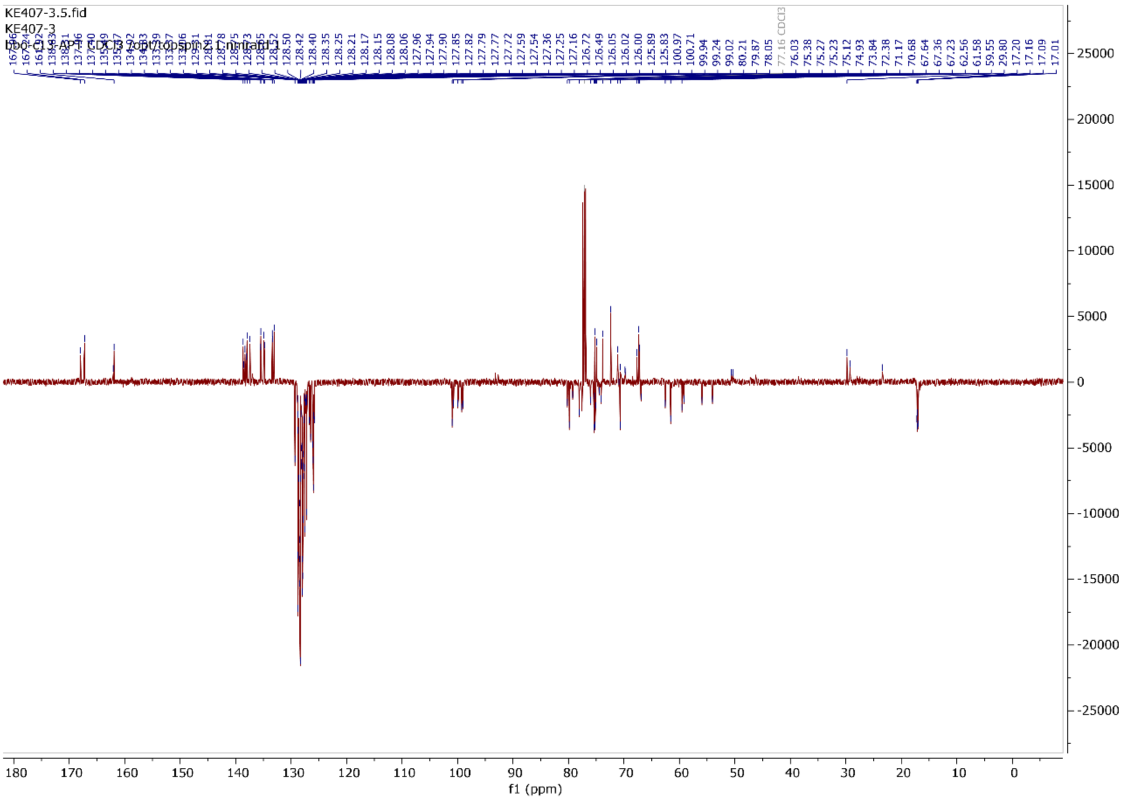
KE407-3.5.fid
KE407-3
f1 (ppm)

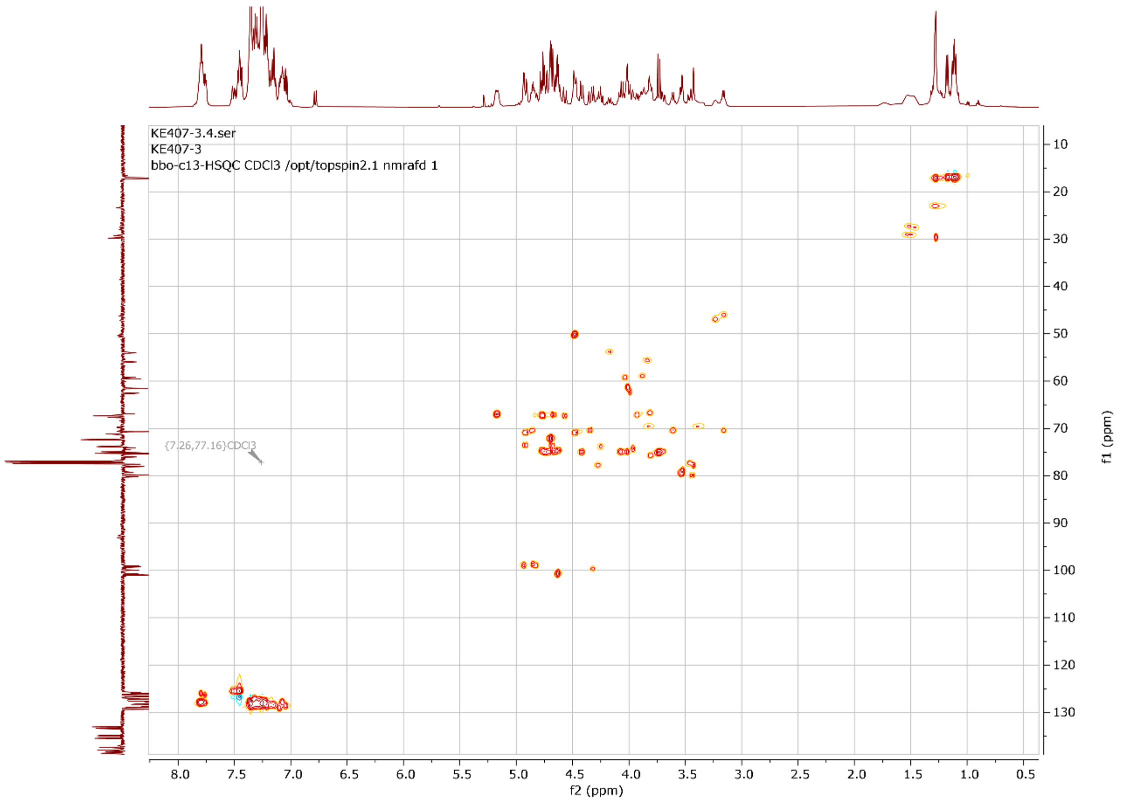
KE407-3.4.ser
KE407-3
bbo-c13-HSQC CDCl3 /opt/topspin2.1 nmrafd 1
{7.26,77.16}CDCl3
f2 (ppm)
f1 (ppm)

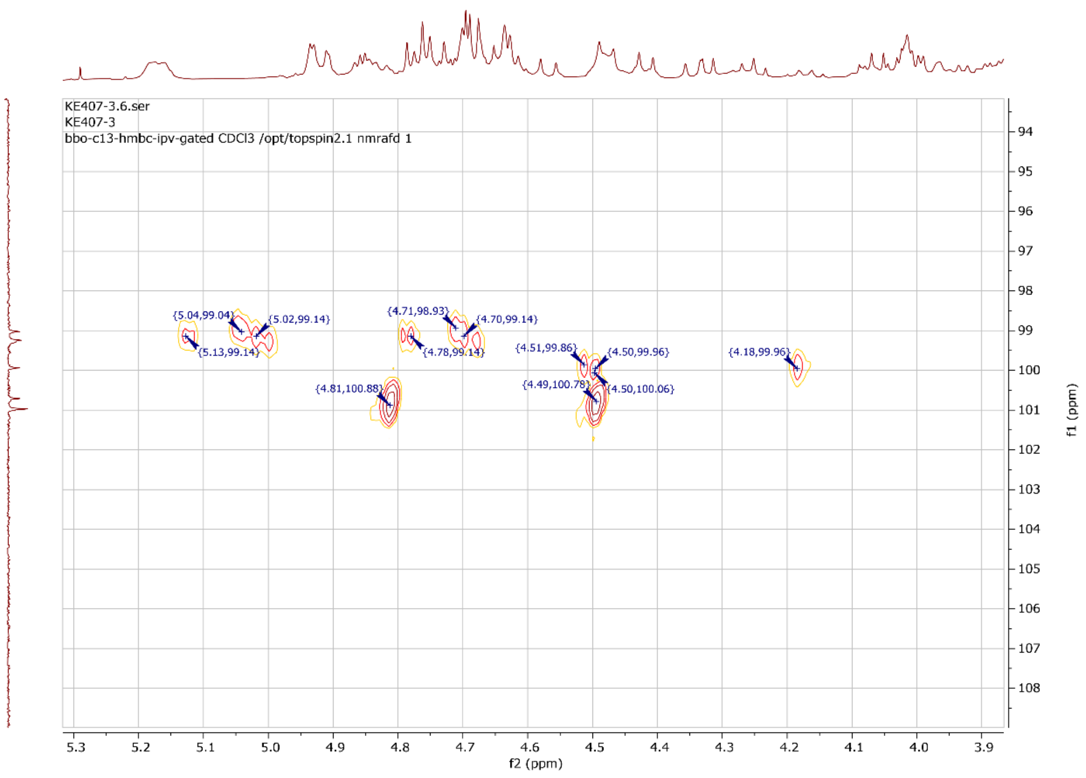
KE407-3.6.ser
KE407-3
bbo-c13-hmbc-ipv-gated CDCl3 /opt/topspin2.1 nmrafd 1
{5.04,99.04}
{5.02,99.14}
{5.13,99.14}
{4.71,98.93}
{4.70,99.14}
{4.78,99.14}
{4.81,100.88}
{4.51,99.86}
{4.50,99.96}
{4.49,100.78}
{4.50,100.06}
{4.18,99.96}
f2 (ppm)
f1 (ppm)

CP5-*O*-Ac-Hexasaccharide (2)

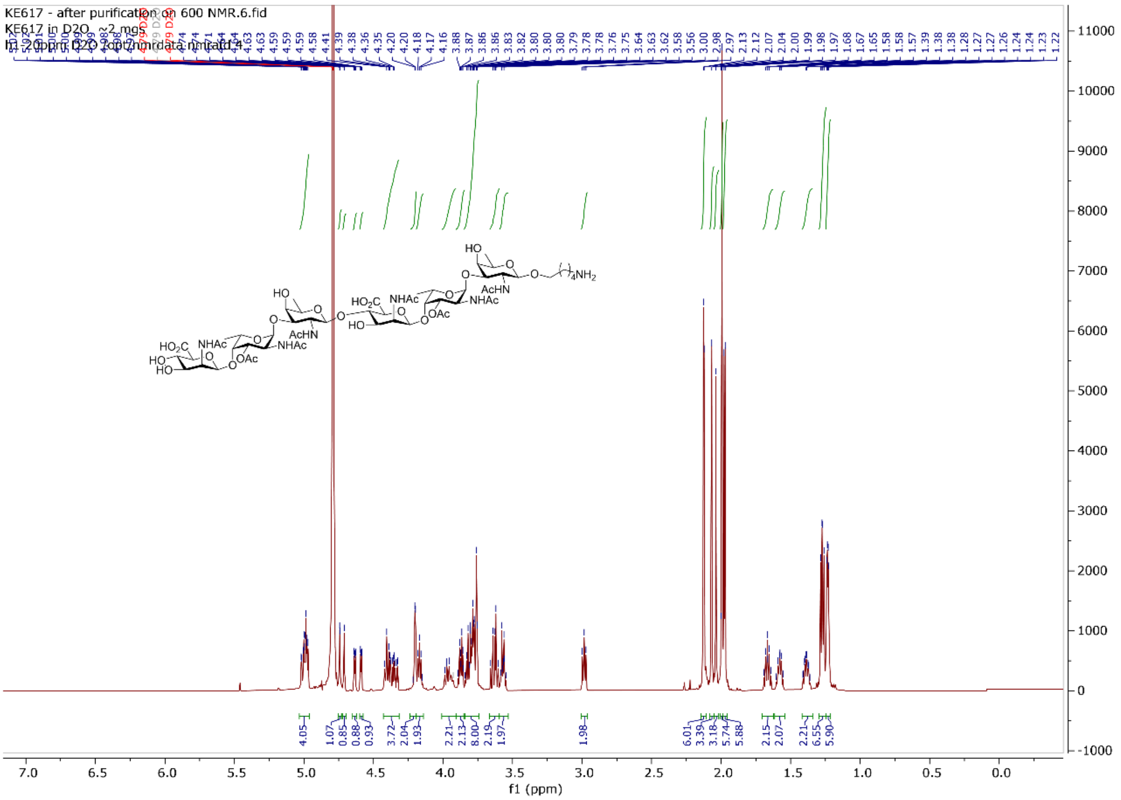

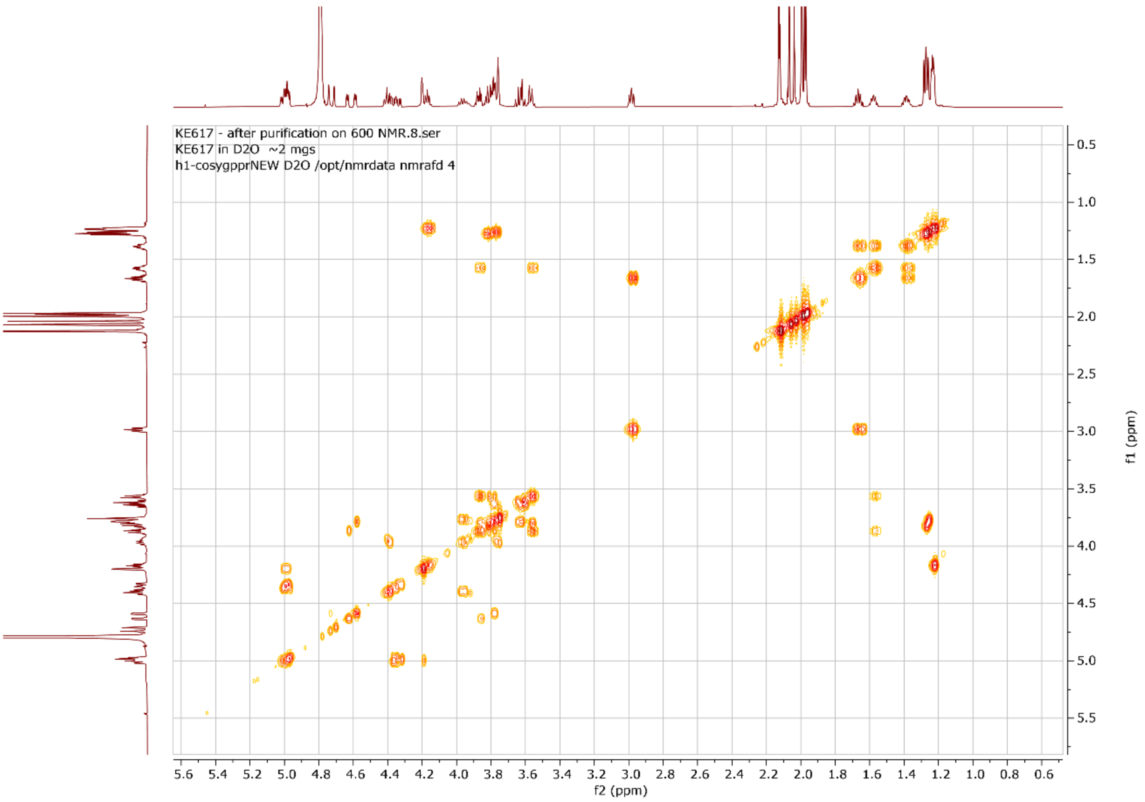

KE617 - after purification on 600 NMR.8.ser
KE617 in D2O ~2 mgs
h1-cosygpprNEW D2O /opt/nmrdata nmrafd 4
f2 (ppm)
f1 (ppm)

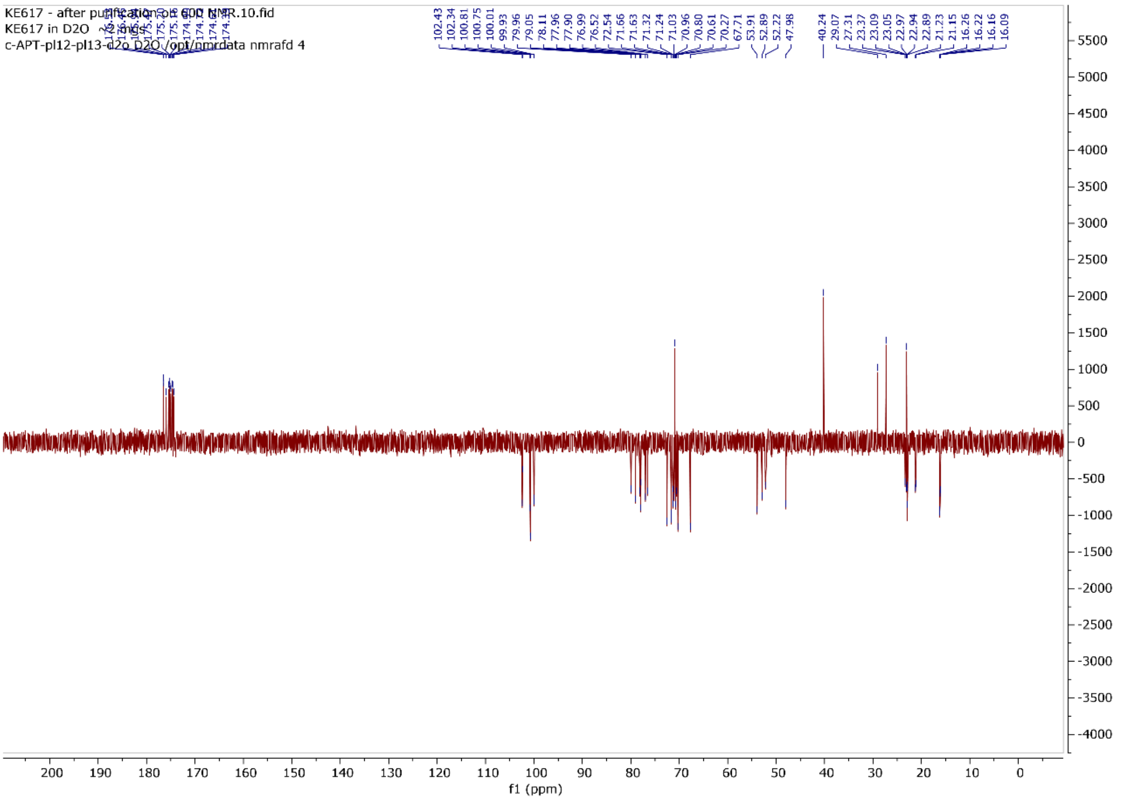

KE617 - after purification on 600 NMR.10.fid
KE617 in D2O ~2 mgs
c-APT-pl12-pl13-d2o D2O /opt/nmrdata nmrafd 4
102.43
102.34
100.81
100.75
100.01
99.93
79.96
79.05
78.11
77.96
77.90
76.99
76.52
72.54
71.66
71.63
71.32
71.24
71.03
70.96
70.80
70.61
70.27
67.71
53.91
52.89
52.22
47.98
40.24
29.07
27.31
23.37
23.09
23.05
22.97
22.94
22.89
21.23
21.15
16.26
16.22
16.16
16.09
f1 (ppm)

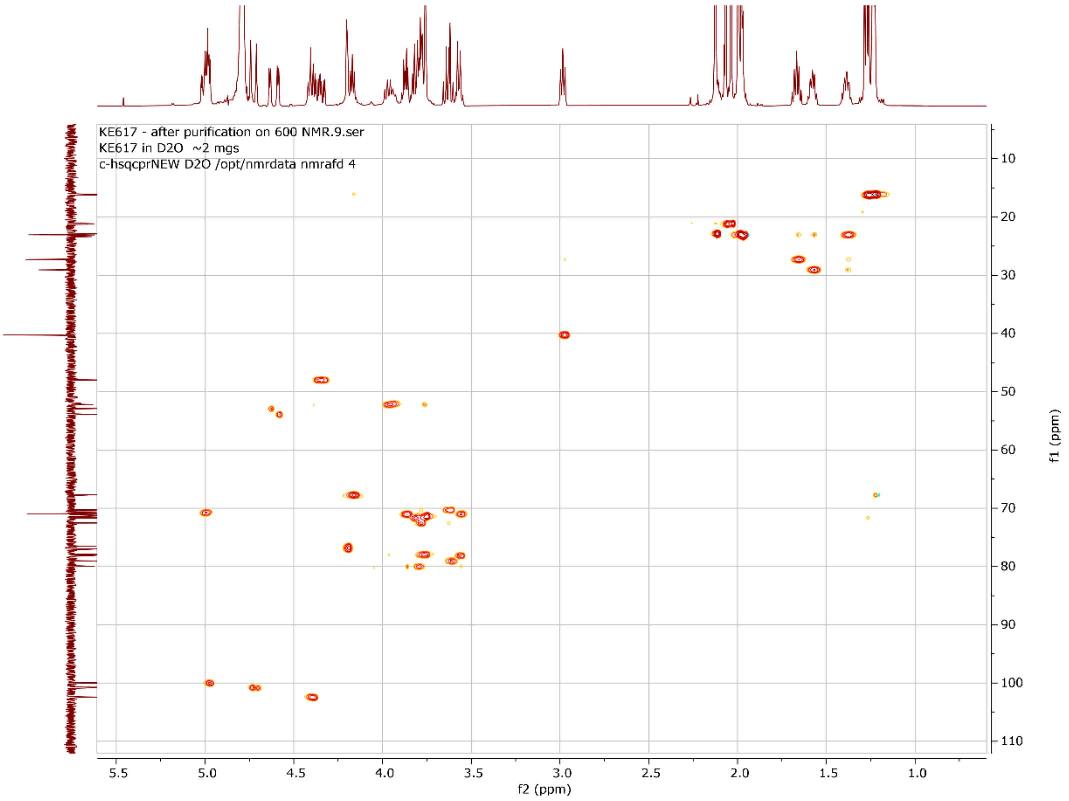

Hexasaccharide-protected with ONap on L-Fuc, PMB on D-Man and *N*-TCA (34)

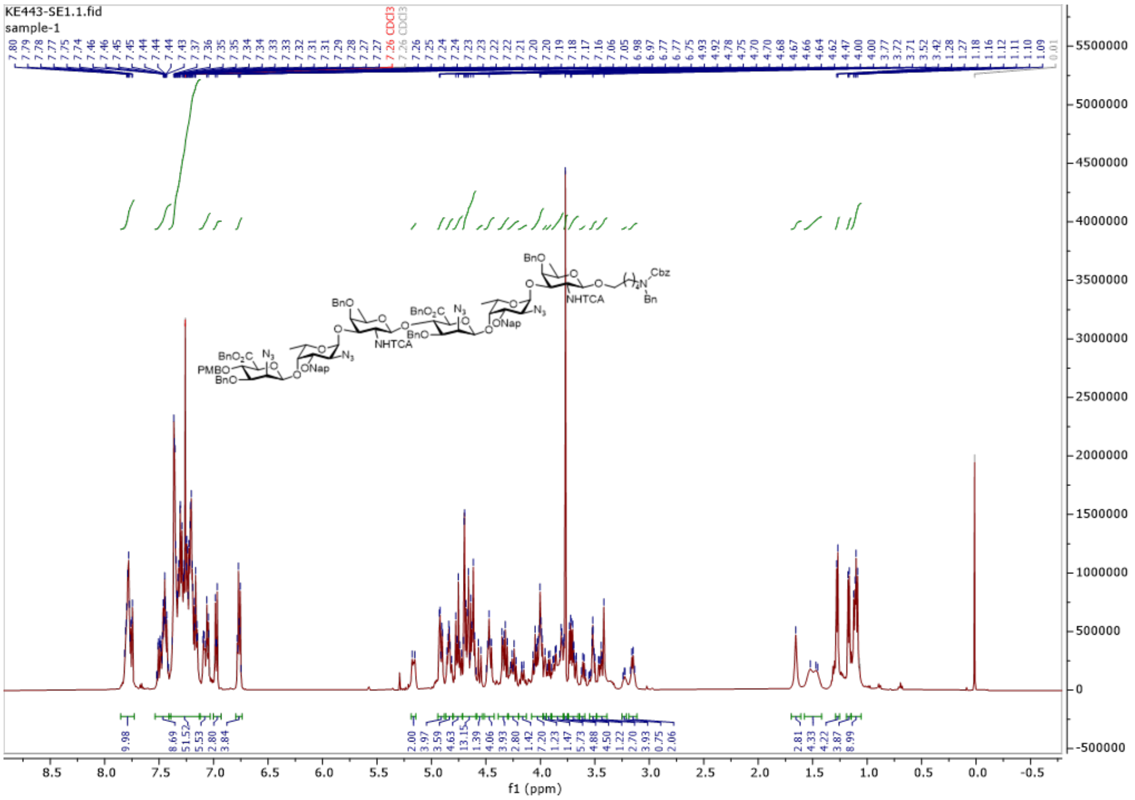
KE443-SE1.1.fid
sample-1
f1 (ppm)

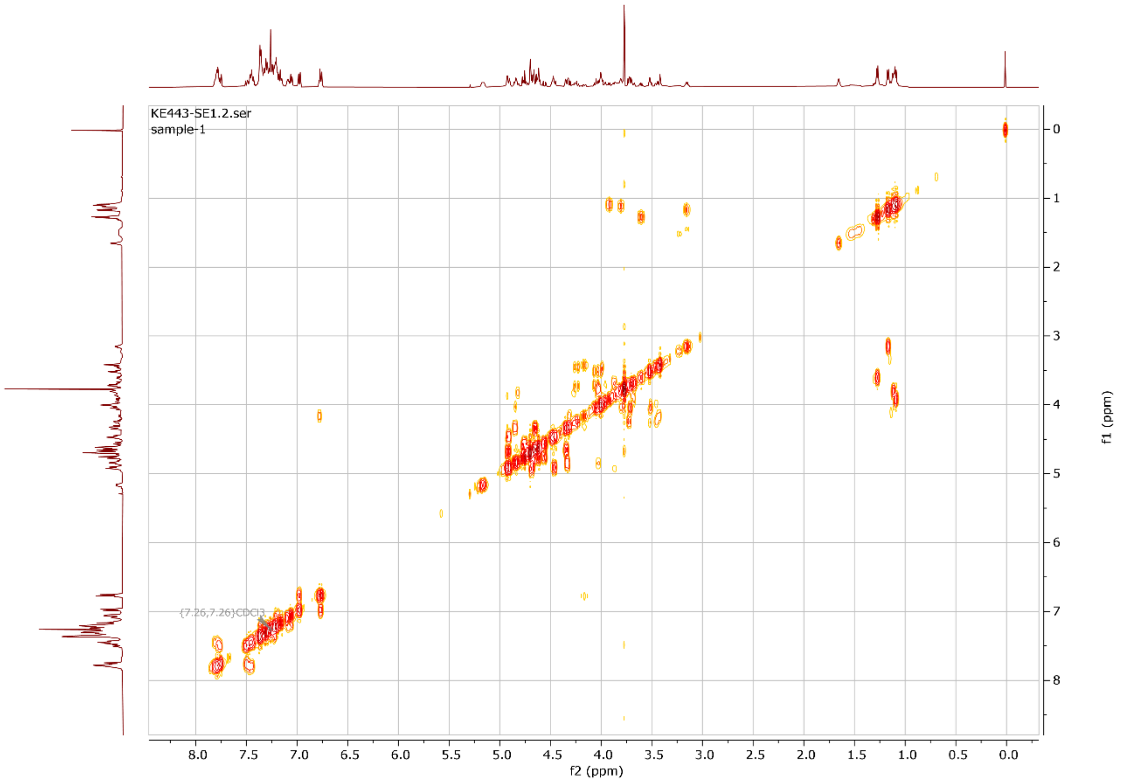
KE443-SE1.2.ser
sample-1
(7.26,7.26)CDCl3
f2 (ppm)
f1 (ppm)

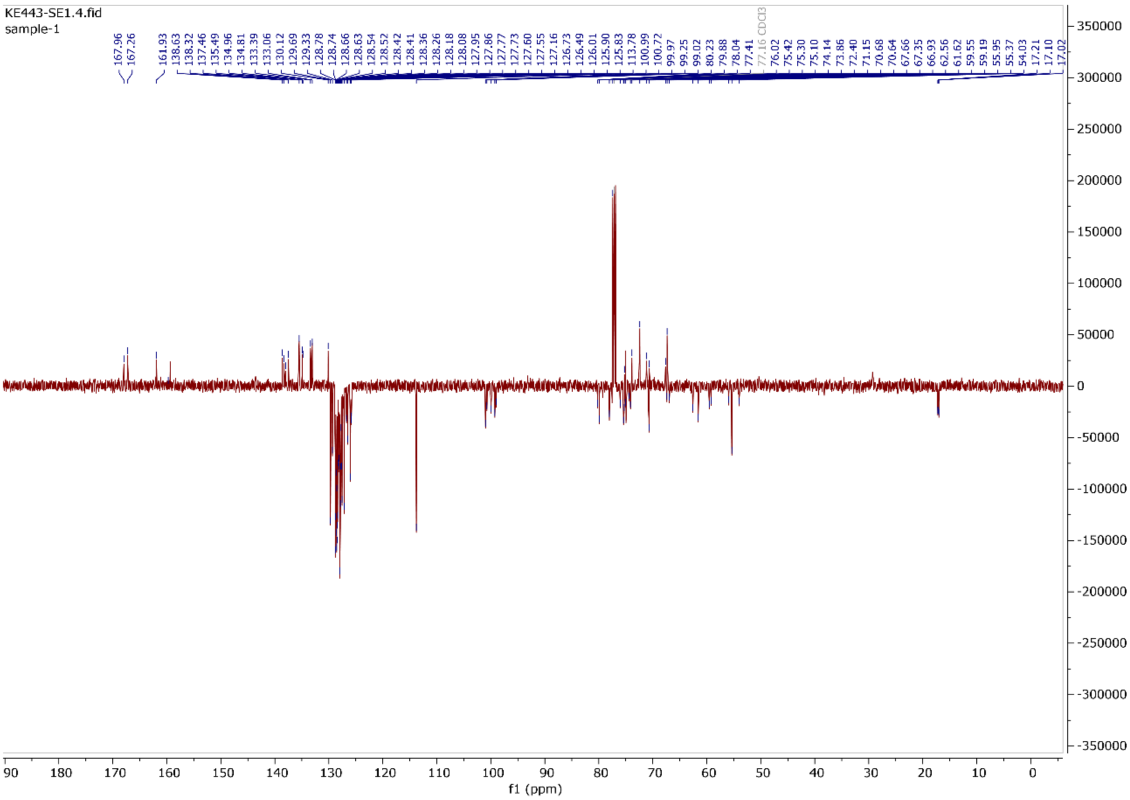
KE443-SE1.4.fid
sample-1
f1 (ppm)

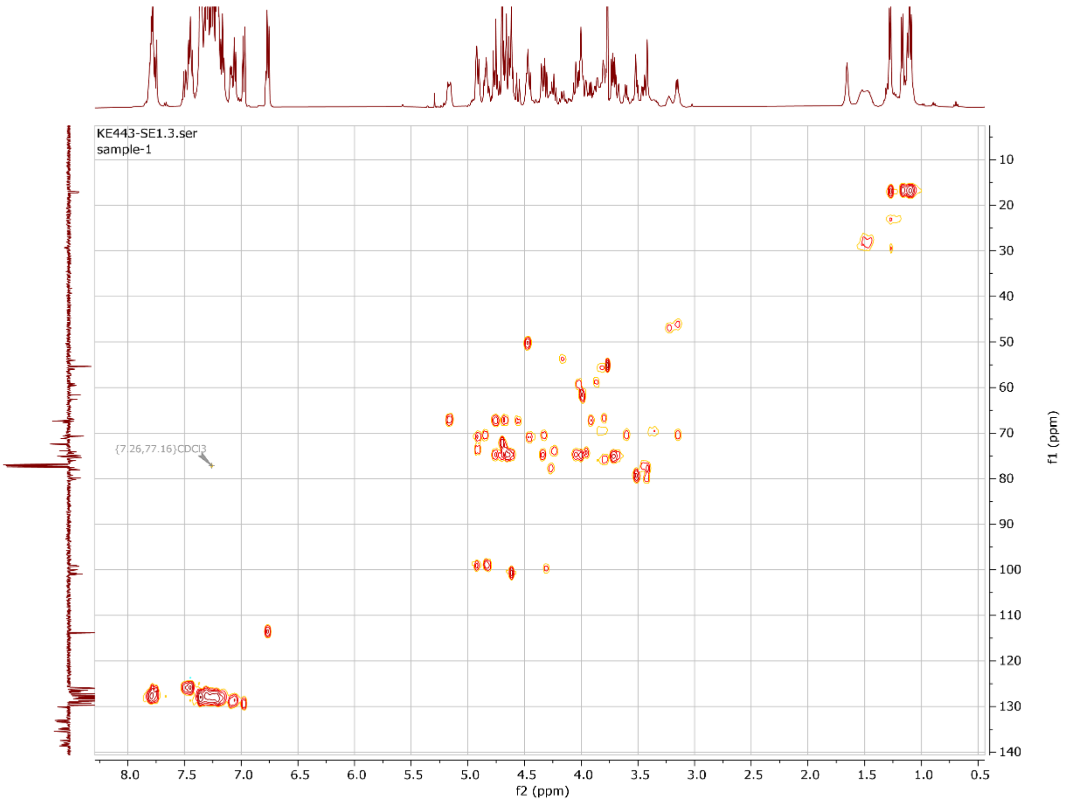
KE443-SE1.3.ser
sample-1
f2 (ppm)
f1 (ppm)

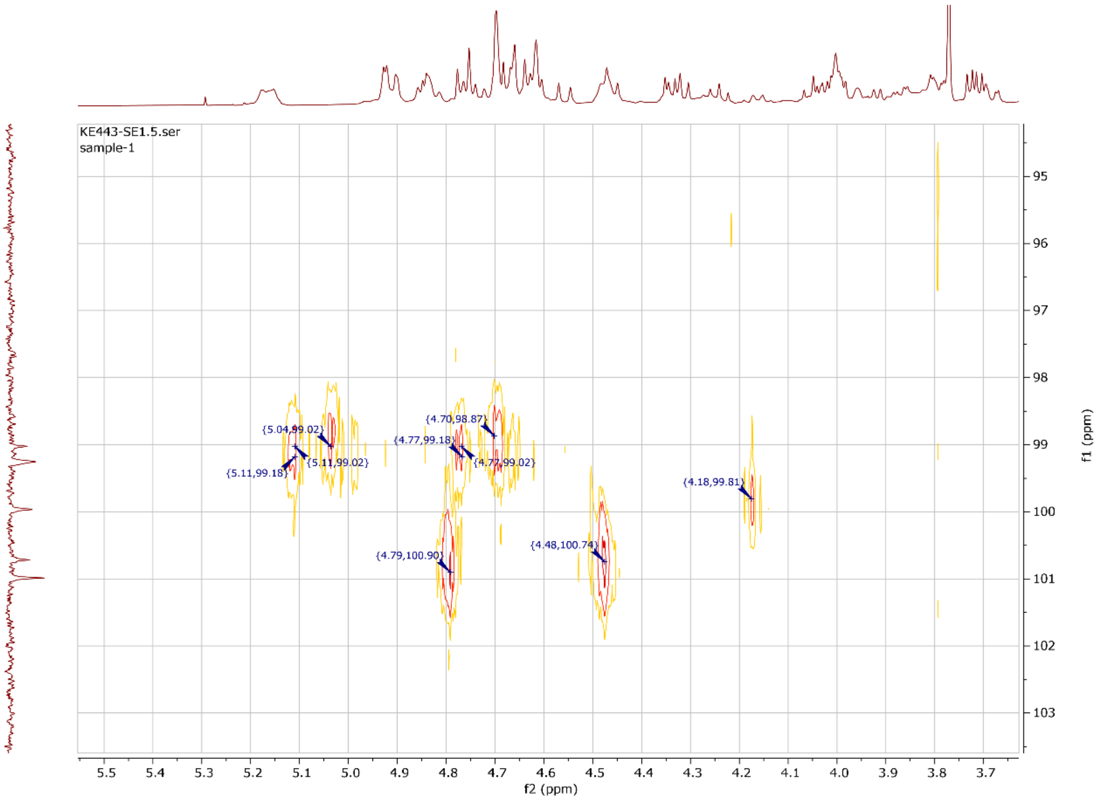

Hexasaccharide-protected with ONap on L-Fuc, PMB on D-Man and *N*-TFA (36)

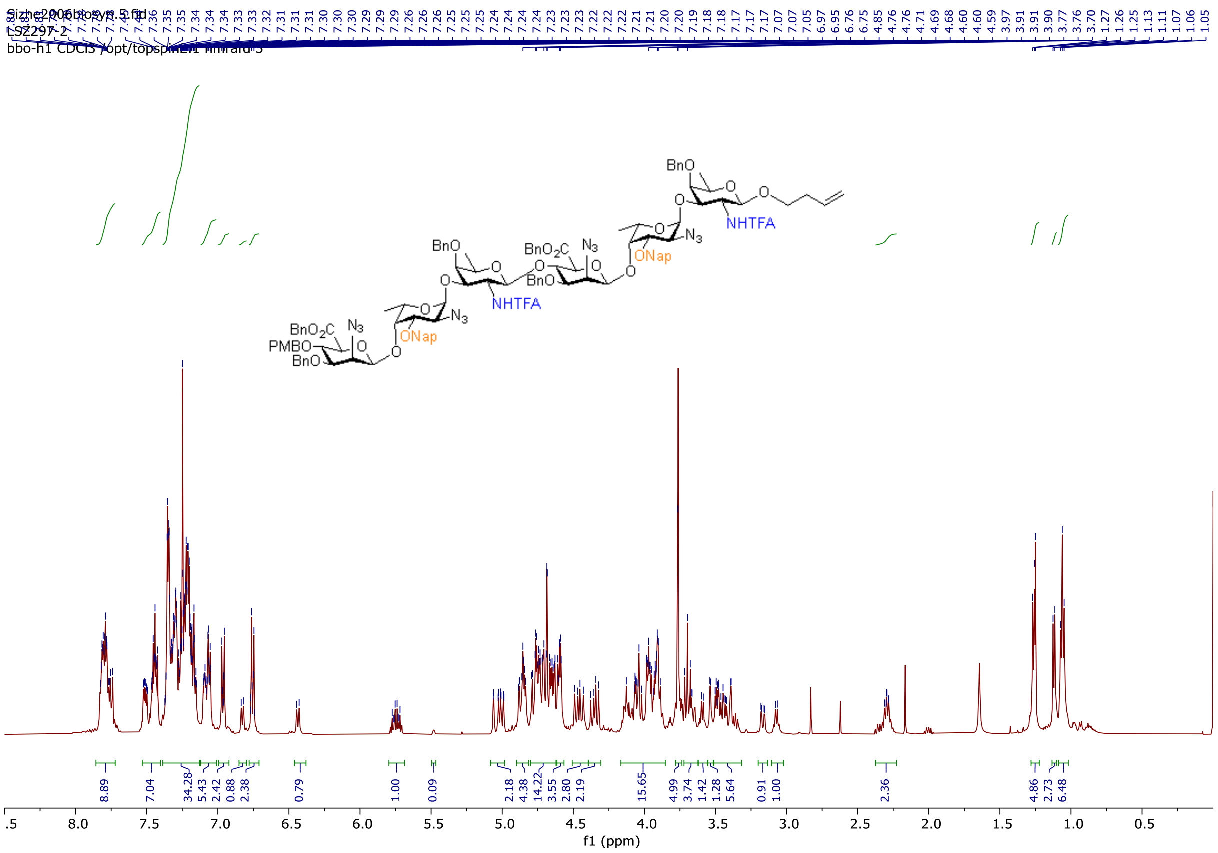

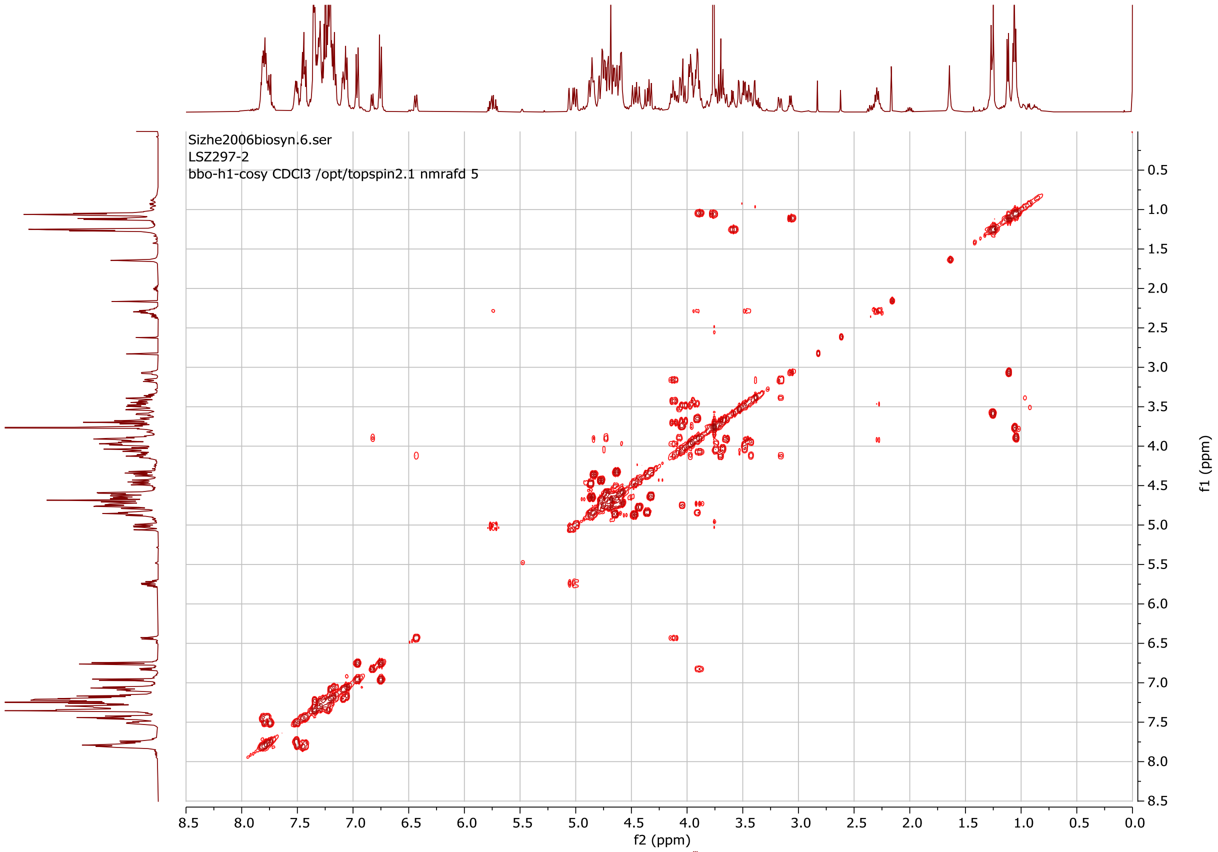
Sizhe2006biosyn.6.ser
LSZ297-2
bbo-h1-cosy CDCl3 /opt/topspin2.1 nmrafd 5
f2 (ppm)
f1 (ppm)

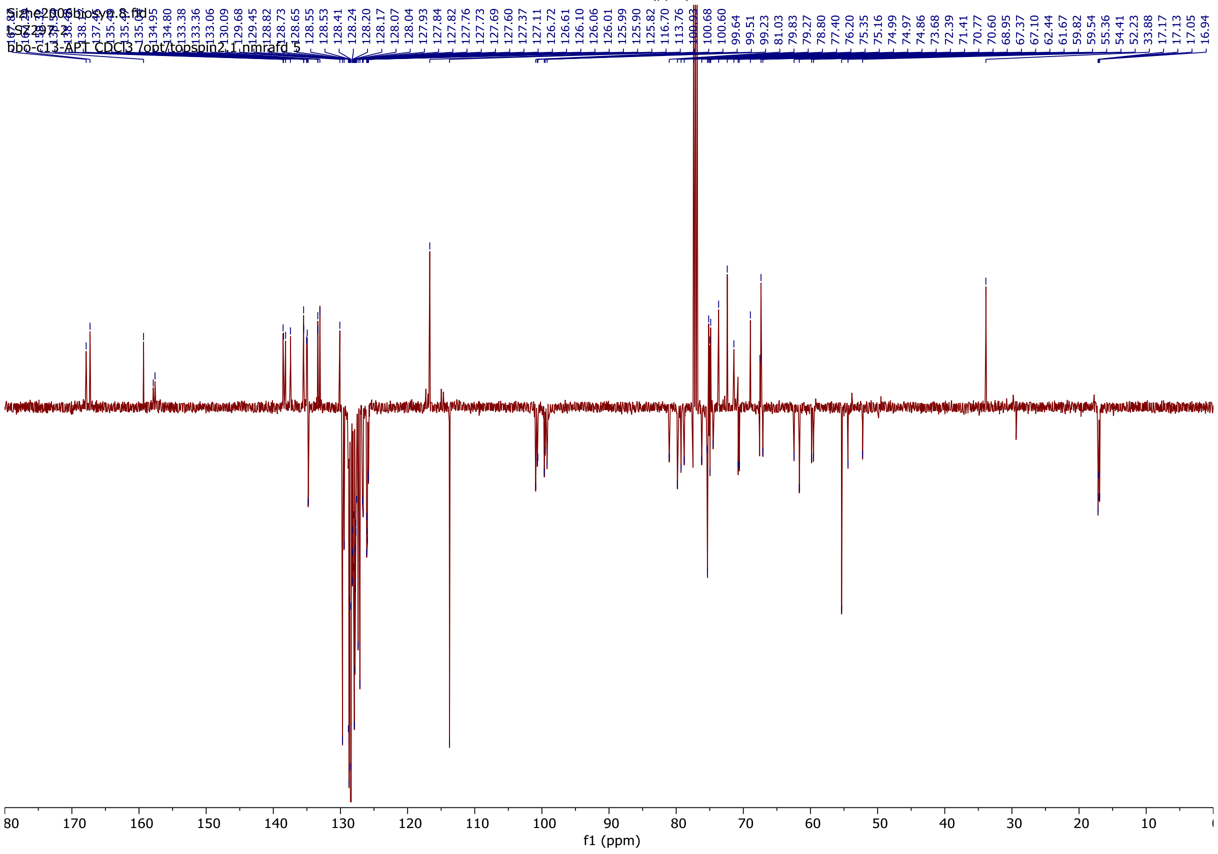
LSZ297-2
bbo-c13-APT CDCl3 /opt/topspin2.1 nmrafd 5
f1 (ppm)

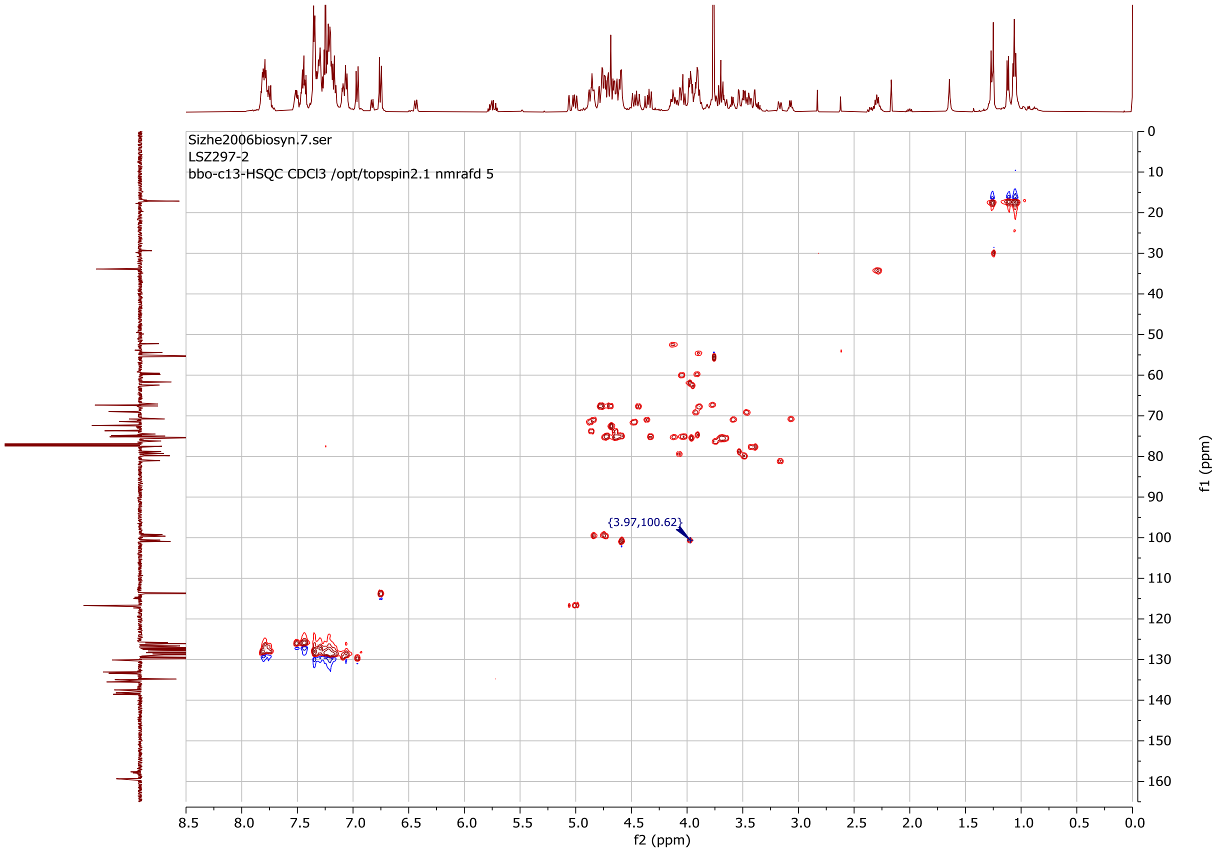

Hexasaccharide-protected with ONap on L-Fuc and *N*-TCA as acceptor (35)

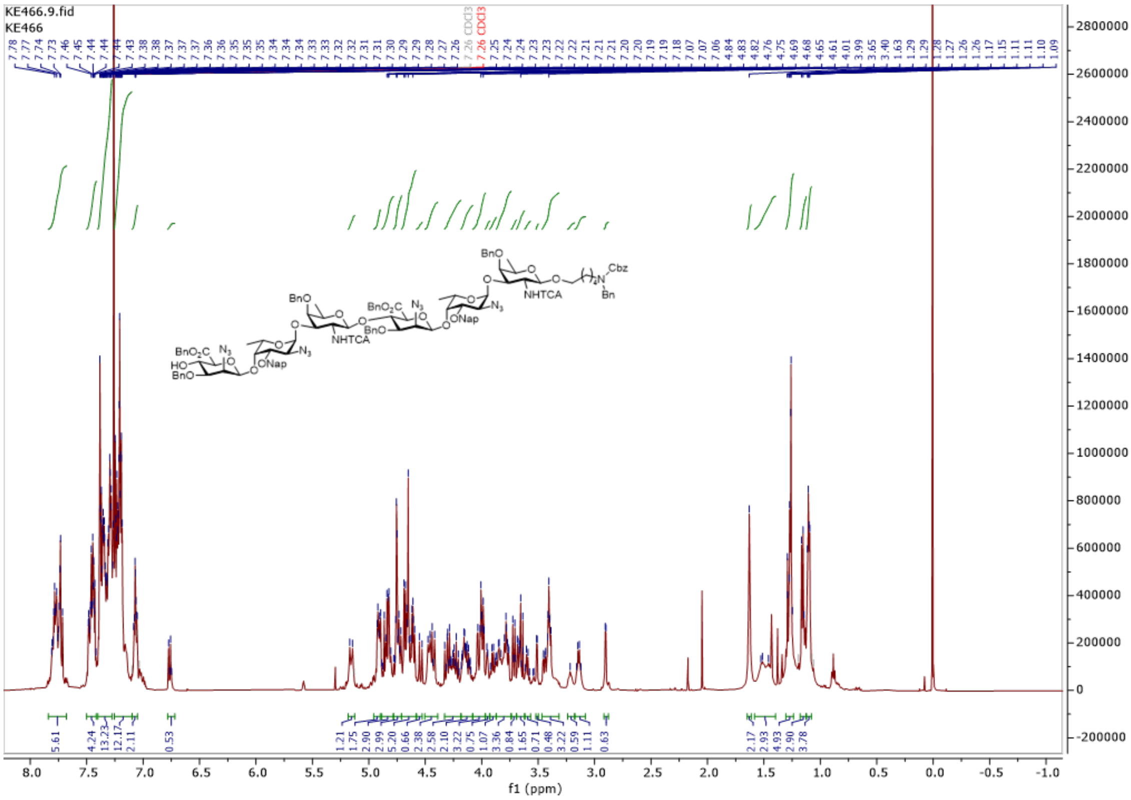
KE466.9.fid
KE466
BnO
NHTCA
Cbz
Bn
BnO₂C
N₃
ONap
HO
f1 (ppm)

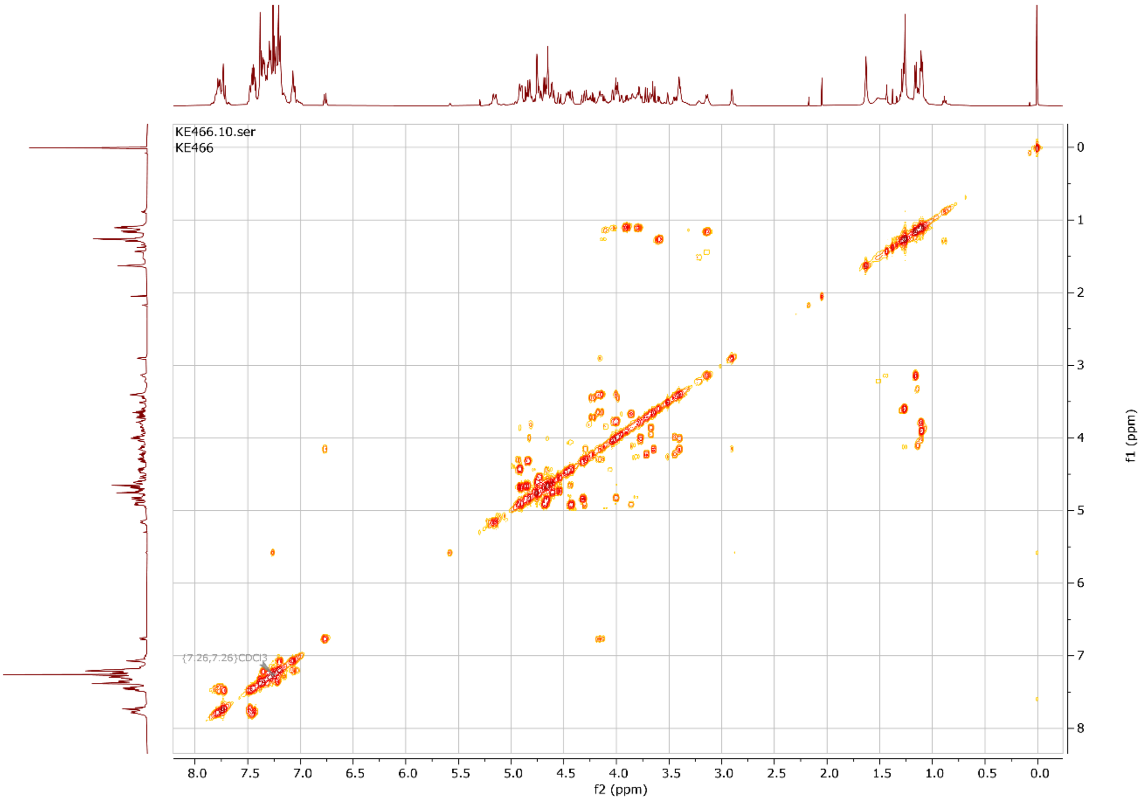
KE466.10.ser
KE466
f2 (ppm)
f1 (ppm)

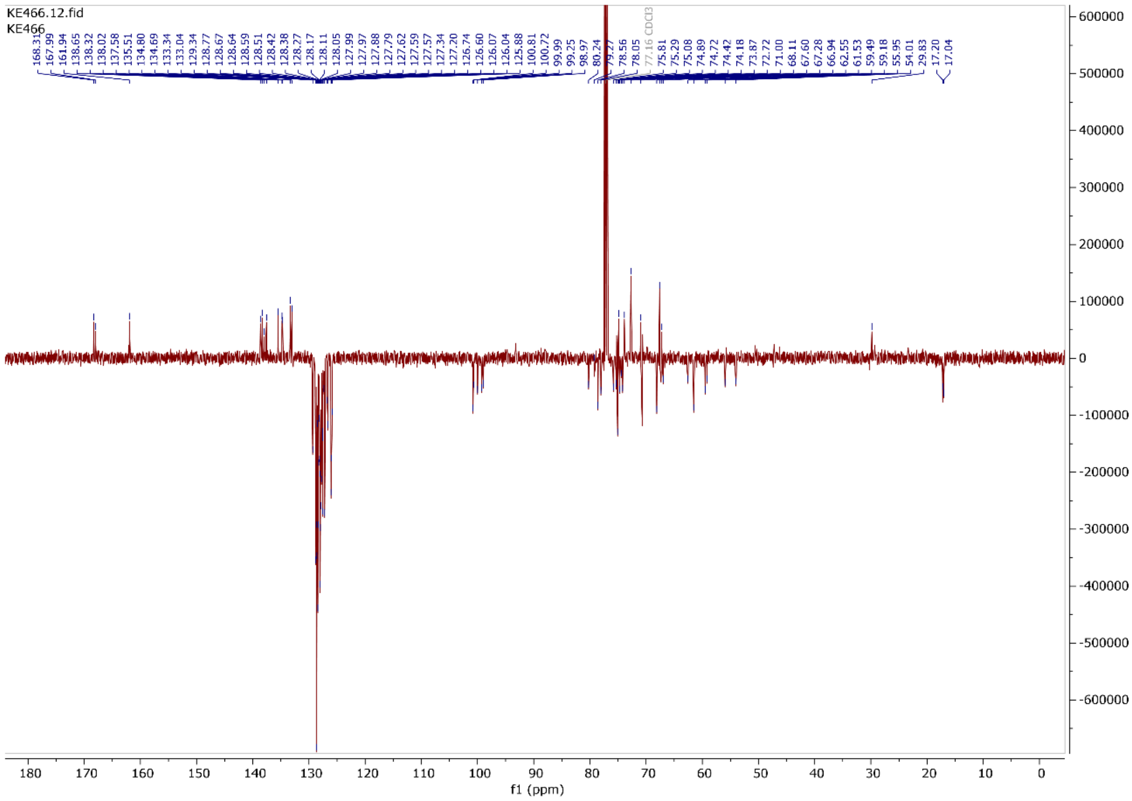

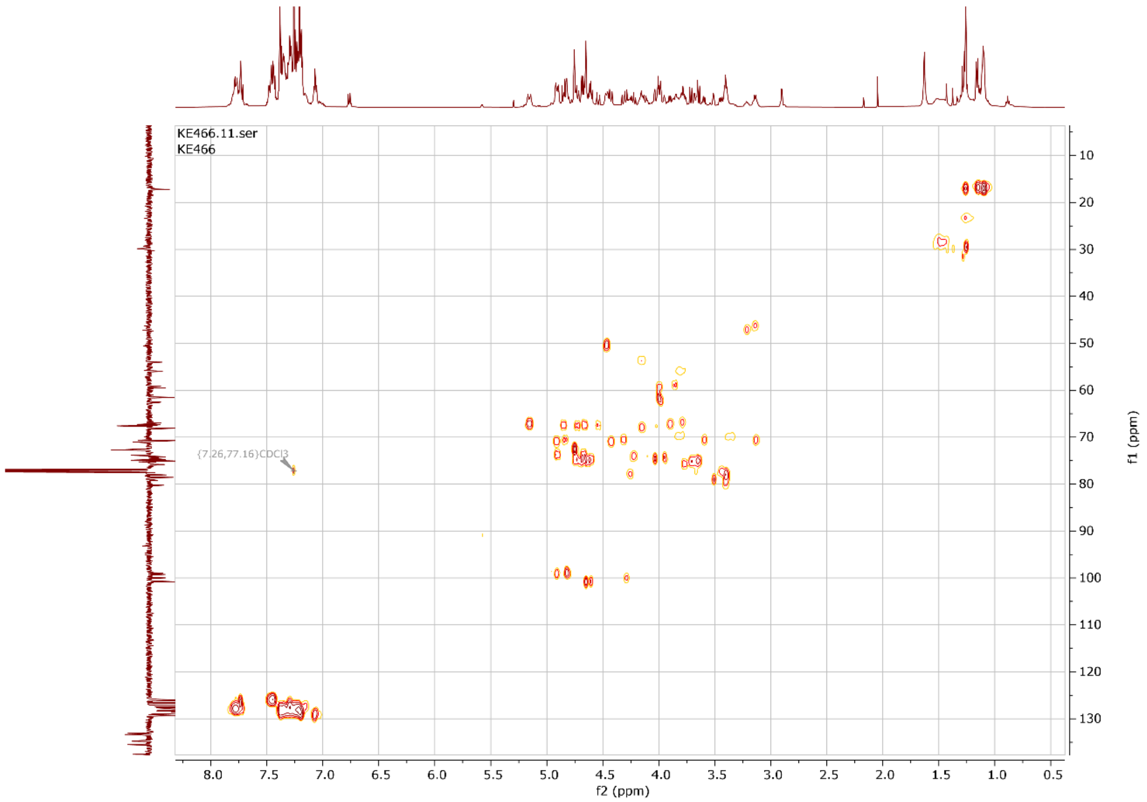

Hexasaccharide-protected with ONap on L-Fuc and *N*-TFA as acceptor (37)

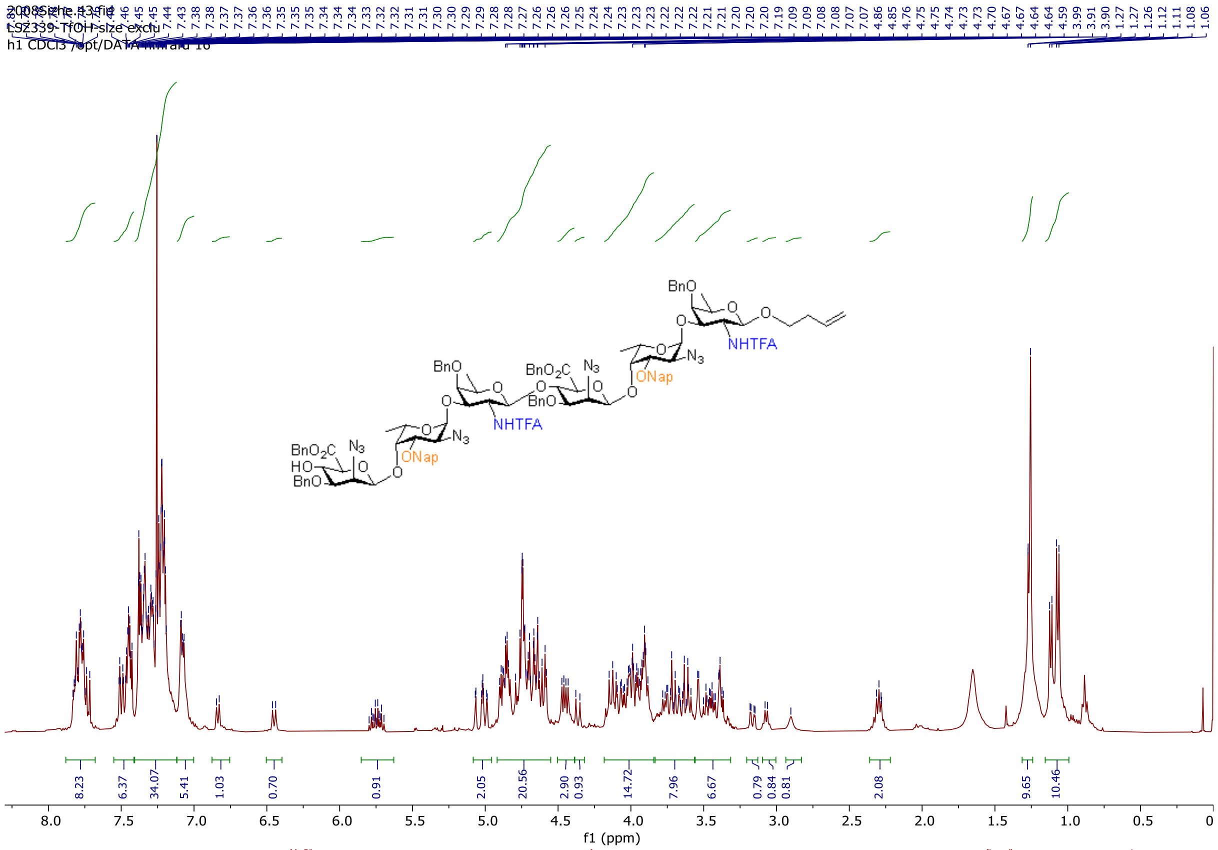

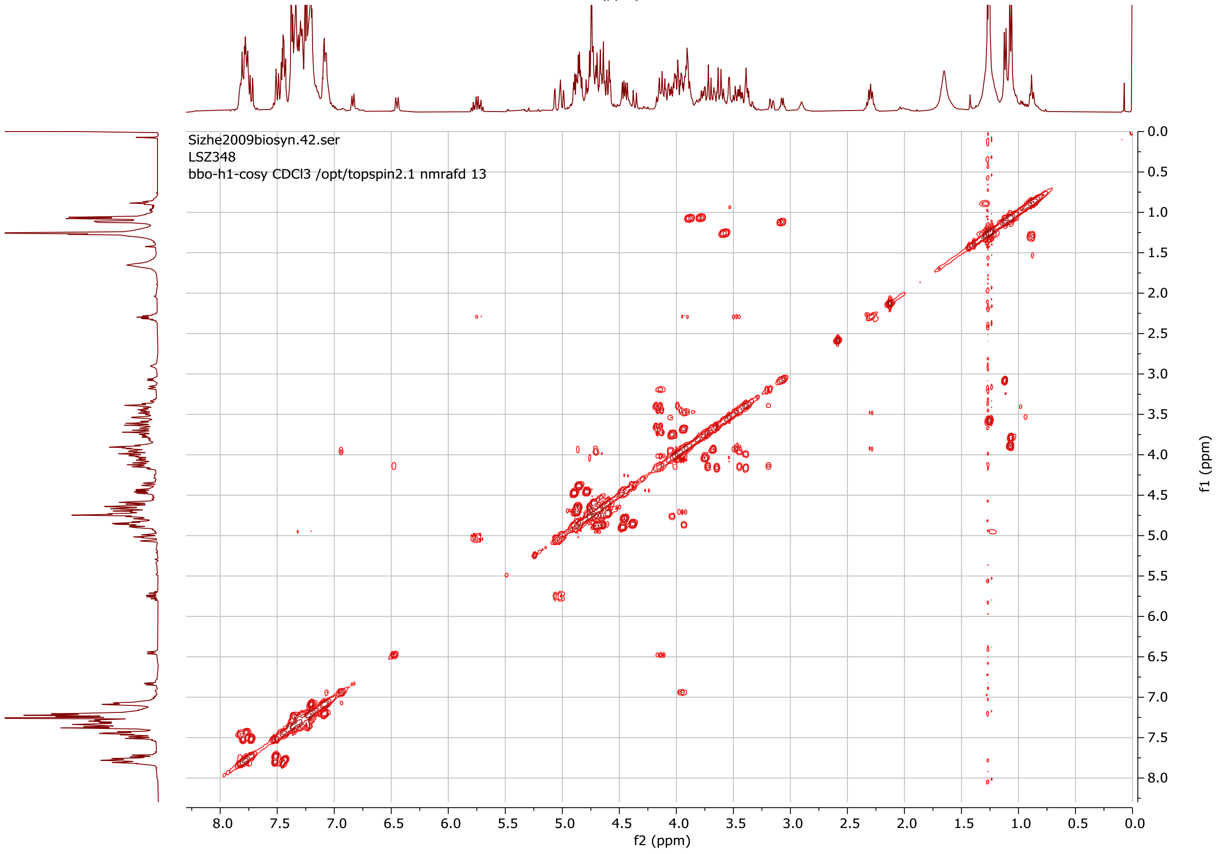
Sizhe2009biosyn.42.ser
LSZ348
bbo-h1-cosy CDCl3 /opt/topspin2.1 nmrafd 13
f2 (ppm)
f1 (ppm)

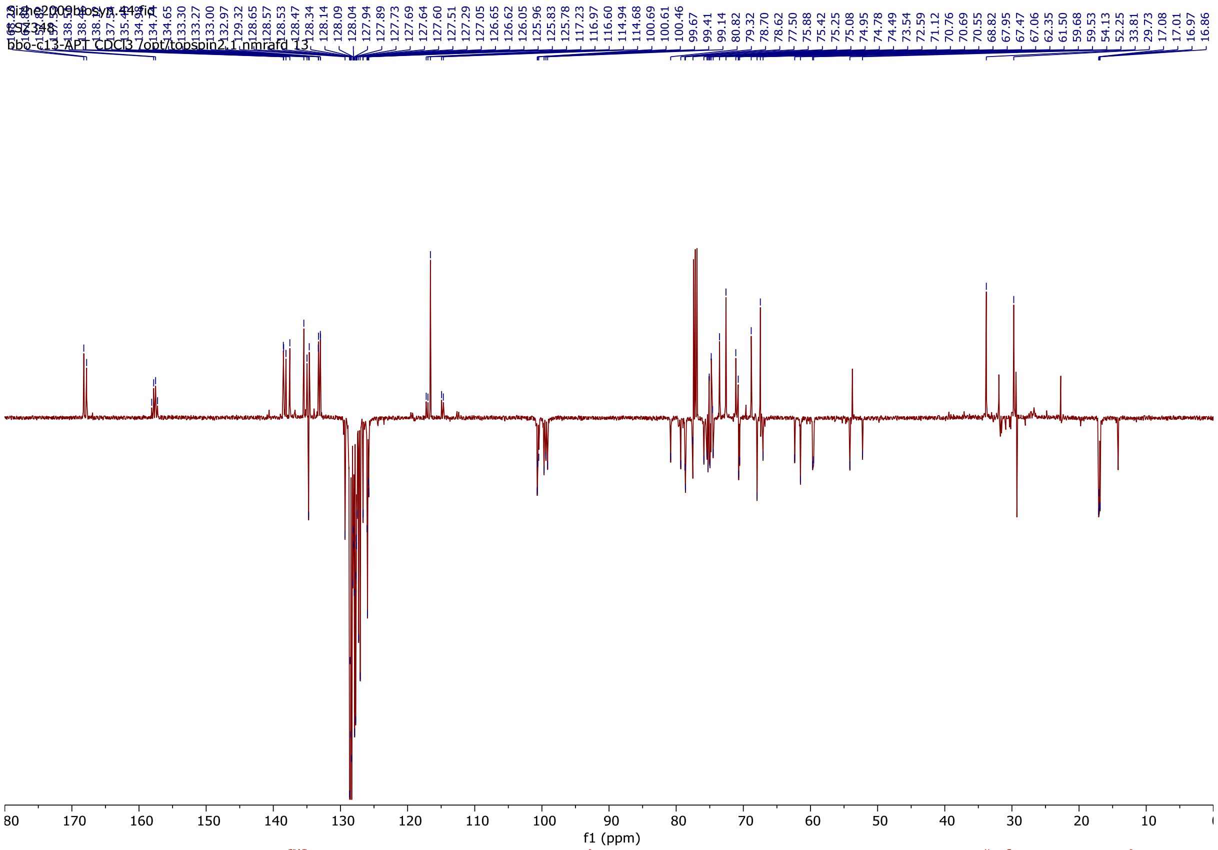

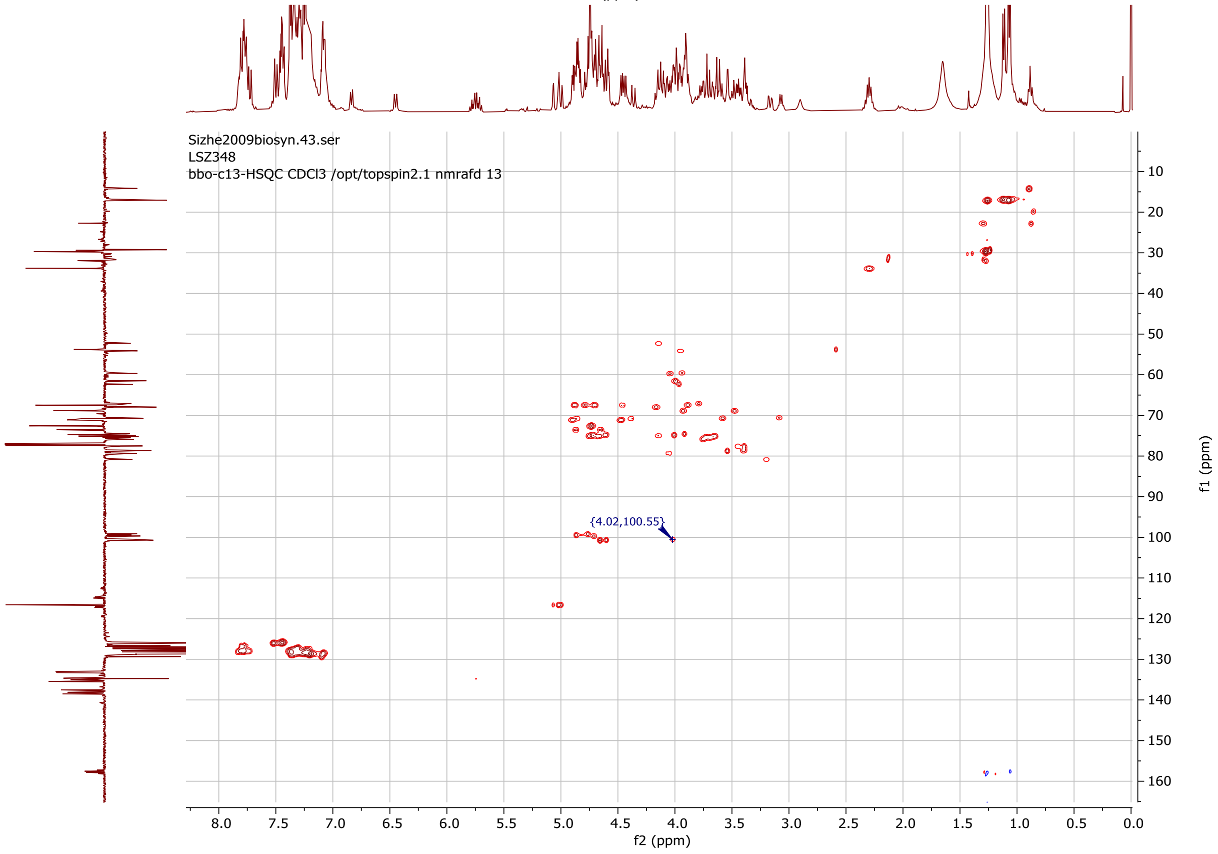

Nonasaccharide-protected with ONap on L-Fuc, Bn on D-Man and *N*-TCA (38)

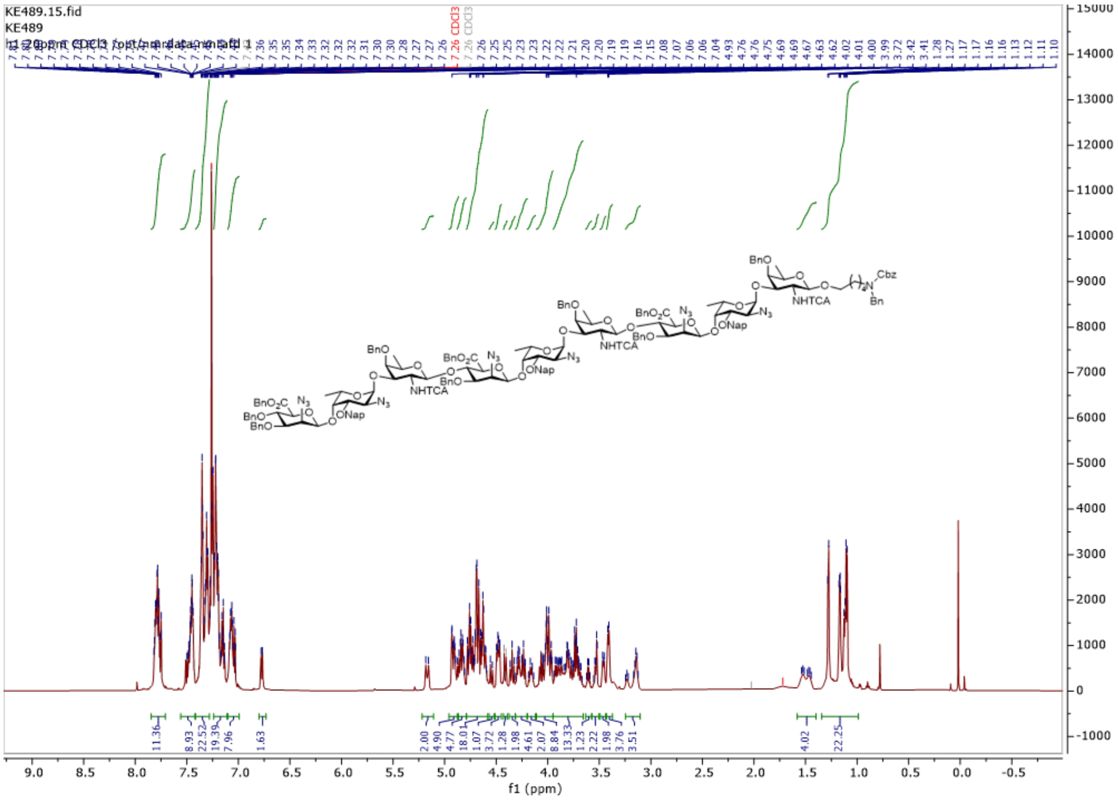
KE489.15.fid
KE489
h1 20ppm CDCl3 /opt/nmrdata nmrafd 1
BnO
NHTCA
Cbz
Bn
BnO$_2$C
N_3
ONap
f1 (ppm)

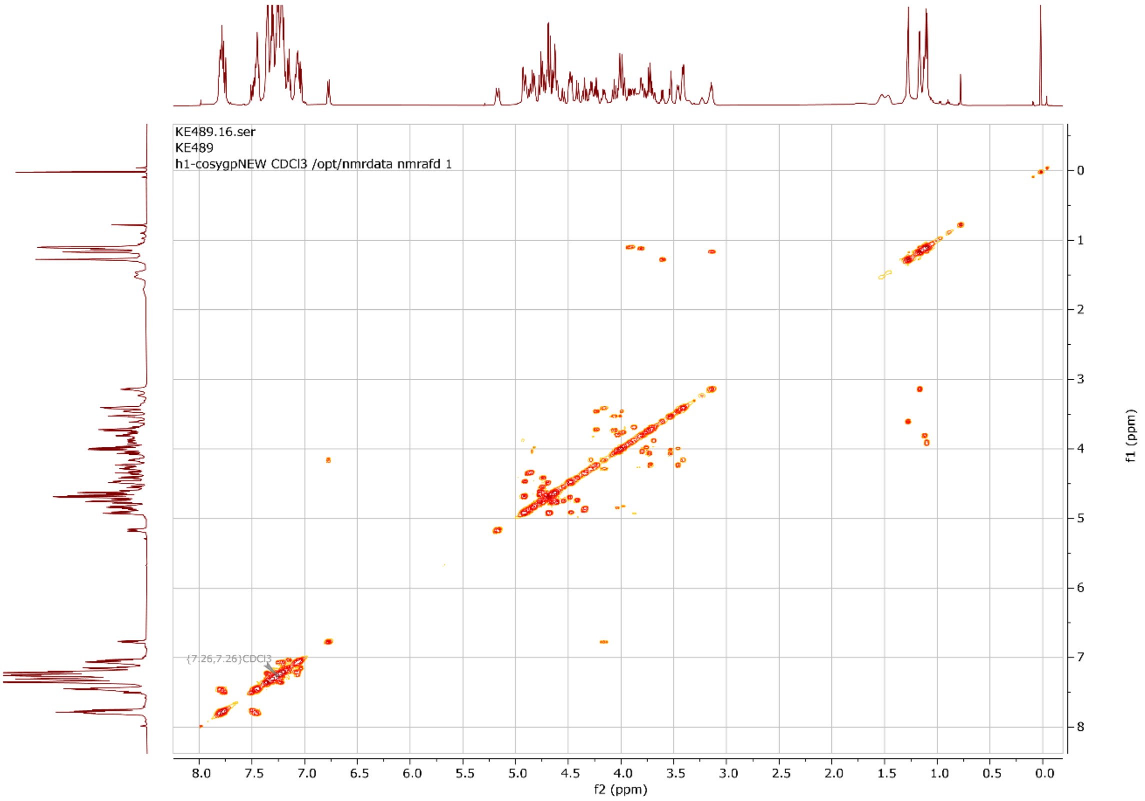
KE489.16.ser
KE489
h1-cosygpNEW CDCl3 /opt/nmrdata nmrafd 1
f2 (ppm)
f1 (ppm)

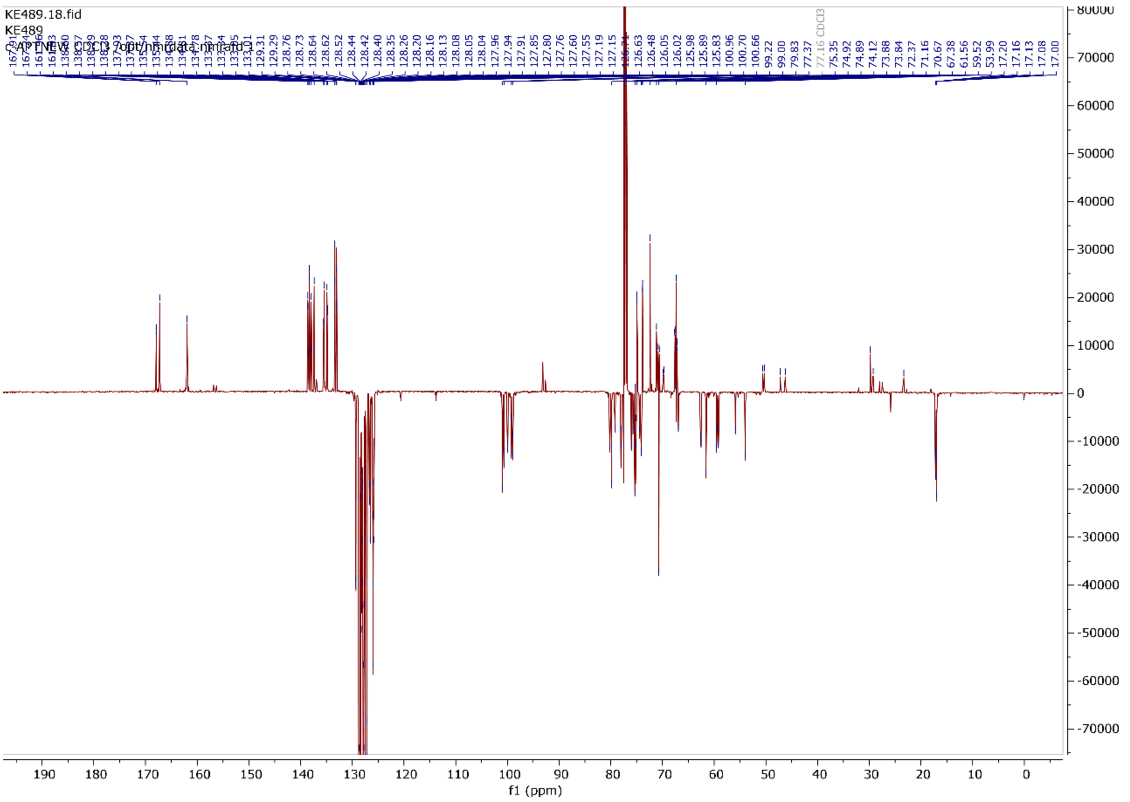

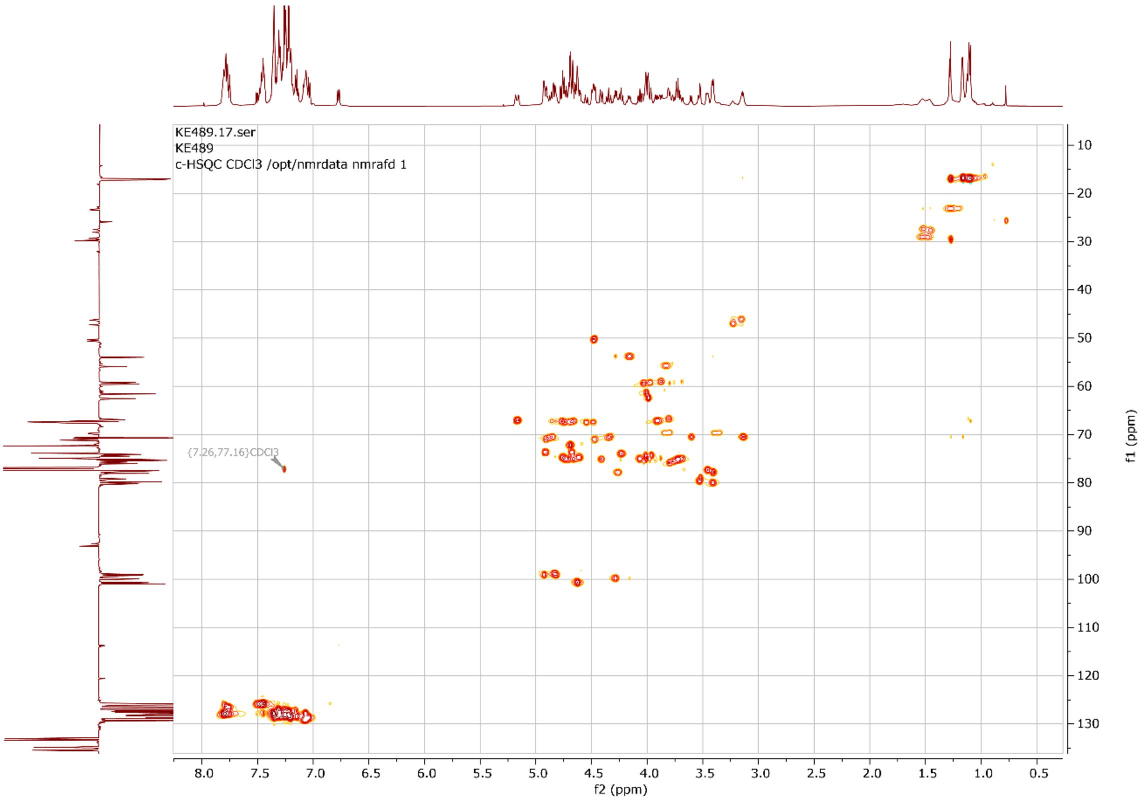

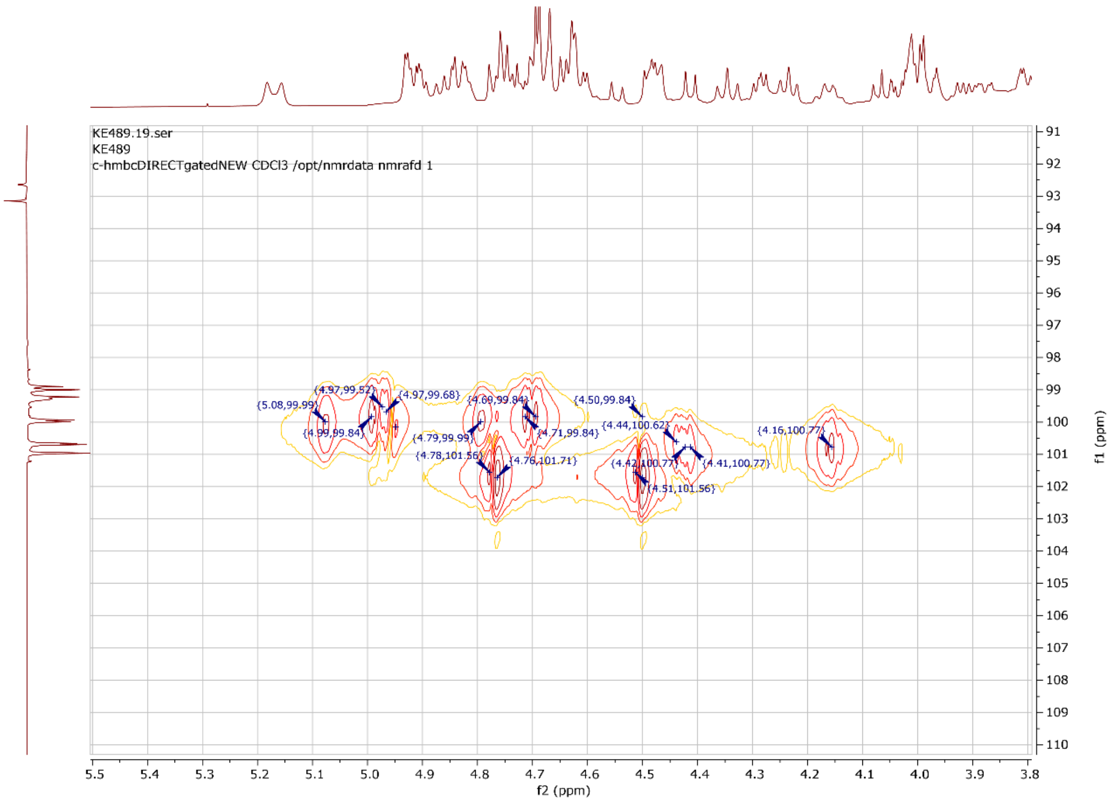

Nonasaccharide-protected with ONap on L-Fuc, Bn on D-Man and *N*-TFA (39)

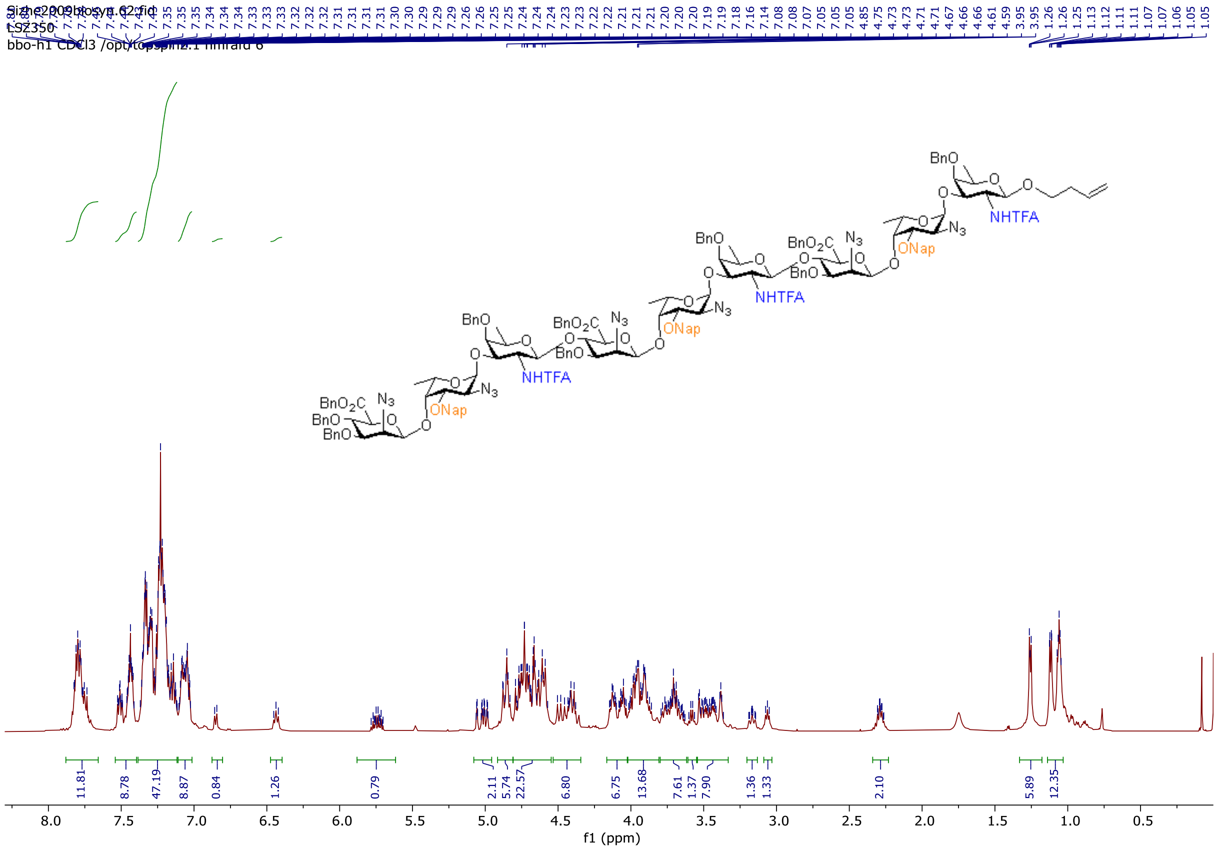
Sizhe2009biosyn.62.fid
LSZ350
bbo-h1 CDCl3 /opt/topspin2.1 nmrafd 6
BnO
NHTFA
ONap
BnO$_2$C
N$_3$
f1 (ppm)

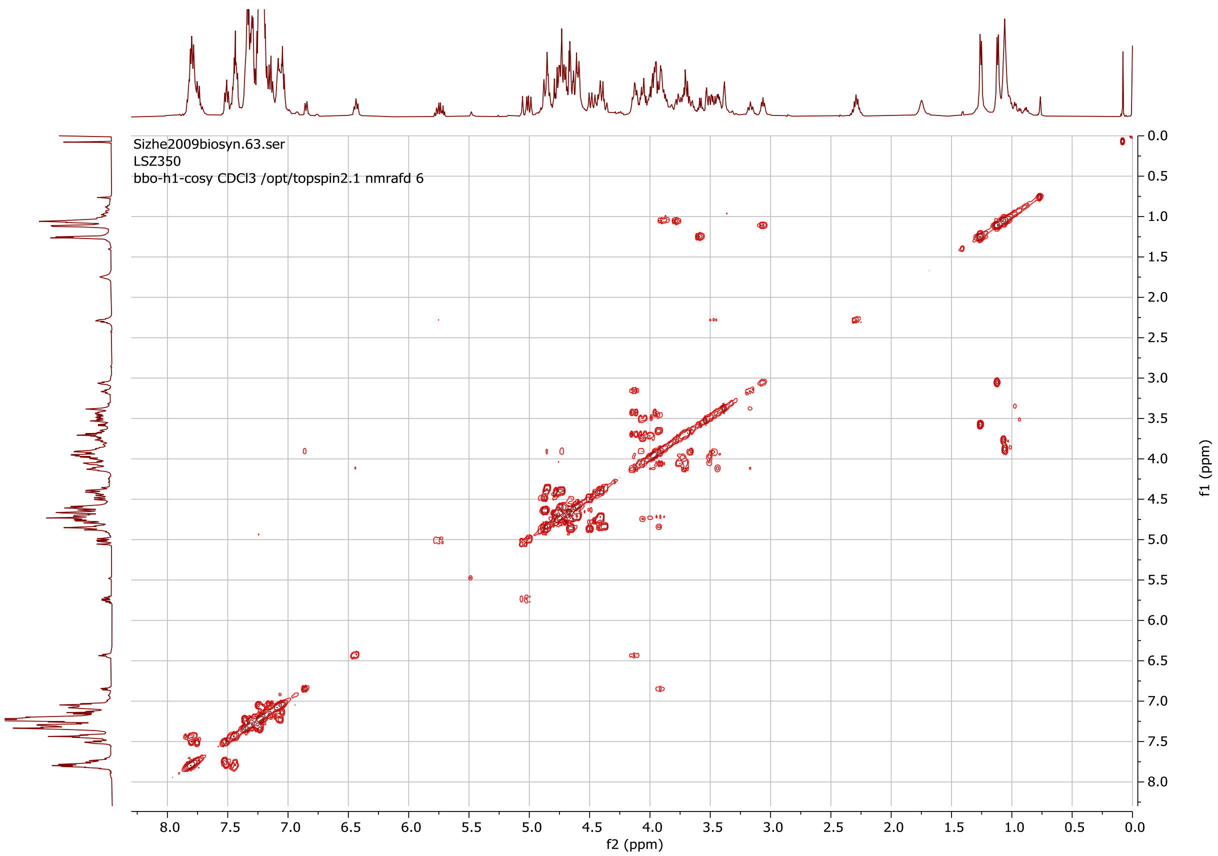
Sizhe2009biosyn.63.ser
LSZ350
bbo-h1-cosy CDCl3 /opt/topspin2.1 nmrafd 6
f2 (ppm)
f1 (ppm)

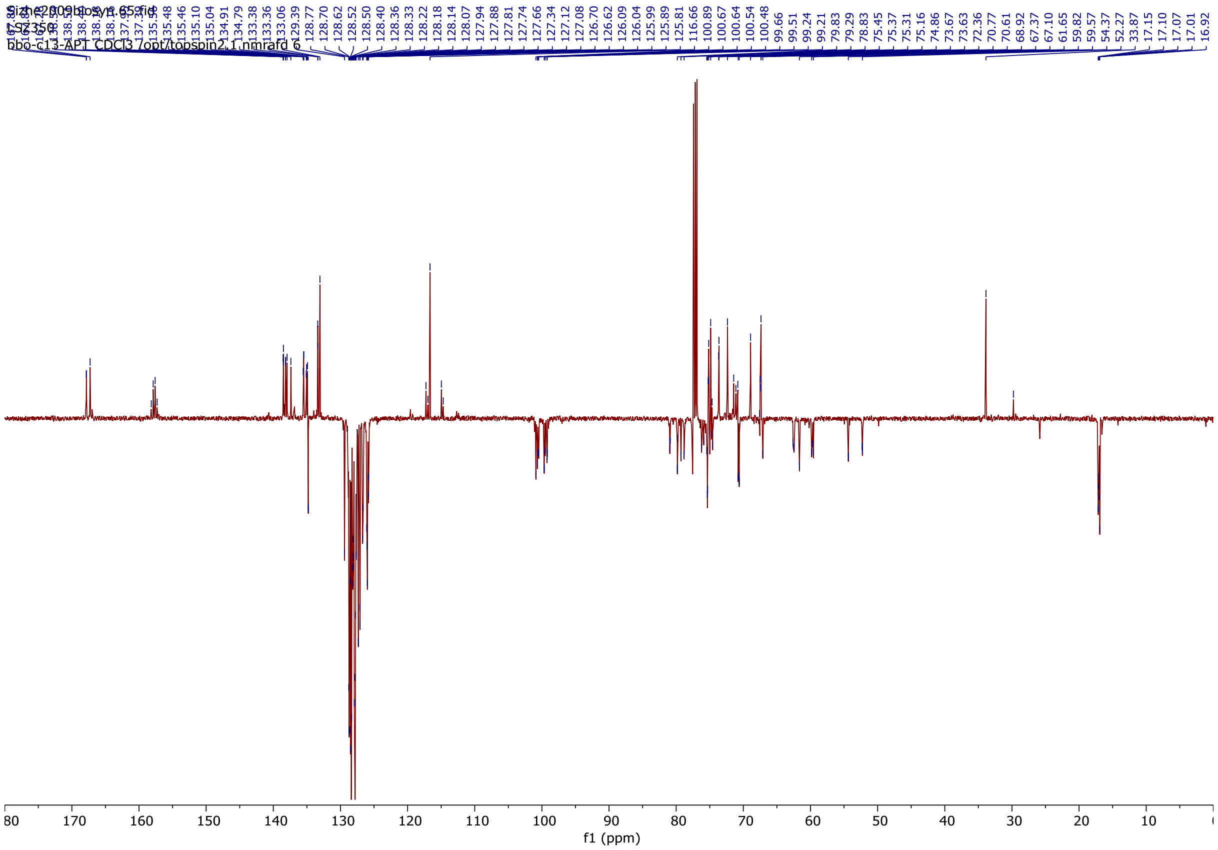
LSZ350
bbo-c13-APT CDCl3 /opt/topspin2.1 nmrafd 6
129.39
128.77
128.70
128.62
128.52
128.50
128.40
128.36
128.33
128.22
128.18
128.14
128.07
127.94
127.88
127.81
127.74
127.66
127.34
127.12
127.08
126.70
126.62
126.09
126.04
125.99
125.89
125.81
116.66
100.89
100.67
100.64
100.54
100.48
99.66
99.51
99.24
99.21
79.83
79.29
78.83
75.45
75.37
75.31
75.16
74.86
73.67
73.63
72.36
70.77
70.61
68.92
67.37
67.10
61.65
59.82
59.57
54.37
52.27
33.87
17.15
17.10
17.07
17.01
16.92
f1 (ppm)

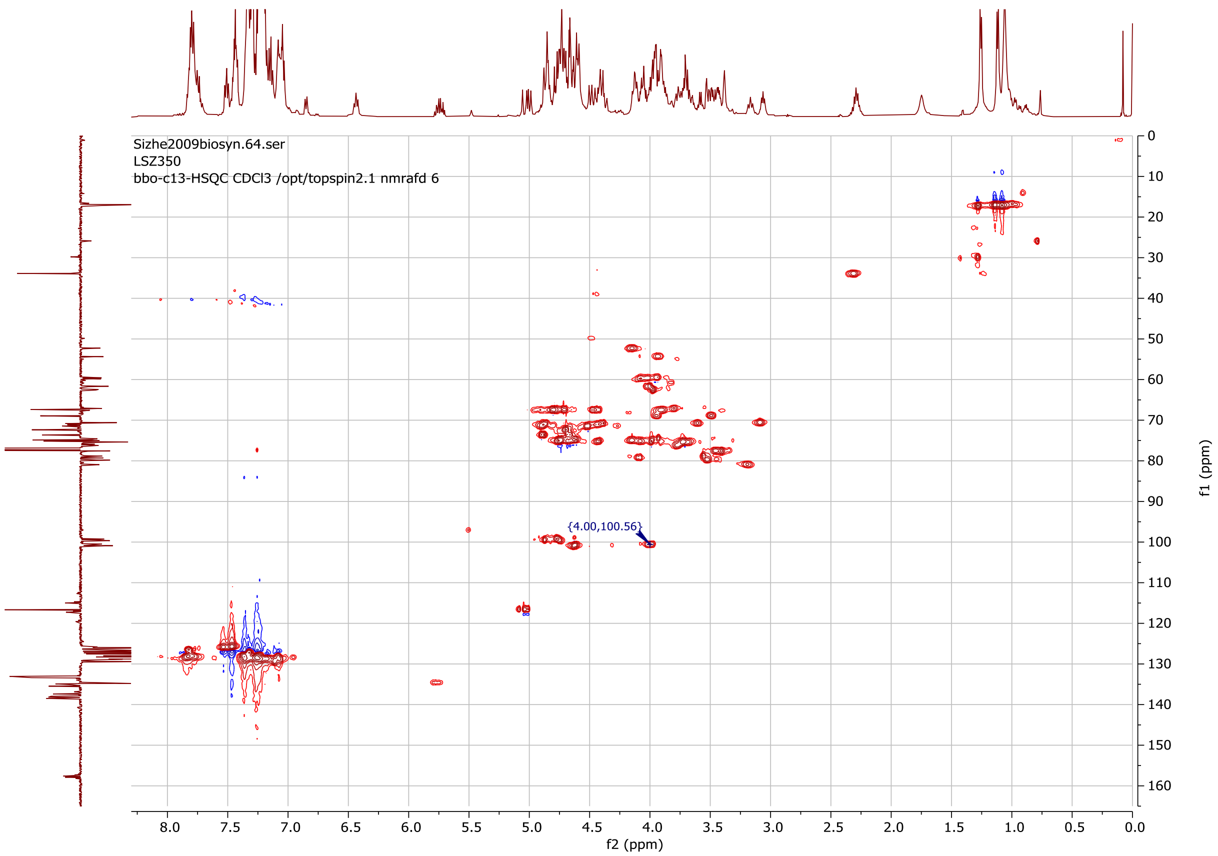
Sizhe2009biosyn.64.ser
LSZ350
bbo-c13-HSQC CDCl3 /opt/topspin2.1 nmrafd 6
{4.00,100.56}
f1 (ppm)
f2 (ppm)

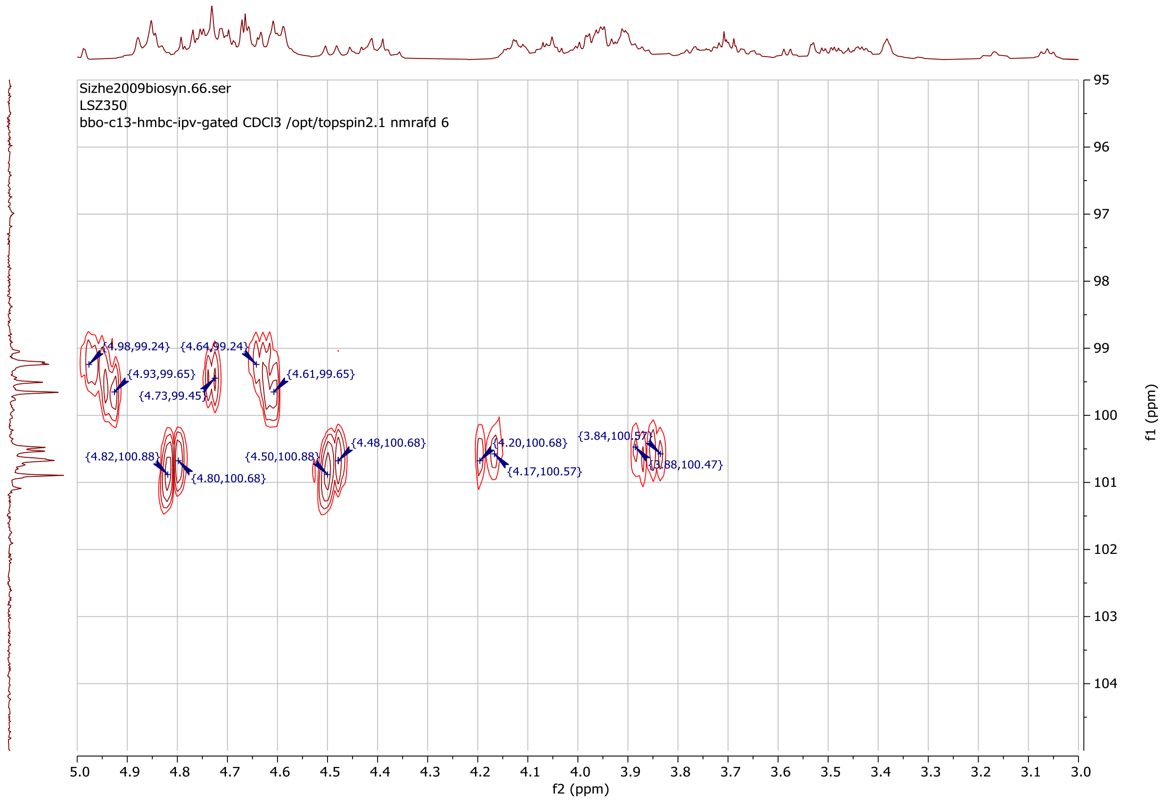

CP5-*O*-Ac-Nonasaccharide (3)

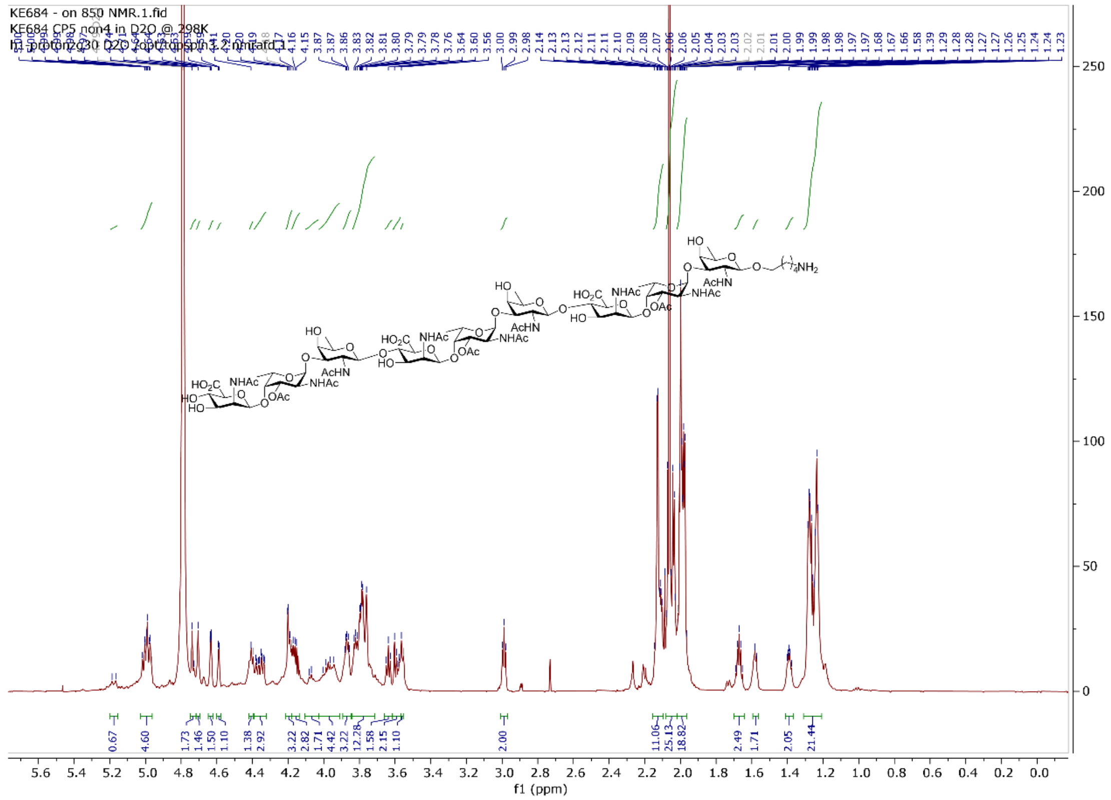

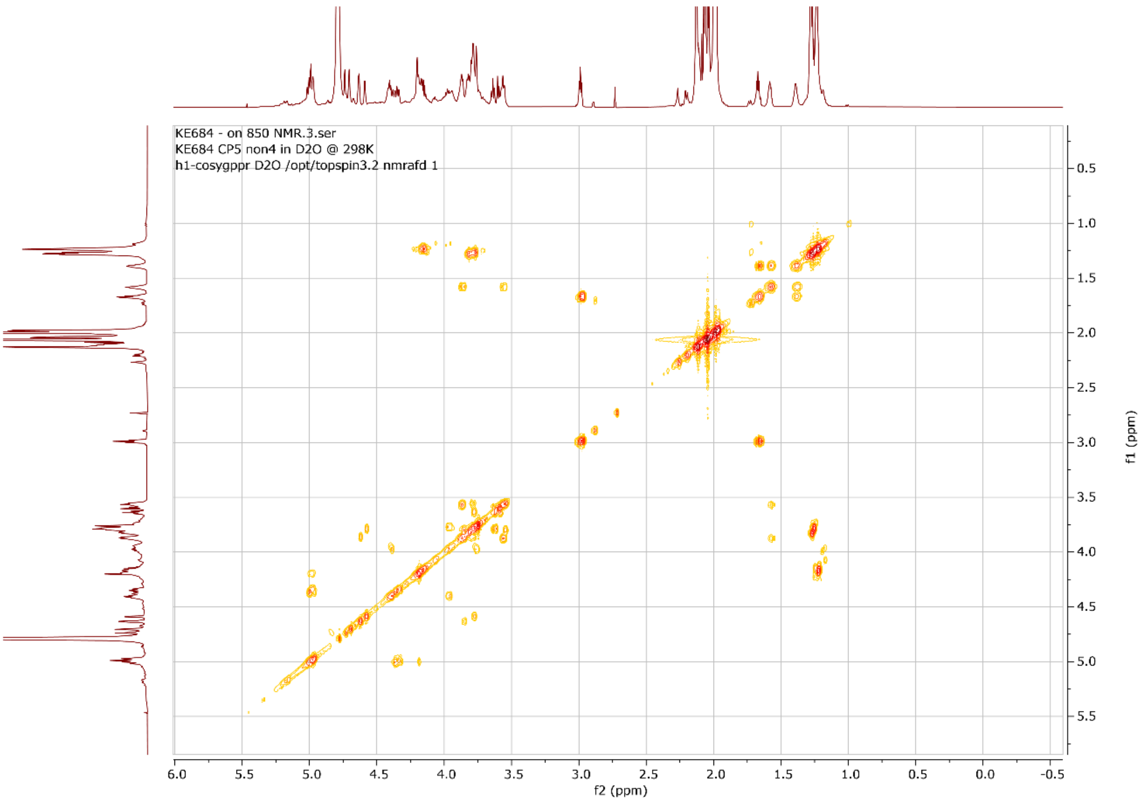
KE684 - on 850 NMR.3.ser
KE684 CP5 non4 in D2O @ 298K
h1-cosygppr D2O /opt/topspin3.2 nmrafd 1
f2 (ppm)
f1 (ppm)

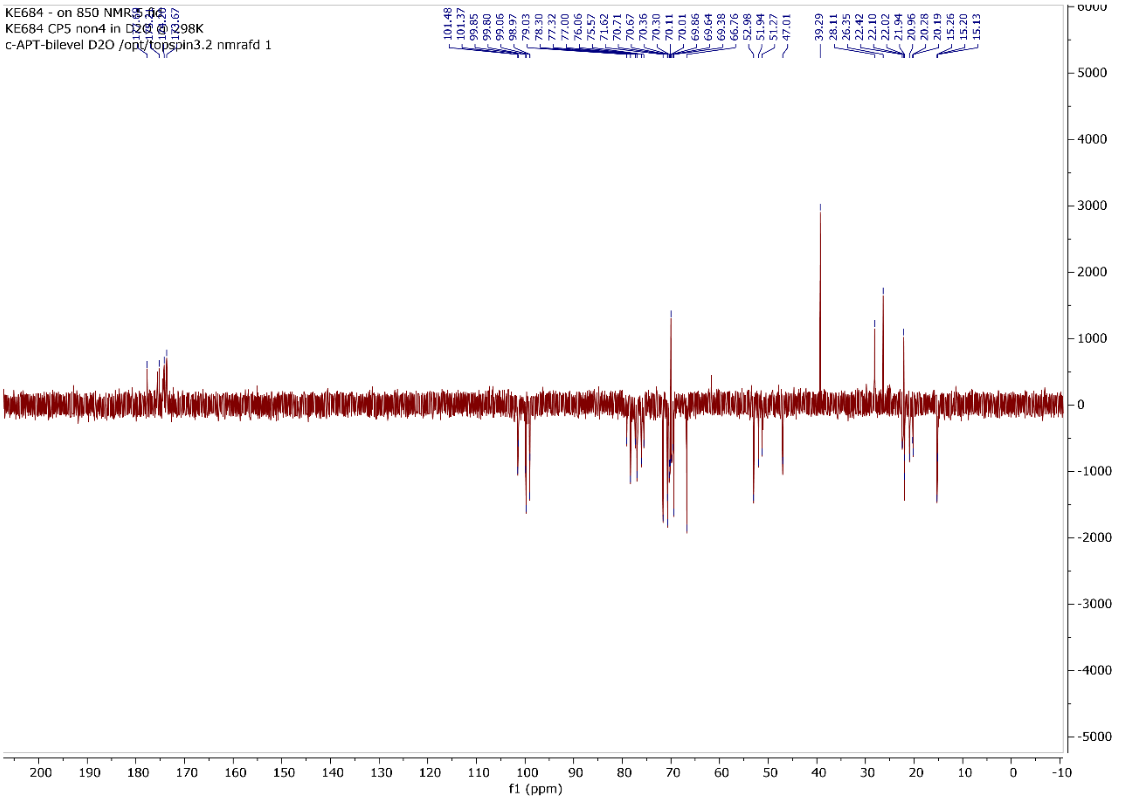
KE684 - on 850 NMR.5.fid
KE684 CP5 non4 in D2O @ 298K
c-APT-bilevel D2O /opt/topspin3.2 nmrafd 1
173.67
101.48 101.37 99.85 99.80 99.06 98.97 79.03 78.30 77.32 77.00 76.06 75.57 71.62 70.71 70.67 70.36 70.30 70.11 70.01 69.86 69.64 69.38 66.76 52.98 51.94 51.27 47.01 39.29 28.11 26.35 22.42 22.10 22.02 21.94 20.96 20.28 20.19 15.26 15.20 15.13
f1 (ppm)

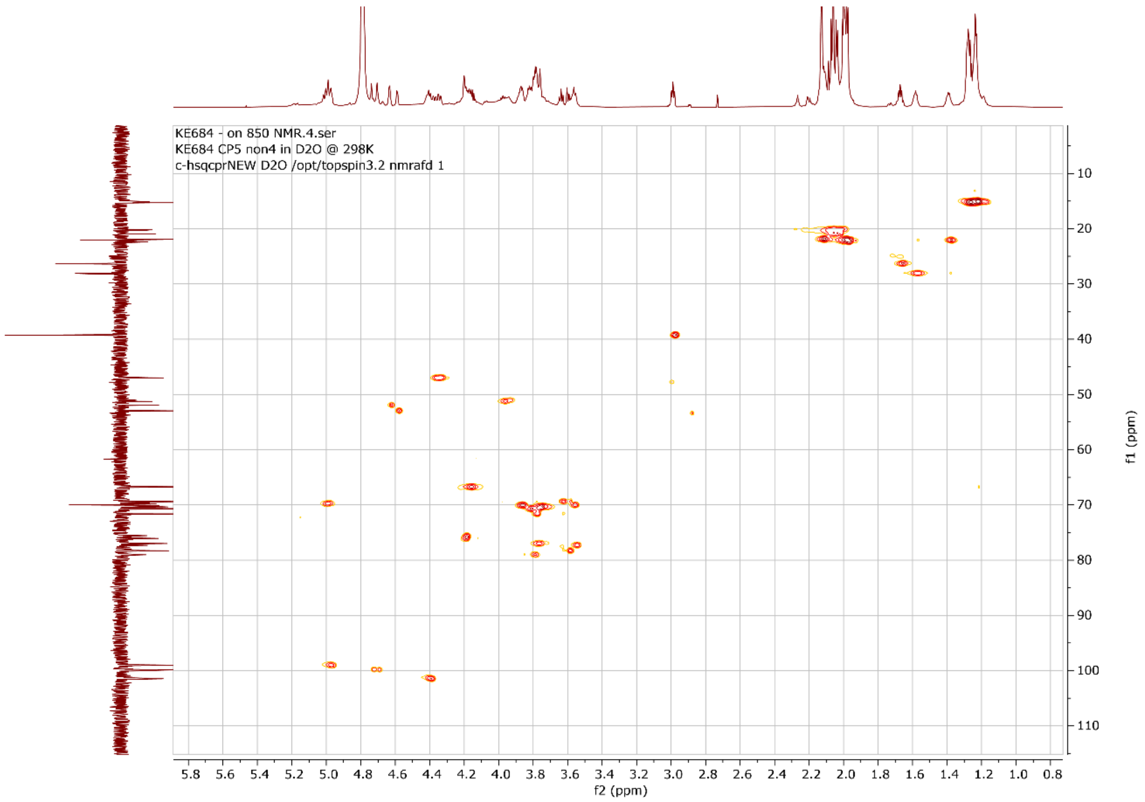

CP5-de-*O*-Ac-Nonasaccharide (4)

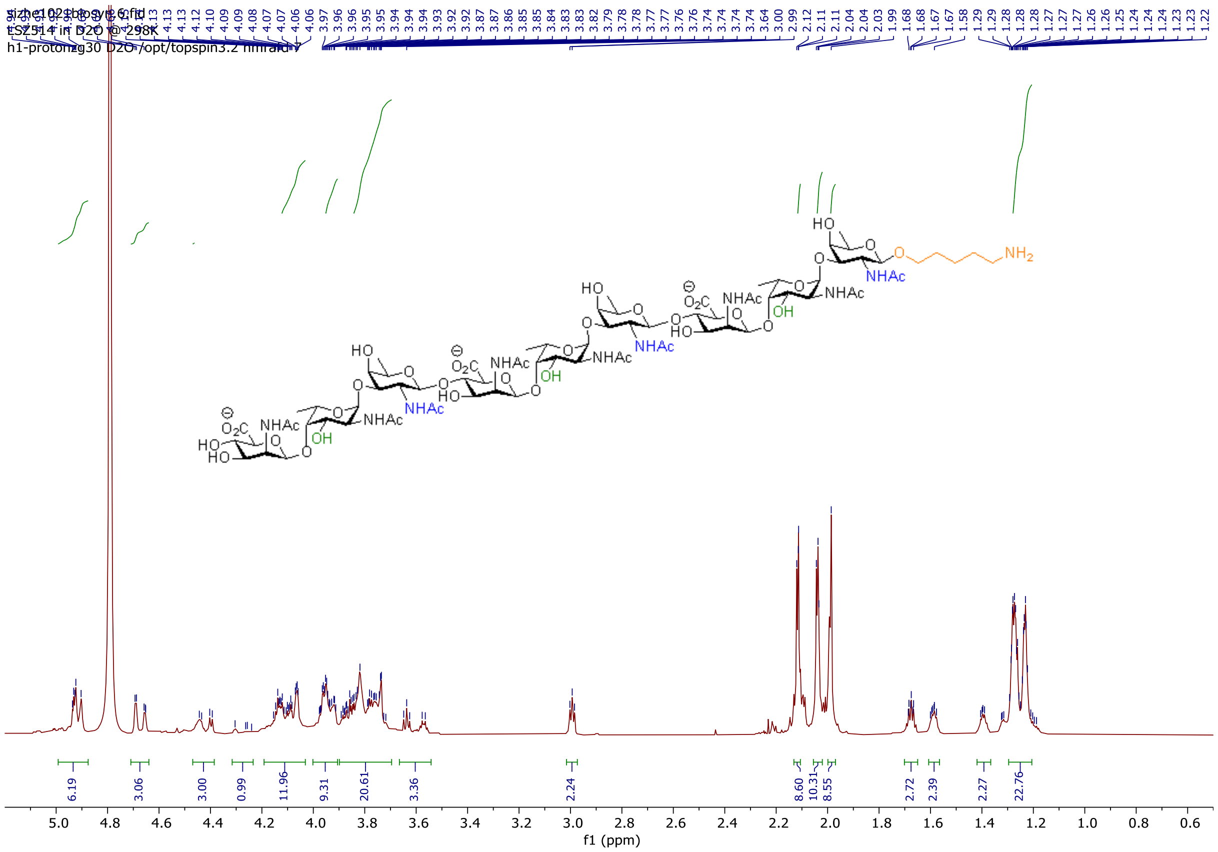

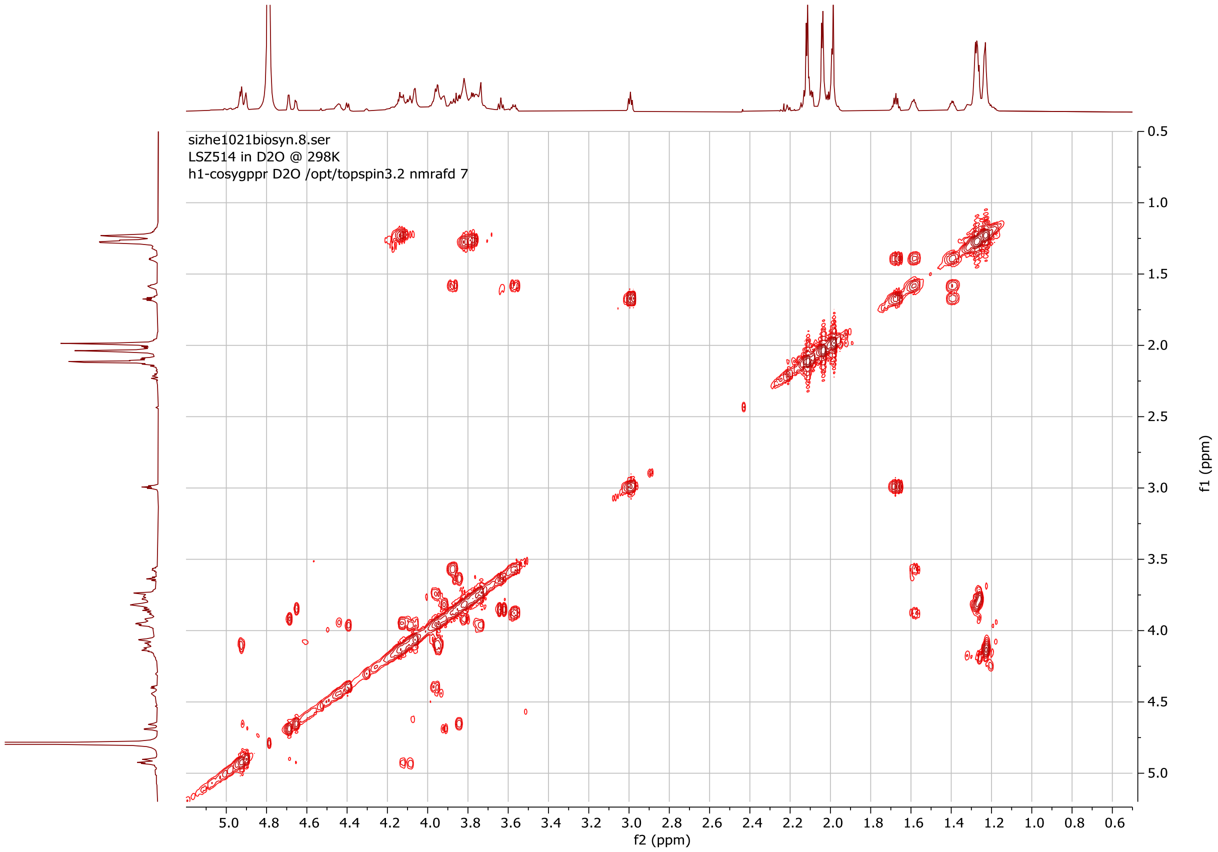
sizhe1021biosyn.8.ser
LSZ514 in D2O @ 298K
h1-cosygppr D2O /opt/topspin3.2 nmrafd 7
f2 (ppm)
f1 (ppm)

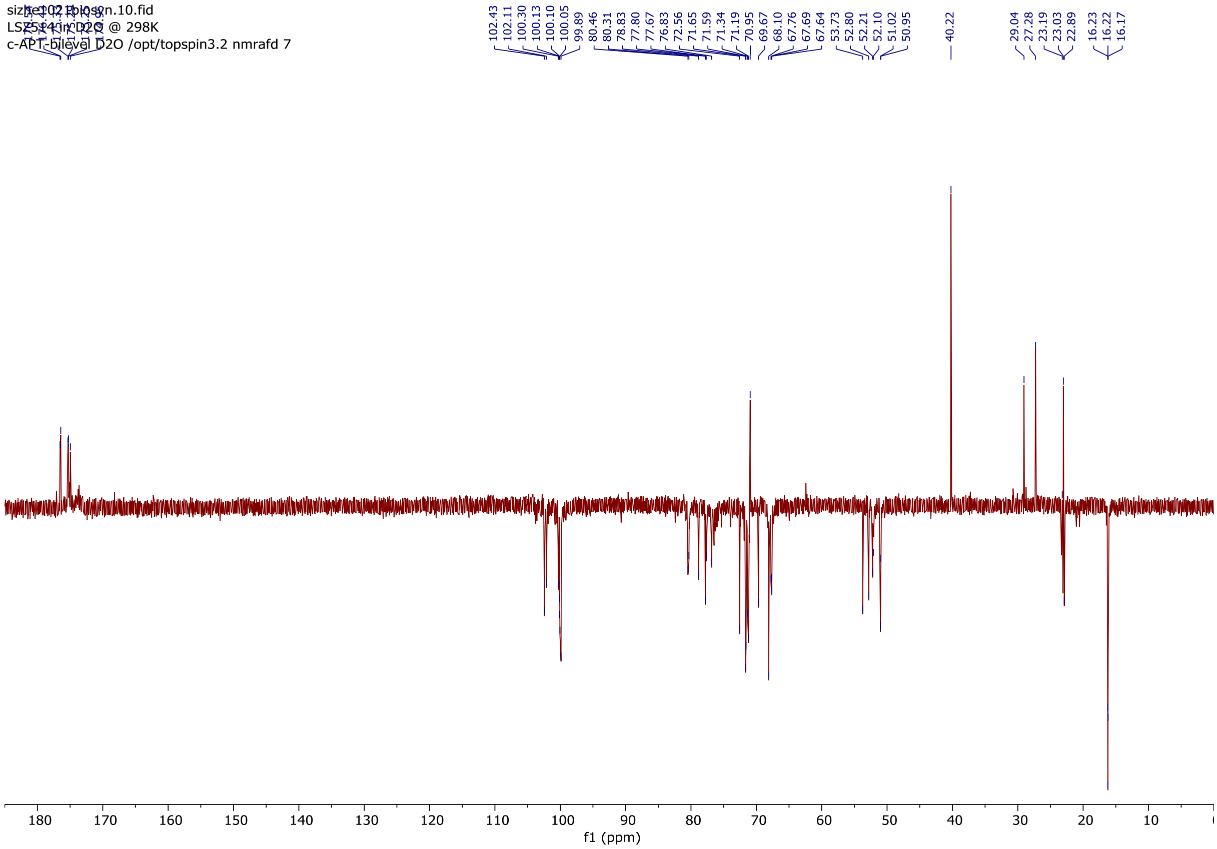
sizhe1021biosyn.10.fid
LSZ514 in D2O @ 298K
c-APT-bilevel D2O /opt/topspin3.2 nmrafd 7
102.43
102.11
100.30
100.13
100.10
100.05
99.89
80.46
80.31
78.83
77.80
77.67
76.83
72.56
71.65
71.59
71.34
71.19
70.95
69.67
68.10
67.76
67.69
67.64
53.73
52.80
52.21
52.10
51.02
50.95
40.22
29.04
27.28
23.19
23.03
22.89
16.23
16.22
16.17
f1 (ppm)

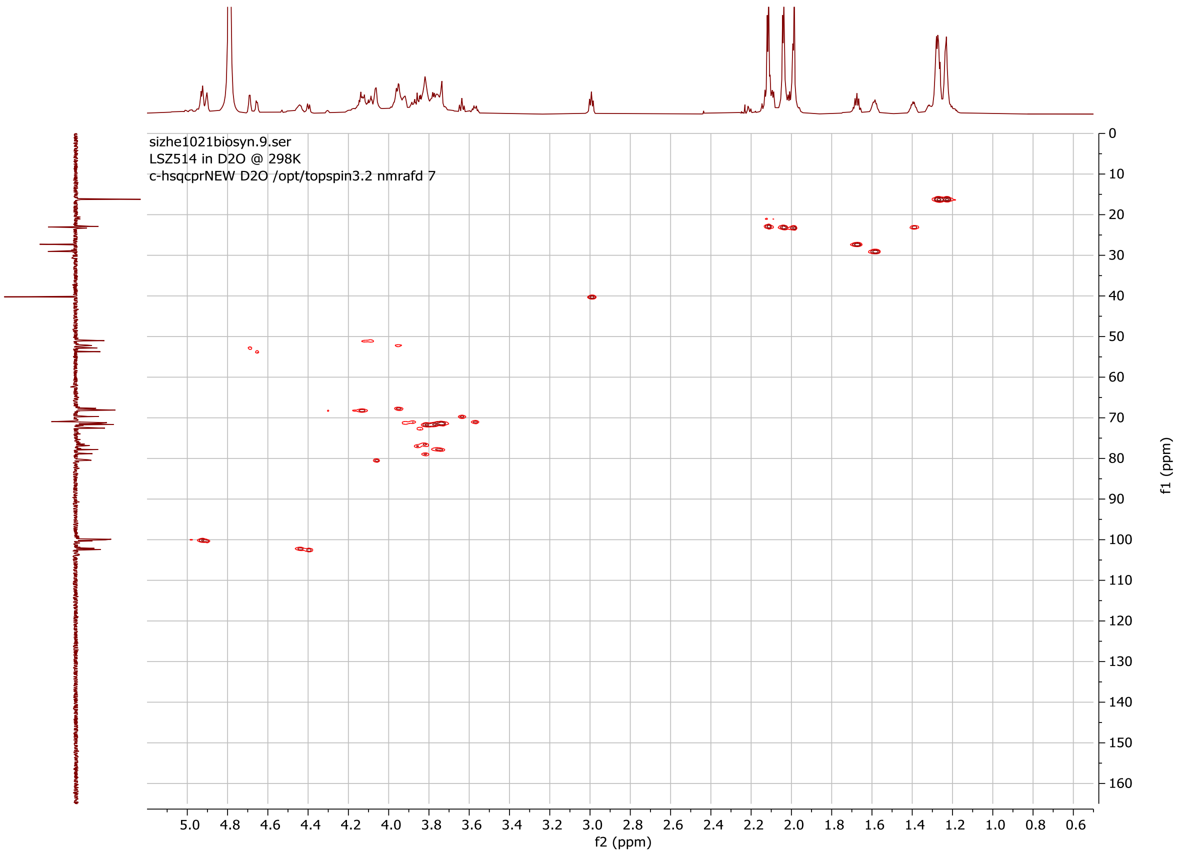

CP5-Zwiterionic-Nonasaccharide (5)

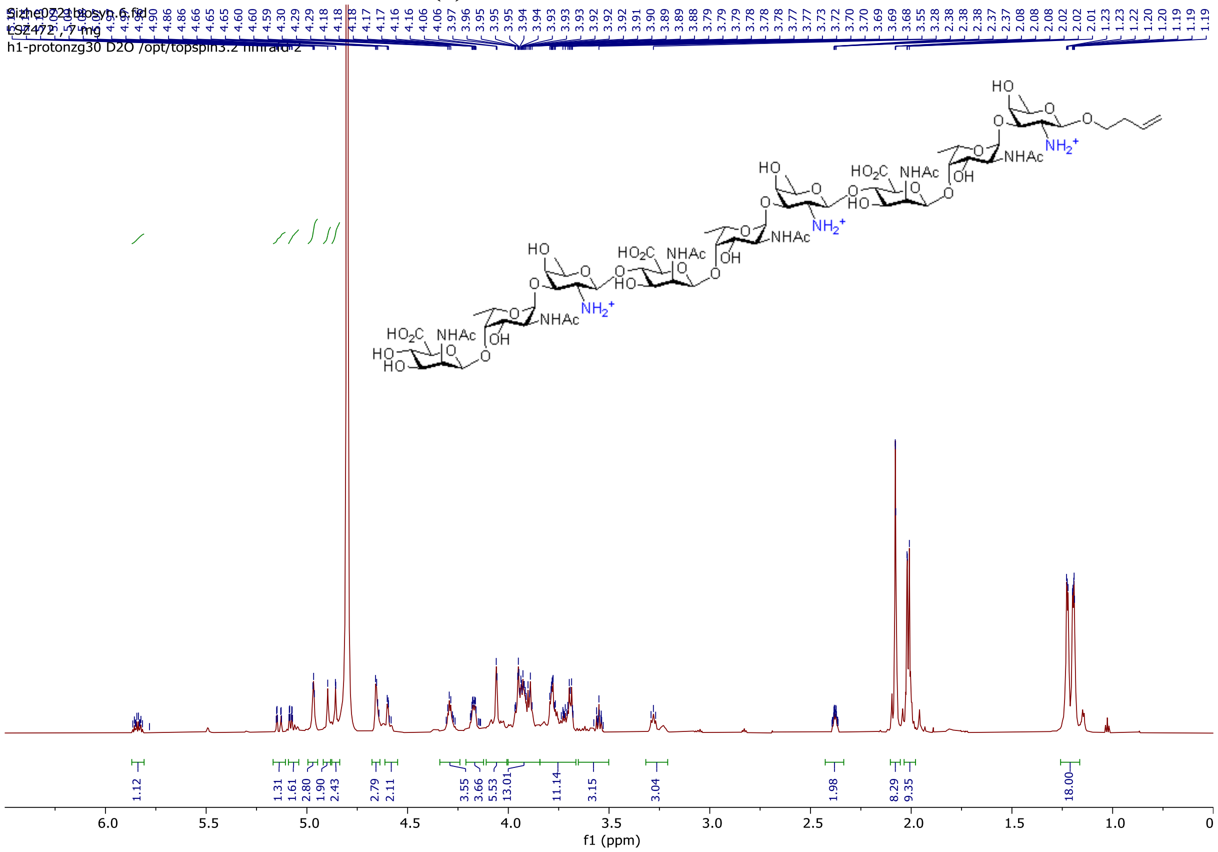

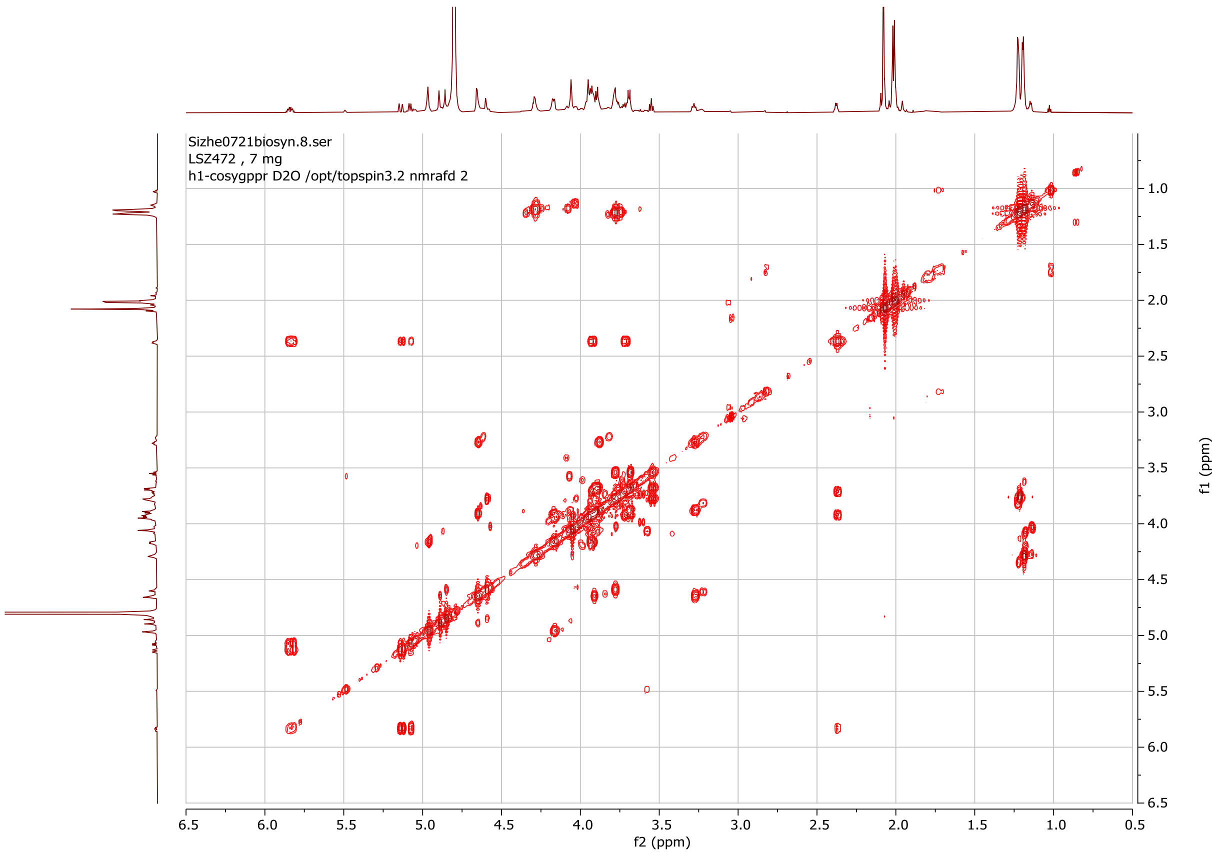
Sizhe0721biosyn.8.ser
LSZ472 , 7 mg
h1-cosygppr D2O /opt/topspin3.2 nmrafd 2
f2 (ppm)
f1 (ppm)

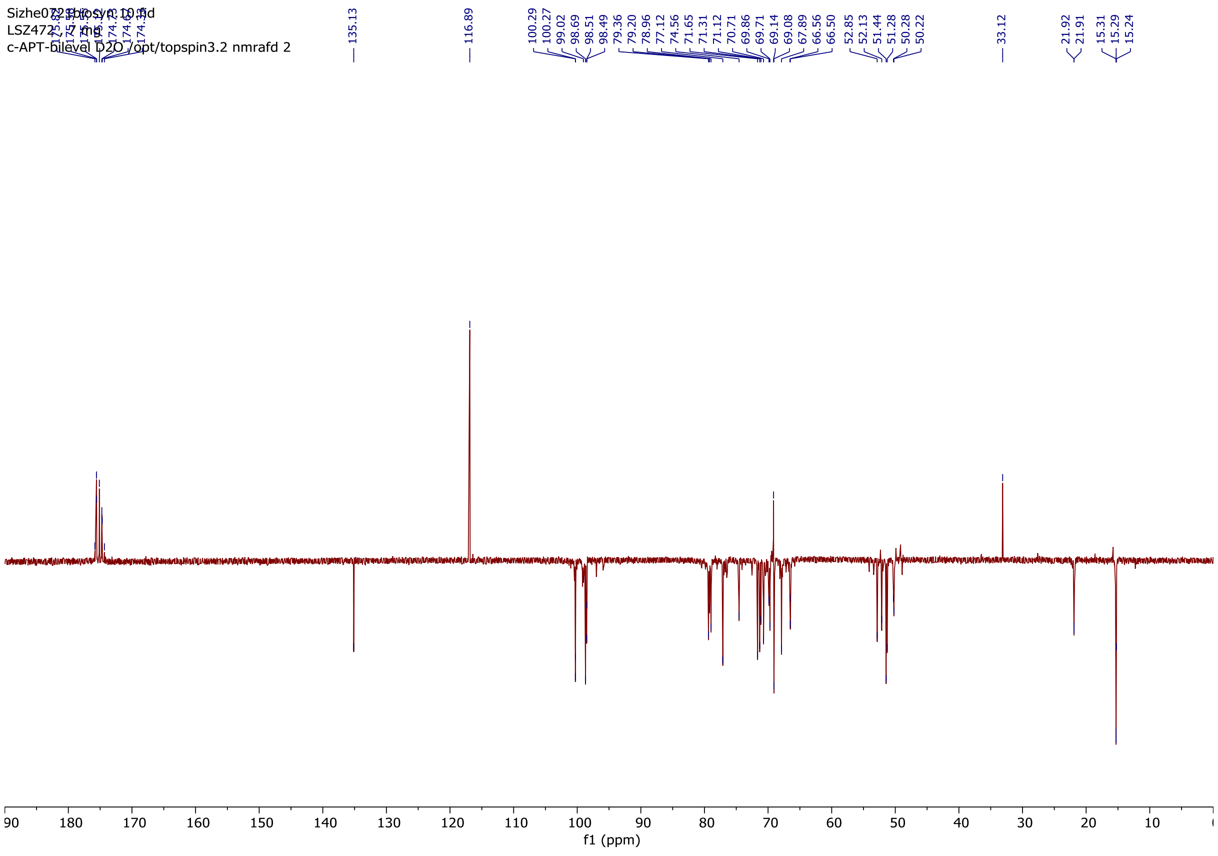
Sizhe0721biosyn.10.fid
LSZ472 , 7 mg
c-APT-bilevel D2O /opt/topspin3.2 nmrafd 2
135.13
116.89
100.29
100.27
99.02
98.69
98.51
98.49
79.36
79.20
78.96
77.12
74.56
71.65
71.31
71.12
70.71
69.86
69.71
69.14
69.08
67.89
66.56
66.50
52.85
52.13
51.44
51.28
50.28
50.22
33.12
21.92
21.91
15.31
15.29
15.24
f1 (ppm)

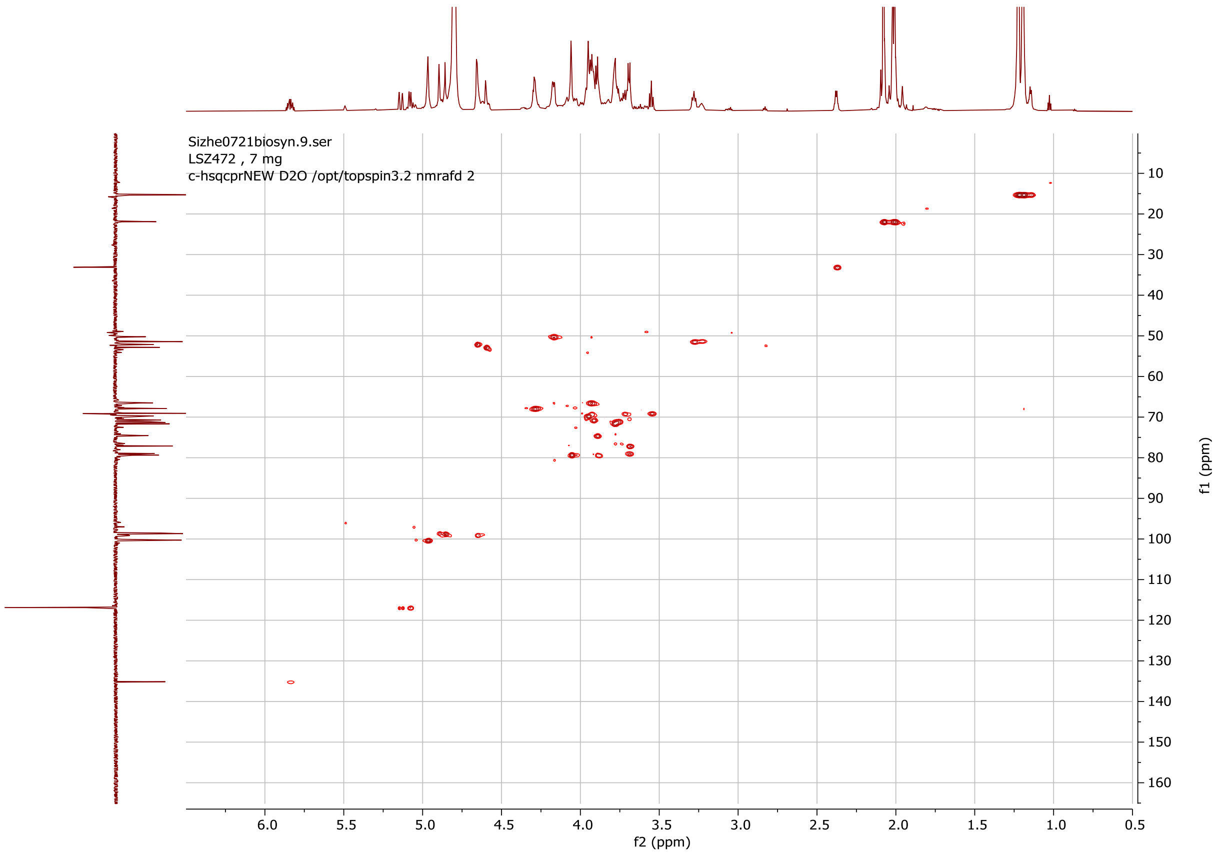
Sizhe0721biosyn.9.ser
LSZ472 , 7 mg
c-hsqcprNEW D2O /opt/topspin3.2 nmrafd 2
f2 (ppm)
f1 (ppm)
